# A model to explain specific cellular communications and cellular harmony:- a hypothesis of coupled cells and interactive coupling molecules

**DOI:** 10.1186/1742-4682-11-40

**Published:** 2014-09-14

**Authors:** Cyril J Craven

**Affiliations:** 1 Queensland University of Technology (QUT), Brisbane, Australia

## Abstract

**Background:**

The various cell types and their relative numbers in multicellular organisms are controlled by growth factors and related extracellular molecules which affect genetic expression pathways. However, these substances may have both/either inhibitory and/or stimulatory effects on cell division and cell differentiation depending on the cellular environment. It is not known how cells respond to these substances in such an ambiguous way. Many cellular effects have been investigated and reported using cell culture from cancer cell lines in an effort to define normal cellular behaviour using these abnormal cells.

A model is offered to explain the harmony of cellular life in multicellular organisms involving interacting extracellular substances.

**Methods:**

A basic model was proposed based on asymmetric cell division and evidence to support the hypothetical model was accumulated from the literature. In particular, relevant evidence was selected for the Insulin-Like Growth Factor system from the published data, especially from certain cell lines, to support the model. The evidence has been selective in an attempt to provide a picture of normal cellular responses, derived from the cell lines.

**Results:**

The formation of a pair of coupled cells by asymmetric cell division is an integral part of the model as is the interaction of couplet molecules derived from these cells. Each couplet cell will have a receptor to measure the amount of the couplet molecule produced by the other cell; each cell will be receptor-positive or receptor-negative for the respective receptors. The couplet molecules will form a binary complex whose level is also measured by the cell. The hypothesis is heavily supported by selective collection of circumstantial evidence and by some direct evidence. The basic model can be expanded to other cellular interactions.

**Conclusions:**

These couplet cells and interacting couplet molecules can be viewed as a mechanism that provides a controlled and balanced division-of-labour between the two progeny cells, and, in turn, their progeny. The presence or absence of a particular receptor for a couplet molecule will define a cell type and the presence or absence of many such receptors will define the cell types of the progeny within cell lineages.

## A model of life

A simple model is offered to explain the requisite harmony of multicellular life. From this basic model, complexity needs to be added to explain the abundance, profusion and variety of life and the sophistication of human existence.

The adult worm *Caenorhabditis elegans* has exactly 959 cells in the hermaphrodite, having lost exactly 131 defined cells by apoptosis and fusion during ontogenesis [1,2]. Could we expect the same organised, awe-inspiring exactitude of proliferation, differentiation, apoptosis etc. for a human with 50–100 × 10^12^ cells? The current model offers the reciprocal interactions of coupled cells which have been derived from asymmetric cell division, as the basis for this exactitude of multicellular life.

## (A) Background:- questions within existing knowledge

The model offered here relates to the regulation of cell division by extracellular messages and relates to questions as to when and why a growing cell decides to divide symmetrically or asymmetrically and what particular type of symmetric or asymmetric division occurs.

### When will a cell proliferate, differentiate or apoptose or otherwise live or die?

#### Chemical messages will be an integral part of this decision-making

A ***unicellular*** organism is a fully-armoured, selfish, intelligent cell. It is often in an anarchistic milieu, an unpredictable and fickle environment [3], within which it needs to respond appropriately to an array of gross changes and predicaments which are monitored by the cell for the cell’s sole information. In a favorable environment, an increase in effector molecules (e.g. nutrients) will induce appropriate enzymes for their own catabolism, thus to increase metabolism from quiescence [4], perhaps to increase cell size (hyperplasia) and perhaps to proliferate by cell division. In an adverse environment, with nutritional deprivation, senescence [5] or sporulation may be the response.

**Figure 1:**
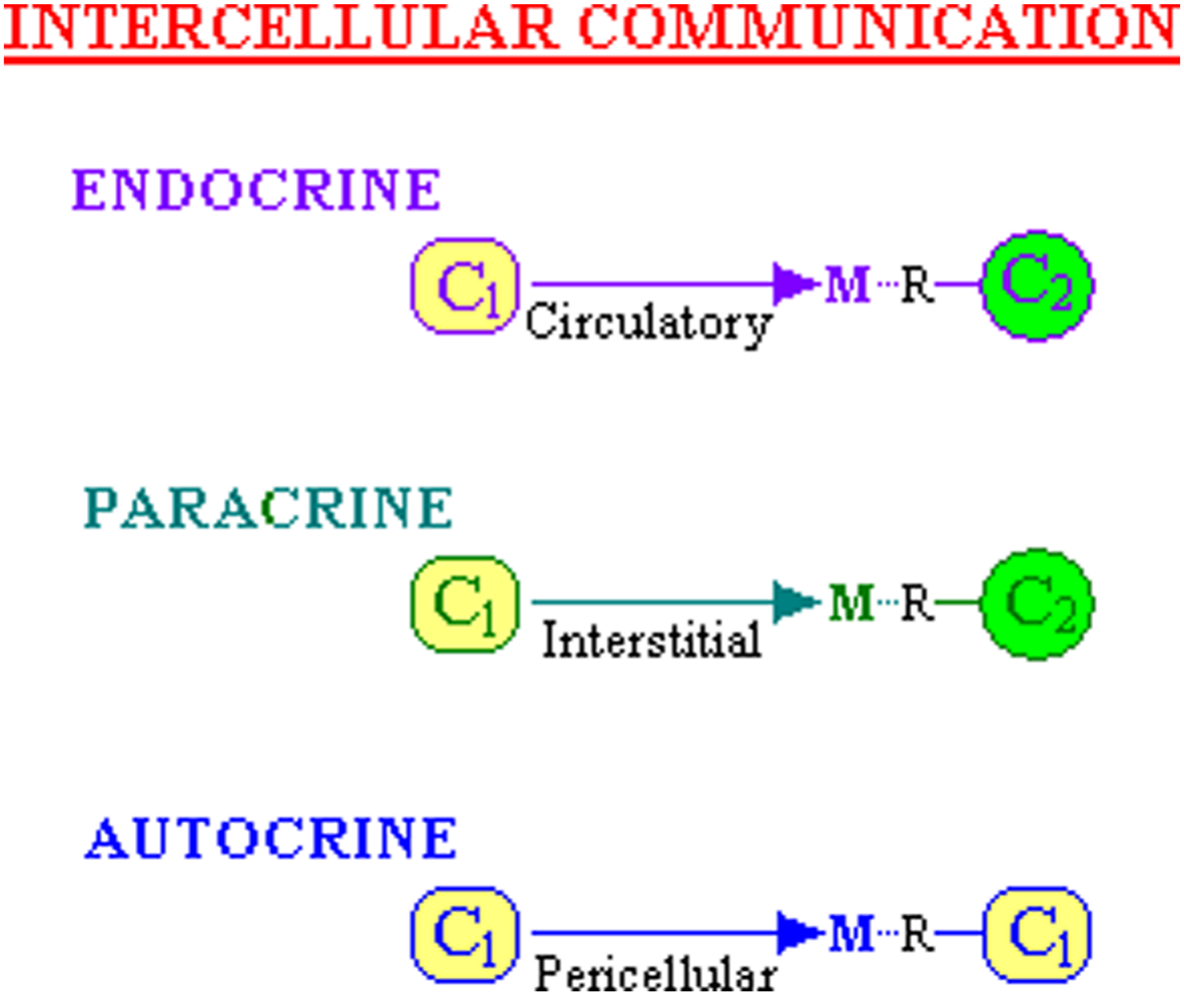
**Types of communication between cells.***This illustrates the terms used to describe communication between cells (C*_*1*_*and C*_*2*_*are cells) where a message (M), carried by a molecule (eg. a Ligand) released from one cell into the external environment, is received by a receptor (R) either on another type of cell or on a cell of its own kind. Each term reflects the distance that the message/signal has to travel and is dependent on the separation of the cells involved - between tissues (Circulatory) or within a tissue (Interstitial or Pericellular). The Figure does not illustrate the other possible communication mechanism, Homocrine and Juxtacrine, which are discussed in the main text.*

In a complex ***multi-cellular***, ***multi-organ*** organism (e.g. an animal), all cells have intelligence from which coordinated growth emanates, and in order to monitor the environment, certain specialised cells exist. Some cells possess radiation detectors which will be interpreted as sight or sound while other cells have chemical receptors used to transduce information as smell, touch/pressure or taste. Indeed, virtually all cells are functionally specialised, labour is thus divided, co-operation is inherent and the environment of each cell is more controlled; nutrients are generally available and ambient changes are normally more subtle than for a less developed organism. However, in order to coordinate whole body function, each cell will be exposed to a variety of secreted metabolic messages from other body cells, some of which may lead to cellular proliferation or to another response. Of these secreted messenger molecules, there will be specific messages derived from distant, differentiated cells e.g. of the nervous, immune and endocrine systems. Within a specific organ or tissue, together with these messages from perhaps metres away, there will also be messages from other differentiated cells, perhaps centimetres away (the paracrine system), along with messages from identical self cells (i.e. cells which contain identically active genetic machinery) perhaps millimetres away (the autocrine system) (See Figure 1).

If an Autocrine interaction is considered to be the reception of a ligand signal produced by exactly the same cell, then a Paracrine interaction could be considered to be either Homotypic or Heterotypic, where a Homotypic one refers to the reception of the ligand signal by a cell of the same type that produced it. Thus this would be referred to as a Homocrine interaction and the Heterotypic Paracrine interaction referred to as simply Paracrine [6].

A Juxtacrine interaction is a more intimate type of cellular communication where the chemical message is passed between two cells that are in physical contact with each other, being received directly or via secreted extracellular matrix (ECM). It involves a receptor on one cell and a ligand from another cell which is anchored or fixed as part of the cell itself [7]. (If the communication is bidirectional, then perhaps both act as ligand and receptor simultaneously – each perhaps a ligeptor). Another interaction is via intercytoplasmic conduits, “Gap junctions”, which are channels made of connexin proteins between neighbouring cells, allowing small-molecule exchange and perhaps signalling [8]. Further, there are reciprocal/bidirectional communications as observed in pre-and post-synaptic chemical signalling in neurons [9], and with inside-out and outside-in signalling via integrins [10]. The focus of this discussion is on chemical interactions of ligand and receptor between cells and the consequent intelligent use of these messages.

#### Reception and interpretation of chemical messages will give intelligence to cells for this decision-making

Within tissues and organs, these messenger molecules will often be present within the extracellular matrix which, while organised, will also contain a veritable soup of metabolic intermediates, end-products of metabolism, nutrients, vitamins, false messengers from foreign cells or invasive molecules that would redirect the genetic manifestation of that cell and, in some circumstances, other molecules which may be toxic to metabolism - lytic or necrotic.

In order to respond selectively to the coordinating messages from self-type cells, the target cell needs specific cell-membrane receptors which will receive only the specific signal for that cell type. The cell can then interpret each message accurately via coupling of the receptor to intracellular molecules which transmit the extracellular signal to the nucleus, and thus the cell can respond appropriately. The cell exists “in the dark” and can only gain knowledge of the extracellular environment by receptors. The receptor-mediated response may be a signal to induce a change in protein synthesis to alter metabolic reactions with adaptation and/or to cause hyperplasia, migration, proliferation, transformation, differentiation, apoptosis or other. The signal may be necessary but not sufficient and the cell may only respond provided other requirements are met. A message received or interpreted falsely may lead to a cell not synchronized or not in harmony with its neighbours or it may cause the cell to transform to one which is uncontrolled in its growth, to the detriment of the whole organism.

Membrane receptors translate the external environment to cause intracellular reaction and a specific set of receptors will control and define the inherent properties of a cell. Indeed, cells have long been identified by cell markers - proteins on the cell membrane - many of which are receptors. For example, the Cluster of differentiation (CD) is a group of membrane proteins used to define immunologically the cell surface molecules of blood cells, especially white blood cells. Other cell markers also exist e.g. Lin (a marker used to detect lineage commitment), Sca (Stem cell antigen), c-kit (the receptor for stem cell factor) The presence or absence of the various CD proteins and other markers that act as receptors, ligands, enzymes or adhesion molecules, is used to identify specific cells. For example, two subsets of murine pluripotent hematopoietic stem cells exist, one with the phenotype Lin(-) Sca(+) kit(+) CD38(+) CD34(-), the other Lin(-) Sca(+) kit(+) CD38(-) CD34(+) [11]. While there are over 300 CD proteins already detected, it has been estimated that there would be between 2,400 and 5000 cell-surface molecules on leucocytes [12]. Many of these would be receptors. A cell is then defined by these receptors it uses to receive messages and to appropriately respond. While the number of molecules of particular receptors might vary with adaptation or maturation of a cell, a major change in the types of cell-membrane receptors is considered here to indicate a change of the cell’s type and the change in receptors would be a critical part of a differentiation process. (See Additional file 1,1 *“Terminology”* for Definitions). Cells are then defined by their receptors and their messages will control both the metabolic pathways of the cell and the cell’s decision to “proliferate, differentiate or apoptose or otherwise live or die”. Receptor-mediated uptake of signal-carrying molecules is an integral part of the model presented herein.

A molecule may be a signal with a message but it would only be so “in the eyes of the beholder”; the recipient cell (i.e. a cell whose status makes it receptive to the message at that time) must have an ability to interpret the exact message based on the both the molecule’s structure and its concentration over a period of time. For the latter to occur, the cell needs to measure this concentration by comparison against the concentration of a relevant, related molecule. In other words, the cell can “count” but it likely uses ratios of concentrations rather than absolute numbers. This intelligent use of this information received resides in all cells allowing both local and whole-of-body decisions to be made and appropriate changes to ensue. In multicellular organisms, local decisions are supervised by cells that reside within the specialised and integrated cells (e.g. within the brain) which produce the whole-of-body intellect. For Insulin-Like Growth Factor-I (IGF-I), this growth factor may be either locally produced to have a paracrine action [13] or it may be derived from hepatocytes that secrete it into the systemic system under the control of pituitary-derived growth hormone (GH). In turn, GH secretion is controlled by the GH-releasing-hormone released from the hypothalamus to stimulate the pituitary.

Information may then be transferred via receptor-mediated uptake of growth factors, cytokines, adipokines, chemokines, hormones and other bioactive substances. As a group, these are referred to herein as generic *Information-Carrying Molecules and Inter-Cellular Messengers* (ICMs) (See Additional file 1,1 *“Terminology”*).

Simply put, the information carried by ICMs derived from one cell type will be interpretated by a second cell via specific receptors and consequent intracellular changes, thus providing intelligence to this second cell.

### What were the transition steps in the evolution from a unicellular entire (e.g. a Protozoa) to an organised but internally symbiotic multicellular organism (e.g. a metazoan)?

The development of the original eukaryotic cell types may have been based on asymmetric cell division and on common cellular interactions [14]. The former underlies the fundamental basis for the developmental evolution of organisms and for the functioning of totipotent or multipotent stem cells [15]. An example of the latter is that the same molecules (e.g. phorbol esters, diacylglycerol, tetrapyrroles) that stimulate cell division in two unicellular eukaryotes (the ciliate *Tetrahymena thermophila* and the yeast *Saccharomyces cerevisiae*) also cause cell division and other activities in multicellular mammalian cells [16].

#### Asymmetric cell division, with division of labour, is likely a part of this transition process

Cooperativity is part of earliest life, where some cells of a unicellular group reacted to the environment and evolved, with cell division, to cooperate metabolically with their predecessors. (See Additional file 1,2 *“Change in Cellular Characteristics with or without Differentiation (with Cell Division), with Division of Labour*” for examples of this). Asymmetric cell division is considered to be an extension of this, to separate clearly the metabolic labour of survival and growth. A cell community becomes more efficient by allocating some tasks to specific cells; each cell does not then need to utilise its whole armamentarium of genes.

Put another way, one early evolutionary step could have involved a purposeful asymmetric division of a unicellular life form, whereby some of the metabolic labour was then divided between two cell types (initially). These two, coupled cells and their progeny from subsequent symmetric cell division, would need to form a mutualism wherein each couplet cell of one type would need to know of the combined metabolic activity state of the other couplet cells. This could be achieved by a molecular signal generated by each couplet being received by the partner via a specific receptor. This latter scenario would be similar to the situation in the yeast *Saccharomyces cerevisiae*, where there are two types of haploid cells, the alpha-cell and the a-cell, whose formation involves asymmetric cell divisions. The alpha-cell secretes the alpha-factor which binds to a specific receptor on a-cells to transmit a signal and, in a reciprocal way, the a-cell secretes an a-factor which binds to a receptor on alpha-cells. Each factor or pheromone induces hyperexpression of genes specific for the opposite cell type [17]. A similar system occurs in other fungi [18].

These examples in fungi and the following example of pheromones, are the early prototypes of the current “*CTC Model*” proposed in Section B of this article, involving asymmetric cell division (AsCD) with reciprocal interactions.

**Figure 2:**
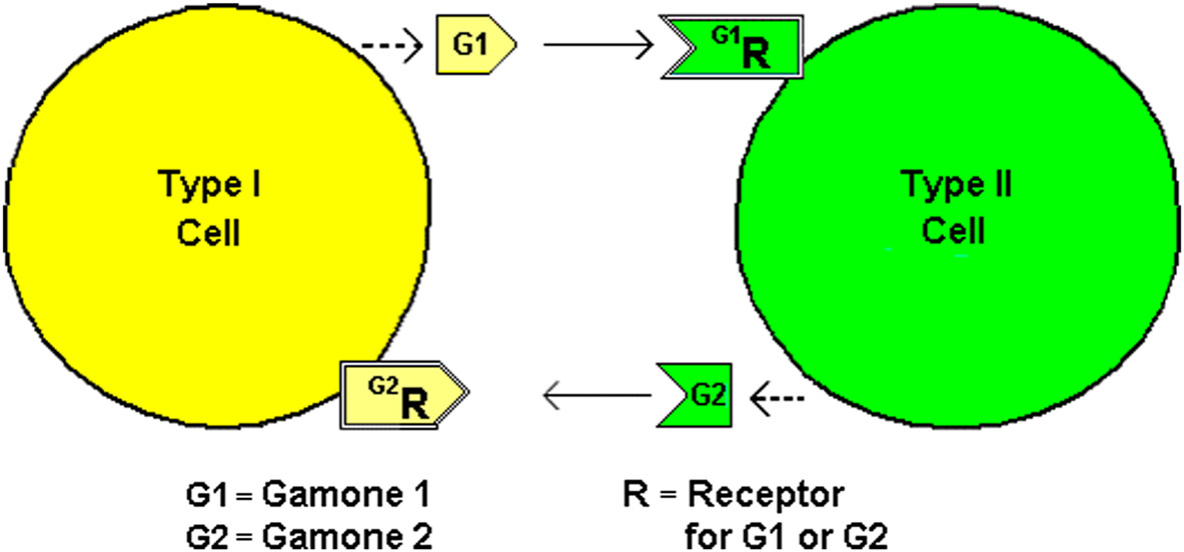
***Blepharisma japonicum*** **mating cells.***Types I and II cells secrete Gamone 1 and Gamone 2 respectively. Gamone 1 (blepharmone) is recognized by a putative Gamone 1 receptor on Type II cells and Gamone 2 (blepharismone) is recognized by a putative Gamone 2 receptor on Type I cells.*

Pheromones, also known as Gamones, are secreted by gametes for sexual reproduction. In an ancestral ciliate, *Blepharisma japonicum,* two mating cells (Types I and II) are formed as offspring cells from AsCD [19]. Type I cells secrete Gamone 1 (a glycoprotein) and Type II cells secrete Gamone 2 (a trp derivative) and it is thought that Gamone 1 is recognized by putative Gamone 1 receptors on Type II cells and Gamone 2 is recognized by putative Gamone 2 receptors on Type I cells [20] (See Figure 2). This is a similar reciprocal arrangement as for *S. cerevisiae*. In the ciliate, *Euplotes raikovi,* however, a receptor for the pheromone Er-1 has been identified in Type I cells although the this particular pheromone stimulates cells that produce it i.e. it appears to act autocrinely [21,22]. An alternate explanation, consistent with the model offered herein, would be that the cells are in fact of two types – one producing, the other internalizing the pheromone.

Division of metabolic labour may be the reason for such formation of coupled cells, with AsCD as the mechanism. Many different biological species are syntrophic in that one species lives off the products of another species while others are symbiotic with mutual advantage by interaction between two different species. A third situation appears for a archeal biofilm where two sets of cells, physiologically and possibly genetically differentiated with respect to each other but derived from a single-species, are involved in mutual syntrophic reactions. It seems that one cell type of the archea produces methane from hydrogen (methanogenic) and the other uses the methane to produce perhaps acetate or formate (methanotrophic) which is used by the first methanogenic cell [23]. This could be interpreted as a division of labour (DOL) resulting from a single precursor cell, by AsCD. DOL has also been described in Additional file 1,2 for *Cyanobacteria* and algae but for a more complex multicellular organism, the total sum of catabolic and anabolic reactions for the whole is enormous. Many descriptions of metabolism impose a complex interplay of reactions on a single cell, whereas the evolution of multicellular organisms has been towards specialisation and division of metabolic labour. Indeed early cells were more complex with more diverse constituents than modern cells [3]. The specialisation is evidenced by certain cells of the human body producing metabolites which are released into the extracellular fluid, taken up by other cells via transporters/receptors and used metabolically by these cells. Certain human cells that have stored sources of energy (glycogen and triacylglycerols) release glucose and fatty acids for other cells. Products of metabolism, alanine and lactate, are transferred from muscle to specific liver cells and used for energy or glucose production. Also, lactate is produced in one cell (astrocyte), secreted and taken-up by a paracrine cell (a neuron) for fuel [24] and for long-term memory formation [25]. Adipocytes also have a specific G-protein-coupled receptor for lactate [26]. Glutamine is produced in one cell (glial), secreted and then taken-up by another type of cell (GABAergic neurons) for the production of GABA (gamma-amino butyric acid) [27]. Extracellular glutamine is particularly relevant for cancer cells with a glutamine-stimulated anabolic state [28,29]. Citrate is secreted by epithelial cells of the prostate [30], while other cells (sperm) have influx citrate transporters and these cells use citrate for fuel and for the production of long-chain fatty acids, cholesterol and steroids [31]. These are then examples of cooperativity, possibly orchestrated by a cell-fate plan involving asymmetric cell division with consequent interdependence of cells associated with division of labour.

While DOL will obviously reduce the number of genes expressed in a single cell, it has been estimated that even a dedicated cell such as a B-cell lymphocyte, will express more than 10 000 genes [32]. A dedicated cell with responsive receptors is still a complex entity even with DOL.

Of course, not all of the examples of division of labour involve a direct AsCD or mutual survival. Erythrocytes, terminally differentiated cells which lack a nucleus, mitochondria, lysosomes, endoplasmic reticulum and Golgi apparatus, are dedicated to serving others by supplying oxygen and removing tissue acidity via their haemoglobin content. These are “singlet” cells with no coupled cells needed to balance this formation of erythrocytes, but their own survival for about 110 days is dependent on a variety of other cells for nutrients. Although being dedicated and loaded with haemoglobin, one red blood cell still contains more than 750 proteins for its survival [33]. A very dedicated cell without responsive receptors is still somewhat complex.

As opposed to the division of labour by a fully deployed cell using AsCD, it is also relevant to acknowledge the possible function of opposing cell actions such as fusion, engulfment and endosymbiosis in both evolution [34,35] and in the growth of modern multicellular organisms.

The CTC model proposed in this hypothesis evolves from a consideration of division of labour with fewer genes expressed, involving asymmetric cell division with reciprocal interactions as indicated between fungi cells and for pheromone interactions.

#### A balance of cells in a multicellular organism is achieved by complementation of the types of cell division (CD) - Symmetric (SCD) versus Asymmetric (AsCD)

It is often assumed that eukaryotic cells, in culture, undergo simple symmetrical division to proliferate. This may be a complete assumption related to the observation that many biology textbooks focus on mitosis with symmetric division or it may be based on evidence from light microscopy where there is no obvious physical change in cellular characteristics. However, despite the latter, there may have been asymmetric cell division with many possible outcomes as discussed in the next section. Of course, there could be a combination of SCD and AsCD in these cultures just as there is for growth and development of a whole organism [36].

A classical line of thought has been that, after SCD, one cell could be transformed by the environment (heat, dehydration, presence of an interacting ICM) into a “new” cell and this cell could produce duplicated progeny of this new cell by SCD. Alternatively, one cell of a pair could be altered occultly (or be primed) by the environment such that a new cell type only becomes obvious after another SCD. As such, there was no asymmetric cell division.

However, currently, while the environment is still considered a critical part of the decision making, AsCD is known to be widespread in many aspects of *Life* – even ubiquitous. AsCD may be produced in several ways and survival of multicellular organisms is a balance of SD versus AsCD. AsCD, controlled by events that occur pre-cytokinesis, can be classified as either cell-intrinsic, involving an inherited determinant, or cell-extrinsic, involving intercellular communication or environmental factors. In addition, the determination of asymmetry could be post-cytokinesis and extrinsic, following an initial symmetric distribution of cellular constituents. (See Additional file 1,3* Note 1 “The Roles of Symmetric* versus *Asymmetric Cell Division”* for evidence and causes of AsCD and factors affecting AsCD/SCD balance).

Stem cells are thought to balance self-renewal and differentiation through judicious variation of asymmetric and symmetric divisions. The balance is not hardwired into the genes but is responsive to extrinsic and intrinsic cues [37]. Hemopoietic stem cells will form different progeny (erythrocyte, monocyte, lymphocyte etc.) based on the exposure to a variety of ICMs such as interleukins, colony-stimulating factors (CSFs) and erythropoeitin [38]. The balance of AsCD versus SCD can depend on these types of extracellular factors and also on cellular molecules such as p53 protein, cAMP or GMP, or on the lineage, the stage of growth or even light exposure (See Additional file 1,3* Note 1*).

Modelling has been used to determine the relative contributions of the various options of AsCD and SCD. Mathematical modelling and lineage studies to explain neuron formation within the mouse neocortex have focused on the relative amounts of three types of CD - AsCD, SCD with “terminal” differentiated cells produced and SCD with “non-terminal” proliferative cells produced [39]. The conclusion was that asymmetric and both types of symmetric cell divisions coexist during the entire period of neurogenesis. Other models of cell division, in hematopoietic stem cells, indicate a need to consider the time taken for, and the frequency of, cell division by the AsCD and SCD of stem and progenitor cells and a need for control by external signals [40]. In the *Drosophila* optic lobe, four signalling pathways control the sequential transition of symmetrically-dividing neuroepithelial cells into asymmetrically-dividing neuroblasts as the proneural wave progresses across the neuroepithelium [41].

While AsCD is often considered to be a fundamental characteristic of stem cells, in an active intestinal crypt setting, it is SCD, not AsCD, which balances the stem- and progenitor-cell losses by proliferation of nearby cells [42].

Overall, the balance of cells in a multicellular organism is achieved by both SCD and AsCD as directed by the cell-fate plan of the organism with control by external signals. The CTC model proposed in this hypothesis explains how external signals control cellular balance by appropriate selection of SCD or of AsCD as required.

#### Cells within a multicellular organism may be able to select from a portfolio of mechanisms to divide in order to produce a required outcome

Within the types of symmetric and asymmetric cell divisions, there are many theoretical possibilities. These are depicted and discussed in Additional file 1,3* Note 2 “Categories of Symmetric and Asymmetrical Cell Divisions”* Figure 1. This Figure lists at least ten options of cells in forming a lineage, including self-renewal and differentiation which may be terminal. Some other examples of the AsCD categories are discussed in Additional file 1,3* Note 3 “Further Examples of these AsCD Categories”*.

While this portfolio of options is impressive, to encompass totally the options of a cell, it would be necessary to include the potential of cells to reverse or change from a differentiated state via a cell division. Some of these extra options are included in Additional file 1,3* Note 4 “Change of differentiation type – options of a differentiated cell”.*

The CTC model proposed in this hypothesis explains how external signals help select the appropriate type of cell division required from these options.

### How does a multicellular messenger - a growth factor - affect cellular decisions?

An example of a extracellular molecule which communicates information via a plasma membrane receptor is the Insulin-Like Growth Factor-I (IGF-I) - a 70-amino acid ICM which participates in communication between cells in endocrine, paracrine and autocrine modes [43] and whose internalization may lead to changes in hyperplasic growth or to proliferation etc. The “IGF system” is complex, with several essential components including ligands; IGF-I and IGF-II, cell membrane-bound receptors (e.g. IGF-IR), at least six soluble “binding proteins” (e.g. IGFBP-1…-6) which form binary complexes with IGFs in the extracellular environment, plus even a ternary complex in serum, form the system. Also, IGFBP proteases will play a part [44].

In our current understanding of this system, the transfer of information from one cell to another by IGF involves receptor binding, subsequent kinase activation, followed by increased metabolism and a decision to divide, perhaps to differentiate or not to apoptose. This IGF receptor-binding is reduced by the limited availability of the free IGF due to its binding by the IGF binding proteins (IGFBPs). The IGFBPs have other suggested functions e.g. reducing the loss of IGF via the kidney due to the larger size of the complex, decreasing proteolytic cleavage and increasing storage. Modified IGFs that don’t bind IGFBPs {e.g. des(1–3)IGF-I}, have a higher activity than IGF itself and this is explained by the increased availability of the free modified IGF [45]. While these explanations seem valid, other explanations are required to explain fully the IGF system, especially the IGF-independent effects of IGFBPs [46]. The CTC model proposed in this hypothesis offers an alternate explanation.

### This understanding is limited. What are the unanswered questions?

How does the reception of an IGF-I signal define how the cell will respond to the signal? The cell may change its rate or focus of metabolism and/or the signal may be interpreted to produce a life change to the cell. The former *metabolic* effect (involving phosphorylation of IGF-IR and activation of the mitogen-activated-protein-kinase path and/or of the phosphatidylinositol-3-kinase path) may be a prerequisite for the *cellular* effect (involving nuclear interactions, transcription effects, chromosomal reorganization and other whole-of-cell decisions). For the latter, how does the cell know which switches are to be activated or deactivated; will the cell divide, differentiate, transform or undergo apoptosis or otherwise change its status? What intelligence is required to make these decisions; what is the messenger molecule and how does the message reach and be interpreted by the nucleus?

How is IGF-I mostly the well-known stimulator of growth [47] but sometimes an inhibitor of growth (in cultured smooth muscle cells [48], lymphocytes [49] and Wilm’s tumour cells [50])? How also is a particular IGF-BP an inhibitor of growth (of a breast cancer cell line [51] and a fibroblast cell line [52]) and, at other times, a stimulator of growth (in cultured fibroblasts [53] and osteoblast-like bone cells [54])? How can it be explained that an IGFBP may have an ability to stimulate cells in an IGF-independent fashion [53,54] if the current model has the IGFBP solely as a binder of IGF thereby restricting IGF loss and IGF activity? Overall, how does the whole organism know if there is a balance of the cells producing IGFs and those producing IGFBPs? Some of the answers may be associated with the discovery of receptors for certain IGFBPs and the presence of such receptors becomes part of the current CTC model, described in Section (B), used to answer these questions.

Similar questions apply to other growth factors and related molecules (ICMs) which have both stimulatory and/or inhibitory effects depending on cell-type used, the concentration of ICM, the presence of other ICMs, the time of exposure and the developmental state of the cells. {See Additional file 1,4 *“The Effects of Growth Factors and other Bioactive Molecules (collectively referred to here as ICMs - Information-Carrying Molecules and Inter-Cellular Messengers)”* for the effects of some fifty ICMs on cell proliferation, both stimulatory and inhibitory).

How do these ICMs have opposite effects on different cells and/or in different circumstances? Of particular note is that a number of binding proteins (BPs) (besides the IGFBPs) have both stimulatory and inhibitory effects e.g. FGF Binding Protein, Sex-Hormone Binding Globulin, Kallistatin (Kallikrein Binding Protein), Corticosteroid Binding Globulin. Again, how can these BPs that bind ICMs be stimulants of cell growth if they reduce the amount of the biologically active Free ICM? Again, the current model relies on receptors for these BPs to explain their conflicting effects.

## (B) Results and discussion:- a new model – a simplistic basic model

### 1. Description of the essentials of the basic model – a balance of Trefones and couplet cells

#### (a) Primary couplet cells and Trefone couplets – the CTC Model with the IGF system as a prime example

A signalling molecule which is secreted from one specific cell to communicate information relating to cellular activities, via a receptor in another “couplet” cell, and which binds another “couplet” signalling molecule produced by this other “couplet” cell, is defined herein as a **“Trefone” (T)**. It is a specific ICM which transmits a chemical signal (1) to affect certain metabolic reactions and (2) to produce a life change to the cell that receives its message. In this model, an IGF-I could be a Trefone for a cell which produces it and which also has a receptor for its couplet molecule – a specific IGFBP. Equally an IGF-BP could be a Trefone for a cell which produces it and has a receptor for IGF-I. Each Trefone-receptor complex would be internalised into the responding cell, to be part of nuclear messages to affect metabolic and cellular-life decisions. The IGF-I and the IGFBP would be classed as “Couplet Trefones” as they form their own complex (IGF-I:IGFBP) – a complex herein referred to as a Trefone Couplet Complex (TCC). (See Additional file 1,5* Note 1, “Additional Information on the Definition of a Trefone*” for more on the properties of a Trefone and Additional file 1,5* Note 2 “Clarification on Ligand and Binding Protein Interaction; the TCC*” for more discussion on the complex of ligand and binding protein).

One Trefone is produced and secreted by each of two separate cells and these initial two cells are coupled by their prior formation via asymmetric cell division of their parent cell. These two “Couplet Cells” are also coupled in another way in that each cell type has a receptor for the Trefone produced by the other and each cell is altered in response to the signal tranduced from the Trefone of the other cell. The Trefone signal from one cell will transfer a message for the other cell (i) normally to increase metabolism and to stimulate production of the other Trefone and (ii) if necessary, to stimulate cell division or induce some other “whole-of-cell, life/death” action to balance the activity of total “a” cells and “i” cells. Lower levels of the Trefone would induce a metabolic effect while higher levels, prolonged [55], would induce cell division or other action. For example, insulin from a beta-islet cell, when endocytosed at a low level by an insulin receptor into a specially receptive cell (a couplet cell, perhaps an alpha-pancreatic cell), would stimulate metabolism and the secretion of the couplet Trefone (perhaps glucagon), while at a high level, upon prolonged exposure, insulin would stimulate the proliferation of the receptive alpha-cell, to produce even more Trefone (glucagon) secretion. (Note that these effects are separate from the receptor-mediated effect of insulin on carbohydrate and on other metabolic activities of cells generally). For IGF-I signalling, the metabolic and the whole-of-cell effects use the same pathway; in human intestinal smooth muscle cells, the *metabolic* effect of IGF-I (e.g. to regulate IGFBP production) is mediated by activation of distinct MAP kinase and PI 3-kinase pathways, the same pathways through which IGF-I stimulates *growth*[56].

**Figure 3:**
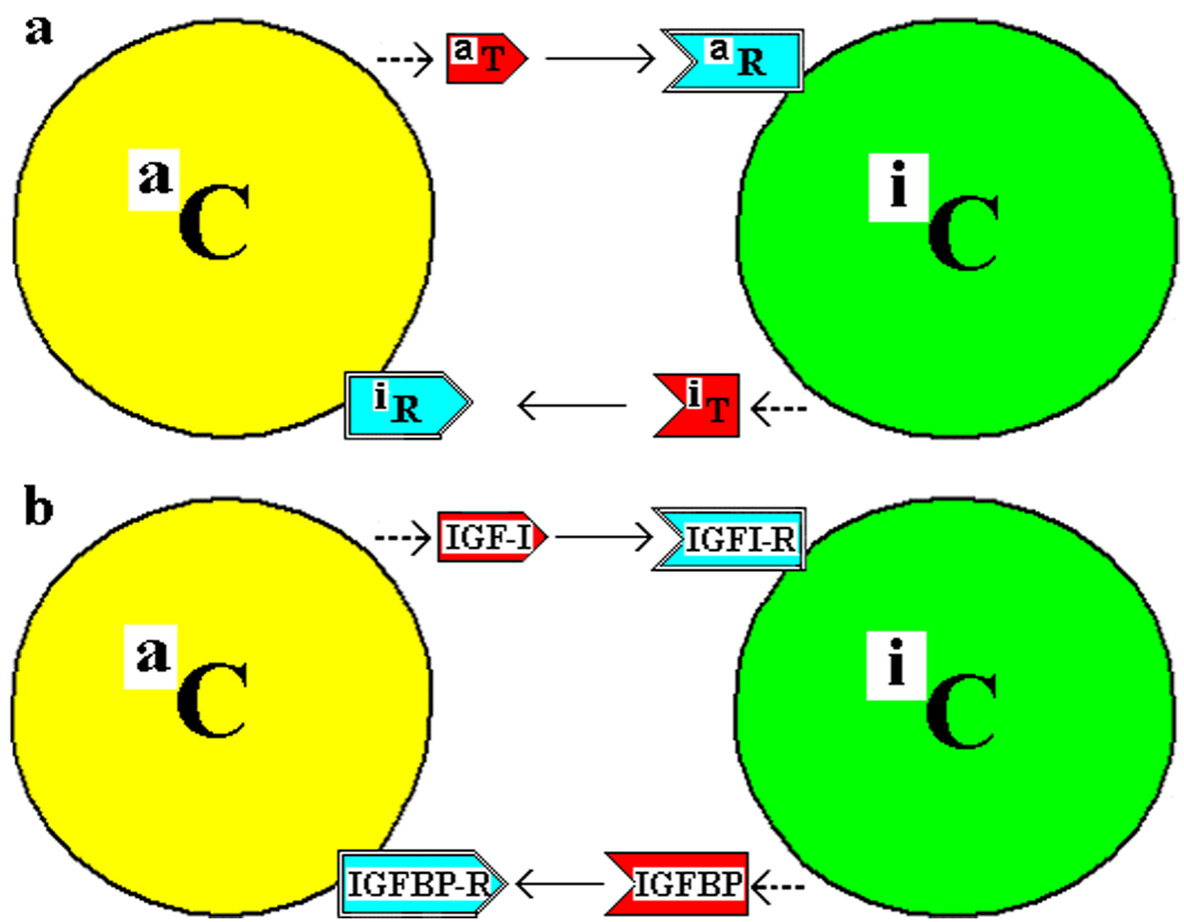
**Couplet Trefones and cells and their membrane receptors. (a)***A couplet of cells (*^*a*^*C and*^*i*^*C) with each producing a soluble Trefone and each having a receptor (R) to bind the soluble Trefone produced by the other cell.*^*a*^*R on*^*i*^*C binds*^*a*^*T and*^*i*^*R on*^*a*^*C binds*^*i*^*T.***(b)***A couplet of cells with the a-Cell producing the “a” Trefone, IGF-I, the i-Cell producing the “i” Trefone, one specific IGFBP, and each having a receptor to bind the Trefone produced by the other cell. There is a receptor for IGF-I on the i-Cell and a receptor for the IGFBPn on the a-Cell.*

The “Couplet Cells” would have been produced by asymmetric cell division of an o-Cell (the original cell - a stem or progenitor cell) to produce two cells committed to or already differentiated. This is referred to as Dual LCDf in Additional file 1,3* Note 2 “Categories of Symmetric and Asymmetrical Cell Divisions”* Figure 1, B(iv), where non-identical C/D_1_ and C/D_2_ cells are produced by AsCD. (Subsequent cell divisions may be symmetrical (SCD, SRE) to produce more of each of these two types of cells). The coupled progeny cells will be referred to herein as the “a” Cell type (a-Cell or ^a^C) and the “i” Cell type (i-Cell or ^i^C), rather than C/D_1_ and C/D_2_. (See Additional file 1,5* Note 3* “*Cell Couplets – AsCD and the a-Cell and i-Cell”* for why the letters “a” and “i” are selected and for the format of the diagram of the AsCD that produces these couplet cells). The a-Cell synthesizes and secretes the “a” Trefone (a-Trefone or ^a^T) and the i-Cell synthesizes and secretes the “i” Trefone (i-Trefone or ^i^T). The a-Cell has a receptor (^i^R) for the i-Trefone and the i-Cell has a receptor for the a-Trefone (^a^R). In this model, both cells have receptors for the Trefone Couplet Complex (TCC) of ^a^T:^i^T but, for simplicity, this is not shown in Figure 3(a). A specific example is given in Figure 3(b) for the IGF-I and IGFBPn couplet. This system of Coupled Trefones and Cells is referred to as the “CTC” model.

The purpose of Trefones is to maintain a balance of cells. This can be understood by a simple example:- If the number of cells of one type (a-Cells) is in excess (or if they have a high metabolic rate) of that of cells of the couplet (i-Cells), then the concentration of (Free) a-Trefone will be in excess of that of i-Trefone, with a limited concentration of TCC. Free ^a^T then stimulates the i-Cells initially to increase the biosynthetic rate of ^i^T production, and then stimulates the i-Cells to divide symmetrically (SRE) so as to produce more ^i^T to match the concentration of ^a^T. This stimulation of the i-Cells is also dependent on a signal that relates to the concentration of TCC and continues until the latter is high and the Free ^n^T concentrations, [^a^T]_F_ and [^i^T]_F,_ are low and equal. In the same period, the a-cells would be quiescent or perhaps undergo cell divisions, of the types indicated in Additional file 1,5 *Note 3,* Table S1, to produce more i-Cells. For the IGF system, IGF-I from the a-Cell will stimulate the i-Cell and an IGFBP from the i-Cell would stimulate the a-Cell but each response would be modified by the level of the IGF-I:IGFBP complex. Each cell would thus measure these external Trefones to assess the activity/number of couplet cells and would respond to maintain a balance.

The situation is similar if the number of a-Cells and i-Cells are equal and in harmony in a mix and then ^a^T is added in excess. The i-Cells will undergo cell division to replenish rapidly these cells to balance the perception of a-Cell excess. In the same period, this would produce reducing-to-zero stimulation (i.e. inhibition) of a-Cell metabolism plus stimulation of some a-Cells to produce two i-Cells and perhaps some a-Cells would divide to produce more i-Cells while maintaining a-Cells. This latter scenario would also occur if only cells of one type (e.g. a-Cells) are present initially and the corresponding Trefone that they produce is added. Again, these types of cell divisions for the a-Cell are indicated in Additional file 1,5 *Note 3*, Table S1.

Thus, Trefones would control an individual cell’s metabolic activity and also cell numbers and types by regulation of SCD and AsCD. a-Cells need to balance i-Cells because the initial AsCD (dual LCDf) of the o-Cell was designed to allow a “division of labour” (DOL) with consequent increased efficiency of production and use of metabolites. The function of the this reciprocal relationship of Trefones with receptors is to balance mutually the activity and number of Couplet Cells produced by this AsCD and by subsequent cell divisions (symmetric or asymmetric) in a particular cell lineage. The presumption here is that the total body of cells of an organism is in harmony and balance and that this living equilibrium is maintained by metabolic induction/inhibition within cells and/or by an increase/decrease in cell number and/or by variation in the types of cell produced by cell division and associated differentiation.

A potential CTC system exists in *Drosophila* and this is examined in Additional file 1,5* Note 4 “Drosophila – Potential Trefones and Asymmetric Cell Division”.*

In the CTC model, the two Trefones that are newly expressed by the two progeny following a specific cell division are primary Trefones. Secondary Trefones will be discussed later.

#### (b) The reason for a cell’s need to measure the Trefone couplet complex (TCC)

How does a cell know if one Trefone is in excess of the other? If the cells have been produced from the progenitor cell for “division of labour” then each cell couplet needs to know (by “counting”) the state of the other couplet and to be able enhance or reduce its own metabolism or cell number or status in response to signals. The two cells of the couplet need to be in harmony or more exactly the two groups of a-Cells and i-Cells need to be in harmony. Their end product(s) within the inferred DOL agreement need to balance and each individual cell of the two groups of cells plays a part by responding to the Trefone from the other group. How does a cell decide, based on one measured Trefone level, whether there is a balance of the Trefone produced by one group versus the Trefone produced by the other group cell?

Put again, each cell will need more information than a single Trefone concentration gleaned by Trefone-receptor internalization, in order to assess the balance of Trefones (and thereby the balance of the cells) and thus subsequently to make decisions. For each couplet cell, does it decide to change just its metabolic rate or must it alter its cellular status – does it proliferate by SCD or AsCD, does it differentiate or apoptose or otherwise live or die? The CTC model suggests that each cell couplet will compute the balance or relative amounts of the two respective extracellular Trefones by assessing the concentration not only of one Trefone but also that of the TCC. The cell needs these two discrete signals and will perform a relative “count” of its own Trefone versus the couplet Trefone by measuring the amount of complex. For example, whether ^a^T stimulates a cell would depend on the cell type and the relative amounts of Free ^a^T (or Free ^i^T) and ^a^T:^i^T (TCC). One option of this CTC model is that this concentration of extracellular TCC would be directly measured by a separate receptor for it, which, along with one Trefone, is internalized into that cell. The two could be then transported within the ER to a common compartment wherein a new equilibrium of ^a^T, ^i^T and the TCC complex is established. These new equilibrium levels of Free Trefone and Bound Trefone (i.e.TCC) will allow each cell to “know” the balance of the external two Trefones. The cell can then respond to any imbalance by the interaction of Free Trefone of TCC with specific signal transducers or directly with transcription factors within the nucleus to change gene expression.

**Figure 4:**
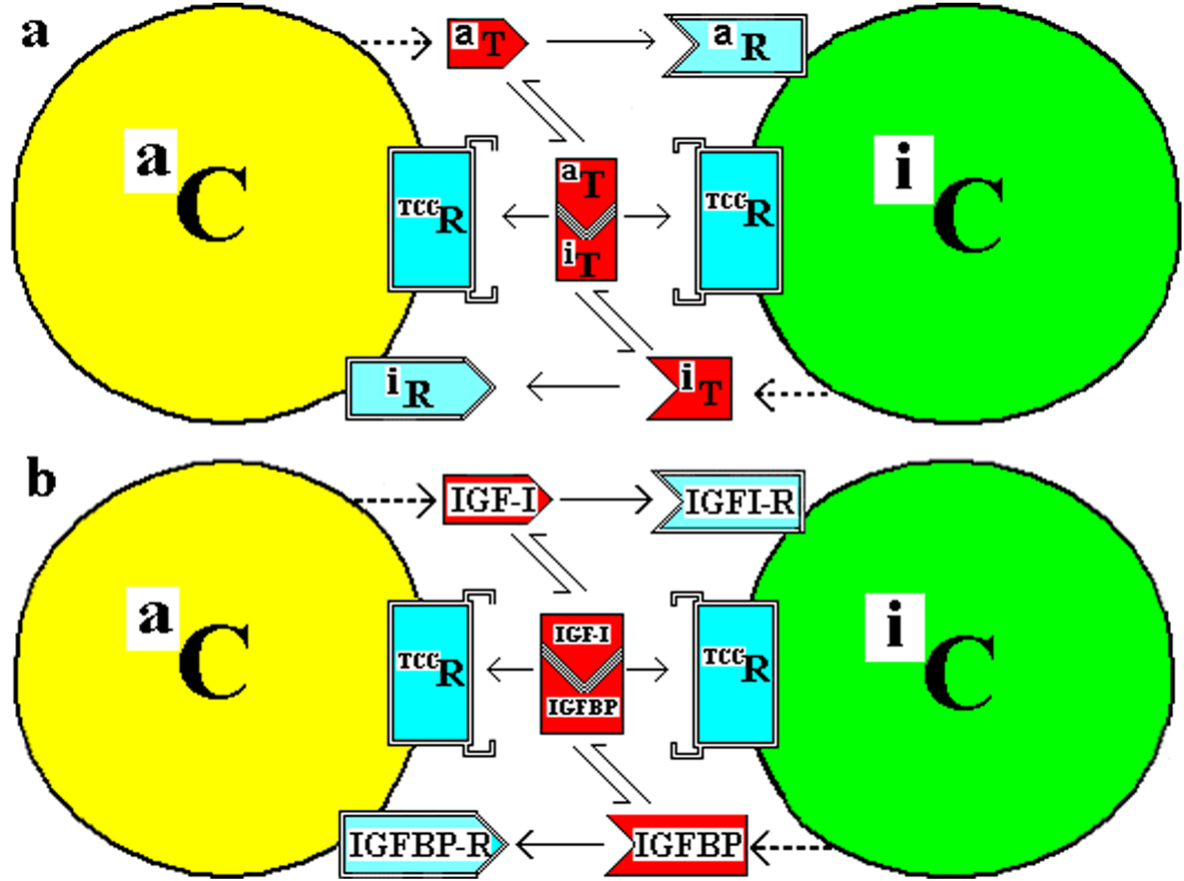
***Couplet Cells with membrane TCC receptors*. (a)***A couplet of cells (*^*a*^*C and*^*i*^*C) with each producing a Trefone and each having a receptor (R) to bind the Trefone produced by the other cell. This is as in Figure 3 but with a TCC receptor (*^*TCC*^*R) on each cell.***(b)***A couplet of cells with the a-Cell producing the “a” Trefone, IGF-I, the i-Cell producing the “i” Trefone, an IGFBP, and each having a receptor to bind the Trefone produced by the other cell. This is as in Figure*3*but with a receptor for an IGF-I:IGFBP complex on each cell.*

This latter is analogous to a model in *Drosophila* for the Patched receptor which binds the morphogen-ligand Hedgehog; it is the ratio of the internalized Free Patched (i.e. with no bound ligand) to the internalized Bound Patched (i.e. a Patched-Hedgehog complex) which determines cell response, not just the absolute number of Free Patched receptor molecules internalized [57].A receptor for the TCC (the complex of the Trefones) on each cell of a couplet, is one option of this CTC model. Alternatives to this option of a receptor for the TCC will be described later. Figure 4 illustrates this generically and for the IGF system.

How does the cell then interpret the combined signals from the ^n^T and the TCC? The signals could be interpreted to our understanding, based on an association constant (Ka) where Ka equals [^a^T:^i^T]/[^a^T].[^i^T] for the reversible formation of the TCC (^a^T:^i^T) from ^a^T and ^i^T i.e. ^a^T + ^i^T ⇆ ^a^T:^i^T. If the total concentrations of ^a^T and ^i^T were initially the same, and if the Ka value was assumed to be 1 × 10^10^, then this allows calculation of the Free concentrations of the Trefones at a range of given Total Trefone concentrations. From this input, the importance of knowing the concentration of Free levels of Trefones and of the TCC, plus the value of just having ^a^T bind to ^i^T, can be appreciated. (See Additional file 1,6 Table S1*, “Calculated Levels of Trefones and TCC, Connected to Cellular Actions”* for possible cellular reactions to varying concentrations of Trefones). An association can be made between the variation in concentrations of Free Trefones and of TCC with cellular responses, where the predicted response focuses on whole-of-cell decisions rather than metabolic changes. This Table is a critical part of the CTC model.

Depending on the concentration of Free ^i^T or ^a^T and of TCC, the ^a^Cells and ^i^Cells respectively, will respond. From the messages received, the consequent cell action may be symmetric cell division (SCD), asymmetric cell division (AsCD), transdifferentiation (TD)/SCD, apoptosis (APO), quiescence (QSC), differentiation or dedifferentiation to a progenitor to restart the lineage. The two examples described in Additional file 1,6 of quantitative and objective interpretations from the data in this Table show how the cells are able to make intelligent decisions based on their Trefone environment. In one case, the concentration of TCC is critical for decision-making where the concentration of Free Trefone is identical in three different situations which require different cellular reactions; in the other case, the presence of the complex amplifies the change in concentration of the Free Trefone. A simple example would be that if Free ^a^T were deficient, possibly indicating a deficiency of a-Cells, then existing a-Cells would proliferate by SCD (SRE) to double their number and some i-Cells would divide to form an a-Cell (plus an i-Cell) or even to produce two a-Cells by mechanisms explained in Additional file 1,5 *Note 3*, Table S1.

Each couplet cell will have internalized a certain amount of one Trefone and the TCC. As an example of an abnormal situation, let us assume that ^a^T is being normally produced by the a-Cell but the external ^a^T is not producing a response at ^a^R of the i-Cell because ^a^T is being somehow acutely destroyed or diverted elsewhere and the effective level of ^a^T is Lo (but not due to lack of response by a variant ^a^R or low ^a^R on the i-Cell). Residual extracellular ^a^T would form Lo ^a^T:^i^T complex and the i-Cell would therefore lack ^a^T stimulus so that decreased ^i^T production and i-Cell proliferation are expected. The extracellular ^i^T level would be initially in excess of ^a^T but slowly decreasing as it continues to stimulate the a-Cell. Intracellularly, the a-Cell, which produces ^a^T, has acquired a Hi level of ^i^T but a Lo level of TCC from receptor-mediated uptakes. Upon release of ^i^T and ^a^T:^i^T from receptors in a communal endosomal compartment, there would be a mixing and re-equilibration of the internalized ^i^T and ^a^T:^i^T so that the final level of the intracellular TCC and of free ^i^T or ^a^T would reflect the extracellular balance of ^i^T and ^a^T.

In the cell itself, this mathematical interpretation would be replaced by a concentration dependent activation and/or inhibition of specific genes to direct proliferation (or other) by its innate, inherited intelligence [58]; knowledge which will allow it to know whether there is balance between the two Trefones and thus between the two types of cells. The balance of extracellular Trefones, as determined by intracellular equilibrium levels, could then control cell decisions by altering the level of a transcription factor; for example, during mammalian embryo development, increased Cdx2 levels are associated with more SCDs, while downregulation of Cdx2 is associated with more AsCDs [59].

This CTC(EC) model with a receptor to measure the extracellular (EC) complex (TCC) is preferred to the alternate CTC(IC) model where only an intercellular (IC) TCC complex is important. This model will be discussed later but the intelligence from extracellular TCC(EC) is more direct and more appropriate than the involvement of only intracellular TCC. This is because the TCC(EC) is derived from sampling the production of its own specifically produced ^n^T which is secreted by its whole family of n-cells and not just by the individual cell.

The reasons that a cell-membrane receptor for extracellular TCC has not been detected include the lack of a convincing search and the fact that the number of receptor molecules may need only be very low to sample the large concentration of TCC. This will be addressed later.

#### (c) How does this model change our understanding?

The CTC model includes a controversial issue; cultured cells are not homogeneous but will contain a-Cells and i-Cells in variable amounts depending on the medium used to isolate them. The heterogeneity of cultured cells is well-known and will be heavily verified later. On the other hand, the difference between a-Cells and i-Cells may be minimal with no obvious physical transformation. The cells may be microscopically identical and may require microarray, mass cytometry or similar techniques [60] to detect genetic expression differences associated with the division of labour. The Couplet Cells may differ only in minor detail - the octet of cells in embryonic development was once believed to consist of identical cells.

An example of how the CTC model changes our understanding relates to the molecule derived from IGF-I by the loss of three N-terminal amino acid residues, the highly active des(1–3)IGF-I. The current reason accepted for the higher activity of compared to IGF-I, is that, because the desIGF-I does not bid IGFBPs very well, there is more *free* desIGF-I to act on the IGF-I receptor, thus producing a high receptor-mediated response.

In a CTC model, cultured cells would have been isolated in a medium containing a significant amount of IGF-I from the fetal calf serum or equivalent. If the model in Figure 4(b) is used here, the cells isolated would likely be predominantly i-Cells because a-Cells are not stimulated to grow by IGF-I. Some a-Cells may be present and they can be generated at any time by a change in the relative amounts of IGF-I and the corresponding IGFBPn Trefone which form the IGF-I:IGFBPn complex. The a-Cells potentially would be stimulated by the IGFBPn produced by the i-Cells but this BP would be completely bound by the IGF. DesIGF is then not a potent stimulator of a cell because of its high *free* concentration *per se,* rather, at the same time, the cell monitors/detects a low concentration of TCC because of the low association of desIGF and the IGFBP. Consequently, the cell interprets this as a low concentration of IGFBPn (normally produced by that cell), so there is a potent stimulation of growth of the i-Cells to divide by SCD to produce more IGFBPn. As the time of growth (in days) continues and the IGF-I decreases slowly and the IGFBPn increases slowly, there will be further changes in the cell composition as various SCD and AsCD options eventuate. It follows that with added IGF-I or added IGFBPn to a cell culture, the cell type which is in the minority – and there will always be some of both types present because of basal AsCD – may become the majority cell type. The minority type would be stimulated to SCD to increase its numbers, while the majority type would undergo AsCD to increase also the minority type.

{Another alternative explanation has been offered for the lower biological activity of IGF-I as compared with des(1–3)IGF-I as occurs in Ishikawa endometrial cancer cells with high levels of cell surface-associated IGFBP-3; it is the presence of cell surface-associated IGFBP-3 (not the secreted IGFBP) which reduces IGF-I-induced IGF-IR-signalling but not that of des(1–3)IGF-I [61]}.

Another dilemma, mentioned previously, is the number of growth factors and other ICMs which have both stimulatory and inhibitory effects on growth. At least 50 ICMs have this dual activity and these are listed in Additional file 1,4*.* While there may be many reasons to explain some of these dual actions, as outlined in the File, a complete explanation is lacking. The explanation from the perspective of the CTC model is that one Trefone will stimulate one cell type of the couplet cells and may inhibit the other cell type e.g. the a-Trefone will stimulate i-Cells but may inhibit a-Cells. The concentration of the Trefone complex, the TCC, will delineate effects.

### (2) Supporting evidence for the model

#### (a) Binding proteins as Trefone

In the CTC model, soluble binding proteins are part of the Trefone couplet – they are Trefones that carry a message of their own, separate from the message of the other interacting Trefone. An IGFBPn, once internalised, carries a message just as IGF-I does. Specific cells will have receptors for a specific IGFBP; perhaps for only IGFBP-1 or for only IGFBP-5. While the Trefone carries a message, the responding cell will specify the outcome of the consequent signalling, rather than the specific Trefone. Specificity is principally a product of the transcription factor repertoire of a given cell at the time of signalling. While it is the state of the cell rather than the nature of the signal itself that determines the outcome of signalling [62], more specifically, the concentration of Trefones, their relative ratio and the specific transcription factors will control the cell’s reaction to the message. Trefone activation of a receptor (e.g. the EGF receptor) controls the binding of two transcription factors in the *Drosophila* eye to affect EGF signalling [63].

#### (b) Evidence for cell-membrane receptors for binding proteins/Trefones

##### (i) Preamble

The presumption here is that a receptor for a particular substance would mean that the cell has a particular purpose for that substance – it may be a an essential metal ion, a favoured energy source, a substance that is required for metabolism that the cell is incapable of synthesizing because it hasn’t the genes (e.g. a vitamin), or it may be a messenger molecule. For the latter, if the molecule is not internalised, then the receptor would need to be very specific to translate and transfer the message; if it is to be internalised, the receptor would not need to be specific in that once the molecule is intracellular then it can deliver its specific message directly or by formation of some complex. In the latter case, the message becomes more specific and accurate in that it is now contingent on the presence of two Trefones.

##### (ii) Receptors for IGF binding proteins/Trefones

A typical description of an effect of IGFBP on a cell activity (e.g. to stimulate or inhibit growth) is that it is partly dependent on its binding to IGF-I, thereby preventing the IGF from binding to its receptor, and partly independent of this interaction, based on evidence using various IGF analogs that bind receptor or IGFBP or both. Cell-, surface- or membrane-associated IGFBP is often reported and this cellular binding is usually offered to explain the independent effects of a particular IGFBP. While some IGFBPs may be transiently bound to the cell-membrane as they are being secreted from a cell at low temperatures, there is now ample evidence to support the presence of a real receptor which binds an IGFBP. A receptor for each IGFBP has been reported with some 35 reports of evidence relating specifically to IGFBP receptors. See Additional file 1,7 *“A Listing of Receptors for the Six IGFBPs with Associated Cellular Effects, especially of Proliferation”* for this evidence. An IGFBP may then be a Trefone for specific cells.

This receptor binding may be specific for one or more of the IGFBPs or perhaps not so specific. Again, this lack of specificity is not critical as the specificity of action can be determined by the specific IGFBP once internalised.

The reception of an IGFBP message produces an intracellular activation of pathways and signalling independent of IGF-I. IGFBP-5 stimulates secretion of IGF-I and growth in human intestinal smooth muscle cells by activation of p38 MAP kinase-dependent and Erk1/2-dependent pathways, not reliant on IGF-I [64].

Note that an IGFBP can both produce an intracellular metabolic response and play a role in regulating cell proliferation. IGFBP-5 stimulates phosphorylation of the IGFBP-5 receptor [65] just as IGF-I stimulates the phosphorylation the Type 1 IGF receptor [66]. IGFBP-3 also stimulates the activity of an intracellular phosphotyrosine phosphatase activity that deactivates insulin receptor substrate-1. This down-regulates the IGF-I signalling pathway suggesting a major role for IGFBP-3 in regulating cell proliferation [67].

The CTC model would require that specific cells would react to only one Trefone that bound IGF-I. There is ample evidence of this IGFBP specificity: In a breast cancer cell line (MCF-7), IGFBP-3 stimulates a phosphotyrosine phosphatase activity that down-regulates the IGF-I signalling pathway, This is specific to IGFBP-3 since IGFBP-5 (structurally the closest to IGFBP-3), had no such effect and, of the cells tested, the IGFBP-3 effect occurs only in MCF-7 cells indicating that the stimulation is cell-type specific [68].

Other examples of IGFBP specificity are seen with normal calvaria bone cells and an osteosarcoma cell line. In calvaria cells, which produce IGF- I, only IGFBP-5 (of IGFBP-2 to -6) stimulates DNA synthesis while in Saos-2 cells, which produce little IGF I, only IGFBP-6 stimulates [69].

Overall, then, a specific IGFBP may be a Trefone for specific cells with an IGF the couplet Trefone.

##### (iii) Receptors for other binding proteins/Trefones, including “Soluble Receptors”

Firstly, there is evidence that every ICM that is potentially a Trefone, has a soluble binding protein, just as IGF-I has an IGFBP. There are many *binding proteins* for ICMs that are synthesised and secreted normally with no apparent relationship with receptor proteins. Other protein that bind ICMs are structurally related to receptors, being derived either from the extracellular domain of the membrane receptor by enzymic cleavage (shedding) or from alternative splicing of the precursor mRNA; these are referred to as soluble receptors. Additional file 1,8 *Note 1*, Table S1* “Extracellular Binding Proteins and Soluble Receptors”,* contains some 60 examples of these, excluding the six extracellular binding proteins for Insulin-like Growth Factor.

Secondly, some of these binding proteins/Trefones have themselves been shown to have cell receptors. Additional file 1,8 *Note 2*, Table S1 *“Cell Receptors for binding Proteins and/or Soluble Receptors”* lists 14 binding proteins for which a receptor has been detected, again excluding the IGFBPs. IGFBPs are not alone in being binding proteins that may be Trefones.

#### (c) Evidence for receptors for extracellular binary complexes - *ternary membrane complex formation*

##### (i) The IGF system – receptors for the binary complexes IGFI/II:IGFBPn –TCC receptors

The presence of a membrane receptor for the extracellular complex of two Trefones is one possible mechanism by which a cell could acquire the intelligence required for the assessment of the balance or imbalance of couplet cells. (An intracellular complex is another option and this will be discussed later). For the IGF system, a membrane receptor which internalizes the IGF:IGFBP complex would provide the necessary information.

Indirect evidence for this was reported in proliferating cultured opossum kidney cells, where, in separate experiments, labeled IGF-I and labeled IGFBP-3 were internalized and rapidly transported directly to the nucleus. When cells were treated with both labeled molecules simultaneously, both localized to the nucleus in a synchronous manner. The best interpretation was that there was an internalization of an IGF-I:IGFBP-3 complex and then nuclear translocation of the complex. IGFBP-3, which contains a nuclear localization signal (NLS), could act as a carrier for the complex, as IGF-I does not have a NLS. In the individual cases, there was probably endogenous unlabeled IGFBP-3 and IGF-I present (respectively) to allow formation of a complex. The fact that des(1–3)IGF-I, which binds the IGF-I receptor but not IGFBP-3, was not transported to the nucleus supports the interpretation [70].

Other evidence supporting the presence of a TCC receptor for the IGF system is presented in Additional file 1,9 *Note 1.* “*Further Evidence for the Existence of a Receptor for TCC”.*

Because of this indirect evidence for a receptor and because its existence could explain some other experiments, attempts have been made to identify positively this putative TCC receptor but without success [71,72]. Attempts may have failed because of technical reasons: if the IGF is first bound to IGFBP by the crosslinker DSS (disuccinimidyl suberate), then the amino groups through which the DSS crosslinks the TCC may not be available for subsequent cross-linking of the TCC with the receptor. See Additional file 1,9 *Note 2* “*Problems with Cross-linking Experiments”* for further discussion on this and other reasons for non-successful detection of receptors*.*

Overall, the reasons that a cell membrane receptor for extracellular TCC has not been detected include the lack of a convincing search and the fact that the number of receptor molecules need only be very low to sample the large concentration of TCC that is present extracellularly compared to the individual Trefones.

##### (ii) Other ICM systems – receptors for other binary complexes

Internalization of ternary complexes is not unusual and there are even some examples of quaternary and higher complexes involved in internalizations, including those involving co-receptors or receptor accessory protein (such as for IL-1 [73]) or the megalin complex [74]. It is acknowledged that some internalizations may be involved in simple removal of “waste” products and that evidence of a signalling response is needed following the internalization of the complex. However, even an assumed waste product, the heme-hemopexin complex with LRP/alpha 2-MR as its receptor, induced heme-oxygenase 1 mRNA transcription and protein synthesis in cultured monocyte [75]. Further evidence supporting the presence of receptors for a variety of complexes in other systems is presented in Additional file 1,10 “*Evidence for Binary Receptors aside from the IGF System”.* Some 30 examples are given. Internalization of the TCC is therefore very feasible and it is the favoured but not critical component of this current model because, as an alternative, the TCC could form intracellularly, in addition to its extracellular formation. [See Section 3 (a)].

##### (iii) Two receptors

The CTC model explains the variability of the effects of Trefones by the presence of two receptors - one cell-membrane receptor for one Trefone (either ^a^T or ^i^T) and one for the extracellular (EC) complex of the two couplet Trefones (for the TCC i.e. ^a^T:^i^T). The relative intracellular amounts of one Trefone and the TCC would direct cell stimulation or inhibition.

For the external-membrane receptor for the TCC, its binding site could be very similar in structure to the receptor that binds one or the other free Trefone. Just as an antibody prepared against ^a^T could be expected also to bind the ^a^T:^i^T complex (unless the epitope of ^a^T is obscured by the binding site of ^a^T to ^i^T), so there could be some receptor cross-reactivity between a free Trefone and the bound Trefone in the TCC complex. Receptor A may bind the ^a^T with high affinity, while Receptor B, which binds ^a^T:^i^T with high affinity, could bind ^a^T with low affinity. The evidence for two receptors, one with high affinity to bind one Trefone and one with high affinity to bind the TCC, is not directly available but there is ample evidence for the existence of two receptors (at least) for many ICMs. Often the receptors are shown to have a high-affinity and low-affinity binding sites for a specific Trefone. While the presence of these two (or more) receptors can be explained in other ways (Trefone or cell specificity; synergistic, permissive or modulatory effects, glycosylation differences), the view here is that the receptor with high affinity is the true receptor for the Trefone and the one with low affinity is in fact the TCC receptor. Further evidence supporting the concept of a receptor for a TCC is presented in Additional file 1,11 “*Evidence for the Presence of Two Receptors for a Particular Trefone, outside the IGF System”* with reference to some 30 ICMs.

While more defined knowledge of the receptors is known for some of the cases in Additional file 1,11 the emphasis is on the existence of one receptor which binds one Trefone with high affinity and some other receptor that binds it with low affinity, possibly indicating the binding by this latter receptor of the TCC (with high affinity). The existence of a TCC receptor is then not inconsistent with the known variability of the number of receptors or of receptor sites.

#### (d) Evidence for internalization, intracellular signal transfer and nuclear location

##### (i) Endocytosis of ligands, receptors and their nuclear localization (NL)

Firstly, a Trefone can affect cell **metabolism** by causing receptor phosphorylation with subsequent transmission of a message to activate or inhibit pathways of metabolism perhaps via a receptor kinase and downstream phosphatidylinositide 3-kinases (PI3Ks) and/or mitogen-activated protein kinases (MAPKs). Secondly, a Trefone can also affect **cellular proliferation** which is also regulated by these specific activated PI3Ks and/or MAPKs which translocates the message into the nucleus. The message is then translated into gene expression effects. The concentration of certain intracellular enzymes may then be altered by modifications to the level of their DNA transcription and/or RNA translation firstly to maintain or enhance a specific proteomic state for the cell and secondly, if necessary, to direct the choice of type of cell cycle dependent on the ratio of the signals, via DNA/chromosomal changes. Nuclear transfer of the message carried by each Trefone is critical for both functions.

In some cases, it has been shown that activation of receptor tyrosine kinase, internalization and nuclear localization of a Trefone (e.g. FGF-1) are required for stimulation of cellular proliferation [76,77]. However, while receptor autophosphorylation is part of the metabolic effect, it may be dissociated from a growth-factor mediated mitogenic response in the case of PDGF [78]. Again, this nuclear localization seems to be necessary for the mitogenic response of Schwannoma-derived growth factor (of the EGF family), but not for the early response involving the activation of “early” genes (NGFI-A and c-fos) [79].

Information in Additional file 1,12 *Note 1* “*Mechanisms of Nuclear Localization”* expands on the evidence for NL with emphasis on possible mechanisms.

##### (ii) Evidence for NL relating to the Trefones IGF-I/II

Proliferating opossum kidney cells internalize IGF-I and transport it directly to the nucleus, in contrast to resting cells where IGF-I is internalized by a clathrin-coated pit pathway and conveyed to endosomes [70].

IGF-I has been shown to translocate into the nucleus of epithelial cells from chicken embryonic lens but not into the nucleus of fiber cells that were derived from the differentiation of those epithelial cells [80]. There is also evidence that IGF-I induces nuclear translocation of IRS-1 (a down-stream effector of IGF-I) to the nucleoli within special fibroblast cells transfected with IGFI-R [81]. In addition, IGF-I does cause nuclear effects relating to cell division; in oligodendrocyte progenitor cells, IGF-I promotes nuclear localization of cyclin B/cdk1, a complex that regulates progression through G2 and entry into mitosis, as well as of Cdc25C, an activator of cdk1 which then enhances progression through G2/M to cell division [82].

##### (iii) Evidence for NL relating to the Trefone receptor, IGF-I receptor

A receptor that is found to be localized in the nucleus may have translocated there of its own accord or it may have been co-transferred with a Trefone/ligand. The experimental approach has often focused on the detection of only the receptor and not both the receptor and Trefone/ligand so that there is no way of knowing if the complex is present or not. On the other hand, a receptor molecule alone may carry a message transferred from a Trefone molecule and may simply transfer the signal into the nucleus in the absence of the Trefone itself.

Early evidence of a nuclear translocation was obtained with cultured keratinocytes; after the addition of IGF-I to these cells, most receptors appeared within the cytoplasm in a perinuclear location whereas, in the absence of IGF-I, receptors were located at the cell surface [83]. At the same time, IGF-I receptors were detected in the nuclei of hamster kidney cells [84].

Regulated by IGF levels, cell-surface IGF-IR translocated to the nucleus following clathrin-mediated endocytosis. Nuclear IGF-IR was present in renal and breast cancer cells and in nonmalignant tissues characterized by a high proliferation rate. In addition, the nuclear IGFR was phosphorylated in response to IGF-I, it was bound to chromatin and acted directly as a transcriptional enhancer [85].

IGF-I dependent nuclear translocation of receptor was also reported in melanoma cells but the receptor was first modified by small ubiquitin-like modifier protein-1 (SUMO-1) before its association with enhancer-like elements to increase transcription [86].

In contrast to the above, it has been shown that IGF-IR localizes to the nucleus of corneal epithelial cells and is associated with nuclear chromatin, but this appeared to be independent of IGF-I in that neither IGF-I withdrawal nor IGF-I stimulation altered nuclear IGF-IR [87]. Again, IGF-IR translocates to the nucleus in breast cancer cells and IGF-IR stimulates *IGF-IR* gene expression [88]. In orbital fibroblasts from patients with Graves’ disease, IGF-I and just the alpha subunit of the IGF-I receptor {derived from the full receptor (α, β) by a membrane protease} appear in the nucleus [89].

Overall, the evidence for receptors in the nucleus could be interpreted as being a feed-forward effect where, if there is more ligand/receptor internalization and inadequate recycling of receptor, then there will be more stimuli to produce more receptor *de novo*.

##### (iv) Evidence for NL relating to the Trefones IGFBP-1… IGFBP-6

There have been several reports of nuclear localization of IGFBPs, specifically relating to IGFBP-2, -3 and -5. Of course, nuclear localization is necessary but not sufficient to prove intranuclear activity affecting cell division; functional roles of nuclear localization needs to be established. See Additional file 1,12 *Note 2* “*NL of IGFBPs”.*

##### (v) Evidence for NL relating to the other Trefones and ICMs and their receptors

There are numerous reports of NL for a variety of ICMs with and without their receptors. The cellular effects are often mediated by chromatin interactions but it is not possible to deduce that it is just the receptor or just the ligand that influences these effects. See Additional file 1,12 *Note 3“NL of Potential Trefones other than those of the IGF System”* for evidence relating to these issues.

##### (vi) Conclusion

There is ample evidence for nuclear localization of Trefones (e.g. IGFs, IGFBPs and other potential Trefones) and their receptors. The presence of receptors in the nucleus would be explained here as a means of regulating the synthesis of receptors for the cell surface and thus control metabolic reactions; the presence of either free ^a^T or free ^i^T or of the ^a^T ^i^T complex (TCC) in the nucleus would regulate the transcription of genes for cell division.

#### (e) Evidence for couplet cells

##### (i) A Mechanism for the formation of couplet cells – asymmetric cell division (AsCD)

AsCD is widespread in nature (as documented in (A) Section 2) and with dual LCDf (Lineage Commitment to Differentiation), couplet cells could readily be produced. Cell types are usually identified by the use of membrane proteins and/or use Cluster of Differentiation (CD) markers so there is an expectation that progeny cells from AsCD will differ in their membrane proteins, and it is plausible that their types of membrane receptor molecules will differ.

Precarious equilibrium of life is sustained by individual cellular adaptation plus variation in cell numbers by SCD and AsCD both of which also balance cell deaths. The key to life may be in the balance and harmony of cell types with each type performing specific functions to make the multicellular organism a multifunctional entity. This CTC model suggests the formation of two coupled cells (an “a-Cell” and an “i-Cell”) from the parent “o-Cell” by AsCD. For the IGF system (See Figures 3 and 4), this could involve IGF-I (an ^a^T, produced and secreted by a-Cells) and one IGFBP (IGFBP-n, an ^i^T, produced and secreted by i-Cells). i-Cells would have a receptor for IGF-I, while the a-Cell would have a receptor for the IGFBP-n. Receptors for the TCC (the IGF-I:IGFBP-n binary complex) would be on both cells but because of the predominance of the complex, compared to the concentration of free IGF-I and free IGFBP, very few TCC receptors would be needed to monitor the extracellular concentration of TCC.

##### (ii) Examples of potential “a” Cells and “i”cells as evidence for this CTC model

Cells controlled by the IGF system

The literature data on the effects of components of the IGF system are enormous and confounding as documented partially above. Our understanding is limited to selective pockets of information and there is no all-embracing umbrella theory to explain the myriad and maze of confusing results and some examples follow. IGFs are often stimulators of proliferation but sometimes inhibitors [90]. With a cell line from a Wilms's tumour, the addition of IGF-I or -II inhibits growth over four days and this is apparently specific as the addition of IGFBP-2 removes this growth inhibition [50]. IGFBPs are also inhibitors of growth by virtue of their ability to complex IGFs and prevent their stimulatory properties, but, independent of this binding, IGFBPs may inhibit or stimulate, perhaps by their own receptors as described in Additional file 1,7 Table S1. IGFBP-5 is an inhibitor of proliferation of mink lung epithelial cells [91] yet it is a stimulator of osteoblast-like bone cells [54]. IGFBP-2 exhibits both properties also [92], with opposing effects on different cell types - sometimes pro-proliferative, sometimes an inhibitor of proliferation [93], with conflicting roles in suppressing the growth of normal prostate epithelial cells, while enhancing the growth of prostate cancer cells [94].

It is proposed here that the current explanations used to understand the complicated IGF system are inadequate partly because our understanding of cancer cell growth is inadequate. Experimental results are derived mostly from *in vitro* studies where firstly, a variety of cell lines have been used, often derived from cancer or immortalized cells where extrapolation is made to normal cell behaviour, and secondly, the activity of cells is monitored in synthetic and ill-defined media and sometimes at extreme concentrations. A further potential inadequacy is added here; that the cultured cells are not homogeneous perhaps initially and probably not after 1–6 days of growth. The assertion here is that the cells are initially, predominantly of one type, the a-Cell type or the i-Cell type. With growth in the 1–6 days of experimental observations, a mixture of asymmetric and symmetric cell divisions may create a heterogeneous mixture of cells. Dependent on the presence or absence of the Trefones either provided in the medium or produced by the predominant cell type, a predominantly a-Cell population may be converted to a predominantly i-Cell population or *vice versa* or conversion may occur to produce a final cell population of 50:50 or anywhere in-between. The following Section (iii) details evidence of heterogeneity of cells cultures.

**Figure 5:**
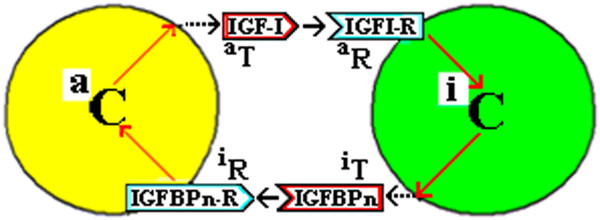
***Couplet cells with Trefones IGF-I and IGFBP-n and their membrane receptors.****One Trefone added to one cell type stimulates the production of the couplet Trefone. IGF-I from the a-Cell will stimulate the production of an IGFBP by the i-Cell, and conversely. This is similar to FIGURE*3*(b). (In another couplet of cells, IGF-II would bind its own specific IGFBP-n)*.

The CTC model is again described in Figure 5. Some preliminary examples and expectations of this “a-Cell and i-Cell” model follow, with respect to Trefones IGF-I/II and IGFBP-n, before a detailed description of evidence is provided. In the following, both a-Cells and i-Cells are present but one cell type predominates. With cell division by appropriate SCD and/or AsCD, cells of one predominant type can decrease with increase of the other or both can growth. If the a-Cell cannot produce the i-Cell by AsCD or SCD *(or vice versa)* as possibly in a cancer cell line, then anomalies will be observed.

***a-Cell Predomination*** – Proliferating of a-Cells occurs with IGFBP-n as a stimulant and growth should be inhibited by the presence or addition of IGF which would reduce the Free IGFBP-n by formation of the complex IGF:IGFBP-n*.*

*The IGFBP-n is then a stimulant if the IGF:IGFBP-n concentration is limited.* For example*,* IGFBP-3 induces cell growth in a dose- and time-dependent manner *in vitro,* in three metastatic/highly aggressive colon carcinoma cell lines [95].

*The IGFI/II inhibition should be blocked by IGFBP-n.* This is observed when growth of a colon carcinoma cell line is inhibited by IGF-I/II and the inhibition is blocked by concurrent addition of IGFBP-3 [96]. High levels of the TCC would inhibit growth as high TCC signals will pass a message that high growth of both cell types has occurred.

***i-Cell Predomination****-* Proliferating occurs with IGFI/II as the stimulant and growth should be inhibited by the presence or addition of IGFBP-n which would reduce the Free IGF by formation of the complex IGF:IGFBP-n.

*IGFI/II is then a stimulant if the IGF:IGFBP-n concentration is limited.* This is the normal “growth factor” effect of IGFI/II for which it was named

*The IGFBP-n inhibition should be blocked by IGFI/II.* Prior inhibition of IGF-responsive colon tumor cell line by IGFBP-2 was reversed by the addition of IGF, while the lower growth activity, measured in transfected embryonic kidney clones secreting IGFBP-2, was compensated in great part by the administration of IGF-I or –II [97]. The addition of IGFBP-2 produces less proliferation by binding IGF-I. This is the interpretation of results of studies with an intestinal epithelial cell line derived from rat jejunal crypts. Two IGF-I analogs which have a reduced affinity for IGFBPs, exhibited 10-fold greater potency than IGF-I, presumably because, with growth, endogenously secreted IGFBP-2 depresses the Free IGF-I available to bind cell receptors and cause cell division [98].

For the IGF system, the a-Cell Type and the i-Cell Type could be identified by the following list of properties.

(1) For the **a-Cell Type:**

(i) The cells will have receptors for the IGFBPn but will not produce the IGFBPn (^i^T).

(ii) The cells will produce and secrete IGF-I/II (^a^T).

(a) And have receptors for the specific IGFBP-n.

(b) But have no receptors for IGF-I/II.

(c) But will not produce nor secrete IGFBP-n.

(iii) IGFBP-n will stimulate IGF-I/II protein/mRNA production by a-Cells; IGFBP-n receptor is presumed present.

(iv) IGFBP-n will normally stimulate proliferation of a-Cells; IGFBP-n receptor is presumed present.

(v) IGFBP-n will inhibit proliferation of a-Cells if in excess of a high level of IGF-I/II.

(vi) The cells will have receptors for the specific IGFBP-n (^i^T) but not for IGF-I/II; IGF-I/II will inhibit proliferation of a-Cells by binding IGFBPn.

(vii) The cells will have no IGF-I receptor and not secrete IGFBPn.

(2) For the **i-Cell Type;**

(i) The cells will have receptors for IGF-I/II but will not produce the IGF-I/II (^a^T).

(ii) The cells will produce and secrete the specific IGFBP-n (^i^T).

(A Specific IGFBP may be assumed to be the specific Trefone for the cell).

(a) And have receptors for IGF-I/II.

(b) But have no receptors for the specific IGFBP-n.

(c) But will not produce and secrete IGF-I/II.

(iii) IGF-I/II will stimulate IGFBP-n protein/mRNA production by i-Cells; IGF-I/II receptor is presumed present.

(iv) IGF-I/II will stimulate proliferation of i-Cells; IGF-I/II receptor is presumed present.

(v) IGF-I/II will inhibit proliferation of i-Cells if in excess of a high level of IGFBPn.

(vi) The cells will have receptors for IGF-I/II but not for the specific IGFBP-n; IGFBP-n will inhibit proliferation of i-Cells by binding IGF-I/II.

(vii) The cells will have no IGFBPn receptor and will not secrete IGF-I.

Extensive evidence is offered in Additional file 1,13 *“Detailed Description of Evidence Supporting the Existence of a-Cells and i-Cells”* to support the concept of a- and i-Cells, with reference to the above list of expected properties. The selective evidence is consistent with the proposed model and is chosen from a variety of cell types from a wide range of published information. The evidence has been pieced together by selectively picking snapshots of cellular activities to form a whole picture - a montage, from a mountain of often disparate and incongruous activities of a variety of often cancer-derived cells which have been stimulated to function perhaps in unnatural circumstances. The selection of data has been biased in order to produce the CTC model of what normal cells may do in normal organisms.

With the information from Additional file 1,13 it is possible to allocate cells into an a-Cell type or an i-Cell type. This is presented in Additional file 1,14 *“Summary of Candidates with mainly a-Cell-Type or i-Cell-Type Characteristics”* with additional Notes 1–11. It is noted that some of the differences in results could be due to a change in the cell types within the course of the experiment due to variation in cell-division types. The extent of change would depend on the content of the media and the experimental additives.

While an a-Cell and the couplet i-Cell may exist in close proximity in a tissue, they may also be separated within the body. One cell could be localized in the thyroid, pituitary gland or endometrium and the other in the liver. Certainly hepatocytes and other liver cells are very heterogeneous [99-101] and a specific individual Trefone could be produced by a specific cell type within the liver and other organs/tissues; there may be specific cells for the various binding proteins produced by the liver e.g. corticosteroid-, thyroxine- and sex hormone- binding globulins (CBG, SHBG, TBG). This separated locality could occur if the couplet cells were formed early in embryogenesis leading to separation within the adult body. Mobile cells in the blood and lymphatic system could also be identified as a-Cells or i-Cells.

Cells controlled by systems of ICMs other than the IGF system

There are many examples of cells and cellular properties that respond to other potential Trefones outside of the IGF system. Evidence that supports the CTC model is presented:- an ^a^T is produced in one cell type and the receptor is present in a different cell type; an ^i^T is produced by, and the receptor for the ^a^T is present in, the same cell type; the receptor for an ^a^T is present in a cell type but the cell does not produce the ^a^T; one Trefone added to a cell type stimulates the production of the couplet Trefone. There are also some specific examples of Couplet Cells and Couplet Trefones in the evidence presented in Additional file 1,15 “*Interacting Cells and Trefones not associated with the IGF System”.*

##### (iii) Reservations concerning the validity of experimental interpretations derived from cell-culture studies

Heterogeneity/variability of cells in culture

How is it known that a pure culture of cells is used in an experiment?

Cell lines may be contain a mixture of similar cells or contaminating cells or be misidentified or even replaced by cells derived from a different individual, tissue or species, as discussed in Additional file 1,14 Note 5. Further, during growth over a period of several days, a minor species or type may become dominant if the conditions favour its proliferation over the originally major species or type. Numerous examples of reported heterogeneous cultured cells are listed in Additional file 1,16 “*Examples of Heterogeneity/Variability of Cells in Culture”*.

If heterogeneity of cell lines is a major factor, then many incongruous results may be explained, including confusing the inhibition/stimulation effects of Trefones. Heterogeneity with just two or three types of cells present (a-Cell, i-Cell and possibly o-Cells), is a necessary requirement for the CTC model, but, of course, heterogeneity is not sufficient to prove the existence of couplet cells.

Are cell lines appropriate models for an understanding of normal cell activities?

The facts that immortalized cells are often transformed cells and often give rise to tumors when injected into animals are a clear indication of the abnormality of cell lines. Cancer cells generally have an abnormal karyotype with an abnormal numbers of chromosomes (polyploid or aneuploid) and with abnormal changes in chromosomal content due to translocations, deletions, duplications and/or inversions.

It follows that the cellular characteristics detected in experiments with cell lines will be misleading, in part, when trying to paint a picture of a normal cell in real life. This is elaborated on in Additional file 1,17 “*Cell lines are not Typical of Normal Cells; Warning - Deductions with Care”.* For example, in terms of gene expression, tumour cell lines are further removed from tumour tissue than tumour tissue is to normal tissue [102].

##### (iv) The concept of a-cells and i-cells, identified by the presence or absence of a cell membrane receptor and secreted Trefones

The CTC model requires the presence or absence of two receptors of different types to distinguish between coupled cell types. One cell would be identified as ^i^R⊕, ^a^R ⊖ and the other ^i^R⊖, ^a^R⊕. Further receptor-markers would be added as the cell line expands. This concept is not inconsistent with the immuno-identification of leucocytes and other cells by the presence or absence of a Cluster of Differentiation (CD) cell markers or other cell-surface proteins.

There are many examples of CDs that are receptors and this is emphasized in Additional file 1,18 *“Cell Receptors and Cell Markers”.* The presence or absence of receptors can then be used to delineate populations along with the ability (or not) to secrete specific proteins or other ICMs.

##### (v) A specific example of couplet cells and couplet Trefones

The current model has two Trefones and two coupled cells with appropriate receptors in a double paracrine loop. The following evidence, relating to the IGF system supports this model.

**Figure 6:**
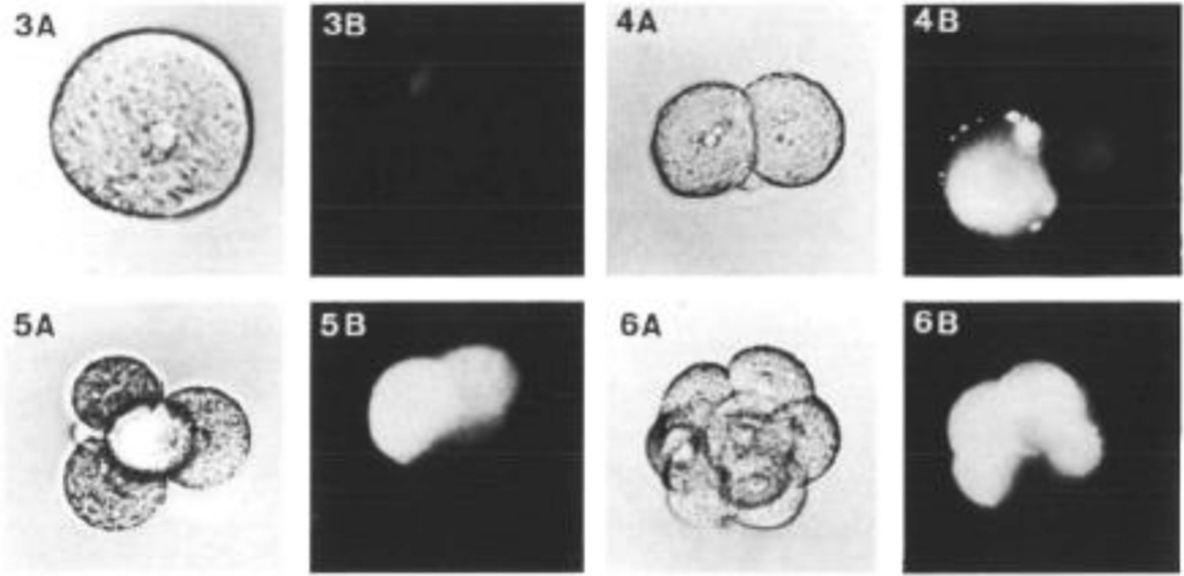
**Mouse preimplantation embryos.** (Reproduced with permission). **(A)***light microscopy to detect cells.***(B)***fluorescence microscopy to detect IGF-II receptor. (Figures*3*,*4*,*5*and*6*refer to Fertilized egg, 2-, 4- and 8-cell embryos).*

IGF-II is suggested to be the specific embryonic growth factor [103] and the IGF-II receptor is detected in exactly half the cells derived from the zygote [104]. Within this latter publication, Figures 3A to 6B (p.1058) show fixed and stained mouse preimplantation embryos. Figure 6 here, shows a copy of these results. This evidence clearly shows that only one cell of the 2-cell stage (4A versus 4B), two cells of the 4-cell stage (5A versus 5B) and four cells of the 8-cell stage (6A versus 6B) have the receptor for IGF-II. This is exactly the expectation of the CTC model and this is decisive evidence to support it. The publication [104] states “In the majority of early cleavage stage embryos observed, there was a greater degree of staining on ~ 50% of the blastomeres present (Figures 4, 5 and 6)”.

The other expectation is that only half (the other half) of the cells would produce and secrete IGF-II. IGF-II is expressed in early embryonic cells [105] in increasing amounts from the fertilized egg and from the 2-, 4- and 8-cell embryos as expected but the immunohistochemical evidence presented in this study refers only to the 2-cell and the 8-cell. The 2-cell does show a definite difference in stain for cellular IGF-II between the two cells but the 8-cell does not show a 4:4 clear difference. Rather, four are clearly stained as expected but another two are less stained, with the other two not discernible. However, it must be noted that cells with a receptor will also have some internalised IGF-II from receptor-mediated uptake, making it difficult to distinguish between a-Cells that synthesise and secrete IGF-II and i-Cells that internalise and degrade it. The two cells with less stain may have internalized some extracellular IGF-II. Apart from the less-than-perfect demonstration of the distribution of IGF-II, there is also a need to identify exactly that cells that produce the GF-II are the ones with absent IGF-II receptors. This would be needed to confirm directly the CTC model.

Internalization of a substance derived from another cell is a real issue in other similar situations. uPA (urokinase plasminogen activator) is detected in endothelial cells of tumour stroma but it is actually derived from fibroblastlike cells and internalized by the endothelial cells [106].

In both cases, the presence of the relevant mRNA would be more discerning than the presence of the protein itself.

#### (f) Evidence for couplet Trefones and/or couplet cells

##### (i) Examples of couplet Trefones with known couplet cells

The CTC model requires two specific Trefones that bind each other, and two coupled cells presumed to be derived from asymmetric cell division. Two examples are presented here.

(a) Insulin from Beta Cells and Glucagon from Alpha Cells of the Pancreas

(b) Gastrin from G-Cells and Histamine from ECL Cells, especially of the Stomach Additional file 1,19 “*Defined Couplet Cells for (I) Insulin and Glucagon and (II) Gastrin and Histamine”* contains the evidence of couplet cells for these Trefone couplets. The evidence includes the reciprocal stimulation of secretion of Trefones and the reciprocal stimulation of proliferation of each cell type by the Trefone of the other cell. The evidence is very consistent with the CTC model.

##### (ii) Examples of couplet Trefones (not binding proteins) with potential couplet Trefones but unknown couplet cells

The evidence presented here to support the CTC model is focused just on the coupling of Trefones, because, aside from alpha and beta cells, along with G-Cells and ECL-Cells, potentially coupled cells have not been identified. Additional file 1,20 *“Potential Couplet Trefones”* contains evidence for the following Trefone Couplets:-

(1) Dopamine and Neurotensin

(2) Serotonin and ACTH

(3) CGRP and IL-1

(4) Other Possible/Potential Examples:- Follistatin and Myostatin; Uteroglobin and Progesterone; Rankl and Osteoprotegerin; Sarcolectin and Migration Inhibitory Factor; Glutamate and Acetyl Choline; Wnt Interactants.

The evidence presented is consistent with the CTC model but there are gaps in the knowledge from lack of directed experiments to assess the expected properties of couplet Trefones and couplet cells.

##### (iii) Other evidence that is consistent with this CTC model

There are a number of other examples of ICMs and cells which have properties expected of components of the CTC model. When some of these examples are taken individually, they may seem of less consequence, but, in total, they reinforce the more specific examples above and expand the scope of the model. Additional file 1,21 *“Further Examples of Potential Trefone Couplets and Potential Cell Couplets”* contains a variety of examples not inconsistent with the CTC model.

### (3) Variations and extensions of the model

#### (a) An alternate of the model; measurement of the complex (TCC) or free Trefone

One purpose of the formation of TCC is to allow a relative count to occur to ensure harmony of couplet cells. An alternative to the detection of an extracellular TCC by a membrane receptor within the CTC(EC) model, would be that the TCC forms in an intracellular (IC) compartment to allow a “count” to assess Trefone balance – the CTC(IC) model. The TCC would be formed between one ^n^T, synthesized by one n-Cell, and the other ^n^T, internalized by receptor into that cell but derived from the other couplet cell. Such intracellular contact between the two ^n^Ts and the formation of an intracellular TCC could occur in a common compartment of the endoplasmic reticulum (ER) or within the Golgi Network (GN). Given that secretion occurs via the ER and the GN, and receptor-internalization occurs via endosomes with possible retrograde transport [107,108], the idea of an intracellular (IC) compartment for the formation of TCC is feasible. A certain amount of the normally secreted Trefone (^a^T) or a derivative would be hived off to interact with incoming ^i^T.

If, in the a-Cell, which synthesizes and secretes ^a^T and endocytoses ^i^T, the two Trefones interact in a common IC compartment to form an IC TCC, the consequent concentration of TCC and the Free ^a^T and the Free ^i^T from the equilibrium will reflect the balance of Trefones. For example, a high level of Free ^i^T translocated from that common compartment to the nucleus with a low level of TCC, would indicate a deficiency of ^a^T and produce a stimulation of ^a^T synthesis or even a major cell change e.g. cell division.

FGF and its binding protein IGFBP may be an example of this. The major isoform of fibroblast growth factor 3 (FGF-3) is partially secreted and partially distributed into the nucleus [109]. This could be in keeping with an a-Cell that synthesizes and secretes the ^a^T (FGF) but with some hived-off into a nuclear compartment where it comes in contact with incoming ^i^T (endocytosed FGF binding protein, FGFBP). FGF-2 could be different. It occurs in more than one form, with a 18 kDa form that is cytoplasmic and two forms (21–23 kDa) that are nuclear [110]. The difference is a 37-amino acid nuclear localization signal so that the larger form could interact with the FGFBP to form the IC TCC. The other half of the CTC model would also occur; cells that synthesize FGF receptor-1, also produce an IC FGF binding protein – a truncated receptor (FGFBP) which localizes to the nuclear membrane [111]. FGF receptor-related proteins migrated multidirectionally within chondrocytes: receptor accumulated in plasma and nuclear membranes, while truncated receptor was detected on the nuclear membrane [111]. These latter cells would correspond to i-Cells where some of the FGFBP produced and retained in the cell could interact with incoming FGF (^a^T).

Some steroids have intracellular receptors in specific tissues. Estrogen receptor, ER (alpha or beta), with bound ligand (estrogen, E) will activate gene transcription in i-Cells. The CTC model would require that the concentration of this ER:E complex in a particular i-Cell would be controlled by the level of free E, after internalization of extracellular E synthesized from the a-Cells. The level of free E would in turn be controlled by the presence of intracellular SHBG, its Trefone couplet, synthesized by that individual i-Cell. Indeed, SHBG is colocalized with ER in epithelial and muscle cells of Fallopian tube tissues with SHBG of both, intra- and extra-cellular origin [112].

Certainly the CTC(IC) model and possibly the CTC(EC) model would require an IC compartment for assessment of the extracellular balance of Trefones and thus, by extrapolation, the balance of cells. In both cases, the TCC binary complex may directly affect nuclear activity.

Binary complexes are known to interact with DNA or transcription factors to regulate cellular activity. Additional file 1,22 *“Examples of Cellular Regulation by Complexes”* contains some examples of these complexes, in general, which have an effect on cellular activity by interaction with nuclear components. The alternative option that the free levels of Trefones affect nuclear activity is still feasible as discussed for estrogen.

Overall, while the intracellular formation of a complex of ^a^T and ^i^T is feasible and the TCC could be nuclearly active, the advantage of the measurement of extracellular TCC is that it allows the cell to assess the dual production of Trefones by the whole family of a-Cells and i-Cells rather than just by individual cells. Of course, even if there is no input from a receptor-mediated uptake of the complex into the re-equilibration that occurs intracellularly, an extracellular complex will still form to restrict the amount of incoming free Trefones.

#### (b) Variations of the model; other intercellular communication mechanisms

##### (i) Trefones:- enzymes with anti-enzymes

A variation on the basic CTC model is to include an unexpected type of Trefone, namely proteolytic enzymes. This inclusion is focused on their interaction with anti-enzymes (protease inhibitors) and is not related to their enzymic activity as such. Each couplet of enzyme and inhibitor binds to form a complex, a TCC, and each has proliferative abilities outside their catalytic and anticatalytic activity. Evidence for the following potential couplets is presented in Additional file 1,23 “*Proteolytic enzymes and their inhibitors (mainly)”.*

1. Elastase and Alpha-1-antiprotease

2. Matrix Metalloproteinases (MMPs) and Tissue Inhibitors of Metalloproteinases (TIMPs):

3. Kallikrein and Kallistatin (=Kallikrein Binding Protein)

4. Thrombin and Antithrombin III

5. Plasmin and Alpha-2-antiplasmin

6. Other Possible/Potential Enzymes as Trefones

The evidence for the CTC model is not particularly strong in these pairs but again the data are consistent with the model although, again, lacking in directed experiments to assess the properties expected within the model.

##### (ii) Trefones and classes of couplet cells – the CTC model expanded

To this point, the CTC model has, in terms of chemical communications, involved the interaction of soluble couplet Trefones and the requisite formation of a complex (^a^T:^i^T). The purpose of the TCC is to connect the interaction of two couplet cells where these cells were derived from asymmetric cell division. This is the heart of the CTC model.

However, there are other possibilities, although with less evidence to support them. The CTC model described is expanded here into a larger group of modes of communications where possibly, a reciprocal dependence between cells is maintained. For this speculative expansion of the model, the definition of Trefone is expanded to include Trefones that are membrane bound i.e. Fixed Trefones. The Fixed Trefone will still have two functions. Firstly, it may either contribute to intercellular binding or directly affect metabolism as previously. Secondly, it would still have an intracellular effect to induce a change in cell fate. A soluble Trefone would normally be part of a couplet of Trefones derived from, and controlling, reciprocatively, a couplet of cells via internalization of these Trefones. A Fixed Trefone could also be part of a couplet Trefone and couplet of cells if they form a double, reciprocal communicative interaction to induce especially a change in cell fate. In Additional file 1,24 *“Expanded Definition of ‘Trefone’ and Classes of Couplet Cell Interactions”,* seven classes of cellular activities are proposed including Fixed- and Soluble- Trefone interactions and shedding or regulated intramembrane proteolysis. In addition, the possible interaction of other proteolytic and transferase enzymes, along with proTrefones and derived Trefones are incorporated into intercellular signalling couplets.

#### (c) Extensions of the model

##### (i) Couplet progeny and secondary Trefone couplets

The formation of a multicellular organism from a fertilized zygote likely involves AsCD from the first cell division [113] with subsequent repeated rounds of AsCD and SCD within lineages. Thus, using Couplet Cells and the proposed Trefones, there can be a critical balance and control to the growth of cells in the early stages of development which is continued into the adult with a reserve of stem cells and other pluripotent precursor cells. Some of these cells are stashed as safeguards for emergency use on the journey to the complete organism and all are essential for turnover, maintenance and repair of tissue and organs incessantly until death. However, the balance is not only between two cell types which are harmonized by their primary Trefone couplet. A lineage of cells, derived from an a-Cell early in the lineage, will need control molecules at every level of differentiation – there would be a need for secondary, tertiary, quaternary … couplet Trefones and even cross-linking Trefones.

A secondary couplet of cells would refer to the two progeny of an a-Cell derived from a subsequent AsCD. These two a-cell descendants would still produce the a-Trefone required by the i-Cell of the original couplet (and would have the receptors for the i-Trefone) but the progeny would also be a coupled as new, more differentiated a- and i-Cells. The new couplet cells would now have their own primary couplet Trefones but each would still produce the now-secondary a-Trefone. These couplets are further discussed in Additional file 1,25 *Note 1“Extended Trefone Couplets”*.

There are many examples of the synergistic effects of two hormones. These could be explained by primary and secondary levels of Trefones. Both IGF and EGF interactions regulate epidermal growth and hyperproliferative skin diseases [114]. An IGF will also promote proliferation of chromaffin cells by itself but acts synergistically with FGF-2 and NGF [115]. In addition IGF promotes proliferation of rabbit aortic smooth muscle cells [116] and of oligodendrocyte progenitor cells [117] and acts synergistically with FGF-2 for both types of cells. IGF-I and estrogen act independently and synergistically on breast cancer cells (e.g. estrogen receptor alpha- positive MCF-7 cells) [118].

Cells existing without their primary or secondary Trefones may dedifferentiate to a previous stage in the lineage. In this dedifferentiation, cells do not simply lose the mature coupled-cell phenotype. The couplet cells must actively reverse the normal differentiation program to revert to an earlier stage of the lineage dependent on the remaining Trefones in the environment.

##### (ii) Cells that go it alone - Singlet cells

Some “labour” tasks are performed by single purpose, “disposable” cells. For example, mammalian erythrocytes are terminally differentiated cells and survive on average 120 days in humans. They derive from reticulocytes which themselves do not divide [119] but they are genetically programmed to change into the erythrocytes. Reticulocytes would be singlet cells as they have no couplet cells. The properties of singlet cells are further discussed in Additional file 1,25* Note 2 “Singlet Cells”.*

Other examples of singlet cells may exist. A self-sufficient organism would be impervious to hazardous substances or other life forms in the environment. In reality, a human has allergic and immune responses to counter harmful components of the environment. It may be then that there can be multifunctional singlet cell equivalent to a half of a “Cell and Trefone Couplet”. A B- or T-cell could have a receptor to a foreign molecule which stimulates the release of an antibody to form a complex with the antigen. As a half of a “Cell and Trefone Couplet”, this cell with an antigen receptor would also have a receptor for the antibody-antigen complex. Certainly some cells have receptors for antibody-antigen complexes - many lymphoid or myeloid cells, such as B lymphocytes, macrophages or dendritic cells do [120]. The receptor-mediated uptake of the complex (equivalent to a TCC) could stimulate the formation of more singlet cells by SCD.

## (C) Conclusions

### Preamble

The starting point for the model offered here is basic. The two types of cells derived from an asymmetric cell division are connected by Trefone interactions. This coupling continues in the progeny throughout the lineage, adding complexity to the ability of cells to respond to extracellular messages. For the development of a cell line in an embryonic state, the original cell of the line (°C) has its chromosomes and DNA programmed so as to produce “n” specialized cells if the development occurs in the “prerequisite” environment. This may include not only adequate nutrients but also the specific growth factors, differentiation factors and other ICMs and this required environment will be provided by the concurrent formation of other surrounding cell lines. There would be various branches and sub-branches of each cell line, along with terminally differentiated cells, in the subsequent progeny of cells as the program unfolds. But all of these cells need to be connected and in communication with each other in some way to allow a whole organism to form even with separated couplet cells and to be sustained in the face of cell death. The real life situation is then complex and secondary, tertiary etc. Trefones could provide the family ties and the cohesion needed in an organized structure. In an organism, there may be only a low number of original Couplet Cells (e.g. Totipotent stem cells), more progenitor Couplet Cells (e.g. pluripotent stem cells), many mature Couplet Cells (which are more likely to divide by symmetric cell division) perhaps along with a multitude of dedicated singlet cells. The cells in a lineage will be identifiable by the presence ⊕ or absence ⊖ of the various receptors that respond to the couplet Trefones of the various parental cells. This identication is clearly similar to the use of CD markers to classify cells.

### The importance and relevance of the model:- applications in medicine

#### (a) Diabetes

Evidence is presented to support the basic tenet that insulin and glucagon are Trefone couplets with the beta and alpha cells being the cell couplets (See Additional file 1,19).

Glucagon stimulates both insulin secretion by, and proliferation of, beta cells via glucagon receptors. Reciprocally, insulin stimulates glucagon secretion by, and proliferation of, alpha cells via insulin receptors. These effects obviously occur within the organized architecture of the pancreatic lobes. Within the pancreas, where the local concentrations will be much higher than in plasma, and in the presence of zinc, the complex of insulin and glucagon will exist in a significant concentration. Reciprocating, pulsatile production of insulin and glucagon will occur within the pancreas reflecting the release from the couplet cells responding to their respective stimulating Trefones and the TCC.

This simplicity obviously does not take into account the somatostatin-secreting (D) cells, the pancreatic polypeptide-secreting (PP or F) cells nor the organized distribution of the cells among the four pancreatic lobes. Also, the effects of ICMs from distant organs (epinephrine, glucocorticicoids, glucagon-like peptide) need to be incorporated into any overall understanding of pancreatic function.

### (b) Cancer

The application of the CTC model to cancer has been discussed in Additional file 1,26 “*Background for an Understanding of Cancer Research”.* Many cancers have associated changes in receptors due to oncogenes and the apparent autonomous nature of the growth of cancer cells has been long recognised and has been explained by the acquired ability of these cells to act in an autocrine way [121]. This means that the cell producing a critical growth factor also has a receptor for it and responds in a vicious circle of cell cycling and stimulation in an incestuous way. The CTC model is different and one explanation using the model would have the two coupled cells stimulating each other without the restraint of a receptor for the TCC which may have mutated. As oncogenes are involved in growth factor/receptor pathways, then the CTC model links oncogenes, growth factors and their intracellular transduction paths with possible causes of cancer just as the autocrine concept is so linked [122]. For example, Schwannoma cells strongly overexpress the IGF-I receptor and secrete IGFs to produce a possible autocrine system [123] but again this is not inconsistent with the CTC model with couplet cells involved.

Some mutation or epigenetic change in the Trefone, Trefone receptors, proteins of internalization, transporters to the nucleus or in the transcription factor(s) binding the Trefone, would initiate an abnormal cellular state to stimulate the progress to a cancer cell. By a sequence of AsCDs and/or SCDs, cancer cells (CCs) may be produced following some mutation in an a-Cell or i-Cell. The proliferation and differentiation of these abnormal CCs will be controlled by the Trefones in a normal way within the communal environment of a tumour or in the tissue location of the progenitors of a liquid cancer. An autonomous cell may also evolve from production of a singlet cell which is not part of a CTC. Singlet cells will still require nutrients and Trefones for cell division but they will not be constrained by the formation of a Trefone couplet complex. Whether the CCs are abnormal couplets of cells or just singlet cells, they would be stimulated by Trefones from surrounding normal cells or from cells attracted to the site and could then flourish as a cancer.

Again, it is noted that AsCD by B (i) in Figure 1 (Additional file 1,3* Note 2 “Categories of Symmetric and Asymmetrical Cell Divisions”)*, is not normally favoured because the single C/D cell has no controlling partner. This C/D without a controlling couplet or a similar “singlet” cell, would have an autonomy property shared with cancer cells.

Within the CTC model, the response to an imbalance of Trefones may involve interconversions of a-Cells and i-Cells. This may require SCD, AsCD and dedifferentiation. The latter may be a part of sarcomagenesis [124] and hepatocellular carcinomas may arise by dedifferentiation of mature liver cells with maturation arrest of progressively maturing liver stem cells [125]. Certainly, differentiation and dedifferentiation are a big part of the process of cancer-cell formation [126].

### (c) Stomach function – *Helicobacter pylori*

Hypergastrinemia in transgenic mice who are also infected with *Helicobacter* infection leads to the accelerated development of intramucosal carcinoma [127]. One of the virulence factors of *H. pylori* is the oncoprotein cytotoxin-associated antigen A (CagA). Overexpressed CagA itself, which affects various intracellular pathways, is sufficient to induce multiple malignancies, including gastric cancer, in transgenic mice [128]. In addition, H. pylori induces epigenetic alterations, such as DNA methylation and histone modification which could influence cancer development [129]. But, although this relationship between *H. pylori* infection and gastric cancer is established, knowledge of the exact mechanism of tumor initiation is lacking [130].

One possible mechanism relates to the observation that CagA specifically interacts with PAR1/MARK kinase [131], which has an important role in epithelial cell polarity as one of the six par genes essential for the asymmetric division of the *C. elegans* zygote [132]. However, the Par1-protein kinases are evolutionarily conserved from yeast to humans and in mammals and there are several Par1-related proteins. Since CagA affects both polarity and growth regulation, then the former could direct the abnormal proliferation of epithelial cells that causes gastric carcinogenesis in humans [133]. If CagA prevents the asymmetric cell division (AsCD) options described in Figure 1 (Additional file 1,3 *Note 2“Categories of Symmetric and Asymmetrical Cell Divisions”)* or causes an aberrant AsCD, then the homeostasis of the couplet cells (G- and ECL-cells) which produce the Trefones gastrin and histamine, respectively, would be disrupted and abnormal proliferation would ensue, perhaps with excess of either or both Trefones, ulceration and/or cancer.

### (d) Immune response

Asymmetric cell division (AsCD) occurs also in immune responses. AsCD of T lymphocytes occurs in the initiation of a response in adaptive immunity e.g. response to a microbe. The determinants of AsCD (including Numb and polarity regulators Scribble and aPKC) are segregated while there is prolonged interaction between antigen-presenting cell and the T cell, before it divides. The two progeny T cells are then fated differently toward effector and memory lineages [134-136]. Within the full lineage of T cells, the multipotent progenitor (MPP) cell may form the common lymphocyte progenitor (CLP) plus the common myeloid progenitor (CMP) by AsCD while the double positive DP thymocyte (derived from the CLP) may divide by AsCD to form CD4^+^ and CD8^+^ T cells.

Within the full lineage of T cells, the multipotent progenitor (MPP) may form the common lymphocyte progenitor (CLP) plus the common myeloid progenitor (CMP) by AsCD, as might the CMP dividing to form the megakaryocyte-erythroid progenitor (MEP) plus the granulocyte-macrophage progenitor (GMP). AsCD could also be involved in the double positive DP thymocyte dividion which forms CD4^+^ and CD8^+^ T cells. Subsequent cell division of the CLP, GMP and MEP would also likely involve AsCD.

There is also evidence of reciprocal interactions, as suggested for the Trefones of coupled cells, between dendritic cells and Gammadelta T cells, two cell types of innate immunity involved in the initiation of the immune response against *Mycobacterium* tuberculosis infection. Dendritic cells produce IL-12 which stimulates gammadelta T cells while these T cells produce interferon-gamma which stimulates (BCG) infected dendritic cells, in a reciprocal functional relationship between these cell populations [137].

If B-cells, which produce an antibody against a foreign antigen, respond to the endocytosis of the whole molecule, then these singlet cells, as half of the Cell and Trefone Couplet (CTC), should endocytose the antibody-antigen (immune) complex as well. Indeed, various Fc receptors (e.g. FcgammaRII [138]) will bind the immune complex (and less so the antibody), and various immune cells endocytose the immune complex [139]. While both B cells and macrophages can endocytose the immune complex *in vitro* via the Fcgamma Receptor, dendritic cells may be more important in the uptake and processing of the immune complex via this receptor *in vivo*[140].

Within the CTC model, an antibody (Ab) is not just a protein that bind antigen (Ag); it is a Trefone without a couplet cell. The specific immune cell and Trefone constitute a half a CTC because the cell is reactionary to an antigen which is made to be a specific stimulating Trefone. The Ab is first secreted from the cell by antigen stimulation (i.e. via a receptor) and then the complex with antigen is internalised via receptor-mediated uptake into the singlet cell. The actual deficiency of Ab (as measured within the cell by the amount of Ab-Ag complex relative to the amount of Ag, derived also from direct receptor-mediated uptake) stimulates proliferative cell division and more Ab production. Also, both activating and inhibiting effects occur. Activating-type FcR, such as FcγRI, FcγRIIa and FcγRIIIa which are characterized by the presence of a cytoplasmic immune-receptor tyrosine-based activation motif (ITAM) sequence, promote disease development and the inhibitory-type, FcgammaRIIB which is characterized by the presence of an immune-receptor tyrosine-based inhibitory motif (ITIM), can suppress antibody-mediated autoimmunity [141]. However, there are ambiguous effects of ITAMs and ITIMs [142] which could be explained by whether they are part of receptors for the antibody only or for the immune complex also.

### (e) Atherosclerosis

Atherosclerosis and cancer share features of cellular heterogeneity and have common molecular pathways of development and progression. Growth factor (Trefone) interactions with receptor tyrosine kinases feature in both, along with altered cell adhesion and angiogenesis [143]. Further, the cells and intercellular molecules of the immune system are indispensable for the development and progression of both cardiovascular disease and cancer [144].

In addition, the expression of proteases is altered in thrombolysis of atherosclerotic plaque expansion and in the metastasis of malignancy [143], and control of cell proliferation is crucial to both. In this respect, there is evidence for a definitive direct proliferative effect of TIMPs and of MMPs (as discussed earlier). MMP-12 (macrophage elastase) is expressed in human atherosclerotic lesions in carotid endarterectomy samples but not in normal arteries [145] and it is upregulated in atherosclerotic lesions in transgenic rabbits overexpressing MMP-12 [146]. TIMP-3(-) foamy cell macrophages (FCMs) occur in the deeper layers of the plaque and have an increased proliferation rate compared to TIMP-3(+) cells [147]; MMP-12 is also upregulated in FCMs compared to nonfoamy macrophages [148]. Related to this, is the existence of two subpopulations of foamy cell macrophages (FCM), MMP-14^Hi^TIMP-3^Lo^ and MMP-14^Lo^TIMP-3^Hi^. MMP-14 is a membrane-type MMP that binds TIMP-3, so they are candidates to be Trefones produced by possible couplet cells of the two adjacent populations of cells - the TIMP-3 negative FCMs appearing to form discrete islands or nodules within surrounding TIMP-3 positive cells [147].

### (f) Chronic airway inflammatory disease

The concentration of the complex of neutrophil elastase naturally bound to alpha1-antiprotease (i.e. alpha1-anti-trypsin, AAT) in alveolar lavage fluid has been used to assess lower respiratory tract inflammation and potential lung damage by smoking [149]. The change in the amount of complex observed may reflect a variation of the association constant for the pair in the lower tract compared to plasma to reduce neutrophil elastase inhibitory capacity [150]. This imbalance of protease/antiprotease, where elastase (or other protease) is present in excess of its major inhibitor, seems to occur in a variety of chronic airway inflammatory diseases that exists in cystic fibrosis (CF) and non-CF bronchiectasis airways and of chronic bronchitis [151,152]. In the *Pseudomonas*-infected cystic fibrosis (CF) lung, the secretory leucoprotease inhibitor (SLPI) of elastase from the neutrophil was decreased in bronchoalveolar lavage fluid because of high levels of neutrophil elastase activity which cleaved the SLPI, thereby increasing the protease:antiprotease complex [153]. The protease-antiprotease imbalance in associated with emphysema and chronic obstructive pulmonary disease [154]. If the proposed Trefone couplet of elastase and AAT are proliferators and are both derived from neutrophils, cellular inmbalance of the different types of neutrophils may be involved in lung diseases.

### (g) Bacterial persistence

Persisters are a small subpopulation of microbial cells that can survive antimicrobial treatments although being genetically susceptible, by entering a dormant state and they may not be eliminated by the immune system. In studies of E.coli, in addition to the wild-type cells, there may be subpopulations of Type I and Type II persister cells. Type I cells are growth-arrested cells, produced at the stationary phase of the previous growth cycle by a starvation signal, and are present in the next culture in proportion to the inoculum size. Type II cells are generated continuously in the exponential growth of normally growing cells [155,156]. Persistence is associated with the formation of biofilms in which bacteria may grow as a consortium of different species where cells in different zones of a biofilm are in different metabolic states [157].

In the CTC model suggested here, the following is a scenario. If the normal cell were the a-Cell, then the persister cell would be the i-Cell. The a-Cell is growing exponentially because the medium contains the growth factors and nutrients needed by it. For example, the i-Trefone is in the medium in excess, stimulating the growth of the a-Cell by SCD. As the a-Trefone is released from the a-Cell, it binds the i-Trefone to form the complex. There is little free a-Trefone to stimulate the i-Cell to grow by SCD; in anything, AsCD could occur to boost the a-Cell’s production of the a-Trefone for homeostasis. In that growth state, added antibiotic will kill the a-Cells but not the dormant i-Cells. When placed in new medium after the death of the a-Cells, some i-Cells will divide by SCD and some by AsCD to produce a-Cells.

While it is tantalizing to associate the current model with Toxin-Antitoxin modules [158-160], this would require the production of the toxin and antitoxin by two different cell types and an extracellular meeting of the proteins or RNAs. This is realistically possible in that bacterial biofilms are structured communities of cells enclosed in a self-produced polymeric matrix containing polysaccharides, enzymes, other proteins and DNA [161] where the extracellular DNA is not from cell lysis but is deliberately released [162]. However, there is no evidence of extracellular toxin:antitoxin complexes.

### (h) Alzheimer’s disease

The β-amyloid precursor protein (APP) of Alzheimer's disease is a type I transmembrane protein with multiple spliced isoforms. The three most abundant isoforms of APP are APP_770_, APP_751_, and the predominantly neuronal APP_695_ and from these either the P3 peptide or the Alzheimer's amyloid protein (Aβ) is proteolytically derived. Regulated intramembrane proteolysis of APP occurs by secretase complexes and an intracellular peptide AICD is produced. With further proteolytic cleavages of the APP residue in the membrane, two extracellular peptides are produced, soluble APP (sAPP) and either the P3 peptide normally or amyloid beta (of 36–43 amino acids) in the amyloidogenic pathway [163].

Both AICD and the sAPP are proliferatively active and, within couplet cells, AICD would be the Trefone for the cell that produces it internally, say the a-Cell, and sAPP would stimulate the i-Cell via receptor-mediated uptake.

The AICD translocates to the nucleus and has been implicated in transcriptional regulation by forming a multimeric complex with the nuclear adaptor protein Fe65 and the histone acetyltransferase Tip60 [164]. It thus negatively modulates neurogenesis [165] and inhibits proliferation but promotes differentiation in neuronal cells. It inhibits canonical Wnt signalling in a GSK3β kinase activity-dependent manner and its loss produces an increase in response to Wnt/β-catenin-mediated transcription [166].

There have been several reports of the proliferative, stimulatory ability of the sAPP (usually sAPP695), in fibroblasts [167], neural stem cells [168,169], a thyroid epithelial cell line (FRTL-5) [170] a human keratinocyte cell line (HaCaT) [171], in adult neurogenesis [172] and in neural progenitor cells, mesenchymal stem cells and human decidua parietalis placenta stem cells [173].

Specific cell binding (indicating a receptor) have been detected in a human keratinocyte cell line (HaCaT) [171] and in the largest neurogenic area of the adult brain (the subventricular zone of the lateral ventricle) and binding occurs on specific progenitor cells that respond to EGF [172]. It has been shown to be an active regulator of transthyretin and Klotho gene expression [174] and has many other suggested roles [175].

While these postulated Trefones are active, other components of the amyloid system also have some biological activity. The Abeta peptide (Abeta42), produced in the amyloidogenic pathway, has proliferative properties. It induces neurogenesis in cultured neural stem cells [176] and induces the proliferation of a mouse microglial cell line (Ra2) [177]. However it has an inhibitory effect on growth of some tumout cell lines [178]. This peptide has receptors and secreted Abeta can bind to cell surface receptors (e.g. LRP, RAGE, FPRL1, NMDA receptors and α7nAChR [179]), and can be internalized. While Abeta(1–40) and Abeta(1–42) predominate in the brain, the fragment Abeta(25–35) is also present in elderly people and its aggregation properties may make it more toxic than the fibrillogenic Abeta42 [180]. Aβ(25–35) itself may be toxic as its oligomer inhibits the proliferation of neural stem cells [181]. Internalised Abeta, along with intracellularly produced Abeta, can accumulate and form oligomers of Abeta, which, along with intracellular tangled tau protein and extracellular amyloidal plaques, strongly contribute to Alzheimer’s disease. As aging is often associated with Alzheimer’s disease, one might expect that epigenetic modifications would contribute to the dysfunction of these proteins and/or the enzyme complexes involved in amyloid metabolism [182]. The interaction of AICD and Tip60, a histone acetyltransferase, might be relevant to this specific epigenetic modification.

### (i) Other diseases

While there is little evidence for the CTC system in the following diseases, the involvement of couplet Trefones, or Trefone plus receptor, in these diseases may be a clue to verify a full CTC system.

#### Schizophrenia

The NRG1 growth factor and its receptor, ERBB4, have been shown to modulate neuronal functions and have been identified as leading schizophrenia risk genes. The ERBB4 intracellular domain (E4ICD) derived from proteolytic cleavage, regulates neuroregulin-1-induced gene expression in hippocampal neurons [183]. The NRG1 and the ERBB4 would need to be on separate couplet cells to be a CTC system.

#### Psoriasis

IGF-I receptor and EGF receptor expressions are increased in psoriatic epidermis with the the pattern of IGF-I receptor expression increasing with increased keratinocyte proliferation. Both growth factor receptor systems may control cellular expression and differential regulation of epidermal proliferation [184]. As an alternate system, the keratinocyte growth factor (KGF), which is also a potent mitogen for wide variety of epithelial cells, and keratinocytes in particular, would seem a logical one. KGF and its receptor were found to be frequently elevated in psoriatic skin specimens and distributed within different areas of the skin - increased KGF expression occurred in the dermal layer, while receptor expression occurred in the basal layer of keratinocytes in normal skin and in addition, in the suprabasal layers of the psoriatic epidermis where there was expanded proliferative keratinocyte pool [185].

Asymmetric cell division is also a crucial element of skin growth.

#### Graves’ disease (GD)

IGF-I and its receptor seem to be involved in the autoimmune syndrome GD. Insulin-like growth factor-1 receptor (IGF-IR) has two subunits, a ligand binding site on extracellular IGF-IRα and a tyrosine phosphorylation site on IGF-IRβ. Upon IGF-I or GDIgG stimulation, IGF-IRα accumulates in the nucleus specifically in orbital fibroblasts from GD patients [89].

#### Rheumatoid arthritis (RA)

RANKL and its potential Trefone couplet osteoprotegerin are both increased in RA [186].

### Restrictions and final observations

#### (a) Restrictions

Not every molecule that stimulates a cell to change its metabolism or to secrete a product will be a Trefone for that cell. A growth factors or hormone may stimulate certain cells to metabolic change but may not be a Trefone for those cells. For example, glucagon-like peptide-1 may stimulate insulin release from beta-pancreatic cells but is not a Trefone for beta cells. A substance could be a Trefone for a particular pair of cells but just a metabolic stimulant or inhibitor for other types of cells. Equally every receptor ligand is not a Trefone. Ligands may be differentiation agents (DA) or factors (DF). IGF-I is not necessarily a primary Trefone for every cell it affects; it may be an inhibitor of growth of a cell by binding an IGFBPn which may be a Trefone, coupled with IGF-II, for that cell.

Although the basic difference between a-Cells and i-Cells is the presence or absence of a Trefone and receptor, there will be other cellular infrastructure changes to accommodate the production and reception of the respective Trefone and TCC and also other minor or major differences reflecting the specific functions of the cells formed in this “division of labour”.

#### (b) Final observations

Every cell is in limbo in its differentiated state; in the absence of Trefones, it dedifferentiates and reverts back to its default ego state.

This CTC model may interlock with the idea of “complementarity”. An a-Cell will produce a Trefone (perhaps a peptide) and an i-Cell will produce a receptor for the peptide. This could be achieved within a “complementarity” framework. Evidence has been provided that certain mRNA sequences, complementary to the mRNA sequences for some receptors, were highly homologous to those of their ligands [187]. This was illustrated for the ligands (Trefones) epidermal growth factor, interleukin-2 and transferrin with their respective receptors. This would mean that after ACD, one cell would make a receptor from one strand and the couplet cell would make the Trefone/ligand form the other strand of the same allele and *vice versa* for the other Trefone and receptor. The complex formation of a-Trefone with i-Trefone, a-Trefone with i-Receptor, a-Trefone:i-Trefone complex receptor would reflect molecular complementarity [188].

In an evolutionary sense, given the usual lack of transcription in a single cell of both strands of DNA for a particular gene, then couplet cells could have developed where each uses a certain strand of the double DNA; “…. the mere transcription of a DNA sequence by one cell and the complementary sequence by another could allow for cellular recognition and communication via the interacting protein products” [187].

The proposed model is an approach to simplify the multitude of reported experimental results derived from the properties of cancer, modified and normal cells, often growing in size and/or number in a synthetic environment. In many cases, the results appear to be a myriad of disconnected dots of information, orphans looking for a place in a confusing network of life without structure. The conceptual simplicity of this Cell and Trefone Couplet model with consequent division of labour is in contrast to the vastly complicated metabolic pathways with a network of numerous control loops which are often inflicted on a single cell.

However, the plausibility of a hypothesis is no argument for its veracity or its generality. Hopefully too many facts have not been twisted or manipulated to fit the model. Also, hopefully, this model will stimulate more novel experimental designs.

**Quote:-** “It may be useful to think of a cell culture as being in equilibrium between multipotent stem cells and mature differentiated cells and to suppose that that the equilibrium may shift according to the environmental conditions” [189].

### Summary

A model is offered to explain the harmony of cellular life in multicellular organisms. Two different cells, referred to here as the a-Cell and the i-Cell, are derived from the parent cell, referred to as the original o-Cell, by cell division. These cells, formed by a type of asymmetric cell division, are referred to as “Couplet Cells” and in the basic model, are linked by two specific molecules, one produced from each cell, which produce a binary complex. The cells are also linked by the presence on each cell of a receptor which binds the specific molecule secreted by the other cell; the a-Cell has a receptor that binds the molecule from the i-Cell and *vice versa*. This reciprocal interaction allows intercellular communications between the two progeny, which will regulate the number and activity of the two cells. This interconnection of cell couplets can flow onto other cells of a lineage derived from either the a-Cell or i-Cell.

The specific molecules which are coupled by complex formation are named “Trefones” and with “Couplet Cells” and “Couplet Trefones”, the model is referred to as the “Cell Trefone Couplet” (CTC) model.

The basic CTC model then equates to asymmetric cell division to produce a double paracrine system between the two progeny (couplet cells), with formation of a binary complex between the specific molecules secreted from each cell. It is suggested that the rationale for the asymmetric cell division is to allow a “division of labour” within a multicellular organism.

The evidence for the model focuses on the insulin-like growth factor (IGF) system and other similar systems with couplet molecules that stimulate or inhibit cell metabolism and proliferation. The model has implications for various medical conditions e.g. diabetes and cancer.

This is my own hypothesis and I am the only contributor. There are no acknowledgements to be made and I have no competing interests.

## Competing interests

The author declares that he has no competing interests.

## Supplementary Material

Additional file 1 1. “Terminology”. 2. “Change in Cellular Characteristics”. 3. Note 1 “The Roles of SCD versus ACD”. Note 2. “Categories of SCD and ACD”. Note 3 “Further Examples of these AsCD Categories”. Note 4 “Change of differentiation type.”. 4. “The Effects of Growth Factors and other Bioactive Molecules 5. Note 1, “Additional Information on the Definition of a Trefone”. Note 2 “Clarification on Ligand and Binding Protein Interaction”. Note 3 “Cell Couplets – AsCD and the a-Cell and i-Cell”. Note 4 “Drosophila – Potential Trefones and ACD”. 6. “Calculated Levels of Trefones and TCC, with Cellular Actions”. 7. “Receptors for the Six IGFBPs and Cellular Effects”. 8. Note 1 “Extracellular Binding Proteins/ Soluble Receptors”. Note 2 “Cell Receptors for Binding Proteins/Soluble Receptors”. 9. Note 1. “Further Evidence for a Receptor for TCC”. Note 2 “Problems with Cross-linking Experiments”. 10. “Evidence for Binary Receptors aside from the IGF System”. 11. “Evidence for Two Receptors for a Particular Trefone, outside the IGF System”. 12. Note 1 “Mechanisms of Nuclear Localization”. Note 2 “NL of IGFBPs”. Note 3 “NL of Potential Trefones other than the IGF System”. 13. “Evidence Supporting the Existence of a-Cells and i-Cells”. 14. “Candidates with mainly a-Cell-Type or i-Cell-Type Characteristics”. 15. “Interacting Cells and Trefones not associated with the IGF System”. 16. “Heterogeneity/Variability of Cells in Culture.” 17. “Cell lines are not Typical of Normal Cells”. 18. “Cell Receptors and Cell Markers”. 19. “Defined Couplet Cells for Insulin and Glucagon; Gastrin and Histamine”. 20. “Potential Couplet Trefones”. 21. “Further Examples of Potential Trefone and Cell Couplets”. 22. “Examples of Cellular Regulation by Complexes”. 23. “Proteolytic enzymes and their inhibitors”. 24. “Expanded Definition of ‘Trefone’ and Classes of Couplet Cell Interactions”. 25. Note 1 “Extended Trefone Couplets”. Note 2 “Singlet Cells”. 26. “Background of Cancer Research”. Click here for file
