# Supplementary material for: A model to explain specific cellular communications and cellular harmony:- a hypothesis of coupled cells and interactive coupling molecules
Source: Theor Biol Med Model. 2014 Sep 14;11:40. doi: 10.1186/1742-4682-11-40 (PMC4237941; doi:10.1186/1742-4682-11-40)
Supplement: Additional file 1 — 1. “Terminology”. 2. “Change in Cellular Characteristics”. 3. Note 1 “The Roles of SCD versus ACD”. Note 2. “Categories of SCD and ACD”. Note 3 “Further Examples of these AsCD Categories”. Note 4 “Change of differentiation type.”. 4. “The Effects of Growth Factors and other Bioactive Molecules 5. Note 1, “Additional Information on the Definition of a Trefone”. Note 2 “Clarification on Ligand and Binding Protein Interaction”. Note 3 “Cell Couplets – AsCD and the a-Cell and i-Cell”. Note 4 “Drosophila – Potential Trefones and ACD”. 6. “Calculated Levels of Trefones and TCC, with Cellular Actions”. 7. “Receptors for the Six IGFBPs and Cellular Effects”. 8. Note 1 “Extracellular Binding Proteins/ Soluble Receptors”. Note 2 “Cell Receptors for Binding Proteins/Soluble Receptors”. 9. Note 1. “Further Evidence for a Receptor for TCC”. Note 2 “Problems with Cross-linking Experiments”. 10. “Evidence for Binary Receptors aside from the IGF System”. 11. “Evidence for Two Receptors for a Particular Trefone, outside the IGF System”. 12. Note 1 “Mechanisms of Nuclear Localization”. Note 2 “NL of IGFBPs”. Note 3 “NL of Potential Trefones other than the IGF System”. 13. “Evidence Supporting the Existence of a-Cells and i-Cells”. 14. “Candidates with mainly a-Cell-Type or i-Cell-Type Characteristics”. 15. “Interacting Cells and Trefones not associated with the IGF System”. 16. “Heterogeneity/Variability of Cells in Culture.” 17. “Cell lines are not Typical of Normal Cells”. 18. “Cell Receptors and Cell Markers”. 19. “Defined Couplet Cells for Insulin and Glucagon; Gastrin and Histamine”. 20. “Potential Couplet Trefones”. 21. “Further Examples of Potential Trefone and Cell Couplets”. 22. “Examples of Cellular Regulation by Complexes”. 23. “Proteolytic enzymes and their inhibitors”. 24. “Expanded Definition of ‘Trefone’ and Classes of Couplet Cell Interactions”. 25. Note 1 “Extended Trefone Couplets”. Note 2 “Singlet Cells”. 26. “Background of Cancer Research”. [file 1742-4682-11-40-S1.zip › 3484671921331230_add1/3484671921331230_add01.doc]

**Additional File *One*:-**

**(b) Reception and interpretation of chemical messages will give intelligence to cells.**

***Terminology***

*The purpose of this File is to define the terminology used in this model, with some examples quoted to help explain and justify why the final definition has been chosen. The definitions focus on whether changes occur within or without a requirement for concomitant cell division. This is particularly important for the term “Differentiation”. This File also displays the wide diversity of cell options especially those associated with cell division.*

**Cell Division** here refers to the [process](http://en.wikipedia.org/wiki/Cell_division) by which a parent [cell](http://en.wikipedia.org/wiki/Cell_(biology)) divides into two or more offspring cells. A cell is defined as either a [membrane](http://www.biology-online.org/dictionary/Membrane)-bound 3-dimensional [structure](http://www.biology-online.org/dictionary/Structure) containing [biomolecules](http://www.biology-online.org/dictionary/Biomolecules) (proteins etc), which is involved in metabolic reactions to support its own life and/or that of a [multicellular](http://www.biology-online.org/dictionary/Multicellular) [organism](http://www.biology-online.org/dictionary/Organism) of which it is a part; or, more usually, a unit of life similarly involved but which is also self-replicating. Examples of the former include, in mammals, a proplatelet without a nucleus dividing to produce platelet cells and a red blood cell or erythrocyte without a nucleus, ribosomes or mitochondria. This latter cell is formed, via a reticulocyte which with no nucleus, from an erythroblast by an asymmetric division of a cell with the other offspring, a pyrenocyte, containing the nucleus**.**

**Stem Cell.** While self-renewal and multipotency are usually taken as the defining characteristics of stem cells**,** the definition of a stem cell is not fully established. A functional definition could be that “a stem cell is identified by its ability to provide differentiated cells or progenitors of differentiated cells necessary to the development and/or maintenance of a biological system”. A more exact definition of a stem cell is not required for this present model.

**Proliferation** here refers to the increase in the number of functional cells by cell division (CD) - symmetrical (SCD) or asymmetrical (AsCD).

**Differentiation** can be defined in many different ways. It may be defined as a process whereby a precursor cell in a particular proteomic state is converted to a cell which is different in clearly defined functional properties. In the development of a multicellular organism and in the maintenance of life, a cell, at a particular point in time, is on a journey to, or is at, a predetermined, single, “final” fate which will contribute a specific functional property to the whole organism. This differentiation journey may include several differentiation stages - a stem cell state, various intermediate states {referred to as intermediate progenies, progenitor cells or as a transit (amplifying) population and single-fated cells (terminally differentiated cells). Differentiation also indicates a cellular change of state to a more specialized and more dependent entity and can be defined also in terms of changes induced in response to intercellular chemical messages. It can be defined in two parts. Firstly, there is an essential genetic change(s) associated with a cell division (CD) causing the exposure, concealment or chemical alteration of genes leading to the potential gain of expression of new characteristics and/or to the loss of parental characteristics, in the progenic cell. Some of the transcribed euchromatin would become unexpressed heterochromatin in eukaryocytes and vice versa. The procession through the cell cycle would be activated by inducing signal(s) in the extracellular environment. Soluble signals could include a Competence Factor (eg. EGF - Epidermal Growth Factor) and a Progression Factor (eg. IGF-I – Insulin-Like Growth Factor-I)**,** both of which will determine the transcription of genes for a cell cycle. Secondly, associated with the genetic changes caused by cell division, the “primed” offspring cell(s) has(ve) potential to change further, but these changes will only be observable or detectable changes if there is exposure to the appropriate extracellular inductive, differentiation factor(s){DF(s)}. These DFs may be the same as caused/permitted the genetic reorganization associated with the prior cell division, so that in this situation there would be a seamless transition to the differentiated state. If different, these post-CD, differentiative changes may be variably dependent on the properties and concentration of the DF(s) which would stabilize or select between the phenotypic options inherited by the cell. In other words, the offspring cells may have constrained/limited options or may still be somewhat plastic in life choices, but in either case, would have fewer options than the parent cell.

Similarly, others have suggested that two levels of control of gene activity can be defined; (1) a Yes/No switch as to whether the gene is accessible for activation or not, and (2) if Yes to (1), a “Zero to High” gene-activity dial, dependent on inducer concentration. As examples, hematopoietic stem cells respond to high or low concentrations of c-Kit receptor to form either myeloid or and lymphoid development respectively**;** the differentiation of human myeloblastoid leukemia HL60 cells induced by 1,25-dihydroxyvitamin D could be divided into at least two distinct phases, firstly proliferation then differentiation. Once a cell is in a lineage, it has “cellular memory” – whether a gene is “on” or “off” is inherited to ensure that the defined gene expression and cell phenotype are maintained epigenetically over future generations by SCD, until stimulated otherwise**.**

Differentiation will then involve changes in genetic expression - a Chromosomal Organisational Upheaval (a COU), and cell division is required to “untie, change and retie” the DNA strands so that only the DNA required in the new environment is accessible or activable. Lineage-specific genes, activated or repressed by lineage-defining transcription factors, would regulate the COU and the subsequent differentiation changes, but perhaps not as simplistic as a genetic cassette change. Although the cells have a more limited and more defined repertoire of genes which are required in a phase-specific parts of the cell cycle, certainly many differentiated cells can still divide.

Full differentiation would be a gradual process requiring a pre-programmed, intricately-orchestrated sequence of cell divisions (CDs) before the full transformation is completed. Up to five CDs may be required for erythroid progenitors before the final formation of reticulocytes. Perhaps maturation of the intermediate cells occurs along the lineage before the next designated CD can occur.

On the other hand, it may be the place not the number of rounds of CD that determines the timing of differentiation. When corneal epithelial cells divide in the basal layer, they do so for a variable number of rounds of CD before they detach from the basal lamina to enter the wing-cell layer and then differentiate.

For a cell to be given a specific task in the body corporate, then the cell needs to be protected such that it doesn’t digress to other functions at the expense of the whole. The simplest approach would be to lock up the cell’s not-to-be-used DNA such that it cannot be accessed by the now-dedicated cell. This would be easiest done during cell division when the DNA would be more readily accessible, and only undone again with a cell division cycle. In the presence of a Differentiation Factor, then a prior cell division is required to release the differentiation program to commence after the division.

In a life-plan for a multicellular organism, a stem or progenitor cell, which is destined to its predetermined “final” state having a set of defined functions (a z-Cell - in a state which is normally not reversed), will transit various stages to the y-Cell (the penultimate cell) which will produce the z-Cell. This transit will only occur if the precursor cells are in the presence of the specific DFs. The y-Cell could form the final, matured (terminal) state in the presence of a DF either within the final cell division or following “maturation” of the cell programmed by the previous CD. This “final” fate will be transient until the cell no longer exists due to phagocytosis, apoptosis or necrosis, or the cell could change its proteomic state from the predetermined “final” fate, by fusion with another cell (some sort of complementary cell), by dedifferentiation, by transdifferentiation or redifferentiation. (See later for definitions of these.) As an exception, the “final” life state of a cell within an organism could be less transient and perhaps more perpetual, such as for a germ cell.

Differentiation, defined as a change in the characteristics of a cell, is usually associated with a change in the proteins which are within the cell membrane and exposed to the extracellular environment. New proteins will be present with/without the loss of old ones and will include signalling receptors or fixed ligands for communications with close and/or distant cells, along with adhesion proteins for specific cellular interactions. For signalling molecules, a normal differentiation would produce a lineage commitment as defined by the expression of new receptors which are specific for signalling molecules from that lineage. An abnormal differentiation could produce a cell with receptors that respond to signalling molecules from another lineage and these cells could flourish as a cancer. For adhesion molecules, their types would determine the position of the cell in the organism, just as injected bone-marrow stem cells seek and find the marrow from the blood. An abnormal differentiation could produce a cell with a change in adhesion proteins with local contact adhesion lost and such cells could flourish as a cancer in another part of the body. Differentiation may change the specialisation of just one or both cells formed after an asymmetric or symmetric cell division with no change in any self-renewal cell.

A growth factor may also be a DF. For example, IGF-I and brain-derived neurotrophic factor are DFs for postmitotic CNS stem cell-derived neuronal precursors. Another example is seen in the formation of a uterine-vulval connection in *Caenorhabditis elegans*. A growth factor, LIN-3, normally derived from an anchor cell, interacts with a receptor (LET-23) and induces three of the six vulval precursor cell to adopt vulval fates. A list of DFs would also include TGF-β, Retinoic acid, Vitamin D, Angiotensin II, Nerve Growth Factor, Connective Tissue Growth Factor and Thyroxine

Note that some DFs can be antiproliferative and that differentiation can be caused by deprivation of a growth factor eg. of FGF in the differentiation of myoblasts.

It is notable that, in studies on the process of normal differentiation, the possibility of a preceding or accompanying AsCD is often ignored.

**Transformation (TF)** is defined here as an obvious change in outward form, appearance or property. It is similar to extreme differentiation but the latter is a more gradual process involving progressive changes. TF is a major event with easily observable, physical changes and new attributes again associated with a cell that has acquired new receptors and/or lost old ones, noticeable immediately after cell division or subsequently caused by the presence of environment of messages/ligands interacting with the cell’s receptors. There may not be any concomitant cell division but in multicellular organisms, it occurs in the offspring cell(s) only after a permissive division of the parent cell followed by the equivalent to the second stage of differentiation. In unicellular bacteria, the transformation occurs in response to the environmental changes eg. in stressful conditions, the flagella of *Chlamydomonas* unicells may be lost or resorbed in preparation for cell division**.** Again, a differentiation-like factor would be required for transformation and may be a quorum-sensing molecule or a bacterial peptide pheromone. While some bacteria have two vegetative cell types, others can transform to polymorphic vegetative cell types. After AsCD of *Caulobacter crescentus*, one offspring cell, the swarmer cell, can transform into the other type of offspring cell, the stalk cell, and then recommence another AsCD.

Controlled proheterocyst transformation occurs in the blue-green alga *Anabaena*. Following AsCD in these algae growing in nitrogen-free medium, only the smaller offspring cell may transform to a heterocyst (a specialised cell formed at regular intervals within the filamentous vegetative cells). But not all such cells do so; all are candidates and several in a local area may become a preheterocyst but only one of these become a heterocyst in that area; the rest regress. In this case, before the transformation event, there is a prerequisite genetic predisposition produced in the small cell by the AsCD, which, in the absence of high levels of two inhibitors in the environment, produces the heterocyst via the preheterocyst.

A mutant enzyme may cause transformation. Expression of a mutant isocitrate dehydrogenase 1 in cells of a human erythroleukemia cell line (TF-1) produces leukemic transformation indicated by the new properties of growth factor independence and impaired differentiation. A specific isomer of 2-hydroxyglutarate [(R)-2HG] also causes this transformation.

**Dedifferentiation (DD)** refers to moving backwards along its previous life line, by cell division (CD), to be controlled again by the previous signalling molecules (hormone/growth factors/cytokines). It is defined here as the reversion of a specialized, partially differentiated cell to a more complex, unspecialized form, of an earlier developmental stage or lineage of that cell and caused by a reprogramming of expressed genes.

Note that a change back to an embryonic state is not a change to a simpler or primitive state; rather it is to a more complex and independent state eg. to a cell with stemness^,^. DD of spores can be viewed as a regulated and orderly retreat, which, in turn, may be reversed (redifferentiation). DD is often part of a regenerative [mechanism](http://www.biology-online.org/dictionary/Mechanism) in lower life forms such as [worms](http://www.biology-online.org/dictionary/Worm) and [amphibians](http://www.biology-online.org/dictionary/Amphibian). DD of mouse embryonic or adult fibroblasts can be induced by introducing four defined factors, Oct3/4, Sox2, c-Myc, and Klf4 to produce pluripotent stem cells. Differentiation may be reversible over a number of passages. A subpopulation of cells of a colon carcinoma cell line (HT-29) will grow in the absence of glucose and, after confluence, exhibit enterocytic differentiation. But, with glucose added to the differentiated cells, after seven passages, the cells revert to their undifferentiated state. Germline stem cells can also arise from reversion of spermatogonia in *Drosophila* and in mice. Controlled proheterocyst DD occurs in the blue-green alga *Anabaena*. As described above, all but one of the preheterocysts regress. Some reversal of cellular characteristics could occur without CD but this is not included here as DD.

**Retrodifferentiation** has a definition that it “.... characterizes a biological phenomenon of completely reversible differentiation events, whereby all morphological and functional properties of the retrodifferentiated and the previously more undifferentiated cells are indistinguishable”. This would be equivalent to DD but without CD. This term is not used again in this article.

**Redifferentiation (RD)** refers to the changing of state of a cell with cell division, to a previous differentiated state and retaining some of its signalling molecules, and is defined as the process of differentiation in a cell type that had undergone dedifferentiation. In the kidney, specific subpopulations of apparently differentiated cells can de-differentiate in response to stress, and re-differentiate when the crisis passes.

**Transdifferentiation (TrD)** refers to moving across to another lineage normally by CD, to be controlled by new signalling molecules. It is defined here as the switching of a differentiated cell from one lineage into another differentiated cell type of a different lineage. This “crossover” may be direct TrD involving no intermediate progenitor cell type or any other pluripotent state. As such, it occurs in vitro by transfection of cells with transcription factors eg. fibroblasts, forced to express MyoD and thus converted to myoblasts by TrD; fibroblasts also produced “induced pluripotent stem cells” by this “lineage reprogramming”. Natural TrD is not direct but rather involves three steps- redifferentiation involving regressing to a point where cells can then switch lineages by cell division, then differentiating within the new lineage. Again, cell division is required for the COU, as observed in the postnatal mouse cochlea where support cells surrounding hair cells, retain the ability to divide and transdifferentiate into new hair cells, in culture. The TrD of the multipotent epithelium of a budding tunicate requires at least one round of cell division.

Perhaps for the second step, the actual reversible crossover can be induced by the addition or withdrawal of a growth factor or of a cAMP analog as described for cultivated Schwann cells. The addition of dexamethasone to pluripotent cells (derived from a pancreatic tumour) cause a TD to hepatic-like cells over a period of three weeks. The TrD of mesenchymal stem cells from bone marrow into bone, muscle and other type cells illustrates the plasticity of stem cells .

This TD crossover could be referred to as a transdetermination, defined as a switch in [lineage](http://www.ncbi.nlm.nih.gov/pmc/articles/PMC2835174/) commitment in a stem or progenitor cell, although as here, not necessarily to a closely related cell type. TD is then a type of [metaplasia](http://en.wikipedia.org/wiki/Metaplasia), which is a more generic term to include all cell-fate switches, including the interconversion of stem cells^,^ .

**Reversible Transdifferentiation (RTD)** is a natural occurrence wherein mammalian breast adipocytes convert to secretory epithelial cells in pregnancy and revert after lactation. Both conversion of pancreatic-type cells to hepatic-type cells and the reverse have been documented and the interconversion of somatotrophs and lactotrophs in pituitary hyperplasia is another example.

**Terminal Differentiation (TD)** is here defined as the state of permanent cessation of cell division. If the whole chromosome set is retained in a cell, then theoretically cell division is still possible if the right gene are turned on or off, so that TD may be difficult to establish as a permanent end-point except as being qualified as TD in a certain environment. On the other hand, corneocytes (functional cell corpses derived from keratinocytes, lens fiber cells and erythrocytes, all of which have lost their nuclei, have definitely undergone TD. Also, multinucleated muscle fibres formed from fused myoblasyts are considered to be irretrievably differentiated; deprivation of a growth factor can cause differentiation and then this fusion event.

These definitions assume that cell division is necessary for selective reversal of the silencing of genes within the facultative heterochromatin and/or for selective silencing of previously activated/transcribed genes within the euchromatin. These occur by appropriate acylation, phosphorylation, sumoylation or ubiquitination of histones or by methylation of CpG sites of DNA or by reversal of such previous chemical modifications. Also, gene silencing or activation may be modified by position within the chromosomal packaging, position-effect variegation or by neighbourhood effects or by non-coding RNA but all of these would be controlled and programmed for the offspring cells by cell division. In addition, proteins such as the retinoblastoma protein, affect silencing by regulation of pericentric heterochromatin. Do proliferating bacteria differentiate (or perhaps it is a dedifferentiation) to spores when starved of nutrients? Starvation induces *Bacillus subtilis* to form spores by AsCD with (de)differentiation, associated with the activation of the master transcription regulator, Spo0A, which directly regulates the transcription of 121 genes, with about one-third of these being activated and the remainder repressed. As an alternative to sporulation requiring a prior AsCD to produce the spore precursor plus a helper cell (with death), SCD with differentiation could occur to produce two spore precursors.

##  **Adaptation** is defined as a change in the metabolism of a cell associated with altered genetic expression to cope with a new level of incoming nutrients (or other messages) from the environment; this usually involves a quantitative change in the production of specific proteins, including receptors. This may involve the up- or down-regulation of hundreds of proteins as is observed when *E. coli* is changed from laboratory media to whole fresh bovine milk.

The distinguishing feature between adaptation and differentiation in that the latter requires a prior CD to make the progeny permissive to a DF while the former activity is more “inbuilt” plasticity.

## **Maturation** is similar to adaptation in that there is also a change in the metabolism of a cell associated with altered genetic expression to cope with a new level of incoming nutrients (or messages) from the environment. However, maturation implies a pre-programmed change that occurs if certain conditions apply where adaptation is more a generic skill to cope with more random changes in the environment. Maturation, in an extreme, is used to refer to the intracellular losses of nucleus, mitochondria and ribosomes that occur as an erythroblast changes to a reticulocyte and then to an erythrocyte.

**Phenotype switching,** where a cell switches reversibly between two “types” of cell**,** is assumed here to occur without cell division. In switching between white and opaque types of *Candida albicans*, hundreds of genes change expression levels by over 2-fold. Equally, resource polyphenism, another example of phenotypic plasticity where a single genome produces alternative phenotypes in response to specific environmental changes, could belong to this same category, but, in addition, may require cell division.

**Quiescence** is defined as a temporary arrest of proliferation.

**Information-Carrying Molecules and Inter-Cellular Messengers (ICMs).**

Information may be transferred by growth factors, cytokines, adipokines, chemokines, hormones and other bioactive substances. These, as a group, are referred to herein as generic *Information-Carrying Molecules and Inter-Cellular Messengers* (ICMs).

A growth factor, a differentiation factor or a neurotransmitter would be an ICM. An ICM may cause a change in cellular form or activity with or without cell division to produce differentiation, DD, RD, TrD, RTD, TD, TF or other related cellular change. A particular substance may be an ICM for one cell type but not for another cell type.

**Qualification.** Every definition in this section is debatable, even controversial, but the definitions are those implied where used in the current text.

**REFERENCES:-**

1.	Thon JN, Montalvo A, Patel-Hett S, Devine MT, Richardson JL, Ehrlicher A, Larson MK, Hoffmeister K, Hartwig JH, Italiano JE, Jr.: **Cytoskeletal mechanics of proplatelet maturation and platelet release**. *J Cell Biol* 2010, **191**(4):861-874.

2.	McGrath KE, Kingsley PD, Koniski AD, Porter RL, Bushnell TP, Palis J: **Enucleation of primitive erythroid cells generates a transient population of "pyrenocytes" in the mammalian fetus**. *Blood* 2008, **111**(4):2409-2417.

3.	Potten CS, Loeffler M: **Stem cells: attributes, cycles, spirals, pitfalls and uncertainties. Lessons for and from the crypt**. *Development* 1990, **110**(4):1001-1020.

4.	Parker GC, Anastassova-Kristeva M, Eisenberg LM, Rao MS, Williams MA, Sanberg PR, English D: **Stem cells: shibboleths of development, part II: Toward a functional definition**. *Stem Cells Dev* 2005, **14**(5):463-469.

5.	Duncan MD, Korman LY, Bass BL: **Epidermal growth factor primes intestinal epithelial cells for proliferative effect of insulin-like growth factor I**. *Dig Dis Sci* 1994, **39**(10):2197-2201.

6.	Loeffler M, Roeder I: **Tissue stem cells: definition, plasticity, heterogeneity, self-organization and models--a conceptual approach**. *Cells Tissues Organs* 2002, **171**(1):8-26.

7.	Okuyama R, Koguma M, Yanai N, Obinata M: **Bone marrow stromal cells induce myeloid and lymphoid development of the sorted hematopoietic stem cells in vitro**. *Blood* 1995, **86**(7):2590-2597.

8.	Wang X, Studzinski GP: **Activation of extracellular signal-regulated kinases (ERKs) defines the first phase of 1,25-dihydroxyvitamin D3-induced differentiation of HL60 cells**. *J Cell Biochem* 2001, **80**(4):471-482.

9.	Mahmoudi T, Verrijzer CP: **Chromatin silencing and activation by Polycomb and trithorax group proteins**. *Oncogene* 2001, **20**(24):3055-3066.

10.	Miller SA, Weinmann AS: **Molecular mechanisms by which T-bet regulates T-helper cell commitment**. *Immunol Rev* 2010, **238**(1):233-246.

11.	Dalgaard JZ, Vengrova S: **Selective gene expression in multigene families from yeast to mammals**. *Sci STKE* 2004, **2004**(256):re17.

12.	Ge WP, Zhou W, Luo Q, Jan LY, Jan YN: **Dividing glial cells maintain differentiated properties including complex morphology and functional synapses**. *Proc Natl Acad Sci U S A* 2009, **106**(1):328-333.

13.	Dolznig H, Bartunek P, Nasmyth K, Mullner EW, Beug H: **Terminal differentiation of normal chicken erythroid progenitors: shortening of G1 correlates with loss of D-cyclin/cdk4 expression and altered cell size control**. *Cell Growth Differ* 1995, **6**(11):1341-1352.

14.	Beebe DC, Masters BR: **Cell lineage and the differentiation of corneal epithelial cells**. *Invest Ophthalmol Vis Sci* 1996, **37**(9):1815-1825.

15.	Cremisi F, Philpott A, Ohnuma S: **Cell cycle and cell fate interactions in neural development**. *Curr Opin Neurobiol* 2003, **13**(1):26-33.

16.	Arsenijevic Y, Weiss S: **Insulin-like growth factor-I is a differentiation factor for postmitotic CNS stem cell-derived neuronal precursors: distinct actions from those of brain-derived neurotrophic factor**. *J Neurosci* 1998, **18**(6):2118-2128.

17.	Newman AP, Sternberg PW: **Coordinated morphogenesis of epithelia during development of the Caenorhabditis elegans uterine-vulval connection**. *Proc Natl Acad Sci U S A* 1996, **93**(18):9329-9333.

18.	Clegg CH, Linkhart TA, Olwin BB, Hauschka SD: **Growth factor control of skeletal muscle differentiation: commitment to terminal differentiation occurs in G1 phase and is repressed by fibroblast growth factor**. *J Cell Biol* 1987, **105**(2):949-956.

19.	Quarmby LM: **Cellular deflagellation**. *Int Rev Cytol* 2004, **233**:47-91.

20.	Johnson UG, Porter KR: **Fine structure of cell division in Chlamydomonas reinhardi. Basal bodies and microtubules**. *J Cell Biol* 1968, **38**(2):403-425.

21.	Morrison DA: **Streptococcal competence for genetic transformation: regulation by peptide pheromones**. *Microb Drug Resist* 1997, **3**(1):27-37.

22.	Whittenbury R, Dow CS: **Morphogenesis and differentiation in Rhodomicrobium vannielii and other budding and prosthecate bacteria**. *Bacteriol Rev* 1977, **41**(3):754-808.

23.	Jenal U: **Signal transduction mechanisms in Caulobacter crescentus development and cell cycle control**. *FEMS Microbiology Reviews* 2000, **24**(2):177-191.

24.	Wilcox M, Mitchison GJ, Smith RJ: **Pattern formation in the blue-green alga, Anabaena. I. Basic mechanisms**. *J Cell Sci* 1973, **12**(3):707-723.

25.	Higa KC, Rajagopalan R, Risser DD, Rivers OS, Tom SK, Videau P, Callahan SM: **The RGSGR amino acid motif of the intercellular signalling protein, HetN, is required for patterning of heterocysts in Anabaena sp. strain PCC 7120**. *Mol Microbiol* 2012, **83**(4):682-693.

26.	Losman JA, Looper RE, Koivunen P, Lee S, Schneider RK, McMahon C, Cowley GS, Root DE, Ebert BL, Kaelin WG, Jr.: **(R)-2-hydroxyglutarate is sufficient to promote leukemogenesis and its effects are reversible**. *Science* 2013, **339**(6127):1621-1625.

27.	Wilcox M, Mitchison GJ, Smith RJ: **Pattern formation in the blue-green alga Anabaena. II. Controlled proheterocyst regression**. *J Cell Sci* 1973, **13**(3):637-649.

28.	Lander AD: **The 'stem cell' concept: is it holding us back?** *J Biol* 2009, **8**(8):70.

29.	Snippert HJ, van der Flier LG, Sato T, van Es JH, van den Born M, Kroon-Veenboer C, Barker N, Klein AM, van Rheenen J, Simons BD *et al*: **Intestinal crypt homeostasis results from neutral competition between symmetrically dividing Lgr5 stem cells**. *Cell* 2010, **143**(1):134-144.

30.	Katoh M, Shaw C, Xu Q, Van Driessche N, Morio T, Kuwayama H, Obara S, Urushihara H, Tanaka Y, Shaulsky G: **An orderly retreat: Dedifferentiation is a regulated process**. *Proc Natl Acad Sci U S A* 2004, **101**(18):7005-7010.

31.	Takahashi K, Yamanaka S: **Induction of pluripotent stem cells from mouse embryonic and adult fibroblast cultures by defined factors**. *Cell* 2006, **126**(4):663-676.

32.	Zweibaum A, Pinto M, Chevalier G, Dussaulx E, Triadou N, Lacroix B, Haffen K, Brun JL, Rousset M: **Enterocytic differentiation of a subpopulation of the human colon tumor cell line HT-29 selected for growth in sugar-free medium and its inhibition by glucose**. *J Cell Physiol* 1985, **122**(1):21-29.

33.	Nakagawa T, Sharma M, Nabeshima Y, Braun RE, Yoshida S: **Functional hierarchy and reversibility within the murine spermatogenic stem cell compartment**. *Science* 2010, **328**(5974):62-67.

34.	Hass R: **Rejuvenation in distinct cell populations - What does it mean?** *Exp Gerontol* 2009, **44**(10):634-638.

35.	Sequeira Lopez ML, Pentz ES, Nomasa T, Smithies O, Gomez RA: **Renin Cells Are Precursors for Multiple Cell Types that Switch to the Renin Phenotype When Homeostasis Is Threatened**. *Dev Cell* 2004, **6**(5):719-728.

36.	Xie H, Ye M, Feng R, Graf T: **Stepwise reprogramming of B cells into macrophages**. *Cell* 2004, **117**(5):663-676.

37.	Davis RL, Weintraub H, Lassar AB: **Expression of a single transfected cDNA converts fibroblasts to myoblasts**. *Cell* 1987, **51**(6):987-1000.

38.	Graf T, Enver T: **Forcing cells to change lineages**. *Nature* 2009, **462**(7273):587-594.

39.	Jopling C, Boue S, Izpisua Belmonte JC: **Dedifferentiation, transdifferentiation and reprogramming: three routes to regeneration**. *Nat Rev Mol Cell Biol* 2011, **12**(2):79-89.

40.	White PM, Doetzlhofer A, Lee YS, Groves AK, Segil N: **Mammalian cochlear supporting cells can divide and trans-differentiate into hair cells**. *Nature* 2006, **441**(7096):984-987.

41.	Kawamura K, Fujiwara S: **Cellular and molecular characterization of transdifferentiation in the process of morphallaxis of budding tunicates**. *Semin Cell Biol* 1995, **6**(3):117-126.

42.	Monje PV, Soto J, Bacallao K, Wood PM: **Schwann cell dedifferentiation is independent of mitogenic signaling and uncoupled to proliferation: role of cAMP and JNK in the maintenance of the differentiated state**. *J Biol Chem* 2010, **285**(40):31024-31036.

43.	Shen CN, Slack JM, Tosh D: **Molecular basis of transdifferentiation of pancreas to liver**. *Nat Cell Biol* 2000, **2**(12):879-887.

44.	Krabbe C, Zimmer J, Meyer M: **Neural transdifferentiation of mesenchymal stem cells--a critical review**. *APMIS* 2005, **113**(11-12):831-844.

45.	Manohar R, Lagasse E: **Transdetermination: a new trend in cellular reprogramming**. *Mol Ther* 2009, **17**(6):936-938.

46.	Tosh D, Slack JM: **How cells change their phenotype**. *Nat Rev Mol Cell Biol* 2002, **3**(3):187-194.

47.	Li WC, Yu WY, Quinlan JM, Burke ZD, Tosh D: **The molecular basis of transdifferentiation**. *J Cell Mol Med* 2005, **9**(3):569-582.

48.	Morroni M, Giordano A, Zingaretti MC, Boiani R, De Matteis R, Kahn BB, Nisoli E, Tonello C, Pisoschi C, Luchetti MM *et al*: **Reversible transdifferentiation of secretory epithelial cells into adipocytes in the mammary gland**. *Proc Natl Acad Sci U S A* 2004, **101**(48):16801-16806.

49.	Shen CN, Burke ZD, Tosh D: **Transdifferentiation, metaplasia and tissue regeneration**. *Organogenesis* 2004, **1**(2):36-44.

50.	Vidal S, Horvath E, Kovacs K, Lloyd RV, Smyth HS: **Reversible transdifferentiation: interconversion of somatotrophs and lactotrophs in pituitary hyperplasia**. *Mod Pathol* 2001, **14**(1):20-28.

51.	Lippens S, Denecker G, Ovaere P, Vandenabeele P, Declercq W: **Death penalty for keratinocytes: apoptosis versus cornification**. *Cell Death Differ* 2005, **12 Suppl 2**:1497-1508.

52.	Counis MF, Chaudun E, Arruti C, Oliver L, Sanwal M, Courtois Y, Torriglia A: **Analysis of nuclear degradation during lens cell differentiation**. *Cell Death Differ* 1998, **5**(4):251-261.

53.	Shen F, Kirmani KZ, Xiao Z, Thirlby BH, Hickey RJ, Malkas LH: **Nuclear protein isoforms: implications for cancer diagnosis and therapy**. *J Cell Biochem* 2011, **112**(3):756-760.

54.	Koch CM, Joussen S, Schellenberg A, Lin Q, Zenke M, Wagner W: **Monitoring of cellular senescence by DNA-methylation at specific CpG sites**. *Aging Cell* 2012, **11**(2):366-369.

55.	Fodor BD, Shukeir N, Reuter G, Jenuwein T: **Mammalian Su(var) genes in chromatin control**. *Annu Rev Cell Dev Biol* 2010, **26**:471-501.

56.	West AG, Fraser P: **Remote control of gene transcription**. *Hum Mol Genet* 2005, **14 Spec No 1**:R101-111.

57.	Mattick JS: **The genetic signatures of noncoding RNAs**. *PLoS Genet* 2009, **5**(4):e1000459.

58.	Isaac CE, Francis SM, Martens AL, Julian LM, Seifried LA, Erdmann N, Binne UK, Harrington L, Sicinski P, Berube NG *et al*: **The retinoblastoma protein regulates pericentric heterochromatin**. *Mol Cell Biol* 2006, **26**(9):3659-3671.

59.	Piggot PJ, Hilbert DW: **Sporulation of Bacillus subtilis**. *Curr Opin Microbiol* 2004, **7**(6):579-586.

60.	Lippolis JD, Bayles DO, Reinhardt TA: **Proteomic changes in Escherichia coli when grown in fresh milk versus laboratory media**. *J Proteome Res* 2009, **8**(1):149-158.

61.	Lan CY, Newport G, Murillo LA, Jones T, Scherer S, Davis RW, Agabian N: **Metabolic specialization associated with phenotypic switching in Candidaalbicans**. *Proc Natl Acad Sci U S A* 2002, **99**(23):14907-14912.
