# Supplementary material for: A model to explain specific cellular communications and cellular harmony:- a hypothesis of coupled cells and interactive coupling molecules
Source: Theor Biol Med Model. 2014 Sep 14;11:40. doi: 10.1186/1742-4682-11-40 (PMC4237941; doi:10.1186/1742-4682-11-40)
Supplement: Additional file 1 — 1. “Terminology”. 2. “Change in Cellular Characteristics”. 3. Note 1 “The Roles of SCD versus ACD”. Note 2. “Categories of SCD and ACD”. Note 3 “Further Examples of these AsCD Categories”. Note 4 “Change of differentiation type.”. 4. “The Effects of Growth Factors and other Bioactive Molecules 5. Note 1, “Additional Information on the Definition of a Trefone”. Note 2 “Clarification on Ligand and Binding Protein Interaction”. Note 3 “Cell Couplets – AsCD and the a-Cell and i-Cell”. Note 4 “Drosophila – Potential Trefones and ACD”. 6. “Calculated Levels of Trefones and TCC, with Cellular Actions”. 7. “Receptors for the Six IGFBPs and Cellular Effects”. 8. Note 1 “Extracellular Binding Proteins/ Soluble Receptors”. Note 2 “Cell Receptors for Binding Proteins/Soluble Receptors”. 9. Note 1. “Further Evidence for a Receptor for TCC”. Note 2 “Problems with Cross-linking Experiments”. 10. “Evidence for Binary Receptors aside from the IGF System”. 11. “Evidence for Two Receptors for a Particular Trefone, outside the IGF System”. 12. Note 1 “Mechanisms of Nuclear Localization”. Note 2 “NL of IGFBPs”. Note 3 “NL of Potential Trefones other than the IGF System”. 13. “Evidence Supporting the Existence of a-Cells and i-Cells”. 14. “Candidates with mainly a-Cell-Type or i-Cell-Type Characteristics”. 15. “Interacting Cells and Trefones not associated with the IGF System”. 16. “Heterogeneity/Variability of Cells in Culture.” 17. “Cell lines are not Typical of Normal Cells”. 18. “Cell Receptors and Cell Markers”. 19. “Defined Couplet Cells for Insulin and Glucagon; Gastrin and Histamine”. 20. “Potential Couplet Trefones”. 21. “Further Examples of Potential Trefone and Cell Couplets”. 22. “Examples of Cellular Regulation by Complexes”. 23. “Proteolytic enzymes and their inhibitors”. 24. “Expanded Definition of ‘Trefone’ and Classes of Couplet Cell Interactions”. 25. Note 1 “Extended Trefone Couplets”. Note 2 “Singlet Cells”. 26. “Background of Cancer Research”. [file 1742-4682-11-40-S1.zip › 3484671921331230_add1/3484671921331230_add02.doc]

**Additional File *Two*:-**

**(a) Asymmetric cell division, with division of labour, is likely a part of this development.**

***Change in Cellular Characteristics with or without Differentiation (with Cell Division), with Division of Labour*.**

*The purpose of this File is to provide evidence for the transition of unicellular life to multicellular by one cell’s adaptation to the environment being supported by another cell of the same type. This evolutionary division of metabolic labour was formalized in the more modern asymmetric cell division (AsCD).*

Some bacteria form biofilms which represent a step in the transition from a single cell to a community of interactive cells – a significant rung in the ladder towards fully multicellular organisms. Biofilms and multicellularity give social advantages:- colonization of nutrient-rich areas, direct exchange of nutrients, protection from or resistance to environmental stresses, to biocides and predation. These community activities are generated by genetic diversity and may be strengthened by the presence of specialized subpopulations spawned during biofilm growth of a clonal population eg. in *Pseudomonas aeruginosa*. Aside from cellular adaptation and transformation, asymmetric [cell](http://www.ncbi.nlm.nih.gov/pubmed?term=biofilm asymmetric cell division) division facilitates biofilm formation eg. in *Caulobacter crescentus*, where one offspring cell is a motile swarmer cell but [the other](http://www.ncbi.nlm.nih.gov/pubmed?term=biofilm asymmetric cell division) remains as a stalked cell stuck to the surface substrate forming the biofilm. Of note is the observation that biofilms can form into sophisticated architectures with individual cells taking on specific tasks.

Cellular diversity allows survival. Even in single-species of ancient *Archaea*, biofilm is found to contain cells that are physiologically and possibly genetically differentiated with respect to each other. Possible dual syntrophic reactions between methanogenic and methanotrophic cell types would be further evidence of the genetic versatility and cooperativity needed in multicellular development.

An unstructured population of identical bacterial cells can form spatially organized, multicellular structures by rearranging into a “fruiting body” in response to starvation. For example, cells of the bacterium *Myxococcus xanthus* respond to starvation by using multiple intercellular signals to construct these fruiting bodies within which a minority of cells (10-20%) dedifferentiate into stress-resistant dormant myxospores as a survival strategy. Such cooperative social behaviour requires both temporal and spatial differentiation and coordination in three dimensions, controlled by intercellular interaction either by contact signalling or by soluble messages. These intercellular signals control [the expression](http://www.ncbi.nlm.nih.gov/pmc/articles/PMC94986/?tool=pubmed) of developmentally regulated genes via a complex signal transduction system. This includes the two-component systems (with sensor histidine kinases and response regulators) which are important for normal motility and fruiting body formation and sporulation. However, these spores of *M. xanthus* are produced by remodeling of the original rod-shaped cell into a sphere with thick cell walls - dedifferentiation in the absence of cell division, in a 1-3-day starvation-induced multicellular developmental program. *Bacillus subtilis* is different. While it also forms endospores in response to starvation, they arise from an asymmetric cell division and this produces a prespore that becomes engulfed by, matured within, and then released from, the parent cell.

Early evolution into multicellularity may have been utilised by a single cell as a means of improving metabolic efficiency; many species of *Cyanobacteria* use photosynthesis and fix nitrogen. Because these two anabolic activities are difficult to combine in a single cell, some species can separate the two activities spatially by confining the nitrogen fixation to differentiated cells called heterocysts. These heterocysts occur singly, at regular intervals between two vegetative cells within a filament of cells, produced by an asymmetric cell division. Another example of this division of labour with AsCD is in the algae *Volvox carteri* wherein two distinct cell types are produced; small cells which become vegetative, biflagellate cells, and large cells which become reproductive gonidia. In contrast, the algae *Chlamydomonas reinhardtii* alternates between a biflagellate vegetative cell and, after flagella retraction, a reproductive cell which undergoes symmetric cell division (SCD). Another rationale for the evolution into multicellularity may have been for a selfish cell merely to propagate itself by dispersion, thereby generating a “life cycle” of change in an intellectual response to environmental challenge. A basic example is the slime mould *Dictyostelium discoideum,* which grows as a single-celled amoeba and achieves multicellularity by the aggregation of up to 10^5^ cells in response to nutrient depletion. Intercellular communication by secreted cyclic-AMP, along with a DIF **(**Differentiation Inducing Factor) or a “conditioned medium factor”, signals the cells to aggregate to a motile slug and form into a tiny fungus-like fruiting body with a spore ball on top of a stalk. Within the slug cell, differentiation has occurred with asymmetric divisions to produce seven new functional cells (eg. the prespore and prestalk cells which organise and form the fruiting body for spore release) plus null cells. Spore release provides new life by dispersion. In a more complex situation, early asymmetric division of cells of the embryo of the nematode *Caenorhabditis elegans* are critical, thereby producing the six "founder" cells which [account](http://www.ncbi.nlm.nih.gov/pubmed?term=Bowerman 1998 asymmetric) for much of the worm’s body plan.

Aside from stem cells, AsCD has also been extensively observed in the non-vertebrates eg. *C. elegans*, *Drosophila* and in other animals including mammalian embryo development. This occurs even from the first cell division where it is possible to identify the one cell which will contribute a greater proportion of its progeny to the abembryonic part of the blastocyst and the other cell which will contribute a greater proportion of its progeny to the embryonic part containing the inner cell mass. Another example is the mammalian primordial oocyte which undergoes asymmetric cell divisions to reach the mature oocyte by meioses with the formation of polar bodies – an extreme form of AsCD.

**REFERENCES:-**

1.	King N: **The unicellular ancestry of animal development**. *Dev Cell* 2004, **7**(3):313-325.

2.	Boles BR, Thoendel M, Singh PK: **Self-generated diversity produces "insurance effects" in biofilm communities**. *Proc Natl Acad Sci U S A* 2004, **101**(47):16630-16635.

3.	Kirkpatrick CL, Viollier PH: **Cell dispersal in biofilms: an extracellular DNA masks nature's strongest glue**. *Mol Microbiol* 2010, **77**(4):801-804.

4.	Kolter R, Greenberg EP: **Microbial sciences: the superficial life of microbes**. *Nature* 2006, **441**(7091):300-302.

5.	Brazelton WJ, Mehta MP, Kelley DS, Baross JA: **Physiological differentiation within a single-species biofilm fueled by serpentinization**. *MBio* 2011, **2**(4).

6.	Shimkets LJ: **Intercellular signaling during fruiting-body development of Myxococcus xanthus**. *Annu Rev Microbiol* 1999, **53**:525-549.

7.	Shi X, Wegener-Feldbrugge S, Huntley S, Hamann N, Hedderich R, Sogaard-Andersen L: **Bioinformatics and experimental analysis of proteins of two-component systems in Myxococcus xanthus**. *J Bacteriol* 2008, **190**(2):613-624.

8.	Piggot PJ, Hilbert DW: **Sporulation of Bacillus subtilis**. *Curr Opin Microbiol* 2004, **7**(6):579-586.

9.	Kaiser D: **Building a multicellular organism**. *Annu Rev Genet* 2001, **35**:103-123.

10.	Wilcox M, Mitchison GJ, Smith RJ: **Pattern formation in the blue-green alga, Anabaena. I. Basic mechanisms**. *J Cell Sci* 1973, **12**(3):707-723.

11.	Adams DG, Carr NG: **Heterocyst differentiation and cell division in the cyanobacterium Anabaena cylindrica: effect of high light intensity**. *J Cell Sci* 1981, **49**:341-352.

12.	Sakr S, Jeanjean R, Zhang CC, Arcondeguy T: **Inhibition of cell division suppresses heterocyst development in Anabaena sp. strain PCC 7120**. *J Bacteriol* 2006, **188**(4):1396-1404.

13.	Kirk DL: **A twelve-step program for evolving multicellularity and a division of labor**. *Bioessays* 2005, **27**(3):299-310.

14.	Nishii I, Miller SM: **Volvox: simple steps to developmental complexity?** *Curr Opin Plant Biol* 2010, **13**(6):646-653.

15.	Bonner JT: **Observations on polarity in the slime mold Dictyostelium discoideum**. *Biol Bull* 1950, **99**(2):143-151.

16.	Kay RR, Flatman P, Thompson CR: **DIF signalling and cell fate**. *Semin Cell Dev Biol* 1999, **10**(6):577-585.

17.	Gomer RH, Jang W, Brazill D: **Cell density sensing and size determination**. *Dev Growth Differ* 2011, **53**(4):482-494.

18.	Jang W, Gomer RH: **Initial cell type choice in Dictyostelium**. *Eukaryot Cell* 2011, **10**(2):150-155.

19.	Bowerman B: **Maternal control of pattern formation in early Caenorhabditis elegans embryos**. *Curr Top Dev Biol* 1998, **39**:73-117.

20.	Doe CQ, Bowerman B: **Asymmetric cell division: fly neuroblast meets worm zygote**. *Curr Opin Cell Biol* 2001, **13**(1):68-75.

21.	Zhong W, Chia W: **Neurogenesis and asymmetric cell division**. *Curr Opin Neurobiol* 2008, **18**(1):4-11.

22.	Knoblich JA: **Asymmetric cell division during animal development**. *Nat Rev Mol Cell Biol* 2001, **2**(1):11-20.

23.	Bruce AW, Zernicka-Goetz M: **Developmental control of the early mammalian embryo: competition among heterogeneous cells that biases cell fate**. *Curr Opin Genet Dev* 2010, **20**(5):485-491.

24.	Antczak M, Van Blerkom J: **Oocyte influences on early development: the regulatory proteins leptin and STAT3 are polarized in mouse and human oocytes and differentially distributed within the cells of the preimplantation stage embryo**. *Mol Hum Reprod* 1997, **3**(12):1067-1086.

25.	Piotrowska K, Wianny F, Pedersen RA, Zernicka-Goetz M: **Blastomeres arising from the first cleavage division have distinguishable fates in normal mouse development**. *Development* 2001, **128**(19):3739-3748.

26.	Wang L, Wang ZB, Zhang X, FitzHarris G, Baltz JM, Sun QY, Liu XJ: **Brefeldin A disrupts asymmetric spindle positioning in mouse oocytes**. *Dev Biol* 2008, **313**(1):155-166.
