# Supplementary material for: A model to explain specific cellular communications and cellular harmony:- a hypothesis of coupled cells and interactive coupling molecules
Source: Theor Biol Med Model. 2014 Sep 14;11:40. doi: 10.1186/1742-4682-11-40 (PMC4237941; doi:10.1186/1742-4682-11-40)
Supplement: Additional file 1 — 1. “Terminology”. 2. “Change in Cellular Characteristics”. 3. Note 1 “The Roles of SCD versus ACD”. Note 2. “Categories of SCD and ACD”. Note 3 “Further Examples of these AsCD Categories”. Note 4 “Change of differentiation type.”. 4. “The Effects of Growth Factors and other Bioactive Molecules 5. Note 1, “Additional Information on the Definition of a Trefone”. Note 2 “Clarification on Ligand and Binding Protein Interaction”. Note 3 “Cell Couplets – AsCD and the a-Cell and i-Cell”. Note 4 “Drosophila – Potential Trefones and ACD”. 6. “Calculated Levels of Trefones and TCC, with Cellular Actions”. 7. “Receptors for the Six IGFBPs and Cellular Effects”. 8. Note 1 “Extracellular Binding Proteins/ Soluble Receptors”. Note 2 “Cell Receptors for Binding Proteins/Soluble Receptors”. 9. Note 1. “Further Evidence for a Receptor for TCC”. Note 2 “Problems with Cross-linking Experiments”. 10. “Evidence for Binary Receptors aside from the IGF System”. 11. “Evidence for Two Receptors for a Particular Trefone, outside the IGF System”. 12. Note 1 “Mechanisms of Nuclear Localization”. Note 2 “NL of IGFBPs”. Note 3 “NL of Potential Trefones other than the IGF System”. 13. “Evidence Supporting the Existence of a-Cells and i-Cells”. 14. “Candidates with mainly a-Cell-Type or i-Cell-Type Characteristics”. 15. “Interacting Cells and Trefones not associated with the IGF System”. 16. “Heterogeneity/Variability of Cells in Culture.” 17. “Cell lines are not Typical of Normal Cells”. 18. “Cell Receptors and Cell Markers”. 19. “Defined Couplet Cells for Insulin and Glucagon; Gastrin and Histamine”. 20. “Potential Couplet Trefones”. 21. “Further Examples of Potential Trefone and Cell Couplets”. 22. “Examples of Cellular Regulation by Complexes”. 23. “Proteolytic enzymes and their inhibitors”. 24. “Expanded Definition of ‘Trefone’ and Classes of Couplet Cell Interactions”. 25. Note 1 “Extended Trefone Couplets”. Note 2 “Singlet Cells”. 26. “Background of Cancer Research”. [file 1742-4682-11-40-S1.zip › 3484671921331230_add1/3484671921331230_add03.doc]

**Additional File *Three*:-**

# (b) Cell division (CD) – Symmetric (SCD) versus Asymmetric (AsCD) – a balance of cells is achieved by complementation.

***Note 1. The Mechanisms and Roles of Asymmetric Cell Division and the Balance with Symmetric Cell Division.***

*The purpose of this Note is to summarize the extensive evidence of AsCD, its mechanisms and its role in development, to describe the causes of AsCD and to discuss the balance of AsCD with SCD.*

*Caenorhabditis elegans* and *Drosophila melanogaster* have been at the forefront of studies of the importance of AsCD, especially in development. Figure 1 shows an example of AsCD in *Drosophila.*

**FIGURE 1 . An Example of Asymmetric Cell**

**Divisions in *Drosophila.***

*The bristle mechano- sensory organs*

*(microchaete lineage) of the adult fly*

*are composed of four different cells*

*that originate from a single precursor*

*cell, pI, via two rounds of AsCD*

*with apoptosis of the glial cell***.**

*For the other cells (socket, Hair etc),*

*SCD then is required to form the required*

*functional mass of cells in each case.*

AsCD may be produced in several ways. The process may be autonomous ie. purely genetically predetermined or it may be genetic but conditional on extragenetic molecules; in the latter case, it may be cell-intrinsic, requiring the presence of ICMs separated into one progeny cell from cell division or cell-extrinsic, requiring direct contact with a neighbouring cell or requiring ICMs from the environment, perhaps secreted from other cells. Various strategies of extrinsic signalling due to ICMs in cell fate specification include inductive, gradient, antagonist, cascade and lateral signalling. In addition, the AsCD may be completed pre- or post-cytokinesis.

AsCD is often due to asymmetry in the segregation of molecules or organelles during cell division leading to different fates in equally or unequally sized offspring cells. It can be caused by the asymmetrical cortical distribution or polarisation of segregated cellular components (eg. protein**,** RNA**,** both their types or concentrations) perhaps due to directed transport along actin microfilaments into one offspring cell.

Asymmetry may also be due to different molecules associating with different centrosomes (the new-offspring centrosome *versus* the old-parent centrosome) or with their resident centrioles or peri-centriolar material or due to the number of centrosomes and the organization of the mitotic apparatus. As an example, in the AsCD of the embryo of *C. elegans*, an axis of polarity is established by the sperm-supplied centrosome leading to the asymmetry of position of PAR proteins which determines the asymmetric placement of the mitotic apparatus leading to AsCD. The *par* genes were discovered in genetic screens for regulators of cytoplasmic partitioning in the early embryo of *C. elegans*, and encode six different proteins required for asymmetric cell division by the worm zygote. Some of the PAR proteins are localized asymmetrically and form [physical](http://www.ncbi.nlm.nih.gov/pubmed/17981131) complexes with one another.

This asymmetry of CD associated with events occurring prior to or during cell division (pre-cytokinesis), causes a potential difference in cellular expression and/or in the genetic direction of the offspring.

The type of AsCD which is strictly cell autonomous (occurring independent of the environmental conditions) and occurs pre-cytokinesis, is reported in the generation of polarized blastomeres in *Xenopus* embryos. An example of AsCD which is cell-intrinsic, is in the *Drosophila* peripheral nervous system wherein Numb biases mitosis to specify distinct offspring cells. The cell-extrinsic type of AsCD involves induction of only one cell of two equipotent cells, causing different fates for the individual offspring, due to direct contact with neighbouring cells affecting spindle orientation or by secreted signalling molecules eg. Wnt protein or by matrix effects. Polar distribution of intracellular proteins may also be caused by contact with other cells or membranes. Inversely, by disrupting adherens junctions in *Drosophila* neuroepithelial cells, the process of symmetric division is converted into an asymmetric division. For T cells, AsCD can occur while the T cell is physically attached to the antigen-presenting cell and the interaction provides the cue for polarization of cell-fate determinants and subsequent orientation of the mitotic spindle for division.

In contrast, the cause of asymmetry could be post-cytokinesis and extrinsic, following an initial symmetric distribution of cellular constituents. A difference in the cell fates of the offspring can follow this SCD, because the non-uniform environment surrounding the couple induces a change in just one of the progeny. **(See Figure 2.)** The indirect differentiation of Fig.2(b) is accepted here because the priming event is compatible with the definition of “differentiation” in **Additional File** ***One*** which requires cell division for differentiation. Otherwise, the change is simply an “adaptation”.

**FIGURE 2. Post-Cell Division, Different-**

**iative Change Caused by the Extracellular**

**Environment – Two Possibilities.**

***(a)*** *Any progenitor cell (C_0_) could produce a*

*differentiated cell (C_1_) following a cell division*

*where a change (even a transformation) occurs*

*to only one progeny due to unequal exposure*

*to the extracellular constituents. Here the con-*

*centrated part of the gradient (black) produces*

*or allows a change, by activation of specific*

*genes. The alternative is that the dilute part*

*of the gradient (white) inhibits the differentiation*

*that occurs to the other cell.*

***(b)*** *The gradient here “primes” the C_0_ cell to*

*become a committed cell (C_0_^*^) which then pro-*

*duces two differentiated cells.*

As an example, diffusible gradients within

the multicellular embryo of the brown alga are responsible for AsCD and cell fates.

Alternatively, a difference may occur because some stochastic choice produces a change in one progeny and a nil or compensating change in the other progeny – lateral signalling. For example, from the two equivalent cells derived from a SCD within the gonad of *C. elegans*, the cell with the highest activity of the receptor Lin 12 which binds the ligand lag-2, becomes a ventral uterine precursor cell while the other progeny becomes the anchor cell. The cells’ fates following SCD may also be determined by contact with the neighbouring cells as occurs in the four-cell stage of the *C. elegans* embryo to form different AB cells.

The description of an event in development as “stochastic” may be an inappropriate description because the event is not random but is controlled by genetic predisposition or environmental factors eg. growth factors or intercellular contacts.

In assessing the relative importance of AsCD versus SCD, an important feature is that when examined by cell numbers, AsCD and SCD can produce the same result. **(See Figure 3.)** SCD can produce two offspring cells that are identical to the parent cell or two cells that are identical to each other but different to the parent ie. differentiated. However, AsCD of cells is often conservative ie. one progeny is identical to the parent cell and the second progeny is different from the parent. Stem cells can undergo both types of SCD and if in equal numbers, will achieve the same result as AsCD; this is sometimes referred to as “stochastic” differentiation .

**FIGURE 3. Identical Cellular Products Derived from Two AsCDs and Two SCDs.**

***(a)*** *When two stem cells (Cs_0_) each undergo*

*asymmetric cell division, two stem cells and*

*two differentiated cells (Cs_1_) are produced.*

*The ratio of Cs_0_ to Cs_1_ produced from these*

*two divisions will always be 1:1, but subseq-*

*uent SCDs will produce the versatility in the*

*number of different cells required.*

***(b)*** *When two stem cells (Cs_0_) each undergo*

*symmetric cell division with one self-*

*renewing division and the other producing*

*two differentiated cells, again two stem cells*

*and two differentiated cells (Cs_1_) are*

*produced. The ratio of Cs_0_ to Cs_1_, will again*

*be 1:1 from these two divisions but subseq-*

*uent SCDs will again produce the versatility*

*in the number of different cells required*.

The “stem cell niche” is a microenvironment where soluble signals or cell-cell interactions control the fate of stem cells to produce niche-induced AsCD. In the *Drosophila* *melanogaster* testis, both germline stem cells and cyst stem cells in the niche divide asymmetrically with self-renewal, involving AsCD in nearly 100% of the latter cells. Within the niche at the base of the vertebrate endodermal intestinal crypt, again CD is usually asymmetric with self-renewal and only occasional symmetric division, regulated by growth factors and cytokines from neighboring mesenchymal cells. However, the genetic lineage is critical and the possible fates of even a stem cell would be determined by both its intrinsic programming and signals from the mesenchyme.

While AsCD is often included in the definition of a stem cell, SCD of stem cells is also important in producing stem cell populations. [Stem cells](http://www.ncbi.nlm.nih.gov/pmc/articles/PMC2267762/) are then thought to balance self-renewal and differentiation through judicious variation of asymmetric and symmetric divisions. The balance is not hardwired into the genes but is responsive to extrinsic and intrinsic cues. The p53 anti-oncogene can induce cells to switch from SCD to an AsCD stem cell growth pattern by inhibiting GMP formation; opposingly but in a related way**,** xanthosine, a precursor of GMP has been shown to switch cells from AsCD to SCD in adult rat stem cell lines.

Perpetual AsCD, combined with SCD, occurs also in mammalian hematopoietic progenitor cells; in thymic lymphocytes, >40% of the cells are dividing asymmetrically. In the epidermis, while progenitor cells may divide symmetrically to produce either two cycling progeny or two non-cycling progeny, the bulk of cells divide asymmetrically. Other examples of AsCD include neurogenesis and gliogenesis in discrete regions of the mammalian brain.

Other examples of AsCD are well documented in a variety of cells of land plants (maize, *Arabidopsis*) green and brown algae, zebrafish, sea urchin, sea squirt, leeches and grasshoppers.

However, even in bacteria where exponential doubling by SCD is usually assumed, AsCD is not uncommon as evidenced in some of the examples in **Additional File *Two***. A further example is *Mycobacterium tuberculosis* where four types of cell division were observed in various isolates in exponential growth and five different types of cell shapes were observed. Indeed, each and every cell division may be functionally asymmetric with the presence of an “old” pole in one offspring and a newly synthesised one in the other**.** Certainly**,** polarisation is not uncommon and is maybe even universal. While bacterial intelligence is associated with quorum-sensing and associated differentiation, it seems that AsCD is not necessarily a part of this response to community overcrowding. An exception is *B. subtilis* where the quorum-sensing pentapeptide CSF (Competence and Sporulation Factor), when at high concentrations, inhibits competence and stimulates sporulation which is achieved by AsCD.

The decision whether SCD or AsCD occurs is then determined by “intrinsic” genetic programs and/or by external signals. The asymmetric first meiotic division in mouse oocytes can be converted, by the elevation of the intracellular cAMP level, to a symmetric cell division. A presumption would be that extracellular signals acting via G protein-coupled receptors could therefore influence the cell’s decision. The opposite occurs naturally within the optic lobes of *Drosophila* where the neural stem cells switch from symmetric to asymmetric division as they progress from neuroepithelial cells undergoing SCD, to neuroblasts undergoing AsCD, by a process partially controlled by Notch receptor levels. The type of CD in glioma stem cells (GSCs) depends on the growth environment – under expansive growth conditions, SCD predominates and produces two GSCs or two differentiated progeny, while with growth factor withdrawal, AsCD increases to 21% from 4% .

In a *Helobdella* leech, which exhibits asymmetry in the first embryonic division, inhibition of normal microtubule function forces symmetric division in a significant number of embryos.

Another example is seen with the blue-green algae *Anabaena,* where upon exposure to intense light, there is an increase in the percentage of cell divisions that produce identical offspring cells and double, not single heterocysts are formed. A similar increase occurs with addition of 7-azatryptophan.

Obviously, in embryogenesis and subsequent development, the sequence of cell division, AsCD and/or SCD, needs to be specifically programmed, unambiguous and occurring in a controlled environment to produce a normal fetus. In cell turnover, tissue maintenance and repair in the adult, while similar control is required, there needs to be more flexibility and adaptation to suit the circumstances.

The nature of the differentiation lineage of specifically formed progenitor cells will depend on the ICMs in the environment. The specific AsCDs and SCDs of hemopoietic stem cells are promoted by interleukin-1 (IL-1), IL-3, IL-6, and granulocyte colony-stimulating factor (G-CSF), granulocyte/macrophage-CSF (GM-CSF) and erythropoietin. More recently, in addition to the above, other hematopoietic growth factors - macrophage CSF (M-CSF) and stem cell factor - are deemed to be involved in differentiation and survival of blood stem cells. But there is doubt expressed about whether cytokines instruct stem cells, pre-cytokinesis, to differentiate directly into a specific lineage or whether stochastically, cytokines stimulate the proliferation or allow the survival of lineage-restricted cells from a pool formed after cell division. However, M-CSF and G-CSF can actually instruct murine hematopoietic progenitor cells in lineage choice rather than these cytokines being selective of just the right cell types.

***Note 2. Categories of Symmetric (SCDs) and Asymmetrical Cell Divisions (AsCDs)***

*The purpose of this Note is to illustrate the variety of options of a cell especially with AsCD.*

Within the types of symmetric and asymmetric cell divisions, there are many theoretical possibilities which are uniquely and schematically depicted in **Figure 1**.

These processes and outcomes of SCD and AsCD can be described and categorized in a novel and complete way as follows:-

(A) Symmetric:- (i)	Self-Renewal Expansion (SRE)

(ii) Lineage Commitment to Differentiation (LCDf)

(iii) Commitment to Terminal Differentiation (CTD)

(B) Asymmetric (i)	Self-Renewal (SR) with LCDf	(v)	LCDf with CTD

(ii) SR with CTD	(vi) LCDf with a Dead End (DE)

(iii) Dual CTD	(vii) CTD with a DE

(iv) Dual LCDf

**FIGURE 1 – A Diagram of Possible Types of Cell Division, with Named Cells.**

*Cell division may be (A) Symmetric ie. two identical cells produced at cytokinesis (SCD) from the parent cell [C]:-*

*(i) Cells identical to parent cell = Self-Renewal Expansion (SRE).*

*(ii) Differentiation to a new type of cell [C/D] capable of further cell division = Lineage Commitment to Differentiation (LCDf).*

*(iii) Differentiation to a new type of cell [C/TD] not capable of further cell division = Commitment to Terminal Differentiation (CTD).*

*Cell division may be (B) Asymmetric ie. non-identical cells produced:-*

*(i) One identical to parent (self-renewal), the other differentiated [C/D] = Self-Renewal (SR) with LCDf.*

*(ii) One identical to parent, the other terminally differentiated [C/TD] = SR with CTD.*

*(iii) Both terminally differentiated, but different from each other [C/TD_1_, C/TD_2_] = Dual CTD.*

*(iv) Both differentiated, but different from each other [C/D_1_, C/D_2_] = Dual LCDf.*

*(v) Both differentiated, one terminally [C/D, C/TD] = LCDf with CTD.*

*(vi) Both differentiated, one apoptosed [C/D, C/A] = LCDf with a Dead End (DE).*

*(vii) Both differentiated, one terminally, one apoptosed [C/TD, C/A] = CTD with a DE. These outcomes may occur pre- or post-cytokinesis.*

Within the options described in **Figure 1**, there are even more possible outcomes:-

1. For SCD options, the pair of C/D cells derived from a nominated cell (C) by SCD with LCDf [ie. A(ii)], may not be a single option. In other words, the C/D cells may be a pair of C/D_1_ cells or a pair of C/D_2_ cells or of C/D_3_ cells. If the original cell were a stem cell, then these C/D_n_ cells would be progenitor cells for different lineages of cells. Also for A(iii), the C/TD cells need not be a single type of cell option, with a pair of C/TD_1_ or a pair of C/TD_2_ cells etc as possible outcomes. Cell fate would be determined by specific ICMs.

2. Similarly, for AsCD options from a nominated cell, the C/D cell of B(i) may be variable. Also, the C/D_1_ and C/D_2_ progeny cells of B(iv) may be variable eg. C/D_3_, C/D_4_ etc. Equally, cells designated C/TD, C/TD_1_ and C/TD_2_ may include a variety of possible cells.

3. A C/D cell derived from a Lineage Commitment to Differentiation (LCDf), from ASD or AsCD, may not be immediately differentiated. It may be committed to a certain cell lineage but only actually changes cell type upon exposure to a differentiation factor. Similarly, for a C/TD cell, actual differentiation to a terminal state only occurs with exposure to a specific differentiation factor.

4. A C/TD cell cannot undergo an AsCD but could undergo a SCD. This cell has reached a final state but can produce copies of itself. All of the cell progeny with double arrows in Figure3 may undergo every option of SCD and AsCD.

5. A C/A cell cannot undergo any CD and may hit a Dead End immediately upon formation. However, it may live for a long period of time as a dedicated cell before apoptosis or destruction by other means such as engulfment.

6. The Figure does not include possible reversal of differentiation or transdifferentiation both of which would include cell division. The arrows do not show this possibility of reversal.

(See **Additional File *One “Terminology”*** for definitions and **Additional File *Three, Note 3 “Further Examples of these SCD and AsCD Categories”*** for further discussion on these possible outcomes of SCD and AsCD.)

There is also another description of AsCD not included above. This is the special case of the *Caulobacter crescentus* cell where, after AsCD with LCDf, one cell progeny may differentiate into the other progeny. The parent cell matures into a “predivisional” cell with flagellum which divides asymmetrically to produce two progeny, the flagellated swarmer cell and the sessile stalked cell. But the former cell can lose its flagellum and mature to become a stalked cell, so that an asymmetric dual LCDf becomes effectively, after a period of time, a SCD with LCDf. Also, the stalked cell can mature back to the mother cell so that the original cell division could also be considered as effectively an AsCD with Self-Renewal and transient LCDf, but eventually becomes a SCD with SRE Of course, this cyclical behavior may be more a survival mechanism than a division of labour.

Interestly, in terms of evolution, it may be that asymmetric division without self-renewal preceded the asymmetric division with self-renewal.

In summary, cells have many options:- those listed above and those listed in ***Note 4*** below.

***Note 3. Further Examples of these AsCD Categories***

*The purpose of this Note is to provide further examples of the AsCD categories listed in Figure 1, Note 2 above.*

Symmetric and asymmetrical cell divisions can be categorized as described in **Figure 1, *Note 2*** above. Some further examples of these AsCD categories follow.

AsCD with self-renewal (SR) and Lineage Commitment to Differentiation (LCDf) [ie. B(i)], is considered to be one important, perhaps essential, part of a stem cell’s function in forming or maintaining a lineage of cells. Yet, AsCD with double commitment to differentiaton (Dual LCDf) [ie. B(iv)], is also considered to be part of the lineage division sequence proposed in certain stem cell niches.

These two examples also occur in *Saccharomyces cerevisiae* which has cell types D (Daughter) and M (Mother) and two mating types of haploid cells, alpha and sigma (α and σ). AsCD with self-renewal, B(i), occurs ( Dα → Dα + Mα; Dσ → Dσ + Mσ) as does AsCD with dual LCDf, B(iv), in “pair switch” (Mα → Mσ + Dσ ; Mσ → Mα + Dα). A similar pattern occurs with *Schizosaccharomyces pombe.*

An example of B(iii), dual CTD, is known in the rat retina, where a single retinal precursor cell can produce two cells of different types at the last mitosis before maturation.

There are a number of examples of a C/A or Dead End (DE) cell, associated with either LCDf, B(vi), or with CTD, B(vii). This “Dead End” is likely via apoptosis but there are at least three possibilities:-

***(i) Programmed cell death – perhaps Apoptosis***

There are a number of examples from various cell lineages. As chick chrondrocytes change to osteoblast-like cells, there is an AsCD where one offspring remains viable and the other one dies. In *C. elegans*, within the HSN/PHB lineage of neurons, a neuroblast divides asymmetrically to produce a HSN/PHB precursor cell plus a progeny that undergoes programmed cell death. Similarly, in the microchaete lineage of *Drosophila*, the AsCD of a pIIb cell produces a pIIIb cell plus a glial cell which undergoes apoptosis. Programmed cell death, along with AsCD and SCD, is a mechanism of regulation of the numbers of hematopoietic stem cells. Here, the death of one cell could follow an AsCD but SCD would be a more efficient way to expose a genetic program for cell death whereby both progeny would die. This assumes that the genetic program is normally hidden and only exposed within the cell cycle and CD.

In flowering plants, the tetrad of microspores each undergo AsCD with production of a generative cell and a terminally-differentiated vegetative cell.

Apoptosis could also be part of the bacterial toxin-antitoxin system where post-segregational killing of the cell that loses certain genes occurs and it suffers the destructive effect of the toxin.

ICM molecules such as transforming growth factor-beta (TGF-beta) may participate in apoptosis. The apoptotic response in fetal hepatocytes depends upon their state of differentiation. With very high concentrations and/or long-term exposure of fetal hepatocytes to TGF-beta1, cell death occurs by an apoptotic mechanism. However, 40-50% of cells always survive. This process of cell survival is coincident with differentiation (with changes in morphology and phenotype, with cells showing a fibroblastic appearance and eliciting an epithelial-fibroblastic transition). This could be associated with cell division type B(vi) differentiation with apoptosis, where 50% of cells would be expected to be of a differentiated type.

***(ii) Fusion***

The Dead End state of a cell could be a fused cell. Multinucleated muscle fibres are considered to be irretrievably differentiated; the deprivation of a growth factor can cause the differentiation of myoblasts followed by fusion.

Another example occurs in the morphogenesis of epithelia during development of *C. elegans*, where the anchor cell initially acts to induce and to organize specialization of cells by intercellular signalling pathways and generates progeny with which the anchor cell fuses to form the multinucleate “utse” cell.

***(iii) Nucleus separation with phagocytosis***

The path to a Dead End state of a cell could be biphasic. Primitive erythroblasts enucleate by a cellular division to generate erythrocytes and the nucleus-containing pyrenocytes. The erythrocytes exist for about 115 days but macrophages engulf the pyrenocytes quickly as they send an “eat me” signal to macrophages by loss of phosphatidyl serine asymmetry.

***Note 4. Change of differentiation type – options of a differentiated cell***

*The purpose of this Note is to provide examples of the options of a differentiated cell in terms of outcomes of possible reversal or change in differentiated type, each involving cell division.*

Following AsCD and/or SCD, a differentiated cell type may be formed. This cell may continue to divide by SCD and /or AsCD as part of a cell lineage. As opposed to this forward direction of growth, potentially, this differentiated state can be reversed or changed and dedifferentiation (DD) or transdifferentiation (TD) can occur. By definition, cell division is here considered necessary for DD and TD. A C/D cell (formed by SCD or AsCD) could revert either by SCD to produce two of the original cells or by AsCD to produce an original cell plus itself or plus a different C/D cell. For DD, the following possibilities then exist for the situation of a stem or progenitor cell (the original cell, o-Cell) and the possible C/D cells derived from cell division (C/Ds). These C/D cells are generically identified as an n-Cell or specifically identified as a b-Cell, c-Cell, d-Cell, e-Cell, or f-Cell, where each represents the initial cell of a lineage. (LCDf = Lineage Commitment to Differentiation; SR = Self-Renewal)

If, as in SCD, an o-Cell → 2x n-Cell ( eg. 2x b-Cell by LCDf), then the following reversion types could occur from the two progeny:-

- by SCD	- 2x b-Cell → 4x o-Cell {2x Dual Reversion (Rv)}

or - 1x b-Cell → 2x o-Cell and 1x b-Cell → 2x c-Cell (Dual Rv + LCDf)

- by AsCD - 2x b-Cell → 2x o-Cell + 2x b-Cell {Single Rv with SR of b-Cell}

or - 2x b-Cell → 2x o-Cell + 2x any other n-Cell (Single Rv with LCDf)

If, as in AsCD, an o-Cell → o-Cell* + b-Cell (SR of o-Cell with LCDf)

or an o-Cell → c-Cell	+ d-Cell (Dual LCDf)

or an o-Cell → e-Cell	+ f-Cell	(Dual LCDf), then the following reversion types could occur from any specified cell (with no reversion by the o-Cell*)

- by SCD	- each n-Cell → 2x o-Cell (Dual Rv)

- by AsCD - each n-Cell → o-Cell + the same n-Cell (Single Rv with SR of n-Cell)

- each n-Cell → o-Cell + any other n-Cell (Single Rv with dual LCDf)

In summary, there are many options for reversal. In a tissue, there is then the possibility of many options of both forward-growth direction and its reversal. Differentiation, DD, TD etc are all options of most cells and the direction of cell divisions would depend on the environment of ICMs.

If the options for the o-Cell* (SCD and AsCD) are added, then other cells will be obtained eg. SCD could produce 2x c-Cell. If it is accepted that SCD of an o-Cell can produce not just one pair of specifically differentiated cells but as many types of differentiated cells as AsCD (ie. of n-Cells, or specifically b-Cells, c-Cells etc), the conclusion is that the end result of reversal is no different for cells reversed by AsCD or by SCD. This is the same sort of conclusion as illustrated in **Note 1, Figure 3** (above) for forward growth.

**REFERENCES:-**

1.	Lu B, Jan LY, Jan YN: **Asymmetric cell division: lessons from flies and worms**. *Curr Opin Genet Dev* 1998, **8**(4):392-399.

2.	Gonczy P: **Mechanisms of asymmetric cell division: flies and worms pave the way**. *Nat Rev Mol Cell Biol* 2008, **9**(5):355-366.

3.	Gho M, Bellaiche Y, Schweisguth F: **Revisiting the Drosophila microchaete lineage: a novel intrinsically asymmetric cell division generates a glial cell**. *Development* 1999, **126**(16):3573-3584.

4.	Fichelson P, Gho M: **The glial cell undergoes apoptosis in the microchaete lineage of Drosophila**. *Development* 2003, **130**(1):123-133.

5.	Horvitz HR, Herskowitz I: **Mechanisms of asymmetric cell division: two Bs or not two Bs, that is the question**. *Cell* 1992, **68**(2):237-255.

6.	Edlund T, Jessell TM: **Progression from extrinsic to intrinsic signaling in cell fate specification: a view from the nervous system**. *Cell* 1999, **96**(2):211-224.

7.	Schneider SQ, Bowerman B: **Cell polarity and the cytoskeleton in the Caenorhabditis elegans zygote**. *Annu Rev Genet* 2003, **37**:221-249.

8.	Goldstein B, Macara IG: **The PAR proteins: fundamental players in animal cell polarization**. *Dev Cell* 2007, **13**(5):609-622.

9.	Long RM, Singer RH, Meng X, Gonzalez I, Nasmyth K, Jansen RP: **Mating type switching in yeast controlled by asymmetric localization of ASH1 mRNA**. *Science* 1997, **277**(5324):383-387.

10.	Broadus J, Doe CQ: **Extrinsic cues, intrinsic cues and microfilaments regulate asymmetric protein localization in Drosophila neuroblasts**. *Curr Biol* 1997, **7**(11):827-835.

11.	Lambert JD, Nagy LM: **Asymmetric inheritance of centrosomally localized mRNAs during embryonic cleavages**. *Nature* 2002, **420**(6916):682-686.

12.	Chia W, Somers WG, Wang H: **Drosophila neuroblast asymmetric divisions: cell cycle regulators, asymmetric protein localization, and tumorigenesis**. *J Cell Biol* 2008, **180**(2):267-272.

13.	Yamashita YM, Mahowald AP, Perlin JR, Fuller MT: **Asymmetric inheritance of mother versus daughter centrosome in stem cell division**. *Science* 2007, **315**(5811):518-521.

14.	Macara IG, Mili S: **Polarity and differential inheritance--universal attributes of life?** *Cell* 2008, **135**(5):801-812.

15.	Lindner AB, Madden R, Demarez A, Stewart EJ, Taddei F: **Asymmetric segregation of protein aggregates is associated with cellular aging and rejuvenation**. *Proc Natl Acad Sci U S A* 2008, **105**(8):3076-3081.

16.	Yamashita YM, Fuller MT: **Asymmetric centrosome behavior and the mechanisms of stem cell division**. *J Cell Biol* 2008, **180**(2):261-266.

17.	Stearns T: **Centrosome duplication. a centriolar pas de deux**. *Cell* 2001, **105**(4):417-420.

18.	Shimizu T, Ishii R, Takahashi H: **Unequal cleavage in the early Tubifex embryo**. *Dev Growth Differ* 1998, **40**(3):257-266.

19.	Cowan CR, Hyman AA: **Asymmetric cell division in C. elegans: cortical polarity and spindle positioning**. *Annu Rev Cell Dev Biol* 2004, **20**:427-453.

20.	Muller HA: **Of mice, frogs and flies: generation of membrane asymmetries in early development**. *Dev Growth Differ* 2001, **43**(4):327-342.

21.	Guo M, Jan LY, Jan YN: **Control of daughter cell fates during asymmetric division: interaction of Numb and Notch**. *Neuron* 1996, **17**(1):27-41.

22.	Cheng J, Tiyaboonchai A, Yamashita YM, Hunt AJ: **Asymmetric division of cyst stem cells in Drosophila testis is ensured by anaphase spindle repositioning**. *Development* 2011, **138**(5):831-837.

23.	Goldstein B, Takeshita H, Mizumoto K, Sawa H: **Wnt signals can function as positional cues in establishing cell polarity**. *Dev Cell* 2006, **10**(3):391-396.

24.	Congdon KL, Reya T: **Divide and conquer: how asymmetric division shapes cell fate in the hematopoietic system**. *Curr Opin Immunol* 2008, **20**(3):302-307.

25.	Lu B, Roegiers F, Jan LY, Jan YN: **Adherens junctions inhibit asymmetric division in the Drosophila epithelium**. *Nature* 2001, **409**(6819):522-525.

26.	Oliaro J, Van Ham V, Sacirbegovic F, Pasam A, Bomzon Z, Pham K, Ludford-Menting MJ, Waterhouse NJ, Bots M, Hawkins ED *et al*: **Asymmetric cell division of T cells upon antigen presentation uses multiple conserved mechanisms**. *J Immunol* 2010, **185**(1):367-375.

27.	Bouget FY, Berger F, Brownlee C: **Position dependent control of cell fate in the Fucus embryo: role of intercellular communication**. *Development* 1998, **125**(11):1999-2008.

28.	Seydoux G, Greenwald I: **Cell autonomy of lin-12 function in a cell fate decision in C. elegans**. *Cell* 1989, **57**(7):1237-1245.

29.	Wilkinson HA, Fitzgerald K, Greenwald I: **Reciprocal changes in expression of the receptor lin-12 and its ligand lag-2 prior to commitment in a C. elegans cell fate decision**. *Cell* 1994, **79**(7):1187-1198.

30.	Evans TC, Crittenden SL, Kodoyianni V, Kimble J: **Translational control of maternal glp-1 mRNA establishes an asymmetry in the C. elegans embryo**. *Cell* 1994, **77**(2):183-194.

31.	Zernicka-Goetz M, Huang S: **Stochasticity versus determinism in development: a false dichotomy?** *Nat Rev Genet* 2010, **11**(11):743-744.

32.	Potten CS, Loeffler M: **Stem cells: attributes, cycles, spirals, pitfalls and uncertainties. Lessons for and from the crypt**. *Development* 1990, **110**(4):1001-1020.

33.	Clayton E, Doupe DP, Klein AM, Winton DJ, Simons BD, Jones PH: **A single type of progenitor cell maintains normal epidermis**. *Nature* 2007, **446**(7132):185-189.

34.	Till JE, McCulloch EA, Siminovitch L: **A STOCHASTIC MODEL OF STEM CELL PROLIFERATION, BASED ON THE GROWTH OF SPLEEN COLONY-FORMING CELLS**. *Proc Natl Acad Sci U S A* 1964, **51**:29-36.

35.	Ogawa M: **Hemopoietic stem cells: stochastic differentiation and humoral control of proliferation**. *Environ Health Perspect* 1989, **80**:199-207.

36.	Lin H: **The stem-cell niche theory: lessons from flies**. *Nat Rev Genet* 2002, **3**(12):931-940.

37.	Knoblich JA: **Mechanisms of asymmetric stem cell division**. *Cell* 2008, **132**(4):583-597.

38.	de Cuevas M, Matunis EL: **The stem cell niche: lessons from the Drosophila testis**. *Development* 2011, **138**(14):2861-2869.

39.	Bach SP, Renehan AG, Potten CS: **Stem cells: the intestinal stem cell as a paradigm**. *Carcinogenesis* 2000, **21**(3):469-476.

40.	Leedham SJ, Brittan M, McDonald SA, Wright NA: **Intestinal stem cells**. *J Cell Mol Med* 2005, **9**(1):11-24.

41.	Wright NA: **Epithelial stem cell repertoire in the gut: clues to the origin of cell lineages, proliferative units and cancer**. *Int J Exp Pathol* 2000, **81**(2):117-143.

42.	Morrison SJ, Kimble J: **Asymmetric and symmetric stem-cell divisions in development and cancer**. *Nature* 2006, **441**(7097):1068-1074.

43.	Wu M, Kwon HY, Rattis F, Blum J, Zhao C, Ashkenazi R, Jackson TL, Gaiano N, Oliver T, Reya T: **Imaging hematopoietic precursor division in real time**. *Cell Stem Cell* 2007, **1**(5):541-554.

44.	Sherley JL, Stadler PB, Johnson DR: **Expression of the wild-type p53 antioncogene induces guanine nucleotide-dependent stem cell division kinetics**. *Proc Natl Acad Sci U S A* 1995, **92**(1):136-140.

45.	Lee HS, Crane GG, Merok JR, Tunstead JR, Hatch NL, Panchalingam K, Powers MJ, Griffith LG, Sherley JL: **Clonal expansion of adult rat hepatic stem cell lines by suppression of asymmetric cell kinetics (SACK)**. *Biotechnol Bioeng* 2003, **83**(7):760-771.

46.	Sugimoto M, Yasuda T: **Asymmetric (differential) cell division of thymic lymphocytes by means of cytoplasmic polarization: possible biological meanings**. *Thymus* 1983, **5**(5-6):297-310.

47.	Lechler T, Fuchs E: **Asymmetric cell divisions promote stratification and differentiation of mammalian skin**. *Nature* 2005, **437**(7056):275-280.

48.	Zhong W, Feder JN, Jiang MM, Jan LY, Jan YN: **Asymmetric localization of a mammalian numb homolog during mouse cortical neurogenesis**. *Neuron* 1996, **17**(1):43-53.

49.	Bonaguidi MA, Wheeler MA, Shapiro JS, Stadel RP, Sun GJ, Ming GL, Song H: **In vivo clonal analysis reveals self-renewing and multipotent adult neural stem cell characteristics**. *Cell* 2011, **145**(7):1142-1155.

50.	Scheres B, Benfey PN: **Asymmetric Cell Division in Plants**. *Annu Rev Plant Physiol Plant Mol Biol* 1999, **50**:505-537.

51.	Farnia P, Mohammad RM, Merza MA, Tabarsi P, Zhavnerko GK, Ibrahim TA, Kuan HO, Ghanavei J, Ranjbar R, Poleschuyk NN *et al*: **Growth and cell-division in extensive (XDR) and extremely drug resistant (XXDR) tuberculosis strains: transmission and atomic force observation**. *Int J Clin Exp Med* 2010, **3**(4):308-314.

52.	Shapiro L, Losick R: **Protein localization and cell fate in bacteria**. *Science* 1997, **276**(5313):712-718.

53.	Stewart EJ, Madden R, Paul G, Taddei F: **Aging and Death in an Organism That Reproduces by Morphologically Symmetric Division**. *PLoS Biol* 2005, **3**(2):e45.

54.	Lazazzera BA, Solomon JM, Grossman AD: **An exported peptide functions intracellularly to contribute to cell density signaling in B. subtilis**. *Cell* 1997, **89**(6):917-925.

55.	Chen D, Zhang Y, Yi Q, Huang Y, Hou H, Hao Q, Cooke HJ, Li L, Sun Q, Shi Q: **Regulation of asymmetrical cytokinesis by cAMP during meiosis I in mouse oocytes**. *PLoS One* 2012, **7**(1):e29735.

56.	Egger B, Gold KS, Brand AH: **Regulating the balance between symmetric and asymmetric stem cell division in the developing brain**. *Fly (Austin)* 2011, **5**(3):237-241.

57.	Lathia JD, Hitomi M, Gallagher J, Gadani SP, Adkins J, Vasanji A, Liu L, Eyler CE, Heddleston JM, Wu Q *et al*: **Distribution of CD133 reveals glioma stem cells self-renew through symmetric and asymmetric cell divisions**. *Cell Death Dis* 2011, **2**:e200.

58.	Ren X, Weisblat DA: **Asymmetrization of first cleavage by transient disassembly of one spindle pole aster in the leech Helobdella robusta**. *Dev Biol* 2006, **292**(1):103-115.

59.	Adams DG, Carr NG: **Heterocyst differentiation and cell division in the cyanobacterium Anabaena cylindrica: effect of high light intensity**. *J Cell Sci* 1981, **49**:341-352.

60.	Adams DG, Carr NG: **The developmental biology of heterocyst and akinete formation in cyanobacteria**. *Crit Rev Microbiol* 1981, **9**(1):45-100.

61.	Whichard ZL, Sarkar CA, Kimmel M, Corey SJ: **Hematopoiesis and its disorders: a systems biology approach**. *Blood* 2010, **115**(12):2339-2347.

62.	Rieger MA, Hoppe PS, Smejkal BM, Eitelhuber AC, Schroeder T: **Hematopoietic cytokines can instruct lineage choice**. *Science* 2009, **325**(5937):217-218.

63.	Goley ED, Iniesta AA, Shapiro L: **Cell cycle regulation in Caulobacter: location, location, location**. *J Cell Sci* 2007, **120**(Pt 20):3501-3507.

64.	Gaziova I, Bhat KM: **Generating asymmetry: with and without self-renewal**. *Prog Mol Subcell Biol* 2007, **45**:143-178.

65.	Watt FM, Hogan BL: **Out of Eden: stem cells and their niches**. *Science* 2000, **287**(5457):1427-1430.

66.	Herskowitz I: **Life cycle of the budding yeast Saccharomyces cerevisiae**. *Microbiol Rev* 1988, **52**(4):536-553.

67.	Bahler J, Pringle JR: **Pom1p, a fission yeast protein kinase that provides positional information for both polarized growth and cytokinesis**. *Genes Dev* 1998, **12**(9):1356-1370.

68.	Turner DL, Cepko CL: **A common progenitor for neurons and glia persists in rat retina late in development**. *Nature* 1987, **328**(6126):131-136.

69.	Kroemer G, Galluzzi L, Vandenabeele P, Abrams J, Alnemri ES, Baehrecke EH, Blagosklonny MV, El-Deiry WS, Golstein P, Green DR *et al*: **Classification of cell death: recommendations of the Nomenclature Committee on Cell Death 2009**. *Cell Death Differ* 2009, **16**(1):3-11.

70.	Roach HI, Erenpreisa J, Aigner T: **Osteogenic differentiation of hypertrophic chondrocytes involves asymmetric cell divisions and apoptosis**. *J Cell Biol* 1995, **131**(2):483-494.

71.	Guenther C, Garriga G: **Asymmetric distribution of the C. elegans HAM-1 protein in neuroblasts enables daughter cells to adopt distinct fates**. *Development* 1996, **122**(11):3509-3518.

72.	Domen J: **The role of apoptosis in regulating hematopoietic stem cell numbers**. *Apoptosis* 2001, **6**(4):239-252.

73.	Bedinger P: **The remarkable biology of pollen**. *Plant Cell* 1992, **4**(8):879-887.

74.	Van Melderen L, Saavedra De Bast M: **Bacterial toxin-antitoxin systems: more than selfish entities?** *PLoS Genet* 2009, **5**(3):e1000437.

75.	Sanchez A, Alvarez AM, Lopez Pedrosa JM, Roncero C, Benito M, Fabregat I: **Apoptotic response to TGF-beta in fetal hepatocytes depends upon their state of differentiation**. *Exp Cell Res* 1999, **252**(2):281-291.

76.	Clegg CH, Linkhart TA, Olwin BB, Hauschka SD: **Growth factor control of skeletal muscle differentiation: commitment to terminal differentiation occurs in G1 phase and is repressed by fibroblast growth factor**. *J Cell Biol* 1987, **105**(2):949-956.

77.	Newman AP, Sternberg PW: **Coordinated morphogenesis of epithelia during development of the Caenorhabditis elegans uterine-vulval connection**. *Proc Natl Acad Sci U S A* 1996, **93**(18):9329-9333.

78.	McGrath KE, Kingsley PD, Koniski AD, Porter RL, Bushnell TP, Palis J: **Enucleation of primitive erythroid cells generates a transient population of "pyrenocytes" in the mammalian fetus**. *Blood* 2008, **111**(4):2409-2417.
