# Supplementary material for: A model to explain specific cellular communications and cellular harmony:- a hypothesis of coupled cells and interactive coupling molecules
Source: Theor Biol Med Model. 2014 Sep 14;11:40. doi: 10.1186/1742-4682-11-40 (PMC4237941; doi:10.1186/1742-4682-11-40)
Supplement: Additional file 1 — 1. “Terminology”. 2. “Change in Cellular Characteristics”. 3. Note 1 “The Roles of SCD versus ACD”. Note 2. “Categories of SCD and ACD”. Note 3 “Further Examples of these AsCD Categories”. Note 4 “Change of differentiation type.”. 4. “The Effects of Growth Factors and other Bioactive Molecules 5. Note 1, “Additional Information on the Definition of a Trefone”. Note 2 “Clarification on Ligand and Binding Protein Interaction”. Note 3 “Cell Couplets – AsCD and the a-Cell and i-Cell”. Note 4 “Drosophila – Potential Trefones and ACD”. 6. “Calculated Levels of Trefones and TCC, with Cellular Actions”. 7. “Receptors for the Six IGFBPs and Cellular Effects”. 8. Note 1 “Extracellular Binding Proteins/ Soluble Receptors”. Note 2 “Cell Receptors for Binding Proteins/Soluble Receptors”. 9. Note 1. “Further Evidence for a Receptor for TCC”. Note 2 “Problems with Cross-linking Experiments”. 10. “Evidence for Binary Receptors aside from the IGF System”. 11. “Evidence for Two Receptors for a Particular Trefone, outside the IGF System”. 12. Note 1 “Mechanisms of Nuclear Localization”. Note 2 “NL of IGFBPs”. Note 3 “NL of Potential Trefones other than the IGF System”. 13. “Evidence Supporting the Existence of a-Cells and i-Cells”. 14. “Candidates with mainly a-Cell-Type or i-Cell-Type Characteristics”. 15. “Interacting Cells and Trefones not associated with the IGF System”. 16. “Heterogeneity/Variability of Cells in Culture.” 17. “Cell lines are not Typical of Normal Cells”. 18. “Cell Receptors and Cell Markers”. 19. “Defined Couplet Cells for Insulin and Glucagon; Gastrin and Histamine”. 20. “Potential Couplet Trefones”. 21. “Further Examples of Potential Trefone and Cell Couplets”. 22. “Examples of Cellular Regulation by Complexes”. 23. “Proteolytic enzymes and their inhibitors”. 24. “Expanded Definition of ‘Trefone’ and Classes of Couplet Cell Interactions”. 25. Note 1 “Extended Trefone Couplets”. Note 2 “Singlet Cells”. 26. “Background of Cancer Research”. [file 1742-4682-11-40-S1.zip › 3484671921331230_add1/3484671921331230_add04.doc]

**Additional File *Four:-***

***4. This Understanding is Limited; What are the Unanswered Questions?***

**The Effects of Growth Factors and other Bioactive Molecules {collectively referred to here as ICMs (Information-Carrying Molecules and Inter-Cellular Messengers)}.**

*The purpose of this File is to highlight the diverse activities of ICMs, which can have effects on cell proliferation etc that are stimulatory, inhibitory or both. It is noted that some of these effects may not reflect a direct action on the cell cycle or on cell division.*

**Stimulation**, often assessed by a substantial increase in DNA synthesis, could be due to increased cell division (CD - symmetric and/or asymmetric) with an increase in the “expected”* proliferation, while hypertrophy could also increase DNA synthesis. The stimulating molecule may directly enhance CD by decreasing the time for CD – it may be a Competence Factor or a Progression Factor for the cell cycle.

Stimulation by a molecule may also occur due to extracellular and other intracellular effects:-

- stimulation of production of an actual stimulator by a neighboring cell

eg. stimulation of proteolytic release (shedding) of a bioactive domain

- destruction/cleavage of an inhibitor, or binding and complex formation of an inhibitor

- prevention of contact inhibition

- prevention of programmed cell death (eg. Apoptosis)

- support of adhesion and/or of migration of cells

Equally, **inhibition,** often assessed by a reduction in DNA synthesis, could be due to prevention of CD with a decrease in “expected”* proliferation, while hypotrophy could also decrease DNA synthesis.

Inhibition by a molecule may also occur due to extracellular and other intracellular effects:

- inhibition of production of an actual stimulator by a neighboring cell

- stimulation of production of an inhibitor (eg. a binding protein) by a neighboring cell

(eg. growth hormone may induce production of an IGFBP which inhibits IGF-I)

- induction of differentiation ie. the molecule may be a differentiation factor ( DF)

- induction of programmed cell death (eg. Apoptosis)

- necrosis (Cytotoxic)

- binding of a stimulator (eg. by a binding protein)

- extracellular matrix interaction or related adhesion and migration effects

Ultimate inhibition would involve cell death, by apoptosis for example, but in most cases of “inhibition” the cell is still viable where the effect is cytostatic.

**[*** A confounding issue is the establishment of what is the baseline for comparison to decide whether a stimulation or inhibition has occurred. For cell growth *in vitro*, what basal medium is used to establish “expected” or normal baseline growth? There are few examples of exactly defined media in terms of the concentrations of all organic substances needed for tissue cell growth to the exclusion of inhibitory substances. Routinely, growth occurs in the presence of a percentage of serum - perhaps 5-10% calf serum - which contains hundreds of organic substances including many ICMs including possible inhibitors.]

Also of relevance is that increased thymidine incorporation may occur without actual cell division, due to the inhibition of cytokinesis and that an increase in DNA synthesis from 1% to 95% could result in only a doubling of cell number. Obviously, cell counting is the most direct and accurate way to detect proliferation. In addition, microscopic observation or other examination should monitor the type of cell growth. Does differentiation or cell death of some cells occur while other cells double their numbers? What proportion of cells undergo symmetric versus asymmetric cell division? These questions should be addressed in monitoring the stimulatory or inhibitory effects of ICMs on cell growth.

**TABLE S1**

**MOLECULES WITH BOTH STIMULATORY AND INHIBITORY ACTIVITIES ON PROLIFERATION**

| **MOLECULE** | **ACTIVITY, CELL TYPE and REFERENCE** | |
| --- | --- | --- |
|  | **Stimulation** | **Inhibition** |
| Insulin-like Growth Factor (IGF-I)^a^ | Numerous types | Lymphocytes ; Smooth muscle ; Wilm’s tumour |
| Insulin-like Growth Factor Binding Proteins | Osteoblast-like bone; Transfected breast cancer (MCF-7) ; Fibroblasts; Adrenocortical tumor Y-1; Ewing sarcoma A673; Neuroblastoma SKN-SHEP; Prostate cancer (DU145) | Breast cancer (Hs578T);  Fibroblasts; Fetal podocytes; Embryonic kidney (HEK-293) ; Prostate cancer LAPC-4 |
| Epidermal Growth Factor (EGF)^a^ | Smooth muscle | Epidermoid carcinoma (A431) |
| Fibroblast Growth Factor-1 (FGF-1)^a^ | Vascular endothelial | Sarcoma Breast carcinoma (MDA-MB-134) |
| FGF Binding protein | Adrenal carcinoma;  Fibroblasts (NIH-3T3) | Swiss 3T3 (biphasic) |
| Growth Hormone (GH)^a^ | Chondrocytes ;  T- lymphocytes | Fibroblasts |
| Hepatocyte Growth Factor (HGF) | Hepatocytes, melanocytes | Various tumours |
| Nerve Growth Factor (NGF) | Adrenal chromaffin | Pheochromocytoma |
| Parathyroid Hormone (PTH) | An osteoblast precursor (TE-85) | A differentiated osteoblastic osteogenic sarcoma (UMR-106) |
| PTH-related Protein (PTHrP) | Aortic vascular smooth muscle (VSMC) (endogenous) ; Intrarenal VSMC (hypertensive rats, exogenous)^37^ | Aortic VSMC (exogenous)^37^; Intrarenal VSMC (hypertensive rats, endogenous)^37^ |
| Platelet-Derived Growth Factor (PDGF)^a^ | Smooth muscle | Mesothelioma |
| Transforming Growth Factor- beta (TGF-β)^b^ | Glioblastoma ;  Kidney epithelial; Colonic epithelial | Glioma; Kidney epithelial; Colorectal cancer |
| Transforming Growth Factor- alpha (TGF-α) | Hepatocytes | Pituitary tumour |
| Tumour Necrosis Factor (TNF)^b^ | Cervical cancer;  Smooth muscle; Fibroblasts | Epithelial cervical |
| Vascular Endothelial Growth Factor (VEGF)^a^ | Large follicle granulosa; Extravillous trophoblast, choriocarcinoma | Small follicle granulosa |
| Amphiregulin | Human fibroblasts and some tumor cells | Carcinoma cell lines |
| Angiotensin II | Smooth muscle | Adrenal glomerulosa |
| Beta amyloid | Glial | Neurons |
| Beta-2 microglobulin | Prostatic carcinoma | Leukemia; Myeloma |
| Endostatin | Hemangioma endothelial | Endothelial |
| Epithelin | Keratinocytes | Epidermal carcinoma |
| Erythropoietin | Bone marrow | Heart fibroblasts |
| Gastrin | Gastric enterochromaffin-like | Colon cancer |
| Insulin | Breast cancer | Melanoma |
| Interferon-γ (IFN-γ) | Megakaryocyte | Hematopoietic progenitors |
| Interleukin 1 alpha (IL-1α) | Cervical cancer  Smooth muscle | Epithelial cervical |
| Macrophage Inflammatory Protein (MIP) | Schwann | Astrocytes;  Haemopoietic stem |
| [Neuregulin-1](http://en.wikipedia.org/wiki/Neuregulin_1) (NRG1), (Heregulin) | Mammary epithelial | Breast cancer |
| Neurotensin | Astrocytic tumor | T cell lymphoma |
| Secreted frizzled-related proteins - sFRPs | Adipocytes; Breast adenocarcinoma | Osteoblasts |
| Transferrin | Mesenchymes;  Muscle | Oligodendroglial |
| Vasoactive intestinal polypeptide (VIP) | Trabecular meshwork | Myocyte |
| Vasopressin | Hypothalamic non-neuronal | Adrenocortical tumor |
| Wnt | Colonic | Adipocytes;  Smooth muscle |
| Acetylcholine | Brain tumors | Schwann |
| γ-Aminobutyric acid (GABA) | Cerebellar granule;  Ventricular neural progenitor | Subventricular neural progenitor |
| ATP | Smooth muscle | Fibroblasts |
| Dopamine | Chinese hamster ovary; Subventricular zone | Pituitary |
| Histamine | Enterochromaffin-like | Pancreatic carcinoma |
| Lysophosphatidic acid (LPA) | Fibroblasts | Myeloma |
| Melatonin | Splenocyte  Myeloma | Breast cancer;  Lymphocytes; Retinal pigment epithelial |
| Retinoic acid | Keratinocytes | Sebocytes |
| Serotonin | Smooth muscle | Blood lymphocytes |
| Adrenocorticotropic hormone (ACTH) | Myogenic;  Melanocytes | Adrenocortical |
| Corticotropin releasing factor (CRF) | Lymphocytes | Corticotrope tumor |
| Cortisol | Cultured costal chondrocytes | Periosteal cells; Lymphocytes |
| Estradiol | Breast cancer (MCF-7) | Smooth muscle |
| Progesterone | Cholangiocytes | Non-small cell lung cancer |
| Sex-Hormone Binding Globulin | Prostate cancer (ALVA-41) | Breast cancer (MCF-7) |
| Kallikrein | Neural stem | Smooth muscle |
| Kallistatin = Kallikrein binding protein | Smooth muscle | Endothelial |

# *This Table lists ICMs along with their ability to stimulate or inhibit the growth of various cell types.*

# Many of these growth factors are well-established as either stimulators^a^ or inhibitors^b^ of proliferation and were discovered by, and named from, this very activity.

# This list is obviously not comprehensive but designed to give some examples of opposing activities in a variety of cells. Vitamins are not listed but, as expected, vitamins or their derivatives will stimulate proliferation but, unexpectedly some vitamins inhibit proliferation. Vitamins A, C and E inhibit cultures of normal and immortalized human oral epithelial cells, while vitamin D3 blocks basal cell carcinoma proliferation.

**DUAL ACTIVITY**

In addition to being either activators or inhibitors in different cells, some ICMs are known to have a dual action activity in the same cell types. This depends on several factors:-

**(i) Concentration of ICM**

EGF is stimulatory at low concentrations for epidermoid carcinoma cells but inhibitory at high concentrations. **S**imilarly, TNF-α is a tumour-regressing agent when administered in very high doses, but at physiological levels, it promotes tumour growth. Also, at low [concentrations](http://www.ncbi.nlm.nih.gov/pubmed?term=Zain j 3133) of thrombin, tumor cell growth is enhanced *in vitro* while at higher concentrations, cell growth is impaired**.**

For mesothelioma cells in vitro, with the PDGF-A/PDGF receptor loop at a high-level, growth is inhibited whereas with PDGF-A expression prevented, growth is stimulated. Again**,** Swiss 3T3 cells are stimulated by low concentrations of FGF binding protein but inhibited at high concentrations.

Low concentrations of PTH (10^-11^ mol/L) stimulate the proliferation of an osteoblastic cell line but high concentration of PTH leads to growth arrest. Low levels produce more ERK1/2 phosphorylation/activation but high levels produce more MKP-1 induction.

This type of response may be viewed as a bell-shaped response curve. The plasminogen activator inhibitor-1 is proangiogenic at physiological levels and antiangiogenic at higher levels, producing a bell-shaped response curve. Splenocytes also show a bell-shaped response curve with growth hormone. Similarly, in retinal axon growth, Ephrin-A2 promoted growth at lower concentrations but produced inhibition at high concentrations.

**(ii) Concentration of Binding Protein**

In some fibroblasts, low levels of α_2-_macroglobulin enhance PDGF growth while high levels are inhibitory in other cell types.

**(iii) Presence of other ICMs**

Serotonin stimulates proliferation of melanocytes in medium without growth factors, but inhibits growth in the presence of growth factors. Similarly, TGF-β acts as a stimulator of growth or as a strong growth inhibitor of an embryonal carcinoma cell line depending on the conditions.

(**iv) Time of Exposure**

The effect of ACTH on cultured fetal rat adrenocortical cells is inhibitory in the first 24 h but during the third day, there is an intense stimulatory effect.

**(v) Developmental State of Cells**

With porcine granulosa cells, VEGF exerts an inhibitory effect on the proliferation in small follicles and a stimulatory one in medium and large follicles.

(vi) **Developmental State of Cells and Concentration of ICMs**

The amyloid beta protein is neurotrophic to undifferentiated hippocampal neurons at low concentrations and neurotoxic to mature neurons at higher concentrations.

The current model attempts to explain this diversity of activities.

**REFERENCES:-**

1.	Greco RM, Ehrlich HP: **Differences in cell division and thymidine incorporation with rat and primate fibroblasts in collagen lattices**. *Tissue Cell* 1992, **24**(6):843-851.

2.	Baserga R: **The path less traveled by**. *Endocrinology* 1997, **138**(6):2217-2218.

3.	Petley T, Graff K, Jiang W, Yang H, Florini J: **Variation among cell types in the signaling pathways by which IGF-I stimulates specific cellular responses**. *Horm Metab Res* 1999, **31**(2-3):70-76.

4.	Daughaday WH: **Growth hormone axis overview--somatomedin hypothesis**. *Pediatr Nephrol* 2000, **14**(7):537-540.

5.	Hunt P, Eardley DD: **Suppressive effects of insulin and insulin-like growth factor-1 (IGF1) on immune responses**. *Journal of Immunology* 1986, **136**(11):3994-3999.

6.	Schriever C, Schmidt A, Breithardt G, Buddecke E: **Human recombinant insulin-like growth factor I and -II stimulate the expression of basic fibroblast growth factor but suppress the division of bovine coronary smooth muscle cells**. *Atherosclerosis* 1996, **122**(2):255-263.

7.	Granerus M, Johannisson A, Ekblom P, Engstrom W: **Insulin-like growth factors I and II induce cell death in Wilms's tumour cells**. *Molecular Pathology* 2001, **54**(1):30-35.

8.	Andress DL, Birnbaum RS: **Human osteoblast-derived insulin-like growth factor (IGF) binding protein-5 stimulates osteoblast mitogenesis and potentiates IGF action**. *Journal of Biological Chemistry* 1992, **267**(31):22467-22472.

9.	Chen JC, Shao ZM, Sheikh MS, Hussain A, LeRoith D, Roberts CT, Jr., Fontana JA: **Insulin-like growth factor-binding protein enhancement of insulin-like growth factor-I (IGF-I)-mediated DNA synthesis and IGF-I binding in a human breast carcinoma cell line**. *J Cell Physiol* 1994, **158**(1):69-78.

10.	Conover CA: **Potentiation of insulin-like growth factor (IGF) action by IGF-binding protein-3: studies of underlying mechanism**. *Endocrinology* 1992, **130**(6):3191-3199.

11.	Hoeflich A, Fettscher O, Lahm H, Blum WF, Kolb HJ, Engelhardt D, Wolf E, Weber MM: **Overexpression of insulin-like growth factor-binding protein-2 results in increased tumorigenic potential in Y-1 adrenocortical tumor cells**. *Cancer Res* 2000, **60**(4):834-838.

12.	Schutt BS, Langkamp M, Rauschnabel U, Ranke MB, Elmlinger MW: **Integrin-mediated action of insulin-like growth factor binding protein-2 in tumor cells**. *J Mol Endocrinol* 2004, **32**(3):859-868.

13.	Russo VC, Schutt BS, Andaloro E, Ymer SI, Hoeflich A, Ranke MB, Bach LA, Werther GA: **Insulin-like growth factor binding protein-2 binding to extracellular matrix plays a critical role in neuroblastoma cell proliferation, migration, and invasion**. *Endocrinology* 2005, **146**(10):4445-4455.

14.	Moore MG, Wetterau LA, Francis MJ, Peehl DM, Cohen P: **Novel stimulatory role for insulin-like growth factor binding protein-2 in prostate cancer cells**. *Int J Cancer* 2003, **105**(1):14-19.

15.	Oh Y, Muller HL, Lamson G, Rosenfeld RG: **Insulin-like growth factor (IGF)-independent action of IGF-binding protein-3 in Hs578T human breast cancer cells. Cell surface binding and growth inhibition**. *Journal of Biological Chemistry* 1993, **268**(20):14964-14971.

16.	Valentinis B, Bhala A, DeAngelis T, Baserga R, Cohen P: **The human insulin-like growth factor (IGF) binding protein-3 inhibits the growth of fibroblasts with a targeted disruption of the IGF-I receptor gene**. *Molecular Endocrinology* 1995, **9**(3):361-367.

17.	Bridgewater DJ, Matsell DG: **Insulin-like growth factor binding protein-2 modulates podocyte mitogenesis**. *Pediatr Nephrol* 2003, **18**(11):1109-1115.

18.	Hoflich A, Lahm H, Blum W, Kolb H, Wolf E: **Insulin-like growth factor-binding protein-2 inhibits proliferation of human embryonic kidney fibroblasts and of IGF-responsive colon carcinoma cell lines**. *FEBS Lett* 1998, **434**(3):329-334.

19.	Yuan QX, McRoberts JA, Lakshmanan J, Yagi H, Hyman PE: **Newborn rabbit gastric smooth muscle cell culture: EGF and TGF-alpha are potent mitogens**. *Journal of Pediatric Gastroenterology & Nutrition* 1993, **17**(2):153-160.

20.	Barnes DW: **Epidermal growth factor inhibits growth of A431 human epidermoid carcinoma in serum-free cell culture**. *J Cell Biol* 1982, **93**(1):1-4.

21.	Smith KB, Losonczy I, Sahai A, Pannerselvam M, Fehnel P, Salomon DS: **Effect of 12-O-tetradecanoylphorbol-13-acetate (TPA) on the growth inhibitory and increased phosphatidylinositol (PI) responses induced by epidermal growth factor (EGF) in A431 cells**. *J Cell Physiol* 1983, **117**(1):91-100.

22.	Gimenez-Gallego G, Rodkey J, Bennett C, Rios-Candelore M, DiSalvo J, Thomas K: **Brain-derived acidic fibroblast growth factor: complete amino acid sequence and homologies**. *Science* 1985, **230**(4732):1385-1388.

23.	Schweigerer L, Neufeld G, Gospodarowicz D: **Basic fibroblast growth factor as a growth inhibitor for cultured human tumor cells**. *J Clin Invest* 1987, **80**(5):1516-1520.

24.	McLeskey SW, Ding IY, Lippman ME, Kern FG: **MDA-MB-134 breast carcinoma cells overexpress fibroblast growth factor (FGF) receptors and are growth-inhibited by FGF ligands**. *Cancer Res* 1994, **54**(2):523-530.

25.	Czubayko F, Smith RV, Chung HC, Wellstein A: **Tumor growth and angiogenesis induced by a secreted binding protein for fibroblast growth factors**. *J Biol Chem* 1994, **269**(45):28243-28248.

26.	Tassi E, Al-Attar A, Aigner A, Swift MR, McDonnell K, Karavanov A, Wellstein A: **Enhancement of fibroblast growth factor (FGF) activity by an FGF-binding protein**. *J Biol Chem* 2001, **276**(43):40247-40253.

27.	Wu DQ, Kan MK, Sato GH, Okamoto T, Sato JD: **Characterization and molecular cloning of a putative binding protein for heparin-binding growth factors**. *J Biol Chem* 1991, **266**(25):16778-16785.

28.	Chen JH, Wang XC, Sato JD: **Bifunctional effects of heparin-binding protein HBp17 on DNA synthesis in cells**. *Cell Biol Int* 2001, **25**(6):567-570.

29.	Madsen K, Friberg U, Roos P, Eden S, Isaksson O: **Growth hormone stimulates the proliferation of cultured chondrocytes from rabbit ear and rat rib growth cartilage**. *Nature* 1983, **304**(5926):545-547.

30.	Postel-Vinay MC, de Mello Coelho V, Gagnerault MC, Dardenne M: **Growth hormone stimulates the proliferation of activated mouse T lymphocytes**. *Endocrinology* 1997, **138**(5):1816-1820.

31.	Wiepz GJ, Houtman JC, Cha D, Bertics PJ: **Growth hormone attenuation of epidermal growth factor-induced mitogenesis**. *J Cell Physiol* 1997, **173**(1):44-53.

32.	Shima N, Itagaki Y, Nagao M, Yasuda H, Morinaga T, Higashio K: **A fibroblast-derived tumor cytotoxic factor/F-TCF (hepatocyte growth factor/HGF) has multiple functions in vitro**. *Cell Biol Int Rep* 1991, **15**(5):397-408.

33.	Lillien LE, Claude P: **Nerve growth factor is a mitogen for cultured chromaffin cells**. *Nature* 1985, **317**(6038):632-634.

34.	Greene LA, Tischler AS: **Establishment of a noradrenergic clonal line of rat adrenal pheochromocytoma cells which respond to nerve growth factor**. *Proc Natl Acad Sci U S A* 1976, **73**(7):2424-2428.

35.	Onishi T, Zhang W, Cao X, Hruska K: **The mitogenic effect of parathyroid hormone is associated with E2F-dependent activation of cyclin-dependent kinase 1 (cdc2) in osteoblast precursors**. *J Bone Miner Res* 1997, **12**(10):1596-1605.

36.	Onishi T, Hruska K: **Expression of p27Kip1 in osteoblast-like cells during differentiation with parathyroid hormone**. *Endocrinology* 1997, **138**(5):1995-2004.

37.	Massfelder T, Dann P, Wu TL, Vasavada R, Helwig JJ, Stewart AF: **Opposing mitogenic and anti-mitogenic actions of parathyroid hormone-related protein in vascular smooth muscle cells: a critical role for nuclear targeting**. *Proc Natl Acad Sci U S A* 1997, **94**(25):13630-13635.

38.	Bornfeldt KE, Campbell JS, Koyama H, Argast GM, Leslie CC, Raines EW, Krebs EG, Ross R: **The mitogen-activated protein kinase pathway can mediate growth inhibition and proliferation in smooth muscle cells. Dependence on the availability of downstream targets**. *J Clin Invest* 1997, **100**(4):875-885.

39.	Metheny-Barlow LJ, Flynn B, van Gijssel HE, Marrogi A, Gerwin BI: **Paradoxical effects of platelet-derived growth factor-A overexpression in malignant mesothelioma. Antiproliferative effects in vitro and tumorigenic stimulation in vivo**. *American Journal of Respiratory Cell & Molecular Biology* 2001, **24**(6):694-702.

40.	Jennings MT, Maciunas RJ, Carver R, Bascom CC, Juneau P, Misulis K, Moses HL: **TGF beta 1 and TGF beta 2 are potential growth regulators for low-grade and malignant gliomas in vitro: evidence in support of an autocrine hypothesis**. *International Journal of Cancer* 1991, **49**(1):129-139.

41.	Holley RW, Baldwin JH, Greenfield S, Armour R: **A growth regulatory factor that can both inhibit and stimulate growth**. *Ciba Foundation Symposium* 1985, **116**:241-252.

42.	Xu Y, Pasche B: **TGF-beta signaling alterations and susceptibility to colorectal cancer**. *Hum Mol Genet* 2007, **16 Spec No 1**(16):R14-20.

43.	Mead JE, Fausto N: **Transforming growth factor alpha may be a physiological regulator of liver regeneration by means of an autocrine mechanism**. *Proc Natl Acad Sci U S A* 1989, **86**(5):1558-1562.

44.	Ramsdell JS: **Transforming growth factor-alpha and -beta are potent and effective inhibitors of GH4 pituitary tumor cell proliferation**. *Endocrinology* 1991, **128**(4):1981-1990.

45.	Woodworth CD, McMullin E, Iglesias M, Plowman GD: **Interleukin 1 alpha and tumor necrosis factor alpha stimulate autocrine amphiregulin expression and proliferation of human papillomavirus-immortalized and carcinoma-derived cervical epithelial cells**. *Proc Natl Acad Sci U S A* 1995, **92**(7):2840-2844.

46.	Owens MW, Grisham MB: **Cytokines increase proliferation of human intestinal smooth muscle cells: possible role in inflammation-induced stricture formation**. *Inflammation* 1993, **17**(4):481-487.

47.	Vilcek J, Palombella VJ, Henriksen-DeStefano D, Swenson C, Feinman R, Hirai M, Tsujimoto M: **Fibroblast growth enhancing activity of tumor necrosis factor and its relationship to other polypeptide growth factors**. *J Exp Med* 1986, **163**(3):632-643.

48.	Grasselli F, Basini G, Bussolati S, Tamanini C: **Effects of VEGF and bFGF on proliferation and production of steroids and nitric oxide in porcine granulosa cells**. *Reprod Domest Anim* 2002, **37**(6):362-368.

49.	Athanassiades A, Hamilton GS, Lala PK: **Vascular endothelial growth factor stimulates proliferation but not migration or invasiveness in human extravillous trophoblast**. *Biol Reprod* 1998, **59**(3):643-654.

50.	Shoyab M, McDonald VL, Bradley JG, Todaro GJ: **Amphiregulin: a bifunctional growth-modulating glycoprotein produced by the phorbol 12-myristate 13-acetate-treated human breast adenocarcinoma cell line MCF-7**. *Proc Natl Acad Sci U S A* 1988, **85**(17):6528-6532.

51.	Bunni MA, Kramarenko, II, Walker L, Raymond JR, Garnovskaya MN: **Role of integrins in angiotensin II-induced proliferation of vascular smooth muscle cells**. *Am J Physiol Cell Physiol* 2011, **300**(3):C647-656.

52.	Otis M, Campbell S, Payet MD, Gallo-Payet N: **Angiotensin II stimulates protein synthesis and inhibits proliferation in primary cultures of rat adrenal glomerulosa cells**. *Endocrinology* 2005, **146**(2):633-642.

53.	Araujo DM, Cotman CW: **Beta-amyloid stimulates glial cells in vitro to produce growth factors that accumulate in senile plaques in Alzheimer's disease**. *Brain Res* 1992, **569**(1):141-145.

54.	Watt JA, Pike CJ, Walencewicz-Wasserman AJ, Cotman CW: **Ultrastructural analysis of beta-amyloid-induced apoptosis in cultured hippocampal neurons**. *Brain Res* 1994, **661**(1-2):147-156.

55.	Rowley DR, Dang TD, McBride L, Gerdes MJ, Lu B, Larsen M: **Beta-2 microglobulin is mitogenic to PC-3 prostatic carcinoma cells and antagonistic to transforming growth factor beta 1 action**. *Cancer Res* 1995, **55**(4):781-786.

56.	Mori M, Terui Y, Ikeda M, Tomizuka H, Uwai M, Kasahara T, Kubota N, Itoh T, Mishima Y, Douzono-Tanaka M *et al*: **Beta(2)-microglobulin identified as an apoptosis-inducing factor and its characterization**. *Blood* 1999, **94**(8):2744-2753.

57.	Min R, Li Z, Epstein J, Barlogie B, Yi Q: **Beta(2)-microglobulin as a negative growth regulator of myeloma cells**. *Br J Haematol* 2002, **118**(2):495-505.

58.	Boye E, Yu Y, Paranya G, Mulliken JB, Olsen BR, Bischoff J: **Clonality and altered behavior of endothelial cells from hemangiomas**. *J Clin Invest* 2001, **107**(6):745-752.

59.	Folkman J: **Antiangiogenesis in cancer therapy--endostatin and its mechanisms of action**. *Exp Cell Res* 2006, **312**(5):594-607.

60.	Shoyab M, McDonald VL, Byles C, Todaro GJ, Plowman GD: **Epithelins 1 and 2: isolation and characterization of two cysteine-rich growth-modulating proteins**. *Proc Natl Acad Sci U S A* 1990, **87**(20):7912-7916.

61.	Jacobs K, Shoemaker C, Rudersdorf R, Neill SD, Kaufman RJ, Mufson A, Seehra J, Jones SS, Hewick R, Fritsch EF *et al*: **Isolation and characterization of genomic and cDNA clones of human erythropoietin**. *Nature* 1985, **313**(6005):806-810.

62.	Wen Y, Zhang XJ, Ma YX, Xu XJ, Hong LF, Lu ZH: **Erythropoietin attenuates hypertrophy of neonatal rat cardiac myocytes induced by angiotensin-II in vitro**. *Scand J Clin Lab Invest* 2009, **69**(4):518-525.

63.	Prinz C, Scott DR, Hurwitz D, Helander HF, Sachs G: **Gastrin effects on isolated rat enterochromaffin-like cells in primary culture**. *Am J Physiol* 1994, **267**(4 Pt 1):G663-675.

64.	Song LJ, Liu RJ, Zeng Z, Alper SL, Cui HJ, Lu Y, Zheng L, Yan ZW, Fu GH: **Gastrin inhibits a novel, pathological colon cancer signaling pathway involving EGR1, AE2, and P-ERK**. *J Mol Med (Berl)* 2012, **90**(6):707-718.

65.	Gliozzo B, Sung CK, Scalia P, Papa V, Frasca F, Sciacca L, Giorgino F, Milazzo G, Goldfine ID, Vigneri R *et al*: **Insulin-stimulated cell growth in insulin receptor substrate-1-deficient ZR-75-1 cells is mediated by a phosphatidylinositol-3-kinase-independent pathway**. *Journal of Cellular Biochemistry* 1998, **70**(2):268-280.

66.	Haring HU, White MF, Kahn CR, Kasuga M, Lauris V, Fleischmann R, Murray M, Pawelek J: **Abnormality of insulin binding and receptor phosphorylation in an insulin-resistant melanoma cell line**. *Journal of Cell Biology* 1984, **99**(3):900-908.

67.	Shiohara M, Koike K, Nakahata T, Komiyama A: **Hematopoietic progenitors and synergism of interferon-gamma and stem cell factor**. *Leuk Lymphoma* 1994, **14**(3-4):203-211.

68.	Khan S, Wigley C: **Different effects of a macrophage cytokine on proliferation in astrocytes and Schwann cells**. *Neuroreport* 1994, **5**(11):1381-1385.

69.	Graham GJ, Wright EG, Hewick R, Wolpe SD, Wilkie NM, Donaldson D, Lorimore S, Pragnell IB: **Identification and characterization of an inhibitor of haemopoietic stem cell proliferation**. *Nature* 1990, **344**(6265):442-444.

70.	Marte BM, Jeschke M, Graus-Porta D, Taverna D, Hofer P, Groner B, Yarden Y, Hynes NE: **Neu differentiation factor/heregulin modulates growth and differentiation of HC11 mammary epithelial cells**. *Mol Endocrinol* 1995, **9**(1):14-23.

71.	Daly JM, Jannot CB, Beerli RR, Graus-Porta D, Maurer FG, Hynes NE: **Neu differentiation factor induces ErbB2 down-regulation and apoptosis of ErbB2-overexpressing breast tumor cells**. *Cancer Res* 1997, **57**(17):3804-3811.

72.	Camby I, Salmon I, Bourdel E, Nagy N, Danguy A, Brotchi J, Pasteels JL, Martinez J, Kiss R: **Neurotensin-mediated effects on astrocytic tumor cell proliferation**. *Neuropeptides* 1996, **30**(2):133-139.

73.	Ramez M, Bagot M, Nikolova M, Boumsell L, Vita N, Chalon P, Caput D, Ferrara P, Bensussan A: **Functional characterization of neurotensin receptors in human cutaneous T cell lymphoma malignant lymphocytes**. *J Invest Dermatol* 2001, **117**(3):687-693.

74.	Christodoulides C, Lagathu C, Sethi JK, Vidal-Puig A: **Adipogenesis and WNT signalling**. *Trends Endocrinol Metab* 2009, **20**(1):16-24.

75.	Melkonyan HS, Chang WC, Shapiro JP, Mahadevappa M, Fitzpatrick PA, Kiefer MC, Tomei LD, Umansky SR: **SARPs: a family of secreted apoptosis-related proteins**. *Proc Natl Acad Sci U S A* 1997, **94**(25):13636-13641.

76.	Chung YS, Baylink DJ, Srivastava AK, Amaar Y, Tapia B, Kasukawa Y, Mohan S: **Effects of secreted frizzled-related protein 3 on osteoblasts in vitro**. *J Bone Miner Res* 2004, **19**(9):1395-1402.

77.	Ekblom P, Thesleff I, Saxen L, Miettinen A, Timpl R: **Transferrin as a fetal growth factor: acquisition of responsiveness related to embryonic induction**. *Proc Natl Acad Sci U S A* 1983, **80**(9):2651-2655.

78.	Beach RL, Popiela H, Festoff BW: **The identification of neurotrophic factor as a transferrin**. *FEBS Lett* 1983, **156**(1):151-156.

79.	Garcia C, Paez P, Davio C, Soto EF, Pasquini JM: **Apotransferrin induces cAMP/CREB pathway and cell cycle exit in immature oligodendroglial cells**. *J Neurosci Res* 2004, **78**(3):338-346.

80.	Koh SW, Yeh TH, Morris SM, Leffler M, Higginbotham EJ, Brenneman DE, Yue BY: **Vasoactive intestinal peptide stimulation of human trabecular meshwork cell growth**. *Investigative Ophthalmology & Visual Science* 1997, **38**(13):2781-2789.

81.	van Assche G, Depoortere I, de Vos R, Geboes K, Janssens JJ, Collins SM, Peeters TL: **Region-specific antiproliferative effect of VIP and PACAP-(1-38) on rabbit enteric smooth muscle**. *American Journal of Physiology* 1999, **276**(1 Pt 1):G303-310.

82.	Worley RT, Pickering BT: **Non-neuronal cells of rat hypothalamus in dissociated cell culture. Evidence that neurophysin modulates growth and DNA synthesis of non-neuronal cells**. *Cell Tissue Res* 1984, **237**(1):161-168.

83.	Schwindt TT, Forti FL, Juliano MA, Juliano L, Armelin HA: **Arginine vasopressin inhibition of cyclin D1 gene expression blocks the cell cycle and cell proliferation in the mouse Y1 adrenocortical tumor cell line**. *Biochemistry* 2003, **42**(7):2116-2121.

84.	Peifer M, Polakis P: **Wnt signaling in oncogenesis and embryogenesis--a look outside the nucleus**. *Science* 2000, **287**(5458):1606-1609.

85.	Ross SE, Erickson RL, Gerin I, DeRose PM, Bajnok L, Longo KA, Misek DE, Kuick R, Hanash SM, Atkins KB *et al*: **Microarray analyses during adipogenesis: understanding the effects of Wnt signaling on adipogenesis and the roles of liver X receptor alpha in adipocyte metabolism**. *Mol Cell Biol* 2002, **22**(16):5989-5999.

86.	Yu XM, Wang L, Li JF, Liu J, Li J, Wang W, Wang J, Wang C: **Wnt5a inhibits hypoxia-induced pulmonary arterial smooth muscle cell proliferation by downregulation of beta-catenin**. *Am J Physiol Lung Cell Mol Physiol* 2012.

87.	Gurwitz D, Razon N, Sokolovsky M, Soreq H: **Expression of muscarinic binding sites in primary human brain tumors**. *Brain Res* 1984, **316**(1):61-70.

88.	Loreti S, Ricordy R, Egle De Stefano M, Augusti-Tocco G, Maria Tata A: **Acetylcholine inhibits cell cycle progression in rat Schwann cells by activation of the M2 receptor subtype**. *Neuron Glia Biol* 2007, **3**(4):269-279.

89.	Fiszman ML, Borodinsky LN, Neale JH: **GABA induces proliferation of immature cerebellar granule cells grown in vitro**. *Brain Res Dev Brain Res* 1999, **115**(1):1-8.

90.	Haydar TF, Wang F, Schwartz ML, Rakic P: **Differential modulation of proliferation in the neocortical ventricular and subventricular zones**. *J Neurosci* 2000, **20**(15):5764-5774.

91.	Erlinge D: **Extracellular ATP: a growth factor for vascular smooth muscle cells**. *Gen Pharmacol* 1998, **31**(1):1-8.

92.	Katayama B, Sakaguchi M, Li JW, Pu H, Inoue Y, Namba M: **ATP inhibition of proliferation of immortalized human fibroblasts is greater than that of normal human diploid fibroblasts**. *Int J Mol Med* 1998, **2**(5):603-606.

93.	Lajiness ME, Chio CL, Huff RM: **D2 dopamine receptor stimulation of mitogenesis in transfected Chinese hamster ovary cells: relationship to dopamine stimulation of tyrosine phosphorylations**. *J Pharmacol Exp Ther* 1993, **267**(3):1573-1581.

94.	Coronas V, Bantubungi K, Fombonne J, Krantic S, Schiffmann SN, Roger M: **Dopamine D3 receptor stimulation promotes the proliferation of cells derived from the post-natal subventricular zone**. *J Neurochem* 2004, **91**(6):1292-1301.

95.	Carretero J, Vazquez RJ, Santos M, Cacicedo L, Rubio M, Sanchez-Franco F, Vazquez R: **Dopamine inhibits in vitro release of VIP and proliferation of VIP-immunoreactive pituitary cells**. *Neuropeptides* 1996, **30**(1):81-86.

96.	Bakke I, Qvigstad G, Brenna E, Sandvik AK, Waldum HL: **Gastrin has a specific proliferative effect on the rat enterochromaffin-like cell, but not on the parietal cell: a study by elutriation centrifugation**. *Acta Physiol Scand* 2000, **169**(1):29-37.

97.	Cricco G, Martin G, Labombarda F, Cocca C, Bergoc R, Rivera E: **Human pancreatic carcinoma cell line Panc-I and the role of histamine in growth regulation**. *Inflamm Res* 2000, **49 Suppl 1**:S68-69.

98.	van Corven EJ, Groenink A, Jalink K, Eichholtz T, Moolenaar WH: **Lysophosphatidate-induced cell proliferation: identification and dissection of signaling pathways mediated by G proteins**. *Cell* 1989, **59**(1):45-54.

99.	Tigyi G, Dyer DL, Miledi R: **Lysophosphatidic acid possesses dual action in cell proliferation**. *Proc Natl Acad Sci U S A* 1994, **91**(5):1908-1912.

100.	Markowska M, Mrozkowiak A, Skwarlo-Sonta K: **Influence of melatonin on chicken lymphocytes in vitro: involvement of membrane receptors**. *Neuroendocrinol Lett* 2002, **23 Suppl 1**:67-72.

101.	Persengiev SP, Kyurkchiev S: **Selective effect of melatonin on the proliferation of lymphoid cells**. *Int J Biochem* 1993, **25**(3):441-444.

102.	Liburdy RP, Sloma TR, Sokolic R, Yaswen P: **ELF magnetic fields, breast cancer, and melatonin: 60 Hz fields block melatonin's oncostatic action on ER+ breast cancer cell proliferation**. *J Pineal Res* 1993, **14**(2):89-97.

103.	Yu HS, Hernandez V, Haywood M, Wong CG: **Melatonin inhibits the proliferation of retinal pigment epithelial (RPE) cells in vitro**. *In Vitro Cellular & Developmental Biology Animal* 1993, **29A**(5):415-418.

104.	Gendimenico GJ, Mezick JA: **Pharmacological effects of retinoids on skin cells**. *Skin Pharmacol* 1993, **6 Suppl 1**:24-34.

105.	Fanburg BL, Lee SL: **A new role for an old molecule: serotonin as a mitogen**. *Am J Physiol* 1997, **272**(5 Pt 1):L795-806.

106.	Slauson DO, Walker C, Kristensen F, Wang Y, de Weck AL: **Mechanisms of serotonin-induced lymphocyte proliferation inhibition**. *Cell Immunol* 1984, **84**(2):240-252.

107.	Cossu G, Cusella-De Angelis MG, Senni MI, De Angelis L, Vivarelli E, Vella S, Bouche M, Boitani C, Molinaro M: **Adrenocorticotropin is a specific mitogen for mammalian myogenic cells**. *Dev Biol* 1989, **131**(2):331-336.

108.	Abdel-Malek Z, Swope VB, Suzuki I, Akcali C, Harriger MD, Boyce ST, Urabe K, Hearing VJ: **Mitogenic and melanogenic stimulation of normal human melanocytes by melanotropic peptides**. *Proc Natl Acad Sci U S A* 1995, **92**(5):1789-1793.

109.	Heikkila P, Arola J, Salmi A, Kahri AI: **ACTH-induced c-myc proto-oncogene expression precedes antimitogenic effect during differentiation of fetal rat adrenocortical cells**. *J Endocrinol* 1995, **145**(2):379-385.

110.	McGillis JP, Park A, Rubin-Fletter P, Turck C, Dallman MF, Payan DG: **Stimulation of rat B-lymphocyte proliferation by corticotropin-releasing factor**. *J Neurosci Res* 1989, **23**(3):346-352.

111.	Melzig MF: **Corticotropin releasing factor inhibits proliferation of AtT-20 cells**. *In Vitro Cell Dev Biol Anim* 1994, **30A**(11):741-743.

112.	Takano T, Takigawa M, Suzuki F: **Stimulation by glucocorticoids of the differentiated phenotype of chondrocytes and the proliferation of rabbit costal chondrocytes in culture**. *J Biochem* 1985, **97**(4):1093-1100.

113.	Chyun YS, Kream BE, Raisz LG: **Cortisol decreases bone formation by inhibiting periosteal cell proliferation**. *Endocrinology* 1984, **114**(2):477-480.

114.	Faict D, Ceuppens JL, De Moor P: **Transcortin modulates the effect of cortisol on mitogen-induced lymphocyte proliferation and immunoglobulin production**. *J Steroid Biochem* 1985, **23**(5A):553-555.

115.	Katzenellenbogen BS, Kendra KL, Norman MJ, Berthois Y: **Proliferation, hormonal responsiveness, and estrogen receptor content of MCF-7 human breast cancer cells grown in the short-term and long-term absence of estrogens**. *Cancer Res* 1987, **47**(16):4355-4360.

116.	Dai-Do D, Espinosa E, Liu G, Rabelink TJ, Julmy F, Yang Z, Mahler F, Luscher TF: **17 beta-estradiol inhibits proliferation and migration of human vascular smooth muscle cells: similar effects in cells from postmenopausal females and in males**. *Cardiovasc Res* 1996, **32**(5):980-985.

117.	Ishibashi H, Suzuki T, Suzuki S, Niikawa H, Lu L, Miki Y, Moriya T, Hayashi S, Handa M, Kondo T *et al*: **Progesterone receptor in non-small cell lung cancer--a potent prognostic factor and possible target for endocrine therapy**. *Cancer Res* 2005, **65**(14):6450-6458.

118.	Nakhla AM, Rosner W: **Stimulation of prostate cancer growth by androgens and estrogens through the intermediacy of sex hormone-binding globulin**. *Endocrinology* 1996, **137**(10):4126-4129.

119.	Fortunati N, Fissore F, Fazzari A, Becchis M, Comba A, Catalano MG, Berta L, Frairia R: **Sex steroid binding protein exerts a negative control on estradiol action in MCF-7 cells (human breast cancer) through cyclic adenosine 3',5'-monophosphate and protein kinase A**. *Endocrinology* 1996, **137**(2):686-692.

120.	Kizuki K, Iwadate H, Ookubo R: **Growth-stimulating effect of kallikrein on rat neural stem cells--II. Immunocytochemical analysis and specificity of the enzyme for neural stem cells**. *Yakugaku Zasshi* 2007, **127**(5):919-922.

121.	Murakami H, Yayama K, Miao RQ, Wang C, Chao L, Chao J: **Kallikrein gene delivery inhibits vascular smooth muscle cell growth and neointima formation in the rat artery after balloon angioplasty**. *Hypertension* 1999, **34**(2):164-170.

122.	Chao J, Miao RQ, Chen V, Chen LM, Chao L: **Novel roles of kallistatin, a specific tissue kallikrein inhibitor, in vascular remodeling**. *Biol Chem* 2001, **382**(1):15-21.

123.	Mason B, Ghanee N, Haigh WG, Lee SP, Oda D: **Effect of vitamins A, C and E on normal and HPV-immortalized human oral epithelial cells in culture**. *Anticancer Res* 1999, **19**(6B):5469-5474.

124.	Tang JY, Xiao TZ, Oda Y, Chang KS, Shpall E, Wu A, So PL, Hebert J, Bikle D, Epstein EH, Jr.: **Vitamin D3 inhibits hedgehog signaling and proliferation in murine Basal cell carcinomas**. *Cancer Prev Res (Phila)* 2011, **4**(5):744-751.

125.	Chajry N, Martin PM, Pages G, Cochet C, Afdel K, Berthois Y: **Relationship between the MAP kinase activity and the dual effect of EGF on A431 cell proliferation**. *Biochem Biophys Res Commun* 1994, **203**(2):984-990.

126.	Balkwill F: **Tumor necrosis factor or tumor promoting factor?** *Cytokine Growth Factor Rev* 2002, **13**(2):135-141.

127.	Anderson GM, Nakada MT, DeWitte M: **Tumor necrosis factor-alpha in the pathogenesis and treatment of cancer**. *Curr Opin Pharmacol* 2004, **4**(4):314-320.

128.	Zain J, Huang YQ, Feng X, Nierodzik ML, Li JJ, Karpatkin S: **Concentration-dependent dual effect of thrombin on impaired growth/apoptosis or mitogenesis in tumor cells**. *Blood* 2000, **95**(10):3133-3138.

129.	Qin L, Qiu P, Wang L, Li X, Swarthout JT, Soteropoulos P, Tolias P, Partridge NC: **Gene expression profiles and transcription factors involved in parathyroid hormone signaling in osteoblasts revealed by microarray and bioinformatics**. *J Biol Chem* 2003, **278**(22):19723-19731.

130.	Devy L, Blacher S, Grignet-Debrus C, Bajou K, Masson V, Gerard RD, Gils A, Carmeliet G, Carmeliet P, Declerck PJ *et al*: **The pro- or antiangiogenic effect of plasminogen activator inhibitor 1 is dose dependent**. *FASEB Journal* 2002, **16**(2):147-154.

131.	Hansen MJ, Dallal GE, Flanagan JG: **Retinal axon response to ephrin-as shows a graded, concentration-dependent transition from growth promotion to inhibition**. *Neuron* 2004, **42**(5):717-730.

132.	Bonner JC, Badgett A, Osornio-Vargas AR, Hoffman M, Brody AR: **PDGF-stimulated fibroblast proliferation is enhanced synergistically by receptor-recognized alpha 2-macroglobulin**. *J Cell Physiol* 1990, **145**(1):1-8.

133.	Slominski A, Pisarchik A, Zbytek B, Tobin DJ, Kauser S, Wortsman J: **Functional activity of serotoninergic and melatoninergic systems expressed in the skin**. *J Cell Physiol* 2003, **196**(1):144-153.

134.	van Zoelen EJ, Koornneef I, Holthuis JC, Ward-van Oostwaard TM, Feijen A, de Poorter TL, Mummery CL, van Buul-Offers SC: **Production of insulin-like growth factors, platelet-derived growth factor, and transforming growth factors and their role in the density-dependent growth regulation of a differentiated embryonal carcinoma cell line**. *Endocrinology* 1989, **124**(4):2029-2041.

135.	Arola J, Heikkila P, Voutilainen R, Kahri AI: **Role of adenylate cyclase-cyclic AMP-dependent signal transduction in the ACTH-induced biphasic growth effect of rat adrenocortical cells in primary culture**. *J Endocrinol* 1993, **139**(3):451-461.

136.	Yankner BA, Duffy LK, Kirschner DA: **Neurotrophic and neurotoxic effects of amyloid beta protein: reversal by tachykinin neuropeptides**. *Science* 1990, **250**(4978):279-282.
