# Supplementary material for: A model to explain specific cellular communications and cellular harmony:- a hypothesis of coupled cells and interactive coupling molecules
Source: Theor Biol Med Model. 2014 Sep 14;11:40. doi: 10.1186/1742-4682-11-40 (PMC4237941; doi:10.1186/1742-4682-11-40)
Supplement: Additional file 1 — 1. “Terminology”. 2. “Change in Cellular Characteristics”. 3. Note 1 “The Roles of SCD versus ACD”. Note 2. “Categories of SCD and ACD”. Note 3 “Further Examples of these AsCD Categories”. Note 4 “Change of differentiation type.”. 4. “The Effects of Growth Factors and other Bioactive Molecules 5. Note 1, “Additional Information on the Definition of a Trefone”. Note 2 “Clarification on Ligand and Binding Protein Interaction”. Note 3 “Cell Couplets – AsCD and the a-Cell and i-Cell”. Note 4 “Drosophila – Potential Trefones and ACD”. 6. “Calculated Levels of Trefones and TCC, with Cellular Actions”. 7. “Receptors for the Six IGFBPs and Cellular Effects”. 8. Note 1 “Extracellular Binding Proteins/ Soluble Receptors”. Note 2 “Cell Receptors for Binding Proteins/Soluble Receptors”. 9. Note 1. “Further Evidence for a Receptor for TCC”. Note 2 “Problems with Cross-linking Experiments”. 10. “Evidence for Binary Receptors aside from the IGF System”. 11. “Evidence for Two Receptors for a Particular Trefone, outside the IGF System”. 12. Note 1 “Mechanisms of Nuclear Localization”. Note 2 “NL of IGFBPs”. Note 3 “NL of Potential Trefones other than the IGF System”. 13. “Evidence Supporting the Existence of a-Cells and i-Cells”. 14. “Candidates with mainly a-Cell-Type or i-Cell-Type Characteristics”. 15. “Interacting Cells and Trefones not associated with the IGF System”. 16. “Heterogeneity/Variability of Cells in Culture.” 17. “Cell lines are not Typical of Normal Cells”. 18. “Cell Receptors and Cell Markers”. 19. “Defined Couplet Cells for Insulin and Glucagon; Gastrin and Histamine”. 20. “Potential Couplet Trefones”. 21. “Further Examples of Potential Trefone and Cell Couplets”. 22. “Examples of Cellular Regulation by Complexes”. 23. “Proteolytic enzymes and their inhibitors”. 24. “Expanded Definition of ‘Trefone’ and Classes of Couplet Cell Interactions”. 25. Note 1 “Extended Trefone Couplets”. Note 2 “Singlet Cells”. 26. “Background of Cancer Research”. [file 1742-4682-11-40-S1.zip › 3484671921331230_add1/3484671921331230_add05.doc]

**Additional File *Five:-***

***1. A Description of the Essentials of the Basic Model – a Balance of Trefones and Couplet Cells***

**1. (a) Primary Couplet Cells and Trefone Couplets – the CTC Model with the IGF system as a prime example**

***Note 1. Additional Information on the Definition of a Trefone***

*The purpose of this Note is to clarify the derivation and definition of a* ***Trefone.***

The word **Trefone** is derived from Trephone (Gr. Trephein to feed) which is defined as “a hypothetical substance manufactured by certain cells of the body and used by other cells in the building up of their protoplasm”; Reference:- *Dorland’s Illustrated Medical Dictionary*, 24^th^ edn, 1965. W.B.Saunders Company. Its definition is extended here to reflect primarily more than just building up the living substance or protoplasm (ie. cytoplasm plus nucleoplasm) of a single cell. A Trefone is then used here to refer to an ICM (Information-Carrying Molecule and Inter-Cellular Messenger) but its message is especially associated with cell fate decisions of the nucleoplasm – cell division (symmetric or asymmetric), differentiation, apoptosis etc. It will also affect decisions of the cytoplasm – the relative catabolic and anabolic rates of reaction for sustenance of a single cell. The same substance may have one function in some cells but be one of a Trefone pair in other couplet cells. A neurotransmitter (dopamine) would not be a Trefone in its activity to transfer an excitatory or inhibitory message from one cell to another but it could be a Trefone in a specific couplet of cells. A specific hormone (eg. insulin, glucagon) may stimulate many cells but not be a Trefone for those cells although it may be one of a Trefone pair for a specific couplet of cells in the pancreas. A specific Trefone will be critical for some cells but the ICM may be irrelevant for others, while an ICM other than a Trefone can stimulate the cell’s metabolic activity and indirectly stimulate cell-life decisions.

Note that some Trefones may be defined as having an intracrine action An intracrine hormone has been defined as follows:-“For the purpose of this analysis, a factor/hormone will be deemed an intracrine if it is a peptide that has been reported (a) to be found in the extracellular space and to bind to cell surface receptors with the production of a biological effect and (b) to be associated with one or more intracellular organelles not involved in the secretory pathway”. Trefones are similar in that they are likely involved in the nucleus and thus have an intracrine-like function but they are not necessarily peptides and may also be involved in the secretory pathway. Importantly, each Trefone has a couplet Trefone with the pair derived from couplet cells.

***Note 2. Clarification on Ligand and Binding Protein Interaction; the TCC***

*The purpose of this Note is to expand on the properties of a ligand and its binding protein.*

Note that in a complex of ligand and so-called “binding protein” (BP), the interaction is mutual, with each molecule likely to participate equally in the binding partnership of one to the other, via coupling non-covalent chemical bonds. The BP is so called only because it is the larger of the two polypeptide molecules in the couplet but there is then a false connotation of the BP “capturing” the smaller ligand, yet the smaller molecule may be using the larger one to hide from predators! In the equilibrium between the Complex and the Free participants, the “on” association rate and the “off” dissociation rate are determined by the molecules and atoms in contact, there being equal, bidirectional electrostatic and polar interactions and hydrogen bonds holding the BP and ligand in the complex once formed. It should be noted that some “growth factors” have high molecular weights (of up to 40kDa) more expected of a BP. On the other hand, it is possible that induced spatial and chemical changes could occur to bias the actual association/pull or dissociation/push forces towards one participant, perhaps the larger one. However, with the evidence that BPs have biological properties independent of their ligands, including the existence of their own receptors, then the ligand and BP should be considered equals in their Trefone activities. Herein, the ligand and BP are considered as potential Trefones, as equals, and referred to as couplet molecules that form a “Trefone Couplet Complex”, a TCC.

The Association Constant for the couplet molecules IGF-I and IGFBPs depends on the type of IGFBP but is usually between 5x10^9^ and 1.7x10^10^ . The concentration of Free IGF-I in serum has been measured to be 1% or less of the total IGF-I. In other words, the concentration of TCC is nearly 100 times the concentration of Free IGF-I.

***Note 3. Cell Couplets – AsCD and the a-Cell and i-Cell***

*The purpose of this Note is to (i) explain the derivation of the “a” and “i” abbreviations (ii) clarify the format used in diagrams to distinguish these specific differentiated cells (the a-Cell and the i-Cell) and (iii) describe more options for their cell division involving dedifferentiation and transdifferentiation.*

***(i) The “a” and the “i” of Trefone and Cell Couplets.***

The “a” is taken from the Chinese word “Yang” while the “i” is taken from the opposing word “Yin”. The idea of harmony and balance, derived from these two tendencies which oppose one another in their actions and which may swing out of equilibrium, is the basis of Yang and Yin theory or philosophy. In this theory, while all things and phenomena in the universe contain these two opposite aspects, they are at the same time, both interdependent and in conflict; they are complementary but yet in opposition to each other. The Yin and Yang of the control of cell growth has been addressed previously with reference to malignant tumor development where loss of growth inhibition is considered as an alternative cause to a gain in stimulation**.** “When designing the cell, Nature generally built in a system of checks and balances that allow cellular activities to be closely regulated by opposing stimulatory and inhibitory influences.” This source also refers to Ruth Sager of Harvard's Dana-Farber Cancer Institute and her reference to the "yin-yang theory of cancer"- meaning “that the loss of inhibitory responses may be just as important for unleashing the malignant potential of cells as is activation of the stimulatory forces.”

The letters are herein used to denote normal cells and normal molecules. Note that the “a” and “i” initials are not to be taken as meaning “activation” and “inhibition” as either an “a”- Trefone or an “i”-Trefone could be involved in either process.

A source quotes Sir Edward Schafer (the scientist who coined the name “Insuline” eight years before its discovery by Banting and Best in 1921), from his book The Endocrine Organs (1916):- Schafer avoided the term “hormone” and used the terms “autacoid” for a substance with excitatory action and “chalone” for one with inhibitory action; Schafer went on to describe how ‘insuline’ had both excitatory and inhibitory actions!

The term Trefone then encapsulates both autacoid and chalone but a Trefone is not necessarily an activator/stimulator or an inhibitor in that it depends on context. A Trefone may have either property depending on the amount of the other Trefone and the amount of the complex (TCC) formed.

***(ii) The format used in diagrams.***

The Couplets Cells that produce the Trefones are special differentiated cells - the a-Cell and the i-Cell. Their formation is via AsCD of the precursor cell, the original or “o”Cell. Figure 1 compares the generic format of presentation as used in **Figure 1** of the **Additional File *Three*, Note 2**, with the more specific format now used for these couplet cells in the CTC model.

**Figure 1. Asymmetrical cell division with two formats.**

***(a)*** *This is the format from* **Additional File *Three, Note 2, Figure 1.***

***(b)*** *This is the format used in the “Coupled Trefones and Cells” (CTC) model.*

In (a) the AsCD of cell C produces two differentiated cells (C/D_1_ and CD_2_) in the generic format from Figure 4 above. In (b), in an equivalent way, the “o”Cell produces the a-Cell plus the i-Cell in the CTC format.

***(iii) More options for cell division.***

The CTC model leads to further consideration of other relevant options aside from those listed in **Figure 1** of **Additional File *Three*, Note 2**. This has been discussed in a general way in **Additional File *Three*, Note 2** and is discussed here specifically for the a-Cell and i-Cell.

By cell division (CD), an a-Cell could produce all of the options of Figure 3. For example, it could produce two a-Cells or two newly differentiated cells, with further cell progeny as the cells move forward or down a lineage of cells. But, in addition, there are other possible progeny from the cell division of an a-Cell as listed in Table 1.

**TABLE S**1. Additional Categories of Cell Division.

| **Type** | **Progeny of Cell Division of an a-Cell** | **Process of Cell Division** |
| --- | --- | --- |
| **(i)** | 2x o-Cell | A dedifferentiation (DD) type of SCD |
| **(ii)** | o-Cell + a-Cell | A semi-DD type of AsCD with Self-Renewal (SR) |
| **(iii)** | o-Cell + i-Cell | A semi-DD type of AsCD with Transdifferentiation (TD) |
| **(iv)** | i-Cell + a-Cell | A semi-TD type of AsCD with SR |
| **(v)** | 2x i-Cell | A TD type of SCD (TD/SCD) |

*This Table describes the types of cells possibly derived from the cell division of an a-Cell and the processes involved in various differentiation modes.*

Within these options, the formation of an i-Cell from an a-Cell is considered a trans-differentiation. This is not considered a major step; it seems very feasible as the prior CD of the o-Cell is the fork in the road to two new lineages so that the “cross-over” is less complicated genetically than if the cells were further down the lineage branch or root. It is noted that the same result (a-Cells → i-Cells) could be achieved by a Type (i) DD of an a-Cell back to two o-Cells, followed by an SCD of o-Cells to i-Cells. Equivalent options exist for the i-Cell and, of course, exist for any differentiated cell. To include these options, Diagram (b) in **Figure 1** above would need bidirectional arrows between the o-Cell and its progeny, plus a new double arrow between the a-Cell and the i-Cell.

The potential to change from an a-Cell to an i-Cell is an important aspect for understanding what happens in a cell culture. (This is further identified in **Additional File** ***Six***.) Within a culture, it is usually assumed that SCD occurs to double the number of just one cell type, unless obvious transformation occurs. Within the CTC model, it is proposed that the culture is initially not 100% of just one cell type. Both a-Cells and i-Cells are present in various proportions depending on the conditions and the relative numbers will change depending on any change in conditions including self-produced changes with age or with inflicted changes eg. by additives. The model allows a-Cells to change easily to i-Cells or back to o-Cells. If the i-Cells numbers are reduced, then to maintain balance, an AsCD of the semi-transdifferentiation type {Table 1, Type (iv)}, by which an a-Cell would produce an a-Cell plus an i-Cell, could produce the balance of cells required. If the i-Cells are much reduced, an SCD of the transdifferentiation type {Table 1, Type (v)}, by which an a-Cell would produce two i-Cells, could produce the balance of cells required. The i-Cells could double by SCD as required for balance.

Preimplantation genetic diagnosis typically involves the removal of one blastomere from the 8-cell embryo. If that blastomere had a specific function, then it would have to be replaced. This could occur if the other cell progeny from the cell division of the particular parent blastomere of the 4-cell embryo, divided by a Type (iv) mechanism with semi-transdifferentiation and with self-renewal.

**Note 4.**

***Drosophila – Potential Trefones and Asymmetric Cell Division.***

*The purpose of this note is to examine if a system in* Drosophila *conforms to the CTC model.*

In the *Drosophila* embryonic ectoderm, embryo, there is a single-cell-wide stripe in each anterior parasegment (PS) which expresses a secreted wingless (wg) protein which is dependent on a signal from the adjacent, posterior cells, which secrete hedgehog (hh). Each PS is divided into cells that are competent to secrete either wg or hh, but not both. This physical situation could be easily explained by a prior series of asymmetric cell divisions in a plane. Each progeny cell produces a signal which stimulates the expression of the other in the adjacent cell. A reciprocal paracrine signalling system could operate in these secreting cells as they become mutually dependent. Figure 1 show the suggested interactions.

**Figure 1. A suggested model for interaction between two cell types of *Drosophila* which are bound together.**

*Frizzled is the receptor for the secreted Wg protein and Patched is the receptor for the secreted Hh protein.* *The a-Cell represents an anterior cell and the i-Cell represents a posterior cell of the parasegment.* *The distance between the two is exaggerated by the triple line which indicates intercellular binding.* *The Figure is based on similar models*.

The current model then has Wg and Hh proteins as couplet Trefones produced by couplet cells of the anterior and posterior of parasegments of *Drosophila*. Further, the cells would have been derived from AsCD of earlier embryonic cells.

**REFERENCES:-**

1.	Re RN: **Toward a theory of intracrine hormone action**. *Regul Pept* 2002, **106**(1-3):1-6.

2.	Loddick SA, Liu XJ, Lu ZX, Liu C, Behan DP, Chalmers DC, Foster AC, Vale WW, Ling N, De Souza EB: **Displacement of insulin-like growth factors from their binding proteins as a potential treatment for stroke**. *Proc Natl Acad Sci U S A* 1998, **95**(4):1894-1898.

3.	Takada M, Nakanome H, Kishida M, Hirose S, Hasegawa T, Hasegawa Y: **Measurement of free insulin-like growth factor-I using immunoradiometric assay**. *J Immunoassay* 1994, **15**(3):263-276.

4.	Frystyk J, Skjaerbaek C, Dinesen B, Orskov H: **Free insulin-like growth factors (IGF-I and IGF-II) in human serum**. *FEBS Lett* 1994, **348**(2):185-191.

5.	Marx JL: **The yin and yang of cell growth control**. *Science* 1986, **232**(4754):1093-1095.

6.	Sonksen P, Sonksen J: **Insulin: understanding its action in health and disease**. *Br J Anaesth* 2000, **85**(1):69-79.

7.	Cadigan KM, Grossniklaus U, Gehring WJ: **Localized expression of sloppy paired protein maintains the polarity of Drosophila parasegments**. *Genes Dev* 1994, **8**(8):899-913.

8.	Hooper JE: **Distinct pathways for autocrine and paracrine Wingless signalling in Drosophila embryos**. *Nature* 1994, **372**(6505):461-464.

9.	Yoffe KB, Manoukian AS, Wilder EL, Brand AH, Perrimon N: **Evidence for engrailed-independent wingless autoregulation in Drosophila**. *Dev Biol* 1995, **170**(2):636-650.

10.	Orsulic S, Peifer M: **Cell-cell signalling: Wingless lands at last**. *Curr Biol* 1996, **6**(11):1363-1367.
