# Supplementary material for: A model to explain specific cellular communications and cellular harmony:- a hypothesis of coupled cells and interactive coupling molecules
Source: Theor Biol Med Model. 2014 Sep 14;11:40. doi: 10.1186/1742-4682-11-40 (PMC4237941; doi:10.1186/1742-4682-11-40)
Supplement: Additional file 1 — 1. “Terminology”. 2. “Change in Cellular Characteristics”. 3. Note 1 “The Roles of SCD versus ACD”. Note 2. “Categories of SCD and ACD”. Note 3 “Further Examples of these AsCD Categories”. Note 4 “Change of differentiation type.”. 4. “The Effects of Growth Factors and other Bioactive Molecules 5. Note 1, “Additional Information on the Definition of a Trefone”. Note 2 “Clarification on Ligand and Binding Protein Interaction”. Note 3 “Cell Couplets – AsCD and the a-Cell and i-Cell”. Note 4 “Drosophila – Potential Trefones and ACD”. 6. “Calculated Levels of Trefones and TCC, with Cellular Actions”. 7. “Receptors for the Six IGFBPs and Cellular Effects”. 8. Note 1 “Extracellular Binding Proteins/ Soluble Receptors”. Note 2 “Cell Receptors for Binding Proteins/Soluble Receptors”. 9. Note 1. “Further Evidence for a Receptor for TCC”. Note 2 “Problems with Cross-linking Experiments”. 10. “Evidence for Binary Receptors aside from the IGF System”. 11. “Evidence for Two Receptors for a Particular Trefone, outside the IGF System”. 12. Note 1 “Mechanisms of Nuclear Localization”. Note 2 “NL of IGFBPs”. Note 3 “NL of Potential Trefones other than the IGF System”. 13. “Evidence Supporting the Existence of a-Cells and i-Cells”. 14. “Candidates with mainly a-Cell-Type or i-Cell-Type Characteristics”. 15. “Interacting Cells and Trefones not associated with the IGF System”. 16. “Heterogeneity/Variability of Cells in Culture.” 17. “Cell lines are not Typical of Normal Cells”. 18. “Cell Receptors and Cell Markers”. 19. “Defined Couplet Cells for Insulin and Glucagon; Gastrin and Histamine”. 20. “Potential Couplet Trefones”. 21. “Further Examples of Potential Trefone and Cell Couplets”. 22. “Examples of Cellular Regulation by Complexes”. 23. “Proteolytic enzymes and their inhibitors”. 24. “Expanded Definition of ‘Trefone’ and Classes of Couplet Cell Interactions”. 25. Note 1 “Extended Trefone Couplets”. Note 2 “Singlet Cells”. 26. “Background of Cancer Research”. [file 1742-4682-11-40-S1.zip › 3484671921331230_add1/3484671921331230_add06.doc]

**Additional File *Six:-***

**(b) The Reason for a Cell’s Need to Measure the Trefone Couplet Complex**

***Calculated Levels of Trefones and TCC, Connected to Associated Cellular Actions.***

*The purpose of this File is to demonstrate the variation in concentrations of Trefones and how these variations connect with cellular responses.*

These calculations are based on an association constant (Ka) where Ka =	[^a^T:^i^T]	for the reversible formation of the complex ^a^T:^i^T from ^a^T and ^i^T	[^a^T] [^i^T]

ie. for the equilibrium ^a^T + ^i^T ⮀ ^a^T:^i^T.

If an assumption is made about the association constant of ^a^T and ^i^T, then at a range of concentrations of Total ^a^T and Total ^i^T, the concentrations of Free ^a^T, Free ^i^T and TCC can be calculated. Here, the equation of the Law of Mass Action was used for calculations assuming an Association Constant of 1x10^10^ which is typical of the binding of IGF-I to IGFBPs.

The interpretation of the message derived from this information and the consequent actions of the a-Cells and i-Cells are suggested in **Table 1.**

**TABLE S1. Calculated Variation in the Concentration of the Trefone-Couplet-Complex and Free Trefones with Change in Concentration of Total Individual Trefones with Possible Cell Action. (Trefone Table with Ka assumed = 1x10^10^)**

| **[TCC]** | **[^a^T ]** | | **[^i^T]** | | **Interpretation of message and**  **cell action*** |
| --- | --- | --- | --- | --- | --- |
|  | ***Total*** | ***Free*** | ***Total*** | ***Free*** |  |
| **Very Low(vLo)**  0.4 | **vLo**  1.0 | 0.6 | **vLo**  1.0 | 0.6 | a-Cells very **under**active - SCD  i-Cells very **under**active - SCD |
| 0.9 | **vLo**  1.0 | #  0.1 | Lo  10 | 9.1 | a-Cells very **under**active - SCD  i-Cells **under**active - AsCD > SCD |
| ~1.0 | **vLo**  1.0 | 0.01 | Me  100 | ~10^2^ | a-Cells very **under**active - SCD  i-Cells duly active – TD/SCD >AsCD |
| ~1.0 | **vLo**  1.0 | 0.001 | Hi  1000 | ~10^3^ | a-Cells very **under**active - SCD  i-Cells **over**active – TD/SCD >APO |
| **Low** (Lo)  7.3 | **Lo**  10 | 2.7 | **Lo**  10 | 2.7 | a-Cells **under**active - SCD  i-Cells **under**active - SCD |
| ~10 | **Lo**  10 | #  0.1 | Me  100 | 90 | a-Cells **under**active - SCD  i-Cells duly active - AsCD |
| ~10 | **Lo**  10 | 0.01 | Hi  1000 | ~10^3^ | a-Cells **under**active - SCD  i-Cells **over**active - APO > TD/SCD |
| ***Medium*** (Me)  90 | **Me**  **100** | **10** | **Me**  **100** | **10** | **Situation balanced**  **- each SCD/QSC** |
| ~100 | **Me**  100 | #  0.1 | Hi  1000 | 900 | a-Cells duly active - SCD/QSC  i-Cells **over**active - APO/QSC |
| **High** (Hi)  ~1000 | **Hi**  1000 | 30 | **Hi**  1000 | 30 | a-Cells **over**active - APO/QSC  i-Cells **over**active - APO/QSC |

*Concentrations x10^10^ eg. “Medium” concentration (100 in the Table)* ***= ~****1x10^-8^ mol/L*

*The calculation is based on an Association Constant (Ka) of 1x10^10^ with set, initial (Total) concentrations of each Trefone which is varied from 1x10^-10^ {Very Low (vLo)} to 1x10^-7^ mol/L {High(Hi)}. An identical Table would apply if [^a^T ] and [^i^T] were reversed in concentrations.*

** For a-Cells and i-Cells, the messages received and the consequent cell action are based on the relative amounts of Free ^i^T and Free ^a^T concentrations (respectively) and on the TCC concentration. Abbreviations used:- SCD = Symmetric Cell Division. AsCD = Asymmetric Cell Division. TD = Transdifferentiation. APO = Apoptosis. QSC = Quiescence.*

The SCD referred to in the Table would normally be SRE {Type A(i) in **Additional File *Three, Note 2, Figure 1***.} by which the a- or i-Cell could double their number. If the i-Cells are deemed *somewhat* deficient, an AsCD of the semi-transdifferentiation type {Type (iv) in **Additional File *Five***, **Table 1**}, by which an a-Cell would produce an a-Cell plus an i-Cell, could produce the balance of cells required. If the i-Cells are deemed *very* deficient, an SCD of the transdifferentiation type {Type (v) in **Additional File *Five***, **Table 1**} by which an a-Cell would produce two i-Cells, could produce the balance of cells required.

Cell action could also include a decision to dedifferentiate to two o-Cells {Type (i) in **Additional File *Five***, **Table 1**} in order to restart the lineage, as the TD/SCD option in the Table above. Overactivity of cells may also initiate a differentiation pathway dependent on the presence/concentration of a specific differentiation factor (DF). Other options, as indicated in **Additional File *Three, Note 2, Figure 1*** and in **Additional File *Five***, **Table 1**, would also be possible depending on the chemical signals in the environment affecting other cell receptors. Changes could occur to produce a more sustainable cell appropriate to the environment eg. in adverse conditions for microbiological systems. Note that “Quiescence” may have various states.

***The cells could be able to make intelligent interpretations of their circumstances from the data in the Table:-***

***Interpretation 1***. When the i-Cell detects a Free [^a^T ] of 0.1 on three occasions (#), its status and response is correctly assessed by the concentration of TCC (0.9, ~10 or ~100) which signals underactive, duly active or overactive status of the i-Cell which has produced low, medium and high levels of total ^i^T, respectively.

***Interpretation 2***. When [^a^T ] is fixed at a vLo level (1x10^-10^ mol/L) and [^i^T] is varied over a range of 1000 {from 1x10^-10^ to 1x10^-7^ (Hi)}, then while [TCC] remains nearly constant at the very low level (0.4 - < 1 x10^-8^, the Free level of [^a^T ] varies over a range of 600 (from 0.6x10^-10^ to 1x10^-13^) and the ratio of the Free Trefones shows an enormously amplified range of 10^6^ (from 1:1 to 1:10^6^). This means that, although the [TCC] has not signalled much of a change in information, because of the fact that an actual complex has been formed, the a-Cells and i-Cells will be exposed to an amplified difference in Free Trefones so to respond more sensitively.

The response described in **Table 1** above, in terms of cell division, is the second possible response; the first response would be to regain equilibrium by an increase or decrease in the metabolic activity of individual cells, prior to resorting to cell division.

This **Table** applies only to sets of normal cells in a tissue or organism, which are coupled by Trefones. Cancers derived from coupled calls and their cell lines may not have appropriate receptors or infrastructure to be able to respond nor able to undergo the many optional cell divisions, so their response will differ from that predicted here. The original, normal cells of the cancer tissue may have undergone an earlier abnormal cell division to produce the abnormally responsive cells.

Overall, the control of the proliferation in normal cells and cell lines can be explained by a double paracrine regulation where growth is controlled by the presence of two Trefones and their receptors on coupled cells, along with the TCC.

**REFERENCE:-**

1.	Loddick SA, Liu XJ, Lu ZX, Liu C, Behan DP, Chalmers DC, Foster AC, Vale WW, Ling N, De Souza EB: **Displacement of insulin-like growth factors from their binding proteins as a potential treatment for stroke**. *Proc Natl Acad Sci U S A* 1998, **95**(4):1894-1898.

2.	Daignan-Fornier B, Sagot I: **Proliferation/quiescence: the controversial "aller-retour"**. *Cell Div* 2011, **6**:10.
