# Supplementary material for: A model to explain specific cellular communications and cellular harmony:- a hypothesis of coupled cells and interactive coupling molecules
Source: Theor Biol Med Model. 2014 Sep 14;11:40. doi: 10.1186/1742-4682-11-40 (PMC4237941; doi:10.1186/1742-4682-11-40)
Supplement: Additional file 1 — 1. “Terminology”. 2. “Change in Cellular Characteristics”. 3. Note 1 “The Roles of SCD versus ACD”. Note 2. “Categories of SCD and ACD”. Note 3 “Further Examples of these AsCD Categories”. Note 4 “Change of differentiation type.”. 4. “The Effects of Growth Factors and other Bioactive Molecules 5. Note 1, “Additional Information on the Definition of a Trefone”. Note 2 “Clarification on Ligand and Binding Protein Interaction”. Note 3 “Cell Couplets – AsCD and the a-Cell and i-Cell”. Note 4 “Drosophila – Potential Trefones and ACD”. 6. “Calculated Levels of Trefones and TCC, with Cellular Actions”. 7. “Receptors for the Six IGFBPs and Cellular Effects”. 8. Note 1 “Extracellular Binding Proteins/ Soluble Receptors”. Note 2 “Cell Receptors for Binding Proteins/Soluble Receptors”. 9. Note 1. “Further Evidence for a Receptor for TCC”. Note 2 “Problems with Cross-linking Experiments”. 10. “Evidence for Binary Receptors aside from the IGF System”. 11. “Evidence for Two Receptors for a Particular Trefone, outside the IGF System”. 12. Note 1 “Mechanisms of Nuclear Localization”. Note 2 “NL of IGFBPs”. Note 3 “NL of Potential Trefones other than the IGF System”. 13. “Evidence Supporting the Existence of a-Cells and i-Cells”. 14. “Candidates with mainly a-Cell-Type or i-Cell-Type Characteristics”. 15. “Interacting Cells and Trefones not associated with the IGF System”. 16. “Heterogeneity/Variability of Cells in Culture.” 17. “Cell lines are not Typical of Normal Cells”. 18. “Cell Receptors and Cell Markers”. 19. “Defined Couplet Cells for Insulin and Glucagon; Gastrin and Histamine”. 20. “Potential Couplet Trefones”. 21. “Further Examples of Potential Trefone and Cell Couplets”. 22. “Examples of Cellular Regulation by Complexes”. 23. “Proteolytic enzymes and their inhibitors”. 24. “Expanded Definition of ‘Trefone’ and Classes of Couplet Cell Interactions”. 25. Note 1 “Extended Trefone Couplets”. Note 2 “Singlet Cells”. 26. “Background of Cancer Research”. [file 1742-4682-11-40-S1.zip › 3484671921331230_add1/3484671921331230_add07.doc]

**Additional File** ***Seven***

***(ii)* Receptors for IGF Binding Proteins/Trefones**

***A Listing of Receptors for the Six IGFBPs, with Associated Cellular Effects especially of Proliferation***

*The purpose of this File is to demonstrate the existence of receptors for each IGFBP to thus support the concept that these BPs are Trefones, just as IGF-I and IGF-II are.*

The search for receptors for IGFBPs has been undertaken since the cell-surface binding and the IGF-independent effects of IGFBPs were discovered. The evidence for IGFBP signalling occurring independently of IGF is typically summarized as follows**. “**These experiments show that the overexpression of IGFBP-3 has an inhibitory effect on cell growth which does not involve IGF binding to, or signal transduction via, the Type I IGF-R. We conclude that the cellular production of IGFBPs serves as a negative regulator of cell proliferation which involves a ***cellular signalling pathway separate from the IGF-IGF-R system***.”

The presence of IGFBP receptors was first suspected from evidence of the binding of IGFs to cell-associated IGFBPs. This binding to surface/cell/membrane-associated sites, besides to the actual specific IGF receptors, was reported in placenta and brain in 1984, where evidence was presented that this was due to bound IGFBPs. Surface-bound IGFBP was also implicated in the “paradoxical” increase of labeled IGF-I in binding studies. The magnitude of this binding to IGFBPs compared to receptor binding was persuasive as demonstrated in fibroblasts and endometrial cancer cells and seemed related to IGFBP’s ability to potentiate the cellular response to IGF-I in various fibroblasts and in smooth muscle cells. This was then more than just extracellular binding, as IGFBPs were internalized. For example, about 90% of the cell-associated radioactivity from labelled IGFBP-3 was internalized from human fetal skin fibroblasts and rat glioma cells in four hours. Note that many surface-binding studies were performed at 12-15^o^C to allow the binding of protein to the surface but to prevent the next, normally-rapid, internalization step that occurs at 37^o^C.

The cell-associated IGFBP is bound from the extracellular space and is not secreted IGFBP derived from the same cell. In a hamster fibroblast cell line, low concentrations of IGF-I produce a decrease in IGFBP secretion but an increase in cell-surface IGFBP. At high concentrations of IGF-I, there is an increase in IGFBP secretion but a decrease in cell-surface IGFBP. ***Table* 1** summaries the evidence for the presence of IGFBP receptors.

**TABLE S1. Receptors for IGFBPs and Related Cellular Effects of IGFBPs**

| ***Cell type*** | ***Receptor Description*** | ***Reported Cell Effect*; Reference*** |
| --- | --- | --- |
| **IGFBP-1** | | |
| Chinese hamster ovary | α_5_β_1-_ Integrin | Increased migration |
| Trophoblast | α_5_β_1-_ Integrin | Increased migration but no increased proliferation |
| Schwannoma | α_5_β_1-_ Integrin | Increased proliferation, activation of pathway to localize FAK in the nucleus |
| Breast cancer  (T47D, Hs578T) | α_5_β_1-_Integrin | Apoptosis; caused significant  dephosphorylation of FAK. |
| **IGFBP-2** | | |
| Small cell lung cancer | Surface-associated | Decreased proliferation |
| Olfactory bulb | Surface-associated, ~ 200 kDa chondroitin-  sulfate proteoglycans | Not assessed |
| Neuroblastoma line  SKN-SHEP | Surface-associated, chondroitin-sulfate proteoglycans | Increased proliferation by overexpression of IGFBP-2 |
| Smooth muscle cell | Surface-associated | Decreased proliferation |
| Breast cancer  (MCF-7,transfected) | α_v_β_3_ Integrin | Decreased proliferation |
| Breast cancer Hs578T; Ewing sarcoma A673 | α_5_β_1_-Integrin | Decreased proliferation |
| Prostate cancer cells LAPC-4 | Mooted | Increased proliferation |
| **IGFBP-3** | | |
| Fibroblasts | Membrane-associated | Preincubation with BP enhanced IGF-I effect |
| Chick embryo fibroblasts | Low affinity, 60,000 sites per cell | Decreased proliferation |
| Sertoli | Membrane- associated | Nil reported |
| Breast cancer  (Hs578T) | Membrane-  associated | Decreased proliferation |
| Breast cancer  (MCF-7) | Membrane-  associated | Decreased proliferation |
| Mink lung epithelial | Type V TGF-β** | Decreased proliferation |
| Normal tissue | “Cell death receptor” | Apoptosis |
| Prostate cancer | Ternary complex with transferrin (Tf) and Tf receptor | Mediator of apoptosis |
| Endometrial cancer | Surface-associated | Decreased proliferation |
| Platelets | Membrane-associated | Stored in cells for release |
| Microvascular endothelial | Membrane- associated; Low affinity | Cell affinity |
| **IGFBP-4** | | |
| Mink lung epithelial | Type V TGF-β** | Decreased proliferation |
| Microvascular endothelial | Membrane- associated ;  Low affinity, | Cell affinity |
| **IGFBP-5** | | |
| Osteoblast | Surface-associated | Increased proliferation |
| Osteoblast-like | 420-kDa receptor | Increased proliferation.  Phosphorylation of receptor |
| Osteoblast | Surface-associated | Increased proliferation |
| Mesangial | Surface-associated | Stimulates cell migration |
| Mink lung epithelial | Type V TGF-β** | Decreased proliferation |
| Intestinal smooth muscle cells (SMC) | Activation of intracellular kinases | Increased proliferation |
| Vascular SMC | Cellular entry and nuclear translocation | Cell migration |
| **IGFBP-6** | | |
| Rhabdomyosarcoma | Not specified | Increased migration; IGF-independent chemotaxis |
| Fibroblasts | Not specified | Prevents apoptosis and delays senescence |
| Rhabdomyosarcoma and kidney/neuron | Leads to nuclear localization | increased apoptosis |
| Osteosarcoma | Cell binding (?) | Increased proliferation |

*This Figure lists the potential receptors for the IGFBPs (1-6) and the cell effects related to receptor binding.*

** Inhibitory effects reported are thought to be in addition to any effect of the IGFBP binding*

*to an IGF which would thus reduce stimulation by the IGF.*

*** Type V TGF-β is identical to the low-density lipoprotein receptor-related protein 1 (LRP1), also known as the α_2_-macroglobulin receptor (α_2_MR) ie. the LRP-1/alpha2M receptor. Also, it is sometimes known as Apolipoprotein E receptor (ApoER) or Cluster of Differentiation 91 (CD91)*.

Overall, the evidence for receptors for IGFBPs is very strong. With the added evidence of associated cellular effects (especially proliferation), the IGFBPs fit within the category of Trefones.

**REFERENCES:-**

1.	Valentinis B, Bhala A, DeAngelis T, Baserga R, Cohen P: **The human insulin-like growth factor (IGF) binding protein-3 inhibits the growth of fibroblasts with a targeted disruption of the IGF-I receptor gene**. *Molecular Endocrinology* 1995, **9**(3):361-367.

2.	Grizzard JD, D'Ercole AJ, Wilkins JR, Moats-Staats BM, Williams RW: **Affinity-labeled somatomedin-C receptors and binding proteins from the human fetus**. *J Clin Endocrinol Metab* 1984, **58**(3):535-543.

3.	Clemmons DR, Elgin RG, Han VK, Casella SJ, D'Ercole AJ, Van Wyk JJ: **Cultured fibroblast monolayers secrete a protein that alters the cellular binding of somatomedin-C/insulinlike growth factor I**. *J Clin Invest* 1986, **77**(5):1548-1556.

4.	De Vroede MA, Tseng LY, Katsoyannis PG, Nissley SP, Rechler MM: **Modulation of insulinlike growth factor I binding to human fibroblast monolayer cultures by insulinlike growth factor carrier proteins released to the incubation media**. *J Clin Invest* 1986, **77**(2):602-613.

5.	Phillips PD, Pignolo RJ, Cristofalo VJ: **Insulin-like growth factor-I: specific binding to high and low affinity sites and mitogenic action throughout the life span of WI-38 cells**. *J Cell Physiol* 1987, **133**(1):135-143.

6.	Hsu D, Olefsky JM: **Characterization of insulin-like growth factor (IGF) binding proteins and their role in modulating IGF-I action in BHK cells**. *J Biol Chem* 1992, **267**(35):25576-25582.

7.	Kleinman D, Karas M, Roberts CT, Jr., LeRoith D, Phillip M, Segev Y, Levy J, Sharoni Y: **Modulation of insulin-like growth factor I (IGF-I) receptors and membrane-associated IGF-binding proteins in endometrial cancer cells by estradiol**. *Endocrinology* 1995, **136**(6):2531-2537.

8.	Elgin RG, Busby WH, Jr., Clemmons DR: **An insulin-like growth factor (IGF) binding protein enhances the biologic response to IGF-I**. *Proceedings of the National Academy of Sciences of the United States of America* 1987, **84**(10):3254-3258.

9.	Yang YW, Yanagishita M, Rechler MM: **Heparin inhibition of insulin-like growth factor-binding protein-3 binding to human fibroblasts and rat glioma cells: role of heparan sulfate proteoglycans**. *Endocrinology* 1996, **137**(10):4363-4371.

10.	Oh Y, Muller HL, Lamson G, Rosenfeld RG: **Insulin-like growth factor (IGF)-independent action of IGF-binding protein-3 in Hs578T human breast cancer cells. Cell surface binding and growth inhibition**. *Journal of Biological Chemistry* 1993, **268**(20):14964-14971.

11.	Jones JI, Gockerman A, Busby WH, Jr., Wright G, Clemmons DR: **Insulin-like growth factor binding protein 1 stimulates cell migration and binds to the alpha 5 beta 1 integrin by means of its Arg-Gly-Asp sequence**. *Proc Natl Acad Sci U S A* 1993, **90**(22):10553-10557.

12.	Gleeson LM, Chakraborty C, McKinnon T, Lala PK: **Insulin-like growth factor-binding protein 1 stimulates human trophoblast migration by signaling through alpha 5 beta 1 integrin via mitogen-activated protein Kinase pathway**. *Journal of Clinical Endocrinology & Metabolism* 2001, **86**(6):2484-2493.

13.	Ammoun S, Schmid MC, Zhou L, Ristic N, Ercolano E, Hilton DA, Perks CM, Hanemann CO: **Insulin-like growth factor-binding protein-1 (IGFBP-1) regulates human schwannoma proliferation, adhesion and survival**. *Oncogene* 2012, **31**(13):1710-1722.

14.	Perks CM, Newcomb PV, Norman MR, Holly JM: **Effect of insulin-like growth factor binding protein-1 on integrin signalling and the induction of apoptosis in human breast cancer cells**. *J Mol Endocrinol* 1999, **22**(2):141-150.

15.	Reeve JG, Morgan J, Schwander J, Bleehen NM: **Role for membrane and secreted insulin-like growth factor-binding protein-2 in the regulation of insulin-like growth factor action in lung tumors**. *Cancer Res* 1993, **53**(19):4680-4685.

16.	Russo VC, Bach LA, Werther GA: **Cell membrane association of insulin-like growth factor binding protein-2 (IGFBP-2) in the rat brain olfactory bulb**. *Prog Growth Factor Res* 1995, **6**(2-4):329-336.

17.	Russo VC, Bach LA, Fosang AJ, Baker NL, Werther GA: **Insulin-like growth factor binding protein-2 binds to cell surface proteoglycans in the rat brain olfactory bulb**. *Endocrinology* 1997, **138**(11):4858-4867.

18.	Russo VC, Rekaris G, Baker NL, Bach LA, Werther GA: **Basic fibroblast growth factor induces proteolysis of secreted and cell membrane-associated insulin-like growth factor binding protein-2 in human neuroblastoma cells**. *Endocrinology* 1999, **140**(7):3082-3090.

19.	Russo VC, Schutt BS, Andaloro E, Ymer SI, Hoeflich A, Ranke MB, Bach LA, Werther GA: **Insulin-like growth factor binding protein-2 binding to extracellular matrix plays a critical role in neuroblastoma cell proliferation, migration, and invasion**. *Endocrinology* 2005, **146**(10):4445-4455.

20.	Cohen P, Noveral JP, Bhala A, Nunn SE, Herrick DJ, Grunstein MM: **Leukotriene D4 facilitates airway smooth muscle cell proliferation via modulation of the IGF axis**. *Am J Physiol* 1995, **269**(2 Pt 1):L151-157.

21.	Pereira JJ, Meyer T, Docherty SE, Reid HH, Marshall J, Thompson EW, Rossjohn J, Price JT: **Bimolecular interaction of insulin-like growth factor (IGF) binding protein-2 with alphavbeta3 negatively modulates IGF-I-mediated migration and tumor growth**. *Cancer Res* 2004, **64**(3):977-984.

22.	Schutt BS, Langkamp M, Rauschnabel U, Ranke MB, Elmlinger MW: **Integrin-mediated action of insulin-like growth factor binding protein-2 in tumor cells**. *J Mol Endocrinol* 2004, **32**(3):859-868.

23.	Moore MG, Wetterau LA, Francis MJ, Peehl DM, Cohen P: **Novel stimulatory role for insulin-like growth factor binding protein-2 in prostate cancer cells**. *Int J Cancer* 2003, **105**(1):14-19.

24.	Conover CA, Ronk M, Lombana F, Powell DR: **Structural and biological characterization of bovine insulin-like growth factor binding protein-3**. *Endocrinology* 1990, **127**(6):2795-2803.

25.	Delbe J, Blat C, Desauty G, Harel L: **Presence of IDF45 (mlGFBP-3) binding sites on chick embryo fibroblasts**. *Biochem Biophys Res Commun* 1991, **179**(1):495-501.

26.	Smith EP, Cheung PT, Ferguson A, Chernausek SD: **Mechanisms of Sertoli cell insulin-like growth factor (IGF)-binding protein-3 regulation by IGF-I and adenosine 3',5'-monophosphate**. *Endocrinology* 1992, **131**(6):2733-2741.

27.	Chen JC, Shao ZM, Sheikh MS, Hussain A, LeRoith D, Roberts CT, Jr., Fontana JA: **Insulin-like growth factor-binding protein enhancement of insulin-like growth factor-I (IGF-I)-mediated DNA synthesis and IGF-I binding in a human breast carcinoma cell line**. *J Cell Physiol* 1994, **158**(1):69-78.

28.	Leal SM, Liu Q, Huang SS, Huang JS: **The type V transforming growth factor beta receptor is the putative insulin-like growth factor-binding protein 3 receptor**. *J Biol Chem* 1997, **272**(33):20572-20576.

29.	Wu HB, Kumar A, Tsai WC, Mascarenhas D, Healey J, Rechler MM: **Characterization of the inhibition of DNA synthesis in proliferating mink lung epithelial cells by insulin-like growth factor binding protein-3**. *Journal of Cellular Biochemistry* 2000, **77**(2):288-297.

30.	Ingermann AR, Yang YF, Han J, Mikami A, Garza AE, Mohanraj L, Fan L, Idowu M, Ware JL, Kim HS *et al*: **Identification of a novel cell death receptor mediating IGFBP-3-induced anti-tumor effects in breast and prostate cancer**. *J Biol Chem* 2010, **285**(39):30233-30246.

31.	Rajah R, Valentinis B, Cohen P: **Insulin-like growth factor (IGF)-binding protein-3 induces apoptosis and mediates the effects of transforming growth factor-beta1 on programmed cell death through a p53- and IGF-independent mechanism**. *Journal of Biological Chemistry* 1997, **272**(18):12181-12188.

32.	Lee KW, Liu B, Ma L, Li H, Bang P, Koeffler HP, Cohen P: **Cellular internalization of insulin-like growth factor binding protein-3: distinct endocytic pathways facilitate re-uptake and nuclear localization**. *J Biol Chem* 2004, **279**(1):469-476.

33.	Karas M, Danilenko M, Fishman D, LeRoith D, Levy J, Sharoni Y: **Membrane-associated insulin-like growth factor-binding protein-3 inhibits insulin-like growth factor-I-induced insulin-like growth factor-I receptor signaling in ishikawa endometrial cancer cells**. *J Biol Chem* 1997, **272**(26):16514-16520.

34.	Mochizuki T, Sakai K, Iwashita M: **Effects of insulin-like growth factor (IGF) binding protein-3 (IGFBP-3) on endometrial cancer (HHUA) cell apoptosis and EGF stimulated cell proliferation in vitro**. *Growth Horm IGF Res* 2006, **16**(3):202-210.

35.	Taylor VL, Spencer EM: **Characterisation of insulin-like growth factor-binding protein-3 binding to a novel receptor on human platelet membranes**. *J Endocrinol* 2001, **168**(2):307-315.

36.	Knudtson KL, Boes M, Sandra A, Dake BL, Booth BA, Bar RS: **Distribution of chimeric IGF binding protein (IGFBP)-3 and IGFBP-4 in the rat heart: importance of C-terminal basic region**. *Endocrinology* 2001, **142**(9):3749-3755.

37.	Leal SM, Huang SS, Huang JS: **Interactions of high affinity insulin-like growth factor-binding proteins with the type V transforming growth factor-beta receptor in mink lung epithelial cells**. *J Biol Chem* 1999, **274**(10):6711-6717.

38.	Mohan S, Nakao Y, Honda Y, Landale E, Leser U, Dony C, Lang K, Baylink DJ: **Studies on the mechanisms by which insulin-like growth factor (IGF) binding protein-4 (IGFBP-4) and IGFBP-5 modulate IGF actions in bone cells**. *Journal of Biological Chemistry* 1995, **270**(35):20424-20431.

39.	Andress DL: **Heparin modulates the binding of insulin-like growth factor (IGF) binding protein-5 to a membrane protein in osteoblastic cells**. *J Biol Chem* 1995, **270**(47):28289-28296.

40.	Andress DL: **Insulin-like growth factor-binding protein-5 (IGFBP-5) stimulates phosphorylation of the IGFBP-5 receptor**. *Am J Physiol* 1998, **274**(4 Pt 1):E744-750.

41.	Schmid C, Schlapfer I, Gosteli-Peter MA, Froesch ER, Zapf J: **Effects and fate of human IGF-binding protein-5 in rat osteoblast cultures**. *Am J Physiol* 1996, **271**(6 Pt 1):E1029-1035.

42.	Abrass CK, Berfield AK, Andress DL: **Heparin binding domain of insulin-like growth factor binding protein-5 stimulates mesangial cell migration**. *Am J Physiol* 1997, **273**(6 Pt 2):F899-906.

43.	Kuemmerle JF, Zhou H: **Insulin-like growth factor-binding protein-5 (IGFBP-5) stimulates growth and IGF-I secretion in human intestinal smooth muscle by Ras-dependent activation of p38 MAP kinase and Erk1/2 pathways**. *J Biol Chem* 2002, **277**(23):20563-20571.

44.	Xu Q, Li S, Zhao Y, Maures TJ, Yin P, Duan C: **Evidence that IGF binding protein-5 functions as a ligand-independent transcriptional regulator in vascular smooth muscle cells**. *Circ Res* 2004, **94**(5):E46-54.

45.	Bach LA, Fu P, Yang Z: **Insulin-like growth factor-binding protein-6 and cancer**. *Clin Sci (Lond)* 2013, **124**(4):215-229.

46.	Micutkova L, Diener T, Li C, Rogowska-Wrzesinska A, Mueck C, Huetter E, Weinberger B, Grubeck-Loebenstein B, Roepstorff P, Zeng R *et al*: **Insulin-like growth factor binding protein-6 delays replicative senescence of human fibroblasts**. *Mech Ageing Dev* 2011, **132**(10):468-479.

47.	Iosef C, Gkourasas T, Jia CY, Li SS, Han VK: **A functional nuclear localization signal in insulin-like growth factor binding protein-6 mediates its nuclear import**. *Endocrinology* 2008, **149**(3):1214-1226.

48.	Schmid C, Keller C, Gosteli-Peter M, Zapf J: **Mitogenic and antiapoptotic effects of insulin-like growth factor binding protein-6 in the human osteoblastic osteosarcoma cell line Saos-2/B-10**. *Biochem Biophys Res Commun* 1999, **263**(3):786-789.

49.	Huang SS, Ling TY, Tseng WF, Huang YH, Tang FM, Leal SM, Huang JS: **Cellular growth inhibition by IGFBP-3 and TGF-beta1 requires LRP-1**. *Faseb J* 2003, **17**(14):2068-2081.

50.	May P, Woldt E, Matz RL, Boucher P: **The LDL receptor-related protein (LRP) family: an old family of proteins with new physiological functions**. *Ann Med* 2007, **39**(3):219-228.
