# Supplementary material for: A model to explain specific cellular communications and cellular harmony:- a hypothesis of coupled cells and interactive coupling molecules
Source: Theor Biol Med Model. 2014 Sep 14;11:40. doi: 10.1186/1742-4682-11-40 (PMC4237941; doi:10.1186/1742-4682-11-40)
Supplement: Additional file 1 — 1. “Terminology”. 2. “Change in Cellular Characteristics”. 3. Note 1 “The Roles of SCD versus ACD”. Note 2. “Categories of SCD and ACD”. Note 3 “Further Examples of these AsCD Categories”. Note 4 “Change of differentiation type.”. 4. “The Effects of Growth Factors and other Bioactive Molecules 5. Note 1, “Additional Information on the Definition of a Trefone”. Note 2 “Clarification on Ligand and Binding Protein Interaction”. Note 3 “Cell Couplets – AsCD and the a-Cell and i-Cell”. Note 4 “Drosophila – Potential Trefones and ACD”. 6. “Calculated Levels of Trefones and TCC, with Cellular Actions”. 7. “Receptors for the Six IGFBPs and Cellular Effects”. 8. Note 1 “Extracellular Binding Proteins/ Soluble Receptors”. Note 2 “Cell Receptors for Binding Proteins/Soluble Receptors”. 9. Note 1. “Further Evidence for a Receptor for TCC”. Note 2 “Problems with Cross-linking Experiments”. 10. “Evidence for Binary Receptors aside from the IGF System”. 11. “Evidence for Two Receptors for a Particular Trefone, outside the IGF System”. 12. Note 1 “Mechanisms of Nuclear Localization”. Note 2 “NL of IGFBPs”. Note 3 “NL of Potential Trefones other than the IGF System”. 13. “Evidence Supporting the Existence of a-Cells and i-Cells”. 14. “Candidates with mainly a-Cell-Type or i-Cell-Type Characteristics”. 15. “Interacting Cells and Trefones not associated with the IGF System”. 16. “Heterogeneity/Variability of Cells in Culture.” 17. “Cell lines are not Typical of Normal Cells”. 18. “Cell Receptors and Cell Markers”. 19. “Defined Couplet Cells for Insulin and Glucagon; Gastrin and Histamine”. 20. “Potential Couplet Trefones”. 21. “Further Examples of Potential Trefone and Cell Couplets”. 22. “Examples of Cellular Regulation by Complexes”. 23. “Proteolytic enzymes and their inhibitors”. 24. “Expanded Definition of ‘Trefone’ and Classes of Couplet Cell Interactions”. 25. Note 1 “Extended Trefone Couplets”. Note 2 “Singlet Cells”. 26. “Background of Cancer Research”. [file 1742-4682-11-40-S1.zip › 3484671921331230_add1/3484671921331230_add08.doc]

**Additional File** ***Eight***

***(ii) Receptors for Other Binding Proteins/Trefones,* including “Soluble Receptors”**

***Note 1. Extracellular Binding Proteins and Soluble Receptors***

*The purpose of this Note is to verify the wide-spread occurrence of potential Trefones that masquerade as simple binding proteins, soluble receptors or decoys.*

Binding proteins that complex with ICMs (*Information-Carrying Molecules and Inter-Cellular Messengers*) are said to have many functions. As part of the complex, they can act as a carrier and a storage or slow-release form of the ICM or may protect the ICM from enzymic breakdown. The total size of the complex may also restrict movement of the ICM into certain tissues of the human body and prevent rapid renal excretion.

Soluble proteins structurally related to receptors may be generated by the proteolytic cleavage of ectodomains (shedding), including release of GPI-linked ectodomains, or generated either by alternative splicing of receptor-related mRNA transcripts to produce a soluble receptor ectodomain without the transmembrane or by transcription of distinct genes homologous with the receptor to encode soluble binding protein. (In the table below, the latter is described as a binding protein, normal secretion.) Other exosome-related mechanisms are also possible.

The observation that activation of ectodomain shedding of syndecan-1 by metalloproteinases occurs via signal transduction pathways (involving protein kinase C, protein tyrosine kinase and mitogen-activated protein kinase) makes these soluble co-receptors more interesting in the context of their function in Trefone signalling.

Many proposed Trefone couplets are listed later, namely Insulin and Glucagon, Gastrin and Histamine, Dopamine and Neurotensin, Serotonin and ACTH, CGRP and IL-1, Follistatin and Myostatin, Uteroglobin and Progesterone, Rankl and Osteoprotegerin, Sarcolectin and Migration Inhibitory Factor, Glutamate and Acetyl Choline, Wnt Interactants and GDNF (Glial Cell-Derived Neurotrophic Factor) and Soluble Receptor. Of these, excluding the obvious GDNF soluble receptor, only Il-1 has a binding protein or soluble receptor. There is a tick histamine binding protein but it has a distinct non-Trefone function. Progesterone also has a binding protein in plasma besides the small uteroglobin which is predominantly from Clara cells of lung bronchioles. **TABLE S1** contains a list of these binding proteins and soluble receptors; solubilised membrane receptors are not included in the list nor are strictly intracellular binding proteins

**TABLE S1. Extracellular Binding Proteins (BPs) and/or Soluble Receptors for ICMs ie. Potential Trefones**

| **ICM** | **BP or cell**  **Receptor**  **For ICM** | **Mechanism of formation**  **or /release of BP** | **Source or Cell type;**  **Reference** |
| --- | --- | --- | --- |
| Epidermal Growth Factor (EGF) | EGFR; ErbB-1; HER1 | Alternative splicing* | Liver |
| Fibroblast Growth Factor-1 (FGF-1) | FGFR-1 | Alternative splicing | Fibroblasts |
| Fibroblast Growth Factor-1 (FGF-2) | Binding protein | Normal secretion# | Keratinocytes[8]; Squamous cell carcinomas[9] |
| FGF-2 | Soluble co-receptor (syndecan-1) | Shedding** | HeLa cells and human primary macrophages |
| Fibroblast Growth Factor-1 (FGF-23) | Secreted Klotho | Shedding | Plasma |
| Growth Hormone (GH)^a^ | GH Binding protein | Shedding (Human);  Alternative splicing* (Rodents) | Serum |
| Hepatocyte Growth Factor (HGF) | HGF Receptor  (c-Met) | Truncated beta chain | Culture supernates |
| Nerve Growth Factor (NGF) | NGF receptor  (TrkA) | Truncated  *** | Cultured Schwann cells |
| Brain-derived neurotrophic factor (BDNF), | TrkB | Alternative splicing | Expressed |
| Neurotrophin-3 | TrkC | Truncated  [gp90 (TrkCNC2)] | Primary cultures of cortical neurons and brain extracts |
| Platelet-Derived Growth Factor (PDGF) | PDGFR-alpha | Normal;  Shedding | Serum;  Culture medium of osteosarcoma cell line |
| Transforming Growth Factor- alpha (TGF-α) | EGF receptor  (See above) | - | - |
| Transforming Growth Factor- beta (TGF-β) | TGF beta receptor | Hamster ovary- | Cytosolic;  Serum |
| Tumour Necrosis Factor (TNF) | TNF-R | Shedding | Serum, Urine |
| Vascular Endothelial Growth Factor (VEGF) | Soluble Flt-1 | Alternative splicing | Vascular endothelial  Trophoblast;  Serum |
| Endostatin | Tropomyosin | Normal | In vitro |
| Erythropoietin (Epo) | Epo receptor | Alternative splicing | Various cancer cell lines |
| Fas ligand | Decoy receptor 3 ([DcR3](http://en.wikipedia.org/wiki/DcR3)) | Alternative splicing | Primary lung and colon tumours |
| Gastrin-Releasing  Peptide | GRP binding protein | - | Fibroblasts (Swiss 3T3 cells) |
| Granulocyte-macrophage colony-stimulating factor (GM-CSF) | GM-CSF receptor | Truncated | Chorio-carcinoma cells. |
| Granulocyte  colony-stimulating factor (G-CSF) | G-CSF receptor | Alternative splicing | Leukemia cell lines and placenta |
| Interferon-γ | Soluble IFN-γ receptor | Alternative splicing | Myeloma cell lines (U266S) |
| Interleukin-1(IL-1) | IL-1 type I receptor (IL-1RI);  IL-1 type II receptor | Shedding  Shedding | T cells and others  Myelomonocytic cells |
| Interleukin-2(IL-2) | IL-2Ralpha | Shedding  Serum | T cells (activated);  Mononuclear cells  T-cell lymphoma,  Plasma cell disorders |
| Interleukin-4(IL-4) | IL-4Ralpha receptor | Alternative splicing | Cytotoxic T lymphocytes |
| Interleukin-5(IL-5) | IL-5Ralpha receptor | Alternative splicing | Broncho-alveolar lavage fluid |
| Interleukin-6(IL-6) | IL-6 receptor | Shedding | Monocytes |
| Interleukin-7(IL-7) | IL-7Ralpha | Alternative splicing | T-Cells |
| Interleukin-9(IL-9) | IL-9Ralpha | Alternative splicing | T-Cells |
| Interleukin-17 (IL-17) | IL-17 binding protein | Normal secretion | B- and T-Cells |
| Interleukin-18 (IL-18) | IL-18 binding protein | Normal secretion | Urine |
| Leukemia inhibitory factor (LIF) | LIFRalpha | Alternative splicing | T-Cells |
| [Neuregulin-1](http://en.wikipedia.org/wiki/Neuregulin_1) (NRG1), (Heregulin) | ErbB-3  ErbB-4 | Normal secretion | Ovarian carcinoma-derived cell line |
| Prolactin | Prolactin binding protein | Alternative splicing | Rabbit milk; Seminal fluid; Body organs |
| RANKL,  TRAIL | Osteoprotegerin | Secreted | Serum^,^ |
| Thrombopoietin  (TPO) | Soluble TPO receptor (MPL) | Secreted | Serum |
| Vasoactive intestinal polypeptide (VIP) | Soluble PAC1 receptor | Alternative splicing | Potential |
| Wnt | Frizzled-related protein | Normal secretion | Embryonic  tissue |
| Acetylcholine | Binding protein | Normal secretion | Glia cells |
| ACTH | Binding protein | Normal secretion | Plasma (not albumin with MW 40kDa) |
| γ-Aminobutyric acid (GABA) | GABA receptor B (a GPCR) | Alternative splicing | Peripheral tissues (Kidney, small intestine, adrenal, muscle…) |
| Androgens | Androgen binding protein | Normal secretion | Plasma^-^ |
| Cortisol | Cortisol binding protein | Normal secretion | Plasma^-^ |
| Corticotropin releasing hormone (CRH) | CRH binding protein | Normal secretion | Brain  Plasma |
| Estradiol | Sex-hormone binding protein | Normal secretion | Plasma |
| Progesterone | Cortisol binding protein | Normal secretion | Plasma |
| Thyroid hormones (T3 and T4) | Thyroxine-binding globulin (TBG) | Normal secretion | Plasma |
| Thyroid stimulating hormone (TSH) | TSH binding protein | Normal secretion | Plasma |
| TSH releasing hormone (TRH) | TRH binding protein | Normal secretion | Plasma^-^ |
| Kallikrein (KLK) | Kallistatin = KLK binding protein | Normal secretion | Serum |
| Prostatic specific antigen (PSA) | Apha2-macroglobulin | Normal secretion | Prostate cancer cell lines (2) |
| tPA | PAI-1 | Normal  secretion | Plasma |
| Urokinase-type Plasminogen activator (uPA) | uPA receptor | Shedding | Plasma |
| Phospholipase A2 | PLA2 receptor | Alternative splicing | Kidney |
| Folate | Folate Binding Protein | Normal secretion | Serum/milk |
| Biotin | Biotin-Binding Protein | Normal secretion | Chick plasma |
| Retinol | Retinol binding protein 4 | Normal secretion | Serum |
| Riboflavin | Riboflavin carrier protein | Normal secretion | Serum |
| Vitamin B_12_ | Transcobalamin  Intrinsic factor | Normal secretion | Serum  Duodenum |
| Vitamin D | Binding protein | Normal secretion | Serum |
| Lag-2 and apx-1 (fixed ligands) | Truncated forms | Normal secretion | *Caenorhabditis elegans* |

*The Table* *lists the binding proteins and/or soluble receptors for a variety of ICMs with the mechanism of formation or release of the couplet substance along with the source or cell type*

** Alternate splicing of the receptor precursor mRNA followed by normal secretion of a truncated receptor.*

*# Normal protein synthesis of a binding protein, not related to the receptor, and normal secretion from the cell.*

*** Shedding refers to the proteolytic cleavage of a membrane receptor in situ to release the extracellular domain from the cell.*

**** “Truncated” proteins may be derived from alternative splicing or from shedding by proteolytic cleavage.*

***Note 2. Cell Receptors for Binding Proteins/Soluble Receptors***

*The purpose of this Note is to support the concept that binding proteins/soluble receptors are potential Trefones by evidence of the presence of receptors for them.*

Many binding proteins/Trefones have been shown to have cell-membrane receptors. Receptors for the six IGFBPs have already been discussed in **Additional File** ***Seven***. Here, **TABLE S1** contains a list of these other BPs (ie. excluding IGFBPs) for which a receptor has been detected.

**TABLE S1. Binding Proteins for which a Receptor has been Detected**

| **Binding Protein/Trefone** | **Receptor cell or Source; Reference** |
| --- | --- |
| Corticosteroid binding globulin (transcortin) | Human syncytiotrophoblast placental cells |
| Sex Hormone Binding Globulin | Prostate gland  Breast cancer cells (MCF-7)  Hepatoma cells (Hep G2) |
| Thyroxine binding globulin | Hepatoma cells (Hep G2) |
| Transthyretin | Human peripheral mononuclear cells |
| Biotin-Binding Protein | Oocyte vitelline membrane |
| Folate Binding Protein (apo & holo)) | Renal epithelia Megalin Complex |
| Intrinsic factor (Free) | Brush borders of enterocytes/ileum |
| Retinol-binding protein | Retinal pigment epithelial cell membrane |
| Riboflavin Carrier Protein | Chicken oocyte |
| Transcobalamin | Liver kidney plasma membranes |
| Vit D Binding Protein | Pre-B-lymphoma cell line |
| Lactoferrin | Neutrophilic polymorpho-nuclear Leukocytes |
| α_2_-Macroglobulin | Rheumatoid cells |
| Klotho (Secreted) | Renal epithelial cells |
| uPAR (Soluble) | Normal and neoplastic hematopoietic cells |

*This Table lists a number of binding proteins/soluble receptors that have cell-membrane receptors and lists the cells with these receptors***.**

**“Co-receptors” as receptors for these potential Trefones:**

Cell-surface co-receptors have functions in signalling and it is feasible that some of these are actually receptors for binding proteins/soluble receptors. Some of these contain high levels of carbohydrate which may participate in the binding. Heparin sulphate has a role in signalling by the ligand hepatocyte growth factor/scatter factor and its receptor Met.

**Transmembrane HSPG (heparan sulphate proteoglycan) as receptors:**

While proteoglycans (PGs) containing the [glycosaminoglycan](http://en.wikipedia.org/wiki/Glycosaminoglycan) heparan sulphate, HS, (eg.perlecan) may have functions within the extracellular matrix by storing ICMs or by the heparanase release of bioactive fragments, transmembrane HSPGs eg. [syndecans](http://en.wikipedia.org/wiki/Syndecans) and the [glycosylphosphatidylinositol](http://en.wikipedia.org/wiki/Glycosylphosphatidylinositol) (GPI) anchored [glypicans](http://en.wikipedia.org/wiki/Glypicans), have receptor functions

Either the protein core or the heparan sulphate chains may contain specific binding sites for Trefones leading to their internalization. The product of shedding of syndecan-1 (soluble Sdc1) from stromal fibroblasts stimulates breast cancer cell proliferation perhaps by the formation of a ternary complex with FGF-2 and its receptor in a reciprocal feedback loop. Breast carcinoma cells induce expression of syndecan in mammary fibroblasts; reciprocally the fibroblasts stimulate the carcinoma cell growth by involvement in Sdc1 shedding from syndecans. Proteolytic conversion of Sdc1 from a membrane-bound co-receptor into a soluble molecule marks a switch from a proliferative to an invasive phenotype.

**REFERENCES:-**

1.	Heaney ML, Golde DW: **Soluble cytokine receptors**. *Blood* 1996, **87**(3):847-857.

2.	Levine SJ: **Mechanisms of soluble cytokine receptor generation**. *J Immunol* 2004, **173**(9):5343-5348.

3.	Brule S, Charnaux N, Sutton A, Ledoux D, Chaigneau T, Saffar L, Gattegno L: **The shedding of syndecan-4 and syndecan-1 from HeLa cells and human primary macrophages is accelerated by SDF-1/CXCL12 and mediated by the matrix metalloproteinase-9**. *Glycobiology* 2006, **16**(6):488-501.

4.	Petch LA, Harris J, Raymond VW, Blasband A, Lee DC, Earp HS: **A truncated, secreted form of the epidermal growth factor receptor is encoded by an alternatively spliced transcript in normal rat tissue**. *Mol Cell Biol* 1990, **10**(6):2973-2982.

5.	Johnson DE, Lu J, Chen H, Werner S, Williams LT: **The human fibroblast growth factor receptor genes: a common structural arrangement underlies the mechanisms for generating receptor forms that differ in their third immunoglobulin domain**. *Mol Cell Biol* 1991, **11**(9):4627-4634.

6.	Root LL, Shipley GD: **Normal human fibroblasts produce membrane-bound and soluble isoforms of FGFR-1**. *Mol Cell Biol Res Commun* 2000, **3**(2):87-97.

7.	Wang JF, Shen M, Fong GH, Hill DJ: **A soluble fibroblast growth factor receptor is released from HL-60 promyelocytic leukemia cells: implications for paracrine growth control**. *Growth Factors* 2000, **17**(3):203-214.

8.	Imura A, Iwano A, Tohyama O, Tsuji Y, Nozaki K, Hashimoto N, Fujimori T, Nabeshima Y: **Secreted Klotho protein in sera and CSF: implication for post-translational cleavage in release of Klotho protein from cell membrane**. *FEBS Lett* 2004, **565**(1-3):143-147.

9.	Postel-Vinay MC, de Mello Coelho V, Gagnerault MC, Dardenne M: **Growth hormone stimulates the proliferation of activated mouse T lymphocytes**. *Endocrinology* 1997, **138**(5):1816-1820.

10.	Prat M, Crepaldi T, Gandino L, Giordano S, Longati P, Comoglio P: **C-terminal truncated forms of Met, the hepatocyte growth factor receptor**. *Mol Cell Biol* 1991, **11**(12):5954-5962.

11.	DiStefano PS, Johnson EM, Jr.: **Identification of a truncated form of the nerve growth factor receptor**. *Proc Natl Acad Sci U S A* 1988, **85**(1):270-274.

12.	Eide FF, Vining ER, Eide BL, Zang K, Wang XY, Reichardt LF: **Naturally occurring truncated trkB receptors have dominant inhibitory effects on brain-derived neurotrophic factor signaling**. *J Neurosci* 1996, **16**(10):3123-3129.

13.	Mateos S, Calothy G, Lamballe F: **The noncatalytic TrkCNC2 receptor is cleaved by metalloproteases upon neurotrophin-3 stimulation**. *Oncogene* 2003, **22**(5):740-745.

14.	Huang JS, Huang SS, Deuel TF: **Specific covalent binding of platelet-derived growth factor to human plasma alpha 2-macroglobulin**. *Proc Natl Acad Sci U S A* 1984, **81**(2):342-346.

15.	Tiesman J, Hart CE: **Identification of a soluble receptor for platelet-derived growth factor in cell-conditioned medium and human plasma**. *J Biol Chem* 1993, **268**(13):9621-9628.

16.	Roy SK, Kole AR: **Transforming growth factor-beta receptor type II expression in the hamster ovary: cellular site(s), biochemical properties, and hormonal regulation**. *Endocrinology* 1995, **136**(10):4610-4620.

17.	Blann AD, Wang JM, Wilson PB, Kumar S: **Serum levels of the TGF-beta receptor are increased in atherosclerosis**. *Atherosclerosis* 1996, **120**(1-2):221-226.

18.	Wallach D, Engelmann H, Nophar Y, Aderka D, Kemper O, Hornik V, Holtmann H, Brakebusch C: **Soluble and cell surface receptors for tumor necrosis factor**. *Agents Actions Suppl* 1991, **35**:51-57.

19.	Aderka D: **The potential biological and clinical significance of the soluble tumor necrosis factor receptors**. *Cytokine Growth Factor Rev* 1996, **7**(3):231-240.

20.	Kendall RL, Thomas KA: **Inhibition of vascular endothelial cell growth factor activity by an endogenously encoded soluble receptor**. *Proc Natl Acad Sci U S A* 1993, **90**(22):10705-10709.

21.	Clark DE, Smith SK, He Y, Day KA, Licence DR, Corps AN, Lammoglia R, Charnock-Jones DS: **A vascular endothelial growth factor antagonist is produced by the human placenta and released into the maternal circulation**. *Biol Reprod* 1998, **59**(6):1540-1548.

22.	MacDonald NJ, Shivers WY, Narum DL, Plum SM, Wingard JN, Fuhrmann SR, Liang H, Holland-Linn J, Chen DH, Sim BK: **Endostatin binds tropomyosin. A potential modulator of the antitumor activity of endostatin**. *J Biol Chem* 2001, **276**(27):25190-25196.

23.	Arcasoy MO, Jiang X, Haroon ZA: **Expression of erythropoietin receptor splice variants in human cancer**. *Biochem Biophys Res Commun* 2003, **307**(4):999-1007.

24.	Pitti RM, Marsters SA, Lawrence DA, Roy M, Kischkel FC, Dowd P, Huang A, Donahue CJ, Sherwood SW, Baldwin DT *et al*: **Genomic amplification of a decoy receptor for Fas ligand in lung and colon cancer**. *Nature* 1998, **396**(6712):699-703.

25.	Kane MA, Portanova LB, Kelley K, Holley M, Ross SE, Boose D, Escobedo-Morse A, Alvarado B: **Novel soluble, high-affinity gastrin-releasing peptide binding proteins in Swiss 3T3 fibroblasts**. *Peptides* 1994, **15**(6):993-1001.

26.	Raines MA, Liu L, Quan SG, Joe V, DiPersio JF, Golde DW: **Identification and molecular cloning of a soluble human granulocyte-macrophage colony-stimulating factor receptor**. *Proc Natl Acad Sci U S A* 1991, **88**(18):8203-8207.

27.	Fukunaga R, Seto Y, Mizushima S, Nagata S: **Three different mRNAs encoding human granulocyte colony-stimulating factor receptor**. *Proc Natl Acad Sci U S A* 1990, **87**(22):8702-8706.

28.	Abramovich C, Ratovitski E, Lundgren E, Revel M: **Identification of mRNAs encoding two different soluble forms of the human interferon alpha-receptor**. *FEBS Lett* 1994, **338**(3):295-300.

29.	Fanslow WC, Sims JE, Sassenfeld H, Morrissey PJ, Gillis S, Dower SK, Widmer MB: **Regulation of alloreactivity in vivo by a soluble form of the interleukin-1 receptor**. *Science* 1990, **248**(4956):739-742.

30.	Orlando S, Sironi M, Bianchi G, Drummond AH, Boraschi D, Yabes D, Mantovani A: **Role of metalloproteases in the release of the IL-1 type II decoy receptor**. *J Biol Chem* 1997, **272**(50):31764-31769.

31.	Morris JC, Waldmann TA: **Advances in interleukin 2 receptor targeted treatment**. *Ann Rheum Dis* 2000, **59 Suppl 1**:i109-114.

32.	Orson FM, Saadeh CK, Lewis DE, Nelson DL: **Interleukin 2 receptor expression by T cells in human aging**. *Cell Immunol* 1989, **124**(2):278-291.

33.	Wasik MA, Sioutos N, Tuttle M, Butmarc JR, Kaplan WD, Kadin ME: **Constitutive secretion of soluble interleukin-2 receptor by human T cell lymphoma xenografted into SCID mice. Correlation of tumor volume with concentration of tumor-derived soluble interleukin-2 receptor in body fluids of the host mice**. *Am J Pathol* 1994, **144**(5):1089-1097.

34.	Smith SR, Morgan L: **Soluble IL-6R in plasma cell dyscrasias**. *Leuk Lymphoma* 1997, **26**(5-6):489-495.

35.	Mosley B, Beckmann MP, March CJ, Idzerda RL, Gimpel SD, VandenBos T, Friend D, Alpert A, Anderson D, Jackson J *et al*: **The murine interleukin-4 receptor: molecular cloning and characterization of secreted and membrane bound forms**. *Cell* 1989, **59**(2):335-348.

36.	Liu LY, Sedgwick JB, Bates ME, Vrtis RF, Gern JE, Kita H, Jarjour NN, Busse WW, Kelly EA: **Decreased expression of membrane IL-5 receptor alpha on human eosinophils: I. Loss of membrane IL-5 receptor alpha on airway eosinophils and increased soluble IL-5 receptor alpha in the airway after allergen challenge**. *J Immunol* 2002, **169**(11):6452-6458.

37.	Tavernier J, Tuypens T, Plaetinck G, Verhee A, Fiers W, Devos R: **Molecular basis of the membrane-anchored and two soluble isoforms of the human interleukin 5 receptor alpha subunit**. *Proc Natl Acad Sci U S A* 1992, **89**(15):7041-7045.

38.	Mullberg J, Schooltink H, Stoyan T, Gunther M, Graeve L, Buse G, Mackiewicz A, Heinrich PC, Rose-John S: **The soluble interleukin-6 receptor is generated by shedding**. *Eur J Immunol* 1993, **23**(2):473-480.

39.	Goodwin RG, Friend D, Ziegler SF, Jerzy R, Falk BA, Gimpel S, Cosman D, Dower SK, March CJ, Namen AE *et al*: **Cloning of the human and murine interleukin-7 receptors: demonstration of a soluble form and homology to a new receptor superfamily**. *Cell* 1990, **60**(6):941-951.

40.	Renauld JC, Druez C, Kermouni A, Houssiau F, Uyttenhove C, Van Roost E, Van Snick J: **Expression cloning of the murine and human interleukin 9 receptor cDNAs**. *Proc Natl Acad Sci U S A* 1992, **89**(12):5690-5694.

41.	Haudenschild D, Moseley T, Rose L, Reddi AH: **Soluble and transmembrane isoforms of novel interleukin-17 receptor-like protein by RNA splicing and expression in prostate cancer**. *J Biol Chem* 2002, **277**(6):4309-4316.

42.	Dinarello CA: **Targeting interleukin 18 with interleukin 18 binding protein**. *Ann Rheum Dis* 2000, **59 Suppl 1**:i17-20.

43.	Owczarek CM, Layton MJ, Robb LG, Nicola NA, Begley CG: **Molecular basis of the soluble and membrane-bound forms of the murine leukemia inhibitory factor receptor alpha-chain. Expression in normal, gestating, and leukemia inhibitory factor nullizygous mice**. *J Biol Chem* 1996, **271**(10):5495-5504.

44.	Lee H, Maihle NJ: **Isolation and characterization of four alternate c-erbB3 transcripts expressed in ovarian carcinoma-derived cell lines and normal human tissues**. *Oncogene* 1998, **16**(25):3243-3252.

45.	Raikar RS, Dattatreyamurty B, Sheth AR: **Evidence for the presence of specific prolactin binding protein in rat seminal vesicle fluid**. *Endocrinol Exp* 1981, **15**(1):55-64.

46.	Trott JF, Hovey RC, Koduri S, Vonderhaar BK: **Alternative splicing to exon 11 of human prolactin receptor gene results in multiple isoforms including a secreted prolactin-binding protein**. *J Mol Endocrinol* 2003, **30**(1):31-47.

47.	Ziolkowska M, Kurowska M, Radzikowska A, Luszczykiewicz G, Wiland P, Dziewczopolski W, Filipowicz-Sosnowska A, Pazdur J, Szechinski J, Kowalczewski J *et al*: **High levels of osteoprotegerin and soluble receptor activator of nuclear factor kappa B ligand in serum of rheumatoid arthritis patients and their normalization after anti-tumor necrosis factor alpha treatment**. *Arthritis Rheum* 2002, **46**(7):1744-1753.

48.	Emery JG, McDonnell P, Burke MB, Deen KC, Lyn S, Silverman C, Dul E, Appelbaum ER, Eichman C, DiPrinzio R *et al*: **Osteoprotegerin is a receptor for the cytotoxic ligand TRAIL**. *J Biol Chem* 1998, **273**(23):14363-14367.

49.	Hornkohl A, Selesi D, Bennett L, Hockman H, Nichol J, Hunt P: **Thrombopoietin and sMPL concentrations in the serum and plasma of normal donors.** *Blood* 1995, **86**:898a.

50.	Blechman J, Levkowitz G: **Alternative Splicing of the Pituitary Adenylate Cyclase-Activating Polypeptide Receptor PAC1: Mechanisms of Fine Tuning of Brain Activity**. *Front Endocrinol (Lausanne)* 2013, **4**:55.

51.	Mii Y, Taira M: **Secreted Wnt "inhibitors" are not just inhibitors: regulation of extracellular Wnt by secreted Frizzled-related proteins**. *Dev Growth Differ* 2011, **53**(8):911-923.

52.	Bovolenta P, Esteve P, Ruiz JM, Cisneros E, Lopez-Rios J: **Beyond Wnt inhibition: new functions of secreted Frizzled-related proteins in development and disease**. *J Cell Sci* 2008, **121**(Pt 6):737-746.

53.	Smit AB, Syed NI, Schaap D, van Minnen J, Klumperman J, Kits KS, Lodder H, van der Schors RC, van Elk R, Sorgedrager B *et al*: **A glia-derived acetylcholine-binding protein that modulates synaptic transmission**. *Nature* 2001, **411**(6835):261-268.

54.	Jenkins PJ, Cross TA, Perry LA, Medbak SA, Besser GM, Clark AJ: **The influence of plasma on basal and ACTH-stimulated in vitro adrenocortical steroidogenesis**. *J Endocrinol* 1999, **162**(1):155-161.

55.	Schwarz DA, Barry G, Eliasof SD, Petroski RE, Conlon PJ, Maki RA: **Characterization of gamma-aminobutyric acid receptor GABAB(1e), a GABAB(1) splice variant encoding a truncated receptor**. *J Biol Chem* 2000, **275**(41):32174-32181.

56.	Potter E, Behan DP, Linton EA, Lowry PJ, Sawchenko PE, Vale WW: **The central distribution of a corticotropin-releasing factor (CRF)-binding protein predicts multiple sites and modes of interaction with CRF**. *Proc Natl Acad Sci U S A* 1992, **89**(9):4192-4196.

57.	Suda T, Iwashita M, Tozawa F, Ushiyama T, Tomori N, Sumitomo T, Nakagami Y, Demura H, Shizume K: **Characterization of corticotropin-releasing hormone binding protein in human plasma by chemical cross-linking and its binding during pregnancy**. *J Clin Endocrinol Metab* 1988, **67**(6):1278-1283.

58.	Couet J, Sar S, Jolivet A, Hai MT, Milgrom E, Misrahi M: **Shedding of human thyrotropin receptor ectodomain. Involvement of a matrix metalloprotease**. *J Biol Chem* 1996, **271**(8):4545-4552.

59.	Chao J, Miao RQ, Chen V, Chen LM, Chao L: **Novel roles of kallistatin, a specific tissue kallikrein inhibitor, in vascular remodeling**. *Biol Chem* 2001, **382**(1):15-21.

60.	Misra UK, Payne S, Pizzo SV: **Ligation of prostate cancer cell surface GRP78 activates a proproliferative and antiapoptotic feedback loop: a role for secreted prostate-specific antigen**. *J Biol Chem* 2011, **286**(2):1248-1259.

61.	Brogren H, Sihlbom C, Wallmark K, Lonn M, Deinum J, Karlsson L, Jern S: **Heterogeneous glycosylation patterns of human PAI-1 may reveal its cellular origin**. *Thromb Res* 2008, **122**(2):271-281.

62.	Montuori N, Ragno P: **Multiple activities of a multifaceted receptor: roles of cleaved and soluble uPAR**. *Front Biosci* 2009, **14**:2494-2503.

63.	Ancian P, Lambeau G, Mattei MG, Lazdunski M: **The human 180-kDa receptor for secretory phospholipases A2. Molecular cloning, identification of a secreted soluble form, expression, and chromosomal localization**. *J Biol Chem* 1995, **270**(15):8963-8970.

64.	Henderson GB: **Folate-binding proteins**. *Annu Rev Nutr* 1990, **10**:319-335.

65.	White HB, 3rd, Whitehead CC: **Role of avidin and other biotin-binding proteins in the deposition and distribution of biotin in chicken eggs. Discovery of a new biotin-binding protein**. *Biochem J* 1987, **241**(3):677-684.

66.	Rask L, Anundi H, Fohlman J, Peterson PA: **The complete amino acid sequence of human serum retinol-binding protein**. *Ups J Med Sci* 1987, **92**(2):115-146.

67.	Visweswariah SS, Adiga PR: **Isolation of riboflavin carrier proteins from pregnant human and umbilical cord serum: similarities with chicken egg riboflavin carrier protein**. *Biosci Rep* 1987, **7**(7):563-571.

68.	England JM, Clarke HG, Down MC, Chanarin I: **Studies on the transcobalamins**. *Br J Haematol* 1973, **25**(6):737-749.

69.	Festen HP: **Intrinsic factor secretion and cobalamin absorption. Physiology and pathophysiology in the gastrointestinal tract**. *Scand J Gastroenterol Suppl* 1991, **188**:1-7.

70.	Bouillon R, van Baelen H, de Moor P: **The measurement of the vitamin D-binding protein in human serum**. *J Clin Endocrinol Metab* 1977, **45**(2):225-231.

71.	Cooke NE, Walgate J, Haddad JG, Jr.: **Human serum binding protein for vitamin D and its metabolites. II. Specific, high affinity association with a protein in nucleated tissue**. *J Biol Chem* 1979, **254**(13):5965-5971.

72.	Sun X, Artavanis-Tsakonas S: **Secreted forms of DELTA and SERRATE define antagonists of Notch signaling in Drosophila**. *Development* 1997, **124**(17):3439-3448.

73.	Avvakumov GV, Strel'chyonok OA: **Evidence for the involvement of the transcortin carbohydrate moiety in the glycoprotein interaction with the plasma membrane of human placental syncytiotrophoblast**. *Biochim Biophys Acta* 1988, **938**(1):1-6.

74.	Hryb DJ, Khan MS, Romas NA, Rosner W: **Solubilization and partial characterization of the sex hormone-binding globulin receptor from human prostate**. *Journal of Biological Chemistry* 1989, **264**(10):5378-5383.

75.	Porto CS, Lazari MF, Abreu LC, Bardin CW, Gunsalus GL: **Receptors for androgen-binding proteins: internalization and intracellular signalling**. *J Steroid Biochem Mol Biol* 1995, **53**(1-6):561-565.

76.	Frairia R, Fortunati N, Berta L, Fazzari A, Fissore F, Gaidano G: **Sex steroid binding protein (SBP) receptors in estrogen sensitive tissues**. *J Steroid Biochem Mol Biol* 1991, **40**(4-6):805-812.

77.	Hashizume K, Sakurai A, Miyamoto T, Yamauchi K, Nishii Y: **Effect of thyroxine-binding globulin (TBG) on thyroxine (T4) uptake by human peripheral mononuclear cells: evidence of TBG-dependent uptake of T4**. *Endocrinol Jpn* 1986, **33**(5):665-674.

78.	Divino CM, Schussler GC: **Receptor-mediated uptake and internalization of transthyretin**. *J Biol Chem* 1990, **265**(3):1425-1429.

79.	Vieira AV, White HB, 3rd, Vieira PM: **An oocytic membrane receptor for biotin-binding protein**. *FEBS Lett* 1996, **382**(1-2):183-185.

80.	Birn H, Zhai X, Holm J, Hansen SI, Jacobsen C, Christensen EI, Moestrup SK: **Megalin binds and mediates cellular internalization of folate binding protein**. *Febs J* 2005, **272**(17):4423-4430.

81.	Marcoullis G, Grasbeck R: **Solubilised intrinsic factor receptor from pig ileum and its characteristics**. *Biochim Biophys Acta* 1977, **496**(1):36-51.

82.	Bavik CO, Eriksson U, Allen RA, Peterson PA: **Identification and partial characterization of a retinal pigment epithelial membrane receptor for plasma retinol-binding protein**. *J Biol Chem* 1991, **266**(23):14978-14985.

83.	Sooryanarayana, Sarkar S, Adiga PR, Visweswariah SS: **Identification and characterization of receptors for riboflavin carrier protein in the chicken oocyte. Role of the phosphopeptide in mediating receptor interaction**. *Biochim Biophys Acta* 1998, **1382**(2):230-242.

84.	Scott JS, Bowman EP, Cooksley WG: **A comparison of cobalamin binding by liver and kidney in rat and man**. *Clin Sci (Lond)* 1985, **68**(3):357-364.

85.	Esteban C, Geuskens M, Ena JM, Mishal Z, Macho A, Torres JM, Uriel J: **Receptor-mediated uptake and processing of vitamin D-binding protein in human B-lymphoid cells**. *J Biol Chem* 1992, **267**(14):10177-10183.

86.	Maneva AI, Sirakov LM, Manev VV: **Lactoferrin binding to neutrophilic polymorphonuclear leucocytes**. *Int J Biochem* 1983, **15**(7):981-984.

87.	Misra UK, Gonzalez-Gronow M, Gawdi G, Pizzo SV: **Up-regulation of the alpha2-macroglobulin signaling receptor on rheumatoid synovial fibroblasts**. *J Biol Chem* 1997, **272**(1):497-502.

88.	Doi S, Zou Y, Togao O, Pastor JV, John GB, Wang L, Shiizaki K, Gotschall R, Schiavi S, Yorioka N *et al*: **Klotho inhibits transforming growth factor-beta1 (TGF-beta1) signaling and suppresses renal fibrosis and cancer metastasis in mice**. *J Biol Chem* 2011, **286**(10):8655-8665.

89.	Mizukami IF, Todd RF, 3rd: **A soluble form of the urokinase plasminogen activator receptor (suPAR) can bind to hematopoietic cells**. *J Leukoc Biol* 1998, **64**(2):203-213.

90.	Kirkbride KC, Ray BN, Blobe GC: **Cell-surface co-receptors: emerging roles in signaling and human disease**. *Trends Biochem Sci* 2005, **30**(11):611-621.

91.	Kemp LE, Mulloy B, Gherardi E: **Signalling by HGF/SF and Met: the role of heparan sulphate co-receptors**. *Biochem Soc Trans* 2006, **34**(Pt 3):414-417.

92.	Patel VN, Knox SM, Likar KM, Lathrop CA, Hossain R, Eftekhari S, Whitelock JM, Elkin M, Vlodavsky I, Hoffman MP: **Heparanase cleavage of perlecan heparan sulfate modulates FGF10 activity during ex vivo submandibular gland branching morphogenesis**. *Development* 2007, **134**(23):4177-4186.

93.	McFall AJ, Rapraeger AC: **Identification of an adhesion site within the syndecan-4 extracellular protein domain**. *J Biol Chem* 1997, **272**(20):12901-12904.

94.	Tkachenko E, Rhodes JM, Simons M: **Syndecans: new kids on the signaling block**. *Circ Res* 2005, **96**(5):488-500.

95.	Su G, Blaine SA, Qiao D, Friedl A: **Shedding of syndecan-1 by stromal fibroblasts stimulates human breast cancer cell proliferation via FGF2 activation**. *J Biol Chem* 2007, **282**(20):14906-14915.

96.	Nikolova V, Koo CY, Ibrahim SA, Wang Z, Spillmann D, Dreier R, Kelsch R, Fischgrabe J, Smollich M, Rossi LH *et al*: **Differential roles for membrane-bound and soluble syndecan-1 (CD138) in breast cancer progression**. *Carcinogenesis* 2009, **30**(3):397-407.
