# Supplementary material for: A model to explain specific cellular communications and cellular harmony:- a hypothesis of coupled cells and interactive coupling molecules
Source: Theor Biol Med Model. 2014 Sep 14;11:40. doi: 10.1186/1742-4682-11-40 (PMC4237941; doi:10.1186/1742-4682-11-40)
Supplement: Additional file 1 — 1. “Terminology”. 2. “Change in Cellular Characteristics”. 3. Note 1 “The Roles of SCD versus ACD”. Note 2. “Categories of SCD and ACD”. Note 3 “Further Examples of these AsCD Categories”. Note 4 “Change of differentiation type.”. 4. “The Effects of Growth Factors and other Bioactive Molecules 5. Note 1, “Additional Information on the Definition of a Trefone”. Note 2 “Clarification on Ligand and Binding Protein Interaction”. Note 3 “Cell Couplets – AsCD and the a-Cell and i-Cell”. Note 4 “Drosophila – Potential Trefones and ACD”. 6. “Calculated Levels of Trefones and TCC, with Cellular Actions”. 7. “Receptors for the Six IGFBPs and Cellular Effects”. 8. Note 1 “Extracellular Binding Proteins/ Soluble Receptors”. Note 2 “Cell Receptors for Binding Proteins/Soluble Receptors”. 9. Note 1. “Further Evidence for a Receptor for TCC”. Note 2 “Problems with Cross-linking Experiments”. 10. “Evidence for Binary Receptors aside from the IGF System”. 11. “Evidence for Two Receptors for a Particular Trefone, outside the IGF System”. 12. Note 1 “Mechanisms of Nuclear Localization”. Note 2 “NL of IGFBPs”. Note 3 “NL of Potential Trefones other than the IGF System”. 13. “Evidence Supporting the Existence of a-Cells and i-Cells”. 14. “Candidates with mainly a-Cell-Type or i-Cell-Type Characteristics”. 15. “Interacting Cells and Trefones not associated with the IGF System”. 16. “Heterogeneity/Variability of Cells in Culture.” 17. “Cell lines are not Typical of Normal Cells”. 18. “Cell Receptors and Cell Markers”. 19. “Defined Couplet Cells for Insulin and Glucagon; Gastrin and Histamine”. 20. “Potential Couplet Trefones”. 21. “Further Examples of Potential Trefone and Cell Couplets”. 22. “Examples of Cellular Regulation by Complexes”. 23. “Proteolytic enzymes and their inhibitors”. 24. “Expanded Definition of ‘Trefone’ and Classes of Couplet Cell Interactions”. 25. Note 1 “Extended Trefone Couplets”. Note 2 “Singlet Cells”. 26. “Background of Cancer Research”. [file 1742-4682-11-40-S1.zip › 3484671921331230_add1/3484671921331230_add09.doc]

### **Additional File** ***Nine***

***(i) The IGF System - Receptors for the binary complexes IGFI/II:IGFBPn (*TCC)**

***Note 1. Further Evidence for the Existence of a Receptor for TCC***

*The purpose of this Note is to support the CTC(EC) model which requires an extracellular membrane receptor for the complex of IGF-I and IGFBPn.*

There is evidence that TCC does not share a receptor with the IGFBPn. The fact that the putative integrin receptor for IGFBP-2 does not bind IGFBP-2:IGFI/II complexes would suggest that a TCC receptor would be distinct from the IGFBP receptor. However, there is evidence of a TCC receptor for IGFBPn:IGFI/II that requires IGFBP to be bound to a receptor before this binary complex forms a ternary complex with IGF-I. The fact that exogenous IGF-I binds to an IGFBPn which is on the cell-surface membranes of various cells {eg. IGFBP-3 bound to fibroblasts; IGFBP-2 bound to neuroblastoma cells} in labeled-IGF-I cross-linking studies, is a strong indication that a receptor capable of binding both the IGF-I and an IGFBP complex exists on the cell membrane. A receptor molecule could therefore be present on the cell, capable of binding the complex, although sequential binding may be required; IGFBP binds first, then IGF-I. This would be acceptable within the CTC(EC) model as the amount of complex bound would still reflect the relative amounts of the two Trefones. Similarly, the existence of a TCC receptor has been offered as an explanation for the increased binding of IGF to bone cells. With cell monolayers of a breast cancer cell line (T-47D), preincubation with IGFBP-3 enhanced binding of IGF-I to the membrane, again indicating that membrane-bound IGFBP-3 was able to bind IGF-I to form a ternary complex of TCC and receptor.

In addition, with cell monolayers of a different breast cancer cell line (MCF-1), the IGFBP-3:IGF-I complex induced a larger intracellular effect than did IGFBP-3 alone in a system where IGF-1 produced no change. Further, **e**vidence that the binding of IGFBP-2 to heparin and to the extracellular matrix of fibroblasts only occurs if an IGF is present, adds to the concept of a TCC receptor. But the direct evidence is that megakaryocytes internalize the IGF-I:IGFBP-3 complex and package it into the alpha-granules of platelets.

### An IGF-I:IGFBP complex, chemically cross-linked, did show biological activity in endothelial cells as indicated by the stimulation of glucose and alpha-aminoisobutyric acid uptake and, because the TCC did not bind the IGF-I receptor, a separate receptor for the TCC was considered feasible.

### So, the idea of a receptor for the binary complex is not new but remains unproven. Attempts to identify positively a receptor for the IGF-I:IGFBP-3 complex may have failed because of technical reasons. If the IGF is first bound to IGFBP by the crosslinker DSS (disuccinimidyl suberate), then the amino groups of the TCC may not be available for subsequent cross-linking reactions with the putative receptor using the same reagent.

### Previous attempts to identify a TCC receptor had also failed – maybe because of the reasons offered above or because the TCC receptor on the membrane of a specific cell line will be specific for a particular IGF:IGFBP complex. For the latter, the binding will be determined by the presence of either IGF-I or IGF-II and the presence of one specific IGFBP of the six possible, giving 12 possible TCC receptors. Some such specificity is seen between IGFBP-4 and IGFBP-5 with IGF-I and IGF-II in the myogenic response to these Trefones.

***Note 2. Problems with Cross-linking Experiments***

*The purpose of this Note is to contest the lack of evidence for a TCC receptor.*

###  Cross-linking to detect a ternary complex of IGF-I:IGFBP-3 with a cell membrane receptor using the homobifunctional crosslinker DSS (disuccinimidyl suberate) has a number of problems.

**Heterogeneous products with DSS**

In the formation of a binary complex of IGF-I:IGFBP-3 using DSS, this reagent cross-links via amino groups and the products would be very heterogeneous. IGF-I has three Lys residues plus an N-terminal Gly to supply four amino groups for cross-linking while IGFBP-3 has 19 Lys plus N-terminal Ala for linking. After treatment of IGF-I plus IGFBP-3 with DSS, then potentially a number of cross-linked products could form. IGF-I potentially could form 1-4 cross-links with any of the 20 reactive amino groups of BP-3. On the other hand, every possible combination of cross-linked amino pairs could not happen because of the physical constraints within the binary complex itself and after the first and possible subsequent bond(s) are formed. Further, 13 of the 19 lysine residues of BP-3 are near or within the thyroglobulin type 1 domain which contains three close disulphide bonds (residues 213-240, 251-262, 264-285), perhaps restricting the availability of each Є-amino groups to the cross-linking reagent and to close neighboring lysine residues. There are also five glycosylation sites in this domain to restrict further the spatial access requirements of the 368-MW DSS reagent (ie. more than the equivalent of the total weight of three average amino-acid residues). Overall, then potentially the products would be very heterogeneous but in practice, there may be only a few products. Perhaps larger cross-linked products occur as the reaction of IGFBP-3 with DSS produces some dimers of the IGFBP.

Similar heterogeneous products may occur with other reagents eg. in biotinylation reactions.

**Deficiency of amino groups for reaction**

In the formation of the binary complex of IGF:IGFBPn, all of the exposed, outer amino groups may have reacted with each other ie. may be totally involved in links already. In any attempt to detect then a ternary complexes of IGF-I:IGFBP-3 bound to the proposed TCC receptor by addition of the same reagent (DSS) to the binary complex incubated with cell membranes, there may few, if any, amino groups within the binary complex available to cross-link with the TCC receptor. Even if available for reaction, residual amino groups may be hidden within the ternary complex or not be physically close enough or spatially orientated correctly to form cross-links.

**Complication introduced by the label**

If the IGFBP-3 were first labelled by biotinylation using D-biotinoyl-ε-aminocaproic acid-N-hydroxysuccinimide ester (biotin-7-NHS), which also reacts with amino groups, then, again, there could be very few unreacted lysines remaining on the biotinylated IGFBP-3 to react with DSS in cross-linking to IGF-I or receptor.

In a similar way, the presence of an atom or two of iodine (additional weight of 126 or 252 Da) on potentially each of three tyrosine residues (potential additional weight of up to 756 Da) of a molecule of 70 amino-acid residues (MW 7649 Da), may influence the manner of binding of a radioactively labelled species of IGF when interacting alone or in a binary complex. Radio-iodinated IGFBP-3, with nine tyrosine residues in a molecule of 264 amino-acid residues, has been found not to be internalized into cells . Normal IGFBP-3 is internalized.

**Altered tertiary structure**

Within the products from the DSS reaction, maybe none would bind to the proposed receptor for the TCC complex. Perhaps none would retain the same 3D spatial arrangement of the binding site as the natural binary complex so as to be able to bind to the receptor and to produce the ternary complex. The cross-linking may fix the 3D structure such that it no longer has the flexible conformation to adopt the energetically favorable orientation needed to fit the receptor. The label of biotin or iodine may further complicate possible binding.

These potential deficiencies in methods for detection of TCC receptors may explain why evidence of their existence is lacking. Also, given that the level of TCC may be 100 times that of a Trefone, fewer TCC receptor molecules would be needed to sample the extracellular environment. More sensitive methods may be needed.

**REFERENCES:-**

1.	Schutt BS, Langkamp M, Rauschnabel U, Ranke MB, Elmlinger MW: **Integrin-mediated action of insulin-like growth factor binding protein-2 in tumor cells**. *J Mol Endocrinol* 2004, **32**(3):859-868.

2.	Russo VC, Rekaris G, Baker NL, Bach LA, Werther GA: **Basic fibroblast growth factor induces proteolysis of secreted and cell membrane-associated insulin-like growth factor binding protein-2 in human neuroblastoma cells**. *Endocrinology* 1999, **140**(7):3082-3090.

3.	Mohan S, Nakao Y, Honda Y, Landale E, Leser U, Dony C, Lang K, Baylink DJ: **Studies on the mechanisms by which insulin-like growth factor (IGF) binding protein-4 (IGFBP-4) and IGFBP-5 modulate IGF actions in bone cells**. *Journal of Biological Chemistry* 1995, **270**(35):20424-20431.

4.	Mishra S, Murphy LJ: **Phosphorylation of insulin-like growth factor (IGF) binding protein-3 by breast cancer cell membranes enhances IGF-I binding**. *Endocrinology* 2003, **144**(9):4042-4050.

5.	Ricort JM, Lombet A, Lassarre C, Binoux M: **Insulin-like growth factor binding protein-3 increases intracellular calcium concentrations in MCF-7 breast carcinoma cells**. *FEBS Lett* 2002, **527**(1-3):293-297.

6.	Arai T, Busby W, Jr., Clemmons DR: **Binding of insulin-like growth factor (IGF) I or II to IGF-binding protein-2 enables it to bind to heparin and extracellular matrix**. *Endocrinology* 1996, **137**(11):4571-4575.

7.	Chan K, Spencer EM: **Megakaryocytes endocytose insulin-like growth factor (IGF) I and IGF-binding protein-3: a novel mechanism directing them into alpha granules of platelets**. *Endocrinology* 1998, **139**(2):559-565.

8.	Booth BA, Bar RS, Boes M, Dake BL, Bayne M, Cascieri M: **Intrinsic bioactivity of insulin-like growth factor-binding proteins from vascular endothelial cells**. *Endocrinology* 1990, **127**(6):2630-2638.

9.	Ewton DZ, Coolican SA, Mohan S, Chernausek SD, Florini JR: **Modulation of insulin-like growth factor actions in L6A1 myoblasts by insulin-like growth factor binding protein (IGFBP)-4 and IGFBP-5: a dual role for IGFBP-5**. *J Cell Physiol* 1998, **177**(1):47-57.

10.	Oh Y, Muller HL, Pham H, Rosenfeld RG: **Demonstration of receptors for insulin-like growth factor binding protein-3 on Hs578T human breast cancer cells**. *J Biol Chem* 1993, **268**(35):26045-26048.

11.	Paharkova-Vatchkova V, Lee KW: **Nuclear export and mitochondrial and endoplasmic reticulum localization of IGF-binding protein 3 regulate its apoptotic properties**. *Endocr Relat Cancer* 2010, **17**(2):293-302.
