# Supplementary material for: A model to explain specific cellular communications and cellular harmony:- a hypothesis of coupled cells and interactive coupling molecules
Source: Theor Biol Med Model. 2014 Sep 14;11:40. doi: 10.1186/1742-4682-11-40 (PMC4237941; doi:10.1186/1742-4682-11-40)
Supplement: Additional file 1 — 1. “Terminology”. 2. “Change in Cellular Characteristics”. 3. Note 1 “The Roles of SCD versus ACD”. Note 2. “Categories of SCD and ACD”. Note 3 “Further Examples of these AsCD Categories”. Note 4 “Change of differentiation type.”. 4. “The Effects of Growth Factors and other Bioactive Molecules 5. Note 1, “Additional Information on the Definition of a Trefone”. Note 2 “Clarification on Ligand and Binding Protein Interaction”. Note 3 “Cell Couplets – AsCD and the a-Cell and i-Cell”. Note 4 “Drosophila – Potential Trefones and ACD”. 6. “Calculated Levels of Trefones and TCC, with Cellular Actions”. 7. “Receptors for the Six IGFBPs and Cellular Effects”. 8. Note 1 “Extracellular Binding Proteins/ Soluble Receptors”. Note 2 “Cell Receptors for Binding Proteins/Soluble Receptors”. 9. Note 1. “Further Evidence for a Receptor for TCC”. Note 2 “Problems with Cross-linking Experiments”. 10. “Evidence for Binary Receptors aside from the IGF System”. 11. “Evidence for Two Receptors for a Particular Trefone, outside the IGF System”. 12. Note 1 “Mechanisms of Nuclear Localization”. Note 2 “NL of IGFBPs”. Note 3 “NL of Potential Trefones other than the IGF System”. 13. “Evidence Supporting the Existence of a-Cells and i-Cells”. 14. “Candidates with mainly a-Cell-Type or i-Cell-Type Characteristics”. 15. “Interacting Cells and Trefones not associated with the IGF System”. 16. “Heterogeneity/Variability of Cells in Culture.” 17. “Cell lines are not Typical of Normal Cells”. 18. “Cell Receptors and Cell Markers”. 19. “Defined Couplet Cells for Insulin and Glucagon; Gastrin and Histamine”. 20. “Potential Couplet Trefones”. 21. “Further Examples of Potential Trefone and Cell Couplets”. 22. “Examples of Cellular Regulation by Complexes”. 23. “Proteolytic enzymes and their inhibitors”. 24. “Expanded Definition of ‘Trefone’ and Classes of Couplet Cell Interactions”. 25. Note 1 “Extended Trefone Couplets”. Note 2 “Singlet Cells”. 26. “Background of Cancer Research”. [file 1742-4682-11-40-S1.zip › 3484671921331230_add1/3484671921331230_add10.doc]

**Additional File** ***Ten***

***(ii) Other ICM Systems – Receptors for Other Binary Complexes***

***Evidence for Binary Receptors aside from the IGF System***

*The purpose of this SI is to support the concept of a potential extracellular receptor for the binary complex TCC, by providing evidence of receptors for other binary complexes and even for ternary complexes.*

In certain bacteria (*Escherishia coli* and *Salmonella typhimurium*) in the chemosensory two-component pathway, ligands (eg. maltose, galactose, glucose, ribose, dipeptide) form a complex with specific soluble binding proteins in the periplasm and the binary complexes are bound by specific transmembrane receptors to activate intracytoplasmic responses. These receptors would be the equivalent of a TCC receptor

Good evidence for such a receptor also comes from **Interleukin-6** (IL-6). IL-6, IL-11 and ciliary neurotrophic factor (CNTF), when bound to their respective soluble receptors as a TCC, become associated with gp130 - a signal-transducing “co-receptor”. For IL-6, the extracellular complex of itself and soluble IL-6 receptor (IL-6R) can interact with this membrane glycoprotein (gp130) in a myeloma cell line and can mediate the IL-6 signal. In addition, IL-6R with IL-6, stimulates the generation of megakaryocytes. Further, endothelial cells (HUVEC) do not express the subunit-specific IL-6R and are therefore unresponsive to IL-6 but they express gp130 and are responsive to a combination of IL-6 and soluble IL-6R. The response includes clear activation of the transcription factor STAT3, but no significant change in proliferation. However a fusion protein of sIL-6R and IL-6, linked by a flexible peptide chain, was fully active and increased proliferation at 100- to 1,000-fold lower concentrations than the combination of unlinked IL-6 and IL-6R. Thus gp130 could be considered to be a receptor for the complex of the two Trefones, the TCC ie. the gp130 would be a TCC receptor. Further, the soluble IL-6-R is generated by one cell type and the complex stimulates different cell types which have gp130 but which lack membrane IL-6R. This has been referred to as trans-signalling but is consistent with the current CTC model where an i-Cell would have the gp130 but lack the IL-6R and the a-Cell would secrete the soluble IL-6R and have a membrane IL-6R.

The situation with **granulocyte-macrophage colony-stimulating factor (GM-CSF)** can also be interpreted as a Trefone couplet. The receptor for GM-CSF (GMR) has an alpha and a beta subunit; a binary complex of GM-CSF and a soluble isoform of the alpha subunit of the receptor for GM-CSF (GMR) interact with a soluble form of the beta subunit of GMR to form a ternary complex. However, the interactions are complicated. Receptor beta is shared with IL-3 and IL-5 and similar effects may occur with them.

**Fibroblast growth factor** binds a soluble form of the FGF receptor to produce a binary complex which then binds to heparin to form a ternary complex; no soluble FGF receptor was bound to immobilized heparin unless FGF-2 was added to the binding medium. Different sequences of reaction could occur in the formation of a ternary complex**,** but within the current CTC model, a heparin-bearing receptor would bind the complex of the two Trefones - one FGF, the other the soluble receptor. Indeed, soluble FGF receptor requires FGF for stable heparin interaction and the crystalline structure of a ternary FGF-‘FGFR’-heparin complex has been examined (although the ‘FGFR’ was only two-thirds of the extracellular ligand binding portion). While a proteoglycan (PG) containing the [glycosaminoglycan](http://en.wikipedia.org/wiki/Glycosaminoglycan) heparan sulphate, HS, (eg. perlecan, a HSPG) may have functions within the extracellular matrix by storing ICMs or by the heparanase-release of bioactive fragments, transmembrane HSPGs (eg. [syndecans](http://en.wikipedia.org/wiki/Syndecans)) and the [glycosylphosphatidylinositol](http://en.wikipedia.org/wiki/Glycosylphosphatidylinositol) (GPI) anchored [glypicans](http://en.wikipedia.org/wiki/Glypicans), have receptor functions. Syndecan-1 is known to forms complexes with growth factors and their cognate receptors. For example, syndecan-1, FGF-2 and FGFR-1 form a ternary complex which is internalized and syndecan-1 and FGF-2 co-localize in the nucleus/perinuclear area via a tubulin complex; the FGFR-1 does not. The extrapolation for the CTC model would be that membrane syndecan would also be a receptor for a FGF bound to soluble FGF receptor, to form the ternary complex.

Either the protein core or the heparan sulphate chains of a membrane-bound HSPG may contain specific binding sites for Trefones leading to their internalization. While a HSPG may act as a receptor for peptides and heparin can activate a protein kinase by acting on FGF-4R independent of FGF, its function in FGF internalization may be more as a co-receptor acting with FGF to bind and activate the FGFR.

Human SHBG complexed with an **androgen** (testosterone or dihydrotestosterone) binds specifically to the plasma membrane of human placental syncytiotrophoblasts. The receptor binds the binary complex but not SHBG if it is devoid of an androgen. Similarly, for decidual endometrium membranes, SHBG complexed with estradiol (but not to testosterone) is bound, but not unliganded SHBG. However, in a prostate cancer cell line, both SHBG-dihydrotestosterone and SHBG-estradiol can regulate growth, indicating the likelihood of a receptor or receptors for both binary complexes.

In a slight contrast, the binding trio of an **estrogen**, sex-hormone binding globulin (SHBG) and a solubilised membrane receptor form a ternary complex; estrogen binds to the pre-complexed receptor:SHBG complex, but the estrogen:SHBG complex does not bind the receptor. Megalin **(**lipoprotein-related protein 2**)** an endocytic receptor, acts as a pathway for cellular uptake of biologically active androgens and estrogens bound to SHBG.

The corticosteroid-binding globulin (CBG) binds **cortisol** and most cells internalize the steroid by itself. But some cells also internalize the binary complex of steroid with CBG indicating a receptor for the complex.

Thyroxine binding globulin (TBG) binds **thyroxine** (T_4_) and is bound by receptors on hepatoma cells (Hep G2); it is suggested that internalization of the T_4_:TBG:TBG receptor complex occurs. Similarly, transthyretin (TTR) is bound by receptors on human peripheral mononuclear cells and it is suggested that internalization of the T_4_:TTR:TTR receptor complex occurs.

Another example of the importance of a binding protein relates to the **corticotropin-releasing factor** (CRF). While CRF can stimulate or inhibit cell proliferation, its activity (as described next) as a transmitter, is enhanced by a binding protein. CRF induces a potentiation of N-methyl-D-aspartate receptor-mediated synaptic transmission in dopamine neurons via a CRF receptor and an associated kinase C. But this potentiation requires the CRF binding protein indicating probable interaction of the binary complex (CRF:CRFBP)**.**

Another example is **vitamin A.** The complex of vitamin A and the retinol-binding protein binds a receptor and is ten-times as efficient at blocking the terminal differentiation of cultured normal human keratinocytes as is the free Vit A.

Also, the complex of **prostate-specific antigen** (PSA) and the activated alpha_2_-macroglobulin (alpha_2_M) is bound by the cell surface GRP78 chaperone and causes an increase in the activation of intracellular pathway kinases and of DNA and protein synthesis. PSA and alpha_2_M could be Trefones, and consistent with the CTC model, the binding of activated alpha_2_M stimulates PSA secretion in two prostate cancer cell lines, although the receptor is again GRP78.

There are many other examples which are listed in **Table S1.**

**TABLE S1**

**Information-Carrying Molecules (ICMs), Binary Complexes and their Receptors**

| **Group** | **Ligand or Source** | **Complex with** | **Receptor(s) for the Complex &/or Investigated Cell Reference** |
| --- | --- | --- | --- |
| Trefone/  hormone-  related | TGFs-beta, TNF-alpha and IL-1 | α_2_-Macroglobulin | Low density lipoprotein receptor-related protein (LRP). /α_2_-macroglobulin receptor (α_2_MR) (LRP/α_2_-MR); Hepatic |
|  | Prolactin | Immunoglobulin G | PRL and Fc receptors; Chronic B-cell lymphocytic leukemia, Lymphocytes |
|  | EGF Binding protein | Protease nexin II | LRP/α_2_-MR |
|  | Testosterone | Androgen binding protein | Epididymal cells |
|  | Cortisol | Transcortin | Liver cells |
|  | Endostatin | Nucleolin and integrin α5β1 | Complex of ligand, nucleolin, integrin α_5_β_1_ and uPAR;  Endothelial cells |
| Vitamin-related | Biotin | Biotin-Binding Protein | Oocyte vitelline membrane  receptor |
|  | Folate | Folate Binding Protein | Megalin Complex**; Renal epithelia |
|  | Riboflavin | Riboflavin Carrier Protein | ? A G-protein, not megalin; Placental trophoblasts |
|  | Vit A | Retinol Binding Protein | Megalin; Keratinocytes, Transepithelial |
|  | Vit B_12_ | Transcobalamin (TCbl) | Megalin Complex;Epithelia. TCbl receptor |
|  |  | Intrinsic Factor | Cubulin/Megalin Complex; Renal epithelia |
|  | Vit D | Vit D Binding Protein | Megalin Complex; Renal epithelia |
| Pheromones | Silk moth  (Antheraea) | Pheromone Binding Protein (PBP) | A G-protein Coupled 7TM Domain Receptor |
|  | Silk moth (*Bombyx*) | PBP^##^ | Undetermined |
|  | Friuit fly  (*Drosophila*) | Odorant Binding Protein (OBP) (LUSH) | Undetermined |
|  | Mouse olfactory epithelium | OBP | Megalin |
| Protease or related proteins | Uurokinase-type plasminogen activator (uPA) | Plasminogen activator inhibitor type-1 (PAI-1) | LRP/α_2_-MR |
|  | uPA: PAI-1 | Urokinase plasmin-ogen activator receptor (uPAR) | LRP/α_2_-MR. uPAR itself is internalised and this requires LRP/ α_2_-MR. |
|  | uPA:soluble uPAR | Vitronectin | ? α_v,_β_3_ integrin; Smooth muscle cells; umbilical vein endothelial cells |
|  | Trypsin, elastase | α_2_-Macroglobulin | Undetermined |
|  | Prostatic specific antigen (PSA) | α_2_-Macroglobulin | GRP78 (surface chaperone) |
| Proteins | Beta amyloid | α_2_-Macroglobulin | LRP/α_2_-MR |
|  | Wnt | Frizzled | LRP5/6 |
|  | Hemoglobin | Haptoglobulin | CD163 |
|  | Heme | Hemopexin | LRP/α_2_-MR |
| Small molecules | Iron | Transferrin | Transferrin receptor |
|  | Fatty acids (FAs) | FA binding protein | CD36; Muscle |
|  | Antigen | Antibody | Fc gamma, alpha or epsilon |
|  | Lipopolysaccharide (LPS) | Myeloid differentiation protein 2 | Monocytes Toll-like receptor 4 (TLR4) |
| Other enzymes | Lecithin:cholesterol acyltransferase (LCAT) | α_2_-Macroglobulin | LRP/α_2_-MR |

*The* *Table lists the receptors or cells that bind various ICMs when the particular ICM is bound in a binary or ternary complex with substances that are usually proteins.*

*** The Megalin Complex is the group of proteins needed for the binding and endocytosis of the ligand megalin. Megalin {or low density lipoprotein-related protein 2 (LRP2)} is complexed with the receptor-associated protein or adaptor protein disabled-2 (Dab2) plus amnionless and cubulin (perhaps). (Since cubilin binds to megalin but is a peripheral membrane protein with no endocytosis signalling sequence, it appears that megalin is responsible for cubilin’s internalization and that of its ligands, in addition to internalizing its own ligands.)*

*^##^ The Pheromone Binding Proteins and the Odorant Binding Proteins are specific to the individual pheromones.*

One conclusion from this listing is that these ICMs should be viewed as potential Trefones as they have a binding partner and a potential TCC receptor.

**REFERENCES:-**

1.	Falke JJ, Bass RB, Butler SL, Chervitz SA, Danielson MA: **The two-component signaling pathway of bacterial chemotaxis: a molecular view of signal transduction by receptors, kinases, and adaptation enzymes**. *Annu Rev Cell Dev Biol* 1997, **13**:457-512.

2.	Rose-John S: **IL-6 Trans-Signaling via the Soluble IL-6 Receptor: Importance for the Pro-Inflammatory Activities of IL-6**. *Int J Biol Sci* 2012, **8**(9):1237-1247.

3.	Taga T, Hibi M, Hirata Y, Yamasaki K, Yasukawa K, Matsuda T, Hirano T, Kishimoto T: **Interleukin-6 triggers the association of its receptor with a possible signal transducer, gp130**. *Cell* 1989, **58**(3):573-581.

4.	Sui X, Tsuji K, Ebihara Y, Tanaka R, Muraoka K, Yoshida M, Yamada K, Yasukawa K, Taga T, Kishimoto T *et al*: **Soluble interleukin-6 (IL-6) receptor with IL-6 stimulates megakaryopoiesis from human CD34(+) cells through glycoprotein (gp)130 signaling**. *Blood* 1999, **93**(8):2525-2532.

5.	Romano M, Sironi M, Toniatti C, Polentarutti N, Fruscella P, Ghezzi P, Faggioni R, Luini W, van Hinsbergh V, Sozzani S *et al*: **Role of IL-6 and its soluble receptor in induction of chemokines and leukocyte recruitment**. *Immunity* 1997, **6**(3):315-325.

6.	Fischer M, Goldschmitt J, Peschel C, Brakenhoff JP, Kallen KJ, Wollmer A, Grotzinger J, Rose-John S: **I. A bioactive designer cytokine for human hematopoietic progenitor cell expansion**. *Nat Biotechnol* 1997, **15**(2):142-145.

7.	Rose-John S, Heinrich PC: **Soluble receptors for cytokines and growth factors: generation and biological function**. *Biochem J* 1994, **300 ( Pt 2)**:281-290.

8.	Prevost JM, Farrell PJ, Iatrou K, Brown CB: **Determinants of the functional interaction between the soluble GM-CSF receptor and the GM-CSF receptor beta-subunit**. *Cytokine* 2000, **12**(3):187-197.

9.	Ornitz DM, Yayon A, Flanagan JG, Svahn CM, Levi E, Leder P: **Heparin is required for cell-free binding of basic fibroblast growth factor to a soluble receptor and for mitogenesis in whole cells**. *Mol Cell Biol* 1992, **12**(1):240-247.

10.	Pantoliano MW, Horlick RA, Springer BA, Van Dyk DE, Tobery T, Wetmore DR, Lear JD, Nahapetian AT, Bradley JD, Sisk WP: **Multivalent ligand-receptor binding interactions in the fibroblast growth factor system produce a cooperative growth factor and heparin mechanism for receptor dimerization**. *Biochemistry* 1994, **33**(34):10229-10248.

11.	Gao G, Goldfarb M: **Heparin can activate a receptor tyrosine kinase**. *Embo J* 1995, **14**(10):2183-2190.

12.	Schlessinger J, Plotnikov AN, Ibrahimi OA, Eliseenkova AV, Yeh BK, Yayon A, Linhardt RJ, Mohammadi M: **Crystal structure of a ternary FGF-FGFR-heparin complex reveals a dual role for heparin in FGFR binding and dimerization**. *Mol Cell* 2000, **6**(3):743-750.

13.	Patel VN, Knox SM, Likar KM, Lathrop CA, Hossain R, Eftekhari S, Whitelock JM, Elkin M, Vlodavsky I, Hoffman MP: **Heparanase cleavage of perlecan heparan sulfate modulates FGF10 activity during ex vivo submandibular gland branching morphogenesis**. *Development* 2007, **134**(23):4177-4186.

14.	Zong F, Fthenou E, Wolmer N, Hollosi P, Kovalszky I, Szilak L, Mogler C, Nilsonne G, Tzanakakis G, Dobra K: **Syndecan-1 and FGF-2, but not FGF receptor-1, share a common transport route and co-localize with heparanase in the nuclei of mesenchymal tumor cells**. *PLoS One* 2009, **4**(10):e7346.

15.	McFall AJ, Rapraeger AC: **Identification of an adhesion site within the syndecan-4 extracellular protein domain**. *J Biol Chem* 1997, **272**(20):12901-12904.

16.	Tkachenko E, Rhodes JM, Simons M: **Syndecans: new kids on the signaling block**. *Circ Res* 2005, **96**(5):488-500.

17.	Bernfield M, Gotte M, Park PW, Reizes O, Fitzgerald ML, Lincecum J, Zako M: **Functions of cell surface heparan sulfate proteoglycans**. *Annu Rev Biochem* 1999, **68**:729-777.

18.	Poon GM, Gariepy J: **Cell-surface proteoglycans as molecular portals for cationic peptide and polymer entry into cells**. *Biochem Soc Trans* 2007, **35**(Pt 4):788-793.

19.	Eswarakumar VP, Lax I, Schlessinger J: **Cellular signaling by fibroblast growth factor receptors**. *Cytokine Growth Factor Rev* 2005, **16**(2):139-149.

20.	Krupenko NI, Avvakumov GV, Strel'chyonok OA: **Binding of human sex hormone-binding globulin-androgen complexes to the placental syncytiotrophoblast membrane**. *Biochemical & Biophysical Research Communications* 1990, **171**(3):1279-1283.

21.	Strel'chyonok OA, Avvakumov GV, Survilo LI: **A recognition system for sex-hormone-binding protein-estradiol complex in human decidual endometrium plasma membranes**. *Biochim Biophys Acta* 1984, **802**(3):459-466.

22.	Nakhla AM, Rosner W: **Stimulation of prostate cancer growth by androgens and estrogens through the intermediacy of sex hormone-binding globulin**. *Endocrinology* 1996, **137**(10):4126-4129.

23.	Hryb DJ, Khan MS, Romas NA, Rosner W: **The control of the interaction of sex hormone-binding globulin with its receptor by steroid hormones**. *J Biol Chem* 1990, **265**(11):6048-6054.

24.	Hammes A, Andreassen TK, Spoelgen R, Raila J, Hubner N, Schulz H, Metzger J, Schweigert FJ, Luppa PB, Nykjaer A *et al*: **Role of endocytosis in cellular uptake of sex steroids**. *Cell* 2005, **122**(5):751-762.

25.	Rosner W, Hryb DJ, Khan MS, Singer CJ, Nakhla AM: **Are corticosteroid-binding globulin and sex hormone-binding globulin hormones?** *Annals of the New York Academy of Sciences* 1988, **538**:137-145.

26.	Hashizume K, Sakurai A, Miyamoto T, Yamauchi K, Nishii Y: **Effect of thyroxine-binding globulin (TBG) on thyroxine (T4) uptake by human peripheral mononuclear cells: evidence of TBG-dependent uptake of T4**. *Endocrinol Jpn* 1986, **33**(5):665-674.

27.	Divino CM, Schussler GC: **Receptor-mediated uptake and internalization of transthyretin**. *J Biol Chem* 1990, **265**(3):1425-1429.

28.	Ungless MA, Singh V, Crowder TL, Yaka R, Ron D, Bonci A: **Corticotropin-releasing factor requires CRF binding protein to potentiate NMDA receptors via CRF receptor 2 in dopamine neurons**. *Neuron* 2003, **39**(3):401-407.

29.	Bavik CO, Peterson A, Eriksson U: **Retinol-binding protein mediates uptake of retinol to cultured human keratinocytes**. *Exp Cell Res* 1995, **216**(2):358-362.

30.	Misra UK, Payne S, Pizzo SV: **Ligation of prostate cancer cell surface GRP78 activates a proproliferative and antiapoptotic feedback loop: a role for secreted prostate-specific antigen**. *J Biol Chem* 2011, **286**(2):1248-1259.

31.	LaMarre J, Wollenberg GK, Gonias SL, Hayes MA: **Cytokine binding and clearance properties of proteinase-activated alpha 2-macroglobulins**. *Lab Invest* 1991, **65**(1):3-14.

32.	Walker AM, Montgomery DW, Saraiya S, Ho TW, Garewal HS, Wilson J, Lorand L: **Prolactin-immunoglobulin G complexes from human serum act as costimulatory ligands causing proliferation of malignant B lymphocytes**. *Proc Natl Acad Sci U S A* 1995, **92**(8):3278-3282.

33.	Knauer DJ, Cunningham DD: **Epidermal growth factor carrier protein binds to cells via a complex with released carried protein nexin**. *Proc Natl Acad Sci U S A* 1982, **79**(7):2310-2314.

34.	Knauer MF, Orlando RA, Glabe CG: **Cell surface APP751 forms complexes with protease nexin 2 ligands and is internalized via the low density lipoprotein receptor-related protein (LRP)**. *Brain Res* 1996, **740**(1-2):6-14.

35.	Gueant JL, Fremont S, Felden F, Nicolas JP, Gerard A, Leheup B, Gerard H, Grignon G: **Evidence that androgen-binding protein endocytosis in vitro is receptor mediated in principal cells of the rat epididymis**. *J Mol Endocrinol* 1991, **7**(2):113-122.

36.	Strel'chyonok OA, Avvakumov GV: **Evidence for the presence of specific binding sites for transcortin in human liver plasma membranes**. *Biochim Biophys Acta* 1983, **755**(3):514-517.

37.	Song N, Ding Y, Zhuo W, He T, Fu Z, Chen Y, Song X, Fu Y, Luo Y: **The nuclear translocation of endostatin is mediated by its receptor nucleolin in endothelial cells**. *Angiogenesis* 2012, **15**(4):697-711.

38.	Vieira AV, White HB, 3rd, Vieira PM: **An oocytic membrane receptor for biotin-binding protein**. *FEBS Lett* 1996, **382**(1-2):183-185.

39.	Birn H, Zhai X, Holm J, Hansen SI, Jacobsen C, Christensen EI, Moestrup SK: **Megalin binds and mediates cellular internalization of folate binding protein**. *Febs J* 2005, **272**(17):4423-4430.

40.	Mason CW, D'Souza VM, Bareford LM, Phelps MA, Ray A, Swaan PW: **Recognition, co-internalization, and recycling of an avian riboflavin carrier protein in human placental trophoblasts**. *J Pharmacol Exp Ther* 2006, **317**(2):465-472.

41.	Christensen EI, Moskaug JO, Vorum H, Jacobsen C, Gundersen TE, Nykjaer A, Blomhoff R, Willnow TE, Moestrup SK: **Evidence for an essential role of megalin in transepithelial transport of retinol**. *J Am Soc Nephrol* 1999, **10**(4):685-695.

42.	Moestrup SK, Birn H, Fischer PB, Petersen CM, Verroust PJ, Sim RB, Christensen EI, Nexo E: **Megalin-mediated endocytosis of transcobalamin-vitamin-B12 complexes suggests a role of the receptor in vitamin-B12 homeostasis**. *Proc Natl Acad Sci U S A* 1996, **93**(16):8612-8617.

43.	Quadros EV, Nakayama Y, Sequeira JM: **The protein and the gene encoding the receptor for the cellular uptake of transcobalamin-bound cobalamin**. *Blood* 2009, **113**(1):186-192.

44.	Birn H, Verroust PJ, Nexo E, Hager H, Jacobsen C, Christensen EI, Moestrup SK: **Characterization of an epithelial approximately 460-kDa protein that facilitates endocytosis of intrinsic factor-vitamin B12 and binds receptor-associated protein**. *J Biol Chem* 1997, **272**(42):26497-26504.

45.	Nykjaer A, Dragun D, Walther D, Vorum H, Jacobsen C, Herz J, Melsen F, Christensen EI, Willnow TE: **An endocytic pathway essential for renal uptake and activation of the steroid 25-(OH) vitamin D3**. *Cell* 1999, **96**(4):507-515.

46.	Prestwich GD, Du G, LaForest S: **How is pheromone specificity encoded in proteins?** *Chem Senses* 1995, **20**(4):461-469.

47.	Pophof B: **Moth pheromone binding proteins contribute to the excitation of olfactory receptor cells**. *Naturwissenschaften* 2002, **89**(11):515-518.

48.	Grosse-Wilde E, Svatos A, Krieger J: **A pheromone-binding protein mediates the bombykol-induced activation of a pheromone receptor in vitro**. *Chem Senses* 2006, **31**(6):547-555.

49.	Zhou JJ, Zhang GA, Huang W, Birkett MA, Field LM, Pickett JA, Pelosi P: **Revisiting the odorant-binding protein LUSH of Drosophila melanogaster: evidence for odour recognition and discrimination**. *FEBS Lett* 2004, **558**(1-3):23-26.

50.	Strotmann J, Breer H: **Internalization of odorant-binding proteins into the mouse olfactory epithelium**. *Histochem Cell Biol* 2011, **136**(3):357-369.

51.	Cubellis MV, Wun TC, Blasi F: **Receptor-mediated internalization and degradation of urokinase is caused by its specific inhibitor PAI-1**. *Embo J* 1990, **9**(4):1079-1085.

52.	Nykjaer A, Petersen CM, Moller B, Jensen PH, Moestrup SK, Holtet TL, Etzerodt M, Thogersen HC, Munch M, Andreasen PA *et al*: **Purified alpha 2-macroglobulin receptor/LDL receptor-related protein binds urokinase.plasminogen activator inhibitor type-1 complex. Evidence that the alpha 2-macroglobulin receptor mediates cellular degradation of urokinase receptor-bound complexes**. *J Biol Chem* 1992, **267**(21):14543-14546.

53.	Conese M, Nykjaer A, Petersen CM, Cremona O, Pardi R, Andreasen PA, Gliemann J, Christensen EI, Blasi F: **alpha-2 Macroglobulin receptor/Ldl receptor-related protein(Lrp)-dependent internalization of the urokinase receptor**. *J Cell Biol* 1995, **131**(6 Pt 1):1609-1622.

54.	Chavakis T, Kanse SM, Yutzy B, Lijnen HR, Preissner KT: **Vitronectin concentrates proteolytic activity on the cell surface and extracellular matrix by trapping soluble urokinase receptor-urokinase complexes**. *Blood* 1998, **91**(7):2305-2312.

55.	Debanne MT, Bell R, Dolovich J: **Uptake of proteinase-alpha-macroglobulin complexes by macrophages**. *Biochim Biophys Acta* 1975, **411**(2):295-304.

56.	Ohlsson K, Laurell CB: **The disappearance of enzyme-inhibitor complexes from the circulation of man**. *Clin Sci Mol Med* 1976, **51**(1):87-92.

57.	Narita M, Holtzman DM, Schwartz AL, Bu G: **Alpha2-macroglobulin complexes with and mediates the endocytosis of beta-amyloid peptide via cell surface low-density lipoprotein receptor-related protein**. *J Neurochem* 1997, **69**(5):1904-1911.

58.	He X, Semenov M, Tamai K, Zeng X: **LDL receptor-related proteins 5 and 6 in Wnt/beta-catenin signaling: arrows point the way**. *Development* 2004, **131**(8):1663-1677.

59.	Kristiansen M, Graversen JH, Jacobsen C, Sonne O, Hoffman HJ, Law SK, Moestrup SK: **Identification of the haemoglobin scavenger receptor**. *Nature* 2001, **409**(6817):198-201.

60.	Hvidberg V, Maniecki MB, Jacobsen C, Hojrup P, Moller HJ, Moestrup SK: **Identification of the receptor scavenging hemopexin-heme complexes**. *Blood* 2005, **106**(7):2572-2579.

61.	Tsunoo H, Sussman HH: **Characterization of transferrin binding and specificity of the placental transferrin receptor**. *Arch Biochem Biophys* 1983, **225**(1):42-54.

62.	Zhang L, Keung W, Samokhvalov V, Wang W, Lopaschuk GD: **Role of fatty acid uptake and fatty acid beta-oxidation in mediating insulin resistance in heart and skeletal muscle**. *Biochim Biophys Acta* 2010, **1801**(1):1-22.

63.	Raghavan M, Bjorkman PJ: **Fc receptors and their interactions with immunoglobulins**. *Annu Rev Cell Dev Biol* 1996, **12**:181-220.

64.	Bonnerot C, Briken V, Amigorena S: **Intracellular signaling and endosomal trafficking of immunoreceptors. Shared effectors underlying MHC class II-restricted antigen presentation**. *Immunol Lett* 1997, **57**(1-3):1-4.

65.	Jerala R: **Structural biology of the LPS recognition**. *Int J Med Microbiol* 2007, **297**(5):353-363.

66.	Krimbou L, Marcil M, Davignon J, Genest J, Jr.: **Interaction of lecithin:cholesterol acyltransferase (LCAT).alpha 2-macroglobulin complex with low density lipoprotein receptor-related protein (LRP). Evidence for an alpha 2-macroglobulin/LRP receptor-mediated system participating in LCAT clearance**. *J Biol Chem* 2001, **276**(35):33241-33248.

67.	Christensen EI, Gburek J: **Protein reabsorption in renal proximal tubule-function and dysfunction in kidney pathophysiology**. *Pediatr Nephrol* 2004, **19**(7):714-721.
