# Supplementary material for: A model to explain specific cellular communications and cellular harmony:- a hypothesis of coupled cells and interactive coupling molecules
Source: Theor Biol Med Model. 2014 Sep 14;11:40. doi: 10.1186/1742-4682-11-40 (PMC4237941; doi:10.1186/1742-4682-11-40)
Supplement: Additional file 1 — 1. “Terminology”. 2. “Change in Cellular Characteristics”. 3. Note 1 “The Roles of SCD versus ACD”. Note 2. “Categories of SCD and ACD”. Note 3 “Further Examples of these AsCD Categories”. Note 4 “Change of differentiation type.”. 4. “The Effects of Growth Factors and other Bioactive Molecules 5. Note 1, “Additional Information on the Definition of a Trefone”. Note 2 “Clarification on Ligand and Binding Protein Interaction”. Note 3 “Cell Couplets – AsCD and the a-Cell and i-Cell”. Note 4 “Drosophila – Potential Trefones and ACD”. 6. “Calculated Levels of Trefones and TCC, with Cellular Actions”. 7. “Receptors for the Six IGFBPs and Cellular Effects”. 8. Note 1 “Extracellular Binding Proteins/ Soluble Receptors”. Note 2 “Cell Receptors for Binding Proteins/Soluble Receptors”. 9. Note 1. “Further Evidence for a Receptor for TCC”. Note 2 “Problems with Cross-linking Experiments”. 10. “Evidence for Binary Receptors aside from the IGF System”. 11. “Evidence for Two Receptors for a Particular Trefone, outside the IGF System”. 12. Note 1 “Mechanisms of Nuclear Localization”. Note 2 “NL of IGFBPs”. Note 3 “NL of Potential Trefones other than the IGF System”. 13. “Evidence Supporting the Existence of a-Cells and i-Cells”. 14. “Candidates with mainly a-Cell-Type or i-Cell-Type Characteristics”. 15. “Interacting Cells and Trefones not associated with the IGF System”. 16. “Heterogeneity/Variability of Cells in Culture.” 17. “Cell lines are not Typical of Normal Cells”. 18. “Cell Receptors and Cell Markers”. 19. “Defined Couplet Cells for Insulin and Glucagon; Gastrin and Histamine”. 20. “Potential Couplet Trefones”. 21. “Further Examples of Potential Trefone and Cell Couplets”. 22. “Examples of Cellular Regulation by Complexes”. 23. “Proteolytic enzymes and their inhibitors”. 24. “Expanded Definition of ‘Trefone’ and Classes of Couplet Cell Interactions”. 25. Note 1 “Extended Trefone Couplets”. Note 2 “Singlet Cells”. 26. “Background of Cancer Research”. [file 1742-4682-11-40-S1.zip › 3484671921331230_add1/3484671921331230_add11.doc]

**Additional File** ***Eleven***

***(iii) Two Receptors***

***Evidence for the Presence of Two Receptors for a Particular Trefone, outside the IGF System***

*The purpose of this File is to list those substances that have two or more receptors or binding sites on the cell membrane. This is to support the possibility that cells may have two similar receptors where one binds one Trefone and the other binds the complex of this Trefone and the other Trefone (ie. the TCC).*

If a cell type had a receptor for one Trefone and a receptor for the complex of that Trefone and another Trefone, then the receptor for the individual Trefone would have high affinity for that Trefone and perhaps the receptor for the complex would have a low affinity for that same individual Trefone. **Table S1** lists examples of cells with more than one receptor for a particular ICM.

**TABLE S1. Information-Carrying Molecules (ICMs) with Two or More Receptors**

| **ICM** | **Receptors** | **Cell Type &/or Notable Other; Reference** |
| --- | --- | --- |
| Corticosteroid binding globulin | Two transcortin receptor variants | Normal-plasma and pregnancy-associated transcortin receptors |
| Corticotropin releasing factor | CRF-R1 and CRF- R2, | Encoded by distinct genes; each with two alternatively spliced forms |
| Epidermal growth factor (EGF) | High- and low-affinity binding sites | Xenopus hepatocytes |
| Erythropoietin | Three EPO receptors | Hematopoietic cells |
| Gastrin | Four receptors | Variable binding to gastrin variants |
| Glucagon | High- and low-affinity binding sites | Hepatocytes |
| Glucagon | Two distinct receptors | Purified rat beta-cells |
| Histamine | H2- and H1-receptors | Possibly inhibitory and stimulatory pathways affected |
| IGF-I | Type 1 IGF-I receptor + a novel fetal receptor | Fetal type does not bind insulin |
| IGF-I/II | Alpha and beta subunit receptor subtypes | Hybrid receptors also |
| Insulin | High- and low-affinity binding sites | Fat cells |
| Insulin | Insulin receptor-related receptor | Islets of Langerhans |
| Interleukin-1 | Two receptors | Both bind IL-1α, IL-1β, IL-1ra |
| Interleukin-2 | Three: high-, low- and intermediate-affinity | Αβ- Dimer, α-monomer, β-monomer respectively |
| Kinins | B1 and B2 | Of the rhodopsin family of G- protein coupled receptors |
| Lactoferrin | High- and low-affinity binding sites | Neutrophilic polymorpho-nuclear leukocytes |
| α_2_-Macroglobulin | High- and low-affinity binding sites | In rheumatoid cells |
| Nerve Growth  Factor (and other Neurotrophins) | α_9_β_1_-Integrin, Neurotrophin receptor p75(NTR) | Colon cancer cell line  α_9_β_1-_ integrin is pro-survival and pro-proliferative |
| Neurotrophin-3 (NT-3) | High- and low-affinity receptors | Embryonic chick sensory neurons |
| Neurotensin | Two subtypes | Dopaminergic neurons |
| Neurokinin-1 | Full-length and truncated receptors on membrane | Monocyte/macrophage differentiation |
| Nicotinic acid | High- and low-affinity binding sites | Adipose tissue and spleen |
| Sex Hormone Binding Globulin | High- and low-affinity receptors | Prostate cells |
| Somatostatin | Six receptor subtypes | sst(1)-sst(5) plus sst(2A) and sst(2B) |
| Pituitary adenylate cyclase-activating polypeptide  (PACAP) | PACAP type I receptors  (Five forms) | Alternatively spliced |
| Thyroid hormone (T_4_ or T_3_) | High- and low-affinity binding sites for both | Hepatocytes |
| Transferrin-iron | High- and low-affinity binding sites | Mammary gland cell |
| Transforming Growth Factor-β | Types I, II, and III.  (RI, RII, RIII) | Mink lung and adenocarcinomic alveolar epithelial cell lines |
| Tumour Necrosis Factor (TNF) | TNFp55R (55 kDa type-1 TNFR) and TNFp75R (75 kDa type-2 TNFR | Proteolytic cleavage of both occurs |
| Vascular endothelial growth factor (VEGF) | VEGFR-1 and VEGFR-2 | Also called Flt-1 and KDR/Flk-1 (Splice variants) |
| Vasoactive intestinal peptide | PACAP type II receptors | Also called VIP1 and VIP2 receptors |

*Information in Table S1 shows some cells with a mixture of receptors with high- and low-affinity binding sites for a particular ICM and some cells with between two and six receptors for a particularICM.*

It would be expected that some of these ICMs would be Trefones. The presence of two or more receptors or receptor sites for these ICMs is then not inconsistent with the current model which requires one receptor for a Trefone and another (perhaps a related) receptor for the binary complex (TCC).

**REFERENCES:-**

1.	Avvakumov GV, Strel'chyonok OA: **Evidence for the involvement of the transcortin carbohydrate moiety in the glycoprotein interaction with the plasma membrane of human placental syncytiotrophoblast**. *Biochim Biophys Acta* 1988, **938**(1):1-6.

2.	Perrin MH, Vale WW: **Corticotropin releasing factor receptors and their ligand family**. *Ann N Y Acad Sci* 1999, **885**:312-328.

3.	Wolffe AP, Bersimbaev RI, Tata JR: **Inhibition by estradiol of binding and mitogenic effect of epidermal growth factor in primary cultures of Xenopus hepatocytes**. *Mol Cell Endocrinol* 1985, **40**(2-3):167-173.

4.	Takahashi T, Chiba S, Hirano N, Yazaki Y, Hirai H: **Characterization of three erythropoietin (Epo)-binding proteins in various human Epo-responsive cell lines and in cells transfected with human Epo-receptor cDNA**. *Blood* 1995, **85**(1):106-114.

5.	Yassin RR: **Signaling pathways mediating gastrin's growth-promoting effects**. *Peptides* 1999, **20**(7):885-898.

6.	Kashiwagi A, Harano Y, Kosugi K, Nakano T, Hidaka H, Shigeta Y: **Reciprocal changes of insulin and glucagon receptors in primary cultured hepatocytes**. *J Biochem (Tokyo)* 1985, **97**(2):679-684.

7.	Mason JC, Tager HS: **Identification of distinct receptor complexes that account for high-and low-affinity glucagon binding to hepatic plasma membranes**. *Proc Natl Acad Sci U S A* 1985, **82**(20):6835-6839.

8.	Moens K, Flamez D, Van Schravendijk C, Ling Z, Pipeleers D, Schuit F: **Dual glucagon recognition by pancreatic beta-cells via glucagon and glucagon-like peptide 1 receptors**. *Diabetes* 1998, **47**(1):66-72.

9.	McIntosh CH, Bakich V, Kwok YN, Wong J, Brown JC: **The effects of substance P, histamine and histamine antagonists on somatostatin and gastrin release from the isolated perfused rat stomach**. *Regul Pept* 1987, **19**(3-4):253-263.

10.	Alexandrides TK, Smith RJ: **A novel fetal insulin-like growth factor (IGF) I receptor. Mechanism for increased IGF I- and insulin-stimulated tyrosine kinase activity in fetal muscle**. *J Biol Chem* 1989, **264**(22):12922-12930.

11.	Garofalo RS, Barenton B: **Functional and immunological distinction between insulin-like growth factor I receptor subtypes in KB cells**. *J Biol Chem* 1992, **267**(16):11470-11475.

12.	Hammond JM, Jarett L, Mariz IK, Daughaday WH: **Heterogeneity of insulin receptors on fat cell membranes**. *Biochem Biophys Res Commun* 1972, **49**(4):1122-1128.

13.	Ozaki K: **Insulin receptor-related receptor in rat islets of Langerhans**. *Eur J Endocrinol* 1998, **139**(2):244-247.

14.	McMahan CJ, Slack JL, Mosley B, Cosman D, Lupton SD, Brunton LL, Grubin CE, Wignall JM, Jenkins NA, Brannan CI *et al*: **A novel IL-1 receptor, cloned from B cells by mammalian expression, is expressed in many cell types**. *Embo J* 1991, **10**(10):2821-2832.

15.	Allouche M, Sahraoui Y, Augery-Bourget Y, Perrakis M, Jasmin C, Georgoulias V: **Interleukin 2 receptors**. *Leuk Res* 1990, **14**(8):699-703.

16.	Leeb-Lundberg LM, Marceau F, Muller-Esterl W, Pettibone DJ, Zuraw BL: **International union of pharmacology. XLV. Classification of the kinin receptor family: from molecular mechanisms to pathophysiological consequences**. *Pharmacol Rev* 2005, **57**(1):27-77.

17.	Maneva AI, Sirakov LM, Manev VV: **Lactoferrin binding to neutrophilic polymorphonuclear leucocytes**. *Int J Biochem* 1983, **15**(7):981-984.

18.	Misra UK, Gonzalez-Gronow M, Gawdi G, Pizzo SV: **Up-regulation of the alpha2-macroglobulin signaling receptor on rheumatoid synovial fibroblasts**. *J Biol Chem* 1997, **272**(1):497-502.

19.	Staniszewska I, Sariyer IK, Lecht S, Brown MC, Walsh EM, Tuszynski GP, Safak M, Lazarovici P, Marcinkiewicz C: **Integrin {alpha}9{beta}1 is a receptor for nerve growth factor and other neurotrophins**. *J Cell Sci* 2008.

20.	Nalivaiko E, Michaud JC, Soubrie P, Le Fur G: **Electrophysiological evidence for putative subtypes of neurotensin receptors in guinea-pig mesencephalic dopaminergic neurons**. *Neuroscience* 1998, **86**(3):799-811.

21.	Lai JP, Ho WZ, Kilpatrick LE, Wang X, Tuluc F, Korchak HM, Douglas SD: **Full-length and truncated neurokinin-1 receptor expression and function during monocyte/macrophage differentiation**. *Proc Natl Acad Sci U S A* 2006, **103**(20):7771-7776.

22.	Wise A, Foord SM, Fraser NJ, Barnes AA, Elshourbagy N, Eilert M, Ignar DM, Murdock PR, Steplewski K, Green A *et al*: **Molecular identification of high and low affinity receptors for nicotinic acid**. *J Biol Chem* 2003, **278**(11):9869-9874.

23.	Hryb DJ, Khan MS, Romas NA, Rosner W: **Solubilization and partial characterization of the sex hormone-binding globulin receptor from human prostate**. *Journal of Biological Chemistry* 1989, **264**(10):5378-5383.

24.	Moller LN, Stidsen CE, Hartmann B, Holst JJ: **Somatostatin receptors**. *Biochim Biophys Acta* 2003, **1616**(1):1-84.

25.	Robberecht P, Gourlet P: **[Recombinant receptors for testing agonists and antagonists of VIP and PACAP receptors]**. *Journal de Pharmacie de Belgique* 1996, **51**(3):165-169.

26.	Hennemann G, Docter R, Friesema EC, de Jong M, Krenning EP, Visser TJ: **Plasma membrane transport of thyroid hormones and its role in thyroid hormone metabolism and bioavailability**. *Endocr Rev* 2001, **22**(4):451-476.

27.	Moutafchiev DA, Shisheva AC, Sirakov LM: **Binding of transferrin-iron to the plasma membrane of a lactating rabbit mammary gland cell**. *Int J Biochem* 1983, **15**(5):755-758.

28.	Zwaagstra JC, Kassam Z, O'Connor-Mccourt MD: **Down-regulation of transforming growth factor-beta receptors: cooperativity between the types I, II, and III receptors and modulation at the cell surface**. *Experimental Cell Research* 1999, **252**(2):352-362.

29.	Tartaglia LA, Goeddel DV: **Two TNF receptors**. *Immunol Today* 1992, **13**(5):151-153.

30.	Hehlgans T, Mannel DN: **The TNF-TNF receptor system**. *Biol Chem* 2002, **383**(10):1581-1585.

31.	Robinson CJ, Stringer SE: **The splice variants of vascular endothelial growth factor (VEGF) and their receptors**. *J Cell Sci* 2001, **114**(Pt 5):853-865.
