# Supplementary material for: A model to explain specific cellular communications and cellular harmony:- a hypothesis of coupled cells and interactive coupling molecules
Source: Theor Biol Med Model. 2014 Sep 14;11:40. doi: 10.1186/1742-4682-11-40 (PMC4237941; doi:10.1186/1742-4682-11-40)
Supplement: Additional file 1 — 1. “Terminology”. 2. “Change in Cellular Characteristics”. 3. Note 1 “The Roles of SCD versus ACD”. Note 2. “Categories of SCD and ACD”. Note 3 “Further Examples of these AsCD Categories”. Note 4 “Change of differentiation type.”. 4. “The Effects of Growth Factors and other Bioactive Molecules 5. Note 1, “Additional Information on the Definition of a Trefone”. Note 2 “Clarification on Ligand and Binding Protein Interaction”. Note 3 “Cell Couplets – AsCD and the a-Cell and i-Cell”. Note 4 “Drosophila – Potential Trefones and ACD”. 6. “Calculated Levels of Trefones and TCC, with Cellular Actions”. 7. “Receptors for the Six IGFBPs and Cellular Effects”. 8. Note 1 “Extracellular Binding Proteins/ Soluble Receptors”. Note 2 “Cell Receptors for Binding Proteins/Soluble Receptors”. 9. Note 1. “Further Evidence for a Receptor for TCC”. Note 2 “Problems with Cross-linking Experiments”. 10. “Evidence for Binary Receptors aside from the IGF System”. 11. “Evidence for Two Receptors for a Particular Trefone, outside the IGF System”. 12. Note 1 “Mechanisms of Nuclear Localization”. Note 2 “NL of IGFBPs”. Note 3 “NL of Potential Trefones other than the IGF System”. 13. “Evidence Supporting the Existence of a-Cells and i-Cells”. 14. “Candidates with mainly a-Cell-Type or i-Cell-Type Characteristics”. 15. “Interacting Cells and Trefones not associated with the IGF System”. 16. “Heterogeneity/Variability of Cells in Culture.” 17. “Cell lines are not Typical of Normal Cells”. 18. “Cell Receptors and Cell Markers”. 19. “Defined Couplet Cells for Insulin and Glucagon; Gastrin and Histamine”. 20. “Potential Couplet Trefones”. 21. “Further Examples of Potential Trefone and Cell Couplets”. 22. “Examples of Cellular Regulation by Complexes”. 23. “Proteolytic enzymes and their inhibitors”. 24. “Expanded Definition of ‘Trefone’ and Classes of Couplet Cell Interactions”. 25. Note 1 “Extended Trefone Couplets”. Note 2 “Singlet Cells”. 26. “Background of Cancer Research”. [file 1742-4682-11-40-S1.zip › 3484671921331230_add1/3484671921331230_add12.doc]

**Additional File** ***Twelve***

**(d) Evidence for Internalization, Intracellular Signal Transfer and Nuclear Location**

***(i) Ligand-stimulated Endocytosis of Receptors and Nuclear Localization***

***Note 1. Mechanisms of nuclear localization***

*The purpose of this Note is to clarify the possible mechanism by which substances are localized to the nucleus*,.

A cell-membrane receptor, especially with a bound Trefone, may be internalized by a classical clathrin- or/and caveolin-dependent mechanism. Many molecules of receptor may then be recycled to the plasma membrane via early and recycling endosomes and some ligand and receptor may be degraded via the late endosome and lysosome. Some receptor molecules, perhaps with bound Trefone, translocate to the perinuclear space, nuclear membrane or to the intranuclear space. Activated EGFR and other RTKs are internalized primarily by clathrin-mediated endocytosis following growth factor-induced receptor clustering in clathrin coated-pits. In addition, clathrin-independent endocytosis can play an important role under particular conditions, such as in cell stimulation with high ligand concentrations. Some specificity may occur because clathrin-mediated endocytosis, but not caveolae-dependent endocytosis or macropinocytosis, is essential for the nuclear translocation of endostatin following the internalization process.

One interpretation of these events is that the receptor is monitoring the external environment for signals and it may be recycled until it detects a specific Trefone/ligand carrying a message; the Trefone and/or receptor then transfers this message directly to DNA or to a nuclear transcription factor or other protein or to RNA. The majority of growth factors/Trefones are targeted to chromatin. The internalized message may thus be deciphered into a “whole-of-cell” action. It should be noted that there are also non-classical pathways of intracellular translocation, independent of endoplasmic reticulum, Golgi and endosomes.

In aortic endothelial cells, FGF-2 seems to be directly translocated to the nucleus and therein could play a role in replication and/or in transcription of DNA. Further, the signal may be transferred downstream to another signalling component and it is then this activated component (eg. activated Akt or ERK) which enters the nucleus. In this case, the induction of nuclear events occurs without the nuclear localization of Trefone or receptor *per se.* This however, then becomes a more generic signal and perhaps not specific enough for a message that may be a critical signal to proliferate (for example).

Specific nuclear localization of a Trefone or its message has been observed. Earlier reviews have suggested that polypeptide ligands or their receptors, or both, might translocate to the nucleus to participate directly in gene regulation and these observations are supported by a similar claim ten years ago:- “A compelling body of new data, however, clearly indicates that polypeptide ligands and/or their receptors are transported into the nucleus, where they function directly to regulate the expression of specific transcription factors and gene loci.” This publication then lists nine polypeptide ligands that translocate into and function within the nucleus, and eight transmembrane polypeptide receptors that have been reported within the nucleus. These references exclude IGFs and IGFBPs but there is ample evidence of nuclear localization of these Trefones. (See the main text for evidence for the former and **Note 2** below for evidence for the latter.)

The presence or absence of a nuclear localization of an ICM may depend on the growth phase. For FGFR1, its nuclear localization within nontransformed human astrocytes is depleted in quiescent, confluent astrocytes, but it accumulated after the transition to a proliferating, subconfluent state, probably because of the cell cycle stage in that it is directly translocated to the nucleus in late G1.

**(d) Evidence for Internalization, Intracellular Signal Transfer and Nuclear Location.**

***(iv) Evidence for Nuclear Localization (NL) relating to the Trefones IGFBP-1… IGFBP-6***

**Note 2*. NL of IGFBPs***

*The purpose of this Note is to give evidence of NL and of functional roles for IGFBPs (-2, -3 and -5).*

IGFBP-2, -3 and -5 have been detected in or around the nucleus.

**IGFBP-2**

IGFBP-2 has been localized in the nucleus of cells of a lung adenocarcinoma line and found to co-locate and interact with an inhibitor of the cyclin-dependent kinase, p21(CIP1/WAF1), during growth inhibition of mouse lung epithelial cells. P21 has been shown to be a positive regulator in IGF-mediated signalling in the regulation of proliferation in a breast cancer cell line. IGFBP-2 is also localized on the nuclear surface of pancreatic cells.

**IGFBP-3**

IGFBP-3 has a domain with an amino-acid sequence corresponding to a putative nuclear localization signal that was predicted to direct the protein to the nucleus. This was found to be the case in a lung cancer cell line which exhibited staining for IGFBP-3 in the nucleus. Proliferating opossum kidney cells also internalize IGFBP-3 and transport it directly to the nucleus, in contrast to resting cells where IGFBP-3 is internalized and conveyed to endosomes. Intranuclear localization of IGFBP-3 also occurs in human keratinocytes during cell division and immunoreactive IGFBP-3 has been found in perinuclear/nuclear fractions in articular chondrocytes. Also, it has been demonstrated that nuclear translocation of endogenous IGFBP-3 by prostate cancer cells requires its secretion and re-uptake. Indicative of its intranuclear activity to modulate transcriptional activity, it has been shown that IGFBP-3 can bind with the nuclear retinoid receptor X receptor (RXR) and together they modulated the induction of apoptosis in prostate cancer cells . RXR regulates intracellular receptor signalling pathways by binding the nuclear receptor Nur77 and the complex moves, in an IGFBP-3-induced mitochondrial translocation, from the nucleus to mitochondria and then induces apoptosis via Bcl-2. IGFBP-3 has a putative nuclear export sequence (NES) which targets it to the endoplasmic reticulum and mitochondria from the nucleus to regulate apoptosis.

**IGFBP-5**

IGFBP-5 also has a nuclear localization signal. Nuclear uptake of IGFBP-5 (and IGFBP-3) but not IGFBP-1 and IGFBP-2 (which have no NLS) occurs by a common pathway in rapidly dividing breast cancer cells (T47D). The import of IGFBP-5 (and IGFBP-3) into the nucleus of Chinese hamster ovary cells is mediated by the nuclear transport protein, importin-β. IGFBP-5 has also been observed in the nuclear compartment of breast cancer cell lines MDA-MB-231 and Hs578T.

Intracellular IGFBP-5, internalized from secreted IGFBP-5, has also been localized in the nucleus of vascular smooth muscle cells, where it exhibits transcription-regulatory activity that is IGF independent.

Other potential transactivation activities of IGFBP-5 include that it interacts with the transcription factor FHL2A (four and a half LIM protein 2) and both are localized in the nucleus of osteoblasts where IGFBP-5 has a physical association with the histone-DNA complex in the nucleus.

**(d) Evidence for Internalization, Intracellular Signal Transfer and Nuclear Location.**

***(v) Evidence for Nuclear Localization (NL) relating to the other Potential Trefones and their Receptors***

**Note 3*. NL of Potential Trefones other than those of the IGF System***

*The purpose of this Note is to give evidence of NL outside of the IGF system and evidence of the functional roles for Trefone* versus *receptor.*

A notable potential Trefone is the growth hormone binding protein which has been detected in the nucleus of several types of cells. Other binding proteins (potentially Trefones) that have a nuclear presence include sex-hormone binding globulin and the closely-related androgen binding globulin (ABG). Once internalized, ABP was found to be confined to the endocytic and nuclear compartments. The nuclear labeling was high in primary spermatocytes and round spermatids but was absent in elongated spermatids with condensed chromatin, in which transcriptional activity had almost stopped

Reviews of the nuclear localizations of ICMs and potential Trefones list amphiregulin (Schwannoma-derived growth factor), angiotensin II, brain-derived growth factor, chorionic gonadotropin, EGF, FGF-1 and -2, gonadotropin, gonadotropin releasing hormone, growth hormone (GH), interferon-beta and -gamma, interleukin-1(alpha and beta) and -5 (IL-1, IL-5), luteinizing hormone releasing hormone, nerve growth factor (NGF), Notch, PDGF and prolactin (PL). Many of these have chromatin as their target.

Additional ICMs that have been located in the nucleus include angiogenin, atrial natriuretic peptide, colony-stimulating factor-1, heregulin, neurotensin, parathyroid stimulating hormone related-protein (PTHrP) and thyrotrophin-releasing hormone. PTHrP may affect RNA rather than DNA; it localizes to the nucleolus where it has the ability to bind to RNA by a motif in its NLS, possibly then regulating ribonucleoprotein complex formation.

Several receptors are also listed in reviews of nuclear localization – EGFR, ErbB-3, ErbB-4, FGFR, GHR, IL-5R, NGFR, PLR. Additional receptors which have been located in the nucleus include receptors for angiotensin type 1, ErbB-3, IL-1, ErbB-2, hepatocyte growth factor (c-Met), TGF-beta (Type I) and VEGF.

A receptor may be internalized after interaction with its ligand and the whole or part of the receptor may translocate to the nucleus. In the latter case, the intracellular domain (ICD) may be released from the transmembrane domain of the receptor proteolytically and the ICD translocates to the nucleus. This occurs for Notch and the EGF receptor ErbB-4 (when it binds the EGF ligand neuregulin), to release then the ICD which binds transcription factors and affects nuclear transcription. In contrast, when EGF itself binds to the EGF receptor ErbB-1, the receptor plus ligand are internalized and the whole receptor molecule translocates to the nucleus with the help of the nuclear transporter, importin-beta.

The following adds to the above reviews of transport of ICMs and receptors into the nucleus. It also focuses on their potential function to regulate the expression of specific transcription factors and gene loci to induce cell division or other life/death decisions. In addition, it focuses on whether the potential Trefone or the receptor is the carrier of the externally-derived signal to the chromatin and how this occurs.

The nuclear Trefone signal may be transferred directly or indirectly. A number of reports have indicated the presence of EGF and/or its receptor in the nucleus of target cells. Extracellular EGF is transported to the nucleus and it bound to chromatin (as is NGF and PDGF). EGFR translocated to the nucleus may regulate gene activation directly by binding to chromatin or indirectly by tyrosine phosphorylation of other chromatin-associated proteins. Both insulin and its receptor have been identified within nuclei and insulin signals both to activate serine kinases within the nucleus directly and thus to regulate a transcription factor and to stimulate the translocation of some active kinases to the nucleus. Of interest is that insulin's transit through the nuclear pore may be independent of its receptor but mediated by cytoplasmic proteins.

While receptors are present in the nucleus, it should be noted that this translocation from the cell membrane usually occurs only after stimulation by the ICM/Trefone (eg. EGF) and not usually in resting cells. Nuclear localization signals (NLS) for both ICMs and receptors need to be considered. The complex is internalized to the cell but it is not clear whether the receptor dissociates from the ICM/Trefone and is translocated into the nucleus by itself or whether it is carried by a NLS-carrying ICM/Trefone. Equally, a NLS-carrying receptor could carry a Trefone into the nucleus to produce a metabolic or cell division event. Once released into the cytosol, the proteins can access the nucleus by importins as described for IGFBP-3 and -5 and FGF receptor.

The classical understanding of the cellular effect of a steroid (eg. an estrogen) is that it readily passes through the cell membrane to form an intracellular complex with its nuclear steroid receptor (ERα or ERβ) which then binds the estrogen response elements in DNA, recruits transcriptional comodulators and thus regulates the expression of target genes. This understanding is modified by the now-known presence of cell-membrane bound steroid receptors. For a progesterone effect, the nuclear progesterone receptor with progesterone bound, is activated (phosphorylated) by active ERK which has been phosphorylated by cell-membrane bound progesterone receptor. The active nuclear complex then activates the cyclin-dependent kinase 2 and possibly causes the displacement of histone H1, a repressor of transcription, thereby inducing target gene expression.

In addition, both IL-1 plus receptor and IL-5 plus receptor are translocated to the nucleus as complexes; IL-5 effects nuclear transport of its α- and β-receptor subunits through a 'piggy back' mechanism. An FGF receptor (FGFR1) translocates to the nucleus in parallel with FGF-2, although syndecan co-locates with FGF-2, while the FGFR1 may be more perinuclear than intranuclear.

Neurotensin (NT) and NT receptor-3 complex are internalized and the mechanism of transport of this ICM/Trefone and receptor complex from the membrane to nucleus may involve retrograde transport via the pericentriolar recycling endosome/trans-Golgi network. This then is evidence for the unexpected translocation of a nonendogenous, ecto protein from endosomes back to the TGN, although this also occurs for various growth factors and cytokines and foreign toxins. Indeed, both the internalized NT ligand and a lower molecular-weight form of the surface receptor were translocated to the TGN.

PTHrP (PTH-related protein), once in the endoplasmic reticulum, may also escape normal proteolysis by translocation back to the cytoplasm by specific transport proteins and thus have access to the nucleus. The receptor for PTHrP is also located in the nucleus and it is possible that the function of a ligand may be just to allow access of the receptor into a particular intracellular pathway. It is also possible that there are two effects; a direct nuclear effect by PTHrP and a cytoplasmic effect by the receptor.

**REFERENCES:-**

1.	von Zastrow M, Sorkin A: **Signaling on the endocytic pathway**. *Curr Opin Cell Biol* 2007, **19**(4):436-445.

2.	Zwang Y, Yarden Y: **Systems biology of growth factor-induced receptor endocytosis**. *Traffic* 2009, **10**(4):349-363.

3.	Sorkin A, Goh LK: **Endocytosis and intracellular trafficking of ErbBs**. *Exp Cell Res* 2009, **315**(4):683-696.

4.	Bareford LM, Swaan PW: **Endocytic mechanisms for targeted drug delivery**. *Adv Drug Deliv Rev* 2007, **59**(8):748-758.

5.	Platta HW, Stenmark H: **Endocytosis and signaling**. *Curr Opin Cell Biol* 2011, **23**(4):393-403.

6.	Sadowski L, Pilecka I, Miaczynska M: **Signaling from endosomes: location makes a difference**. *Exp Cell Res* 2009, **315**(9):1601-1609.

7.	Song N, Ding Y, Zhuo W, He T, Fu Z, Chen Y, Song X, Fu Y, Luo Y: **The nuclear translocation of endostatin is mediated by its receptor nucleolin in endothelial cells**. *Angiogenesis* 2012, **15**(4):697-711.

8.	Prochiantz A, Theodore L: **Nuclear/growth factors**. *Bioessays* 1995, **17**(1):39-44.

9.	Nickel W: **The mystery of nonclassical protein secretion. A current view on cargo proteins and potential export routes**. *Eur J Biochem* 2003, **270**(10):2109-2119.

10.	Prochiantz A: **Protein and peptide transduction, twenty years later a happy birthday**. *Adv Drug Deliv Rev* 2008, **60**(4-5):448-451.

11.	Baldin V, Roman AM, Bosc-Bierne I, Amalric F, Bouche G: **Translocation of bFGF to the nucleus is G1 phase cell cycle specific in bovine aortic endothelial cells**. *Embo J* 1990, **9**(5):1511-1517.

12.	Siddle K: **Molecular basis of signaling specificity of insulin and IGF receptors: neglected corners and recent advances**. *Front Endocrinol (Lausanne)* 2012, **3**:34.

13.	Jans DA, Hassan G: **Nuclear targeting by growth factors, cytokines, and their receptors: a role in signaling?** *Bioessays* 1998, **20**(5):400-411.

14.	Clevenger CV: **Nuclear localization and function of polypeptide ligands and their receptors: a new paradigm for hormone specificity within the mammary gland?** *Breast Cancer Res* 2003, **5**(4):181-187.

15.	Stachowiak EK, Maher PA, Tucholski J, Mordechai E, Joy A, Moffett J, Coons S, Stachowiak MK: **Nuclear accumulation of fibroblast growth factor receptors in human glial cells--association with cell proliferation**. *Oncogene* 1997, **14**(18):2201-2211.

16.	Besnard V, Corroyer S, Trugnan G, Chadelat K, Nabeyrat E, Cazals V, Clement A: **Distinct patterns of insulin-like growth factor binding protein (IGFBP)-2 and IGFBP-3 expression in oxidant exposed lung epithelial cells**. *Biochim Biophys Acta* 2001, **1538**(1):47-58.

17.	Terrien X, Bonvin E, Corroyer S, Tabary O, Clement A, Henrion Caude A: **Intracellular colocalization and interaction of IGF-binding protein-2 with the cyclin-dependent kinase inhibitor p21CIP1/WAF1 during growth inhibition**. *Biochem J* 2005, **392**(Pt 3):457-465.

18.	Dupont J, Karas M, LeRoith D: **The cyclin-dependent kinase inhibitor p21CIP/WAF is a positive regulator of insulin-like growth factor I-induced cell proliferation in MCF-7 human breast cancer cells**. *J Biol Chem* 2003, **278**(39):37256-37264.

19.	Hoeflich A, Reisinger R, Schuett BS, Elmlinger MW, Russo VC, Vargas GA, Jehle PM, Lahm H, Renner-Muller I, Wolf E: **Peri/nuclear localization of intact insulin-like growth factor binding protein-2 and a distinct carboxyl-terminal IGFBP-2 fragment in vivo**. *Biochem Biophys Res Commun* 2004, **324**(2):705-710.

20.	Radulescu RT: **Nuclear localization signal in insulin-like growth factor-binding protein type 3**. *Trends in Biochemical Sciences* 1994, **19**(7):278.

21.	Jaques G, Noll K, Wegmann B, Witten S, Kogan E, Radulescu RT, Havemann K: **Nuclear localization of insulin-like growth factor binding protein 3 in a lung cancer cell line**. *Endocrinology* 1997, **138**(4):1767-1770.

22.	Li W, Fawcett J, Widmer HR, Fielder PJ, Rabkin R, Keller GA: **Nuclear transport of insulin-like growth factor-I and insulin-like growth factor binding protein-3 in opossum kidney cells**. *Endocrinology* 1997, **138**(4):1763-1766.

23.	Wraight CJ, Liepe IJ, White PJ, Hibbs AR, Werther GA: **Intranuclear localization of insulin-like growth factor binding protein-3 (IGFBP-3) during cell division in human keratinocytes**. *Journal of Investigative Dermatology* 1998, **111**(2):239-242.

24.	Sun T, Hunziker EB, Morales TI: **Subcellular distribution of the insulin-like growth factor (IGF) binding proteins (IGFBPs) 2 and 3 in articular chondrocytes**. *J Orthop Res* 2008, **26**(11):1421-1427.

25.	Lee KW, Liu B, Ma L, Li H, Bang P, Koeffler HP, Cohen P: **Cellular internalization of insulin-like growth factor binding protein-3: distinct endocytic pathways facilitate re-uptake and nuclear localization**. *J Biol Chem* 2004, **279**(1):469-476.

26.	Liu B, Lee HY, Weinzimer SA, Powell DR, Clifford JL, Kurie JM, Cohen P: **Direct functional interactions between insulin-like growth factor-binding protein-3 and retinoid X receptor-alpha regulate transcriptional signaling and apoptosis**. *J Biol Chem* 2000, **275**(43):33607-33613.

27.	Lee KW, Cobb LJ, Paharkova-Vatchkova V, Liu B, Milbrandt J, Cohen P: **Contribution of the orphan nuclear receptor Nur77 to the apoptotic action of IGFBP-3**. *Carcinogenesis* 2007, **28**(8):1653-1658.

28.	Paharkova-Vatchkova V, Lee KW: **Nuclear export and mitochondrial and endoplasmic reticulum localization of IGF-binding protein 3 regulate its apoptotic properties**. *Endocr Relat Cancer* 2010, **17**(2):293-302.

29.	Schedlich LJ, Young TF, Firth SM, Baxter RC: **Insulin-like growth factor-binding protein (IGFBP)-3 and IGFBP-5 share a common nuclear transport pathway in T47D human breast carcinoma cells**. *Journal of Biological Chemistry* 1998, **273**(29):18347-18352.

30.	Schedlich LJ, Le Page SL, Firth SM, Briggs LJ, Jans DA, Baxter RC: **Nuclear import of insulin-like growth factor-binding protein-3 and -5 is mediated by the importin beta subunit**. *Journal of Biological Chemistry* 2000, **275**(31):23462-23470.

31.	Butt AJ, Dickson KA, McDougall F, Baxter RC: **Insulin-like growth factor-binding protein-5 inhibits the growth of human breast cancer cells in vitro and in vivo**. *J Biol Chem* 2003, **278**(32):29676-29685.

32.	Xu Q, Li S, Zhao Y, Maures TJ, Yin P, Duan C: **Evidence that IGF binding protein-5 functions as a ligand-independent transcriptional regulator in vascular smooth muscle cells**. *Circ Res* 2004, **94**(5):E46-54.

33.	Amaar YG, Thompson GR, Linkhart TA, Chen ST, Baylink DJ, Mohan S: **Insulin-like growth factor-binding protein 5 (IGFBP-5) interacts with a four and a half LIM protein 2 (FHL2)**. *Journal of Biological Chemistry* 2002, **277**(14):12053-12060.

34.	Zhao Y, Yin P, Bach LA, Duan C: **Several acidic amino acids in the N-domain of insulin-like growth factor-binding protein-5 are important for its transactivation activity**. *J Biol Chem* 2006, **281**(20):14184-14191.

35.	Lobie PE, Garcia-Aragon J, Wang BS, Baumbach WR, Waters MJ: **Cellular localization of the growth hormone binding protein in the rat**. *Endocrinology* 1992, **130**(5):3057-3065.

36.	Gerard A, Egloff M, Gerard H, el Harate A, Domingo M, Gueant JL, Dang CD, Degrelle H: **Internalization of human sex steroid-binding protein in the monkey epididymis**. *J Mol Endocrinol* 1990, **5**(3):239-251.

37.	Gerard H, Gerard A, En Nya A, Felden F, Gueant JL: **Spermatogenic cells do internalize Sertoli androgen-binding protein: a transmission electron microscopy autoradiographic study in the rat**. *Endocrinology* 1994, **134**(3):1515-1527.

38.	Moroianu J, Riordan JF: **Nuclear translocation of angiogenin in proliferating endothelial cells is essential to its angiogenic activity**. *Proc Natl Acad Sci U S A* 1994, **91**(5):1677-1681.

39.	Chabot JG, Morel G, Belles-Isles M, Jeandel L, Heisler S: **ANF and exocrine pancreas: ultrastructural autoradiographic localization in acinar cells**. *Am J Physiol* 1988, **254**(3 Pt 1):E301-309.

40.	Scholl SM, Pallud C, Beuvon F, Hacene K, Stanley ER, Rohrschneider L, Tang R, Pouillart P, Lidereau R: **Anti-colony-stimulating factor-1 antibody staining in primary breast adenocarcinomas correlates with marked inflammatory cell infiltrates and prognosis**. *Journal of the National Cancer Institute* 1994, **86**(2):120-126.

41.	Li W, Park JW, Nuijens A, Sliwkowski MX, Keller GA: **Heregulin is rapidly translocated to the nucleus and its transport is correlated with c-myc induction in breast cancer cells**. *Oncogene* 1996, **12**(11):2473-2477.

42.	Morinville A, Martin S, Lavallee M, Vincent JP, Beaudet A, Mazella J: **Internalization and trafficking of neurotensin via NTS3 receptors in HT29 cells**. *Int J Biochem Cell Biol* 2004, **36**(11):2153-2168.

43.	Massfelder T, Dann P, Wu TL, Vasavada R, Helwig JJ, Stewart AF: **Opposing mitogenic and anti-mitogenic actions of parathyroid hormone-related protein in vascular smooth muscle cells: a critical role for nuclear targeting**. *Proc Natl Acad Sci U S A* 1997, **94**(25):13630-13635.

44.	Clemens TL, Cormier S, Eichinger A, Endlich K, Fiaschi-Taesch N, Fischer E, Friedman PA, Karaplis AC, Massfelder T, Rossert J *et al*: **Parathyroid hormone-related protein and its receptors: nuclear functions and roles in the renal and cardiovascular systems, the placental trophoblasts and the pancreatic islets**. *Br J Pharmacol* 2001, **134**(6):1113-1136.

45.	Morel G, Gourdji D, Grouselle D, Brunet N, Tixier-Vidal A, Dubois PM: **Immunocytochemical evidence for in vivo internalization of thyroliberin into rat pituitary target cells**. *Neuroendocrinology* 1985, **41**(4):312-320.

46.	Aarts MM, Levy D, He B, Stregger S, Chen T, Richard S, Henderson JE: **Parathyroid hormone-related protein interacts with RNA**. *J Biol Chem* 1999, **274**(8):4832-4838.

47.	Lu D, Yang H, Shaw G, Raizada MK: **Angiotensin II-induced nuclear targeting of the angiotensin type 1 (AT1) receptor in brain neurons**. *Endocrinology* 1998, **139**(1):365-375.

48.	Offterdinger M, Schofer C, Weipoltshammer K, Grunt TW: **c-erbB-3: a nuclear protein in mammary epithelial cells**. *J Cell Biol* 2002, **157**(6):929-939.

49.	Curtis BM, Widmer MB, deRoos P, Qwarnstrom EE: **IL-1 and its receptor are translocated to the nucleus**. *Journal of Immunology* 1990, **144**(4):1295-1303.

50.	Wang SC, Lien HC, Xia W, Chen IF, Lo HW, Wang Z, Ali-Seyed M, Lee DF, Bartholomeusz G, Ou-Yang F *et al*: **Binding at and transactivation of the COX-2 promoter by nuclear tyrosine kinase receptor ErbB-2**. *Cancer Cell* 2004, **6**(3):251-261.

51.	Gomes DA, Rodrigues MA, Leite MF, Gomez MV, Varnai P, Balla T, Bennett AM, Nathanson MH: **c-Met must translocate to the nucleus to initiate calcium signals**. *J Biol Chem* 2008, **283**(7):4344-4351.

52.	Zwaagstra JC, Guimond A, O'Connor-McCourt MD: **Predominant intracellular localization of the type I transforming growth factor-beta receptor and increased nuclear accumulation after growth arrest**. *Experimental Cell Research* 2000, **258**(1):121-134.

53.	Feng Y, Venema VJ, Venema RC, Tsai N, Caldwell RB: **VEGF induces nuclear translocation of Flk-1/KDR, endothelial nitric oxide synthase, and caveolin-1 in vascular endothelial cells**. *Biochem Biophys Res Commun* 1999, **256**(1):192-197.

54.	Linggi B, Carpenter G: **ErbB receptors: new insights on mechanisms and biology**. *Trends Cell Biol* 2006, **16**(12):649-656.

55.	Savion N, Vlodavsky I, Gospodarowicz D: **Nuclear accumulation of epidermal growth factor in cultured bovine corneal endothelial and granulosa cells**. *J Biol Chem* 1981, **256**(3):1149-1154.

56.	Boonstra J, Rijken P, Humbel B, Cremers F, Verkleij A, van Bergen en Henegouwen P: **The epidermal growth factor**. *Cell Biol Int* 1995, **19**(5):413-430.

57.	Rakowicz-Szulczynska EM, Rodeck U, Herlyn M, Koprowski H: **Chromatin binding of epidermal growth factor, nerve growth factor, and platelet-derived growth factor in cells bearing the appropriate surface receptors**. *Proc Natl Acad Sci U S A* 1986, **83**(11):3728-3732.

58.	Holt SJ, Alexander P, Inman CB, Davies DE: **Epidermal growth factor induced tyrosine phosphorylation of nuclear proteins associated with translocation of epidermal growth factor receptor into the nucleus**. *Biochemical Pharmacology* 1994, **47**(1):117-126.

59.	Kim SJ, Kahn CR: **Insulin regulation of mitogen-activated protein kinase kinase (MEK), mitogen-activated protein kinase and casein kinase in the cell nucleus: a possible role in the regulation of gene expression**. *Biochem J* 1997, **323 ( Pt 3)**:621-627.

60.	Harada S, Smith RM, Jarett L: **Mechanisms of nuclear translocation of insulin**. *Cell Biochem Biophys* 1999, **31**(3):307-319.

61.	Holt SJ, Alexander P, Inman CB, Davies DE: **Ligand-induced translocation of epidermal growth factor receptor to the nucleus of NR6/HER fibroblasts is serum dependent**. *Exp Cell Res* 1995, **217**(2):554-558.

62.	Reilly JF, Maher PA: **Importin beta-mediated nuclear import of fibroblast growth factor receptor: role in cell proliferation**. *J Cell Biol* 2001, **152**(6):1307-1312.

63.	Brinkmann AO: **Steroid hormone receptors: activators of gene transcription**. *J Pediatr Endocrinol* 1994, **7**(4):275-282.

64.	Roepke TA, Ronnekleiv OK, Kelly MJ: **Physiological consequences of membrane-initiated estrogen signaling in the brain**. *Front Biosci* 2011, **16**:1560-1573.

65.	Vicent GP, Nacht AS, Zaurin R, Ballare C, Clausell J, Beato M: **Minireview: role of kinases and chromatin remodeling in progesterone signaling to chromatin**. *Mol Endocrinol* 2010, **24**(11):2088-2098.

66.	Jans DA, Briggs LJ, Gustin SE, Jans P, Ford S, Young IG: **The cytokine interleukin-5 (IL-5) effects cotransport of its receptor subunits to the nucleus in vitro**. *FEBS Lett* 1997, **410**(2-3):368-372.

67.	Stachowiak MK, Maher PA, Joy A, Mordechai E, Stachowiak EK: **Nuclear accumulation of fibroblast growth factor receptors is regulated by multiple signals in adrenal medullary cells**. *Molecular Biology of the Cell* 1996, **7**(8):1299-1317.

68.	Zong F, Fthenou E, Wolmer N, Hollosi P, Kovalszky I, Szilak L, Mogler C, Nilsonne G, Tzanakakis G, Dobra K: **Syndecan-1 and FGF-2, but not FGF receptor-1, share a common transport route and co-localize with heparanase in the nuclei of mesenchymal tumor cells**. *PLoS One* 2009, **4**(10):e7346.

69.	Vandenbulcke F, Nouel D, Vincent JP, Mazella J, Beaudet A: **Ligand-induced internalization of neurotensin in transfected COS-7 cells: differential intracellular trafficking of ligand and receptor**. *J Cell Sci* 2000, **113 ( Pt 17)**:2963-2975.

70.	Olsnes S, Klingenberg O, Wiedlocha A: **Transport of exogenous growth factors and cytokines to the cytosol and to the nucleus**. *Physiol Rev* 2003, **83**(1):163-182.

71.	Saenz JB, Doggett TA, Haslam DB: **Identification and characterization of small molecules that inhibit intracellular toxin transport**. *Infect Immun* 2007, **75**(9):4552-4561.

72.	Watson PH, Fraher LJ, Hendy GN, Chung UI, Kisiel M, Natale BV, Hodsman AB: **Nuclear localization of the type 1 PTH/PTHrP receptor in rat tissues**. *J Bone Miner Res* 2000, **15**(6):1033-1044.

73.	Maioli E, Fortino V: **The complexity of parathyroid hormone-related protein signalling**. *Cell Mol Life Sci* 2004, **61**(3):257-262.
