# Supplementary material for: A model to explain specific cellular communications and cellular harmony:- a hypothesis of coupled cells and interactive coupling molecules
Source: Theor Biol Med Model. 2014 Sep 14;11:40. doi: 10.1186/1742-4682-11-40 (PMC4237941; doi:10.1186/1742-4682-11-40)
Supplement: Additional file 1 — 1. “Terminology”. 2. “Change in Cellular Characteristics”. 3. Note 1 “The Roles of SCD versus ACD”. Note 2. “Categories of SCD and ACD”. Note 3 “Further Examples of these AsCD Categories”. Note 4 “Change of differentiation type.”. 4. “The Effects of Growth Factors and other Bioactive Molecules 5. Note 1, “Additional Information on the Definition of a Trefone”. Note 2 “Clarification on Ligand and Binding Protein Interaction”. Note 3 “Cell Couplets – AsCD and the a-Cell and i-Cell”. Note 4 “Drosophila – Potential Trefones and ACD”. 6. “Calculated Levels of Trefones and TCC, with Cellular Actions”. 7. “Receptors for the Six IGFBPs and Cellular Effects”. 8. Note 1 “Extracellular Binding Proteins/ Soluble Receptors”. Note 2 “Cell Receptors for Binding Proteins/Soluble Receptors”. 9. Note 1. “Further Evidence for a Receptor for TCC”. Note 2 “Problems with Cross-linking Experiments”. 10. “Evidence for Binary Receptors aside from the IGF System”. 11. “Evidence for Two Receptors for a Particular Trefone, outside the IGF System”. 12. Note 1 “Mechanisms of Nuclear Localization”. Note 2 “NL of IGFBPs”. Note 3 “NL of Potential Trefones other than the IGF System”. 13. “Evidence Supporting the Existence of a-Cells and i-Cells”. 14. “Candidates with mainly a-Cell-Type or i-Cell-Type Characteristics”. 15. “Interacting Cells and Trefones not associated with the IGF System”. 16. “Heterogeneity/Variability of Cells in Culture.” 17. “Cell lines are not Typical of Normal Cells”. 18. “Cell Receptors and Cell Markers”. 19. “Defined Couplet Cells for Insulin and Glucagon; Gastrin and Histamine”. 20. “Potential Couplet Trefones”. 21. “Further Examples of Potential Trefone and Cell Couplets”. 22. “Examples of Cellular Regulation by Complexes”. 23. “Proteolytic enzymes and their inhibitors”. 24. “Expanded Definition of ‘Trefone’ and Classes of Couplet Cell Interactions”. 25. Note 1 “Extended Trefone Couplets”. Note 2 “Singlet Cells”. 26. “Background of Cancer Research”. [file 1742-4682-11-40-S1.zip › 3484671921331230_add1/3484671921331230_add13.doc]

**Additional File *Thirteen***

***(ii) Examples of Potential “a” Cells and “i”Cells as Evidence for this CTC Model***

**(1) Cells Controlled by the IGF System.**

***Detailed Description of Evidence Supporting the Existence of a-Cells and i-Cells***

*The purpose of this File is to provide examples of cell lines that exhibit some of the expected properties of a-Cells and i-Cells, which are listed in the main text, to support the CTC model.*

The list of cell properties below arise from the expectations of the couplet model expressed in **Figure 5** of the main text.

***FIGURE 5 (from main text).***

Couplet Cells withTrefones IGF-I and IGFBP-n and their Membrane Receptors. Both cells may have a membrane receptor for the IGF-I:IGFBPn complex (not shown).

Within the list below, in most quoted cases, a receptor for an IGF or an IGFBP has been positively identified. If an IGF or an IGFBP produces a cellular response, then a receptor for the Trefone is presumed, in some cases.

In the following list of cells and their properties, (+) means present, occurs or increases, while (-) means absent, does not occur or does not increase. Secretion (+) also means the substance is produced within the cell while secretion (-) also infers lack of production.

Abbreviations:- CL = Cell line; CCL = Cancer Cell Line.

**(1) The a-Cell Type (**IGF-insensitive)

(i) the cells will have receptors for the IGFBPn but will not produce the IGFBPn.

*Breast CCL (Hs578T)*.	- IGFBP-2 receptor	(+)

- IGFBP-2 secretion	(-)

(Exposure to IGFBP-2 had a pro-apoptotic, IGF-independent effect which directly

influenced the expression of 77 of 440 genes tested)

(ii)	the cells will produce and secrete IGF-I/II (^a^T)

(a) and have receptors for the specific IGFBP-n (^i^T)

*Intestinal smooth muscle cells*	**-** IGF-I secretion	(+)

- IGFBP-5 receptor	(+)

(ie. stimulates growth)

(b) but have no receptors for IGF-I/II

*Breast CCL (Hs578T)*	- IGF-I receptor	(-)

**-** IGF-I secretion	(+)

*Endometrial CCL (RL95-2)*	- IGF-I receptor	(-)

- IGF-I secretion	(+)

####	*Lymphoblast CL (RPMI 7666)*	- IGF-I receptor	(-)

####	- IGF-I secretion	(+)

(c) but will not produce nor secrete IGFBP-n

*Breast CCL (Hs578T)*	- IGFBP-2 secretion	(-)

- IGF-I secretion	(+)

*Lymphoblastoid cells (COR-L65 and COR-L26)*

- IGFBPs secretion	(-)

- IGF-I secretion	(+)

*Neuroblastoma CL (SK-N-MC)*

- IGFBPs secretion	(-)

- IGF-I secretion	(+)

(iii) IGFBP-n will stimulate IGF-I/II protein/mRNA production by a-Cells;

IGFBP-n receptor is presumed present,

*HUVEC (human umbilical vein endothelial cells)*

- IGFBP-3 stimulation of IGF-I secretion	(+)

*Intestinal smooth muscle cells*	- IGFBP-5 stimulation of IGF-I secretion	(+)

(iv) IGFBP-n will stimulate proliferation (of a-Cells); IGFBP-n receptor presumed present

*Intestinal smooth muscle cells;Kidney mesangial cells; Osteoclasts*;*Osteoblasts;* *Osteoblast clones* (IGF-I KO mice)	- IGFBP-5 stimulation of proliferation	(+)

*Airway smooth muscle cells*, *Colon cancer cells*

- IGFBP-3 stimulation of proliferation	(+)

*Adrenocortical tumour cells*	- IGFBP-2 (overexpressed) stimulation of

proliferation	(+)

(v) IGFBP-n will inhibit proliferation of a-Cells if in excess of a high level of IGF-I/II.

*Breast CCL ( Hs578T)*	- dose-dependent inhibition of cell proliferation

by IGFBP-3(60% inhibition at 20 nmol/L)

(vi) the cells will have receptors for the specific IGFBP-n (^i^T) but not for IGF-I/II

*Mouse embryo fibroblast cell line* - binding of IGFBP-1 onto cell surface	(+)

(but with inhibition).	- IGF-I receptor (-)

*Small cell lung cancer*	- binding of IGFBP-2 onto cell surface	(+)

- IGF-I response	(-)

*Breast CCL (Hs578T)*	- binding of IGFBP-3 onto cell surface	(+)

- IGF-I receptor	(-)

*Endometrial CCL (HHUA)*	- binding of IGFBP-3 onto cell surface	(+)

`	- IGF receptor	(-)

(vii) the cells will have no IGF-I receptor and not secrete IGFBPn.

*Human endometrial adenocarcinoma cell lines* RL952 and AN3CA

- IGF-I receptor	(-)

- IGFBP-1 secretion	(-)

**(2) The i-Cell Type**

(i)	the cells will have receptors for the IGF but will not produce the IGFI/II

####	*Glioblastoma (T98G) cells*	- IGF-I and IGF-II receptors	(+)

####  - IGF-I or -II secretion (-)

*Mink lung epithelial cells*	- IGF-I receptor	(+)

- IGFI/II secretion	(-)

*Osteosarcoma cell line (Saos-2)*	- IGF-I receptor	(+)

- IGF-I secretion	(-)

*Neuroblastoma cell line (CHP 234)*	- IGF-I receptor	(+)	- IGF-I secretion	(-)

(ii)	the cells will produce and secrete the specific IGFBP-n (^i^T)

(A specific IGFBP is assumed to be the specific Trefone for the cell in some cases)*

(a) and have receptors for IGF-I/II

*Endometrial adenocarcinoma cell lines (HEC 1B and KLE);* *Colon carcinoma cells (CaCo-2)*;	- IGFBP-1 secretion	(+)

- IGF-I receptor	(+)

*Neuroblastoma CL (CHP234)*	*-* IGFBP-2 secretion	(+)

- IGF-I receptor	(+)

*Colon carcinoma cells (CaCo-2)* - IGFBP-3 secretion	(+)

- IGF-I receptor	(+)

(b) but have no receptors for the specific IGFBP-n (^i^T)

*Breast CCL (MCF-7)*	- IGFBP-5 mRNA( with added IGF-1)	(+)

- LRP-1 receptor	(-)

(c) but will not produce and secrete IGF-I/II

*Mink lung epithelial cells*	- IGFBP-2 secretion	(+)

- IGFI/II secretion	(-)

(iii) IGF-I/II will stimulate both IGFBP-n *mRNA and protein* (mR&P) production (by i-Cells)

(with IGF-I/II receptor present)

*Kidney podocytes*	- IGF-II stimulation of IGFBP-2 mR&P	(+)	*Normal bovine mammary cell	-* IGF-I (and des-IGF) stimulation

of IGFBP-2 and -3 (+)

*Mammary epithelial cell line*	- IGF-I stimulation of IGFBP-3 mR&P	(+)

*Bone marrow stromal cells*	- IGF-I stimulation of IGFBP-3,-4,-5 protein (+)	- only IGFBP-3 mRNA (+)

*Adenocarcinoma-derived lung cell line A549 cells*

-.IGF-I stimulation of IGFBP-3	(+)

*Human fibroblasts*	- IGF-I stimulation of IGFBP-3	(+)

*Bovine fibroblasts*	- IGF-I stimulation of IGFBP-5 mR&P	(+)

*Intestinal smooth muscle*	- IGF-I stimulation of IGFBP-3, -4, and -5	(+)	*Diploid nontransformed line of rat thyroid follicular (FRTL-5) cells*

- IGF-I stimulation of IGFBP-5 mR&P	(+)

*Primary osteoblastic cells*	- IGF-I stimulation of IGFBP-5 mR&P	(+)

*Aortic smooth muscle cells*	- IGF-I stimulation of IGFBP-5 mR&P	(+)

*Intestinal smooth muscle cells-* IGF-I stimulation of IGFBP-5 mR&P	(+)

*Rat neuronal cell line*	- IGF-I stimulation - IGF-BP	(+)

*Baby hamster kidney cells*	- IGF-I stimulation - IGF-BP	(+)

(NB. Some of these protein findings may be due to a non-receptor mediated mechanism whereby IGFs may bind to and release cell-surface associated IGFBPs or prevent the degradation of IGFBPs by extracellular proteolytic enzymes.)

(iv) IGF-I/II will stimulate proliferation (of i-Cells); IGF-I receptor is present.

The literature is full of examples of this.

(v) IGF-I/II will inhibit proliferation (of i-Cells) when in excess of a high level of IGFBP-n

*Coronary smooth muscle cells* (growth in 10% fetal bovine serum – high IGFBP)

- added IGFs produce inhibition	(+)

(vi) the cells will have receptors for IGF-I/II but not for the specific IGFBP-n

*Breast CCL (MCF-7)*	- IGF-I receptor	(+)

- LRp-1 receptor	(-)	(LRp-1 receptor binds IGFBP-3 -4 and -5)

- α_5_β_1-_Integrin or α_v_β_3-_Integrin	(-)

(These bind IGFBP-1 and -2)

(vii) the cells will have no IGFBPn receptor and will not secrete IGF-I.

*Breast CCL (MCF-7)*	- IGFBP receptors [as above in(vi)]	(-)

- IGF-I secretion	(-)

* Note that it is not sufficient just to produce an IGFBP – indeed many cells secrete more than two IGFBPs in growth – the one special IGFBP stimulated by IGF-1/II is the one of interest for that cell. In the list where a specific IGFBP in named, it is the one which has specifically responded or is the only one present.

**Additional Evidence Consistent with the Model for the IGF System**

**.** A study of non-small-cell lung cancer (NSCLC) cells, showed that IGFBP-2 did not bind to these cells but both IGF-I and IGF-II bound predominantly to IGF-I receptors on these cells, whereas in small-cell lung cancer (SCLC) cells binding of IGFs was principally to surface-associated IGFBP-2. These latter SCLC cells failed to respond to stimulation by IGFs in a DNA-synthesis assay, indicating a lack of receptors for IGF but likely had receptors for IGFBP-2. NSCLC cells could therefore be derived from i-Cells and SCLC cells would then be from a-Cells.

**.** In relation to the distribution of couplet cells, cells of the human renal proximal tubule have a high level of IGF-I receptors and synthesize only a little IGF-I; these are possibly i-Cells. In contrast the cells of the distal tubule and collecting ducts lack IGF-I receptors but synthesize substantial IGF-I; these are possibly a-Cells.

**.** In studies of migrating mesangial cells, it was noted that IGF-I and IGFBP-5, in the absence of IGF-I, stimulated migration but strikingly different cellular phenotypes were induced. This suggests that each may stimulate separate pathways of cytoskeletal reorganization by interaction with their individual receptors. Two types of cells could be present, one type activated by IGF-I and one by the IGFBP.

**.** Preincubation of fibroblasts with IGFBP-3, before the addition of IGF-I, resulted in a potentiation of the subsequent IGF-I effect. This could be explained by the stimulation of cell division (SCD and AsCD) by the IGFBP within the preincubation period of (up to) 72 h with formation of more a-Cells so that subsequent addition of IGF now stimulates more i-Cell proliferation

**.** IGF-I mRNA and IGFBP-2 production show a striking temporal synchronisation in cerebellar and retinal development, but they are produced by different cells; IGF-I mRNA is produced by large projection neurons (cerebellar Purkinje and retinal ganglion cells), IGFBP-2 is produced by contiguous neuroglia (Bergmann glia in the cerebellum, Muller cells and astrocytes of the nerve fibre layer in the retina). This could indicate that, in development, these cells are formed as couplet cells from an AsCD.

**.** In the placenta of the monkey and human, the expressions of messenger RNAs for IGFs and IGFBPs show a spatial and temporal pattern such that IGF and IGFBP production rarely happen together, if at all. Again, this indicates potentially a- and i-Cell types.

**.** High density IGF-I binding sites, IGF-I receptors, in the olfactory bulb of the rat brain are located adjacent to putative cell-associated IGFBP-2, possibly indicating separated a-Cells and i-Cells.

# . Insulin-like growth factor (IGF)-binding protein-3 mutants that do not bind IGF-I or IGF-II, stimulate apoptosis in human prostate cancer cells (PC-3). In the CTC model, high levels of one Trefone in the presence of low levels of the complex (TCC), will produce some apoptosis.

# This information omits references to those cells that simply transport IGF through the cell to neighbours. For example, mouse preimplantation embryos exhibit receptor-mediated binding and **transcytosis** of maternal IGF-I.

**Qualification:-** The data used to support the model has been derived from possible a-Cells or i-Cells where IGFs and IGFBPs have had important activities as expected of Trefones. The task was to select those cellular properties compatible or congruent with a model of normal multicellular growth and its control.

**REFERENCES:-**

1.	Frommer KW, Reichenmiller K, Schutt BS, Hoeflich A, Ranke MB, Dodt G, Elmlinger MW: **IGF-independent effects of IGFBP-2 on the human breast cancer cell line Hs578T**. *J Mol Endocrinol* 2006, **37**(1):13-23.

2.	Kuemmerle JF, Zhou H: **Insulin-like growth factor-binding protein-5 (IGFBP-5) stimulates growth and IGF-I secretion in human intestinal smooth muscle by Ras-dependent activation of p38 MAP kinase and Erk1/2 pathways**. *J Biol Chem* 2002, **277**(23):20563-20571.

3.	De Leon DD, Bakker B, Wilson DM, Hintz RL, Rosenfeld RG: **Demonstration of insulin-like growth factor (IGF-I and -II) receptors and binding protein in human breast cancer cell lines**. *Biochem Biophys Res Commun* 1988, **152**(1):398-405.

4.	Osborne CK, Coronado EB, Kitten LJ, Arteaga CI, Fuqua SA, Ramasharma K, Marshall M, Li CH: **Insulin-like growth factor-II (IGF-II): a potential autocrine/paracrine growth factor for human breast cancer acting via the IGF-I receptor**. *Mol Endocrinol* 1989, **3**(11):1701-1709.

5.	Huff KK, Kaufman D, Gabbay KH, Spencer EM, Lippman ME, Dickson RB: **Secretion of an insulin-like growth factor-I-related protein by human breast cancer cells**. *Cancer Res* 1986, **46**(9):4613-4619.

6.	Reynolds RK, Hu C, Baker VV: **Transforming growth factor-alpha and insulin-like growth factor-I, but not epidermal growth factor, elicit autocrine stimulation of mitogenesis in endometrial cancer cell lines**. *Gynecol Oncol* 1998, **70**(2):202-209.

7.	Sekyi-Otu A, Bell RS, Ohashi C, Pollak M, Andrulis IL: **Insulin-like growth factor 1 (IGF-1) receptors, IGF-1, and IGF-2 are expressed in primary human sarcomas**. *Cancer Res* 1995, **55**(1):129-134.

8.	Massague J, Czech MP: **The subunit structures of two distinct receptors for insulin-like growth factors I and II and their relationship to the insulin receptor**. *J Biol Chem* 1982, **257**(9):5038-5045.

9.	Reeve JG, Payne JA, Bleehen NM: **Production of immunoreactive insulin-like growth factor-I (IGF-I) and IGF-I binding proteins by human lung tumours**. *Br J Cancer* 1990, **61**(5):727-731.

10.	Kiess W, Koepf G, Christiansen H, Blum WF: **Human neuroblastoma cells use either insulin-like growth factor-I or insulin-like growth factor-II in an autocrine pathway via the IGF-I receptor: variability of IGF, IGF binding protein (IGFBP) and IGF receptor gene expression and IGF and IGFBP secretion in human neuroblastoma cells in relation to cellular proliferation**. *Regul Pept* 1997, **72**(1):19-29.

11.	Granata R, Trovato L, Garbarino G, Taliano M, Ponti R, Sala G, Ghidoni R, Ghigo E: **Dual effects of IGFBP-3 on endothelial cell apoptosis and survival: involvement of the sphingolipid signaling pathways**. *Faseb J* 2004, **18**(12):1456-1458.

12.	Berfield AK, Andress DL, Abrass CK: **IGFBP-5(201-218) stimulates Cdc42GAP aggregation and filopodia formationin migrating mesangial cells**. *Kidney Int* 2000, **57**(5):1991-2003.

13.	Kanatani M, Sugimoto T, Nishiyama K, Chihara K: **Stimulatory effect of insulin-like growth factor binding protein-5 on mouse osteoclast formation and osteoclastic bone-resorbing activity**. *J Bone Miner Res* 2000, **15**(5):902-910.

14.	Andress DL, Birnbaum RS: **Human osteoblast-derived insulin-like growth factor (IGF) binding protein-5 stimulates osteoblast mitogenesis and potentiates IGF action**. *Journal of Biological Chemistry* 1992, **267**(31):22467-22472.

15.	Miyakoshi N, Richman C, Kasukawa Y, Linkhart TA, Baylink DJ, Mohan S: **Evidence that IGF-binding protein-5 functions as a growth factor**. *J Clin Invest* 2001, **107**(1):73-81.

16.	Cohen P, Rajah R, Rosenbloom J, Herrick DJ: **IGFBP-3 mediates TGF-beta1-induced cell growth in human airway smooth muscle cells**. *American Journal of Physiology - Lung Cellular & Molecular Physiology* 2000, **278**(3):L545-551.

17.	Kansra S, Ewton DZ, Wang J, Friedman E: **IGFBP-3 mediates TGF beta 1 proliferative response in colon cancer cells**. *Int J Cancer* 2000, **87**(3):373-378.

18.	Hoeflich A, Fettscher O, Lahm H, Blum WF, Kolb HJ, Engelhardt D, Wolf E, Weber MM: **Overexpression of insulin-like growth factor-binding protein-2 results in increased tumorigenic potential in Y-1 adrenocortical tumor cells**. *Cancer Res* 2000, **60**(4):834-838.

19.	Oh Y, Muller HL, Lamson G, Rosenfeld RG: **Insulin-like growth factor (IGF)-independent action of IGF-binding protein-3 in Hs578T human breast cancer cells. Cell surface binding and growth inhibition**. *Journal of Biological Chemistry* 1993, **268**(20):14964-14971.

20.	Cavaille F, Neau E, Vouters M, Bry-Gauillard H, Colombel A, Milliez J, Le Bouc Y: **IGFBP-1 inhibits EGF mitogenic activity in cultured endometrial stromal cells**. *Biochem Biophys Res Commun* 2006, **345**(2):754-760.

21.	Reeve JG, Morgan J, Schwander J, Bleehen NM: **Role for membrane and secreted insulin-like growth factor-binding protein-2 in the regulation of insulin-like growth factor action in lung tumors**. *Cancer Res* 1993, **53**(19):4680-4685.

22.	Oh Y, Muller HL, Pham H, Rosenfeld RG: **Demonstration of receptors for insulin-like growth factor binding protein-3 on Hs578T human breast cancer cells**. *J Biol Chem* 1993, **268**(35):26045-26048.

23.	Mochizuki T, Sakai K, Iwashita M: **Effects of insulin-like growth factor (IGF) binding protein-3 (IGFBP-3) on endometrial cancer (HHUA) cell apoptosis and EGF stimulated cell proliferation in vitro**. *Growth Horm IGF Res* 2006, **16**(3):202-210.

24.	Pekonen F, Nyman T, Rutanen EM: **Human endometrial adenocarcinoma cell lines HEC 1B and KLE secrete insulin-like growth factor binding protein-1 and contain IGF-I receptors**. *Molecular & Cellular Endocrinology* 1991, **75**(1):81-87.

25.	Schlenska-Lange A, Knupfer H, Lange TJ, Kiess W, Knupfer M: **Cell proliferation and migration in glioblastoma multiforme cell lines are influenced by insulin-like growth factor I in vitro**. *Anticancer Res* 2008, **28**(2A):1055-1060.

26.	Bastian SE, Walton PE, Ballard FJ, Belford DA: **Transport of IGF-I across epithelial cell monolayers**. *J Endocrinol* 1999, **162**(3):361-369.

27.	Wu HB, Kumar A, Tsai WC, Mascarenhas D, Healey J, Rechler MM: **Characterization of the inhibition of DNA synthesis in proliferating mink lung epithelial cells by insulin-like growth factor binding protein-3**. *Journal of Cellular Biochemistry* 2000, **77**(2):288-297.

28.	Schmid C, Ghirlanda-Keller C, Zapf J: **Effects of IGF-I and -II, IGF binding protein-3 (IGFBP-3), and transforming growth factor-beta (TGF-beta) on growth and apoptosis of human osteosarcoma Saos-2/B-10 cells: lack of IGF-independent IGFBP-3 effects**. *Eur J Endocrinol* 2001, **145**(2):213-221.

29.	Okazaki R, Conover CA, Harris SA, Spelsberg TC, Riggs BL: **Normal human osteoblast-like cells consistently express genes for insulin-like growth factors I and II but transformed human osteoblast cell lines do not**. *J Bone Miner Res* 1995, **10**(5):788-795.

30.	Kanzaki S, Baxter RC, Knutsen R, Baylink DJ, Mohan S: **Evidence that human bone cells in culture secrete insulin-like growth factor (IGF)-II and IGF binding protein-3 but not acid-labile subunit both under basal and regulated conditions**. *J Bone Miner Res* 1995, **10**(6):854-858.

31.	Hoeflich A, Yang Y, Kessler U, Heinz-Erian P, Kolb H, Kiess W: **Human colon carcinoma cells (CaCo-2) synthesize IGF-II and express IGF-I receptors and IGF-II/M6P receptors**. *Mol Cell Endocrinol* 1994, **101**(1-2):141-150.

32.	Oguchi S, Walker WA, Sanderson IR: **Profile of IGF-binding proteins secreted by intestinal epithelial cells changes with differentiation**. *Am J Physiol* 1994, **267**(5 Pt 1):G843-850.

33.	Sheikh MS, Shao ZM, Clemmons DR, LeRoith D, Roberts CT, Jr., Fontana JA: **Identification of the insulin-like growth factor binding proteins 5 and 6 (IGFBP-5 and 6) in human breast cancer cells**. *Biochem Biophys Res Commun* 1992, **183**(3):1003-1010.

34.	Li Y, Wood N, Parsons PG, Yellowlees D, Donnelly PK: **Expression of alpha2-macroglobulin receptor/low density lipoprotein receptor-related protein on surfaces of tumour cells: a study using flow cytometry**. *Cancer Lett* 1997, **111**(1-2):199-205.

35.	Dong F, Wu HB, Hong J, Rechler MM: **Insulin-like growth factor binding protein-2 mediates the inhibition of DNA synthesis by transforming growth factor-beta in mink lung epithelial cells**. *J Cell Physiol* 2002, **190**(1):63-73.

36.	Bridgewater DJ, Matsell DG: **Insulin-like growth factor binding protein-2 modulates podocyte mitogenesis**. *Pediatr Nephrol* 2003, **18**(11):1109-1115.

37.	McGrath MF, Collier RJ, Clemmons DR, Busby WH, Sweeny CA, Krivi GG: **The direct in vitro effect of insulin-like growth factors (IGFs) on normal bovine mammary cell proliferation and production of IGF binding proteins**. *Endocrinology* 1991, **129**(2):671-678.

38.	Cohick WS, Turner JD: **Regulation of IGF binding protein synthesis by a bovine mammary epithelial cell line**. *J Endocrinol* 1998, **157**(2):327-336.

39.	Grellier P, Feliers D, Yee D, Woodruff K, Abboud SL: **Interaction between insulin-like growth factor-I and insulin-like growth factor-binding proteins in TC-1 stromal cells**. *J Endocrinol* 1996, **149**(3):519-529.

40.	Price WA, Moats-Staats BM, Stiles AD: **Insulin-like growth factor-I (IGF-I) regulates IGFBP-3 and IGFBP-4 by multiple mechanisms in A549 human adenocarcinoma cells**. *Am J Respir Cell Mol Biol* 1995, **13**(4):466-476.

41.	Conover CA, Clarkson JT, Bale LK: **Effect of glucocorticoid on insulin-like growth factor (IGF) regulation of IGF-binding protein expression in fibroblasts**. *Endocrinology* 1995, **136**(4):1403-1410.

42.	Bale LK, Conover CA: **Regulation of insulin-like growth factor binding protein-3 messenger ribonucleic acid expression by insulin-like growth factor I**. *Endocrinology* 1992, **131**(2):608-614.

43.	Kuemmerle JF: **Endogenous IGF-I regulates IGF binding protein production in human intestinal smooth muscle cells**. *Am J Physiol Gastrointest Liver Physiol* 2000, **278**(5):G710-717.

44.	Backeljauw PF, Dai Z, Clemmons DR, D'Ercole AJ: **Synthesis and regulation of insulin-like growth factor binding protein-5 in FRTL-5 cells**. *Endocrinology* 1993, **132**(4):1677-1681.

45.	Dong Y, Canalis E: **Insulin-like growth factor (IGF) I and retinoic acid induce the synthesis of IGF-binding protein 5 in rat osteoblastic cells**. *Endocrinology* 1995, **136**(5):2000-2006.

46.	Duan C, Hawes SB, Prevette T, Clemmons DR: **Insulin-like growth factor-I (IGF-I) regulates IGF-binding protein-5 synthesis through transcriptional activation of the gene in aortic smooth muscle cells**. *J Biol Chem* 1996, **271**(8):4280-4288.

47.	Zimmermann EM, Li L, Hou YT, Cannon M, Christman GM, Bitar KN: **IGF-I induces collagen and IGFBP-5 mRNA in rat intestinal smooth muscle**. *Am J Physiol* 1997, **273**(4 Pt 1):G875-882.

48.	Xin X, Hou YT, Li L, Schmiedlin-Ren P, Christman GM, Cheng HL, Bitar KN, Zimmermann EM: **IGF-I increases IGFBP-5 and collagen alpha1(I) mRNAs by the MAPK pathway in rat intestinal smooth muscle cells**. *Am J Physiol Gastrointest Liver Physiol* 2004, **286**(5):G777-783.

49.	Orlowski CC, Chernausek SD, Akeson R: **Actions of insulin-like growth factor-I on the B104 neuronal cell line: effects on cell replication, receptor characteristics, and influence of secreted binding protein on ligand binding**. *J Cell Physiol* 1989, **139**(3):469-476.

50.	Hsu D, Olefsky JM: **Characterization of insulin-like growth factor (IGF) binding proteins and their role in modulating IGF-I action in BHK cells**. *J Biol Chem* 1992, **267**(35):25576-25582.

51.	Daughaday WH: **Growth hormone axis overview--somatomedin hypothesis**. *Pediatr Nephrol* 2000, **14**(7):537-540.

52.	Schriever C, Schmidt A, Breithardt G, Buddecke E: **Human recombinant insulin-like growth factor I and -II stimulate the expression of basic fibroblast growth factor but suppress the division of bovine coronary smooth muscle cells**. *Atherosclerosis* 1996, **122**(2):255-263.

53.	Lippman ME, Dickson RB, Kasid A, Gelmann E, Davidson N, McManaway M, Huff K, Bronzert D, Bates S, Swain S *et al*: **Autocrine and paracrine growth regulation of human breast cancer**. *J Steroid Biochem* 1986, **24**(1):147-154.

54.	O'Grady P, Huang SS, Huang JS: **Expression of a new type high molecular weight receptor (type V receptor) of transforming growth factor beta in normal and transformed cells**. *Biochem Biophys Res Commun* 1991, **179**(1):378-385.

55.	Masson O, Chavey C, Dray C, Meulle A, Daviaud D, Quilliot D, Muller C, Valet P, Liaudet-Coopman E: **LRP1 receptor controls adipogenesis and is up-regulated in human and mouse obese adipose tissue**. *PLoS One* 2009, **4**(10):e7422.

56.	Meyer T, Marshall JF, Hart IR: **Expression of alphav integrins and vitronectin receptor identity in breast cancer cells**. *Br J Cancer* 1998, **77**(4):530-536.

57.	van der Burg B, Isbrucker L, van Selm-Miltenburg AJ, de Laat SW, van Zoelen EJ: **Role of estrogen-induced insulin-like growth factors in the proliferation of human breast cancer cells**. *Cancer Res* 1990, **50**(24):7770-7774.

58.	Sandra A, Boes M, Dake BL, Stokes JB, Bar RS: **Infused IGF-I/IGFBP-3 complex causes glomerular localization of IGF-I in the rat kidney**. *Am J Physiol* 1998, **275**(1 Pt 1):E32-37.

59.	De Mellow JS, Baxter RC: **Growth hormone-dependent insulin-like growth factor (IGF) binding protein both inhibits and potentiates IGF-I-stimulated DNA synthesis in human skin fibroblasts**. *Biochemical & Biophysical Research Communications* 1988, **156**(1):199-204.

60.	Conover CA, Ronk M, Lombana F, Powell DR: **Structural and biological characterization of bovine insulin-like growth factor binding protein-3**. *Endocrinology* 1990, **127**(6):2795-2803.

61.	Lee WH, Javedan S, Bondy CA: **Coordinate expression of insulin-like growth factor system components by neurons and neuroglia during retinal and cerebellar development**. *Journal of Neuroscience* 1992, **12**(12):4737-4744.

62.	Han VK, Carter AM: **Spatial and temporal patterns of expression of messenger RNA for insulin-like growth factors and their binding proteins in the placenta of man and laboratory animals**. *Placenta* 2000, **21**(4):289-305.

63.	Russo VC, Bach LA, Werther GA: **Cell membrane association of insulin-like growth factor binding protein-2 (IGFBP-2) in the rat brain olfactory bulb**. *Prog Growth Factor Res* 1995, **6**(2-4):329-336.

64.	Hong J, Zhang G, Dong F, Rechler MM: **Insulin-like growth factor (IGF)-binding protein-3 mutants that do not bind IGF-I or IGF-II stimulate apoptosis in human prostate cancer cells**. *Journal of Biological Chemistry* 2002, **277**(12):10489-10497.

65.	Smith RM, Garside WT, Aghayan M, Shi CZ, Shah N, Jarett L, Heyner S: **Mouse preimplantation embryos exhibit receptor-mediated binding and transcytosis of maternal insulin-like growth factor I**. *Biol Reprod* 1993, **49**(1):1-12.
