# Supplementary material for: A model to explain specific cellular communications and cellular harmony:- a hypothesis of coupled cells and interactive coupling molecules
Source: Theor Biol Med Model. 2014 Sep 14;11:40. doi: 10.1186/1742-4682-11-40 (PMC4237941; doi:10.1186/1742-4682-11-40)
Supplement: Additional file 1 — 1. “Terminology”. 2. “Change in Cellular Characteristics”. 3. Note 1 “The Roles of SCD versus ACD”. Note 2. “Categories of SCD and ACD”. Note 3 “Further Examples of these AsCD Categories”. Note 4 “Change of differentiation type.”. 4. “The Effects of Growth Factors and other Bioactive Molecules 5. Note 1, “Additional Information on the Definition of a Trefone”. Note 2 “Clarification on Ligand and Binding Protein Interaction”. Note 3 “Cell Couplets – AsCD and the a-Cell and i-Cell”. Note 4 “Drosophila – Potential Trefones and ACD”. 6. “Calculated Levels of Trefones and TCC, with Cellular Actions”. 7. “Receptors for the Six IGFBPs and Cellular Effects”. 8. Note 1 “Extracellular Binding Proteins/ Soluble Receptors”. Note 2 “Cell Receptors for Binding Proteins/Soluble Receptors”. 9. Note 1. “Further Evidence for a Receptor for TCC”. Note 2 “Problems with Cross-linking Experiments”. 10. “Evidence for Binary Receptors aside from the IGF System”. 11. “Evidence for Two Receptors for a Particular Trefone, outside the IGF System”. 12. Note 1 “Mechanisms of Nuclear Localization”. Note 2 “NL of IGFBPs”. Note 3 “NL of Potential Trefones other than the IGF System”. 13. “Evidence Supporting the Existence of a-Cells and i-Cells”. 14. “Candidates with mainly a-Cell-Type or i-Cell-Type Characteristics”. 15. “Interacting Cells and Trefones not associated with the IGF System”. 16. “Heterogeneity/Variability of Cells in Culture.” 17. “Cell lines are not Typical of Normal Cells”. 18. “Cell Receptors and Cell Markers”. 19. “Defined Couplet Cells for Insulin and Glucagon; Gastrin and Histamine”. 20. “Potential Couplet Trefones”. 21. “Further Examples of Potential Trefone and Cell Couplets”. 22. “Examples of Cellular Regulation by Complexes”. 23. “Proteolytic enzymes and their inhibitors”. 24. “Expanded Definition of ‘Trefone’ and Classes of Couplet Cell Interactions”. 25. Note 1 “Extended Trefone Couplets”. Note 2 “Singlet Cells”. 26. “Background of Cancer Research”. [file 1742-4682-11-40-S1.zip › 3484671921331230_add1/3484671921331230_add14.doc]

**Additional File *Fourteen***

***(ii) Examples of Potential “a” Cells and “i”Cells as Evidence for this CTC Model***

**(1) Cells Controlled by the IGF System.**

***Summary of Candidates with mainly a-Cell-Type or i-Cell-Type Characteristics***

*The purpose of this File is to justify the concept of couplet type a-Cells and i-Cells by allocating cell lines to either type, based on the majority of their properties.*

Based on the evidence provided on the existence of a-Cells and i-Cells (**Additional File** ***13***) and on additional evidence, the following **Table S1** categorizes various cell types into cells that have mostly the properties of either an a-Cell or an i-Cell.

For this table, it is assumed that the IGF system is the Trefone couplet system for these cells, with a focus on IGF-I as the ^a^T. The IGFBP referred to in the Table is not the only one produced by the cells mentioned but seems to be the most likely one to be the Trefone couplet for IGF-I. In the IGF system, it is taken that the receptor for IGFBP-1 is the α_5_β_1-_Integrin_,_ for IGFBP-2 either the α_5_β_1-_Integrin or α_v_β_3-_Integrin, and for IGFBP-4, -5 and -6, LRP-1. (See **Additional File** ***Seven*, Table S1*.***)

**TABLE S1. Examples of Cell Lines with Predominant Properties of Either the a-Cell or i-Cell Ty**pes, based on the IGF System.

| **Cell Lines with Some Properties of a-Cell Type** | **Properties and References** | **Cell Lines with Some Properties of i-Cell Type** | **Properties and**  **References** |
| --- | --- | --- | --- |
| Lymphoblastoid  COR-L65, COR -L26; Daudi line | Produce IGF-I but not any IGFBPs; IGF-I receptor (-) | Mammary epithelial  MAC-T | IGF-I receptors (+); has little IGF-I and secretes IGFBP-2 |
| Lymphoblast  RPMI-1766 | Produces IGF1; IGF-I receptor (-) | Schwannoma | IGF-I receptor (+); produces IGFBP-1 |
| Breast cancer  Hs578T | Produces IGF-I; IGF-I receptor (-); does not produce IGFBP-2 but responds to IGFBP-2; IGFBP-2 receptor α_5_β_1_-Integrin (+) ; has IGFBP-3 receptor. (But also IGF-II receptor (+); secretes IGFBP-3 ; IGF-I mRNA (-) ) | Breast cancer  MCF-7 | IGF-I receptor (+); Does not produce IGF-I; produces IGFBP-2 ; IGFBP-2 receptors α_5_β_1-_Integrin(-), α_v_β_3_Integrin (-), LRP-1 (- ) |
| Breast cancer  MDA-MB-231 | Produces IGF-I; IGFBP receptor (RLP-1) (+) α_5_β_1_ and α_v_β_3_ (+) (But IGF-1 receptor (+) ; produces IGFBP-1 and 3 mRNA, and IGFBP-6) | Breast cancer  MDA-MB-468 | IGF-1 increases cell numbers; IGF-1 receptor (+); produces IGFBP-5; receptors (-) (LRP-1 , α_v_β_3-_Integrin) |
| Endometrial cancer  HHUA | IGF-I receptor (-), IGFBP-3 binds to cell surface | Breast cancer  T-47D | Produces minimal IGF-I; IGF-1 receptor (+); produces IGFBP-5 ; receptors (-) (α_5_β_1-_Integrin, α_v_β_3-_Integrin |
| Endometrial cancer cell line (RL95-2)  (RL952 and AN3CA) | Produce IGF-I, IGF-I receptor (-) ; IGFBP-1, secretion (-), IGF-I receptor (-) | Breast cancer  ZR-75-1 | Produces minimal IGF-I ; IGF-I mRNA (-); IGF-1 receptor (+); produces IGFBP-2 ; Receptors (-) ( α_5_β_1-_Integrin, α_v_β_3-_Integrin |
|  |  | Endometrial cancer cell line (HEC 1B and KLE) | Produce IGFBP-1, IGF-I receptors (+): (HEC 1B had the highest IGFBP-1 and highest IGF-1 receptors) |

*The Table provides examples of cell lines whose properties allow them to be allocated to either an a-Cell type or an i-Cell type. The properties include the production of Trefones (IGFI/II or an IGFBP) and presence of receptors for them.*

**Note 1**. ***Detection Limits***. The list in **Table S1** generally indicates the presence or absence of various members of the IGF system. However there is a decision limit to define presence or absence given that all cells may produce a small constitutive amount of the components of the system. Also, there is a question of quantity when a component is “present”. For example, while both breast cancer cell lines MCF-7 and T47-D have IGF-I receptors, T47-D requires 20 times the amount of IGF for half-maximum DNA synthesis. This could reflect differences in receptor number per cell, receptor transduction efficiency and/or the time for the cell cycle (doubling time).

**Note 2.** ***False Positives and Negatives.*** Some of the evidence that these interpretations are based on, may be spurious. For example, positive reports of IGF-I expression would be false if there is nonspecific detection of mRNAs by the IGF-IcDNA due to cross-reaction. False positive reports of secreted IGF-I in immunoassays could be due to lack of antibody specificity (perhaps detecting IGF-II) while false negative reports could be due to interference by IGF-binding proteins. Reports of an IGF-I receptor based on just cell-surface binding may be false because of IGF binding to IGFBPs on the cell surface.

Immunohistochemical intracellular detection may be misleading in that a Trefone from one type of cell may be internalised into another cell with a receptor and its presence inside the second cell may lead to an interpretation that the second cell can synthesise it. The presence of mRNA for the Trefone would support a deduction of biosynthesis by a particular cell.

**Note 3.** ***Specificity for an IGFBP.*** It is likely that only one IGFBP (of the possible six) is a Trefone for a particular cell. Many cells secrete more than one IGFBP but the Trefone will be the one with a significant increase upon IGF stimulation. Also, if an i-Cell produces a large amount of IGFBPs, then the cells will not respond to more IGF-I unless it also is high enough to exceed the inhibitory IGFBP.

**Note 4.** ***Non-specific Receptors***. Evidence that a non-specific receptor (eg. LRP-1) is present does not prove that it is used as a receptor for the particular Trefone; it may be primarily used for internalization of another ligand which is in higher concentration.

**Note 5.** ***Variation in Stock Culture***. Some of the variations in results from different laboratories could be due to a change in the “clones” that were the original stock culture in the laboratories. IGFBP levels differ between two different pools of human primary diploid fibroblasts (HDFs) available from ATCC (American Type Culture Collection) where each pool consists of HDFs isolated from several different newborn donors. But some of the differences could be due to contamination of cells, especially by a cervical cancer cell line (HeLa-cells, which has been a major consideration for over 50 years. The estimate of cell lines contaminated by HeLa and other cells has been put at between 15 and 29% by some - “For decades, biologists working with contaminated or misidentified cell lines have wasted time and money and produced spurious results”. Others have estimated that there was an 18% misidentification of cells in 1999 compared to a 16% in 1977, where “a substantial proportion of cell lines is mislabeled or replaced by cells derived from a different individual, tissue or species”. A list of cross-contaminated or misidentified cell lines has been published to enable a culture check and a recommendation made for short tandem repeat profiling to prevent cell misidentification and cross-contamination with unambiguous cell authentication.

Also, single clones may have the capacity to change types with increased number of passages, For example, T47D breast cancer cells transfected with IGFBP-3 were initially inhibited by IGFBP-3 bur after several passages, the transfected cells became resistant to these inhibitory effects of IGFBP-3. In addition, the seeding level may influence cell density and this, plus the time for which proliferation continues (in logarithmic growth versus quiescence), can influence production of the components of the IGF system^,^. Indeed, a change of character/phenotype can occur within a single growth phase (eg. comparing young and senescent fibroblasts, the levels of 26 extracellular proteins differed significantly in the conditioned media*.* In cultured fibroblasts, the mRNA of the kallistatin receptor which is also a transcription factor *(KLF4),* is found at high levels in growth-arrested cells and is nearly undetectable in cells that are in the exponential phase of proliferation.

Variation in culture media may also explain some differences, eg. the presence of newborn-calf serum will markedly decrease IGF-I mRNA while increasing cell number by 100%. Also, the amount of fetal/calf bovine serum used is variable from zero to 20% and its composition is not well defined with variable amounts of IGFs and IGFBPs. Serum at levels between 5 and10% can significantly attenuate IGF-II expression in IEC-6 cells. Another difference between results for organ cells derives from tissue integrity and animal age - adult rat hepatocytes have few IGF-I receptors, hepatocytes from regenerating liver have a sixfold increase and those from fetal rat liver have a 12-fold increase. These latter levels are comparable to those in the hepatoma cells (HEP-G2).

**Note 6.** ***Change in Cell Type during Growth.*** “It may be useful to think of a cell culture as being in equilibrium between multipotent stem cells and mature differentiated cells and to suppose that that the equilibrium may shift according to the environmental conditions.”

Some of the differences in results then could be due to a change in the cell types within the course of the experiment due to variation in cell division types. (See **Additional File** ***Three, Note 2***, **Figure 1** “A Diagram of Possible Types of Cell Division” for examples.) In the period of 72 hours, a culture of n Type C cells with 1xSCD (LCDf) followed by 2xSCD (SRE), could result in a final population of cells of 8n Type C/D cells. Or a culture of n Type C cells with 1xAsCD (Dual LCDf) followed by 2xSCD (SRE) could result in a final population of cells of 4n Type C/D_1_ cells and 4n Type C/D_2_ cells. Or there could be a mixture of types in between these and the normally expected 8n Type C cells derived from 4xSCD (SRE). The extent of change would depend on the content of the media and the experimental additives.

**Note 7.** ***Change in Cell Type Not Detected.*** It is well known that even microscopic observation of the cells may not detect change in cell type. Functional change can occur without morphological change. Mutants of PC12 pheochromocytoma cell lines closely resembled each other in most properties tested: cell size and shape; division rate; protein, phosphoprotein, and glycoprotein composition; cell surface morphology. Again, pancreatic carcinoma cells (AR4–2J) transfected with a growth hormone (FGF-2) strongly resemble mock cells in possessing identical morphology by light and electron microscopy analysis and in responding to glucocorticoid-induced differentiation.

Cell variants without morphologic change can be detected by the measurement of enzymes, cell-surface markers (eg. immunophenotyping) or other cell-specific molecules. As an example of functional change, the human colon adenocarcinoma cell line, Caco-2, having reached confluence, undergoes a spontaneous enterocytic differentiation between days 5 and 16 of culture, as indicated by expression of brush border enzymes (eg. sucrase-isomaltase mRNA increased 12-fold). During this change, IGFBP-3 and IGFBP-2 increased (8.5- and 5-fold), whereas IGFBP-4 and a 31kD species decreased (by 43% and 90%).

**Note 8.** ***Imbalance of Cells.*** The predominance of i-Cells in **Table S1** (above) may reflect the bias for i-Cells to be detected in the isolation of cells in culture because of the predisposition of culture media containing additives (eg. fetal calf serum) to stimulate growth of cells with an IGF-I receptor.

Information is also known about other cell lines, many of which seem to have more properties of i-Cells than a-Cells when based on the IGF system eg. human mammary epithelial cells (MCF-10A), rat mammary epithelial (RME) cells, Ewing sarcoma A673 cells, Mink lung epithelial cells (CCL64), prostate cell lines (PC-3, DU-145, LNCaP), neuroblastoma cells (SK-N-MC), cervical cancer cells (HT-3), osteosarcoma cells (SaOS-2), fibroblasts (3T3, WI-38), smooth muscle cells, a conditionally immortalized human marrow stromal cell line, hMS(3-4).

In cultured human dermal cells, keratinocytes possess IGF-I receptors and are responsive to IGF-I which is primarily derived from underlying dermal fibroblasts, and they produce predominantly IGFBP-2. These show again, i-Cell characteristics. Similarly, FRTL-5 cells (a nontransformed line of rat thyroid follicular cells) also have i-Cell characteristics. They possess a IGF-I receptor, produce increased amounts of only IGFBP-5 in response to IGF-1 and do not produce IGF-I . Chinese hamster ovary (CHO) cells would also be of the i-Cell type because they have IGF-I receptors with no secreted IGF-I nor IGF-ImRNA and they secrete IGFBP-4 .

**Note 9.** ***Genetic Shifts.*** In addition to the tabled examples, there are some examples of cells that have the mixed properties of a transvestype – internally one type but dressed as the other type! They may reflect a cancer scenario where the cell has genetically compensated to cope with abnormal receptors or other genetic expressions abnormal to that cell type.

**(i).** JEG-3 choriocarcinoma cells have been shown to express both IGF receptors, the type-1 IGF receptor and the IGF-II/M6PR, and express IGF-II mRNA but not IGF-I .

These cells, which possess type I IGF receptors, were unable to produce IGF-BPs. These would seem to be i-Cells that cannot produce IGFBPs.

**(ii).** Schwannoma cells strongly overexpress IGF-I receptor and IGF-I and -II (which are released from the cells). Also, IGFBP-1 is strongly expressed in schwannoma at the mRNA level, it is secreted and it stimulates proliferation. The IGFBP-1 receptor, α_5_β_1_ integrin, is also expressed on these cells_._ In total then, these Schwannoma cells have all components and characteristics of both a-Cells and i-Cells. In contrast, Schwann cells (as observed in nerve injury) are i-Cells expressing IGF-I receptor and IGFBP-5 and these cells do not secrete IGFBP-1 and have less β_1_integrin receptor.

**(iii).** T98G human glioblastoma cells have the properties of a-Cells (There is no response to IGF-I, there is no IGF-I receptor and they secrete large amount of IGF-I.) and of an i-Cells. (They respond to IGF-I, they have an IGF-I receptor, do not secrete IGF-I but secrete IGFBPs.)

# Note 10. *Bound or Secreted?* The CTC model has the cell with the receptor for a growth factor-like Trefone labelled as the i-Cell. The i-Cell then produces a receptor and perhaps a soluble receptor, acting as an extracellular binding protein, which is the other Trefone. This soluble receptor could be derived from alternative splicing. The initial response then to an a-Trefone will require a decision between the cells synthesizing more or less transmembrane receptor or less or more secreted soluble receptor. This is indicated by the effect of EGF on a splicing model where EGF produces a massive reprogramming of alternative splicing. Akt activation by EGF branches to SR (Ser/Arg) protein-specific kinases (SRPK) to induce their autophosphorylation which then switches the splicing kinases from a Hsp70- to a Hsp90-containing complex. SRPK nuclear translocation is enhanced by the Hsp90 association and SRPK regulates alternative splicing in the nucleus. Thus, the EGF would determine the relative amounts of receptor that is membrane-bound *versus* secreted soluble receptor and, in the CTC model, would stimulate increased production of the secreted form.

**Note 11.** ***Transfection.*** Transfection experiments will produce abnormal cells. Assuming that there is a finely balanced system in a specific cell, say an a-Cell, then the transfection of an IGF-I receptor into that cell will completely disrupt its normal system, in that it is programmed for the production of IGF-I, not its reception. It is already producing a receptor for the couplet IGFBP and now coping with the internalization of both IGF-I and an IGFBP. This would surely produce a grossly abnormal cell with abnormal responses, perhaps producing a programming incompatability or crash and consequent programmed death. In a transfection experiment with both a-Cells and i-Cells present, then the a-Cells would be affected as described and the i-Cells would be grossly overproducing a critical regulatory component with increased intracellular IGF-I due to extra IGF-I receptor. Overall, interpretations from such transfection experiments would need to be cautious.

**REFERENCES:-**

1.	Reeve JG, Payne JA, Bleehen NM: **Production of immunoreactive insulin-like growth factor-I (IGF-I) and IGF-I binding proteins by human lung tumours**. *Br J Cancer* 1990, **61**(5):727-731.

2.	Pfeffer LM, Donner DB, Tamm I: **Interferon-alpha down-regulates insulin receptors in lymphoblastoid (Daudi) cells. Relationship to inhibition of cell proliferation**. *J Biol Chem* 1987, **262**(8):3665-3670.

3.	Zhao X, McBride BW, Politis I, Huynh HT, Akers RM, Burton JH, Turner JD: **Receptor binding and growth-promoting activity of insulin-like growth factor-I in a bovine mammary epithelial cell line (MAC-T3)**. *J Endocrinol* 1992, **134**(2):307-312.

4.	Ramagnolo D, Akers RM, Byatt JC, Wong EA, Turner JD: **IGF-I-induced IGFBP-3 potentiates the mitogenic actions of IGF-I in mammary epithelial MD-IGF-I cells**. *Mol Cell Endocrinol* 1994, **102**(1-2):131-139.

5.	Massague J, Czech MP: **The subunit structures of two distinct receptors for insulin-like growth factors I and II and their relationship to the insulin receptor**. *J Biol Chem* 1982, **257**(9):5038-5045.

6.	Sekyi-Otu A, Bell RS, Ohashi C, Pollak M, Andrulis IL: **Insulin-like growth factor 1 (IGF-1) receptors, IGF-1, and IGF-2 are expressed in primary human sarcomas**. *Cancer Res* 1995, **55**(1):129-134.

7.	Cheng HL, Feldman EL: **Insulin-like growth factor-I (IGF-I) and IGF binding protein-5 in Schwann cell differentiation**. *J Cell Physiol* 1997, **171**(2):161-167.

8.	Ammoun S, Schmid MC, Zhou L, Ristic N, Ercolano E, Hilton DA, Perks CM, Hanemann CO: **Insulin-like growth factor-binding protein-1 (IGFBP-1) regulates human schwannoma proliferation, adhesion and survival**. *Oncogene* 2012, **31**(13):1710-1722.

9.	Huff KK, Kaufman D, Gabbay KH, Spencer EM, Lippman ME, Dickson RB: **Secretion of an insulin-like growth factor-I-related protein by human breast cancer cells**. *Cancer Res* 1986, **46**(9):4613-4619.

10.	De Leon DD, Bakker B, Wilson DM, Hintz RL, Rosenfeld RG: **Demonstration of insulin-like growth factor (IGF-I and -II) receptors and binding protein in human breast cancer cell lines**. *Biochem Biophys Res Commun* 1988, **152**(1):398-405.

11.	Osborne CK, Coronado EB, Kitten LJ, Arteaga CI, Fuqua SA, Ramasharma K, Marshall M, Li CH: **Insulin-like growth factor-II (IGF-II): a potential autocrine/paracrine growth factor for human breast cancer acting via the IGF-I receptor**. *Mol Endocrinol* 1989, **3**(11):1701-1709.

12.	Yee D, Lebovic GS, Marcus RR, Rosen N: **Identification of an alternate type I insulin-like growth factor receptor beta subunit mRNA transcript**. *J Biol Chem* 1989, **264**(36):21439-21441.

13.	Perks CM, McCaig C, Clarke JB, Clemmons DR, Holly JM: **A non-IGF binding mutant of IGFBP-3 modulates cell function in breast epithelial cells**. *Biochemical & Biophysical Research Communications* 2002, **294**(5):988-994.

14.	Frommer KW, Reichenmiller K, Schutt BS, Hoeflich A, Ranke MB, Dodt G, Elmlinger MW: **IGF-independent effects of IGFBP-2 on the human breast cancer cell line Hs578T**. *J Mol Endocrinol* 2006, **37**(1):13-23.

15.	Schutt BS, Langkamp M, Rauschnabel U, Ranke MB, Elmlinger MW: **Integrin-mediated action of insulin-like growth factor binding protein-2 in tumor cells**. *J Mol Endocrinol* 2004, **32**(3):859-868.

16.	Oh Y, Muller HL, Lamson G, Rosenfeld RG: **Insulin-like growth factor (IGF)-independent action of IGF-binding protein-3 in Hs578T human breast cancer cells. Cell surface binding and growth inhibition**. *Journal of Biological Chemistry* 1993, **268**(20):14964-14971.

17.	Oh Y, Muller HL, Pham H, Rosenfeld RG: **Demonstration of receptors for insulin-like growth factor binding protein-3 on Hs578T human breast cancer cells**. *J Biol Chem* 1993, **268**(35):26045-26048.

18.	De Leon DD, Bakker B, Wilson DM, Lamson G, Rosenfeld RG: **Insulin-like growth factor binding proteins in human breast cancer cells: relationship to hIGFBP-2 and hIGFBP-3**. *J Clin Endocrinol Metab* 1990, **71**(2):530-532.

19.	Furlanetto RW, DiCarlo JN: **Somatomedin-C receptors and growth effects in human breast cells maintained in long-term tissue culture**. *Cancer Res* 1984, **44**(5):2122-2128.

20.	Lippman ME, Dickson RB, Kasid A, Gelmann E, Davidson N, McManaway M, Huff K, Bronzert D, Bates S, Swain S *et al*: **Autocrine and paracrine growth regulation of human breast cancer**. *J Steroid Biochem* 1986, **24**(1):147-154.

21.	Deng H, Lin Y, Badin M, Vasilcanu D, Stromberg T, Jernberg-Wiklund H, Sehat B, Larsson O: **Over-accumulation of nuclear IGF-1 receptor in tumor cells requires elevated expression of the receptor and the SUMO-conjugating enzyme Ubc9**. *Biochem Biophys Res Commun* 2011, **404**(2):667-671.

22.	van der Burg B, Isbrucker L, van Selm-Miltenburg AJ, de Laat SW, van Zoelen EJ: **Role of estrogen-induced insulin-like growth factors in the proliferation of human breast cancer cells**. *Cancer Res* 1990, **50**(24):7770-7774.

23.	Adamo ML, Shao ZM, Lanau F, Chen JC, Clemmons DR, Roberts CT, Jr., LeRoith D, Fontana JA: **Insulin-like growth factor-I (IGF-I) and retinoic acid modulation of IGF-binding proteins (IGFBPs): IGFBP-2, -3, and -4 gene expression and protein secretion in a breast cancer cell line**. *Endocrinology* 1992, **131**(4):1858-1866.

24.	Pluijm vd, Vloedgraven H, Papapoulos S, Lowick C, Grzesik W, Kerr J, Robey PG: **Attachment characteristics and involvement of integrins in adhesion of breast cancer cell lines to extracellular bone matrix components**. *Lab Invest* 1997, **77**(6):665-675.

25.	Meyer T, Marshall JF, Hart IR: **Expression of alphav integrins and vitronectin receptor identity in breast cancer cells**. *Br J Cancer* 1998, **77**(4):530-536.

26.	O'Grady P, Huang SS, Huang JS: **Expression of a new type high molecular weight receptor (type V receptor) of transforming growth factor beta in normal and transformed cells**. *Biochem Biophys Res Commun* 1991, **179**(1):378-385.

27.	Li Y, Wood N, Parsons PG, Yellowlees D, Donnelly PK: **Expression of alpha2-macroglobulin receptor/low density lipoprotein receptor-related protein on surfaces of tumour cells: a study using flow cytometry**. *Cancer Lett* 1997, **111**(1-2):199-205.

28.	Masson O, Chavey C, Dray C, Meulle A, Daviaud D, Quilliot D, Muller C, Valet P, Liaudet-Coopman E: **LRP1 receptor controls adipogenesis and is up-regulated in human and mouse obese adipose tissue**. *PLoS One* 2009, **4**(10):e7422.

29.	Chazaud B, Bonavaud S, Plonquet A, Pouchelet M, Gherardi RK, Barlovatz-Meimon G: **Involvement of the [uPAR:uPA:PAI-1:LRP] complex in human myogenic cell motility**. *Exp Cell Res* 2000, **258**(2):237-244.

30.	Newton S, Reeves E, Gralnick H, Mohla S, Yamada K, Olden K, Akiyama S: **Inhibition of experimental metastasis of human breast-carcinoma cells in athymic nude-mice by anti-alpha(5)beta(1) fibronectin receptor integrin antibodies**. *Int J Oncol* 1995, **6**(5):1063-1070.

31.	Yee D, Morales FR, Hamilton TC, Von Hoff DD: **Expression of insulin-like growth factor I, its binding proteins, and its receptor in ovarian cancer**. *Cancer Res* 1991, **51**(19):5107-5112.

32.	Sheikh MS, Shao ZM, Hussain A, Clemmons DR, Chen JC, Roberts CT, Jr., LeRoith D, Fontana JA: **Regulation of insulin-like growth factor-binding-protein-1, 2, 3, 4, 5, and 6: synthesis, secretion, and gene expression in estrogen receptor-negative human breast carcinoma cells**. *J Cell Physiol* 1993, **155**(3):556-567.

33.	Mochizuki T, Sakai K, Iwashita M: **Effects of insulin-like growth factor (IGF) binding protein-3 (IGFBP-3) on endometrial cancer (HHUA) cell apoptosis and EGF stimulated cell proliferation in vitro**. *Growth Horm IGF Res* 2006, **16**(3):202-210.

34.	Yee D, Paik S, Lebovic GS, Marcus RR, Favoni RE, Cullen KJ, Lippman ME, Rosen N: **Analysis of insulin-like growth factor I gene expression in malignancy: evidence for a paracrine role in human breast cancer**. *Mol Endocrinol* 1989, **3**(3):509-517.

35.	Reynolds RK, Hu C, Baker VV: **Transforming growth factor-alpha and insulin-like growth factor-I, but not epidermal growth factor, elicit autocrine stimulation of mitogenesis in endometrial cancer cell lines**. *Gynecol Oncol* 1998, **70**(2):202-209.

36.	Pekonen F, Nyman T, Rutanen EM: **Human endometrial adenocarcinoma cell lines HEC 1B and KLE secrete insulin-like growth factor binding protein-1 and contain IGF-I receptors**. *Molecular & Cellular Endocrinology* 1991, **75**(1):81-87.

37.	Milazzo G, Giorgino F, Damante G, Sung C, Stampfer MR, Vigneri R, Goldfine ID, Belfiore A: **Insulin receptor expression and function in human breast cancer cell lines**. *Cancer Res* 1992, **52**(14):3924-3930.

38.	van den Berg HW, Claffie D, Boylan M, McKillen J, Lynch M, McKibben B: **Expression of receptors for epidermal growth factor and insulin-like growth factor I by ZR-75-1 human breast cancer cell variants is inversely related: the effect of steroid hormones on insulin-like growth factor I receptor expression**. *Br J Cancer* 1996, **73**(4):477-481.

39.	Maxwell P, van den Berg HW: **Changes in the secretion of insulin-like growth factor binding proteins -2 and -4 associated with the development of tamoxifen resistance and estrogen independence in human breast cancer cell lines**. *Cancer Lett* 1999, **139**(2):121-127.

40.	Yee D, Favoni RE, Lebovic GS, Lombana F, Powell DR, Reynolds CP, Rosen N: **Insulin-like growth factor I expression by tumors of neuroectodermal origin with the t(11;22) chromosomal translocation. A potential autocrine growth factor**. *J Clin Invest* 1990, **86**(6):1806-1814.

41.	Pyke C, Kristensen P, Ralfkiaer E, Grondahl-Hansen J, Eriksen J, Blasi F, Dano K: **Urokinase-type plasminogen activator is expressed in stromal cells and its receptor in cancer cells at invasive foci in human colon adenocarcinomas**. *Am J Pathol* 1991, **138**(5):1059-1067.

42.	Micutkova L, Diener T, Li C, Rogowska-Wrzesinska A, Mueck C, Huetter E, Weinberger B, Grubeck-Loebenstein B, Roepstorff P, Zeng R *et al*: **Insulin-like growth factor binding protein-6 delays replicative senescence of human fibroblasts**. *Mech Ageing Dev* 2011, **132**(10):468-479.

43.	Nelson-Rees WA, Flandermeyer RR, Hawthorne PK: **Banded marker chromosomes as indicators of intraspecies cellular contamination**. *Science* 1974, **184**(4141):1093-1096.

44.	Chatterjee R: **Cell biology. Cases of mistaken identity**. *Science* 2007, **315**(5814):928-931.

45.	MacLeod RA, Dirks WG, Matsuo Y, Kaufmann M, Milch H, Drexler HG: **Widespread intraspecies cross-contamination of human tumor cell lines arising at source**. *Int J Cancer* 1999, **83**(4):555-563.

46.	Drexler HG, Dirks WG, Matsuo Y, MacLeod RA: **False leukemia-lymphoma cell lines: an update on over 500 cell lines**. *Leukemia* 2003, **17**(2):416-426.

47.	Capes-Davis A, Theodosopoulos G, Atkin I, Drexler HG, Kohara A, MacLeod RA, Masters JR, Nakamura Y, Reid YA, Reddel RR *et al*: **Check your cultures! A list of cross-contaminated or misidentified cell lines**. *Int J Cancer* 2010, **127**(1):1-8.

48.	Barallon R, Bauer SR, Butler J, Capes-Davis A, Dirks WG, Elmore E, Furtado M, Kline MC, Kohara A, Los GV *et al*: **Recommendation of short tandem repeat profiling for authenticating human cell lines, stem cells, and tissues**. *In Vitro Cell Dev Biol Anim* 2010, **46**(9):727-732.

49.	Firth SM, Fanayan S, Benn D, Baxter RC: **Development of resistance to insulin-like growth factor binding protein-3 in transfected T47D breast cancer cells**. *Biochem Biophys Res Commun* 1998, **246**(2):325-329.

50.	Price WA, Moats-Staats BM, D'Ercole AJ, Stiles AD: **Insulin-like growth factor binding protein production and regulation in fetal rat lung cells**. *Am J Respir Cell Mol Biol* 1993, **8**(4):425-432.

51.	Kutoh E, Schwander J, Margot JB: **Cell-density-dependent modulation of the rat insulin-like-growth-factor-binding protein 2 and its gene**. *Eur J Biochem* 1995, **234**(2):557-562.

52.	Corkins MR, Park JH, Davis DV, Slentz DH, MacDonald RG: **Regulation of the insulin-like growth factor axis by increasing cell number in intestinal epithelial (IEC-6) cells**. *Growth Horm IGF Res* 1999, **9**(6):414-424.

53.	Shields JM, Christy RJ, Yang VW: **Identification and characterization of a gene encoding a gut-enriched Kruppel-like factor expressed during growth arrest**. *J Biol Chem* 1996, **271**(33):20009-20017.

54.	Bornfeldt KE, Arnqvist HJ, Norstedt G: **Regulation of insulin-like growth factor-I gene expression by growth factors in cultured vascular smooth muscle cells**. *J Endocrinol* 1990, **125**(3):381-386.

55.	Caro JF, Poulos J, Ittoop O, Pories WJ, Flickinger EG, Sinha MK: **Insulin-like growth factor I binding in hepatocytes from human liver, human hepatoma, and normal, regenerating, and fetal rat liver**. *J Clin Invest* 1988, **81**(4):976-981.

56.	Freshney RI: **Culture of Animal Cells: A Manual of Basic Technique and Specialized Applications**, Sixth Edition edn: John Wiley & Sons, Inc.; 2011.

57.	Green SH, Rydel RE, Connolly JL, Greene LA: **PC12 cell mutants that possess low- but not high-affinity nerve growth factor receptors neither respond to nor internalize nerve growth factor**. *J Cell Biol* 1986, **102**(3):830-843.

58.	Estival A, Louvel D, Couderc B, Prats H, Hollande E, Vaysse N, Clemente F: **Morphological and biological modifications induced in a rat pancreatic acinar cancer cell line (AR4-2J) by unscheduled expression of basic fibroblast growth factors**. *Cancer Res* 1993, **53**(5):1182-1187.

59.	Park JH, Corkins MR, Vanderhoof JA, Caruso NM, Hrbek MJ, Schaffer BS, Slentz DH, McCusker RH, MacDonald RG: **Expression of insulin-like growth factor-II and insulin-like growth factor binding proteins during Caco-2 cell proliferation and differentiation**. *J Cell Physiol* 1996, **166**(2):396-406.

60.	Tavakkol A, Elder JT, Griffiths CE, Cooper KD, Talwar H, Fisher GJ, Keane KM, Foltin SK, Voorhees JJ: **Expression of growth hormone receptor, insulin-like growth factor 1 (IGF-1) and IGF-1 receptor mRNA and proteins in human skin**. *J Invest Dermatol* 1992, **99**(3):343-349.

61.	Murashita MM, Russo VC, Edmondson SR, Wraight CJ, Werther GA: **Identification of insulin-like growth factor binding proteins from cultured human epidermal keratinocytes**. *J Cell Physiol* 1995, **163**(2):339-345.

62.	Tramontano D, Moses AC, Picone R, Ingbar SH: **Characterization and regulations of the receptor for insulin-like growth factor-I in the FRTL-5 rat thyroid follicular cell line**. *Endocrinology* 1987, **120**(2):785-790.

63.	Backeljauw PF, Dai Z, Clemmons DR, D'Ercole AJ: **Synthesis and regulation of insulin-like growth factor binding protein-5 in FRTL-5 cells**. *Endocrinology* 1993, **132**(4):1677-1681.

64.	Eggo MC, Bachrach LK, Burrow GN: **Role of non-TSH factors in thyroid cell growth**. *Acta Endocrinol Suppl (Copenh)* 1987, **281**:231-237.

65.	Maciel RM, Moses AC, Villone G, Tramontano D, Ingbar SH: **Demonstration of the production and physiological role of insulin-like growth factor II in rat thyroid follicular cells in culture**. *J Clin Invest* 1988, **82**(5):1546-1553.

66.	Jones JI, Gockerman A, Busby WH, Jr., Wright G, Clemmons DR: **Insulin-like growth factor binding protein 1 stimulates cell migration and binds to the alpha 5 beta 1 integrin by means of its Arg-Gly-Asp sequence**. *Proc Natl Acad Sci U S A* 1993, **90**(22):10553-10557.

67.	Fagin JA, Pixley S, Slanina S, Ong J, Melmed S: **Insulin-like growth factor I gene expression in GH3 rat pituitary cells: messenger ribonucleic acid content, immunocytochemistry, and secretion**. *Endocrinology* 1987, **120**(5):2037-2043.

68.	Ritvos O, Ranta T, Jalkanen J, Suikkari AM, Voutilainen R, Bohn H, Rutanen EM: **Insulin-like growth factor (IGF) binding protein from human decidua inhibits the binding and biological action of IGF-I in cultured choriocarcinoma cells**. *Endocrinology* 1988, **122**(5):2150-2157.

69.	Ammoun S, Schmid MC, Ristic N, Zhou L, Hilton D, Ercolano E, Carroll C, Hanemann CO: **The role of insulin-like growth factors signaling in merlin-deficient human schwannomas**. *Glia* 2012, **60**(11):1721-1733.

70.	Cheng HL, Sullivan KA, Feldman EL: **Immunohistochemical localization of insulin-like growth factor binding protein-5 in the developing rat nervous system**. *Brain Res Dev Brain Res* 1996, **92**(2):211-218.

71.	Utermark T, Kaempchen K, Hanemann CO: **Pathological adhesion of primary human schwannoma cells is dependent on altered expression of integrins**. *Brain Pathol* 2003, **13**(3):352-363.

72.	Ambrose D, Resnicoff M, Coppola D, Sell C, Miura M, Jameson S, Baserga R, Rubin R: **Growth regulation of human glioblastoma T98G cells by insulin-like growth factor-1 and its receptor**. *J Cell Physiol* 1994, **159**(1):92-100.

73.	Schlenska-Lange A, Knupfer H, Lange TJ, Kiess W, Knupfer M: **Cell proliferation and migration in glioblastoma multiforme cell lines are influenced by insulin-like growth factor I in vitro**. *Anticancer Res* 2008, **28**(2A):1055-1060.

74.	Zhou Z, Qiu J, Liu W, Zhou Y, Plocinik RM, Li H, Hu Q, Ghosh G, Adams JA, Rosenfeld MG *et al*: **The Akt-SRPK-SR axis constitutes a major pathway in transducing EGF signaling to regulate alternative splicing in the nucleus**. *Mol Cell* 2012, **47**(3):422-433.
