# Supplementary material for: A model to explain specific cellular communications and cellular harmony:- a hypothesis of coupled cells and interactive coupling molecules
Source: Theor Biol Med Model. 2014 Sep 14;11:40. doi: 10.1186/1742-4682-11-40 (PMC4237941; doi:10.1186/1742-4682-11-40)
Supplement: Additional file 1 — 1. “Terminology”. 2. “Change in Cellular Characteristics”. 3. Note 1 “The Roles of SCD versus ACD”. Note 2. “Categories of SCD and ACD”. Note 3 “Further Examples of these AsCD Categories”. Note 4 “Change of differentiation type.”. 4. “The Effects of Growth Factors and other Bioactive Molecules 5. Note 1, “Additional Information on the Definition of a Trefone”. Note 2 “Clarification on Ligand and Binding Protein Interaction”. Note 3 “Cell Couplets – AsCD and the a-Cell and i-Cell”. Note 4 “Drosophila – Potential Trefones and ACD”. 6. “Calculated Levels of Trefones and TCC, with Cellular Actions”. 7. “Receptors for the Six IGFBPs and Cellular Effects”. 8. Note 1 “Extracellular Binding Proteins/ Soluble Receptors”. Note 2 “Cell Receptors for Binding Proteins/Soluble Receptors”. 9. Note 1. “Further Evidence for a Receptor for TCC”. Note 2 “Problems with Cross-linking Experiments”. 10. “Evidence for Binary Receptors aside from the IGF System”. 11. “Evidence for Two Receptors for a Particular Trefone, outside the IGF System”. 12. Note 1 “Mechanisms of Nuclear Localization”. Note 2 “NL of IGFBPs”. Note 3 “NL of Potential Trefones other than the IGF System”. 13. “Evidence Supporting the Existence of a-Cells and i-Cells”. 14. “Candidates with mainly a-Cell-Type or i-Cell-Type Characteristics”. 15. “Interacting Cells and Trefones not associated with the IGF System”. 16. “Heterogeneity/Variability of Cells in Culture.” 17. “Cell lines are not Typical of Normal Cells”. 18. “Cell Receptors and Cell Markers”. 19. “Defined Couplet Cells for Insulin and Glucagon; Gastrin and Histamine”. 20. “Potential Couplet Trefones”. 21. “Further Examples of Potential Trefone and Cell Couplets”. 22. “Examples of Cellular Regulation by Complexes”. 23. “Proteolytic enzymes and their inhibitors”. 24. “Expanded Definition of ‘Trefone’ and Classes of Couplet Cell Interactions”. 25. Note 1 “Extended Trefone Couplets”. Note 2 “Singlet Cells”. 26. “Background of Cancer Research”. [file 1742-4682-11-40-S1.zip › 3484671921331230_add1/3484671921331230_add15.doc]

**Additional File** ***Fifteen***

***(ii) Examples of Potential “a-Cells” and “i-Cells” as Evidence for this CTC Model***

**(2) Cells Controlled by System of ICMs other than the IGF System.**

***Interacting Cells and Trefones not associated with the IGF System***

*The purpose of this File is to justify the concept of couplet-type a-Cells and i-Cells by examples outside the IGF system.*

The properties of the CTC Model require that

(1) For the **a-Cell Type:**

(i) the cells will have receptors for ^i^T but will not produce ^i^T

(ii) the cells will produce and secrete ^a^T

(a) and have receptors for ^i^T

(b) but have no receptors for ^a^T

(c) but will not produce nor secrete ^i^T

(iii) ^i^T will stimulate ^a^T protein/mRNA production by a-Cells;

^i^T receptor is presumed present

(iv) ^i^T will normally stimulate proliferation of a-Cells; ^i^T receptor is presumed present

(v) ^i^T will inhibit proliferation of a-Cells if in excess of a high level of ^a^T

(vi) the cells will have receptors for ^i^T but not for ^a^T;

^a^T will inhibit proliferation of a-Cells by binding ^i^T

(vii) the cells will have no ^a^T receptor and will not secrete ^i^T.

(2) For the **i-Cell Type;**

(i) the cells will have receptors for ^a^T but will not produce ^a^T

(ii) the cells will produce and secrete ^i^T

(a) and have receptors for ^a^T

(b) but have no receptors for ^i^T

(c) but will not produce nor secrete ^a^T

(iii) ^a^T will stimulate ^i^T protein/mRNA production by i-Cells;

^a^T receptor is presumed present

(iv) ^a^T will normally stimulate proliferation of i-Cells; ^a^T receptor is presumed present

(v) ^a^T will inhibit proliferation of i-Cells if in excess of a high level of ^i^T

(vi) the cells will have receptors for ^a^T but not for ^i^T;

^i^T will inhibit proliferation of i-Cells by binding ^a^T.

(vii) the cells will have no ^i^T receptor and will not secrete ^a^T.

Evidence from the IGF system supports the CTC model as does the evidence presented below from other interacting cells and/or potential Trefones.

***(i) ^a^T produced and its receptor ( ^a^R) present in different cells:-***

**1. Glial cell line-derived neurotrophic factor** (**GDNF) and its receptor**

GNDF is produced by rat Sertoli cells, while the GNDF receptors are expressed in the spermatogonial stem cells.

**2. Keratinocyte Growth Factor KGF) and its receptor**

KGF is expressed in theca cells of the bovine ovarian follicle and the KGF receptor (a FGFR-2b,a splice variant of the FGF family receptor member, FGFR-2) is expressed in granulosa cells.

**3.Substance P and its receptor**

In the dorsal motor nucleus of the vagus (DMV) in the brainstem of the rat, Substance P receptors are immunolocalized to the somatic and dendritic membranes of a subpopulation of neurons within the DMV, with the majority of this labeling being separate from the nerve fibres which contain Substance P .

**4. Hepatic Growth Factor/Scatter Factor (HGF) and its receptor (c-met)**

The expression of the gene for HGF is restricted to fibroblasts and other mesenchymal cells while the expression of the gene for the receptor (c-met) is confined to cells that do not produce the ligand.

In human placenta, mRNA for the hepatocyte growth factor (HGF) is expressed in the perivascular tissue around the stem villous arteries while the mRNA for the c-met is limited to the perivillous trophoblast. There was some overlap in protein immune-localization but where this occurred was likely due to receptor-bound ligand.

***(ii)* *^i^T produced by, and ^a^R present in, same cell:-***

**1.** The EGF receptor is present in the A431 human epidermoid carcinoma cell line and an extracellular binding protein is generated from the EGF receptor precursor. This would be indicative of an i-Cell with the ^i^T being the EGF binding protein.

**2.** The prostate cancer cell line LNCaP has properties of an i-Cell but with respect to growth hormone (GH). LNCaP cells have receptors for GH and secrete GH binding protein (GHBP - which is the exon 9-truncated isoform of the GH-receptor molecule). Also these cells do not secrete GH and GH causes stimulation of GHBP mRNA.

**3.** If the sex hormone-binding globulin (SHBG) is synthesized by estrogen-responsive cells (ie. by estrogen target cells), then this would be indicative of the presence of i-Cells. This occurs in the liver, testis, and Fallopian tube as certain cells are estrogen responsive and contain SHBG gene transcripts. The observation that SHBG and the estrogen receptor are colocalized in the human Fallopian tube within the epithelial cells of the ampulla and in muscle cells of the isthmus could then indicate the presence of i-Cells which respond to an estrogen (one Trefone) and produce a binding protein (SHBG - the couplet Trefone).

**4.** Human vascular smooth muscle cells (HVSMC) and human umbilical vein endothelial cells (HUVEC) release soluble uPAR (suPAR) which binds uPA as a potential couplet of Trefones. suPAR alone does not bind to HVSMC or HUVEC but a complex of uPA and suPAR (with vitronexin) does form on the cell surface. Again, this is indicative of a i-Cells that produce suPAR with a complex receptor for uPA.

***(iii) No ^a^T produced but ^a^R present:-***

No EGF is produced by the endometrial cell line RL95-2 but EGF receptors are present, both of which indicate an i-Cell type.

***(iv)*** ***^i^T stimulates ^a^T:-***

The soluble receptor for interleukin-6 (IL-6) stimulates the synthesis of IL-6 in human fibroblasts.

An FGF-binding protein (FGF-BP) exerts its biological function by parallel paracrine stimulation of tumor cell and endothelial cell proliferation through FGF-2 release.

**REFERENCES:-**

1.	de Rooij DG: **Proliferation and differentiation of spermatogonial stem cells**. *Reproduction* 2001, **121**(3):347-354.

2.	Parrott JA, Skinner MK: **Developmental and hormonal regulation of keratinocyte growth factor expression and action in the ovarian follicle**. *Endocrinology* 1998, **139**(1):228-235.

3.	Ladic LA, Buchan AM: **Three-dimensional spatial relationship of neuropeptides and receptors in the rat dorsal vagal complex**. *Brain Res* 1998, **795**(1-2):312-324.

4.	Birchmeier C, Gherardi E: **Developmental roles of HGF/SF and its receptor, the c-Met tyrosine kinase**. *Trends Cell Biol* 1998, **8**(10):404-410.

5.	Somerset DA, Li XF, Afford S, Strain AJ, Ahmed A, Sangha RK, Whittle MJ, Kilby MD: **Ontogeny of hepatocyte growth factor (HGF) and its receptor (c-met) in human placenta: reduced HGF expression in intrauterine growth restriction**. *Am J Pathol* 1998, **153**(4):1139-1147.

6.	Haigler H, Ash JF, Singer SJ, Cohen S: **Visualization by fluorescence of the binding and internalization of epidermal growth factor in human carcinoma cells A-431**. *Proc Natl Acad Sci U S A* 1978, **75**(7):3317-3321.

7.	Masui H, Castro L, Mendelsohn J: **Consumption of EGF by A431 cells: evidence for receptor recycling**. *J Cell Biol* 1993, **120**(1):85-93.

8.	Ullrich A, Coussens L, Hayflick JS, Dull TJ, Gray A, Tam AW, Lee J, Yarden Y, Libermann TA, Schlessinger J *et al*: **Human epidermal growth factor receptor cDNA sequence and aberrant expression of the amplified gene in A431 epidermoid carcinoma cells**. *Nature* 1984, **309**(5967):418-425.

9.	Reiter E, Kecha O, Hennuy B, Lardinois S, Klug M, Bruyninx M, Closset J, Hennen G: **Growth hormone directly affects the function of the different lobes of the rat prostate**. *Endocrinology* 1995, **136**(8):3338-3345.

10.	Weiss-Messer E, Merom O, Adi A, Karry R, Bidosee M, Ber R, Kaploun A, Stein A, Barkey RJ: **Growth hormone (GH) receptors in prostate cancer: gene expression in human tissues and cell lines and characterization, GH signaling and androgen receptor regulation in LNCaP cells**. *Mol Cell Endocrinol* 2004, **220**(1-2):109-123.

11.	Nakonechnaya AO, Jefferson HS, Chen X, Shewchuk BM: **Differential effects of exogenous and autocrine growth hormone on LNCaP prostate cancer cell proliferation and survival**. *J Cell Biochem* 2012.

12.	Bidosee M, Karry R, Weiss-Messer E, Barkey RJ: **Regulation of growth hormone receptors in human prostate cancer cell lines**. *Mol Cell Endocrinol* 2009, **309**(1-2):82-92.

13.	Noe G: **Sex hormone binding globulin expression and colocalization with estrogen receptor in the human Fallopian tube**. *Journal of Steroid Biochemistry & Molecular Biology* 1999, **68**(3-4):111-117.

14.	Chavakis T, Kanse SM, Yutzy B, Lijnen HR, Preissner KT: **Vitronectin concentrates proteolytic activity on the cell surface and extracellular matrix by trapping soluble urokinase receptor-urokinase complexes**. *Blood* 1998, **91**(7):2305-2312.

15.	Reynolds RK, Hu C, Baker VV: **Transforming growth factor-alpha and insulin-like growth factor-I, but not epidermal growth factor, elicit autocrine stimulation of mitogenesis in endometrial cancer cell lines**. *Gynecol Oncol* 1998, **70**(2):202-209.

16.	Lelle RJ, Talavera F, Gretz H, Roberts JA, Menon KM: **Epidermal growth factor receptor expression in three different human endometrial cancer cell lines**. *Cancer* 1993, **72**(2):519-525.

17.	Cichy J, Rose-John S, Pryjma J, Travis J: **Effect of soluble interleukin-6 receptor on interleukin-6 synthesis in human skin fibroblasts**. *Biochem Biophys Res Commun* 1996, **227**(2):318-321.

18.	Aigner A, Butscheid M, Kunkel P, Krause E, Lamszus K, Wellstein A, Czubayko F: **An FGF-binding protein (FGF-BP) exerts its biological function by parallel paracrine stimulation of tumor cell and endothelial cell proliferation through FGF-2 release**. *Int J Cancer* 2001, **92**(4):510-517.
