# Supplementary material for: A model to explain specific cellular communications and cellular harmony:- a hypothesis of coupled cells and interactive coupling molecules
Source: Theor Biol Med Model. 2014 Sep 14;11:40. doi: 10.1186/1742-4682-11-40 (PMC4237941; doi:10.1186/1742-4682-11-40)
Supplement: Additional file 1 — 1. “Terminology”. 2. “Change in Cellular Characteristics”. 3. Note 1 “The Roles of SCD versus ACD”. Note 2. “Categories of SCD and ACD”. Note 3 “Further Examples of these AsCD Categories”. Note 4 “Change of differentiation type.”. 4. “The Effects of Growth Factors and other Bioactive Molecules 5. Note 1, “Additional Information on the Definition of a Trefone”. Note 2 “Clarification on Ligand and Binding Protein Interaction”. Note 3 “Cell Couplets – AsCD and the a-Cell and i-Cell”. Note 4 “Drosophila – Potential Trefones and ACD”. 6. “Calculated Levels of Trefones and TCC, with Cellular Actions”. 7. “Receptors for the Six IGFBPs and Cellular Effects”. 8. Note 1 “Extracellular Binding Proteins/ Soluble Receptors”. Note 2 “Cell Receptors for Binding Proteins/Soluble Receptors”. 9. Note 1. “Further Evidence for a Receptor for TCC”. Note 2 “Problems with Cross-linking Experiments”. 10. “Evidence for Binary Receptors aside from the IGF System”. 11. “Evidence for Two Receptors for a Particular Trefone, outside the IGF System”. 12. Note 1 “Mechanisms of Nuclear Localization”. Note 2 “NL of IGFBPs”. Note 3 “NL of Potential Trefones other than the IGF System”. 13. “Evidence Supporting the Existence of a-Cells and i-Cells”. 14. “Candidates with mainly a-Cell-Type or i-Cell-Type Characteristics”. 15. “Interacting Cells and Trefones not associated with the IGF System”. 16. “Heterogeneity/Variability of Cells in Culture.” 17. “Cell lines are not Typical of Normal Cells”. 18. “Cell Receptors and Cell Markers”. 19. “Defined Couplet Cells for Insulin and Glucagon; Gastrin and Histamine”. 20. “Potential Couplet Trefones”. 21. “Further Examples of Potential Trefone and Cell Couplets”. 22. “Examples of Cellular Regulation by Complexes”. 23. “Proteolytic enzymes and their inhibitors”. 24. “Expanded Definition of ‘Trefone’ and Classes of Couplet Cell Interactions”. 25. Note 1 “Extended Trefone Couplets”. Note 2 “Singlet Cells”. 26. “Background of Cancer Research”. [file 1742-4682-11-40-S1.zip › 3484671921331230_add1/3484671921331230_add16.doc]

**Additional File *Sixteen***

***(iii) Reservations concerning the Validity of Experimental Interpretations derived from Cell-Culture Studies***

**(1) Heterogeneity/Variability of Cells in Culture.**

***Examples of Heterogeneity/Variability of Cells in Culture.***

*The purpose of this File is to justify the presence of two cells (the a-Cell and i-Cell) in the Trefone Couplet Cell model by providing extensive evidence of heterogeneity/variability of cells in culture, such that it is very feasible that a-Cells and i-Cells could coexist in cultures.*

Heterogeneity could be described as being extrinsic (with non-uniform environmental factors), genetic (with non-identical genomes), intrinsic (gene expression/temporal noise) or trait heterogeneity (which includes gene expression noise plus asynchronous oscillations, perhaps cell-cycle related, in both the individual cell and the population). This would be micro-heterogeneity as opposed to macro-heterogeneity which refers to a mixture of cells of distinct cell types or in obviously distinct states. Progenitor cells may be mixed with cells derived from these parental cells and this population heterogeneity is detectable by a multi-modal distribution of a particular trait. In the CTC model, the cells would be likely of the macro type with mixed cells from a parent but could also be micro type with non-identical genome expressions.

How is it known that a pure culture of cells is used in an experiment? Is the culture free from living organisms other than the species required and not contaminated by any foreign organisms ie. axenic, and is there only one type of cell from the species present? (**See Additional File *14***, ***Note 5*** and ***Note 6*** for some other observations on this issue.)

For a bacterial experiment, is there a spore present in a vegetative culture, which is not detected but relevant to experimental results? Indeed, there can be dormancy even in non-sporulating bacteria, which is broken in appropriate conditions to allow regrowth.

Does the cell composition of a cell line then change during an experiment? Some cells in the minority may only begin to grow and proliferate when the culture medium is changed or when a growth factor is added. In some cases then, experimental results are explained by the presence of a small percentage of cells that are induced to grow when the main cell type are less stimulated. The stimulatory effect of platelet-derived growth factor on “pure” oligodendrocyte cells could be explained in part by the effect of PDGF on “contaminating” progenitor cells that account for 10-15% of total cells.

Also, some cells in the minority may only begin to grow because the growth of the major cells in focus, changes the environment. Viable cells of Micrococcus luteus secrete a factor, which promotes the resuscitation and growth of dormant, nongrowing cells of the same organism.

Even within species, well-defined strains have been identified. For example, there are two groups of E. raikovi strains, one denoted PR and the other GA, that both respond to different pheromones, having specific receptors for each pheromone. Offspring strains of E. raikovi may be obtained in the laboratory from wild-type strains and these strains may have changed due to pheromones that are “aged” due to oxidation of methionine residues. The cells change in their response to the aged pheromone which no longer directs an autocrine activity to promote cell growth, but now directs a paracrine activity to induce cell unions in mating pairs of the selfing type (ie. of genetically identical cells). These pairs then generate viable offspring, just as pairs from genetically different cells do.

A change in cell environment caused by both the increasing products of growth and declining substances for growth due to increased cell density may well alter cell type even to cause differentiation. A “spontaneous” change occurs to susceptible fibroblast cells (3T3) when growth is arrested in culture, which leads to increased differentiation into adipose cells.- a change which is heritable. Other examples are described in **Table S1**.

**TABLE S1. Examples of Heterogeneity/Variability of Cells in Culture.**

| **Cell Type** | **Cell Variation** | **Comment or Observation and Reference** |  |
| --- | --- | --- | --- |
| Bacteria | Viability of cells | “The most fundamental questions such as whether a cell is alive, in the sense of being able to divide or to form a colony, may sometimes be very hard to answer, since even axenic microbial cultures are extremely heterogeneous.” |  |
| Bacteria | Definition of “Live” and “Dead” | “Groups of cells within a microbial population may exhibit heterogeneous uptake of fluorescent stains and thus be classified into more subpopulations than “live” and “dead.” The route from “live” to “dead” contains many [steps](http://www.ncbi.nlm.nih.gov/pmc/articles/PMC3165249/figure/F1/) (not all of which will occur or be observed to occur in all cases), and while the extremes are relatively clear-cut, the reversibility of these steps and indeed the moment of death are far from easy to define.” |  |
| Bacteria | Two or more vegetative cell types | The phrase ‘polymorphic vegetative cell cycle types’ describes species that may produce (under different nutrient and physiological regimes, but not so bizarre as to cause malfunction in the cell cycle) two or more morphologically distinct types of cell, each of which undergoes a distinctive and constant cell cycle (eg. *Arthrobacter* species, *Sphaerotilus* species, certain *cyanobacteria, Geodermatophilus, Hyphomicrobium* species, and *Rhodomicrobium* species; all of these have two- or more vegetative cell cycles). |  |
| *Micrococcus luteus* | Dormant cells | “[The number](http://www.ncbi.nlm.nih.gov/pmc/articles/PMC21177/) of observable microbial cells in a natural sample often exceeds the number that can be cultured there from by orders of magnitude). It is not known in general whether such nonculturable (and often noncultured) cells are dead, are killed by our media, or are in a dormant [state](http://www.ncbi.nlm.nih.gov/pmc/articles/PMC21177/) from which we could, in principle, resuscitate them with appropriate growth factors.” |  |
| *Blepharisma japonicum* (ciliate) | Two Mating types | “However, cells can spontaneously change their mating type in either direction (type I -> type II, type II -> type I) during asexual reproduction, or even change at every cell division in certain stocks.” |  |
| *Pseudo-monas putida* | Heterogeneous populations | Carbon-starvation may cause slow growth of subpopulation of cells at the expenses of dead cells and generate stationary-phase mutations in P. Putida. |  |
| Cell lines, generally | Change with time of growth | “From our Data we know that the population of primary cells becomes heterogeneous with time”…... and …. “the frequency of mutants can be extremely high …” |  |
| Fibroblasts (3T3) | Change with time of growth; two classes. | A fibroblast culture changes and cells differentiate into adipose cells; two sublines. |  |
| Dermal fibroblasts | Diverse populat-ion with distinct subpopulations | At least three subpopulations ; multiple subpopulations |  |
| Periodontal fibroblasts | Heterogeneity with subsets | Fibroblasts from human gingiva and periodontal ligament have subsets. |  |
| Fibrosar-coma cells  ( HT1080) | Multiple passages | Fibroblastoid phenotypic cells “spontaneously” convert into an epithelioid phenotype_’_ |  |
| Pancreatic beta cells | Affected by neighbours | The data show that B cells are heterogeneous in terms of their ability to release insulin and provide evidence that cell-to-cell adhesion and/or junctional communication regulate hormone secretion from individual B cells. |  |
| Pancreatic beta cells | Heterogeneous population | B cells ultrastructurally heterogeneous, in vivo. |  |
| Pancreatic beta cells | Heterogeneous expression | Based on expression of glucokinase. |  |
| Pancreatic beta cells | Two groups; Functional subpopulations | Based on secretion of insulin and other hormones;. the metabolically responsive (high responsive) and unresponsive (low responsive) subpopulations^,^ |  |
| Insulinoma cell line (RINm5F) | Wide range of heterogeneity | Based on morphology and hormone content between individual cells. |  |
| Pancreatic beta cell line (MIN6) | Change during growth | “..a sudden loss of glucose-induced insulin secretion from MIN6 cells during the course of the passage is sometimes noticed, possibly due to an outgrowth of cells with a poor response to glucose or a reduced expression of the genes responsible for glucose-induced insulin secretion.” “MIN6 is not a pure beta cell line but a mixed cell line with other pancreatic endocrine hormones” |  |
| Cultured human keratinocytes | Two populations | “These results may suggest the presence of two keratinocyte populations, one responding to EGF and one to IGF-I… or they may be explained on the basis of... ” |  |
| Cholangio-cytes | Heterogeneous | Cells are morphologically and functionally heterogeneous along the normal intrahepatic biliary tree. |  |
| Embryonic cortical precursors | Sub-populations | In studying “virtually pure” cultures of embryonic rat cortical precursors, the authors conceded that the effects observed for various mitogenic factors on these cells could have been due to different sub-populations. |  |
| Platelets | Sub-  populations | 1. Upon stimulation, ~30% of platelets are activated to express a surface-bound protein.  2. Expression of a phosphatase enzyme is restricted to a  subpopulation of ∼5% of platelets.  3. Vascular endothelial growth factor and endostatin are in distinct subpopulations of alpha-granules seemingly in different platelets. |  |
| Macrophages | Two or three types | Each type may be subdivided.  Foamy macrophages also heterogeneous. |  |
| Monocytes | Two major sub-sets |  |  |
| Chick embryo cells | Sub-populations | “….indicate that cell heterogeneity exists and is reflected in the metabolic responses of subpopulations to specific regulatory signals.” |  |
| Mouse embryo cells (C3H10T1/2) | < 50% of cells differentiated  (≥ 2 cell types) | Differentiation of clonally isolated cells to mature adipocytes |  |
| Smooth muscle cells (SMC)  (pulmonary arteries | Heterogeneity of SMC phenotype and function | “…. phenotypically distinct cells with enhanced growth potential exist within the normal arterial media, ..” |  |
| SMC from rat aorta | Two cell populations | “ half contains IP3 receptors and TG-sensitive Ca2+ pumps only; the other half contains, in addition to the IP3-sensitive pool independent from mitochondria, a caffeine-sensitive pool.” |  |
| Myoblasts (C2/C2C12) | Heterogeneity of sub-lines | IGFBP production varies from 1 to 3 types. |  |
| Neurons | Diversity of cell types | Traditional estimates of a few hundred mammalian neuronal subtypes appear to be overly conservative. |  |
| Cerebral neurons | Possible sub-population | Subsets based on immunoreactivity to Fos and neurotensin |  |
| Hypothal-amic non-neuronal cultured cells | Two populations | “At least two populations of non-neuronal hypothalamic cells were present in the cultures, and these were both affected by vasopressin-neurophysin.” |  |
| Hippocampal inhibitory cells | Sixteen morphological phenotypes; 25 response combinations | Subsets of inhibitory cells were excited or inhibited by agonists at receptors for noradrenaline, muscarine and/or, serotonin, and metabotropic glutamate receptors. |  |
| Growth hormone cells | Three types of cells | Within the anterior pituitary |  |
| Thyrotropes of anterior pituitary | A mosaic of cell phenotypes | A mosaic of cell types rather than a single cell type. |  |
| Gonadotropes | Multiple subsets | Multiple Subsets within the anterior pituitary |  |
| Leydig cells | Variable expression | “.. that not all Leydig cells exhibited androgen receptor immunostaining at steady state suggests a differential, functional activity of these cells within the population.”  Perhaps due to the five main cell types that are present in the Leydig cell lineage. |  |
| Oligodendro-cyte cultures | Cells with and cells without | In "pure" oligodendrocyte cultures, ~ half of cells were labeled with a marker for astrocytes. | |
| Erythrocytes | Cells with and cells without | Glucose-6-phosphate dehydrogenase (G6PD) deficiency is a X-chromosomally transmitted disorder of the erythrocyte Heterozygously-deficient women have a normal and a G6PD-deficient population of erythrocytes | |
| Lymphocytes and leukemic  cells | Variable expression | Populations of proliferating lymphocytes and MOLT-4 cells were very heterogeneous in terms of expression of D-type cyclins by individual cells. | |
| Lympho-blasts from myeloma marrow | Two types (I and II) | Two different cell fractions distinguished and separated by cytometry, based on high- and low-affinity insulin receptors | |
| Murine erythroleuk-emia cells | 3 types of colonies | Colonies where 1. All cells stain for heme 2. No cells stain for heme 3. There is a mixture of both cell types. | |
| Human cell line (NK-92) (activated natural killer cells) | Half of cells respond | "Characterization of a cell when tested at 100 U/ml of IL-2 and its expression correlates inversely with the IL-2 concentration. The p75 IL-2 receptor is expressed on about half of the cells at low density irrespective of the IL-2 concentration.” | |
| Colon adeno-carcinoma cell line (Caco-2) | Change with time of growth | Marker for enterocytic differentiation change:-  . sucrase-isomaltase mRNA increased 12-fold between days 5 and 16 of culture  . alkaline phosphatase progressively increased between days 7-13 in culture, temporally correlating with post-confluent phase of negligible growth.  . Carcinoembryonic antigen 16 fold from days 4 to 14 and IGFBP-2 and IGFBP-4 secretion decreased concomitantly. | |
| Epidermoid carcinoma cells (A431 cells) | Change with time of growth | “After more than a year in continuous culture, we noted loss of EGF-stimulated growth in three clones.” Two clones, independently derived from the parental clone 29 in separate laboratories, were strongly inhibited by EGF in contrast to the parental clone whose growth was stimulated by EGF The parent of clone 29, A431 cells, exhibited EGF-mediated growth inhibition so the two clones had reverted. | |
| Osteogenic sarcoma cell (UMR 106) | Change with late passages | “These data point to a significant phenotypic change having taken place in this clonal cell line with prolonged maintenance in culture with the emergence of a calcitonin receptor ...” | |
| Osteosarcoma cells (SaOS-2) | Sub-populations | One with low (LoSaOS) and one with high(Hi) alkaline phosphatase; the basal rate of IGFBP-3 secretion was higher by LoSaOS cells compared to HiSaOS cells. | |
| Endothelial hyperplasia in human glioblastoma | ‘In vitro’ *versus* ‘in vivo’ | “However, permanent cell lines are selected for long-term growth in culture and cannot be considered true representatives of the tumor cell population *in vivo”*. | |

*This Table lists examples (eg. of bacteria and cell lines) as evidence of the heterogeneity of cells in culture along with a comment or observation on this variability.*

The addition of biological stimulants may also produce differentiation, with changes in cell features and function**.** An example is the exposure of a pituitary tumour cell line (GH3) to epidermal growth factor for four days which induced a morphology change and a functional change to express dopamine receptors. Another is the exposure of 3T3-L1 cells (with a [fibroblast](http://en.wikipedia.org/wiki/Fibroblast)-like [morphology](http://en.wikipedia.org/wiki/Morphology_(biology))) to insulin to induce a differentiation into adipocytes.

HeLa cells, derived from a [cervical cancer](http://en.wikipedia.org/wiki/Cervical_cancer), are chromosomally abnormal and heterogeneous; they have a modal [chromosome](http://en.wikipedia.org/wiki/Chromosome) number of 82, with a range of 70 to 164 with various rearrangements, combinations, translocations and with four copies of chromosome [12](http://en.wikipedia.org/wiki/Chromosome_12) and 17 and three copies of chromosomes [6](http://en.wikipedia.org/wiki/Chromosome_6) and [8](http://en.wikipedia.org/wiki/Chromosome_8). The fact the cell populations have a modal number of chromosomes would mean that there are many different types of cells, producing therefore a heterogeneous mixture in a single cell line! And this may change with the number of passages – the prostatic carcinoma cell line (PC-3) showed a modal chromosome number of 62 at 5^th^ passage but 55 at the 50th passages.

In some cases of micro-heterogeneity, the cell population may exist as two classes or types. A division into the ‘haves’ (+) and ‘have-nots’(-) (eg. receptor present or not), would likely involve chromatin silencing where once the epigenetic mark is ‘set’ as a 'closed' or an 'open' configuration and the appropriate genes are ‘off’ or ‘on’, the elements remain in a silenced or activated state so that the phenotype may be maintained during subsequent mitoses by some sort of cellular memory. The binary decision to be a repressed or expressed molecule due to this inactive or active state of the gene could change upon exposure to a new environment of growth and/or differentiation factor to produce a new cell fate through cell division.

There may also be variation in cell type within apparently identical cells because of allelic expression differences. For example, in lymphoid tissues, the products of allelic genes are always present in different cells so that two alleles produce two populations of plasma cells, one containing one allotype and the other the alternative one. Similarly, in hereditary persistence of HbF, the condition in heterozygotes can be either *pancellular* where HbF is present in all erythrocytes, or *heterocellular* where about 50% of cells contain HbF and are named F cells. This latter heterocellular condition appears to involve an inherited allelic exclusion of an HPFH gene in approximately half the erythrocytes.

Even other red blood cells need not be identical in function. Due to random inactivation of one X chromosome in females, some progenitor cells of erythrocytes have one X chromosome active while others have the complementary X chromosome active. Thus a female heterozygous for glucose-6-phosphate dehydrogenase (G6PD) deficiency (an X-linked disease) may have two types of red blood cell in her blood, G6PD-normal or deficient depending on which X-chromosome is not inactivated.

Some cells in a bacterial colony may be different; eg. differences in the types and the number of multifunctional chemotaxis receptors and the possible variation induced by clustering on the cell surface to thereby influence each other's responses in these clusters . Four different receptors can be methylated at up to eight positions and each may have two or more possible conformations. There can possibly be then several thousand receptors that interact with each other in sets of three to form trimers, within each cluster. Each cell will be somewhat unique to respond to multiple environmental influences.

Micro-heterogeneity may also be associated with monoallelic transcription. It is associated with autosomal imprinted genes and with genes subject to X chromosome inactivation, but also is known to occur in a class known as randomly monoallelic autosomal genes eg. the immunoglobulins, receptor genes (odorant, T-cell, pheromone and natural killer cell receptors) and interleukins. More than 300 autosomal genes displaying random monoallelic expression have been identified, thus indicating that it is not uncommon.

The CTC model, if a valid one, would require future experiments to ensure initial cell homogeneity and focus on any change in cell types a reassessment of data derived from research that used cell cultures. The tacit assumption made by biologists about the homogeneity of cell populations needs experimental support in every case. The need is to distinguish between the interpretations of the past where cell regulation was presumed to involve activation/inhibition of identical cells (one cell on itself or one cell on a clonal/identical cell) and the CTC model where cell regulation in cell cultures, in tissues and organisms involves cell couplets with reciprocal interactive Trefones.

**REFERENCES:-**

1.	Huang S: **Non-genetic heterogeneity of cells in development: more than just noise**. *Development* 2009, **136**(23):3853-3862.

2.	Kaprelyants AS, Gottschal JC, Kell DB: **Dormancy in non-sporulating bacteria**. *FEMS Microbiol Rev* 1993, **10**(3-4):271-285.

3.	Fressinaud C, Vallat JM, Pouplard-Barthelaix A: **Platelet-derived growth factor partly prevents chemically induced oligodendrocyte death and improves myelin-like membranes repair in vitro**. *Glia* 1996, **16**(1):40-50.

4.	Mukamolova GV, Kaprelyants AS, Young DI, Young M, Kell DB: **A bacterial cytokine**. *Proc Natl Acad Sci U S A* 1998, **95**(15):8916-8921.

5.	Vallesi A, Ballarini P, Di Pretoro B, Alimenti C, Miceli C, Luporini P: **Autocrine, mitogenic pheromone receptor loop of the ciliate Euplotes raikovi: pheromone-induced receptor internalization**. *Eukaryot Cell* 2005, **4**(7):1221-1227.

6.	Di GG, Miceli C, Zahn R, Damberger F, Wuthrich K, Luporini P: **A structurally deviant member of the Euplotes raikovi pheromone family: Er-23**. *J Eukaryot Microbiol* 2002, **49**(1):86-92.

7.	Alimenti C, Vallesi A, Luporini P, Buonanno F, Ortenzi C: **Cell aging-induced methionine oxidation causes an autocrine to paracrine shift of the pheromone activity in the protozoan ciliate, Euplotes raikovi**. *Exp Cell Res* 2012, **318**(2):144-151.

8.	Green H, Kehinde O: **Spontaneous heritable changes leading to increased adipose conversion in 3T3 cells**. *Cell* 1976, **7**(1):105-113.

9.	Davey HM, Kell DB: **Flow cytometry and cell sorting of heterogeneous microbial populations: the importance of single-cell analyses**. *Microbiol Rev* 1996, **60**(4):641-696.

10.	Davey HM: **Life, death, and in-between: meanings and methods in microbiology**. *Appl Environ Microbiol* 2011, **77**(16):5571-5576.

11.	Whittenbury R, Dow CS: **Morphogenesis and differentiation in Rhodomicrobium vannielii and other budding and prosthecate bacteria**. *Bacteriol Rev* 1977, **41**(3):754-808.

12.	Sugiura M, Tanaka Y, Suzaki T, Harumoto T: **Alternative gene expression in type I and type II cells may enable further nuclear changes during conjugation of Blepharisma japonicum**. *Protist* 2012, **163**(2):204-216.

13.	Saumaa S, Tarassova K, Tark M, Tover A, Tegova R, Kivisaar M: **Involvement of DNA mismatch repair in stationary-phase mutagenesis during prolonged starvation of Pseudomonas putida**. *DNA Repair (Amst)* 2006, **5**(4):505-514.

14.	Terzi M: **Evolution of Somatic Cells in Vitro**. In: *Origin and Natural History of Cell Lines.* Edited by Barigozzi C. NY: Alan R Liss Inc.; 1978: 81-91.

15.	Chang TH, Polakis SE: **Differentiation of 3T3-L1 fibroblasts to adipocytes. Effect of insulin and indomethacin on the levels of insulin receptors**. *J Biol Chem* 1978, **253**(13):4693-4696.

16.	Sorrell JM, Caplan AI: **Fibroblast heterogeneity: more than skin deep**. *J Cell Sci* 2004, **117**(Pt 5):667-675.

17.	Sorrell JM, Caplan AI: **Fibroblasts-a diverse population at the center of it all**. *Int Rev Cell Mol Biol* 2009, **276**:161-214.

18.	Phipps RP, Borrello MA, Blieden TM: **Fibroblast heterogeneity in the periodontium and other tissues**. *J Periodontal Res* 1997, **32**(1 Pt 2):159-165.

19.	Selvais C, D'Auria L, Tyteca D, Perrot G, Lemoine P, Troeberg L, Dedieu S, Noel A, Nagase H, Henriet P *et al*: **Cell cholesterol modulates metalloproteinase-dependent shedding of low-density lipoprotein receptor-related protein-1 (LRP-1) and clearance function**. *Faseb J* 2011, **25**(8):2770-2781.

20.	Frisch SM: **E1a induces the expression of epithelial characteristics**. *J Cell Biol* 1994, **127**(4):1085-1096.

21.	Salomon D, Meda P: **Heterogeneity and contact-dependent regulation of hormone secretion by individual B cells**. *Exp Cell Res* 1986, **162**(2):507-520.

22.	Stefan Y, Meda P, Neufeld M, Orci L: **Stimulation of insulin secretion reveals heterogeneity of pancreatic B cells in vivo**. *J Clin Invest* 1987, **80**(1):175-183.

23.	Jetton TL, Magnuson MA: **Heterogeneous expression of glucokinase among pancreatic beta cells**. *Proc Natl Acad Sci U S A* 1992, **89**(7):2619-2623.

24.	Pipeleers DG: **Heterogeneity in pancreatic beta-cell population**. *Diabetes* 1992, **41**(7):777-781.

25.	Giordano E, Bosco D, Cirulli V, Meda P: **Repeated glucose stimulation reveals distinct and lasting secretion patterns of individual rat pancreatic B cells**. *J Clin Invest* 1991, **87**(6):2178-2185.

26.	Hiriart M, Ramirez-Medeles MC: **Functional subpopulations of individual pancreatic B-cells in culture**. *Endocrinology* 1991, **128**(6):3193-3198.

27.	de Vargas LM, Sobolewski J, Siegel R, Moss LG: **Individual beta cells within the intact islet differentially respond to glucose**. *J Biol Chem* 1997, **272**(42):26573-26577.

28.	Katsuta H, Akashi T, Katsuta R, Nagaya M, Kim D, Arinobu Y, Hara M, Bonner-Weir S, Sharma AJ, Akashi K *et al*: **Single pancreatic beta cells co-express multiple islet hormone genes in mice**. *Diabetologia* 2009, **53**(1):128-138.

29.	Bargsten G: **Cytological and immunocytochemical characterization of the insulin secreting insulinoma cell line RINm5F**. *Arch Histol Cytol* 2004, **67**(1):79-94.

30.	Minami K, Yano H, Miki T, Nagashima K, Wang CZ, Tanaka H, Miyazaki JI, Seino S: **Insulin secretion and differential gene expression in glucose-responsive and -unresponsive MIN6 sublines**. *Am J Physiol Endocrinol Metab* 2000, **279**(4):E773-781.

31.	Nakashima K, Kanda Y, Hirokawa Y, Kawasaki F, Matsuki M, Kaku K: **MIN6 is not a pure beta cell line but a mixed cell line with other pancreatic endocrine hormones**. *Endocr J* 2009, **56**(1):45-53.

32.	Ristow HJ: **Studies on stimulation of DNA synthesis with epidermal growth factor and insulin-like growth factor-I in cultured human keratinocytes**. *Growth Regul* 1996, **6**(2):96-109.

33.	Alpini G, Roberts S, Kuntz SM, Ueno Y, Gubba S, Podila PV, LeSage G, LaRusso NF: **Morphological, molecular, and functional heterogeneity of cholangiocytes from normal rat liver**. *Gastroenterology* 1996, **110**(5):1636-1643.

34.	Lu N, DiCicco-Bloom E: **Pituitary adenylate cyclase-activating polypeptide is an autocrine inhibitor of mitosis in cultured cortical precursor cells**. *Proc Natl Acad Sci U S A* 1997, **94**(7):3357-3362.

35.	Alberio L, Safa O, Clemetson KJ, Esmon CT, Dale GL: **Surface expression and functional characterization of alpha-granule factor V in human platelets: effects of ionophore A23187, thrombin, collagen, and convulxin**. *Blood* 2000, **95**(5):1694-1702.

36.	Smyth SS, Sciorra VA, Sigal YJ, Pamuklar Z, Wang Z, Xu Y, Prestwich GD, Morris AJ: **Lipid phosphate phosphatases regulate lysophosphatidic acid production and signaling in platelets: studies using chemical inhibitors of lipid phosphate phosphatase activity**. *J Biol Chem* 2003, **278**(44):43214-43223.

37.	Italiano JE, Jr., Richardson JL, Patel-Hett S, Battinelli E, Zaslavsky A, Short S, Ryeom S, Folkman J, Klement GL: **Angiogenesis is regulated by a novel mechanism: pro- and antiangiogenic proteins are organized into separate platelet alpha granules and differentially released**. *Blood* 2008, **111**(3):1227-1233.

38.	Gordon S, Taylor PR: **Monocyte and macrophage heterogeneity**. *Nat Rev Immunol* 2005, **5**(12):953-964.

39.	Johnson JL, Newby AC: **Macrophage heterogeneity in atherosclerotic plaques**. *Curr Opin Lipidol* 2009, **20**(5):370-378.

40.	Baseman JB, Hayes NS: **Differential effect of hormones on macromolecular synthesis and mitosis in chick embryo cells**. *J Cell Biol* 1975, **67**(2PT.1):492-498.

41.	Cho YC, Jefcoate CR: **PPARgamma1 synthesis and adipogenesis in C3H10T1/2 cells depends on S-phase progression, but does not require mitotic clonal expansion**. *J Cell Biochem* 2004, **91**(2):336-353.

42.	Frid MG, Aldashev AA, Dempsey EC, Stenmark KR: **Smooth muscle cells isolated from discrete compartments of the mature vascular media exhibit unique phenotypes and distinct growth capabilities**. *Circ Res* 1997, **81**(6):940-952.

43.	Vallot O, Combettes L, Lompre AM: **Functional coupling between the caffeine/ryanodine-sensitive Ca2+ store and mitochondria in rat aortic smooth muscle cells**. *Biochem J* 2001, **357**(Pt 2):363-371.

44.	Damon SE, Haugk KL, Birnbaum RS, Quinn LS: **Retrovirally mediated overexpression of insulin-like growth factor binding protein 4: evidence that insulin-like growth factor is required for skeletal muscle differentiation**. *Journal of Cellular Physiology* 1998, **175**(1):109-120.

45.	Stevens CF: **Neuronal diversity: too many cell types for comfort?** *Curr Biol* 1998, **8**(20):R708-710.

46.	Senger B, Brog JS, Zahm DS: **Subsets of neurotensin-immunoreactive neurons in the rat striatal complex following antagonism of the dopamine D2 receptor: an immunohistochemical double-labeling study using antibodies against Fos**. *Neuroscience* 1993, **57**(3):649-660.

47.	Worley RT, Pickering BT: **Non-neuronal cells of rat hypothalamus in dissociated cell culture. Evidence that neurophysin modulates growth and DNA synthesis of non-neuronal cells**. *Cell Tissue Res* 1984, **237**(1):161-168.

48.	Parra P, Gulyas AI, Miles R: **How many subtypes of inhibitory cells in the hippocampus?** *Neuron* 1998, **20**(5):983-993.

49.	Kurosumi K, Koyama T, Tosaka H: **Three types of growth hormone cells of the rat anterior pituitary as revealed by immunoelectron microscopy using a colloidal gold-antibody method**. *Arch Histol Jpn* 1986, **49**(2):227-242.

50.	Villalobos C, Nunez L, Garcia-Sancho J: **Anterior pituitary thyrotropes are multifunctional cells**. *Am J Physiol Endocrinol Metab* 2004, **287**(6):E1166-1170.

51.	Childs GV: **Cytochemical studies of multifunctional gonadotropes**. *Microsc Res Tech* 1997, **39**(2):114-130.

52.	Vornberger W, Prins G, Musto NA, Suarez-Quian CA: **Androgen receptor distribution in rat testis: new implications for androgen regulation of spermatogenesis**. *Endocrinology* 1994, **134**(5):2307-2316.

53.	Mendis-Handagama SM, Ariyaratne HB: **Differentiation of the adult Leydig cell population in the postnatal testis**. *Biol Reprod* 2001, **65**(3):660-671.

54.	Fressinaud C, Weinrauder H, Delaunoy JP, Tholey G, Labourdette G, Sarlieve LL: **Glutamine synthetase expression in rat oligodendrocytes in culture: regulation by hormones and growth factors**. *J Cell Physiol* 1991, **149**(3):459-468.

55.	Peters AL, Van Noorden CJ: **Glucose-6-phosphate dehydrogenase deficiency and malaria: cytochemical detection of heterozygous G6PD deficiency in women**. *J Histochem Cytochem* 2009, **57**(11):1003-1011.

56.	Gong J, Bhatia U, Traganos F, Darzynkiewicz Z: **Expression of cyclins A, D2 and D3 in individual normal mitogen stimulated lymphocytes and in MOLT-4 leukemic cells analyzed by multiparameter flow cytometry**. *Leukemia* 1995, **9**(5):893-899.

57.	Jehle PM, Lutz MP, Fussgaenger RD: **High affinity binding sites for proinsulin in human IM-9 lymphoblasts**. *Diabetologia* 1996, **39**(4):421-432.

58.	Gusella J, Geller R, Clarke B, Weeks V, Housman D: **Commitment to erythroid differentiation by friend erythroleukemia cells: a stochastic analysis**. *Cell* 1976, **9**(2):221-229.

59.	Gong JH, Maki G, Klingemann HG: **Characterization of a human cell line (NK-92) with phenotypical and functional characteristics of activated natural killer cells**. *Leukemia* 1994, **8**(4):652-658.

60.	Park JH, Corkins MR, Vanderhoof JA, Caruso NM, Hrbek MJ, Schaffer BS, Slentz DH, McCusker RH, MacDonald RG: **Expression of insulin-like growth factor-II and insulin-like growth factor binding proteins during Caco-2 cell proliferation and differentiation**. *J Cell Physiol* 1996, **166**(2):396-406.

61.	Singh P, Dai B, Yallampalli U, Lu X, Schroy PC: **Proliferation and differentiation of a human colon cancer cell line (CaCo2) is associated with significant changes in the expression and secretion of insulin-like growth factor (IGF) IGF-II and IGF binding protein-4: role of IGF-II**. *Endocrinology* 1996, **137**(5):1764-1774.

62.	Street ME, Miraki-Moud F, Sanderson IR, Savage MO, Giovannelli G, Bernasconi S, Camacho-Hubner C: **Interleukin-1beta (IL-1beta) and IL-6 modulate insulin-like growth factor-binding protein (IGFBP) secretion in colon cancer epithelial (Caco-2) cells**. *J Endocrinol* 2003, **179**(3):405-415.

63.	Kawamoto T, Mendelsohn J, Le A, Sato GH, Lazar CS, Gill GN: **Relation of epidermal growth factor receptor concentration to growth of human epidermoid carcinoma A431 cells**. *J Biol Chem* 1984, **259**(12):7761-7766.

64.	Forrest SM, Ng KW, Findlay DM, Michelangeli VP, Livesey SA, Partridge NC, Zajac JD, Martin TJ: **Characterization of an osteoblast-like clonal cell line which responds to both parathyroid hormone and calcitonin**. *Calcif Tissue Int* 1985, **37**(1):51-56.

65.	Nakao Y, Hilliker S, Baylink DJ, Mohan S: **Studies on the regulation of insulin-like growth factor binding protein 3 secretion in human osteosarcoma cells in vitro**. *J Bone Miner Res* 1994, **9**(6):865-872.

66.	Hermansson M, Nister M, Betsholtz C, Heldin CH, Westermark B, Funa K: **Endothelial cell hyperplasia in human glioblastoma: coexpression of mRNA for platelet-derived growth factor (PDGF) B chain and PDGF receptor suggests autocrine growth stimulation**. *Proc Natl Acad Sci U S A* 1988, **85**(20):7748-7752.

67.	Missale C, Castelletti L, Boroni F, Memo M, Spano P: **Epidermal growth factor induces the functional expression of dopamine receptors in the GH3 cell line**. *Endocrinology* 1991, **128**(1):13-20.

68.	Reed BC, Kaufmann SH, Mackall JC, Student AK, Lane MD: **Alterations in insulin binding accompanying differentiation of 3T3-L1 preadipocytes**. *Proc Natl Acad Sci U S A* 1977, **74**(11):4876-4880.

69.	Macville M, Schrock E, Padilla-Nash H, Keck C, Ghadimi BM, Zimonjic D, Popescu N, Ried T: **Comprehensive and definitive molecular cytogenetic characterization of HeLa cells by spectral karyotyping**. *Cancer Res* 1999, **59**(1):141-150.

70.	Ohnuki Y, Marnell MM, Babcock MS, Lechner JF, Kaighn ME: **Chromosomal analysis of human prostatic adenocarcinoma cell lines**. *Cancer Res* 1980, **40**(3):524-534.

71.	Lyko F, Paro R: **Chromosomal elements conferring epigenetic inheritance**. *Bioessays* 1999, **21**(10):824-832.

72.	Mahmoudi T, Verrijzer CP: **Chromatin silencing and activation by Polycomb and trithorax group proteins**. *Oncogene* 2001, **20**(24):3055-3066.

73.	Pernis B, Chiappino G, Kelus AS, Gell PG: **Cellular localization of immunoglobulins with different allotypic specificities in rabbit lymphoid tissues**. *J Exp Med* 1965, **122**(5):853-876.

74.	Boyer SH, Margolet L, Boyer ML, Huisman TH, Schroeder WA, Wood WG, Weatherall DJ, Clegg JB, Cartner R: **Inheritance of F cell frequency in heterocellular hereditary persistence of fetal hemoglobin: an example of allelic exclusion**. *Am J Hum Genet* 1977, **29**(3):256-271.

75.	Bray D: **Genomics. Molecular prodigality**. *Science* 2003, **299**(5610):1189-1190.

76.	Dutta D, Ensminger AW, Zucker JP, Chess A: **Asynchronous replication and autosome-pair non-equivalence in human embryonic stem cells**. *PLoS One* 2009, **4**(3):e4970.

77.	Gimelbrant A, Hutchinson JN, Thompson BR, Chess A: **Widespread monoallelic expression on human autosomes**. *Science* 2007, **318**(5853):1136-1140.

78.	Huang S: **Systems biology of stem cells: three useful perspectives to help overcome the paradigm of linear pathways**. *Philos Trans R Soc Lond B Biol Sci* 2011, **366**(1575):2247-2259.
