# Supplementary material for: A model to explain specific cellular communications and cellular harmony:- a hypothesis of coupled cells and interactive coupling molecules
Source: Theor Biol Med Model. 2014 Sep 14;11:40. doi: 10.1186/1742-4682-11-40 (PMC4237941; doi:10.1186/1742-4682-11-40)
Supplement: Additional file 1 — 1. “Terminology”. 2. “Change in Cellular Characteristics”. 3. Note 1 “The Roles of SCD versus ACD”. Note 2. “Categories of SCD and ACD”. Note 3 “Further Examples of these AsCD Categories”. Note 4 “Change of differentiation type.”. 4. “The Effects of Growth Factors and other Bioactive Molecules 5. Note 1, “Additional Information on the Definition of a Trefone”. Note 2 “Clarification on Ligand and Binding Protein Interaction”. Note 3 “Cell Couplets – AsCD and the a-Cell and i-Cell”. Note 4 “Drosophila – Potential Trefones and ACD”. 6. “Calculated Levels of Trefones and TCC, with Cellular Actions”. 7. “Receptors for the Six IGFBPs and Cellular Effects”. 8. Note 1 “Extracellular Binding Proteins/ Soluble Receptors”. Note 2 “Cell Receptors for Binding Proteins/Soluble Receptors”. 9. Note 1. “Further Evidence for a Receptor for TCC”. Note 2 “Problems with Cross-linking Experiments”. 10. “Evidence for Binary Receptors aside from the IGF System”. 11. “Evidence for Two Receptors for a Particular Trefone, outside the IGF System”. 12. Note 1 “Mechanisms of Nuclear Localization”. Note 2 “NL of IGFBPs”. Note 3 “NL of Potential Trefones other than the IGF System”. 13. “Evidence Supporting the Existence of a-Cells and i-Cells”. 14. “Candidates with mainly a-Cell-Type or i-Cell-Type Characteristics”. 15. “Interacting Cells and Trefones not associated with the IGF System”. 16. “Heterogeneity/Variability of Cells in Culture.” 17. “Cell lines are not Typical of Normal Cells”. 18. “Cell Receptors and Cell Markers”. 19. “Defined Couplet Cells for Insulin and Glucagon; Gastrin and Histamine”. 20. “Potential Couplet Trefones”. 21. “Further Examples of Potential Trefone and Cell Couplets”. 22. “Examples of Cellular Regulation by Complexes”. 23. “Proteolytic enzymes and their inhibitors”. 24. “Expanded Definition of ‘Trefone’ and Classes of Couplet Cell Interactions”. 25. Note 1 “Extended Trefone Couplets”. Note 2 “Singlet Cells”. 26. “Background of Cancer Research”. [file 1742-4682-11-40-S1.zip › 3484671921331230_add1/3484671921331230_add17.doc]

**Additional File** ***Seventeen***

***(iii) Reservations concerning the Validity of Experimental Interpretations derived from Cell-Culture Studies***

**(2) Are Cell Lines Appropriate Models for an Understanding of Normal Cell Activities?**

***Cell lines are not Typical of Normal Cells; Warning - Deductions with Care.***

*The purpose of this File is to provide evidence to support the concept that the forest of information from cell lines may be camouflaging the properties of normal trees.*

Cancer cells generally have an abnormal quantity and quality of chromosomes with consequent expectations of deviations from normality by definition.

**Cell Lines are not normal cells**

Immortalized cells are often transformed cells and often give rise to tumors when injected into animals. Cell lines generally undergo many chromosomal alterations in their generation – they may become initially diploid, but later shift to a tetraploid range and become aneuploid, as documented for 3T3 cells in the generation of spontaneously immortalized cells.

Cytogenetic analysis of the human prostatic carcinoma cell line PC-3, also shows abnormality. “The line is near-triploid with a modal number of 62 chromosomes. There are nearly 20 marker chromosomes commonly found in each cell; and normal N2, N3, N4, N5, N12, and N15 are not found. No normal Y chromosomes could be detected by Q-band analysis” (ATCC data). Another human prostate carcinoma cell line, DU 145, has an aneuploid human karyotype with a modal chromosome number of 64 .

Human Embryonic Kidney (HEK 293) cells are also a hypotriploid with nearly three times the number of chromosomes of a normal haploid human cell, with a [modal](http://en.wikipedia.org/wiki/Mode_(statistics)) chromosome number of 64 occurring in 30% of cells. Chromosomal abnormalities include a total of three copies of the X chromosome and four copies of chromosome N17 and N22 (ATCC data).

Breast cancer cell lines are also abnormal; the Hs578T cell line is aneuploid as is MCF-7. The latter has extensive aneuploidy with both numerical and structural abnormalities and a wide range of heteroploidy. Even MCF-10A cells, generally considered to be a normal breast epithelial cell line, have genetic abnormalities including a spontaneous reciprocal translocation between chromosomes 3 and 9 plus other chromosome changes.

HeLa cells, derived from a [cervical cancer](http://en.wikipedia.org/wiki/Cervical_cancer), are chromosomally abnormal; they have a modal [chromosome](http://en.wikipedia.org/wiki/Chromosome) number of 82, with a range of 70 to 164, with various rearrangements, combinations, translocations and with four copies of chromosome [12](http://en.wikipedia.org/wiki/Chromosome_12) and 17 and three copies of chromosomes [6](http://en.wikipedia.org/wiki/Chromosome_6) and [8](http://en.wikipedia.org/wiki/Chromosome_8). The modal number may change with passages – the prostatic carcinoma cell line (PC-3) shows a modal chromosome number of 62 at 5^th^ passage but 55 at the 50th passage.

**Cellular characteristics may be misleading.**

Cell lines may change and differentiate in culture. 3T3-L1 is a [cell line](http://en.wikipedia.org/wiki/Cell_line) derived from [3T3 cells](http://en.wikipedia.org/wiki/3T3_cells) that is used in biological research on [adipose tissue](http://en.wikipedia.org/wiki/Adipose_tissue). These 3T3-L1 cells have now a [fibroblast](http://en.wikipedia.org/wiki/Fibroblast)-like [morphology](http://en.wikipedia.org/wiki/Morphology_(biology)), but, under appropriate [conditions](http://en.wikipedia.org/wiki/3T3-L1), the cells change a second time and [differentiate](http://en.wikipedia.org/wiki/Cellular_differentiation) into an [adipocyte](http://en.wikipedia.org/wiki/Adipocyte)-like [phenotype](http://en.wikipedia.org/wiki/Phenotype).

Cell lines may not be what they seem to be. Human Embryonic Kidney 293 cells were generated about 40 years ago and used as example of kidney cells until, about ten years ago, it was reported that they were likely transformed from a neuronal lineage cell in the original kidney culture and were not to be considered to be typical renal cells.

The other area of misleading or erroneous results resulted from the contamination of cells, especially with HeLa cells. This has been discussed in SI 16, in the section on the “heterogeneity” of cells used in experiments.

**Do the experimental results have relevance to real life?**

An example comes from hormones and the pancreas. With reference to the relationship between insulin, glucagon and somatostatin within islet cells, the following comment questions the relevance of monolayer growth to tissue growth. “Any methodical intervention to measure changes may alter normal anatomy and physiology. For example, even if paracrine events were convincingly demonstrated in monolayer islet cell cultures, or dispersed islet cells, or isolated islets, the relevance to possible paracrine events in the complex islet micro-organ of the living intact cell is problematic.”

A similar question is raised with respect to MCF-10A cells, generally considered to be a normal breast epithelial cell line. Is there a loss of architectural cues of mammary cells associated with immortalization and long-term culture in vitro? “The ducts and lobules that make up the mammary gland are distinguished by the presence of a central hollow lumen enclosed by an epithelium composed of multiple cell types, including luminal secretory cells, and the surrounding myoepithelial cells and basal cells”. The immortalization of primary human mammary epithelial cells in culture results in the outgrowth of only basal epithelial cells in a disordered and unnatural microenvironment compared to the described architecture.

Additional differences have been described. A tumour-derived beta-cell line (TC-3) does not express functional glucagon receptors but isolated rat pancreatic beta-cells, subpopulations of alpha- and delta-cells and also an insulinoma cell line (INS-1) have specific glucagon receptors. In breast carcinomas, receptors for somatostatin are not always highly expressed whereas the animal tumour models and cultured tumour cell lines usually have a high level of expression.

When gene expression of cellular pathways of normal tissue, tumours and cancer cell lines were compared, tumour tissue was closer to normal tissue than it was to the corresponding cell line. Indeed the cancer cell lines may not represent the tumour cancer itself but may be a minor cell from the mixture of cells of a tumour. Breast cancer cell (BCC) lines are often viewed as not being representative of the original tumours. The clonal nature of the cell line could not reflect the heterogeneity of the tumour with a mixture of variously differentiated cells. In addition, as cancer cells are viewed as genetically unstable with phenotypic changes likely in long-term cultures in an environment different from within the tumour, sub-clones are likely to occur and be different in different laboratories. However, one study has concluded that BCC lines are likely to reflect, to a large extent, the features of cancer cells in vivo. Others have shown that tumor-derived stem cells differ from traditionally-grown tumor cell lines.

In a comparison of 20 laryngeal squamous cell lines with their primary carcinomas, overexpression of 739 genes could be attributed to gene amplification events in cell lines, with only 325 genes showing the same amplification in the primary cancers. It follows that extrapolation from cell line to real cancers can be invalid. Differences in the methylation of tumour-associated genes, acetylation of histones and loss of differentiation also obscure the validity of extrapolation.

The lack of a valid model in diabetes research is reflected in the ongoing search for the perfect one. With limited success in 30 years, there is still a need to establish a "normal" beta cell line preferably of human origin.

**REFERENCES:-**

1.	Todaro GJ, Green H: **Quantitative studies of the growth of mouse embryo cells in culture and their development into established lines**. *J Cell Biol* 1963, **17**:299-313.

2.	Stone KR, Mickey DD, Wunderli H, Mickey GH, Paulson DF: **Isolation of a human prostate carcinoma cell line (DU 145)**. *Int J Cancer* 1978, **21**(3):274-281.

3.	Hackett AJ, Smith HS, Springer EL, Owens RB, Nelson-Rees WA, Riggs JL, Gardner MB: **Two syngeneic cell lines from human breast tissue: the aneuploid mammary epithelial (Hs578T) and the diploid myoepithelial (Hs578Bst) cell lines**. *J Natl Cancer Inst* 1977, **58**(6):1795-1806.

4.	Whang-Peng J, Lee EC, Kao-Shan CS, Seibert K, Lippman M: **Cytogenetic studies of human breast cancer lines: MCF-7 and derived variant sublines**. *J Natl Cancer Inst* 1983, **71**(4):687-695.

5.	Soule HD, Maloney TM, Wolman SR, Peterson WD, Jr., Brenz R, McGrath CM, Russo J, Pauley RJ, Jones RF, Brooks SC: **Isolation and characterization of a spontaneously immortalized human breast epithelial cell line, MCF-10**. *Cancer Res* 1990, **50**(18):6075-6086.

6.	Macville M, Schrock E, Padilla-Nash H, Keck C, Ghadimi BM, Zimonjic D, Popescu N, Ried T: **Comprehensive and definitive molecular cytogenetic characterization of HeLa cells by spectral karyotyping**. *Cancer Res* 1999, **59**(1):141-150.

7.	Ohnuki Y, Marnell MM, Babcock MS, Lechner JF, Kaighn ME: **Chromosomal analysis of human prostatic adenocarcinoma cell lines**. *Cancer Res* 1980, **40**(3):524-534.

8.	Zebisch K, Voigt V, Wabitsch M, Brandsch M: **Protocol for effective differentiation of 3T3-L1 cells to adipocytes**. *Anal Biochem* 2012, **425**(1):88-90.

9.	Shaw G, Morse S, Ararat M, Graham FL: **Preferential transformation of human neuronal cells by human adenoviruses and the origin of HEK 293 cells**. *Faseb J* 2002, **16**(8):869-871.

10.	Samols E, Bonner-Weir S, Weir GC: **Intra-islet insulin-glucagon-somatostatin relationships**. *Clin Endocrinol Metab* 1986, **15**(1):33-58.

11.	Debnath J, Muthuswamy SK, Brugge JS: **Morphogenesis and oncogenesis of MCF-10A mammary epithelial acini grown in three-dimensional basement membrane cultures**. *Methods* 2003, **30**(3):256-268.

12.	Kieffer TJ, Heller RS, Unson CG, Weir GC, Habener JF: **Distribution of glucagon receptors on hormone-specific endocrine cells of rat pancreatic islets**. *Endocrinology* 1996, **137**(11):5119-5125.

13.	Reubi JC, Wuthrich F, Reubi F: **Peptide receptors as molecular targets for cancer diagnosis and therapy**. *Endocr Rev* 2003, **24**(4):389-427.

14.	Ertel A, Verghese A, Byers SW, Ochs M, Tozeren A: **Pathway-specific differences between tumor cell lines and normal and tumor tissue cells**. *Mol Cancer* 2006, **5**(1):55.

15.	Lacroix M, Leclercq G: **Relevance of breast cancer cell lines as models for breast tumours: an update**. *Breast Cancer Res Treat* 2004, **83**(3):249-289.

16.	Lee J, Kotliarova S, Kotliarov Y, Li A, Su Q, Donin NM, Pastorino S, Purow BW, Christopher N, Zhang W *et al*: **Tumor stem cells derived from glioblastomas cultured in bFGF and EGF more closely mirror the phenotype and genotype of primary tumors than do serum-cultured cell lines**. *Cancer Cell* 2006, **9**(5):391-403.

17.	Jarvinen AK, Autio R, Haapa-Paananen S, Wolf M, Saarela M, Grenman R, Leivo I, Kallioniemi O, Makitie AA, Monni O: **Identification of target genes in laryngeal squamous cell carcinoma by high-resolution copy number and gene expression microarray analyses**. *Oncogene* 2006, **25**(52):6997-7008.

18.	van Staveren WC, Solis DY, Hebrant A, Detours V, Dumont JE, Maenhaut C: **Human cancer cell lines: Experimental models for cancer cells in situ? For cancer stem cells?** *Biochim Biophys Acta* 2009, **1795**(2):92-103.

19.	Skelin M, Rupnik M, Cencic A: **Pancreatic beta cell lines and their applications in diabetes mellitus research**. *ALTEX* 2010, **27**(2):105-113.
