# Supplementary material for: A model to explain specific cellular communications and cellular harmony:- a hypothesis of coupled cells and interactive coupling molecules
Source: Theor Biol Med Model. 2014 Sep 14;11:40. doi: 10.1186/1742-4682-11-40 (PMC4237941; doi:10.1186/1742-4682-11-40)
Supplement: Additional file 1 — 1. “Terminology”. 2. “Change in Cellular Characteristics”. 3. Note 1 “The Roles of SCD versus ACD”. Note 2. “Categories of SCD and ACD”. Note 3 “Further Examples of these AsCD Categories”. Note 4 “Change of differentiation type.”. 4. “The Effects of Growth Factors and other Bioactive Molecules 5. Note 1, “Additional Information on the Definition of a Trefone”. Note 2 “Clarification on Ligand and Binding Protein Interaction”. Note 3 “Cell Couplets – AsCD and the a-Cell and i-Cell”. Note 4 “Drosophila – Potential Trefones and ACD”. 6. “Calculated Levels of Trefones and TCC, with Cellular Actions”. 7. “Receptors for the Six IGFBPs and Cellular Effects”. 8. Note 1 “Extracellular Binding Proteins/ Soluble Receptors”. Note 2 “Cell Receptors for Binding Proteins/Soluble Receptors”. 9. Note 1. “Further Evidence for a Receptor for TCC”. Note 2 “Problems with Cross-linking Experiments”. 10. “Evidence for Binary Receptors aside from the IGF System”. 11. “Evidence for Two Receptors for a Particular Trefone, outside the IGF System”. 12. Note 1 “Mechanisms of Nuclear Localization”. Note 2 “NL of IGFBPs”. Note 3 “NL of Potential Trefones other than the IGF System”. 13. “Evidence Supporting the Existence of a-Cells and i-Cells”. 14. “Candidates with mainly a-Cell-Type or i-Cell-Type Characteristics”. 15. “Interacting Cells and Trefones not associated with the IGF System”. 16. “Heterogeneity/Variability of Cells in Culture.” 17. “Cell lines are not Typical of Normal Cells”. 18. “Cell Receptors and Cell Markers”. 19. “Defined Couplet Cells for Insulin and Glucagon; Gastrin and Histamine”. 20. “Potential Couplet Trefones”. 21. “Further Examples of Potential Trefone and Cell Couplets”. 22. “Examples of Cellular Regulation by Complexes”. 23. “Proteolytic enzymes and their inhibitors”. 24. “Expanded Definition of ‘Trefone’ and Classes of Couplet Cell Interactions”. 25. Note 1 “Extended Trefone Couplets”. Note 2 “Singlet Cells”. 26. “Background of Cancer Research”. [file 1742-4682-11-40-S1.zip › 3484671921331230_add1/3484671921331230_add18.doc]

**Additional File *Eighteen***

***(iv) The Concept of a-Cells and i-Cells Identified by the Presence or Absence of a Cell Membrane Receptor***

***Cell Receptors and Cell Markers***

*The purpose of this File is to justify the use the presence or absence of receptors to identify the two cell types in the CTC model.*

Immuno-identification of leucocytes and other cells by the presence or absence of a Cluster of Differentiation (CD) cell marker or other cell-surface protein is common. The presence or absence of receptors or of cell-bound (fixed) Trefones can also be used to delineate populations.

Many cells from a common source can be divided then into the ‘haves’ (+) and the ‘have-nots’(-). The CDs are examples of this, being defined, originally for leucocytes and immunity, by the presence or absence of reaction with a specific antibody. These are cell-surface proteins so they may be signal receptors, enzymes or adhesion proteins etc. There are many examples of CDs that are receptors:- CD30 = tumor necrosis factor (TNF) receptor family, member 8; CD16 = FcγRIII ( Fc receptors for IgG); CD32 = FcγRII; CD64 = FcγRI; CD 25 = IL-2 receptor; CD36 = monocyte scavenger receptor, a major oxidised LDL receptor; CD40 = a receptor for CD40L which is a surface and/or soluble protein; CD71= transferrin receptor; CD74 = migration inhibitory factor receptor**;** CD87 = urokinase receptor **(=** [uPA](http://en.wikipedia.org/wiki/Urokinase) receptor or uPAR); CD89 = IgA Fc receptor; CD91 = LRP-1 receptor; CD110 **= t**hrombopoietin receptor; CD116 **=** GM-CSF receptor; [CD117](http://en.wikipedia.org/wiki/CD117) = c-Kit receptor; CD135 = receptor for Flt3 ligand; CD154 = CD40L, a TNF superfamily member; CD195 = [RANTES](http://en.wikipedia.org/wiki/RANTES) and MIP receptor; CD 271 = p75 neurotrophin receptor; CD281 = Toll-like receptor 1; CD320 = transcobalamin receptor; CD332 = Fibroblast growth factor receptor 2; CD340 = Epidermal Growth Factor Receptor 2 (HER2)

There are many examples of the classification or division of cells based on the presence or absence of CDs, receptors and/or ligands (ICMs). Homogeneous populations of either Thy-1(-) and Thy-1(+) fibroblasts occur in the lung. Thy-1 (or CD90) is a [glycophosphatidylinositol](http://en.wikipedia.org/wiki/Glycophosphatidylinositol)-anchored surface [protein](http://en.wikipedia.org/wiki/Protein) first identified in thymocytes which are T-cell precursors in the thymus. The two populations are functionally different in that Thy-1(-) pulmonary fibroblasts can display class II MHC antigens, synthesize IL-1 and activate T lymphocytes, whereas the Thy-1(+) subset is devoid of these functions. T lymphocytes can also be divided into two distinct lineages based on a receptor, in that these cells express either αβ- or γδ-Tcell receptor complexes. There are also two subpopulations of monocytes which are distinguished by the expression of disparate levels of CD43 (a T cell counter-receptor for the macrophage adhesion receptor, sialoadhesin) - CD43-Hi and CD43-Lo.

But the presence or absence of a one CD is not a definitive classification in that not all (+) cells (nor all (-) cells) will necessarily be the same. A cell in an early part of a lineage may have many CD markers while more specialized cells may only have a few to define a single population.

Four surface proteins identify two endoglin-positive subpopulations that occur in fetal bone marrow: early B-lineage precursor cells (CD19-Hi, CD34-Hi, CD71- ,glycophorin A-) and proerythroblasts (CD19-Lo, CD34-Lo, CD71+ , glycophorin A+).

Four CDs differentiate between cortical thymocytes (CD1+, CD3Lo, CD4+, CD8+) which do not respond to TNF, and prethymocytes (CD1-, CD3-, CD4-, CD8-) for which TNF is mitogenic .

The idea of complementary couplet cells is seen with bone marrow (BM) transplantation where a subset of murine pluripotent hematopoietic stem cells with the phenotype Lin(-) Sca(+) kit(+) CD38(+) CD34(-) required the assistance of another BM cell subset, which has the phenotype Lin(-) Sca(+) kit(+) CD38(-) CD34(+) for rapid proliferation. The two subsets differ in the presence or absence of CD34 (perhaps a receptor) and CD38 (a receptor and ectoenzyme).

Three extracellular ICMs/Trefones in culture supernates identify two subpopulations of peritoneal macrophages; the one population which induces effector Thl cells has a pattern of IL-l^Low^, IL-6^High^ , TNF-α ^Low^, while the other which recruits suppressor Ts cells has IL-l^High^, IL-6^Low^, TNF-α ^Low^ .

The complementary selection and non-selection of certain genes and consequent presence or absence of the corresponding proteins is not unusual. It occurs in X-inactivation, autosomal imprinted genes, “randomly” monoallelic autosomal genes eg. the immunoglobulins, receptor genes (odorant, T-cell, pheromone and natural killer cell receptors). Coupled cells would also have complementary selection and non-selection of certain genes. As mentioned previously, the ciliate, *Blepharisma japonicum* produce complementary mating cells, a similar reciprocal arrangement as for *S. cerevisiae*.

In summary, the typing of cells into two related couplet cells based on the presence or absence of Trefone and/or their receptors is quite a reasonable concept. The use of flow cytometry to identify these cell-surface markers is exemplified by the investigation of the possible link between CD38/CD157 and human diseases of chronic lymphocytic leukemia, myeloma and ovarian carcinoma.

**REFERENCES:-**

1.	Hagood JS, Miller PJ, Lasky JA, Tousson A, Guo B, Fuller GM, McIntosh JC: **Differential expression of platelet-derived growth factor-alpha receptor by Thy-1(-) and Thy-1(+) lung fibroblasts**. *Am J Physiol* 1999, **277**(1 Pt 1):L218-224.

2.	Phipps RP, Borrello MA, Blieden TM: **Fibroblast heterogeneity in the periodontium and other tissues**. *J Periodontal Res* 1997, **32**(1 Pt 2):159-165.

3.	Lee SY, Stadanlick J, Kappes DJ, Wiest DL: **Towards a molecular understanding of the differential signals regulating alphabeta/gammadelta T lineage choice**. *Semin Immunol* 2010, **22**(4):237-246.

4.	Ahuja V, Miller SE, Howell DN: **Identification of two subpopulations of rat monocytes expressing disparate molecular forms and quantities of CD43**. *Cell Immunol* 1995, **163**(1):59-69.

5.	Rokhlin OW, Cohen MB, Kubagawa H, Letarte M, Cooper MD: **Differential expression of endoglin on fetal and adult hematopoietic cells in human bone marrow**. *J Immunol* 1995, **154**(9):4456-4465.

6.	Munoz-Fernandez MA, Pimentel-Muinos FX, Gonzalez A, Gambon F, Alvarez-Vallina L, Fresno M: **Differential effect of tumour necrosis factor on human thymocyte subpopulations**. *Immunology* 1992, **76**(3):439-445.

7.	Zhao Y, Lin Y, Zhan Y, Yang G, Louie J, Harrison DE, Anderson WF: **Murine hematopoietic stem cell characterization and its regulation in BM transplantation**. *Blood* 2000, **96**(9):3016-3022.

8.	Krauter J, Hartl M, Hambach L, Kohlenberg A, Gunsilius E, Ganser A, Heil G: **Receptor-mediated endocytosis of CD34 on hematopoietic cells after stimulation with the monoclonal antibody anti-HPCA-1**. *J Hematother Stem Cell Res* 2001, **10**(6):863-871.

9.	Quarona V, Zaccarello G, Chillemi A, Brunetti E, Singh VK, Ferrero E, Funaro A, Horenstein AL, Malavasi F: **CD38 and CD157: A long journey from activation markers to multifunctional molecules**. *Cytometry B Clin Cytom* 2013.

10.	Marcinkiewicz J, Bryniarski K, Ptak W: **Cyclophosphamide uncovers two separate macrophage subpopulations with opposite immunogenic potential and different patterns of monokine production**. *Cytokine* 1994, **6**(5):472-477.

11.	Miyake A, Harumoto T: **Asymmetrical cell division in Blepharisma japonicum: difference between daughter cells in mating-type expression**. *Exp Cell Res* 1990, **190**(1):65-68.

12.	Herskowitz I: **Life cycle of the budding yeast Saccharomyces cerevisiae**. *Microbiol Rev* 1988, **52**(4):536-553.
