# Supplementary material for: A model to explain specific cellular communications and cellular harmony:- a hypothesis of coupled cells and interactive coupling molecules
Source: Theor Biol Med Model. 2014 Sep 14;11:40. doi: 10.1186/1742-4682-11-40 (PMC4237941; doi:10.1186/1742-4682-11-40)
Supplement: Additional file 1 — 1. “Terminology”. 2. “Change in Cellular Characteristics”. 3. Note 1 “The Roles of SCD versus ACD”. Note 2. “Categories of SCD and ACD”. Note 3 “Further Examples of these AsCD Categories”. Note 4 “Change of differentiation type.”. 4. “The Effects of Growth Factors and other Bioactive Molecules 5. Note 1, “Additional Information on the Definition of a Trefone”. Note 2 “Clarification on Ligand and Binding Protein Interaction”. Note 3 “Cell Couplets – AsCD and the a-Cell and i-Cell”. Note 4 “Drosophila – Potential Trefones and ACD”. 6. “Calculated Levels of Trefones and TCC, with Cellular Actions”. 7. “Receptors for the Six IGFBPs and Cellular Effects”. 8. Note 1 “Extracellular Binding Proteins/ Soluble Receptors”. Note 2 “Cell Receptors for Binding Proteins/Soluble Receptors”. 9. Note 1. “Further Evidence for a Receptor for TCC”. Note 2 “Problems with Cross-linking Experiments”. 10. “Evidence for Binary Receptors aside from the IGF System”. 11. “Evidence for Two Receptors for a Particular Trefone, outside the IGF System”. 12. Note 1 “Mechanisms of Nuclear Localization”. Note 2 “NL of IGFBPs”. Note 3 “NL of Potential Trefones other than the IGF System”. 13. “Evidence Supporting the Existence of a-Cells and i-Cells”. 14. “Candidates with mainly a-Cell-Type or i-Cell-Type Characteristics”. 15. “Interacting Cells and Trefones not associated with the IGF System”. 16. “Heterogeneity/Variability of Cells in Culture.” 17. “Cell lines are not Typical of Normal Cells”. 18. “Cell Receptors and Cell Markers”. 19. “Defined Couplet Cells for Insulin and Glucagon; Gastrin and Histamine”. 20. “Potential Couplet Trefones”. 21. “Further Examples of Potential Trefone and Cell Couplets”. 22. “Examples of Cellular Regulation by Complexes”. 23. “Proteolytic enzymes and their inhibitors”. 24. “Expanded Definition of ‘Trefone’ and Classes of Couplet Cell Interactions”. 25. Note 1 “Extended Trefone Couplets”. Note 2 “Singlet Cells”. 26. “Background of Cancer Research”. [file 1742-4682-11-40-S1.zip › 3484671921331230_add1/3484671921331230_add19.doc]

**Additional File *Nineteen***

**(f) Evidence for Couplet Trefones and/or Couplet Cells**

***(i) Examples of Defined Couplet Trefones with Known Couplet Cells***

***Defined Couplet Cells for (I) Insulin and Glucagon and (II) Gastrin and Histamine***

*The purpose of this File is to justify the CTC model with concrete examples of probable cell couplets and Trefones outside the IGF system and not involving binding proteins.*

For known couplet cells (a-Cells and i-Cells) with a Trefone couplet (^a^T and ^i^T), the following is expected

(1) ^a^T binds ^i^T

(2) (i)	a-Cells have receptors for ^i^T

(ii)	^i^T normally stimulates proliferation of a-Cells

(iii) ^i^T inhibits proliferation of a-Cells when both [^i^T] and [^a^T] are high

(iv) ^i^T stimulates production/secretion of ^a^T by a-Cells

(3) (i)	i-Cells have receptors for ^a^T

(ii)	^a^T normally stimulates proliferation of i-Cells

(iii) ^a^T inhibits proliferation of i-Cells when both [^a^T] and [^i^T] are high

(iv) ^a^T stimulates production/secretion of ^i^T by i-Cells

(4) Each cell has a receptor for the ^a^T: ^i^T complex ie. the TCC .

These expectations are herein matched with observations for (I) Insulin and Glucagon produced by beta and alpha pancreatic cells, and (II) Gastrin and Histamine produced by G-cells and ECL cells, each respectively.

**(I) Insulin and Glucagon**

***The pancreas controls insulin and glucagon levels by secretion from the beta and alpha cells, respectively.*** Hyperglycemia is due to decreased glucose uptake, mainly controlled by insulin, and/or to increased glucose production, mainly controlled by glucagon, with contributions to control by epinephrine, cortisol etc. In a fasted person, the ratio of insulin to glucagon is likely to be stable but will vary depending on food intake being highest with excess exogenous carbohydrate and lowest in starvation. This likely reflects an increase of insulin in the former and an increase of glucagon in the latter.

***Insulin and glucagon could be Trefone couplets with the beta and alpha cells being the cell couplets. Arbitrarily, the beta cell is denoted the a-Cell which produces insulin (^a^T) and the alpha cell is the i-Cell which produces glucagon (***^i^T***).***

***The evidence:-***

***1. Insulin (*^a^T*) binds glucagon (* ^i^T*)***

This binding occurs but with apparent low affinity, with a Kd of 0.9 x 10^-6^, so it is said to have little significance physiologically importance. However, this low binding could be enhanced in vivo by a metal ion. Zinc, which is known to bind insulin, is known to affect binary complex formation with a dramatic (8000-fold) increase in binding affinity, known to be important in control of glucagon secretion and known to have a enhanced secretion (6.5 fold) from beta cells when exposed to glucose) could produce the required enhancement. The formation of a ternary complex of zinc:insulin:glucagon could occur within the islets between cells as the blood flows from beta to alpha cells. The interaction of glucagon and insulin with formation of the complex will also be more relevant because of the higher local concentrations of the Trefones around the islets; the concentration may be 100 times that of plasma levels which are 10-50 pmol/L.

***2. (i) Beta cells have receptors for glucagon***

Isolated rat pancreatic beta-cells have specific glucagon receptors as do a rat insulinoma cell line (INS-1) and a mouse insulinoma beta cell line (MIN6), while another tumour-derived beta-cell line (beta TC-3) does not express functional glucagon receptors

***(ii) Glucagon stimulates proliferation of beta cells***

Overexpression of the glucagon receptor in pancreatic beta-cells increased both insulin content and β-cell mass (while not affecting islet cell organization or architecture).

***(iii) Glucagon inhibits proliferation of beta cells when [Glucagon] & [Insulin] are high***

There is no information available on this.

***(iv) Glucagon stimulates* *production/secretion* of *insulin by beta cells***

Stimulation of insulin release by glucagon was observed in experiments on human subjects nearly 50 years ago - glucagon’s insulinogenic effect. Stimulation has also been shown to occur in isolated perfused rat pancreas, where concentrations of glucagon as low as 10^-12^ mol/L resulted in significant insulin release, and in cultured cloned pancreatic B cells (HIT-T15 and RINm5F) .

***3. (i) Alpha cells have insulin receptors***

The mRNA for the insulin receptor is expressed on clonal pancreatic alpha cells **(**glucagon-secreting cell lines:- In-R1-G9 from an insulinoma, and alpha TC, clone 6 cells from an adenoma) and labelled insulin is bound to receptors. Electron microscopy has detected insulin on alpha cells in isolated rat islets..

***(ii). Insulin stimulates production/secretion of glucagon by alpha cells***

At higher doses, perfused insulin has been shown to stimulate glucagon secretion in whole rats. But other evidence from a cell line (a clonal hamster islet alpha cell line, In-R1-G9) indicates that insulin decreases steady-state glucagon and insulin is widely considered to be a physiological suppressor of glucagon secretion although its mode of action is not clear To the contrary, it seems that the insulin receptor is required in alpha-cells for glucagon secretion induced by glucose because siRNA-mediated “knockdown” of the insulin receptor in a pancreatic alpha-beta-cell line (alpha-TC6), abolishes this glucagon secretion. Some authors question the validity of the extrapolation of evidence from cell cultures (eg. In-R1-G9 above) to the islet micro-organ which is complex hormonally and in cellular organization.

***(iii) Insulin inhibits proliferation of alpha cells when[Insulin] &[Glucagon] are high***

There is no information available on this.

***(iv) Insulin stimulates proliferation of alpha cells***

Insulin produced an up-regulation of α-cell proliferation in αTC1-6 cells (a mouse pancreatic α-cell line, a subclone of a glucagonoma-derived cell line), and, in diabetic mice, both α-cell number and plasma glucagon levels increased as diabetes progressed.

***4. The alpha and beta cells would each have a receptor for the insulin:glucagon complex.***

There is no information available on this. It is possible that one of the isoforms (IR-A or IR-B) of the insulin receptor could bind the insulin:glucagon complex (TCC) or perhaps the insulin receptor-related receptor (IRR) could, as it is known to be in pancreatic islets and likely in beta cells. Beta cells also contain two glucagon receptors – one high-, one low-affinity for glucagon. Perhaps one could bind the glucagon:insulin complex (TCC).

***5. Pulsatile Secretions***

## The release of glucagon and insulin from human pancreatic islets is pulsatile. The kinetics of insulin and glucagon (and somatostatin) releases were studied in batches of 10-15 islets perfused with glucose. An increase of glucose from 3 to 20 mmol/L resulted in a brief pulse of glucagon at one minute, followed by a pulse of insulin. Glucose continued to generate pulsatile releases of the hormones with 7- to 8-min periods but the repetitive glucagon pulses were antisynchronous to pulses of insulin. This could be interpreted in the current model as indicating that the first release of glucagon stimulates insulin, then insulin stimulates glucagon, and so on, where the respective receptors on the beta and alpha cells are momentarily exposed to hormones from the spiked transcellular fluid before this interstitial fluid is whisked away by perfusion fluid. In reality, plasma would contribute to the extracellular fluid and blood flow would rapidly remove the fluid containing the oscillating Trefone concentrations. In this case, the cytoarchitecture of the perfused islets would need to allow some contact with alpha cells before contact with beta cells. Alpha cells are mainly on the periphery but scattered among the beta-cells in human islets.

## 6. General

Insulin is required for metabolic reasons in most/all cells and its presence is expected inside beta cells and insulin receptors have been detected on beta cell lines RINm5F (an insulin-producing rat pancreatic cell line) and B-TC3 (an insulin-secreting beta-cell, insulinoma line). While this evidence is from cell lines, it would be expected that as beta (and alpha) islet cells utilize glucose for energy purposes, they too would possess insulin receptors. However, within beta cells, insulin endocytosed by insulin receptors will not act as a Trefone to affect cell-life decisions outside of energetic considerations. Only in alpha cells will there be an effect of insulin on proliferation via these insulin receptors, just as beta cells need glucagon receptors to control proliferation. Insulin receptor is more abundant in alpha-cells compared to beta-cells as would be expected with a dual function in alpha cells.

In total then, glucagon will stimulate beta cells to produce INS only if nutrients (glucose, fatty acids and/or certain amino acids) and consequently ATP in the cell are high. ATP may be responsible for so-called “nutrient signalling”. Insulin will stimulate alpha cell to produce glucagon only if nutrients (glucose, fatty acids and/or certain amino acids) and consequently ATP in the cell are low. The effect of nutrients is controlled by MTOR **(**Mammalian target of rapamycin) which is a protein kinase that integrates signals from mitogens and nutrients to regulate cellular growth and proliferation. The insulin-stimulated α-cell proliferation acts through the mTOR signalling pathway.

The model offered here is simplified in that a variety of other hormones/growth factors and neurotransmitters are not considered here but they do affect the release of insulin and glucagon by stimulation or inhibition of beta and alpha cellular activity. Somatostatin, glucagon-like peptide and epinephrine are included in the list.

The CTC model would require an asymmetric cell division in the embryonic development of the alpha and beta cells of the pancreas.. A study of neogenesis of alpha and beta cells should indicate coexpression of glucagon and insulin prior to asymmetric cell division(s) to produce the individual alpha and beta cells. The early progenitor cells may produce just one of the Trefones before stimulation perhaps to dual production prior to the AsCD that forms the beta and alpha cells. Post AsCD, the protein expression of each may depend on the immediate cellular environment such that gene activation may have occurred but without translation.

Information from microarray analysis could possibly be zippered into the proposed CTC model although the prevailing model has AsCD producing the alpha cell with a couplet beta/delta cell which subsequently produces the individual beta and delta cells. This would mean that these two cells would both produce insulin and they would be coupled by their own couplet Trefones – perhaps somatostatin and amylin. As delta cells also produce glucagon, a complex interactive grouping of cells could evolve in the pancreas.

Other ICMs may stimulate beta cell growth. For example, the incretin glucagon-like peptide-1 enhances beta-cell neogenesis, but is not here regarded as a Trefone.

(Instead of an insulin/glucagon Trefone couplet, an alternate couplet could exist within the beta cells if couplet beta cells existed. Of the beta cells from a rat pancreas, about 50% are highly responsive and release insulin at low glucose values while the rest respond only at higher glucose levels. In mouse pancreatic beta-cells induced by glucose, about 50% of the cells produced a smooth increase insulin release, but the others produced a rough spiky release of insulin. Perhaps there are two beta cell types so that a-Cells and i-Cells coexist within the islets; glucose and insulin could be Trefones and glycated insulin the complex ie. the TCC)

**7. Conclusion**

The deficiency in insulin activity in diabetes may be due to insulin insensitivity in the presence of adequate insulin or due to insulin deficiency. Where there is a deficiency, this may be due to the rate of apoptosis surpassing the rate of proliferation from SCD of existing cells and from neogenesis from stem cells. In diabetes, the rate of apoptosis exceeds the rate of proliferation and neogenesis all of which are higher than normal. It is then the balance and control of apoptosis versus proliferation that is critical to maintaining normal cell mass and insulin production. It is then a balance between the Trefones (insulin and glucagon) that controls metabolic imbalance leading to diabetes.

**(II) Gastrin and Histamine**

**Gastrin (GAS)** is a hormone that is secreted from G-cells which are mainly in the antrum of the [stomach](http://en.wikipedia.org/wiki/Stomach) and the duodenum, and it stimulates acid secretion by the parietal cells.

The gastrin receptor is one of the two cholecystokinin receptors and it is the cholecystokinin B receptor (CCK_2_). It is a [G-protein coupled receptor and is also found in the CNS.](http://en.wikipedia.org/wiki/G-protein_coupled_receptor)

The CTC model requires that a Trefone will be a proliferator and a secretagogue. Gastrin is both a potent gastrointestinal trophic agent acting as a growth factor to induce cell proliferation and a histamine secretory agent and.

**Histamine (HTM)** acts as a hormone and [neurotransmitter](https://en.wikipedia.org/wiki/Neurotransmitter). It is produced by enterochromaffin-like (ECL) cells and exerts hormonal control of the acidity of the stomach. It is also produced by [mast cells](https://en.wikipedia.org/wiki/Mast_cell) and [basophil](https://en.wikipedia.org/wiki/Basophil)s and triggers an [inflammatory response](https://en.wikipedia.org/wiki/Inflammatory_response) to foreign [pathogens](https://en.wikipedia.org/wiki/Pathogens).

There are four specific types of histamine receptors and they are 7-transmembrane G-protein coupled receptors. The H_2_ receptor functions to stimulate gastric acid production by parietal cells.

***Gastrin and histamine could be Trefone couplets with the G-Cell and enterochromaffin-like (ECL) cell being the cell couplets. Arbitrarily, the G-cell is the a-Cell which produces gastrin*** ***(^a^T) and the ECL cell is the i-Cell which produces histamine ( ^i^T).***

***The evidence:-***

***(1) Gastrin (^a^T) binds Histamine ( ^i^T)***

There is no evidence for this (known to be recorded). Were studies to be conducted to assess this, then the fact that glycine-extended gastrin-17 binds ferric ions at low pH and it is this form which is biologically active as a stimulant to proliferation of colonic mucosa, may need to be considered.

Further, to be fully investigated, all of the five active forms of Gastrin would need to be considered to discover the actual Trefone. Progastrin, the C-terminal Gly-extended gastrins (G34-Gly and G17-Gly) and the C-terminal amidated gastrins (G34 and G17), all have some biological activity.

***(2) (i) G-Cells have receptors for Histamine (HTM)***

Rabbit G-cells have histamine H2-receptors

***(ii)/(iii)HTM stimulates/inhibits proliferation of G-Cells***

While there is no proliferation study (known to be recorded) of G-cells involving histamine, it is known to be a proliferative stimulant for some other cells (eg. airway smooth muscle cells, cultured rat thymic epithelium cells, Leydig cancer cell line ) and an inhibitor for others (eg. colorectal, pancreatic carcinoma cells).

***(iv) HTM stimulates production of Gastrin (GAS) by G-Cells***

Histamine stimulates release of gastrin from G-cells via H2-receptors.

***(3) (i) ECL cells have receptors for GAS***

ECL cells have receptors for gastrin, and mRNA for the cholecystokinin B/gastrin receptor is present in enterochromaffin-like cells of the human stomach.

***(ii) GAS stimulates proliferation of ECL-Cells***

GAS induces ECL cell proliferation in cell culture and it has a specific proliferative effect on rat ECL cells. Amidated gastrin causes increased proliferation of ECL cells in the oxyntic mucosa of the stomach of mice.

***(iii) GAS inhibits proliferation of ECL-cells when [GAS] and (HTM] are high***

GAS inhibition of ECL-cells is not recorded but GAS does inhibit proliferation of colon cancer cells.

***(iv) GAS stimulates production of HTM by ECL-Cells***

Gastrin stimulates the release of histamine from gastric ECL cells in cell culture and from rabbit fundic mucosal cells enriched in ECL cells.

***(4) The G-cells and ECL cells would each have a receptor for the gastrin:histamine***

***complex.***

Gastrin has four cell receptors with variable binding to gastrin variants and one could possibly bind a gastrin:histamine complex (TCC). Histamine has two (H1 and H2) receptors which could possibly bind a histamine:gastrin complex (TCC).

**REFERENCES:-**

1.	Root-Bernstein RS, Dobbelstein C: **Insulin binds to glucagon forming a complex that is hyper-antigenic and inducing complementary antibodies having an idiotype-antiidiotype relationship**. *Autoimmunity* 2001, **33**(3):153-169.

2.	Cunningham BC, Bass S, Fuh G, Wells JA: **Zinc mediation of the binding of human growth hormone to the human prolactin receptor**. *Science* 1990, **250**(4988):1709-1712.

3.	Gromada J, Franklin I, Wollheim CB: **Alpha-cells of the endocrine pancreas: 35 years of research but the enigma remains**. *Endocr Rev* 2007, **28**(1):84-116.

4.	Bonner-Weir S, Orci L: **New perspectives on the microvasculature of the islets of Langerhans in the rat**. *Diabetes* 1982, **31**(10):883-889.

5.	Moens K, Heimberg H, Flamez D, Huypens P, Quartier E, Ling Z, Pipeleers D, Gremlich S, Thorens B, Schuit F: **Expression and functional activity of glucagon, glucagon-like peptide I, and glucose-dependent insulinotropic peptide receptors in rat pancreatic islet cells**. *Diabetes* 1996, **45**(2):257-261.

6.	Kieffer TJ, Heller RS, Unson CG, Weir GC, Habener JF: **Distribution of glucagon receptors on hormone-specific endocrine cells of rat pancreatic islets**. *Endocrinology* 1996, **137**(11):5119-5125.

7.	Yamato E, Ikegami H, Takekawa K, Fujisawa T, Nakagawa Y, Hamada Y, Ueda H, Ogihara T: **Tissue-specific and glucose-dependent expression of receptor genes for glucagon and glucagon-like peptide-1 (GLP-1)**. *Horm Metab Res* 1997, **29**(2):56-59.

8.	Huypens P, Ling Z, Pipeleers D, Schuit F: **Glucagon receptors on human islet cells contribute to glucose competence of insulin release**. *Diabetologia* 2000, **43**(8):1012-1019.

9.	Gelling RW, Vuguin PM, Du XQ, Cui L, Romer J, Pederson RA, Leiser M, Sorensen H, Holst JJ, Fledelius C *et al*: **Pancreatic beta-cell overexpression of the glucagon receptor gene results in enhanced beta-cell function and mass**. *Am J Physiol Endocrinol Metab* 2009, **297**(3):E695-707.

10.	Samols E, Marri G, Marks V: **Interrelationship of glucagon, insulin and glucose. The insulinogenic effect of glucagon**. *Diabetes* 1966, **15**(12):855-866.

11.	Ahren B, Nobin A, Schersten B: **Insulin and C-peptide secretory responses to glucagon in man: studies on the dose-response relationships**. *Acta Med Scand* 1987, **221**(2):185-190.

12.	Kawai K, Yokota C, Ohashi S, Watanabe Y, Yamashita K: **Evidence that glucagon stimulates insulin secretion through its own receptor in rats**. *Diabetologia* 1995, **38**(3):274-276.

13.	Lambert DG, Atkins TW: **Effects of islet hormones on insulin secretion from cloned B cell lines, HIT-T15 and RINm5F**. *J Endocrinol* 1989, **121**(3):479-485.

14.	Kisanuki K, Kishikawa H, Araki E, Shirotani T, Uehara M, Isami S, Ura S, Jinnouchi H, Miyamura N, Shichiri M: **Expression of insulin receptor on clonal pancreatic alpha cells and its possible role for insulin-stimulated negative regulation of glucagon secretion**. *Diabetologia* 1995, **38**(4):422-429.

15.	Patel YC, Amherdt M, Orci L: **Quantitative electron microscopic autoradiography of insulin, glucagon, and somatostatin binding sites on islets**. *Science* 1982, **217**(4565):1155-1156.

16.	Dunbar JC, Walsh MF, Foa PP: **The serum glucose response to glucagon suppression with somatostatin, insulin or antiglucagon serum in depancreatized rats**. *Diabetologia* 1978, **14**(1):53-58.

17.	Philippe J: **Glucagon gene transcription is negatively regulated by insulin in a hamster islet cell line**. *J Clin Invest* 1989, **84**(2):672-677.

18.	Kaneko K, Shirotani T, Araki E, Matsumoto K, Taguchi T, Motoshima H, Yoshizato K, Kishikawa H, Shichiri M: **Insulin inhibits glucagon secretion by the activation of PI3-kinase in In-R1-G9 cells**. *Diabetes Res Clin Pract* 1999, **44**(2):83-92.

19.	Bansal P, Wang Q: **Insulin as a physiological modulator of glucagon secretion**. *Am J Physiol Endocrinol Metab* 2008, **295**(4):E751-761.

20.	Diao J, Asghar Z, Chan CB, Wheeler MB: **Glucose-regulated glucagon secretion requires insulin receptor expression in pancreatic alpha-cells**. *J Biol Chem* 2005, **280**(39):33487-33496.

21.	Samols E, Bonner-Weir S, Weir GC: **Intra-islet insulin-glucagon-somatostatin relationships**. *Clin Endocrinol Metab* 1986, **15**(1):33-58.

22.	Liu Z, Kim W, Chen Z, Shin YK, Carlson OD, Fiori JL, Xin L, Napora JK, Short R, Odetunde JO *et al*: **Insulin and glucagon regulate pancreatic alpha-cell proliferation**. *PLoS One* 2011, **6**(1):e16096.

23.	Ozaki K: **Insulin receptor-related receptor in rat islets of Langerhans**. *Eur J Endocrinol* 1998, **139**(2):244-247.

24.	Moens K, Flamez D, Van Schravendijk C, Ling Z, Pipeleers D, Schuit F: **Dual glucagon recognition by pancreatic beta-cells via glucagon and glucagon-like peptide 1 receptors**. *Diabetes* 1998, **47**(1):66-72.

25.	Hellman B, Salehi A, Gylfe E, Dansk H, Grapengiesser E: **Glucose generates coincident insulin and somatostatin pulses and antisynchronous glucagon pulses from human pancreatic islets**. *Endocrinology* 2009, **150**(12):5334-5340.

26.	Bonner-Weir S, O'Brien TD: **Islets in type 2 diabetes: in honor of Dr. Robert C. Turner**. *Diabetes* 2008, **57**(11):2899-2904.

27.	Gazzano H, Halban P, Prentki M, Ballotti R, Brandenburg D, Fehlmann M, Van Obberghen E: **Identification of functional insulin receptors on membranes from an insulin-producing cell line (RINm5F)**. *Biochem J* 1985, **226**(3):867-872.

28.	Rothenberg PL, Willison LD, Simon J, Wolf BA: **Glucose-induced insulin receptor tyrosine phosphorylation in insulin-secreting beta-cells**. *Diabetes* 1995, **44**(7):802-809.

29.	Franklin I, Gromada J, Gjinovci A, Theander S, Wollheim CB: **Beta-cell secretory products activate alpha-cell ATP-dependent potassium channels to inhibit glucagon release**. *Diabetes* 2005, **54**(6):1808-1815.

30.	McDaniel ML, Marshall CA, Pappan KL, Kwon G: **Metabolic and autocrine regulation of the mammalian target of rapamycin by pancreatic beta-cells**. *Diabetes* 2002, **51**(10):2877-2885.

31.	Kwon G, Marshall CA, Pappan KL, Remedi MS, McDaniel ML: **Signaling elements involved in the metabolic regulation of mTOR by nutrients, incretins, and growth factors in islets**. *Diabetes* 2004, **53 Suppl 3**:S225-232.

32.	Nielsen JH, Galsgaard ED, Moldrup A, Friedrichsen BN, Billestrup N, Hansen JA, Lee YC, Carlsson C: **Regulation of beta-cell mass by hormones and growth factors**. *Diabetes* 2001, **50 Suppl 1**:S25-29.

33.	Teitelman G, Alpert S, Polak JM, Martinez A, Hanahan D: **Precursor cells of mouse endocrine pancreas coexpress insulin, glucagon and the neuronal proteins tyrosine hydroxylase and neuropeptide Y, but not pancreatic polypeptide**. *Development Supplement* 1993, **118**(4):1031-1039.

34.	Chiang MK, Melton DA: **Single-cell transcript analysis of pancreas development**. *Dev Cell* 2003, **4**(3):383-393.

35.	Van Schravendijk CF, Kiekens R, Pipeleers DG: **Pancreatic beta cell heterogeneity in glucose-induced insulin secretion**. *J Biol Chem* 1992, **267**(30):21344-21348.

36.	Bokvist K, Holmqvist M, Gromada J, Rorsman P: **Compound exocytosis in voltage-clamped mouse pancreatic beta-cells revealed by carbon fibre amperometry**. *Pflugers Arch* 2000, **439**(5):634-645.

37.	Rhodes CJ: **Type 2 diabetes-a matter of beta-cell life and death?** *Science* 2005, **307**(5708):380-384.

38.	Ackermann AM, Gannon M: **Molecular regulation of pancreatic beta-cell mass development, maintenance, and expansion**. *J Mol Endocrinol* 2007, **38**(1-2):193-206.

39.	Kidd M, Modlin IM, Tang LH: **Gastrin and the enterochromaffin-like cell: an acid update**. *Dig Surg* 1998, **15**(3):209-217.

40.	Pannequin J, Barnham KJ, Hollande F, Shulkes A, Norton RS, Baldwin GS: **Ferric ions are essential for the biological activity of the hormone glycine-extended gastrin**. *J Biol Chem* 2002, **277**(50):48602-48609.

41.	Dockray GJ, Varro A, Dimaline R, Wang T: **The gastrins: their production and biological activities**. *Annu Rev Physiol* 2001, **63**:119-139.

42.	Bliss PW, Healey ZV, Arebi N, Calam J: **Nalpha-methyl histamine and histamine stimulate gastrin release from rabbit G-cells via histamine H2-receptors**. *Aliment Pharmacol Ther* 1999, **13**(12):1669-1674.

43.	Panettieri RA, Yadvish PA, Kelly AM, Rubinstein NA, Kotlikoff MI: **Histamine stimulates proliferation of airway smooth muscle and induces c-fos expression**. *Am J Physiol* 1990, **259**(6 Pt 1):L365-371.

44.	Head GM, Mentlein R, von Patay B, Downing JE, Kendall MD: **Neuropeptides exert direct effects on rat thymic epithelial cells in culture**. *Dev Immunol* 1998, **6**(1-2):95-104.

45.	Pagotto RM, Monzon C, Moreno MB, Pignataro OP, Mondillo C: **Proliferative effect of histamine on MA-10 Leydig tumor cells mediated through HRH2 activation, transient elevation in cAMP production, and increased extracellular signal-regulated kinase phosphorylation levels**. *Biol Reprod* 2012, **87**(6):150.

46.	Suonio E, Tuomisto L, Alhava E: **Effects of histamine, H1, H2 and Hic receptor antagonists and alpha-fluoromethylhistidine on the growth of human colorectal cancer in the subrenal capsule assay**. *Agents Actions* 1994, **41 Spec No**:C118-120.

47.	Cricco G, Martin G, Labombarda F, Cocca C, Bergoc R, Rivera E: **Human pancreatic carcinoma cell line Panc-I and the role of histamine in growth regulation**. *Inflamm Res* 2000, **49 Suppl 1**:S68-69.

48.	Modlin IM, Tang LH: **The gastric enterochromaffin-like cell: an enigmatic cellular link**. *Gastroenterology* 1996, **111**(3):783-810.

49.	Kulaksiz H, Arnold R, Goke B, Maronde E, Meyer M, Fahrenholz F, Forssmann WG, Eissele R: **Expression and cell-specific localization of the cholecystokinin B/gastrin receptor in the human stomach**. *Cell Tissue Res* 2000, **299**(2):289-298.

50.	Prinz C, Scott DR, Hurwitz D, Helander HF, Sachs G: **Gastrin effects on isolated rat enterochromaffin-like cells in primary culture**. *Am J Physiol* 1994, **267**(4 Pt 1):G663-675.

51.	Bakke I, Qvigstad G, Brenna E, Sandvik AK, Waldum HL: **Gastrin has a specific proliferative effect on the rat enterochromaffin-like cell, but not on the parietal cell: a study by elutriation centrifugation**. *Acta Physiol Scand* 2000, **169**(1):29-37.

52.	Koh TJ, Chen D: **Gastrin as a growth factor in the gastrointestinal tract**. *Regul Pept* 2000, **93**(1-3):37-44.

53.	Song LJ, Liu RJ, Zeng Z, Alper SL, Cui HJ, Lu Y, Zheng L, Yan ZW, Fu GH: **Gastrin inhibits a novel, pathological colon cancer signaling pathway involving EGR1, AE2, and P-ERK**. *J Mol Med (Berl)* 2012, **90**(6):707-718.

54.	Hollande F, Combettes S, Bali JP, Magous R: **Gastrin stimulation of histamine synthesis in enterochromaffin-like cells from rabbit fundic mucosa**. *Am J Physiol* 1996, **270**(3 Pt 1):G463-469.

55.	Yassin RR: **Signaling pathways mediating gastrin's growth-promoting effects**. *Peptides* 1999, **20**(7):885-898.

56.	McIntosh CH, Bakich V, Kwok YN, Wong J, Brown JC: **The effects of substance P, histamine and histamine antagonists on somatostatin and gastrin release from the isolated perfused rat stomach**. *Regul Pept* 1987, **19**(3-4):253-263.
