# Supplementary material for: A model to explain specific cellular communications and cellular harmony:- a hypothesis of coupled cells and interactive coupling molecules
Source: Theor Biol Med Model. 2014 Sep 14;11:40. doi: 10.1186/1742-4682-11-40 (PMC4237941; doi:10.1186/1742-4682-11-40)
Supplement: Additional file 1 — 1. “Terminology”. 2. “Change in Cellular Characteristics”. 3. Note 1 “The Roles of SCD versus ACD”. Note 2. “Categories of SCD and ACD”. Note 3 “Further Examples of these AsCD Categories”. Note 4 “Change of differentiation type.”. 4. “The Effects of Growth Factors and other Bioactive Molecules 5. Note 1, “Additional Information on the Definition of a Trefone”. Note 2 “Clarification on Ligand and Binding Protein Interaction”. Note 3 “Cell Couplets – AsCD and the a-Cell and i-Cell”. Note 4 “Drosophila – Potential Trefones and ACD”. 6. “Calculated Levels of Trefones and TCC, with Cellular Actions”. 7. “Receptors for the Six IGFBPs and Cellular Effects”. 8. Note 1 “Extracellular Binding Proteins/ Soluble Receptors”. Note 2 “Cell Receptors for Binding Proteins/Soluble Receptors”. 9. Note 1. “Further Evidence for a Receptor for TCC”. Note 2 “Problems with Cross-linking Experiments”. 10. “Evidence for Binary Receptors aside from the IGF System”. 11. “Evidence for Two Receptors for a Particular Trefone, outside the IGF System”. 12. Note 1 “Mechanisms of Nuclear Localization”. Note 2 “NL of IGFBPs”. Note 3 “NL of Potential Trefones other than the IGF System”. 13. “Evidence Supporting the Existence of a-Cells and i-Cells”. 14. “Candidates with mainly a-Cell-Type or i-Cell-Type Characteristics”. 15. “Interacting Cells and Trefones not associated with the IGF System”. 16. “Heterogeneity/Variability of Cells in Culture.” 17. “Cell lines are not Typical of Normal Cells”. 18. “Cell Receptors and Cell Markers”. 19. “Defined Couplet Cells for Insulin and Glucagon; Gastrin and Histamine”. 20. “Potential Couplet Trefones”. 21. “Further Examples of Potential Trefone and Cell Couplets”. 22. “Examples of Cellular Regulation by Complexes”. 23. “Proteolytic enzymes and their inhibitors”. 24. “Expanded Definition of ‘Trefone’ and Classes of Couplet Cell Interactions”. 25. Note 1 “Extended Trefone Couplets”. Note 2 “Singlet Cells”. 26. “Background of Cancer Research”. [file 1742-4682-11-40-S1.zip › 3484671921331230_add1/3484671921331230_add20.doc]

**Additional File** ***Twenty***

**(f) Evidence for Couplet Trefones and/or Couplet Cells**

***(ii) Examples of Couplet Trefones (not Binding Proteins) with Potential Couplet Trefones but Unknown Couplet Cells***

*The purpose of this File is to justify the CTC model with examples of other possible Trefones outside the IGF system and not involving binding proteins*

The evidence presented here focuses on ICMs that may be Trefone Couplets without knowledge of possible Cell Couplets. The evidence is then from a myriad of (sometimes unidentified) cells in order to detect or confirm the expected functional properties of Trefones.

The evidence for the first three potential couplets is presented under the following seven headings:-

*a. The two couplets bind to form a stable complex;*

*b. Both are known to stimulate and inhibit proliferation;*

*c. Each stimulates the other’s production;*

*d. A cell that produces one Trefone has the receptor for the other;*

*e. There is co-localization of one Trefone and the receptor for the other;*

*f. The Trefones have intracellular effects or at least are internalized;*

*g.* *A receptor for the TCC is present on certain cells.*

**(1) Dopamine and Neurotensin**

**Dopamine** functions as a [neurotransmitter](http://en.wikipedia.org/wiki/Neurotransmitter) in the [brain](http://en.wikipedia.org/wiki/Brain) but outside the [central nervous system](http://en.wikipedia.org/wiki/Central_nervous_system)(CNS) dopamine acts as a [hormone](http://en.wikipedia.org/wiki/Hormone), produced by the [adrenal medulla, an organ of neural origin.](http://en.wikipedia.org/wiki/Adrenal_gland) As a hormone, it affects the kidneys, heart, immune system and the [sympathetic nervous system](http://en.wikipedia.org/wiki/Sympathetic_nervous_system). The focus here is on proliferation of cells rather than on the synaptic actions of dopamine. Given that there are five dopamine receptor types plus variants, one or more of these G protein coupled receptors may be associated with the proliferative effects of dopamine in a cell-specific way. Each may have a specific function in specific cells as illustrated by the different effects of D2 and D3 receptors in controlling neurotensin mRNA expression in rat nucleus accumbens, negatively and positively, respectively.

**Neurotensin** is a [neuropeptide](http://en.wikipedia.org/wiki/Neuropeptide) hormone found throughout the CNS, especially in the [hypothalamus](http://en.wikipedia.org/wiki/Hypothalamus), and in [endocrine](http://en.wikipedia.org/wiki/Endocrine) [cells](http://en.wikipedia.org/wiki/Cell_(biology)) of the small intestine where it is associated with colorectal cancer. It has interaction with the [dopaminergic system](http://en.wikipedia.org/wiki/Dopaminergic_system) and [regulates](http://en.wikipedia.org/wiki/Regulation) dopamine pathways. Neurotensin has two receptors which contain seven-transmembrane helices and are [G-protein coupled](http://en.wikipedia.org/wiki/G_protein-coupled_receptor).

**Evidence:-**

***a. The two couplets bind to form a stable complex***

Dopamine (DA) binds neurotensin (NT) and this is an essential requirement for couplet Trefones**.**

***b. Both are known to stimulate and inhibit proliferation***

DA stimulates the growth of chinese hamster ovary cell but inhibits the growth of cultures from rat pituitary glands. NT stimulates some astrocytic tumour cell lines and lung cancer cells but inhibits cutaneous T cell lymphoma cell growth.

***c. Each stimulates the other’s production***.

DA stimulates NT production in rat hypothalamic tissue fragments and dopamine D3 receptor antisense reduces neurotensin mRNA levels in rat nucleus accumbens. NT stimulates DA production in rat striatal slices**,** rat posteromedial nucleus accumbens and in mouse neostriatal slices.

***d. A cell that produces one Trefone has the receptor for the other***

Dopaminergic neurons from coronal slices of guinea-pig brain have neurotensin receptors and cells of the ventromedial part of the shell subdivision within the nucleus accumbens, express both dopamine D3 receptor mRNA and NT mRNA.

***e. There is co-localization*** ***of one Trefone and the receptor for the other***

The protein NT and the D2 receptor for DA colocalize in aspiny neurons and dendrites in rat nucleus accumbens while the vast majority of DA neurons in the midbrain nuclei express NT receptors predominantly of the NT1-subtype.

***f. The Trefones have intracellular effects or at least are internalized***

Stimulation of D_2_ dopamine receptors activated the ERK (extracellular signal-regulated kinase) signalling pathway which is involved in regulation of cell proliferation, in denervated rat striatum.

Both DA and NT are internalized and internalized NT eventually moves to surround the nuclei to regulate gene expression.

***g.* *A receptor for the TCC is present on certain cells***

No information is known.

**(2) Serotonin** {5-hydroxytryptamine (5-HT)} **and ACTH** (Adrenocorticotropic hormone, corticotropin)

**Serotonin**, a hormone and neurotransmitter, is mostly produced by the [enterochromaffin cells](http://en.wikipedia.org/wiki/Enterochromaffin_cells) in the alimentary canal, but it is also produced by pulmonary and Leydig cells, by the pineal gland and by [serotonergic](http://en.wikipedia.org/wiki/Serotonergic) [neurons](http://en.wikipedia.org/wiki/Neuron) of the CNS. It has seven major receptor types with two of them having 5-6 subtypes, with all but one of the major type receptors containing seven-transmembrane helices and being [G-protein coupled](http://en.wikipedia.org/wiki/G_protein-coupled_receptor). Serotonin acts on these receptors located on smooth muscles, enterocytes and nerves.

**ACTH** is a [polypeptide](https://en.wikipedia.org/wiki/Peptide) hormone secreted by the [anterior pituitary gland](https://en.wikipedia.org/wiki/Anterior_pituitary) as part of the [hypothalamic-pituitary-adrenal axis](https://en.wikipedia.org/wiki/Hypothalamic-pituitary-adrenal_axis). It stimulates corticosteroid release from the adrenal cortex. The ACTH receptor has seven hydrophobic domains, perhaps transmembrane segments, and has an intracellular domain with protein kinase A and protein-kinase-C motifs present.

Although these are both hormones and one is also a neurotransmitter, it is their interaction with each other and their effect on proliferation etc which are the foci.

**Evidence:-**

***a. The two couplets bind to form a stable complex***

Serotonin binds ACTH*.*

***b. Both are known to stimulate and inhibit proliferation***

5-HT regulates cell proliferation, migration and maturation in a variety of cell types, (eg. lung, kidney, endothelial cells, mast cells, neurons and astrocytes).

# 5-HT stimulates proliferation in aortic/vascular smooth muscle, in rat renal mesangial cells **and** in murine epidermal keratinocytes in situ.

5-HT inhibits proliferation of human peripheral blood lymphocytes but the proliferation of both human and murine primary helper T cells and of human helper T cell lines is inhibited by antagonists to the serotonin receptor 5-HT(1B).

In apparent contradiction, 5-HT stimulated proliferation of melanocytes in medium without growth factors but inhibited proliferation in the presence of growth factors.

ACTH stimulates proliferation of mouse myogenic cells but not of skin fibroblasts. In apparent contradiction again, ACTH inhibits or stimulates proliferation of rat adrenocortical cells in primary culture, depending on the time of exposure to ACTH The primary effect of ACTH was inhibition in the first 24 h, followed by an intense stimulatory phase during the third day of ACTH treatment. ACTH inhibits proliferation of fetal rat adrenocortical cells.

***c. Each stimulates the other’s production***

5-HT stimulates ACTH secretion from male rats when infusing intracerebro-ventricularly and from the neurointermediate lobe of the rat pituitary gland incubated *in vitro* Lamberts . A 5-HT-releasing agent also stimulated ACTH in man and 5-HT is involved in the stimulation of ACTH secretion during stress. ACTH stimulates serotonin production as indicated by the increased content of 5-HT in the hippocampus, midbrain-pons and spinal cord of rats following intraperitoneal injection of ACTH.

***d. A cell that produces one Trefone has the receptor for the other***

Primary cultures of rat pituitary cells have the serotonin receptor 5-HT_3_. These cells release ACTH in response to stimulation by serotonin which suggests that this serotonin receptor is involved in the release of ACTH from the pituitary gland in vivo.

If enterochromaffin cells were one of the cell couplet, then an expectation would be that these cells would have an ACTH receptor. No evidence of this was found.

***e. There is co-localization of*** ***one Trefone and the receptor for the other***

No information is known.

***f. The Trefones have intracellular effects or at least are internalized***

The G-protein coupled receptors activate ERK (extracellular signal-regulated kinase) and Akt (protein kinase B), and the related signalling events, via 5-HT and via ACTH receptors.

***g.* *A receptor for the TCC is present on certain cells***

No information is known.

**(3) CGRP (**calcitonin gene-related peptide) and **IL-1** interleukin-1 beta (IL-1 beta).

CGRP, is a vasodilator and neuropeptide pain transmitter produced by peripheral and central [neurons](http://en.wikipedia.org/wiki/Neurons). It has one receptor which is a [G protein-coupled receptor](http://en.wikipedia.org/wiki/G_protein-coupled_receptor) and is present in T and B lymphocytes, thymus, bone marrow and lymphoid cell lines.

Interleukin 1 beta ([IL-1 beta](http://en.wikipedia.org/wiki/IL1B)) is a cytokine involved in the regulation of immune response and inflammatory reactions but its relevant activity here is its involvement in hematopoiesis. It has two types receptor and both exist in transmembrane and soluble forms. (The soluble receptor or binding protein could then be the couplet Trefone to Il-1beta rather than CGRP).

**Evidence:-**

***a. The two couplets bind to form a stable complex***

No information is known.

***b. Both are known to stimulate and inhibit proliferation***

CGRP has proliferative activity in osteoblast-like cells and in tracheal epithelial cells in primary culture but inhibits proliferation in T lymphocytes and in human peripheral blood mononuclear cells.

IL-1 beta. has proliferative activity in myeloid progenitor cells in human long-term bone marrow cultures but inhibits proliferation in osteoblasts .

***c. Each stimulates the other’s production***

CGRP increases IL-1 beta secretion from human peripheral blood mononuclear cells and CGRP is secreted in response to IL-1 in human type II alveolar epithelial cells.

***d. A cell that produces one Trefone has the receptor for the other***

No information is known.

***e. There is co-localization*** ***of one Trefone and the receptor for the other***

No information is known.

***f. The Trefones have intracellular effects or at least are internalized***

No information is known.

***g.* *A receptor for the TCC is known***

No information is known.

**(4) Other possibilities**

There are six other possible Trefone couplets discussed in this section, with various items of evidence consistent with their pairing:-

**(i) Follistatin and Myostatin**

Follistatin has three isoforms which bind members of the [TGF-β superfamily](http://en.wikipedia.org/wiki/Transforming_growth_factor_beta_superfamily) esp. the [paracrine](http://en.wikipedia.org/wiki/Paracrine) hormone activin. Myostatin is a member of the [TGF beta](http://en.wikipedia.org/wiki/Transforming_growth_factor_beta) protein family and is a secreted [growth factor](http://en.wikipedia.org/wiki/Growth_differentiation_factor) that inhibits [myogenesis](http://en.wikipedia.org/wiki/Myogenesis) and binds a membrane [receptor](http://en.wikipedia.org/wiki/Receptor_(biochemistry)), [activin type IIB.](http://en.wikipedia.org/wiki/Activin_type_2_receptors) Follistatin forms a complex with myostatin and both will stimulate proliferation of cells.

**(ii) Uteroglobin and Progesterone**

Uteroglobin (UG) is a progesterone-inducible, secreted, 10 kDa-, progesterone binding protein which is especially expressed in Clara cells in the lungs. It is bound by the lipocalin-1 receptor.

Progesterone (PG), a steroid hormone, has a nuclear progesterone receptor and two types of membrane receptors involved in “non-genomic”, rapid effects.

Both UG and PG will induce proliferative effects.

**(iii) RANKL and Osteoprotegerin**

RANKL (Receptor Activator of Nuclear factor Kappa B Ligand) is an inducer of osteoclastogenesis via its receptor RANK. RANKL forms a complex with osteoprotegerin and is known to inhibit RANKL, with Syndecan-1 as its receptor. In this case, ^a^T = RANKL, ^a^R = RANK, ^i^T = OPG and ^i^R = Syndecan-1.

**(iv) Sarcolectin and Migration Inhibitory Factor** **MIF**

In mature rheumatoid nodules, the distribution of sarcolectin-specific receptors was restricted to certain areas, including endothelial cells, and the distribution pattern of migration inhibitory factor (MIF) was similar (but not identical). Sarcolectin binds MIF so co-localization of sarcolectin receptor and MIF is consistent with the current model. MIF binds to its receptor, [CD74](http://en.wikipedia.org/wiki/CD74) (with CD44), so CD74 should appear on the other cell type which also would synthesize and release sarcolectin.

**(v) Glutamate (Glu) and Acetyl Choline (AcCh)**

Axons synthesize and release Glu and Glu is taken up by Schwann cells. These cells produces AcCh and AcCh is be taken up by axons, (although this has not been observed by others .

**(vi) WNT Interactants**

The canonical Wnt pathway leads to regulation of [gene](https://en.wikipedia.org/wiki/Gene) [transcription](https://en.wikipedia.org/wiki/Transcription_(genetics)) via stabilization of cytosolic β-catenin but is interwoven with a pathway involving an antagonist Dickkopf 1 (Dkk1). The extracellular Wnt binds to two cellular receptors LRP-5 or -6 (lipoprotein receptor-related protein-6) and Frizzled; Dkk1 binds also to LRP-5/6 and another receptor Kremen. SFRP (Secreted frizzled-related protein) and Wif (Wnt inhibitory factor) are also important as extracellular inhibitors of Wnt signalling.

**REFERENCES:-**

1.	Beaulieu JM, Gainetdinov RR: **The physiology, signaling, and pharmacology of dopamine receptors**. *Pharmacol Rev* 2011, **63**(1):182-217.

2.	Diaz J, Levesque D, Griffon N, Lammers CH, Martres MP, Sokoloff P, Schwartz JC: **Opposing roles for dopamine D2 and D3 receptors on neurotensin mRNA expression in nucleus accumbens**. *Eur J Neurosci* 1994, **6**(8):1384-1387.

3.	Adachi DK, Kalivas PW, Schenk JO: **Neurotensin binding to dopamine**. *J Neurochem* 1990, **54**(4):1321-1328.

4.	Schenk JO, Morocco MT, Ziemba VA: **Interactions between the argininyl moieties of neurotensin and the catechol protons of dopamine**. *J Neurochem* 1991, **57**(5):1787-1795.

5.	Lajiness ME, Chio CL, Huff RM: **D2 dopamine receptor stimulation of mitogenesis in transfected Chinese hamster ovary cells: relationship to dopamine stimulation of tyrosine phosphorylations**. *J Pharmacol Exp Ther* 1993, **267**(3):1573-1581.

6.	Carretero J, Vazquez RJ, Santos M, Cacicedo L, Rubio M, Sanchez-Franco F, Vazquez R: **Dopamine inhibits in vitro release of VIP and proliferation of VIP-immunoreactive pituitary cells**. *Neuropeptides* 1996, **30**(1):81-86.

7.	Camby I, Salmon I, Bourdel E, Nagy N, Danguy A, Brotchi J, Pasteels JL, Martinez J, Kiss R: **Neurotensin-mediated effects on astrocytic tumor cell proliferation**. *Neuropeptides* 1996, **30**(2):133-139.

8.	Leyton J, Garcia-Marin L, Jensen RT, Moody TW: **Neurotensin causes tyrosine phosphorylation of focal adhesion kinase in lung cancer cells**. *Eur J Pharmacol* 2002, **442**(3):179-186.

9.	Ramez M, Bagot M, Nikolova M, Boumsell L, Vita N, Chalon P, Caput D, Ferrara P, Bensussan A: **Functional characterization of neurotensin receptors in human cutaneous T cell lymphoma malignant lymphocytes**. *J Invest Dermatol* 2001, **117**(3):687-693.

10.	Maeda K, Frohman LA: **Neurotensin release by rat hypothalamic fragments in vitro**. *Brain Res* 1981, **210**(1-2):261-269.

11.	Tremblay M, Rouillard C, Levesque D: **Dopamine D3 receptor antisense reduces neuropeptide mRNA levels in rat nucleus accumbens**. *Neuroreport* 1997, **8**(18):3901-3905.

12.	Okuma Y, Fukuda Y, Osumi Y: **Neurotensin potentiates the potassium-induced release of endogenous dopamine from rat striatal slices**. *Eur J Pharmacol* 1983, **93**(1-2):27-33.

13.	Blaha CD, Phillips AG: **Pharmacological evidence for common mechanisms underlying the effects of neurotensin and neuroleptics on in vivo dopamine efflux in the rat nucleus accumbens**. *Neuroscience* 1992, **49**(4):867-877.

14.	Matsuyama S, Higashi H, Maeda H, Greengard P, Nishi A: **Neurotensin regulates DARPP-32 thr34 phosphorylation in neostriatal neurons by activation of dopamine D1-type receptors**. *J Neurochem* 2002, **81**(2):325-334.

15.	Nalivaiko E, Michaud JC, Soubrie P, Le Fur G: **Electrophysiological evidence for putative subtypes of neurotensin receptors in guinea-pig mesencephalic dopaminergic neurons**. *Neuroscience* 1998, **86**(3):799-811.

16.	Delle Donne KT, Sesack SR, Pickel VM: **Ultrastructural immunocytochemical localization of neurotensin and the dopamine D2 receptor in the rat nucleus accumbens**. *J Comp Neurol* 1996, **371**(4):552-566.

17.	Binder EB, Kinkead B, Owens MJ, Nemeroff CB: **Neurotensin and dopamine interactions**. *Pharmacol Rev* 2001, **53**(4):453-486.

18.	Cai G, Zhen X, Uryu K, Friedman E: **Activation of extracellular signal-regulated protein kinases is associated with a sensitized locomotor response to D(2) dopamine receptor stimulation in unilateral 6-hydroxydopamine-lesioned rats**. *J Neurosci* 2000, **20**(5):1849-1857.

19.	Root-Bernstein RS, Westall FC: **Serotonin binding sites. I. Structures of sites on myelin basic protein, LHRH, MSH, ACTH, interferon, serum albumin, ovalbumin and red pigment concentrating hormone**. *Brain Res Bull* 1984, **12**(4):425-436.

20.	Azmitia EC: **Modern views on an ancient chemical: serotonin effects on cell proliferation, maturation, and apoptosis**. *Brain Res Bull* 2001, **56**(5):413-424.

21.	Nemecek GM, Coughlin SR, Handley DA, Moskowitz MA: **Stimulation of aortic smooth muscle cell mitogenesis by serotonin**. *Proc Natl Acad Sci U S A* 1986, **83**(3):674-678.

22.	Fanburg BL, Lee SL: **A new role for an old molecule: serotonin as a mitogen**. *Am J Physiol* 1997, **272**(5 Pt 1):L795-806.

23.	Takuwa N, Ganz M, Takuwa Y, Sterzel RB, Rasmussen H: **Studies of the mitogenic effect of serotonin in rat renal mesangial cells**. *Am J Physiol* 1989, **257**(3 Pt 2):F431-439.

24.	Maurer M, Opitz M, Henz BM, Paus R: **The mast cell products histamine and serotonin stimulate and TNF-alpha inhibits the proliferation of murine epidermal keratinocytes in situ**. *J Dermatol Sci* 1997, **16**(1):79-84.

25.	Slauson DO, Walker C, Kristensen F, Wang Y, de Weck AL: **Mechanisms of serotonin-induced lymphocyte proliferation inhibition**. *Cell Immunol* 1984, **84**(2):240-252.

26.	Yin J, Albert RH, Tretiakova AP, Jameson BA: **5-HT(1B) receptors play a prominent role in the proliferation of T-lymphocytes**. *J Neuroimmunol* 2006, **181**(1-2):68-81.

27.	Slominski A, Pisarchik A, Zbytek B, Tobin DJ, Kauser S, Wortsman J: **Functional activity of serotoninergic and melatoninergic systems expressed in the skin**. *J Cell Physiol* 2003, **196**(1):144-153.

28.	Cossu G, Cusella-De Angelis MG, Senni MI, De Angelis L, Vivarelli E, Vella S, Bouche M, Boitani C, Molinaro M: **Adrenocorticotropin is a specific mitogen for mammalian myogenic cells**. *Dev Biol* 1989, **131**(2):331-336.

29.	Arola J, Heikkila P, Voutilainen R, Kahri AI: **Role of adenylate cyclase-cyclic AMP-dependent signal transduction in the ACTH-induced biphasic growth effect of rat adrenocortical cells in primary culture**. *J Endocrinol* 1993, **139**(3):451-461.

30.	Heikkila P, Arola J, Salmi A, Kahri AI: **ACTH-induced c-myc proto-oncogene expression precedes antimitogenic effect during differentiation of fetal rat adrenocortical cells**. *J Endocrinol* 1995, **145**(2):379-385.

31.	Jorgensen H, Kjaer A, Warberg J, Knigge U: **Differential effect of serotonin 5-HT(1A) receptor antagonists on the secretion of corticotropin and prolactin**. *Neuroendocrinology* 2001, **73**(5):322-333.

32.	Lamberts SW, Bons EG, Uitterlinden P, Hackeng WH: **Cyproheptadine and desmethylcyproheptadine directly inhibit the release of adrenocorticotrophin and beta-lipotrophin/beta-endorphin activity from the neurointermediate lobe of the rat pituitary gland**. *J Endocrinol* 1983, **96**(3):395-400.

33.	Lewis DA, Sherman BM: **Serotonergic stimulation of adrenocorticotropin secretion in man**. *J Clin Endocrinol Metab* 1984, **58**(3):458-462.

34.	Lefebvre H, Contesse V, Delarue C, Vaudry H, Kuhn JM: **Serotonergic regulation of adrenocortical function**. *Horm Metab Res* 1998, **30**(6-7):398-403.

35.	Li HD, Li XC, Zhao BY: **[Changes of the 5-HT content in hippocampus, midbrain-pons, spinal cord and concentration of blood glucose after intraperitoneal injection of ACTH in rats]**. *Zhongguo Yao Li Xue Bao* 1992, **13**(2):146-149.

36.	Calogero AE, Bagdy G, Burrello N, Polosa P, D'Agata R: **Role for serotonin3 receptors in the control of adrenocorticotropic hormone release from rat pituitary cell cultures**. *Eur J Endocrinol* 1995, **133**(2):251-254.

37.	Leone AM, Errico M, Lin SL, Cowen DS: **Activation of extracellular signal-regulated kinase (ERK) and Akt by human serotonin 5-HT(1B) receptors in transfected BE(2)-C neuroblastoma cells is inhibited by RGS4**. *J Neurochem* 2000, **75**(3):934-938.

38.	Cowen DS: **Serotonin and neuronal growth factors - a convergence of signaling pathways**. *J Neurochem* 2007, **101**(5):1161-1171.

39.	Le T, Schimmer BP: **The regulation of MAPKs in Y1 mouse adrenocortical tumor cells**. *Endocrinology* 2001, **142**(10):4282-4287.

40.	Villa I, Melzi R, Pagani F, Ravasi F, Rubinacci A, Guidobono F: **Effects of calcitonin gene-related peptide and amylin on human osteoblast-like cells proliferation**. *Eur J Pharmacol* 2000, **409**(3):273-278.

41.	White SR, Hershenson MB, Sigrist KS, Zimmermann A, Solway J: **Proliferation of guinea pig tracheal epithelial cells induced by calcitonin gene-related peptide**. *Am J Respir Cell Mol Biol* 1993, **8**(6):592-596.

42.	Umeda Y, Takamiya M, Yoshizaki H, Arisawa M: **Inhibition of mitogen-stimulated T lymphocyte proliferation by calcitonin gene-related peptide**. *Biochem Biophys Res Commun* 1988, **154**(1):227-235.

43.	Fox FE, Kubin M, Cassin M, Niu Z, Hosoi J, Torii H, Granstein RD, Trinchieri G, Rook AH: **Calcitonin gene-related peptide inhibits proliferation and antigen presentation by human peripheral blood mononuclear cells: effects on B7, interleukin 10, and interleukin 12**. *J Invest Dermatol* 1997, **108**(1):43-48.

44.	Fibbe WE, Goselink HM, Van Eeden G, Van Damme J, Billiau A, Voogt PJ, Willemze R, Falkenburg JH: **Proliferation of myeloid progenitor cells in human long-term bone marrow cultures is stimulated by interleukin-1 beta**. *Blood* 1988, **72**(4):1242-1247.

45.	Linkhart TA, Linkhart SG, MacCharles DC, Long DL, Strong DD: **Interleukin-6 messenger RNA expression and interleukin-6 protein secretion in cells isolated from normal human bone: regulation by interleukin-1**. *J Bone Miner Res* 1991, **6**(12):1285-1294.

46.	Cuesta MC, Quintero L, Pons H, Suarez-Roca H: **Substance P and calcitonin gene-related peptide increase IL-1 beta, IL-6 and TNF alpha secretion from human peripheral blood mononuclear cells**. *Neurochem Int* 2002, **40**(4):301-306.

47.	Li W, Hou L, Hua Z, Wang X: **Interleukin-1beta induces beta-calcitonin gene-related peptide secretion in human type II alveolar epithelial cells**. *Faseb J* 2004, **18**(13):1603-1605.

48.	Amthor H, Nicholas G, McKinnell I, Kemp CF, Sharma M, Kambadur R, Patel K: **Follistatin complexes Myostatin and antagonises Myostatin-mediated inhibition of myogenesis**. *Dev Biol* 2004, **270**(1):19-30.

49.	Gilson H, Schakman O, Kalista S, Lause P, Tsuchida K, Thissen JP: **Follistatin induces muscle hypertrophy through satellite cell proliferation and inhibition of both myostatin and activin**. *Am J Physiol Endocrinol Metab* 2009, **297**(1):E157-164.

50.	Li ZB, Kollias HD, Wagner KR: **Myostatin directly regulates skeletal muscle fibrosis**. *J Biol Chem* 2008, **283**(28):19371-19378.

51.	Tanaka R, Saito T, Shijubo N, Takehara M, Yamada G, Kawabata I, Itoh Y, Kudo R: **Expression of uteroglobin in normal and carcinogenic endometrium and influence of hormone replacement therapy**. *Int J Cancer* 2004, **109**(1):43-48.

52.	Peter W, Beato M, Suske G: **Recombinant rabbit uteroglobin expressed at high levels in E. coli forms stable dimers and binds progesterone**. *Protein Eng* 1989, **3**(1):61-66.

53.	Zhang Z, Kim SJ, Chowdhury B, Wang J, Lee YC, Tsai PC, Choi M, Mukherjee AB: **Interaction of uteroglobin with lipocalin-1 receptor suppresses cancer cell motility and invasion**. *Gene* 2006, **369**:66-71.

54.	Thomas P: **Characteristics of membrane progestin receptor alpha (mPRalpha) and progesterone membrane receptor component 1 (PGMRC1) and their roles in mediating rapid progestin actions**. *Front Neuroendocrinol* 2008, **29**(2):292-312.

55.	Riffo M, Gonzalez KD, Nieto A: **Uteroglobin induces the development and cellular proliferation of the mouse early embryo**. *J Exp Zool A Ecol Genet Physiol* 2007, **307**(1):28-34.

56.	Ma Y, Katiyar P, Jones LP, Fan S, Zhang Y, Furth PA, Rosen EM: **The breast cancer susceptibility gene BRCA1 regulates progesterone receptor signaling in mammary epithelial cells**. *Mol Endocrinol* 2006, **20**(1):14-34.

57.	Theoleyre S, Kwan Tat S, Vusio P, Blanchard F, Gallagher J, Ricard-Blum S, Fortun Y, Padrines M, Redini F, Heymann D: **Characterization of osteoprotegerin binding to glycosaminoglycans by surface plasmon resonance: role in the interactions with receptor activator of nuclear factor kappaB ligand (RANKL) and RANK**. *Biochem Biophys Res Commun* 2006, **347**(2):460-467.

58.	Mosheimer BA, Kaneider NC, Feistritzer C, Djanani AM, Sturn DH, Patsch JR, Wiedermann CJ: **Syndecan-1 is involved in osteoprotegerin-induced chemotaxis in human peripheral blood monocytes**. *J Clin Endocrinol Metab* 2005, **90**(5):2964-2971.

59.	Zschabitz A, Gabius HJ, Zeng FY, Kunt T, Martens KD, Koepp H, Fassbender HG, Stofft E: **Detection of sarcolectin-specific receptors like the cytokine macrophage migration inhibitory factor in rheumatoid nodules**. *Histol Histopathol* 1999, **14**(3):771-777.

60.	Merk M, Mitchell RA, Endres S, Bucala R: **D-dopachrome tautomerase (D-DT or MIF-2): doubling the MIF cytokine family**. *Cytokine* 2012, **59**(1):10-17.

61.	Okuda H, Shioda S, Nakai Y, Nakayama H, Okamoto M, Nakashima T: **Immunocytochemical localization of nicotinic acetylcholine receptor in rat hypothalamus**. *Brain Res* 1993, **625**(1):145-151.

62.	Coles JA, Abbott NJ: **Signalling from neurones to glial cells in invertebrates**. *Trends Neurosci* 1996, **19**(8):358-362.

63.	Li Y, Lu W, King TD, Liu CC, Bijur GN, Bu G: **Dkk1 stabilizes Wnt co-receptor LRP6: implication for Wnt ligand-induced LRP6 down-regulation**. *PLoS One* 2010, **5**(6):e11014.

64.	Chien AJ, Conrad WH, Moon RT: **A Wnt survival guide: from flies to human disease**. *J Invest Dermatol* 2009, **129**(7):1614-1627.
