# Supplementary material for: A model to explain specific cellular communications and cellular harmony:- a hypothesis of coupled cells and interactive coupling molecules
Source: Theor Biol Med Model. 2014 Sep 14;11:40. doi: 10.1186/1742-4682-11-40 (PMC4237941; doi:10.1186/1742-4682-11-40)
Supplement: Additional file 1 — 1. “Terminology”. 2. “Change in Cellular Characteristics”. 3. Note 1 “The Roles of SCD versus ACD”. Note 2. “Categories of SCD and ACD”. Note 3 “Further Examples of these AsCD Categories”. Note 4 “Change of differentiation type.”. 4. “The Effects of Growth Factors and other Bioactive Molecules 5. Note 1, “Additional Information on the Definition of a Trefone”. Note 2 “Clarification on Ligand and Binding Protein Interaction”. Note 3 “Cell Couplets – AsCD and the a-Cell and i-Cell”. Note 4 “Drosophila – Potential Trefones and ACD”. 6. “Calculated Levels of Trefones and TCC, with Cellular Actions”. 7. “Receptors for the Six IGFBPs and Cellular Effects”. 8. Note 1 “Extracellular Binding Proteins/ Soluble Receptors”. Note 2 “Cell Receptors for Binding Proteins/Soluble Receptors”. 9. Note 1. “Further Evidence for a Receptor for TCC”. Note 2 “Problems with Cross-linking Experiments”. 10. “Evidence for Binary Receptors aside from the IGF System”. 11. “Evidence for Two Receptors for a Particular Trefone, outside the IGF System”. 12. Note 1 “Mechanisms of Nuclear Localization”. Note 2 “NL of IGFBPs”. Note 3 “NL of Potential Trefones other than the IGF System”. 13. “Evidence Supporting the Existence of a-Cells and i-Cells”. 14. “Candidates with mainly a-Cell-Type or i-Cell-Type Characteristics”. 15. “Interacting Cells and Trefones not associated with the IGF System”. 16. “Heterogeneity/Variability of Cells in Culture.” 17. “Cell lines are not Typical of Normal Cells”. 18. “Cell Receptors and Cell Markers”. 19. “Defined Couplet Cells for Insulin and Glucagon; Gastrin and Histamine”. 20. “Potential Couplet Trefones”. 21. “Further Examples of Potential Trefone and Cell Couplets”. 22. “Examples of Cellular Regulation by Complexes”. 23. “Proteolytic enzymes and their inhibitors”. 24. “Expanded Definition of ‘Trefone’ and Classes of Couplet Cell Interactions”. 25. Note 1 “Extended Trefone Couplets”. Note 2 “Singlet Cells”. 26. “Background of Cancer Research”. [file 1742-4682-11-40-S1.zip › 3484671921331230_add1/3484671921331230_add21.doc]

**Additional File** ***TWENTY-ONE***

**(f) Evidence for Couplet Trefones and/or Couplet Cells**

***(iii) Other Evidence that is Consistent with this CTC Model***

***Further Examples of Potential Trefone Couplets and Potential Cell Couplets***

*The purpose of this File is to present evidence to justify the CTC model with further examples of possible Cell and Trefone Couplets.*

***(i) Specific Examples of Couplet Cells and Couplet Trefones***

The CTC model has two Trefones and two coupled cells with appropriate receptors in a double paracrine loop. The following evidence supports this model.

**1.** GDNF (Glial cell-derived neurotrophic factor) and soluble receptor.

The [neurotrophic factor](http://en.wikipedia.org/wiki/Neurotrophic_factor) GDNF (^a^T) binds with high affinity to the GDNF family receptor alpha-1 (GFRalpha-1, ^a^R), which is highly expressed in the midbrain. The receptor is GPI-anchored and also exists as a soluble receptor in neuronal cells, Schwann cells, and injured sciatic nerve. The GDNF:GFRalpha-1 complex binds to RET – a protein tyrosine kinase produced by [RET](http://en.wikipedia.org/wiki/RET_proto-oncogene) (“rearranged during transfection”) protooncogene. The current CTC model would have ^a^T = GDNF, ^a^R = GFRalpha-1, ^i^T = GFR-soluble; ^i^R = Unknown (or RET) and ^a^T:^i^T receptor = RET. RET may bind both soluble GFR and the TCC and have a dual role.

**2.** Fibroblast cells, when in coculture with keratinocytes, produce increased keratinocyte growth factor (KGF, FGF7) and interleukin-1 (IL-1) receptor, while the keratinocytes produce increased IL-1 and the receptor for KGF. This is a double paracrine system of the type suggested herein.

**3.** In the prostate, stromal/fibroblastic cells produce increased KGF (FGF7), an epithelial growth factor, upon induction by IL-1alpha derived from prostatic epithelial cells. This KGF can in turn lead to further epithelial cell growth and increased IL-Ialpha production. Lack of control of this double paracrine loop could explain the development of benign prostatic hyperplasia.

**4.** Similarly, IL-8 is produced by prostatic epithelial cells and it induces secretion of FGF2, another epithelial growth factor, from stromal/fibroblastic cells, which can in turn lead to further epithelial cell growth and increased IL-8 secretion.

**5.**Dendritic cells produce IL-12 which stimulates gammadeltaT cells while these T cells produce interferon-gamma which stimulates (BCG) infected dendritic cells in a reciprocal functional relationship between these cell populations.

**6.** A candidate for a couplet of cells and Trefones would be in the ectoderm of the developing *Drosophila* where Wingless binds the receptor Frizzled to stimulate Hedgehog and Hedgehog binds the receptor Patched to stimulate Wingless expression. However, there is no known evidence of a complex of the two ligands.

**FIGURE 1.**

**Interaction of Wng and Hh with receptors Frz and Ptc.** (The a-Cell could be an anterior cell and the i-Cell a posterior cell.)

***(ii) Indications of the Presence of Two Types of Cells - Possible Couplet Cells***

Of the small subpopulation of cells of the adult mouse thymus {comprising 2-3% of total cells and which are CD8alpha (-), CD4 (-)} just 50% express receptors for interleukin-2 (IL-2); perhaps an example of a prior asymmetric cell division which produced half of the cells with, and half without, IL-2 receptors. In a similar way, in primitive hematopoietic progenitors that are CD34 (+), 30-50% of the cells are IL-6R positive while 50-70% of cells generated are IL-6R negative.

In mammary tumors, binding studies suggest that there are two distinct populations of cells - one half which contains prolactin receptors and the other which possesses very few receptor sites or none at all.

In lymphocytic leukemic MOLT-4 cells, around 50% of cells express cyclins D2 and D3 upon mitogenic stimulation by phytohemagglutinin. This percentage corresponds to the percentage of cells that respond to this mitogen in cultures which indicates perhaps just two types or a heterogeneous mixture of cells. Similarly, for mouse decidual cells, Thy-1 antigens are present on about 50% of the cells, perhaps induced by hormonal control and perhaps indicating two cell types.

***(iii) Two Types of Cells in Bacteria - Possible Couplet Cells***

Even bacteria may have pairs of cells – the “haves” and the “have-nots”!

**·** With nutrient deprivation of *Bacillus subtilis*, about 50% of cells enter a path to sporulation, and the other cells do not. The potential, soon-to-sporulate cells kill nonsporulating siblings in a deliberate system of cannibalism to release nutrients for their own survival and so to delay actual sporulation.

**·** A sub-population of *Streptococcus* *pneumoniae* cells are “naturally” competent to take up free extracellular DNA and incorporate this DNA into their genomes. These have certain cell membrane molecules that allow these cells to engage in killing of non-competent siblings – a process referred to as “allolysis”.

· *E. coli* strains can cause contact-dependent growth inhibition (CDI) of other E. coli strains. The CDI depends on certain cell membrane molecules and cells with these CDI molecules {cdi(+)} directly bind to and inhibit growth of cdi(-) cells by causing a general metabolic downregulation.

· In a *Micrococcus xanthus* population, some cells have the C-signal, a surface exposed protein, and some interact with the C-signal by an assumed receptor

Again, all of this evidence is not inconsistent with the CTC model.

**REFERENCES:-**

1.	Paratcha G, Ledda F, Baars L, Coulpier M, Besset V, Anders J, Scott R, Ibanez CF: **Released GFRalpha1 potentiates downstream signaling, neuronal survival, and differentiation via a novel mechanism of recruitment of c-Ret to lipid rafts**. *Neuron* 2001, **29**(1):171-184.

2.	Saarma M: **GDNF recruits the signaling crew into lipid rafts**. *Trends Neurosci* 2001, **24**(8):427-429.

3.	Maas-Szabowski N, Stark HJ, Fusenig NE: **Keratinocyte growth regulation in defined organotypic cultures through IL-1-induced keratinocyte growth factor expression in resting fibroblasts**. *J Invest Dermatol* 2000, **114**(6):1075-1084.

4.	Giri D, Ittmann M: **Interleukin-1alpha is a paracrine inducer of FGF7, a key epithelial growth factor in benign prostatic hyperplasia**. *American Journal of Pathology* 2000, **157**(1):249-255.

5.	Giri D, Ittmann M: **Interleukin-8 is a paracrine inducer of fibroblast growth factor 2, a stromal and epithelial growth factor in benign prostatic hyperplasia**. *Am J Pathol* 2001, **159**(1):139-147.

6.	Dieli F, Caccamo N, Meraviglia S, Ivanyi J, Sireci G, Bonanno CT, Ferlazzo V, La Mendola C, Salerno A: **Reciprocal stimulation of gammadelta T cells and dendritic cells during the anti-mycobacterial immune response**. *Eur J Immunol* 2004, **34**(11):3227-3235.

7.	Ingham PW, McMahon AP: **Hedgehog signaling in animal development: paradigms and principles**. *Genes Dev* 2001, **15**(23):3059-3087.

8.	Ceredig R, Lowenthal JW, Nabholz M, MacDonald HR: **Expression of interleukin-2 receptors as a differentiation marker on intrathymic stem cells**. *Nature* 1985, **314**(6006):98-100.

9.	Tajima S, Tsuji K, Ebihara Y, Sui X, Tanaka R, Muraoka K, Yoshida M, Yamada K, Yasukawa K, Taga T *et al*: **Analysis of interleukin 6 receptor and gp130 expressions and proliferative capability of human CD34+ cells**. *J Exp Med* 1996, **184**(4):1357-1364.

10.	Costlow ME, McGuire WL: **Autoradiographic localization of prolactin receptors in 7,12-dimethylbnez[a]anthracene-induced rat mammary carcinoma**. *J Natl Cancer Inst* 1977, **58**(4):1173-1175.

11.	Gong J, Bhatia U, Traganos F, Darzynkiewicz Z: **Expression of cyclins A, D2 and D3 in individual normal mitogen stimulated lymphocytes and in MOLT-4 leukemic cells analyzed by multiparameter flow cytometry**. *Leukemia* 1995, **9**(5):893-899.

12.	Bernard O, Scheid MP, Ripoche MA, Bennett D: **Immunological studies of mouse decidual cells. I. Membrane markers of decidual cells in the days after implantation**. *J Exp Med* 1978, **148**(2):580-591.

13.	Bassler BL, Losick R: **Bacterially speaking**. *Cell* 2006, **125**(2):237-246.

14.	Steinmoen H, Knutsen E, Havarstein LS: **Induction of natural competence in Streptococcus pneumoniae triggers lysis and DNA release from a subfraction of the cell population**. *Proc Natl Acad Sci U S A* 2002, **99**(11):7681-7686.

15.	Konovalova A, Sogaard-Andersen L: **Close encounters: contact-dependent interactions in bacteria**. *Mol Microbiol* 2011, **81**(2):297-301.

16.	Aoki SK, Pamma R, Hernday AD, Bickham JE, Braaten BA, Low DA: **Contact-dependent inhibition of growth in Escherichia coli**. *Science* 2005, **309**(5738):1245-1248.
