# Supplementary material for: A model to explain specific cellular communications and cellular harmony:- a hypothesis of coupled cells and interactive coupling molecules
Source: Theor Biol Med Model. 2014 Sep 14;11:40. doi: 10.1186/1742-4682-11-40 (PMC4237941; doi:10.1186/1742-4682-11-40)
Supplement: Additional file 1 — 1. “Terminology”. 2. “Change in Cellular Characteristics”. 3. Note 1 “The Roles of SCD versus ACD”. Note 2. “Categories of SCD and ACD”. Note 3 “Further Examples of these AsCD Categories”. Note 4 “Change of differentiation type.”. 4. “The Effects of Growth Factors and other Bioactive Molecules 5. Note 1, “Additional Information on the Definition of a Trefone”. Note 2 “Clarification on Ligand and Binding Protein Interaction”. Note 3 “Cell Couplets – AsCD and the a-Cell and i-Cell”. Note 4 “Drosophila – Potential Trefones and ACD”. 6. “Calculated Levels of Trefones and TCC, with Cellular Actions”. 7. “Receptors for the Six IGFBPs and Cellular Effects”. 8. Note 1 “Extracellular Binding Proteins/ Soluble Receptors”. Note 2 “Cell Receptors for Binding Proteins/Soluble Receptors”. 9. Note 1. “Further Evidence for a Receptor for TCC”. Note 2 “Problems with Cross-linking Experiments”. 10. “Evidence for Binary Receptors aside from the IGF System”. 11. “Evidence for Two Receptors for a Particular Trefone, outside the IGF System”. 12. Note 1 “Mechanisms of Nuclear Localization”. Note 2 “NL of IGFBPs”. Note 3 “NL of Potential Trefones other than the IGF System”. 13. “Evidence Supporting the Existence of a-Cells and i-Cells”. 14. “Candidates with mainly a-Cell-Type or i-Cell-Type Characteristics”. 15. “Interacting Cells and Trefones not associated with the IGF System”. 16. “Heterogeneity/Variability of Cells in Culture.” 17. “Cell lines are not Typical of Normal Cells”. 18. “Cell Receptors and Cell Markers”. 19. “Defined Couplet Cells for Insulin and Glucagon; Gastrin and Histamine”. 20. “Potential Couplet Trefones”. 21. “Further Examples of Potential Trefone and Cell Couplets”. 22. “Examples of Cellular Regulation by Complexes”. 23. “Proteolytic enzymes and their inhibitors”. 24. “Expanded Definition of ‘Trefone’ and Classes of Couplet Cell Interactions”. 25. Note 1 “Extended Trefone Couplets”. Note 2 “Singlet Cells”. 26. “Background of Cancer Research”. [file 1742-4682-11-40-S1.zip › 3484671921331230_add1/3484671921331230_add22.doc]

**Additional File** ***Twenty-Two***

**(a)	Measurement of the Complex (TCC)**

***Examples of Cellular Regulation by Complexes***

*The purpose of this File is to present examples of complexes (especially) that interact with DNA or transcription factors to regulate cellular activity.*

Following formation of an intracellular TCC, the cell could respond to the concentration of the complex or of the Free Trefone levels. The former may be favored as there are many examples of complexes that can control transcription and protein synthesis, but free levels may be important also:-

***1. Budding yeast, Saccharomyces cerevisiae***

The diploid (a/α) cells produce genes for sporulation and other genes specific to the diploid cell but not those specific receptors and mating pheromones produced by haploid cells (a- or α-cells). This is because of the complex between the protein **a1** derived from a-cells and the protein **α2** from α-cells. This complex (**a1:α2)** and the Free **α2** repress transcription within the diploid cells, of the haploid-specific genes, which include genes necessary for mating, and also block repression of transcription of diploid-specific genes. Obviously, the Association Constant for **a1:α2** will reflect the relative amounts of complex and the amount of Free **α2,** both of which will depend on the transcription levels of **a1** and **α2**.

***2. Fission yeast, Schizosaccharomyces pombe***

A complex is formed between Swi2 and Swi5 proteins and this Swi2–Swi5 complex binds to heterochromatin. The specific localization and interaction of the complex control recombination and donor preference during mating-type switching.

***3. Toxin-Antitoxin modules***

Prokaryotes and Archaea have Toxin-Antitoxin modules in chromosomes or plasmids which prevent cell death by the Toxin component because of the formation of a non-toxic Toxin:Antitoxin Complex. The complex represses the TA operon and the antitoxin enhances this. As an example, HipA, a protein, is a critical *E. coli* persistence factor and toxin that is normally neutralized by binding to HipB, a transcription repressor protein, the antitoxin. HipA forms a complex with HipB and DNA and the structures of the HipA-HipB-DNA complex have been defined.

***4. Steroid Intracellular Receptors***

Steroids classically bind an intracellular receptor and the complex, present in the nucleus, then influences hormone-sensitive elements of DNA to change DNA transcription. Glucocorticoid receptor and androgen receptor are initially predominantly cytoplasmic while the mineralocorticoid receptor is in both the cytoplasm and nucleus. Unliganded or liganded progesterone and estrogen receptors tend to be in the nucleus. Many steroids stimulate proliferation but membrane receptors may also influence these cellular activities by “non-genomic” effects. Hormone-responsive elements of DNA are regulated by a hormone-receptor complex and this occur in the nucleus and mitochondria.

**5. Thyroid Hormone Intracellular Receptors**

Similar to steroids, thyroid hormones (T_4_ and T_3_) have nuclear receptors and cell-membrane receptors and the receptor complexes mediate the genomic and nongenomic actions of these hormones. Again, this is simply an example of a complex affecting nuclear activity.

These examples are not meant to imply that the components of the complexes are Trefones; they are referenced here as examples of other complexes that regulate gene expression.

**REFERENCES:-**

1.	Herskowitz I: **Life cycle of the budding yeast Saccharomyces cerevisiae**. *Microbiol Rev* 1988, **52**(4):536-553.

2.	Jia S, Yamada T, Grewal SI: **Heterochromatin regulates cell type-specific long-range chromatin interactions essential for directed recombination**. *Cell* 2004, **119**(4):469-480.

3.	Buts L, Lah J, Dao-Thi MH, Wyns L, Loris R: **Toxin-antitoxin modules as bacterial metabolic stress managers**. *Trends Biochem Sci* 2005, **30**(12):672-679.

4.	Schumacher MA, Piro KM, Xu W, Hansen S, Lewis K, Brennan RG: **Molecular mechanisms of HipA-mediated multidrug tolerance and its neutralization by HipB**. *Science* 2009, **323**(5912):396-401.

5.	Kumar S, Saradhi M, Chaturvedi NK, Tyagi RK: **Intracellular localization and nucleocytoplasmic trafficking of steroid receptors: an overview**. *Mol Cell Endocrinol* 2006, **246**(1-2):147-156.

6.	Psarra AM, Sekeris CE: **Nuclear receptors and other nuclear transcription factors in mitochondria: regulatory molecules in a new environment**. *Biochim Biophys Acta* 2008, **1783**(1):1-11.

7.	Davis PJ, Lin HY, Mousa SA, Luidens MK, Hercbergs AA, Wehling M, Davis FB: **Overlapping nongenomic and genomic actions of thyroid hormone and steroids**. *Steroids* 2011, **76**(9):829-833.
