# Supplementary material for: A model to explain specific cellular communications and cellular harmony:- a hypothesis of coupled cells and interactive coupling molecules
Source: Theor Biol Med Model. 2014 Sep 14;11:40. doi: 10.1186/1742-4682-11-40 (PMC4237941; doi:10.1186/1742-4682-11-40)
Supplement: Additional file 1 — 1. “Terminology”. 2. “Change in Cellular Characteristics”. 3. Note 1 “The Roles of SCD versus ACD”. Note 2. “Categories of SCD and ACD”. Note 3 “Further Examples of these AsCD Categories”. Note 4 “Change of differentiation type.”. 4. “The Effects of Growth Factors and other Bioactive Molecules 5. Note 1, “Additional Information on the Definition of a Trefone”. Note 2 “Clarification on Ligand and Binding Protein Interaction”. Note 3 “Cell Couplets – AsCD and the a-Cell and i-Cell”. Note 4 “Drosophila – Potential Trefones and ACD”. 6. “Calculated Levels of Trefones and TCC, with Cellular Actions”. 7. “Receptors for the Six IGFBPs and Cellular Effects”. 8. Note 1 “Extracellular Binding Proteins/ Soluble Receptors”. Note 2 “Cell Receptors for Binding Proteins/Soluble Receptors”. 9. Note 1. “Further Evidence for a Receptor for TCC”. Note 2 “Problems with Cross-linking Experiments”. 10. “Evidence for Binary Receptors aside from the IGF System”. 11. “Evidence for Two Receptors for a Particular Trefone, outside the IGF System”. 12. Note 1 “Mechanisms of Nuclear Localization”. Note 2 “NL of IGFBPs”. Note 3 “NL of Potential Trefones other than the IGF System”. 13. “Evidence Supporting the Existence of a-Cells and i-Cells”. 14. “Candidates with mainly a-Cell-Type or i-Cell-Type Characteristics”. 15. “Interacting Cells and Trefones not associated with the IGF System”. 16. “Heterogeneity/Variability of Cells in Culture.” 17. “Cell lines are not Typical of Normal Cells”. 18. “Cell Receptors and Cell Markers”. 19. “Defined Couplet Cells for Insulin and Glucagon; Gastrin and Histamine”. 20. “Potential Couplet Trefones”. 21. “Further Examples of Potential Trefone and Cell Couplets”. 22. “Examples of Cellular Regulation by Complexes”. 23. “Proteolytic enzymes and their inhibitors”. 24. “Expanded Definition of ‘Trefone’ and Classes of Couplet Cell Interactions”. 25. Note 1 “Extended Trefone Couplets”. Note 2 “Singlet Cells”. 26. “Background of Cancer Research”. [file 1742-4682-11-40-S1.zip › 3484671921331230_add1/3484671921331230_add23.doc]

**Additional File** ***Twenty-Three***

**(b) Variations of the Model; Other Intercellular Communication mechanisms**

***(ii) Trefones:- Enzymes Interacting with Anti-enzymes***

***Proteolytic enzymes and their inhibitors (mainly)***

*The purpose of this File is to present evidence to identify some proteolytic enzymes as Trefones.*

Many enzymes have a dual function as a catalyst and a regulator of cell proliferation. The latter may be related to a catalytic activity at a cell-membrane receptor or ligand but this would not classify it as a Trefone. A protease enzyme may activate the zymogen form of a matrix metalloproteinases or release a growth factor from its membrane-bound precursor (or pro-form) and thus indirectly affect proliferation.

It follows that any attempt to interpret evidence related to enzymes and anti-enzymes as to the existence of possible Trefones, is difficult as their effects on proliferation may be indirect and effected by their catalytic or their inhibitory activity. However, if it is a specific receptor-mediated response, then a Trefone activity may be present.

Again, in terms of evidence to support a Trefone couplet, the following expected properties will be (partially) addressed for the first two couplets:-

*1. The two couplets bind to form a stable complex*

*2. Both are known to stimulate and inhibit proliferation*

*3. Each stimulates the other’s production*

*4. A cell that produces one Trefone has the receptor for the other*

*5. Co-localization of one Trefone and the receptor for the other*

*6. Intracellular effects/Internalization*

**A. Elastase and Alpha-1-antiprotease**

Elastase of neutrophils (NE) is a protease that especially hydrolyses elastin. Normally the soluble form is bound to alpha 1-antitrypsin but membrane-bound forms also exist. Elastase is first synthesized by neutrophil precursors in bone-marrow differentiation, then stored in neutrophils, then to be released in target sites.

Alpha 1-Antitrypsin (AAT) is an inhibitor of a wide range of [proteases](http://en.wikipedia.org/wiki/Protease_inhibitor_(biology)) and is of the [serpin](http://en.wikipedia.org/wiki/Serpin) superfamily. It is also named “alpha-1 proteinase inhibitor” and counters elastase of neutrophils and of the liver (which is the largest source of AAT). AAT is also produced by neutrophil precursors and neutrophils themselves still synthesize and secrete AAT.

One couplet of cells would seem to be in the bone marrow within the neutrophil precursors with a Trefone couplet quite separate from the AAT derived from the liver.

***1. The two couplets bind to form a stable complex* *which has a receptor***

The complex of neutrophil elastase and alpha 1-antitrypsin is present in plasma and presumably in bone marrow where both NE and AAT are present, formed by a trapping of the covalently-bound elastase. The complex of AAT and elastase has a receptor on human hepatoma cells and monocytes.

***2. Both are known to stimulate and inhibit proliferation***

Elastase is a proliferator of small arteries while AAT is a proliferator of fibroblasts acting via classical signalling pathways.

Elastase has a receptor on alveolar macrophages and chondrocytes.

***3. Each stimulates the other’s production***

Neutrophil elastase stimulates an increase in steady state levels of AAT mRNA and in the rate of synthesis of AAT protein in monocytes and macrophages.

***4. A cell that produces one Trefone has the receptor for the other***	Nil known

***5. Co-localization of one Trefone and the receptor for the other***	Nil known

***6. Intracellular effects/Internalisation***

Elastase is localized to the nucleus in fibroblasts (3T3) cells while AAT activates MAP kinase signalling pathways.

B. Matrix Metalloproteinases (MMPs) **and Tissue Inhibitors of Metalloproteinases**

**(**TIMPs):

**Matrix** [**metalloproteinases**](http://en.wikipedia.org/wiki/Metalloproteinase) **(MMPs)** are [zinc](http://en.wikipedia.org/wiki/Zinc)-dependent [endopeptidases](http://en.wikipedia.org/wiki/Endopeptidase) especially active on proteins of the extracellular matrix but also active on ectoproteins (receptors, fixed ligands, precursor pro-substances) with a role in [cell proliferation](http://en.wikipedia.org/wiki/Cell_proliferation), [migration](http://en.wikipedia.org/wiki/Cell_migration) etc. The latent precursor forms are pro-MMPs and some of these are membrane bound. There are over 20 MMPs - [collagenases](http://en.wikipedia.org/wiki/Collagenase), [gelatinases](http://en.wikipedia.org/wiki/Gelatinase), stromelysins and membrane-type (MT) MMPs but in terms of the function of MMPs, others have quoted a paradigm shift from the concept of MMPs being just proteases for degrading proteins, to proteases with key signalling function.

**Tissue inhibitors of metalloproteinases (TIMPs)** are a family of four ([TIMP1](http://en.wikipedia.org/wiki/TIMP1) - TIMP4) that bind and inhibit latent or activated MMPs by binding to the catalytic site in a 1:1 stoichiometry.

The evidence to identify a MMP or a TIMP as a Trefone (in addition to qualifications above) would need to exclude the effects of activation or inhibition of these proteases in clearing the extracellular matrix (ECM) for cellular expansion by cell division, by degrading fibronectin, collagen etc, plus possibly liberating growth factors sequestered in the ECM.

There are over 20 MMPs and four TIMPs and the suggestion here is that only one couplet of these 80-odd possibilities will be Trefones for a specific couplet of cells. Identification of this couplet of cells is difficult in that preknowledge of which MMP and which TIMP were involved would be required in order to detect two cell types with specific complementary production of Trefones. One example of the separate production of these is in the subfoveal fibrovascular membranes of the eye. MMP-2 expression appeared to be localized mainly to the vascular endothelial cells, whereas TIMP-1 and TIMP-3 (both inhibitors of MMP-2) were detected in adjacent cell types, such as fibroblastlike cells.

***1. The two couplets bind to form a stable complex***

There are four families of TIMPs that generally bind and inhibit over 20 latent or activated MMPs with specificity with respect to which TIMP binds which MMP.

***2. Both are known to stimulate and inhibit proliferation***

MMP-3 enhanced the proliferation of human umbilical vein endothelial cells, but again its catalytic activity rather than a receptor response may be the reason for the proliferative effect. In contrast, MMP-3 inhibited the proliferation of Schwann cells and cutaneous neurofibroma Schwann cell cultures.

TIMPs has potent growth-promoting activity for a wide range of human and bovine cells, including TIMP-3 with a certain type of hematopoietic stem and progenitor cell and two breast cancer cell lines where TIMP-1 stimulates tyrosine-targeted phosphorylation independent of its inhibitory activity on MMPs. In contradiction to this, enzyme inhibition was assessed to be a major part of the receptor-mediated activation of intracellular kinases that occurs with TIMP-1. Paradoxical roles have been observed for TIMPs in tumorigenesis where they stimulate or inhibit various cancer cells. There is then some evidence for a definitive direct proliferative effect of TIMPs and of MMPs. Interestingly, MMP-12 (macrophage elastase) is expressed in human atherosclerotic lesions in carotid endarterectomy samples but not in normal arteries and it is upregulated in atherosclerotic lesions in transgenic rabbits overexpressing MMP-12. TIMP-3(-) foamy cell macrophages (FCMs) occurred in the deeper layers of the plaque and had an increased proliferation rate compared to TIMP-3 (+) cells while MMP-12 is also upregulated in FCMs compared to nonfoamy macrophages. Related to this, is the existence of two subpopulations of foamy cell macrophages (FCM), MMP-14^Hi^TIMP-3^Lo^ and MMP-14^Lo^TIMP-3^Hi^ [24]. MMP-14 is a membrane-type MMP that binds TIMP-3, so they are candidates to be Trefones produced by possible couplet cells of the two adjacent populations of cells - the TIMP-3 negative FCMs appearing to form discrete islands or nodules within surrounding TIMP-3 positive cells[24].

***3. Each stimulates the other’s production***	Nil known

***4. A cell that produces one Trefone has the receptor for the other***	Nil known

***5. Co-localization of one Trefone and the receptor for the other***	Nil known

***6. Intracellular effects/Internalisation***

Many receptors for MMPs have been detected. For example:- MMP-1 binds the PAR1 receptor; a MMP-2 receptor has been proposed for two human breast adenocarcinoma cells (MDA-MB231 and MCF-7); MMP-9 has the two receptors alpha4beta1-integrin and a CD44 variant on B-CLL cells which bind to a domain on MMP-9 not associated with its catalytic function; MMP-13 has a receptor on osteoblastic and fibroblastic cells which leads to its internalization. With such receptors, some type of intracellular response is usually observed eg. for MMP-9.

TIMP-1 promotes cell survival through the activation of a CD63/integrin beta1 complex on the membrane of MCF10A cells while it prevents apoptotic death in UT-7 cells via a signalling cascade activated by a ternary complex with proMMP-9 and CD44 on the cell-surface.

TIMP-1 activates cell survival signalling pathways involving focal adhesion kinase, phosphatidylinositol 3-kinase, and ERKs (extracellular-signal-regulated kinases) in human breast epithelial cells, independent of its inhibition of MMPs. The TIMP-1 is localized in the nucleus (and cytoplasm) in fibroblasts but not in a random way; rather it accumulates specifically in the nuclei of these cells in a cell-cycle dependent manner.

In addition, TIMP-1 regulates the epithelial-mesenchymal transition in a culture of a kidney cell line (MDCK), where it induces expression of transcription factors leading to downregulation of epithelial markers and upregulation of mesenchymal markers.

**C. Kallikrein and Kallistatin (= Kallikrein Binding Protein)**

The plasma serine-protease **kallikrein** ([*KLK-B1*](http://en.wikipedia.org/wiki/KLKB1)*)* liberates biologically active peptides, [kinins](http://en.wikipedia.org/wiki/Kinin), from the [kininogens](http://en.wikipedia.org/wiki/Kininogen), and the serine-protease inhibitor kallistatin (or serpin A4) binds the protease at its active site and traps the enzyme in a covalent acyl-enzyme intermediate - a binary complex.

**Kallikrein** is a proliferator of rat neural stem cells and of a keratinocyte cell line (HaCaT) but is an inhibitor in that kallikrein gene delivery inhibits vascular smooth muscle cell growth. It has two possible receptors - the bradykinin B(2) receptor and possibly the proteinase-activated receptors (PARs)

**Kallistatin** is a kallikrein binding protein which also is both stimulator (of smooth muscle cells) and inhibitor (of endothelial cells). One known kallistatin receptor is Kruppel-like factor 4 (KLF4) which is also a transcription factor. In cultured fibroblasts, Klf4 mRNA is found at high levels in growth-arrested cells and is nearly undetectable in cells that are in the exponential phase of proliferation.

**D. Thrombin and Antithrombin III**

Aside from its function in coagulation, inflammation and atherogenesis, thrombin stimulates proliferation in many cell types. For example, it stimulates smooth muscle cell (SMC) proliferation by binding to PAR-1 (protease activated receptor-1), a G-protein-coupled transmembrane receptor. Yet it is also an inhibitor of growth of other cell types - of a megakaryoblastic cell line (MEG-01) and of several tumour cell lines. It probably follows that antithrombin has a stimulatory effect in these circumstances but only via binding of thrombin.

**E. Plasmin and Alpha-2-antiplasmin**

Aside from its function in fibrinolysis, collagen remodelling and asthma pathophysiology,

plasmin induces proliferation of smooth muscle cells and does so by inducing phosphorylation of two pathways. Plasmin also has receptors on a glomerular epithelial cell line (E71-A1).

**F. Other Possible/Potential Enzymes as Trefones**

The angiotensin I converting enzyme (ACE) exists in both membrane-bound (CD143) and soluble forms and this soluble extracellular ACE is internalized, transported to the nucleus in smooth muscle and endothelial cells and triggers some cellular signalling.

Phospholipase A2 (PLA2) has a proliferative effect on cell growth via a receptor and, after the secreted enzyme (sPLA2) is internalized, it generally appears within the nucleus. In addition, sPLA2 will bind a soluble form of its receptor found in serum, making a Trefone couplet possible.

An unusual potential Trefone, autocrine motility factor (AMF), is both an ICM and an enzyme (phosphoglucose isomerase) and its receptor (AMFR) is also an enzyme (E3 ubiquitin ligase).

Carboxypeptidase is an enzyme and a sorting receptor in neuroendocrine cells while a protein with aldehyde dehydrogenase activity is a Xenopus cytosolic thyroid-hormone-binding protein.

**REFERENCES:-**

1.	Takahashi H, Nukiwa T, Basset P, Crystal RG: **Myelomonocytic cell lineage expression of the neutrophil elastase gene**. *J Biol Chem* 1988, **263**(5):2543-2547.

2.	Fouret P, du Bois RM, Bernaudin JF, Takahashi H, Ferrans VJ, Crystal RG: **Expression of the neutrophil elastase gene during human bone marrow cell differentiation**. *J Exp Med* 1989, **169**(3):833-845.

3.	Clemmensen SN, Jacobsen LC, Rorvig S, Askaa B, Christenson K, Iversen M, Jorgensen MH, Larsen MT, van Deurs B, Ostergaard O *et al*: **Alpha-1-antitrypsin is produced by human neutrophil granulocytes and their precursors and liberated during granule exocytosis**. *Eur J Haematol* 2011, **86**(6):517-530.

4.	du Bois RM, Bernaudin JF, Paakko P, Hubbard R, Takahashi H, Ferrans V, Crystal RG: **Human neutrophils express the alpha 1-antitrypsin gene and produce alpha 1-antitrypsin**. *Blood* 1991, **77**(12):2724-2730.

5.	Perlmutter DH, Glover GI, Rivetna M, Schasteen CS, Fallon RJ: **Identification of a serpin-enzyme complex receptor on human hepatoma cells and human monocytes**. *Proc Natl Acad Sci U S A* 1990, **87**(10):3753-3757.

6.	Wright JL, Farmer SG, Churg A: **A neutrophil elastase inhibitor reduces cigarette smoke-induced remodelling of lung vessels**. *Eur Respir J* 2003, **22**(1):77-81.

7.	Dabbagh K, Laurent GJ, Shock A, Leoni P, Papakrivopoulou J, Chambers RC: **Alpha-1-antitrypsin stimulates fibroblast proliferation and procollagen production and activates classical MAP kinase signalling pathways**. *J Cell Physiol* 2001, **186**(1):73-81.

8.	Campbell EJ, White RR, Senior RM, Rodriguez RJ, Kuhn C: **Receptor-mediated binding and internalization of leukocyte elastase by alveolar macrophages in vitro**. *J Clin Invest* 1979, **64**(3):824-833.

9.	Bartholomew JS, Lowther DA: **Receptor-mediated binding of leukocyte elastase by chondrocytes**. *Arthritis Rheum* 1987, **30**(4):431-438.

10.	Perlmutter DH, Travis J, Punsal PI: **Elastase regulates the synthesis of its inhibitor, alpha 1-proteinase inhibitor, and exaggerates the defect in homozygous PiZZ alpha 1 PI deficiency**. *J Clin Invest* 1988, **81**(6):1774-1780.

11.	Salipante SJ, Rojas ME, Korkmaz B, Duan Z, Wechsler J, Benson KF, Person RE, Grimes HL, Horwitz MS: **Contributions to neutropenia from PFAAP5 (N4BP2L2), a novel protein mediating transcriptional repressor cooperation between Gfi1 and neutrophil elastase**. *Mol Cell Biol* 2009, **29**(16):4394-4405.

12.	Turk B, Turk D, Turk V: **Protease signalling: the cutting edge**. *Embo J* 2012, **31**(7):1630-1643.

13.	Toft-Hansen H, Nuttall RK, Edwards DR, Owens T: **Key metalloproteinases are expressed by specific cell types in experimental autoimmune encephalomyelitis**. *J Immunol* 2004, **173**(8):5209-5218.

14.	Steen B, Sejersen S, Berglin L, Seregard S, Kvanta A: **Matrix metalloproteinases and metalloproteinase inhibitors in choroidal neovascular membranes**. *Invest Ophthalmol Vis Sci* 1998, **39**(11):2194-2200.

15.	Zheng L, Amano K, Iohara K, Ito M, Imabayashi K, Into T, Matsushita K, Nakamura H, Nakashima M: **Matrix metalloproteinase-3 accelerates wound healing following dental pulp injury**. *Am J Pathol* 2009, **175**(5):1905-1914.

16.	Muir D: **Differences in proliferation and invasion by normal, transformed and NF1 Schwann cell cultures are influenced by matrix metalloproteinase expression**. *Clin Exp Metastasis* 1995, **13**(4):303-314.

17.	Shen Y, Winkler IG, Barbier V, Sims NA, Hendy J, Levesque JP: **Tissue inhibitor of metalloproteinase-3 (TIMP-3) regulates hematopoiesis and bone formation in vivo**. *PLoS One* 2010, **5**(9).

18.	Luparello C, Avanzato G, Carella C, Pucci-Minafra I: **Tissue inhibitor of metalloprotease (TIMP)-1 and proliferative behaviour of clonal breast cancer cells**. *Breast Cancer Res Treat* 1999, **54**(3):235-244.

19.	Hayakawa T, Yamashita K, Tanzawa K, Uchijima E, Iwata K: **Growth-promoting activity of tissue inhibitor of metalloproteinases-1 (TIMP-1) for a wide range of cells. A possible new growth factor in serum**. *FEBS Lett* 1992, **298**(1):29-32.

20.	Porter JF, Shen S, Denhardt DT: **Tissue inhibitor of metalloproteinase-1 stimulates proliferation of human cancer cells by inhibiting a metalloproteinase**. *Br J Cancer* 2004, **90**(2):463-470.

21.	Jiang Y, Goldberg ID, Shi YE: **Complex roles of tissue inhibitors of metalloproteinases in cancer**. *Oncogene* 2002, **21**(14):2245-2252.

22.	Halpert I, Sires UI, Roby JD, Potter-Perigo S, Wight TN, Shapiro SD, Welgus HG, Wickline SA, Parks WC: **Matrilysin is expressed by lipid-laden macrophages at sites of potential rupture in atherosclerotic lesions and localizes to areas of versican deposition, a proteoglycan substrate for the enzyme**. *Proc Natl Acad Sci U S A* 1996, **93**(18):9748-9753.

23.	Liang J, Liu E, Yu Y, Kitajima S, Koike T, Jin Y, Morimoto M, Hatakeyama K, Asada Y, Watanabe T *et al*: **Macrophage metalloelastase accelerates the progression of atherosclerosis in transgenic rabbits**. *Circulation* 2006, **113**(16):1993-2001.

24.	Johnson JL, Sala-Newby GB, Ismail Y, Aguilera CM, Newby AC: **Low tissue inhibitor of metalloproteinases 3 and high matrix metalloproteinase 14 levels defines a subpopulation of highly invasive foam-cell macrophages**. *Arterioscler Thromb Vasc Biol* 2008, **28**(9):1647-1653.

25.	Thomas AC, Sala-Newby GB, Ismail Y, Johnson JL, Pasterkamp G, Newby AC: **Genomics of foam cells and nonfoamy macrophages from rabbits identifies arginase-I as a differential regulator of nitric oxide production**. *Arterioscler Thromb Vasc Biol* 2007, **27**(3):571-577.

26.	Boire A, Covic L, Agarwal A, Jacques S, Sherifi S, Kuliopulos A: **PAR1 is a matrix metalloprotease-1 receptor that promotes invasion and tumorigenesis of breast cancer cells**. *Cell* 2005, **120**(3):303-313.

27.	Emonard HP, Remacle AG, Noel AC, Grimaud JA, Stetler-Stevenson WG, Foidart JM: **Tumor cell surface-associated binding site for the M(r) 72,000 type IV collagenase**. *Cancer Res* 1992, **52**(20):5845-5848.

28.	Redondo-Munoz J, Ugarte-Berzal E, Terol MJ, Van den Steen PE, Hernandez del Cerro M, Roderfeld M, Roeb E, Opdenakker G, Garcia-Marco JA, Garcia-Pardo A: **Matrix metalloproteinase-9 promotes chronic lymphocytic leukemia b cell survival through its hemopexin domain**. *Cancer Cell* 2010, **17**(2):160-172.

29.	Walling HW, Chan PT, Omura TH, Barmina OY, Fiacco GJ, Jeffrey JJ, Partridge NC: **Regulation of the collagenase-3 receptor and its role in intracellular ligand processing in rat osteoblastic cells**. *J Cell Physiol* 1998, **177**(4):563-574.

30.	Jung KK, Liu XW, Chirco R, Fridman R, Kim HR: **Identification of CD63 as a tissue inhibitor of metalloproteinase-1 interacting cell surface protein**. *Embo J* 2006, **25**(17):3934-3942.

31.	Lambert E, Bridoux L, Devy J, Dasse E, Sowa ML, Duca L, Hornebeck W, Martiny L, Petitfrere-Charpentier E: **TIMP-1 binding to proMMP-9/CD44 complex localized at the cell surface promotes erythroid cell survival**. *Int J Biochem Cell Biol* 2009, **41**(5):1102-1115.

32.	Liu XW, Bernardo MM, Fridman R, Kim HR: **Tissue inhibitor of metalloproteinase-1 protects human breast epithelial cells against intrinsic apoptotic cell death via the focal adhesion kinase/phosphatidylinositol 3-kinase and MAPK signaling pathway**. *J Biol Chem* 2003, **278**(41):40364-40372.

33.	Stetler-Stevenson WG: **Tissue inhibitors of metalloproteinases in cell signaling: metalloproteinase-independent biological activities**. *Sci Signal* 2008, **1**(27):re6.

34.	Li H, Nishio K, Yamashita K, Hayakawa T, Hoshino T: **Cell cycle-dependent localization of tissue inhibitor of metalloproteinases-1 immunoreactivity in cultured human gingival fibroblasts**. *Nagoya J Med Sci* 1995, **58**(3-4):133-142.

35.	Zhao WQ, Li H, Yamashita K, Guo XK, Hoshino T, Yoshida S, Shinya T, Hayakawa T: **Cell cycle-associated accumulation of tissue inhibitor of metalloproteinases-1 (TIMP-1) in the nuclei of human gingival fibroblasts**. *J Cell Sci* 1998, **111 ( Pt 9)**:1147-1153.

36.	Jung YS, Liu XW, Chirco R, Warner RB, Fridman R, Kim HR: **TIMP-1 induces an EMT-like phenotypic conversion in MDCK cells independent of its MMP-inhibitory domain**. *PLoS One* 2012, **7**(6):e38773.

37.	Kizuki K, Iwadate H, Ookubo R: **Growth-stimulating effect of kallikrein on rat neural stem cells--II. Immunocytochemical analysis and specificity of the enzyme for neural stem cells**. *Yakugaku Zasshi* 2007, **127**(5):919-922.

38.	Gao L, Chao L, Chao J: **A novel signaling pathway of tissue kallikrein in promoting keratinocyte migration: activation of proteinase-activated receptor 1 and epidermal growth factor receptor**. *Exp Cell Res* 2010, **316**(3):376-389.

39.	Murakami H, Yayama K, Miao RQ, Wang C, Chao L, Chao J: **Kallikrein gene delivery inhibits vascular smooth muscle cell growth and neointima formation in the rat artery after balloon angioplasty**. *Hypertension* 1999, **34**(2):164-170.

40.	Hecquet C, Tan F, Marcic BM, Erdos EG: **Human bradykinin B(2) receptor is activated by kallikrein and other serine proteases**. *Mol Pharmacol* 2000, **58**(4):828-836.

41.	Oikonomopoulou K, Hansen KK, Saifeddine M, Tea I, Blaber M, Blaber SI, Scarisbrick I, Andrade-Gordon P, Cottrell GS, Bunnett NW *et al*: **Proteinase-activated receptors, targets for kallikrein signaling**. *J Biol Chem* 2006, **281**(43):32095-32112.

42.	Chao J, Miao RQ, Chen V, Chen LM, Chao L: **Novel roles of kallistatin, a specific tissue kallikrein inhibitor, in vascular remodeling**. *Biol Chem* 2001, **382**(1):15-21.

43.	Shen B, Smith RS, Jr., Hsu YT, Chao L, Chao J: **Kruppel-like factor 4 is a novel mediator of Kallistatin in inhibiting endothelial inflammation via increased endothelial nitric-oxide synthase expression**. *J Biol Chem* 2009, **284**(51):35471-35478.

44.	Shields JM, Christy RJ, Yang VW: **Identification and characterization of a gene encoding a gut-enriched Kruppel-like factor expressed during growth arrest**. *J Biol Chem* 1996, **271**(33):20009-20017.

45.	Stouffer GA, Runge MS: **The role of secondary growth factor production in thrombin-induced proliferation of vascular smooth muscle cells**. *Semin Thromb Hemost* 1998, **24**(2):145-150.

46.	Vittet D, Mathieu MN, Launay JM, Chevillard C: **Thrombin inhibits proliferation of the human megakaryoblastic MEG-01 cell line: a possible involvement of a cyclic-AMP dependent mechanism**. *J Cell Physiol* 1992, **150**(1):65-75.

47.	Huang YQ, Li JJ, Karpatkin S: **Thrombin inhibits tumor cell growth in association with up-regulation of p21(waf/cip1) and caspases via a p53-independent, STAT-1-dependent pathway**. *J Biol Chem* 2000, **275**(9):6462-6468.

48.	Nicholl SM, Roztocil E, Galaria, II, Davies MG: **Plasmin induces smooth muscle cell proliferation**. *J Surg Res* 2005, **127**(1):39-45.

49.	Becquemont L, Nguyen G, Peraldi MN, He CJ, Sraer JD, Rondeau E: **Expression of plasminogen/plasmin receptors on human glomerular epithelial cells**. *Am J Physiol* 1994, **267**(2 Pt 2):F303-310.

50.	Igic R, Behnia R: **Properties and distribution of angiotensin I converting enzyme**. *Curr Pharm Des* 2003, **9**(9):697-706.

51.	Lucero HA, Kintsurashvili E, Marketou ME, Gavras H: **Cell signaling, internalization, and nuclear localization of the angiotensin converting enzyme in smooth muscle and endothelial cells**. *J Biol Chem* 2009, **285**(8):5555-5568.

52.	Arita H, Hanasaki K, Nakano T, Oka S, Teraoka H, Matsumoto K: **Novel proliferative effect of phospholipase A2 in Swiss 3T3 cells via specific binding site**. *J Biol Chem* 1991, **266**(29):19139-19141.

53.	Fayard JM, Tessier C, Pageaux JF, Lagarde M, Laugier C: **Nuclear location of PLA2-I in proliferative cells**. *J Cell Sci* 1998, **111 ( Pt 7)**:985-994.

54.	Higashino Ki K, Yokota Y, Ono T, Kamitani S, Arita H, Hanasaki K: **Identification of a soluble form phospholipase A2 receptor as a circulating endogenous inhibitor for secretory phospholipase A2**. *J Biol Chem* 2002, **277**(16):13583-13588.

55.	Fairbank M, St-Pierre P, Nabi IR: **The complex biology of autocrine motility factor/phosphoglucose isomerase (AMF/PGI) and its receptor, the gp78/AMFR E3 ubiquitin ligase**. *Mol Biosyst* 2009, **5**(8):793-801.

56.	Cool DR, Normant E, Shen F, Chen HC, Pannell L, Zhang Y, Loh YP: **Carboxypeptidase E is a regulated secretory pathway sorting receptor: genetic obliteration leads to endocrine disorders in Cpe(fat) mice**. *Cell* 1997, **88**(1):73-83.

57.	Yamauchi K, Tata JR: **Characterization of Xenopus cytosolic thyroid-hormone-binding protein (xCTBP) with aldehyde dehydrogenase activity**. *Chem Biol Interact* 2001, **130-132**(1-3):309-321.
