# Supplementary material for: A model to explain specific cellular communications and cellular harmony:- a hypothesis of coupled cells and interactive coupling molecules
Source: Theor Biol Med Model. 2014 Sep 14;11:40. doi: 10.1186/1742-4682-11-40 (PMC4237941; doi:10.1186/1742-4682-11-40)
Supplement: Additional file 1 — 1. “Terminology”. 2. “Change in Cellular Characteristics”. 3. Note 1 “The Roles of SCD versus ACD”. Note 2. “Categories of SCD and ACD”. Note 3 “Further Examples of these AsCD Categories”. Note 4 “Change of differentiation type.”. 4. “The Effects of Growth Factors and other Bioactive Molecules 5. Note 1, “Additional Information on the Definition of a Trefone”. Note 2 “Clarification on Ligand and Binding Protein Interaction”. Note 3 “Cell Couplets – AsCD and the a-Cell and i-Cell”. Note 4 “Drosophila – Potential Trefones and ACD”. 6. “Calculated Levels of Trefones and TCC, with Cellular Actions”. 7. “Receptors for the Six IGFBPs and Cellular Effects”. 8. Note 1 “Extracellular Binding Proteins/ Soluble Receptors”. Note 2 “Cell Receptors for Binding Proteins/Soluble Receptors”. 9. Note 1. “Further Evidence for a Receptor for TCC”. Note 2 “Problems with Cross-linking Experiments”. 10. “Evidence for Binary Receptors aside from the IGF System”. 11. “Evidence for Two Receptors for a Particular Trefone, outside the IGF System”. 12. Note 1 “Mechanisms of Nuclear Localization”. Note 2 “NL of IGFBPs”. Note 3 “NL of Potential Trefones other than the IGF System”. 13. “Evidence Supporting the Existence of a-Cells and i-Cells”. 14. “Candidates with mainly a-Cell-Type or i-Cell-Type Characteristics”. 15. “Interacting Cells and Trefones not associated with the IGF System”. 16. “Heterogeneity/Variability of Cells in Culture.” 17. “Cell lines are not Typical of Normal Cells”. 18. “Cell Receptors and Cell Markers”. 19. “Defined Couplet Cells for Insulin and Glucagon; Gastrin and Histamine”. 20. “Potential Couplet Trefones”. 21. “Further Examples of Potential Trefone and Cell Couplets”. 22. “Examples of Cellular Regulation by Complexes”. 23. “Proteolytic enzymes and their inhibitors”. 24. “Expanded Definition of ‘Trefone’ and Classes of Couplet Cell Interactions”. 25. Note 1 “Extended Trefone Couplets”. Note 2 “Singlet Cells”. 26. “Background of Cancer Research”. [file 1742-4682-11-40-S1.zip › 3484671921331230_add1/3484671921331230_add24.doc]

**Additional File** ***Twenty-Four***

**(b) Variations of the Model; Other Intercellular Communication Mechanisms**

***(i) Expanded definition of “Trefone” and Classes of Couplet Cell Interactions***

*The purpose of this File is to present a speculative, expanded classification of intercellular interactions with examples of redefined Trefone couplets and enzyme interventions.*

**Trefones and Classes of Cells**

The definition of a Trefone is expanded here from soluble molecules to include molecules that are fixed to the cell membrane. They may be internalized but are not secreted. The simple term “Trefone” then means a secreted soluble molecule in contrast to the term “fixed Trefone”.

In the following discussion, molecules which are initially membrane-attached but are cleaved to release a soluble Trefone will be referred to as *proTrefones* (proT). Trefones that are converted by interaction will be referred to as *derived Trefones* (Td). A Td may be internalized but not a proTrefone.

The distinction between a fixed and a soluble Trefones is a grey area. With regulated intramembrane proteolysis (RIP), the extracellular domain of a fixed receptor can be removed to produce a soluble receptor which is equivalent to a “binding protein”. Equally, the extracellular domain of a fixed or ligand precursor (proligand) can be removed to produce a soluble “ligand”. Both of these products (the binding protein and ligand) are “Trefones” in this discussion. For example, proteolysis of the fixed receptor for human growth hormone occurs at the cell surface and releases the extracellular domain, which is the growth hormone-binding protein (GHBP). The tumour necrosis factor-alpha-converting enzyme (TACE) will release this soluble GHBP. This same enzyme will release the ligand, the transforming growth factor-alpha (TGF-alpha), from the transmembrane form of the TGF-alpha (proTGF-alpha). Three of the four activating TGF-alpha ligands in *Drosophila* are secreted after proteolytic release from the proforms within the membrane, to interact with the epidermal growth factor receptor (EGFR) (DER) in its two fixed isoforms. TACE and other ADAMs (A Disintegrin And Metalloproteinase), are involved in a wide range of other transmembrane conversions. Ligands that bind the EGF receptor are initially fixed and are released from mammary epithelial cells by an ADAM activity to produce the soluble ligands. The ligand for RANK (RANKL or TRANCE) is initially membrane-bound but solubilised upon cleavage by TACE.

Indeed, shedding of membrane-anchored growth factors and receptors by ADAM proteinases has been proposed as a means of signal transduction between neighbouring pairs of cell types in a paracrine fashion (along with possible autocrine and juxtacrine activities) involving the soluble growth factors and receptors.

**CLASSIFICATION - Listing:-**

**CLASS A – Fixed Trefones**

(i) Identical Cells.

*(a) Homophilic Fixed Trefones*

*(b) Heterophilic Fixed Trefones*

(ii) Non- Identical, Related Cells; Two Types**.**

*(a) Homophilic Fixed Trefones.*

*(b) Heterophilic Fixed Trefones.*

*(i) One fixed Trefone on one cell type and a pairing, fixed Trefone on*

*the other cell type.*

*(ii) Two different fixed Trefones on each cell type*

**CLASS B –Fixed Trefone/ProTrefone, with Enzyme Intervention. Non-identical Cells;**

**Two Types.**

(i) One cell type with an extracellular receptor and the other with an IC receptor

(ii) Ectodomain shedding. Shedding of a Trefone receptor but this releases a Trefone

(iii) GPI-anchored – Enzyme or Trefone.

**CLASS C -** **Non-Identical, Related Cells, Two Types; One Soluble Trefone, one Fixed Pre-Trefone, Receptor Mediated Uptake: Enzymes Process(es) Involved (Proteolytic or Transferase).**

**CLASS D – Soluble Non-interactive Trefones, Receptor Mediated.**

(i) Identical Cells; Each produces one Trefone and one receptor.

(ii) Non- Identical, Related Cells; Two Types, one produces the Trefone, the

other produces the receptor

(iii) Non- Identical, Related Cells; Two Types, one produces one Trefone plus the

receptor for the Trefone which is produced by the other cell

**CLASS E – Non-Identical, Related Cells, Two Types; Two Soluble Interactive Trefones, Receptor Mediated Uptake.**

(i) The Two Soluble Trefones form a Complex which is detected Extracellularly.

(ii) The Two Soluble Trefones form a Complex which is detected Intracellularly.

(iii) Each Trefone is modified by the Other – Baton-message transfer producing

Two Derived Trefones

**CLASS F	Two Soluble Interactive Trefones, Enzyme Process(es) Involved. – (Non-Identical, Related Cells, Two Types; Receptor Mediated Uptake)**

(i) Two Trefones:- A Pro-Trefone is a Substrate, one Trefone is an Enzyme and

the Product of the Enzyme-catalysed Reaction (Proteolytic or

Transferase) is a Trefone.

(ii) Two Trefones:- Two Pro-Trefones are Substrates, two Trefones are Enzymes

and the Products of the Enzyme-catalysed Reactions (Proteolytic or

Transferase) are Trefones.

(iii) Four Trefones:- One Trefone is a Substrate, one an Enzyme, two are the

Derived Products of the Enzyme-catalysed Reaction (Proteolytic or

Transferase). No receptor for any complex.

(iv) Four Trefones:- Two Trefones are Substrates, two Enzymes that are not

Trefones, two Trefones are the Products of the Enzyme-catalysed

**CLASS G	One Fixed Pro-Trefone, Soluble Interactive Trefones, Enzymes Process(es) Involved. – (Non-Identical, Related Cells, Two Types; Receptor Mediated Uptake)**

(a) Enzyme either Cell-bound or Secreted by External Cells.

(b) Enzyme Secreted by one of the Paired Cells.

**(**c) Enzyme either Cell-bound or Secreted by External Cells and Enzyme

Secreted by one of the Paired Cells.

**CLASS H	Other Less-Likely Variations**

**CLASSIFICATION – Specifications and examples:-**

**CLASS A – Fixed Trefones**

The Trefones are fixed to cell membranes and, when transmitting a signal from the same or a bound Trefone, are not internalized. The signal could be transferred chemically, via an electronic “pulse” through the polypeptide backbone or physically, associated with a type of allosteric effect to change the 3-D structure. This would be whereby the binding of a large protein to a domain or domains or to the end (N- or C-terminal) of the *extra*cellular part of the transmembrane protein, would produce a change in structure of a domain or domains or to the end (C- or N-terminal) of the *intra*cellular part of the transmembrane protein. One-component systems, where a signal transduction occurs in a single protein molecule, involve an input signal at one end of the molecule which produces an output response at the other end, and they dominate signal transduction in prokaryotes. This could be bidirectional/ reciprocal with the external contact of the protein affecting each cell internally. This process may be constrained by the actual within-membrane moiety but the interaction may be enhanced by multiple binding external domains in multiple-pass transmembrane Trefones. Telesensing via physical interaction could be part of the communication system.

A fixed Trefone may be a cell adhesion molecule - a CAM. But CAMs may have other functions. The interaction between CAMs in adjacent cells may contribute to the organized aggregation, cell recognition and adhesion of cells into organs during embryonic development, while, in some cases, the CAM is linked to the cytoskeleton forming a fixed anchor. The linkage may only be self-recognition or recognition between closely related clones of cells. A CAM will only be a fixed Trefone if it has a second function, namely, it can affect cell-life decisions eg proliferation, and does not just physically affect cell organization for growth and/or cell migration.

**(i) Identical Cells**

The interaction between cells of the same clone is about knowing if your neighbour is identical to yourself or not.

***(a) Homophilic Fixed Trefones***

**Figure 1: An example of these Cells Interacting**

This would be a simple mechanism to hold identical cells together in a tissue or group. Each cell may have just a single type of fixed Trefone which binds an identical fixed Trefone on a neighbouring cell, involving thus a dimer formation. E-cadherin and N-CAMs may belong in this category.

***(b) Heterophilic Fixed Trefones*–**

**Figure 2: An example of these Cells Interacting**

This would also be a simple mechanism to hold identical cells together. Each cell here has one or more pairs of fixed Trefones but these are not couplet, fixed Trefones as they are not produced specifically by opposed cells in a cell couplet.

**(ii) Non- Identical, Related Cells; Two Types**

***(a) Homophilic Fixed Trefones. Two different cell types produce the same fixed Trefone.***

**Figure 3: An example of these Cells Interacting**

This would be a simple mechanism to hold related cells together. This interaction, and the following heterophilic types, may change in cancer to allow separation of cells and metastasis.

***(b) Heterophilic Fixed Trefones***

***(i) One fixed Trefone on one cell type and a pairing, fixed Trefone on the other cell type***

***There may be more than one pair of Trefones.***

**Figure 4: An example of these Cells Interacting**

A receptor can be considered a fixed Trefone, in that the soluble receptor is a Trefone, as for the well-known GH receptor and GFBP. There are several examples of this type of interaction of two fixed Trefones, where a receptor is a fixed Trefone:-

**.** The ephrin “ligands” and the Eph “receptors” form a large system of fixed cell-membrane substances and function in a contact-mediated fashion between two opposing cells. T_1_ of **Figure 4**, could be an ephrin (A and/or B) on a cell and T_2_ an Eph “receptor” (A and/or B) on another cell and bidirectional signal transduction may occur. This means that the transduction can both into the receptor cell (a process termed “forward signalling”) and into the ligand cell (“reverse signalling”). Some ephrins also have functional roles as soluble forms.

**.** The antigen presenting cells (APC) {B cells, macrophages, dendritic cells (DCs) and thymic epithelia} have CD40, CD80 and CD86 membrane proteins. These have a paired interaction with membrane proteins CD154, with CTLA4 and CD28, respectively, on T cells, to induce proliferation. These are costimulatory to the primary stimulus of the MHC presented antigen and the T-cell receptor but all cell couplings will not necessarily have all three pairs, perhaps not even two. The stimulus is one way.

**.** The neurexins associated with the presynaptic axon terminal form a coupling adhesion within the synaptic cleft with the neuroligins associated with the postsynaptic terminal of the dendritic spine. The neurexins are potentially involved in specific synaptic development, including induction and stabilization.

**.** In vulval development in *C. elegans*, one of two progeny cells from cell division becomes an AC (anchor cell) or a VU (vulval uterine) cell, by a maturation stage. At the end of the decision-making process as to which cell becomes the AC, lin-12 (a receptor) accumulates on the “to-be” AC and interacts with lag-2, a transmembrane ligand for lin-12, and is finally expressed only on the “to-be” UV cell.

**.** The interaction of the Fas (receptor) on a target cell (eg. a tumour cell) and the Fas ligand on a T-cell would cause apoptosis of the target cell via a one-directional signal. This is a simple view, complicated by the possibility that both cells may have Fas and/or FasL and further, because soluble FasL, released by a MMP, may be present to inhibit the death signal. Soluble Fas could further add to the battle tactics.

**.** Membrane-bound RANK ligand (CD254) in osteoblasts interacts with receptor RANK in progenitor osteoclast cells .

**.** Membrane-bound stem cell factor (SCF, steel factor) interacts with membrane receptor cKit. In a one-directional stimulation, the cKit tyrosine kinase is activated, more persistently than soluble SCF.

**.** This type of interaction may also apply in contact-dependent growth inhibition in *E. coli*.

***(ii) Two different fixed Trefones on each cell type***

**Figure 5. An example of these Cells Interacting**

Cell-to-cell signalling involving C-signal (a surface protein) transmission via a receptor in *Myxococcus xanthus*, may be an example of this type.

In *C. elegans*, prior to the maturation of cells described above, the fixed ligand (Lag-2) and the receptor Lin-12 are distributed equally on the two progeny.

**CLASS B –Fixed Trefone/ProTrefone, *with Enzyme Intervention.* Non-identical Cells; Two Types**

(i) **One cell type with an extracellular receptor and the other with an intracellular (IC) receptor – Figure 6.** **Notch and Delta-like**

**`**

The enzymes involved in the processing of Notch are TACE (Adam 17) (S2) and gamma secretase complex (S3) which, together, release the Notch IntraCellular Domain (NICD) which then moves to the nucleus of Cell 1. The NICD then interacts with the DNA-binding protein RBPJ (an intracellular receptor) to regulate the expression of target genes and to maintain proliferation. It is proposed here that the complex of the residual product from Notch plus the Delta-like membrane protein is internalized into Cell 2 where the complex or the Notch residue would also transmit a message to maintain proliferation. There is also a 33-amino-acid part of Notch between the S2 and S3 enzyme sites. Similar regulated intramembrane proteolysis (RIP) also occurs to other transmembrane proteins. In the processing of the transmembrane tumour-necrosis factor-α precursor (TNFalphaFL) and of the Amyloid Precursor Protein (APP)**,** intracellular peptides analogous to NICD are also produced – TNFalphaICD and AICD respectively. In each case, with proteolytic cleavages, two extracellular peptides are also produced, soluble TNF-alpha plus TNFalpha C-domain and soluble APP plus the P3 peptide (or amyloid beta), respectively.

The intracellular soluble E4ICD derived from the [epidermal growth factor receptor](http://en.wikipedia.org/wiki/ErbB), ErbB4, by proteolytic release after ligand stimulation by neuregulin-1, also has a direct nuclear function. The E4ICD in complex with other proteins, translocates to the nucleus where it binds to specific promoter elements to repress the expression of certain genes. Metalloproteinase cleavage of an ephrin (ligand) will release its extracellular domain which binds the Eph receptor to permits endocytosis of the complex and the residual cytoplasmic cleavage fragments of the ephrin may directly affect signalling through relevant kinases.

**(ii) Ectodomain shedding. Shedding of a Trefone receptor but this releases a Trefone**

TNFR1 can be released from cells to the extracellular compartment by proteolytic cleavage {ie. regulated intramembrane proteolysis (RIP)} of the TNFR1 extracellular domain. This process, called ectodomain shedding, generates soluble TNF-binding protein, a Trefone that may inhibits TNF bioactivity. Glycosylphosphatidylinositol **(**GPI)-anchored proteins are a special example here. [Phospholipase C](http://en.wikipedia.org/wiki/Phospholipase_C) will cleave the phospho-glycerol bond of GPI-anchored proteins to release the protein which may be a soluble receptor. For example, a GPI-anchored receptor for GDNF (glial cell line-derived neurotrophic factor) can be cleaved to release a soluble receptor for GDNF.

**CLASS C -** **Non-Identical, Related Cells, Two Types; One Soluble Trefone, one Fixed Pre-Trefone, Receptor Mediated Uptake: *Enzymes Process(es) Involved (Ectodomain shedding; RIP)* – Figure 7. CD44 processing**

***Ectodomain shedding; Regulated intramembrane proteolysis (RIP).***

CD44 and soluble hyaluronic acid interact as in Figure 7. Hyaluronic acid (HA) has an activity of a Trefone; it induce the proliferation of melanoma cells. Also, CD44 regulates [keratinocyte](http://en.wikipedia.org/wiki/Keratinocyte) proliferation in response to extracellular stimuli. Membrane-bound CD44 will bind soluble HA derived from another type of cell and this will induce the processing of CD44 by enzymes to release the CD44 intracellular domain CD44ICD plus soluble CD44. The CD44ICD is translocated to the nucleus and acts as a signal transduction molecule, activating transcription. The soluble CD44 would bind HA and be bound by the other type of cell.

**CLASS D – *Soluble Non-interactive* Trefones, Receptor Mediated**

**(i) Identical Cells; Each produces one Trefone and one receptor**

***Figure 8: An example of these Cells Interacting***

This type of interaction may occur in bacterial quorum-sensing, with a soluble diffusible ICM, for example a cyclic dipeptide shared by a bacterial species.

**(ii) Non- Identical, Related Cells; Two Types, one produces the Trefone, the other the receptor**

***Figure 9: An example of these Cells Interacting***

This is a basic unidirectional communication of use by one cell type to instruct a response from another cell. This may be a feed-back inhibition effect of a hormone eg. thyroxine from a thyroid cell affecting a pituitary cell that produces the TSH hormone to stimulate the thyroid cell. While this, no doubt, occurs in an organism, it needs to be part of a coupled system to be balanced.

**(iii) Non- Identical, Related Cells; Two Types, one produces one Trefone plus the receptor for the Trefone which is produced by the other cell**

***Figure 10: An example of these Cells Interacting***

This is similar to the proposed CTC model but there is no interaction between the Trefones to produce the necessary balance.

**CLASS E – Non-Identical, Related Cells, Two Types; Two Soluble *Interactive* Trefones, Receptor Mediated Uptake**

**(i) The Two Soluble Trefones form a Complex which is detected Extracellularly**

***Figure 11: An example of these Cells Interacting***

This is the CTC model with a TCC receptor as discussed prior to this classification of the

possible variations.

**(ii) The Two Soluble Trefones form a Complex which is detected Intracellularly**

***Figure 12: An example of these Cells Interacting***

This is the CTC model with an intracellular formation of a TCC complex as discussed

just prior to this classification of possible variations. An extracellular TCC would be

formed but is not included in the diagram

**(iii) Each Trefone is Modified by the Other – Baton-message transfer producing Two Derived Trefones**

***Figure 13: An example of these Cells Interacting***

One of the Trefones (^i^T here) would have transferase or protease enzymic activity, but the enzyme intermediate with the transferred group, would be stabilised. The enzymic protein is not acting as a catalyst here but forms an “enzyme intermediate”, stoichiometrically. The baton-message, (b) in **Figure 13**, could be a phosphate group, a carbohydrate (eg. glycosylated), an acyl group or ADP. A protease could transfer a peptidyl derivative as the baton-message in a transpeptidation reaction where an acyl intermediate is formed with no subsequent hydrolysis. A glutamyl group could be transferred from one protein to the enzyme by itself, a transglutaminase.

Perhaps an added signal can be transmitted by an ADP-ribosyltransferases (ARTs) expressed on the cell surface or secreted in the extracellular compartment. An ADP-ribose group from NAD^+^ can be transferred onto target proteins to produce “ADP-ribosylation” of a ligand (FGF-2 ) and a receptor {PDGF(BB)}.

**CLASS F	Two Soluble Interactive Trefones, *Enzymes Process(es) Involved.* – (Non-Identical, Related Cells, Two Types; Receptor Mediated Uptake)**

**Enzyme(s) Secreted by either or both of the Paired Cells**

**(i) Two Trefones:- A Pro-Trefone is a Substrate, one Trefone is an Enzyme and the Product of the Enzyme-catalysed Reaction (Proteolytic or Transferase) is a Trefone. The complex has a receptor.**

***Figure 14: An example of the Cells Interacting***

The Pro-^a^T is converted to the actual Trefone by the enzyme ^i^T.

**(ii) Two Trefones:- Two Pro-Trefones are Substrates, two Trefones are Enzymes and the Products of the Enzyme-catalysed Reactions (Proteolytic or Transferase) are Trefones. The complex has a receptor.**

***Figure 14: An example of these Cells Interacting***

The Pro-^a^T is converted to the actual ^a^Trefone (which now has enzymic activity) by the enzyme ^i^T. This Trefone is produced from the Pro-iT by the ^a^T enzyme. The reciprocal activation of enzymes from precursors (Pro-uPA and Plasminogen) to uPA and plasmin could be an example of this proposal, but a complex of uPA and plasmin has not been reported

**(iii) Four Trefones:- One Trefone is a Substrate, one an Enzyme, two are the Derived Products of the Enzyme-catalysed Reaction (Proteolytic or Transferase). No receptor for any complex**

***Figure 15: An example of these Cells Interacting***

Two products, now Trefones, are derived from the cleavage of ^a^T by the enzyme ^i^T

**(iv) Four Trefones:- Two Trefones are Substrates, two Enzymes that are not Trefones, two Trefones are the Products of the Enzyme-catalysed Reactions (Proteolytic or Transferase). No receptor for any complex**

***Figure 16: An example of these Cells Interacting***

Each cell is now producing two components to the interactions – an enzyme and a Trefone. Each Trefone is enzymically converted to a derived Trefone (Td) and each cell has receptors for the Trefone from the other cell and the Td from that Trefone

**Or**

***Figure 17: An example of these Cells Interacting***

This is a variation on Figure16 where now each cell has a receptor for the Trefone from the other call and a receptor for the derived Trefone from the Trefone it produced.

**CLASS G	One Fixed Pro-Trefone, Soluble Interactive Trefones, *Enzymes Process(es) Involved.* – (Non-Identical, Related Cells, Two Types; Receptor Mediated Uptake)**

**(a) Enzyme either Cell-bound or Secreted by External Cells**

***Figure 18: An example of these Cells Interacting***

The Pro-^a^T is converted to the actual ^a^Trefone by an external enzyme, perhaps from a specific third cell. It is then similar to the original model with a receptor for the TCC.

This model can be proposed for the urokinase-type plasminogen activator, uPA, where the Pro-uPA is converted by soluble or cell-bound plasmin to uPA (^a^T), which interacts with uPAR (^a^R). The plasminogen activator inhibitor-1 (PAI-1 = ^i^T)) forms a complex with uPA, which can be internalized. PAI-1 itself can be bound to LDL receptor-related protein (LRP = ^i^R).

The separate cellular existences of the Trefone, uPa, and its specific cell-surface receptor, uPAR, are consistent with this paracrine effect. In thirteen adenomas of the colon, u-PA mRNA was present in fibroblastlike cells in the stroma adjacent to the invasive tumour nodules but not in the malignant cells. The u-PAR mRNA was located in cells of malignant origin at the tumoral-stromal interface of invasive foci in all but one adenoma, supporting the paracrine interactions. Thus complementation and synergism of urokinase and its receptor are likely because of their presence on separate cells.

(A complicating factor may be the existence of soluble uPAR released from the [cell membrane](http://en.wikipedia.org/wiki/Cell_membrane) to which it is tethered with a [glycosylphosphotidylinositol](http://en.wikipedia.org/w/index.php?title=Glycosylphosphotidylinositol&action=edit&redlink=1) (GPI) anchor.)

Plasminogen, plasmin and alpha2-antiplasmin (or alpha2-plasmin inhibitor) may also have a cellular relationship that is similar to the above with the known presence of a plasmin receptor. The plasmin does form a complex with alpha2-antiplasmin but plasmin also forms a complex with alpha_2_-macroglobulin which is internalized by certain cells.

**(b) Enzyme Secreted by one of the Paired Cells.**

**Four Trefones:- One ProTrefone is a Fixed Receptor which is a Substrate for another Trefone, an Enzyme, two Trefones are the Products of the Enzyme-catalysed Reaction, another Trefone is the Anti-enzyme which forms a Complex with the Enzyme.**

***Figure 19: An example of the Cells Interacting***

The Trefone with enzymic activity (^a^T), acts on a membrane ProTrefone to produce a derived fragment (Td) and the Trefone (T) which is internalized. With receptors for the ^a^T, Td and ^i^T, a receptor for the TCC may not be necessary as in many cases, the emphasis is on each cell acquiring two messages to prevent signalling errors.

**(c) Enzyme either Cell-bound or Secreted by External Cells and Enzyme Secreted by one of the Paired Cells**

***Figure 20: The interaction of cells producing Thrombin and Antithrombin***

The thrombin system seems to fit in this category which is a mix of (a) plus (b) of this Class.

The thrombin substrate, protease-activated receptor 1 (PAR-1), is a member of the G protein-coupled 7-transmembrane domain receptor family. Thrombin (activated from ProTh by soluble or cell-bound Factor Xa) hydrolyses the tethered peptide from the pre-Trefone, PAR, {(i) to (ii)} to produce the 41-amino-acid peptide referred to here as the derived Trefone Td, with its proposed receptor (R_Td_). The activated PAR-1 is internalized for signalling but is not recycled (Trejo, 98) and the thrombin receptor (R_Th_) is thrombomodulin. The complex of thrombin and antithrombin is internalized via the LDL receptor-related protein 1 (LRP1) as is thrombin itself. The cell C_1_ would be a liver cell, the cell C_2_ could be an endothelial cell. The proposed model does not include the added complexity of thrombin’s interaction with (activated) protein C and the membrane and soluble forms of endothelial cell protein C receptor, the latter of which is indicative of thrombotic risk in cancer patients.

Thrombin also forms a binary complex with protease nexin 1 (PN1) involving a covalent linkage, which is also internalized by LRP1. This system may operate in other cell couplets.

**CLASS H	Other Less-Likely Variations**

Nature has few restraints and other variations and combinations could be envisaged:-

**.** Complementary enzymes could be produced **intracellularly** by paired cells, acting to produce unique products as secreted signal molecules.

Other **mixed intracellular and extracellular** classes could be envisaged to cater for the prorenin/rennin system. While prorenin is bound by and signals via the prorenin receptor (PRR), it is also bound and internalized by the mannose 6-phosphate/insulin-like growth factor II receptor which also activates it to rennin, intracellularly. Also, prorenin (normally proteolytically activated to rennin and which has a binding protein) binds to a receptor to produce a conformational change and rennin activity is generated. In addition, there may be more than two receptors.

**.** Perhaps **a triplet of cell types** could communicate instead of a couplet.

Three cells could be formed by division of a cell into three parts **or** derived from two cell divisions (AsCD) with the fourth cell “lost” by apoptosis, with each interacting with the two others in a triumvirate. There may be three cells in a trinity or triumvirate derived from cell division of a precursor to produce cells ^a^C and ^i^C, plus a different precursor cell producing ^q^C and ^d^C, where the latter dies.

^a^C would produce ^a^T and bind ^q^T and ^q^T/^i^T, ^i^C would produce ^i^T and bind ^a^T and ^a^T/^q^T while ^q^C would produce ^q^T and bind ^i^T and ^i^T/^a^T.

A candidate for this triplet is in the ectoderm of the developing *Drosophila* where Wingless is needed for Engrailed expression, Engrailed is needed for Hedgehog expression and Hedgehog is needed for Wingless expression. This may not agree with all evidence.

Another possibility is a triplet of cells in the pancreas, the alpha- beta- and gamma-cells producing a trio of Trefones, glucagon, insulin and somatostatin respectively.

Also possible is a triplet in the gut. The release of gastrin from G cells, histamine from ECL cells, hydrochloric acid from parietal cells could constitute an interactive trio.

In both of these organs, couplet cells have been suggested elsewhere.

**.** Interaction between mobile (eg. neutrophils) and fixed cells (eg. epithelial cells)

**.** Complexity added by membrane proteins with functions of enzymes and receptors

(a) Catalytic activity could be irrelevant to the receptor processing.

Adenosine deaminase, an ectoenzyme, binds another cell-bound protein, CD26,

which is also a dipeptidyl peptidase.

(b) Complexity associated with various activities:-

(i) ACE (Angiotensin Converting Enzyme), added externally, is internalized to the

nucleus.

(iii) Enzyme activity is sometimes important for cell proliferation:-

Phospholipase 2 and autotoxin produce phosphatidic acid and it increases cell

proliferation.

Transglutaminase is internalized.

Four CDs have enzymic activity, are sometimes associated with cancers as markers

and are perhaps associated with signalling:-

CD26 has dipeptidyl peptidase-4 activity and binds adenosine deaminase

CD38 has NAAD synthase, ADP-ribosyl cyclase and cADP hydrolase activities.

CD39 has nucleotide tri- and di-phosphate hydrolase activities.

CD73 has 5’-nucleotidase activity.

Prostate-specific membrane antigen (PSMA) (also known as g[lutamate carboxy-peptidase II](http://en.wikipedia.org/wiki/Glutamate_carboxypeptidase_II), and as N-acetyl-L-aspartyl-L-glutamate peptidase I (NAALADase I), is an [enzyme](http://en.wikipedia.org/wiki/Enzyme) that in humans is encoded by the FOLH1 (folate hydrolase 1) [gene](http://en.wikipedia.org/wiki/Gene).) and is a type 2 integral membrane [glycoprotein](http://en.wikipedia.org/wiki/Glycoprotein).

**REFERENCES:-**

1.	Schantl JA, Roza M, Van Kerkhof P, Strous GJ: **The growth hormone receptor interacts with its sheddase, the tumour necrosis factor-alpha-converting enzyme (TACE)**. *Biochem J* 2004, **377**(Pt 2):379-384.

2.	Borrell-Pages M, Rojo F, Albanell J, Baselga J, Arribas J: **TACE is required for the activation of the EGFR by TGF-alpha in tumors**. *Embo J* 2003, **22**(5):1114-1124.

3.	Doroquez DB, Rebay I: **Signal integration during development: mechanisms of EGFR and Notch pathway function and cross-talk**. *Crit Rev Biochem Mol Biol* 2006, **41**(6):339-385.

4.	Edwards DR, Handsley MM, Pennington CJ: **The ADAM metalloproteinases**. *Mol Aspects Med* 2008, **29**(5):258-289.

5.	Dong J, Opresko LK, Dempsey PJ, Lauffenburger DA, Coffey RJ, Wiley HS: **Metalloprotease-mediated ligand release regulates autocrine signaling through the epidermal growth factor receptor**. *Proc Natl Acad Sci U S A* 1999, **96**(11):6235-6240.

6.	Lum L, Wong BR, Josien R, Becherer JD, Erdjument-Bromage H, Schlondorff J, Tempst P, Choi Y, Blobel CP: **Evidence for a role of a tumor necrosis factor-alpha (TNF-alpha)-converting enzyme-like protease in shedding of TRANCE, a TNF family member involved in osteoclastogenesis and dendritic cell survival**. *J Biol Chem* 1999, **274**(19):13613-13618.

7.	Ulrich LE, Koonin EV, Zhulin IB: **One-component systems dominate signal transduction in prokaryotes**. *Trends Microbiol* 2005, **13**(2):52-56.

8.	Roux A, Payne SM, Gilmore MS: **Microbial telesensing: probing the environment for friends, foes, and food**. *Cell Host Microbe* 2009, **6**(2):115-124.

9.	Stockinger A, Eger A, Wolf J, Beug H, Foisner R: **E-cadherin regulates cell growth by modulating proliferation-dependent beta-catenin transcriptional activity**. *J Cell Biol* 2001, **154**(6):1185-1196.

10.	Xu Z, Jin B: **A novel interface consisting of homologous immunoglobulin superfamily members with multiple functions**. *Cell Mol Immunol* 2010, **7**(1):11-19.

11.	Dong LL, Liu L, Ma CH, Li JS, Du C, Xu S, Han LH, Li L, Wang XW: **E-cadherin promotes proliferation of human ovarian cancer cells in vitro via activating MEK/ERK pathway**. *Acta Pharmacol Sin* 2012, **33**(6):817-822.

12.	Kuijper S, Turner CJ, Adams RH: **Regulation of angiogenesis by Eph-ephrin interactions**. *Trends Cardiovasc Med* 2007, **17**(5):145-151.

13.	Klein R: **Bidirectional modulation of synaptic functions by Eph/ephrin signaling**. *Nat Neurosci* 2009, **12**(1):15-20.

14.	Beauchamp A, Debinski W: **Ephs and ephrins in cancer: ephrin-A1 signalling**. *Semin Cell Dev Biol* 2012, **23**(1):109-115.

15.	Larsen CP, Knechtle SJ, Adams A, Pearson T, Kirk AD: **A new look at blockade of T-cell costimulation: a therapeutic strategy for long-term maintenance immunosuppression**. *Am J Transplant* 2006, **6**(5 Pt 1):876-883.

16.	Lise MF, El-Husseini A: **The neuroligin and neurexin families: from structure to function at the synapse**. *Cell Mol Life Sci* 2006, **63**(16):1833-1849.

17.	Kenyon C: **A perfect vulva every time: gradients and signaling cascades in C. elegans**. *Cell* 1995, **82**(2):171-174.

18.	Strasser A, Jost PJ, Nagata S: **The many roles of FAS receptor signaling in the immune system**. *Immunity* 2009, **30**(2):180-192.

19.	Matsuo K, Irie N: **Osteoclast-osteoblast communication**. *Arch Biochem Biophys* 2008, **473**(2):201-209.

20.	Miyazawa K, Williams DA, Gotoh A, Nishimaki J, Broxmeyer HE, Toyama K: **Membrane-bound Steel factor induces more persistent tyrosine kinase activation and longer life span of c-kit gene-encoded protein than its soluble form**. *Blood* 1995, **85**(3):641-649.

21.	Konovalova A, Sogaard-Andersen L: **Close encounters: contact-dependent interactions in bacteria**. *Mol Microbiol* 2011, **81**(2):297-301.

22.	Radtke F, Schweisguth F, Pear W: **The Notch 'gospel'**. *EMBO Rep* 2005, **6**(12):1120-1125.

23.	Castel D, Mourikis P, Bartels SJ, Brinkman AB, Tajbakhsh S, Stunnenberg HG: **Dynamic binding of RBPJ is determined by Notch signaling status**. *Genes Dev* 2013, **27**(9):1059-1071.

24.	Bray SJ: **Notch signalling: a simple pathway becomes complex**. *Nat Rev Mol Cell Biol* 2006, **7**(9):678-689.

25.	Wakabayashi T, De Strooper B: **Presenilins: members of the gamma-secretase quartets, but part-time soloists too**. *Physiology (Bethesda)* 2008, **23**:194-204.

26.	Fluhrer R, Haass C: **Signal peptide peptidases and gamma-secretase: cousins of the same protease family?** *Neurodegener Dis* 2007, **4**(2-3):112-116.

27.	Vidal GA, Naresh A, Marrero L, Jones FE: **Presenilin-dependent gamma-secretase processing regulates multiple ERBB4/HER4 activities**. *J Biol Chem* 2005, **280**(20):19777-19783.

28.	Lal M, Caplan M: **Regulated intramembrane proteolysis: signaling pathways and biological functions**. *Physiology (Bethesda)* 2011, **26**(1):34-44.

29.	Ibanez CF: **Beyond the cell surface: new mechanisms of receptor function**. *Biochem Biophys Res Commun* 2010, **396**(1):24-27.

30.	Ahrens T, Assmann V, Fieber C, Termeer C, Herrlich P, Hofmann M, Simon JC: **CD44 is the principal mediator of hyaluronic-acid-induced melanoma cell proliferation**. *J Invest Dermatol* 2001, **116**(1):93-101.

31.	Kaya G, Rodriguez I, Jorcano JL, Vassalli P, Stamenkovic I: **Selective suppression of CD44 in keratinocytes of mice bearing an antisense CD44 transgene driven by a tissue-specific promoter disrupts hyaluronate metabolism in the skin and impairs keratinocyte proliferation**. *Genes Dev* 1997, **11**(8):996-1007.

32.	Okamoto I, Kawano Y, Murakami D, Sasayama T, Araki N, Miki T, Wong AJ, Saya H: **Proteolytic release of CD44 intracellular domain and its role in the CD44 signaling pathway**. *Journal of Cell Biology* 2001, **155**(5):755-762.

33.	Holden MT, Ram Chhabra S, de Nys R, Stead P, Bainton NJ, Hill PJ, Manefield M, Kumar N, Labatte M, England D *et al*: **Quorum-sensing cross talk: isolation and chemical characterization of cyclic dipeptides from Pseudomonas aeruginosa and other gram-negative bacteria**. *Mol Microbiol* 1999, **33**(6):1254-1266.

34.	Berkers CR, de Jong A, Ovaa H, Rodenko B: **Transpeptidation and reverse proteolysis and their consequences for immunity**. *Int J Biochem Cell Biol* 2009, **41**(1):66-71.

35.	Griffin M, Casadio R, Bergamini CM: **Transglutaminases: nature's biological glues**. *Biochem J* 2002, **368**(Pt 2):377-396.

36.	Seman M, Adriouch S, Haag F, Koch-Nolte F: **Ecto-ADP-ribosyltransferases (ARTs): emerging actors in cell communication and signaling**. *Curr Med Chem* 2004, **11**(7):857-872.

37.	Cubellis MV, Wun TC, Blasi F: **Receptor-mediated internalization and degradation of urokinase is caused by its specific inhibitor PAI-1**. *Embo J* 1990, **9**(4):1079-1085.

38.	Degryse B, Neels JG, Czekay RP, Aertgeerts K, Kamikubo Y, Loskutoff DJ: **The low density lipoprotein receptor-related protein is a motogenic receptor for plasminogen activator inhibitor-1**. *J Biol Chem* 2004, **279**(21):22595-22604.

39.	Pyke C, Kristensen P, Ralfkiaer E, Grondahl-Hansen J, Eriksen J, Blasi F, Dano K: **Urokinase-type plasminogen activator is expressed in stromal cells and its receptor in cancer cells at invasive foci in human colon adenocarcinomas**. *Am J Pathol* 1991, **138**(5):1059-1067.

40.	Blasi F: **Urokinase and urokinase receptor: a paracrine/autocrine system regulating cell migration and invasiveness**. *Bioessays* 1993, **15**(2):105-111.

41.	Montuori N, Ragno P: **Multiple activities of a multifaceted receptor: roles of cleaved and soluble uPAR**. *Front Biosci* 2009, **14**:2494-2503.

42.	Burtin P, Fondaneche MC: **Solubilization of the plasmin receptor from human carcinoma cells**. *Biochem Biophys Res Commun* 1990, **170**(2):748-754.

43.	Kolodziej SJ, Klueppelberg HU, Nolasco N, Ehses W, Strickland DK, Stoops JK: **Three-dimensional structure of the human plasmin alpha2-macroglobulin complex**. *J Struct Biol* 1998, **123**(2):124-133.

44.	Coughlin SR: **How the protease thrombin talks to cells**. *Proc Natl Acad Sci U S A* 1999, **96**(20):11023-11027.

45.	Kalafatis M, Mann KG: **Factor V: Dr. Jeckyll and Mr. Hyde**. *Adv Exp Med Biol* 2001, **489**:31-43.

46.	Strickland DK, Kounnas MZ: **Mechanisms of Cellular Uptake of Thrombin-Antithrombin II Complexes Role of the Low-Density Lipoprotein Receptor-Related Protein as a Serpin-Enzyme Complex Receptor**. *Trends Cardiovasc Med* 1997, **7**(1):9-16.

47.	Stefansson S, Lawrence DA, Argraves WS: **Plasminogen activator inhibitor-1 and vitronectin promote the cellular clearance of thrombin by low density lipoprotein receptor-related proteins 1 and 2**. *J Biol Chem* 1996, **271**(14):8215-8220.

48.	Martorell L, Martinez-Gonzalez J, Rodriguez C, Gentile M, Calvayrac O, Badimon L: **Thrombin and protease-activated receptors (PARs) in atherothrombosis**. *Thromb Haemost* 2008, **99**(2):305-315.

49.	Ducros E, Mirshahi SS, Faussat AM, Mirshahi P, Dimicoli S, Tang R, Pardo J, Ibrahim J, Marie JP, Therwath A *et al*: **Soluble endothelial protein C receptor (sEPCR) is likely a biomarker of cancer-associated hypercoagulability in human hematologic malignancies**. *Cancer Med* 2012, **1**(2):261-267.

50.	Low DA, Baker JB, Koonce WC, Cunningham DD: **Released protease-nexin regulates cellular binding, internalization, and degradation of serine proteases**. *Proc Natl Acad Sci U S A* 1981, **78**(4):2340-2344.

51.	Nguyen G: **Renin, (pro)renin and receptor: an update**. *Clin Sci (Lond)* 2011, **120**(5):169-178.

52.	Jan Danser AH, Saris JJ: **Prorenin uptake in the heart: a prerequisite for local angiotensin generation?** *J Mol Cell Cardiol* 2002, **34**(11):1463-1472.

53.	Danser AH, Deinum J: **Renin, prorenin and the putative (pro)renin receptor**. *Hypertension* 2005, **46**(5):1069-1076.

54.	Wheatley SP, Wang Y: **Midzone microtubule bundles are continuously required for cytokinesis in cultured epithelial cells**. *J Cell Biol* 1996, **135**(4):981-989.

55.	Therond PP, Limbourg Bouchon B, Gallet A, Dussilol F, Pietri T, van den Heuvel M, Tricoire H: **Differential requirements of the fused kinase for hedgehog signalling in the Drosophila embryo**. *Development* 1999, **126**(18):4039-4051.

56.	Bhathena SJ, Oie HK, Gazdar AF, Voyles NR, Wilkins SD, Recant L: **Insulin, glucagon, and somatostatin receptors on cultured cells and clones from rat islet cell tumor**. *Diabetes* 1982, **31**(6 Pt 1):521-531.

57.	Kieffer TJ, Heller RS, Unson CG, Weir GC, Habener JF: **Distribution of glucagon receptors on hormone-specific endocrine cells of rat pancreatic islets**. *Endocrinology* 1996, **137**(11):5119-5125.

58.	Lucero HA, Kintsurashvili E, Marketou ME, Gavras H: **Cell signaling, internalization, and nuclear localization of the angiotensin converting enzyme in smooth muscle and endothelial cells**. *J Biol Chem* 2009, **285**(8):5555-5568.

59.	Williams T, Kole R: **Analysis of prostate-specific membrane antigen splice variants in LNCap cells**. *Oligonucleotides* 2006, **16**(2):186-195.
