# Supplementary material for: A model to explain specific cellular communications and cellular harmony:- a hypothesis of coupled cells and interactive coupling molecules
Source: Theor Biol Med Model. 2014 Sep 14;11:40. doi: 10.1186/1742-4682-11-40 (PMC4237941; doi:10.1186/1742-4682-11-40)
Supplement: Additional file 1 — 1. “Terminology”. 2. “Change in Cellular Characteristics”. 3. Note 1 “The Roles of SCD versus ACD”. Note 2. “Categories of SCD and ACD”. Note 3 “Further Examples of these AsCD Categories”. Note 4 “Change of differentiation type.”. 4. “The Effects of Growth Factors and other Bioactive Molecules 5. Note 1, “Additional Information on the Definition of a Trefone”. Note 2 “Clarification on Ligand and Binding Protein Interaction”. Note 3 “Cell Couplets – AsCD and the a-Cell and i-Cell”. Note 4 “Drosophila – Potential Trefones and ACD”. 6. “Calculated Levels of Trefones and TCC, with Cellular Actions”. 7. “Receptors for the Six IGFBPs and Cellular Effects”. 8. Note 1 “Extracellular Binding Proteins/ Soluble Receptors”. Note 2 “Cell Receptors for Binding Proteins/Soluble Receptors”. 9. Note 1. “Further Evidence for a Receptor for TCC”. Note 2 “Problems with Cross-linking Experiments”. 10. “Evidence for Binary Receptors aside from the IGF System”. 11. “Evidence for Two Receptors for a Particular Trefone, outside the IGF System”. 12. Note 1 “Mechanisms of Nuclear Localization”. Note 2 “NL of IGFBPs”. Note 3 “NL of Potential Trefones other than the IGF System”. 13. “Evidence Supporting the Existence of a-Cells and i-Cells”. 14. “Candidates with mainly a-Cell-Type or i-Cell-Type Characteristics”. 15. “Interacting Cells and Trefones not associated with the IGF System”. 16. “Heterogeneity/Variability of Cells in Culture.” 17. “Cell lines are not Typical of Normal Cells”. 18. “Cell Receptors and Cell Markers”. 19. “Defined Couplet Cells for Insulin and Glucagon; Gastrin and Histamine”. 20. “Potential Couplet Trefones”. 21. “Further Examples of Potential Trefone and Cell Couplets”. 22. “Examples of Cellular Regulation by Complexes”. 23. “Proteolytic enzymes and their inhibitors”. 24. “Expanded Definition of ‘Trefone’ and Classes of Couplet Cell Interactions”. 25. Note 1 “Extended Trefone Couplets”. Note 2 “Singlet Cells”. 26. “Background of Cancer Research”. [file 1742-4682-11-40-S1.zip › 3484671921331230_add1/3484671921331230_add25.doc]

**Additional File** ***Twenty-Five***

**(c) Extensions of the Model (i) Couplet Progeny and Secondary Trefone Couplets**

***Note 1 “Extended Trefone Couplets”***

*The purpose of this Note is to extend the idea of Trefone Couplets to understand the control of lineages of cells.*

What is a secondary couplet? In the example of an AsCD where an “original” cell (^o^C) produces ^a^C_o_ and ^i^C_o_, these two progeny will be interconnected by primary Trefones ^a^T_o_ and ^i^T_o_ produced by ^a^C_o_ and ^i^C_o_ respectively. If some ^a^C_o_ cells undergo further AsCD to produce ^a^C_oa_ and ^i^C_oa_ (where the subscript indicates the cell lineage), these cells would be interconnected by a new pair of primary Trefones, ^a^T_oa_ and ^i^T_oa._ However, both of these cells would still produce ^a^T_o_ and be stimulated by ^a^T_i_, these now being secondary Trefones for these cells. Perhaps each offspring cell would produce half that of the parent cell and with subsequent CD’s, the production would be proportionately reduced and eventually become low and constitutive. Residual ^a^C_o_ cells, not undergoing AsCD, could also contribute here. For ^a^C_oa_ and ^i^C_oa_, each primary Trefone could be their respective Competence Factor and the secondary Trefone (^a^T_i_) could be their Progression Factor for subsequent cell division. The “original” cell ^o^C could be a stem cell so as to start two cell lineages or could be a more differentiated cell to produce a branch of the original lineage.

The progeny from asymmetric cell division of a couplet of cells is shown in Figure 1, along with the controlling couplet Trefones. However, not all ^a^C_o_ and ^i^C_o_ cells may have divided asymmetrically as shown; some may have divided symmetrically and retained just the primary Trefones, although all cells will have inherited some historical Trefones and requisite receptors.

**FIGURE 1.** **Primary and Secondary Trefones.** The primary Trefone couplet (^a^T_o_ and ^i^T_o_) controls those cells that remain as ^a^C_o_ and ^i^C_o_ . The control of the cell division when at the second generation of cells (eg. ^a^C_oa_) is primarily associated with Trefone ^i^T_oa_ from cell ^i^C_oa_, but it is also residually controlled by the primary ^i^T_o_ which will continue to be produced by cells ^a^C_oi_ and ^i^C_oi_. In some cell lineages, there would be tertiary, quaternary etc Trefone couplets.

In addition to secondary, tertiary etc Trefone couplets, there may be cross-linking Trefones. A cross-linking Trefone would be a common Trefone (eg. an a-Trefone) shared between i-Cells of different cell lines but each cell type would have a distinctive couplet i-Trefone produced by the different i-Cells. An example would be IGF-I produced as a common Trefone by different a-Cells but with certain i-Cells secreting IGFBP-2, other specific i-Cells producing IGFBP-3, where each cell line has a specific TCC receptor (ie. for IGF-I:IGFBP-2 or IGF-I:IGFBP-3).

Note that the specificity of the action of an IGFBPn on a particular cell is explained by the specificity of the cell’s receptor for IGFBPn and that of the receptor for the complex IGF:IGFBPn – the TCC. The receptor itself for TCC does not have to be absolutely specific for the complex because it is the subsequent intracellular “reception” of the TCC and interpretation of the Trefone level that is critical.

***Note 2 “Singlet Cells”***

*The purpose of this Note is to explain the formation and role of cells that are not coupled.*

A completely self-sufficient organism would require nothing from the environment. There would be no need for oxygen or nutrients, and the “division of labour” rule would require specific cells to produce oxygen and glucose etc. These cells would have specific couplet cells to balance the use of these substances with their production. In our world, this self-sufficiency is not energetically possible and cells require molecules derived from the environment. A human organism requires external nutrients for energy (eg. glucose), essential amino acids and essential fatty acids for anabolic purposes and vitamins for general metabolic reactions. Generally, these substances are necessary for, say, cell division, but they themselves are not growth factors (nor Trefones). The organism then has no cells designed to cope with changes to these essential environmental substances but overcomes potential shortfalls by reutilisation or storage.

An example:- If cells divide by AsCD to share labour tasks, the o-Cell (which has a prescribed “task” of producing metabolites “A” and “B” for itself and for its own couplet cell) may divide to produce an a-Cell which will synthesise metabolite “A” that is essential for the i-Cell (the i-Cell will produce metabolite “B” for the a-Cell). If, however, the metabolite “A” becomes freely available from the environment as a permanent food, then the a-Cell becomes redundant and apoptosis could produce a singlet i-Cell. This singlet cell would still be required to perform the “task” of producing metabolite “B” for the original o-Cell and couplet. This change to a singlet cell could become hardwired into genetic programs, epigenetically, so that significant diet changes may affect general metabolism.

A singlet cell could be single-purposed and terminally-differentiated (TD) produced by AsCD. (Refer to **Additional File Three*, Note 2 “Categories of Symmetric and Asymmetrical Cell Divisions”* Figure 1)** A progenitor cell could form two differentiated cells by SCD and each cell could form a TD cells via another SCD. For example, in the red blood cell lineage, the C/D would be an erythroblast which then accumulates massively by SCD {Type (i)}, and then it produces TD cells (reticulocytes) via CD {Type (iii)}. Reticulocytes change into the TD erythrocytes without cell division.

The differentiated cell (C/D) could either be a coupled cell and would retain the couplet Trefones of the progenitor or it could be a transient singlet cell that immediately underwent AsCD {Type (iii)}. The erythroblast cell would be a coupled cell and would retain the couplet Trefones of the progenitor. However, the reticulocytes would be singlet cells with no controlling Trefones. This singlet cell would be programmed like a single-purpose robot designed to produce the end-product cell, with receptors only for requisite differentiation factors but oblivious to other signalling molecules eg. Trefones. A singlet cell could also be derived from AsCD {Type (ii), (iv) or (vi)} where the other progeny was the regenerated parent, a differentiated cell or a cell which apoptosed, respectively. Such apoptosis is observed in *C. elegans* for example, where exactly 131 defined cells are “lost” by apoptosis and fusion during ontogenesis. In this case, the programmed cell death could have occurred because the cell was no longer needed as its function under the “division of labour” rule could have been superfluous and replaced by another resource eg. the environment. A substance from the environment, perhaps derived from a “permanently” symbiotic cell of another organism, could act as a Trefone thereby eliminating the need for the cell.

It is noted that AsCD by B (i) in **Figure 1 (Additional File Three*, Note 2 “Categories of Symmetric and Asymmetrical Cell Divisions”)***, is not a preferred option in the CTC model. This AsCD is useful as a semi-conservative CD in that the original cell is reformed but it is not favoured as the single C/D cell has no controlling partner; there is no control between the progeny, C and C/D, unless C/D retains its previous Trefones and receptors while changing its characteristics and function. This would not be favoured because the C cell would be wedded to its original partner and to a new couplet cell trying, to do a “division of labour” with two different cells.

**REFERENCES**

1.	Sulston JE, Brenner S: **The DNA of Caenorhabditis elegans**. *Genetics* 1974, **77**(1):95-104.

2.	Hengartner MO, Horvitz HR: **The ins and outs of programmed cell death during C. elegans development**. *Philos Trans R Soc Lond B Biol Sci* 1994, **345**(1313):243-246.
