# Supplementary material for: A model to explain specific cellular communications and cellular harmony:- a hypothesis of coupled cells and interactive coupling molecules
Source: Theor Biol Med Model. 2014 Sep 14;11:40. doi: 10.1186/1742-4682-11-40 (PMC4237941; doi:10.1186/1742-4682-11-40)
Supplement: Additional file 1 — 1. “Terminology”. 2. “Change in Cellular Characteristics”. 3. Note 1 “The Roles of SCD versus ACD”. Note 2. “Categories of SCD and ACD”. Note 3 “Further Examples of these AsCD Categories”. Note 4 “Change of differentiation type.”. 4. “The Effects of Growth Factors and other Bioactive Molecules 5. Note 1, “Additional Information on the Definition of a Trefone”. Note 2 “Clarification on Ligand and Binding Protein Interaction”. Note 3 “Cell Couplets – AsCD and the a-Cell and i-Cell”. Note 4 “Drosophila – Potential Trefones and ACD”. 6. “Calculated Levels of Trefones and TCC, with Cellular Actions”. 7. “Receptors for the Six IGFBPs and Cellular Effects”. 8. Note 1 “Extracellular Binding Proteins/ Soluble Receptors”. Note 2 “Cell Receptors for Binding Proteins/Soluble Receptors”. 9. Note 1. “Further Evidence for a Receptor for TCC”. Note 2 “Problems with Cross-linking Experiments”. 10. “Evidence for Binary Receptors aside from the IGF System”. 11. “Evidence for Two Receptors for a Particular Trefone, outside the IGF System”. 12. Note 1 “Mechanisms of Nuclear Localization”. Note 2 “NL of IGFBPs”. Note 3 “NL of Potential Trefones other than the IGF System”. 13. “Evidence Supporting the Existence of a-Cells and i-Cells”. 14. “Candidates with mainly a-Cell-Type or i-Cell-Type Characteristics”. 15. “Interacting Cells and Trefones not associated with the IGF System”. 16. “Heterogeneity/Variability of Cells in Culture.” 17. “Cell lines are not Typical of Normal Cells”. 18. “Cell Receptors and Cell Markers”. 19. “Defined Couplet Cells for Insulin and Glucagon; Gastrin and Histamine”. 20. “Potential Couplet Trefones”. 21. “Further Examples of Potential Trefone and Cell Couplets”. 22. “Examples of Cellular Regulation by Complexes”. 23. “Proteolytic enzymes and their inhibitors”. 24. “Expanded Definition of ‘Trefone’ and Classes of Couplet Cell Interactions”. 25. Note 1 “Extended Trefone Couplets”. Note 2 “Singlet Cells”. 26. “Background of Cancer Research”. [file 1742-4682-11-40-S1.zip › 3484671921331230_add1/3484671921331230_add26.doc]

**Additional File *Twenty-Six:-***

**5. Applications in Medicine**

**2. Cancer**

***Background for an Understanding of Cancer Research***

*The purpose of this File is to present an overview of cancer research to justify a view that the CTC model is of relevance to an understanding of cancer and its causes. This requires a brief review of mutations and chromosomal changes whereby receptors and internalized Trefones may determine cell division. Because the CTC model is associated with AsCD and control of cell-division type, abnormalities of these may be directly associated with cancer. In particular, the focus is on the interaction of cells within a solid tumour, which may include normal and abnormal cells, where couplet Trefones may interact via abnormally coupled cells. Sections (e), (h), (i) and (k) more specifically connect the CTC Model with cancer.*

**(a) Preamble**

While there are many hallmarks of cancer, **c**ancer is especially characterized by unrestrained proliferation, with loss of organization in a group of cells. This cancerous expansion is best explained by the property that a greater percentage of cancerous progenic cells replicate compared to their normal parental cells. The other options to explain the expansion (that cancer cells replicate through the cell cycle faster or a smaller proportion die) seem less likely but may apply in some cancerous cells as nature is not restricted by human-drawn demarcation lines. However, the condition is not intrinsically caused by the excessive cell proliferation rather the cancer cells must adversely affect the function of normal cells by excessive use of nutrients, release of inappropriate signals or metabolites, perhaps thus to alter the normal balance of symmetric (SCD) and/or asymmetric cell division (AsCD) and/or differentiation.

Cancer is a disease caused by mutated genes (inherited or acquired by mitotic infidelity without repair or by environmental exposure to radiation or air-borne mutagens) and/or by genes altered by epigenetic changes (DNA methylation and histone modifications) associated with diet or drug intake. The latter epigenetic effect may be due to hypermethylation at specific regulatory sites enriched in CpG motifs (CpG islands) in the promoter regions of tumor suppressor genes (Rb, p16, p21, p53, PTEN) causing silencing. Potentially, hypomethylation of protooncogenes (c-jun, c-myc, PIK3CA and IDH1) could cause activation. Other epigenetic events (histone modification, nucleosome relocation with chromatin remodeling) may also contribute to epigenetic changes. Cancer cells could also develop from RNA modifications, RNA instability or inactivation or associated with non-coding RNA. Metabolic stress could also contribute in some way just as bacteria show a response to stressful environments by genetic reorganization and sporulation (for example).

In general, certain genes are a target in tumorigenesis and genetic changes in oncogenes, tumor suppressor genes and/or repair genes are likely, perhaps with sequential accumulation of mutations. The tumor suppressor gene *p53* is considered to be the guardian of the genome in that it has been estimated that half of all cancers have mutation in the *p53* gene.

Within a cell-line that develops cancerous cells, the mutations existing at any one time may be predisposing, the induced epimutations may accumulate with age and cancer may require more than a single critical genetic alteration to cause cancerous growth – the earlier “two-hit” theory. This “two-hit” theory has evolved into more complex models involving multiple hits, rate-limiting events, and genomic instability. The complexity of cancer can be understood if cancer is a gradual progression associated with an increasing number of mutations, each dependent on a previous genetic change and each with a relatively small but positive effect on the net growth of the predestined cells.

The cells of the body exposed to the environment (GIT, lungs, skin) avoid some mutations associated with environmental carcinogens by desquamation of exposed cells. These cells are short-lived so there is no accumulation of mutated cells. Their progenitor cells are normally distanced from exposure directly to the environment but will be affected, because of their high rate of cell division, by other genetic factors that are acquired or inherited to produce cancers (colorectal and lung cancers and melanomas).

**(b) Mutations - Fitness and Driver**

Stated another way, cancer, cells accumulate because a greater proportion of daughter cells replicate compared with normal cells and there is an increase in the ratio of cell birth to cell death ie. there is increased fitness in these cancer cells with “driver” mutations. Many tumours grow over a large number of generations where the cancer cells have only a minor advantage per generation over normal cells. About 90% of cancers arise in epithelial surfaces from cells with a high proliferative rate and many highly proliferative organs have many mutations. There are perhaps ~40–80 coding-gene alterations present in a typical solid tumor with melanomas and lung tumours containing ~200 nonsynonymous mutations per tumor. But some of these mutations are not significant in terms of adding fitness, being referred to as”passenger” mutations that are acquired with or after a “driver” mutation which does confer a selective growth advantage to the cell. There may be only specific 2-8 drivers (or 5-25) in a typical tumour, with the remainder being passengers that confer no selective growth advantage. The total driver group of genes is large; one estimate is ~ 140 for mutated genes, plus additional genes whose transcription has been altered by epigenetic mechanisms which also may cause a selective growth advantage to the cell.

In colorectal cancer (CRC), mutations increase as the lesion evolves to malignancy by acquiring, firstly, a “gatekeeping” mutation (mostly in the APC gene) which allows the cell to outgrow the normal cells and forms a small, slowly growing adenoma. Cell numbers expand when a second “driver” mutation in another gene cause clonal growth. Further “driver” mutations, together with “passenger” mutations that have been accumulating before and over the period, lead to a malignancy with full capacity to escape enclosing basement membrane and metastasize to distant organs eg. to bone, liver, lymph nodes. CRCs arise then through a multistep process with a sequential order of variable events but there are both genetic and epigenetic causes. Genetically, chromosomal instability leading to aneuploidy is a likely participant in cancer development, its role and relative importance is not clarified. While chromosomal instability associated with the traditional path to CRC with sequential deregulation of tumor suppressor genes and oncogenes (eg. APC, KRAS, DCC/SMAD4, TP53) accounts for 60% of CRCs, microsatellite instability associated with DNA mismatch repair malfunctions and the CpG island methylator phenotype (CIMP) account for the rest, with a little overlap of genes.

An alternate suggestion is that only a single event is required for the initial slow growth of a mutant cell line which changes the microenvironment (with possible hxpoxia, acidosis, unusual metabolites) and which permits evolution toward a more malignant population. The time-line for the development of cancer is then determined by these events and is dependent on the cell type and its microenvironment leading to breakdown of growth regulatory mechanisms.

Cancer may be associated with a single point mutation. The initial event may be a simple mutation (a single-base substitution, SBS, affecting a single amino acid as in sickle cell anemia or high bone mass mutations) but it must affect the cell’s ability to divide as per the directions of the intercellular signals from normal neighboring cells. A simple change of a Val to a Glu is the most common activating mutation in the Raf protein (a growth [signal transduction](http://en.wikipedia.org/wiki/Signal_transduction) [protein kinase](http://en.wikipedia.org/wiki/Protein_kinase) affecting [cell division](http://en.wikipedia.org/wiki/Cell_division) and [differentiation](http://en.wikipedia.org/wiki/Cellular_differentiation)) and this kinase is associated with various cancers eg. colorectal cancer, non-Hodgkin lymphoma, malignant melanoma, thyroid carcinoma and non-small cell lung carcinoma and adenocarcinoma of lung. Of ~ 1000 mutations found in the genes for ~ 500 protein kinases in cancer and cancer cell-lines, over 90% are single-base substitutions. Of these, over 60% are missense with another ~20% silent. Mutation with inactivation of hCDC4, a ubiquitin ligase and a tumour suppressor gene with a role in the control of cell cycle and chromosome stability, occurs in several neoplasias, including colorectal tumours, and can cause chromosomal instability**.** A single point mutation in FGF-2 with a change of Ser to Ala, drastically inhibits its mitogenic activity but not its differentiation properties. It seems instinctive to believe that aneuploidy likely derives from simple point mutations.

**(c) Receptor Signalling, Oncogenes and Trefones**

The best known example of the contribution of receptors to cancer is in breast cancer. These cancers are identified by the presence or absence of estrogen receptor (ER), progesterone receptor (PR), epidermal growth factor receptor 2 (HER2) (including the triple-negative phenotype and fibroblast growth factor receptors.

A presumption in the proposed model that follows in Section (e), is that many cancers involve initially a dysregulation of intercellular signalling. Many oncogenes are associated with the signalling process. An oncogene is a normal gene slightly mutated or associated with a translocation so as to produce a protein derived directly or indirectly from that gene with increased total activity due to increased concentration or increased inherent activity compared to normal. Gain-of-function and increased copy number are included here. Some oncogenes are associated with growth factors (Trefones) eg. *sis* is the oncogene and PDGF is the protein product PDGF. Many oncogenes are associated with growth factor receptors and most of these have [protein-tyrosine kinase activity](http://www.ncbi.nlm.nih.gov/books/n/cooper/A2886/def-item/A3295/) eg. *erb4, trk, kit, fig, met and ros;* EGF, NGF, PDGF, FGF, HGF and Insulin receptors). The oncogene may have an altered end-terminal [domain which normally binds an extracellular](http://www.ncbi.nlm.nih.gov/books/n/cooper/A2886/def-item/A3021/) [growth factor but is now constitutively active in the absence of ligand.](http://www.ncbi.nlm.nih.gov/books/n/cooper/A2886/def-item/A3104/) Activation of a receptor without a Trefone will cause disorganized growth of a cell to cause excessive stimulation of the couplet cell by excess production of the couplet Trefone.

Some oncogenes are associated with intracellular transducers (eg. [*Src*](http://en.wikipedia.org/wiki/Src_(gene))*,* [*Syk/ZAP-70*](http://en.wikipedia.org/wiki/Syk-ZAP-70)*,* [*Btk*](http://en.wikipedia.org/wiki/BTK), *abl and raf;* protein kinases:- g*sp and ras;* G-protein related) or with transcription factors (*jun*, *erb-A, FLI1 and myc).* Ras or Raf protein will activate the ERK/MAP kinase pathway which may induce other gene products to enhance the conversion of extracellular signals into changes of gene expression. The mutated *ras* or *raf* (oncogenes) will lead to constitutive MAP kinase activation while mutations in PI3-kinase will also produce cancer. These oncogenes certainly incriminate the pathway of receptor-mediated-uptake of Trefones plus translocation to the nucleus, with the dysregulation of cell division in cancers.

The possible oncogenic activations of the epidermal growth factor (EGF) signalling pathway offer a model for other systems. The EGF pathway may be exaggerated and/or dysregulated in a cancerous way by excessive ligand stimulation or excessive levels of receptors (perhaps by defective downregulation), by any mutation of the EGFR producing a constitutively active variant or by any mutation affecting downstream signalling components that normally transform the receptor message to cellular action. Alternatively, a negative regulator of signalling (eg. Sprouty 1) could be down-regulated or mutated to an inactive inhibitor to cause aberrant FGF receptor signalling in certain cancers. Tumour suppressor genes may also be silenced.

The apparent autonomous nature of the growth of cancer cells has been long recognised and has been explained by the acquired ability of these cells to act in an autocrine way This means that the cell producing a critical growth factor also has a receptor for it and responds in a vicious circle of cell cycling and stimulation in an incestuous way. As oncogenes are involved in this growth factor/receptor pathway, then the autocrine concept links oncogenes, growth factors and their intracellular transduction paths with possible causes of cancer. Consistent with this model, dysregulation of IGF-I and other potential Trefones (HGF, Wnt, TGF-alpha and -beta), their receptors or their downstream signalling components is considered to contribute to proliferation, antiapoptosis and invasive behaviour of cells in human hepatocarcinogenesis.

Epigenetic gene silencing by hypermethylation is relevant for growth factor receptors and their potential Trefones. The expression of VEGF receptor genes is influenced by opposing actions of epigenetic gene silencing and by VEGF activation of these genes. Genes for VEGF receptors (Flt-1 and KDR) have typical CpG islands in their promoter regions, and variable promoter hypermethylation of these genes occurs in primary cancer tissue from patients with stomach, colon and hepa­tocellular cancer. Promoter hypermethylation correlated with decreasing expression of the respective genes in cancer cell lines and the conclusion was that VEGFR gene expression in actual tissue cancer cells is mainly influenced by epigenetic promoter modifications of the gene, and not by VEGF activation. VEGF itself is not affected by methylation.

The FGF receptor gene can also be affected; DNA methyltransferase 3B has been demonstrated to mediate gene silencing and it represses transcription of the FGFR3 via the human polycomb protein, hPc2.

**(d) Chromosomal Changes**

Cancer cells often have chromosomal changes eg. aneuploidy and/or polyploidy. Many theories of cancer presume that these more major changes are the result of the cancerous growth, arising from an initial gene mutation. To the contrary, some suggest that aneuploidy rather than gene mutation may be the initial event causing cancer.

# Aside from simple genomic alterations including single-base substitutions (SBS), missense or nonsense mutation, small insertions, deletions or amplifications, the genetic changes in cancer may involve basic chromosomal rearrangements - inversion (flip-flop of two segments of a single chromosome), translocation (reciprocal rearrangement of segments [**between two**](http://www.medpath.info/MainContent/Neoplasia/Neoplasia_08.html) chromosomes), deletion of a segment of a chromosome. These, along with “chromosomal instability” are likely to be a cause or an enabling characteristic of a cancerous cell growth. Two oncogenes (PIK3CA and IDH1) are often missense mutations while two tumour suppressor genes (RB1 and VHL) are more likely truncating mutations than missense.

#  Major chromosomal-number disturbances eg. gain or loss of chromosomes (aneuploidy) and/or polyploidy, are commonly observed in late cancers and are more likely to be the result of the cancer growth. However, the latter is observed naturally in the giant cells of the trophoblast, megakaryocytes, multinucleated hepatocytes and in the fusion of cells to form myotubes, osteoclasts and syncytiotrophoblasts and therefore need not be an abnormality in spite of apparent chromosomal disorganisation (ACD). One might guess that this ACD observed in many cancer cells is either an indicator of chromosomal programming meltdown in the cell or it is the cell’s deliberate response to this aberrant cancerous life as it seeks a return to normality by testing its repertoire of possible genetic tricks. The ACD might foster new options just as a trapped animal, bird or fish might thrash and jump at random to test for an escape route from an abnormal state. While there are such advantages, there are many disadvantages of being polyploid. However, the cancer cell, in spite of the ACD, still has an organised metabolism (although somewhat abnormal, skewed toward a more glutamine-stimulated anabolic state with high lactate formation and secretion, plus high 2-hydroxyglutarate formation) and undergoes cell division, albeit more often than normal.

Cells can eliminate excess chromatin but it is not known whether this diminution of chromatin is simply to remove waste or if it is specific so as to select the fate of the cell. Either the autophagy for the elimination of micronuclei or of whole nuclei from polyploid cells will provide tumour cells with the opportunity to repair DNA, sort and resort chromatin, reduce DNA content, and enhance survival.

The ACD is well known *in vitro* in human cancer cell lines (See **Additional File** ***17***) where the cells are forcing themselves to reproduce continuously in an abnormal environment. Polyploidy is also observed before neosis and could be associated with stemness . Polyploidy can be transient in giant cells derived from senescent, multi-passaged embryonic cells with the subsequent formation of near-diploid, genome-reduced cells. It has also been suggested that polyploidy may be a part of a cancer cell life cycle with a “mitotic catastrophe” producing a potentially reversible polyploid state.

**(e) The Proposed Model**

The current discussion is not focused on cancer stem cells, a mutator phenotype, field cancerisation or a Darwinian perspective.

The focus is on the interactions of growth factors with their receptors which often induce kinase activities, activate signalling pathways to alter gene expression patterns and induce mitogenesis. These, if deregulated, are related to cancer.

A stem cell can divide by AsCD to form branches for a cell lineage and the progeny can divide by SCD to enhance their numbers when required. From these intermediate progenitor cells, cell division would be controlled by growth factors and differentiation factors with all of the possibilities outlined in Figure 5 of the main text. In a cancer, the normal homeostasis and balance of cells is disturbed such that some of these possibilities are favored or prevented.

AsCD certainly occurs in leukemic promyelocytic cells (HL-60) where “large” cells produce “small” cells which account for 8-9% of the total cells; it also occurs in a small population of lung cancer cells and is further discussed in Section (g).

In the context of the proposed generic CTC model, the earliest cancer cells would be derived from one or a pair of coupled calls. A parent cell of the now cancer cells may have undergone an abnormal asymmetric cell division (AsCD) to produce the abnormally responsive cells. An AsCD could produce one cell (eg. an a-Cell) with a slight propensity to over- or under-react to stimulation compared to its coupled cell (i-Cell), perhaps due to a mutation, epigenetic change or to an unequal division of parental resources beyond the “normal” asymmetric assignment. Over many lifetimes of subsequent SCDs, these a-Cells may have gained a “fitness” advantage over the normally matched i-Cells and consequently more a-Cells are produced. An alternate would be if the a-Cells are not as efficient as i-Cells in producing its Trefone (^a^T) in response to the other Trefone (^i^T) then more a-Cells would be required. These a-Cells may have a mutation or deficiency in a Trefone, appropriate receptors or in their signalling infrastructure to be able to respond in the normal way (eg. to undergo a normal cell-lineage required AsCD) so that their response will differ from that expected. In the current model of coupled cells and Trefones, the responses of the coupled cells to varying concentrations of Trefones are elaborated in Table S1 (within SI 6). This Table applies only to sets of normal cells in a tissue or organism, which are each coupled by a normal response to a Trefone.

The CTC model has two distinct responses to a Trefone signal. The Trefone signal from one cell will transfer two messages for the other cell (i) to increase its *metabolism* and to stimulate production of the other Trefone via the normal kinase pathways and (ii) if necessary, to stimulate cell division or induce some other “*whole-of-cell, life/death*” action via nuclear localization of a Trefone. Some mutation or epigenetic change in the Trefone, Trefone receptors, proteins of internalization, transporters to the nucleus or in the transcription factor(s) binding the Trefone, would initiate an abnormal cellular response to stimulate progress to a cancer cell.

The primary cause of cancer cell formation in this model is the errant second effect but there may also be a *metabolic* passenger effects. The altered expression of a Trefone or protein involved in receptor-mediated uptake, intracellular processing or in its subsequent nuclear presence could cause a reprogramming of the cells chromatin to allow an exposure of some DNA not normally available for expression. The altered Trefone may bind DNA abnormally after internalization but it may first activate the receptor and its kinases quite normally with no metabolic change initially. The nuclear effect could cause the cell to change to become a cancer cell via a symmetric or asymmetric cell division with a change in metabolic emphasis. The cancer cells must have new receptors and must produce new Trefones, and consequently metabolic changes will also occur and cause change to the extracellular matrix. Some secreted metabolites (chemokines) could attract mobile and migratory cells while potentially toxic metabolites could attract inflammatory-response cells.

Some examples of cells that have the overexpressed receptor etc were discussed in **Additional File** ***14***, ***Note 9***, including choriocarcinoma cells, Schwannoma cells and glioblastoma cells. Hybrid cells between a-Cells and i-Cells could explain the cancer scenario in those cells. Receptors other than those associated with the IGF system

A cancer is then presumed to be initially a dysregulation of intercellular signalling as described for liver cancer, associated with Trefone, and associated receptors and infrastructure. It is a dysregulation the Trefone Component Software (TCS) which is integral to the Cell Operating System (COS). This TCS provides the essential intercellular communications to the COS at that time in the cell’s journey which leads to an end-stage within the lineage in that particular multicellular organism/individual. The idea is that an aberrant type or level of the Trefones themselves, their receptors and/or of the components of their intracellular infrastructure, if not corrected, would lead to a serious program error due to [internal self-inconsistency](http://en.wikipedia.org/w/index.php?title=Internal_self-consistency&action=edit&redlink=1). A subsequent program crash could lead to the chromosomal abnormalities, cell division aberrations, aneuploidy or other [chromosomal](http://www.ncbi.nlm.nih.gov/books/n/iga/A4529/def-item/A4659/) misdivisions eg. polyploidy, any of which may be critical part of *in vivo* cancer. This dysregulation with chromosomal aberrations would induce a new state with retention of only part of the normal cassette of controls from the specific intercellular signalling associated with the original cell. This new state of the cell, involving a default program specific to cancer cell types, is abnormal but controlled or restrained by the same mechanisms as before.

By such a definition of cancer, experiments using cancerous cell lines *in vitro* are then expected to produce observations and effects which are only partially normal. This treatise then seeks to extract the normal from the aberrant to produce a picture of the normal TCS and COS.

Experiments with overexpressed receptors or other signalling components may also need care in interpretation. Each type of cell in an organism is exquisitely designed and programmed. It is maintained in a controlled environment (except the high-turnover cells in the GIT etc) and programmed to perform certain tasks within its environment and to respond in certain ways to specified signals, dependent on the input from receptors for the special Trefone and cell couplets. The interpretation of experiments that manipulate a cell type by the targeted overexpression of a receptor would need to be done with caution. The overexpression of a receptor foreign to that cell type would destroy the cell’s normal function in its association with a couplets cell and would cause a reprogramming of the cell to accommodate the new input signal from the overexpressed receptor.

By definition, differentiation will change many of the cell’s surface proteins. An abnormal differentiation within a cell lineage could produce a cell with receptors that respond to signalling molecules from another lineage and these cells could flourish as a cancer cell. An abnormal differentiation could produce a cell with a change in adhesion proteins such that there is no longer contact inhibition and such cells could flourish as a cancer in another part of the body.

**(f) Intratumoral heterogeneity: many different cells form an organized solid tumour.** The cancer cells of the proposed model contain new surface receptors to respond to specific Trefones. A particular Trefone required by a cancer cell may be secreted by a couplet cancer cell derived from AsCD or the Trefone may be secreted from a normal cell which itself has a couplet cell. There may initially be one or two cancer cell types and others if AsCD continues. In addition, there will be a mix of other cells in tumours.

A solid cancer is known to consist of several types of cells. Some are cancer cells and some are non-malignant cells which are, or are derived from, normal cells, organized in a complex microenvironment as a somewhat self-sustaining organ or tissue. The normal type cells would include cells of the vasculature (endothelial cells, pericytes, smooth muscle cells), fibroblasts and those related to inflammation and/or infection (lymphocytes, macrophages, neutrophils, mast cells). Tumour-associated macrophages and neutrophils may appear in various activation states and may be tumour-promoting (stimulating the malignant properties of angiogenesis, migration and invasion) or tumour-inhibiting (suppressing the immune response to cancer cells). Different subpopulations of macrophages may participate at different phases of growth and metastasis.

Some of the cell types are derived from the bone marrow myeloid lineage. Myeloid-derived suppressor cells (MDSC) are a heterogeneous subset of immature and mature myeloid cells with immunosuppressive properties, which expand in the tumor host. MDSCs include direct progenitors of the differentiated myeloid cells – of macrophages, dendritic cells and granulocytes. Endothelial cells and cancer-associated fibroblasts (two types) also contribute to the growth of cancers by their production of growth factors, chemokines and extracellular matrix. Pericytes associated with angiogenesis are also present – these are a specialized multipotent mesenchymal cell type, related to smooth muscle cells, with projections that wrap around the endothelial tubing of blood vessels.

This grouping of cells would be organized within its own controlled environment, having formed its own extracellular proteins and matrix with a unique array of interrelating growth factors and cytokines. For example, breast cancer cell proliferation is stimulated by the product of shedding of syndecan-1 (soluble Sdc1) from stromal fibroblasts. Breast carcinoma cells induce expression of syndecan in mammary fibroblasts; reciprocally the fibroblasts stimulate the carcinoma cell growth by soluble Sdc1 derived from syndecans. Proteolytic conversion of Sdc1 from the membrane-bound co-receptor into a soluble molecule marks a switch from a proliferative to an invasive phenotype.

Indeed, within a tumor there is this mixture of cells; intratumoral heterogeneity of cells is observed rather than a homogeneous monoclonal aggregation of cells derived from a linear progression of cellular differentiation. As a tumor evolves, identical cells apparently “split off” and develop new mutations or other errors, like a tree growing many branches. This means that two parts of the same tumor, as well as the metastases that form when cancer spreads, can look very different genetically. But this recent branched model is similar to a model of >35 years ago which, for solid tumours, effectively proposed asymmetric cell divisions (with some progeny being non-viable perhaps because of immune response) in a model of “clonal evolution in neoplasia”. Asymmetric cell division is then at the heart of both of these models!

Neoplasms (eg. non-small cell lung cancer), thought to be somewhat homogeneous, have cells with a specific mutation that may account for only 5-10% of the total cells. For instance, tumors bearing ALK alterations make up about 4% of non-small cell lung cancers, and tumors bearing epidermal growth factor receptor (EGFR) mutations, 5-10%. These contain many subpopulations of cells as would be derived from branching ie. from asymmetric cell divisions.

This intra-tumour heterogeneity includes cells with mutations or with epigenetically altered genes, which are distinct from the normal cells some of which are derived from the organism’s immune response. Ostensibly-normal cells contribute to the "tumor micro-environment”; for example, adipocytes via lipolysis promote ovarian cancer growth *in vitro* and *in vivo*. Recruited cells of the tumor-associated stroma actively participate in tumorigenesis and the biology of a tumor reflects the heterogeneity of cells.

If tumours are a mosaic of genetically different cells, then the whole mass will also be metabolically heterogeneous. Traditionally, cancer cells are considered to have less dependent on mitochondrial function and to rely on aerobic glycolysis, termed the Warburg Effect. However, the heterogeneity of cells will mean heterogeneity of metabolism. Some cancer subtypes will have enhanced oxidative phosphorylation in mitochondria whereas others will have a high level of glycolysis with lactate production.

**(g) Asymmetric Cell Division (AsCD) and Cancer**

AsCD and SCD are features of normal development and part of everyday replacement of functional cells derived from stem cells. They are also part of cancer development. Mutations that perturb cell polarity that is associated with AsCD can cause neoplastic overproliferation. As an example, *Drosophila* neuroblasts normally undergo AsCD but with a double mutation in *Lgl* (Lethal giant larvae, a tumour suppressor protein) and *pins* (Partner of Inscuteable - a cell polarity determinant for AsCD) now divides symmetrically and fills the brain with neuroblasts instead of neurons. Mutations of other genes involved in polarity {Dlg (Discs large), Scrib (Scribble) - tumour suppressor genes in *Drosophila*} also cause neoplastic overgrowth of tissues. Mutations in cell fate determinants (Numb or Prospero) also cause tumours. Overall then, studies in flies suggest that the formation of tumours results from perturbation of the control by growth factor signalling and of asymmetric cell division.

The extent of AsCD may vary in cancer. Leukemia stem cells tend to renew by SCD but healthy counterparts predominantly use AsCD while the majority of glioma [stem cells](http://www.ncbi.nlm.nih.gov/pubmed/21881602) proliferated by SCD in expansive growth conditions but by AsCD in the absence of growth factors. Another level of asymmetry applies to the asymmetric division of template DNA - the “immortal DNA strand hypothesis” . Here, the older template DNA strand is retained specificially by the progeny stem cell in AsCD with self-renewal (SR), while the newly synthesised strand is in the differentiated cell. This a observed in some cells especially some stem cells. In some lung cancer cell lines and primary tumor cell cultures, a small population of cells asymmetrically divided their template DNA. The extent depends on cell:cell contact, hypoxia and serum deprivation.

**(h) The Trefone Model Revisited**

This section develops the model that cancer cells are so because of mutations affecting either an a-Cell or an i-Cell which then changes the level or effectiveness of synthesis and secretion of both Trefones or are so because of mutations affecting the receptors and other proteins involved in the transfer of the message from either one Trefone or both Trefones to the nucleus and subsequent response.

This Trefone model is consistent with the observed presence of a mix of cancer cells formed by AsCD. SCD, of course, could produce two cancer cells (2x CC1) from a parent cell (C) with a mutated or otherwise altered gene. Then these CC1 cells could initiate formation of a cancer-cell aggregation by some CC1 cells undergoing SCDs to produce more CC1 cells and other CC1 cells producing CC2 cells by SCD due to exposure to different microenvironments. However, AsCD offers more options than SCD. **Additional File** ***Three***, ***Note 2,* Figure 1** contains six possible AsCDs which could produce a cancer cell altered in some way by an asymmetric division of the parent cell’s resources including a mutated gene. Four, {(iii) - (vi)}, have cell progeny with immediate division of labour (DOL) possible, while the first two {(i), (ii)} with a parent cell conserved, could have a DOL if the PC is induced to change by molecules from the other differentiated progeny. Interestingly, within the type (iii) AsCD which produces two differentiated cells, each different from the other (Dual LCD, where LCD = Lineage Commitment to Differentiation), there are further options. One cell progeny may be cancerous, the other of a normal type. Or the cell progeny may both be of cancer types or of normal types but each of a pair different from each other. (The latter example could not be an AsCD initiating a cancer cell line but may occur down the family line.) In any case, with the two types there is an expectation of a division of labour between them. The division may mean that one type of cell from AsCD/LCD eg. myofibroblasts, would focus on aerobic glycolysis (the Warburg effect) and secrete lactate (and pyruvate, ketone bodies), while the coupled cell that would focus on lactate uptake and Oxidative Phosphorylation and would contain more Mitochondria (OPM). This latter OPM has been termed the "Reverse Warburg Effect" and is part of a hypothesis wherein cancer cells induce or corrupt the stromal fibroblasts to differentiate to myofibroblasts which then secrete lactate and pyruvate. These fuels are taken up by the epithelial cancer cells for ATP generation via oxidative phosphorylation. (This hypothesis developed into the paradigm "The Autophagic Tumor Stroma Model of Cancer Cell Metabolism".) The monocarboxylate transporter MCT1 can transport lactate into and out of tumor cells and MCT4, which causes efflux of lactate from cells, is expressed in cancer-associated fibroblasts. Epithelial breast cancer cells undergo anabolic reprogramming to focus on mitochondrial biogenesis thus to become predatory metabolic "parasites" compared with adjacent stromal tissue. The coupled cells of the CTC model with division of labour fits with these observations.

Perhaps the secreted ATP from the coupled cells with OPM completes a reciprocal paracrine effect, as ATP is recognized as a signalling molecule and extracellular ATP is a known growth factor eg. for brain and for vascular smooth muscle cells while the ATP cell receptor (P2 receptor) also is present in a whole variety of cancers.

Human mesenchymal stem cells are bone-marrow derived stromal cells and are a source of stromal carcinoma-associated fibroblasts. Both of these cell types utilize lactate possibly produced by hypoxic- tumor cells.

While division of labour (DOL) is a particular advantage of AsCD, AsCD and ACD are not mutually exclusive – on the contrary SCD will follow AsCD to produce a community of related cancer cells and normal cells **(Figure 1).**

**FIGURE 1. The formation of cancer cells and normal cells with asymmetric cell division (AsCD) and symmetric cell division (SCD).**

*The Figure illustrate normal cells (C) and derived cancer cells (CC). Note that the cancer cells of different types (CC_0_ – CC_3_) could also undergo AsCD to form one normal-type cell. The CC_4_ is terminally differentiated in this diagram.*

This cancer community would have many options and this developmental plasticity would be important in adapting to microenvironmental changes. Regenerative plasticity would also be critical in the face of adversity (eg. selective chemotherapy) where the death of some cancer cells would determine more AsCD and SCD from the remaining cells, and some cell divisions would produce dedifferentiation to replace absent coupled cells. Specifically, if a cancer is from an a-Cell and chemotherapy destroys the a-Cells then the i-Cells can reform a-Cells by AsCD/transdifferentiation/dedifferentiation to regenerate the community. A larger cancer community would be resilient in that selective cell deaths would have less impact on a multicellular, autonomous community.

The Delta/Notch signalling couplet in flies is one example of the connection of cell-cell signalling and tumours. The two progeny of an AsCD can communicate with each other via signals and the formation of different cells relies on the differential activation of a signalling pathway in the two cells. Tumourous cell amplification of just one of the couplet progeny could result from failure in the regulation of the signalling activity.

There may be growth assistance to cancer cells not only by metabolites but also by Trefones secreted from normal cells in a paracrine fashion. Insulin-like growth factors are potent mitogens for breast tumour epithelial cells. Since no synthesis of IGF-I has been demonstrated in breast (or colon) cancer cell lines, IGF-I stimulation of breast carcinoma growth may function in a paracrine fashion because IGF-I mRNA is expressed in the stromal cells (but not by normal or malignant epithelial cells). However, for the IGF-I receptor (IGF-IR), one breast cancer cell line (MCF-7) has 13-fold higher amounts of nuclear IGF-1R than the small amount in breast epithelial cells although expression of IGF-1R was only ~4 higher in MCF-7. On the other hand, IGF-II may be more important than IGF-I in malignant breast lesions.

**(i) Potential Trefones in Cancer**

Within a solid cancer, endothelial cells stimulate proliferation and differentiation of pericyte precursors by secreting PDGF-B, VEGF and TGF-β. Angiopoietins are also produced, in particular Ang-1, by pericytes, which acts on receptors (Tie-2) on endothelial cells, indicating one example of intercellular dependence. While a mutation might instigate a departure from normal homeostasis and cause the initiation of a tumour, normal growth factors and signalling may regulate subsequent events. IGF-I and EGF may regulate clonal expansion, TGF-beta, HGF, FGF and EGF may support tissue invasion while VEGF, FGF and HB-EGF may stimulate angiogenesis. One interpretation of tumour growth is that cancer cells can “instruct” surrounding tissues to undergo changes that promote malignancy. A variety of tumor-derived factors contribute to the emergence of complex local and regional immunosuppressive networks, including IL-10, VEGF and TGF-beta.

The cancer cells may be self-sustaining by an autocrine mode that includes a mutated, constitutively active signalling pathway or they may sustain a continued proliferation via growth factors produced by themselves or by neighboring cells, in a reciprocal, paracrine way. Besides the aforementioned growth regulators, CSF-1, IL-1beta, stromal cell-derived factor 1 and Hegdehog are implicated in reciprocal heterotypic signalling interactions^1^. HGF also signals between mammary fibroblasts and epithelial cells and both HGF and TGF-beta have complex functional roles for signalling in tumor-stromal interactions during mammary tumor-cell scattering and invasion.

Some other normal cell types may also support the growth of the cancer cells. Bone- marrow-derived mesenchymal stem cells secrete the cytokine CCL5/RANTES which causes cancer cells to increase their metastatic potency. In turn, cancer cells stimulate the secretion of the cytokine in a paracrine way. Other tumor-derived factors may be added to the already complex local environment - prostaglandin E(2), IL-10, phosphatidylserine, soluble Fas and soluble Fas ligand.

Perhaps an abnormal concentration of a protein rather than a actual mutation can also contribute to the formation of cancer cells. If a normal cell requires at least two events to gain the property of a cancer cell, then, following one mutation, the second event may be a spontaneous and random ie. a stochastically derived “abnormality”. For any property of apparently identical cells, there would a variation in that property described by perhaps a Gaussian distribution with a mean value = X and a standard deviation = σ. A property of a specific progenic cell, for example a property derived from the concentration of intracellular substances A and B, could vary considerably. Perhaps in 3 cells of a 1000 cells, the concentration of Substance A could be [X_A_ + 3σ_a_] while the concentration of Substance B could be [X_B_ - 3σ_b_]. If the cell responds to the ratio of [A]:[B], then the difference between cells would be magnified such as to evoke a changed response compared to an adjacent cell. If there was no compensation to this change in response so as to maintain homeostasis, then the difference, along with the original mutation, could cause a change in cell type to a cancer cell or this change may only occur following more such apparently symmetrical cell divisions. This is consistent with a continuum model for tumour suppression which reflects “subtle dosage effects”.

But cellular decision-making is multifactorial with two key determinants being the intensity of the internalized mitogenic information and the amount of available nutrients. For the former, IGF-I would be important in many cells for G2/M progression via its activation of PI3K/Akt/mTOR signalling. For the latter, the AMP-activated protein kinase functions to allow cells to cope with nutrient, oxygen and growth factor deprivations. It activates catabolic and inhibits anabolic metabolism and functions to maintain energy homeostasis, consistent with a tumor suppressor activity. But it acts to maintain bioenergetic homeostasis to support cell survival of both normal and cancer cells and may act as a tumor suppressor or as an oncogene dependent on context.

The proposed change in cell type (to a cancer cell) will depend in part on a Trefone (eg. IGF-I) signalling and on a deficiency of nutrients or metabolites necessary for normal cell metabolism or on cell exposure to unusual substances. The metabolites would be formed within the organism (as derivatives of nutrients) or sourced from outside (normal nutrients eg. oxygen and essential nutrients – vitamins, minerals, essential fatty acids, amino acids and carbohydrates). As an example of the effect of just one nutrient, a subpopulation of HT-29 cells will grow in the absence of glucose and, after confluence, exhibit enterocytic differentiation. But, with added glucose, the cells remain undifferentiated.

Variation in availability of derived carbohydrates (eg. N-Ac-glucosamine, N-Ac-galactosamine, sialic acid) or of coupling enzymes, may change the properties of the carbohydrates of glycoproteins. Glycosylation enzymes (eg. glycosyltransferases and glycosidases) are used as cancer biomarkers and most of the clinically useful cancer biomarkers are glycoproteins. Most membrane receptors, many binding proteins and some peptide Trefones are glycoproteins or glycosylated. This specific glycosylation occurs in the Golgi apparatus and would require specific transferases etc and adequate sources of normal and derived monosaccharides.

Perhaps a more critical metabolite is hydroxyglutarate. In many primary human brain cancers, mutations in the gene for the cytosolic enzyme isocitrate dehydrogenase 1 (IDH1) are common. The mutant enzyme has a gain-of-function and will catalyse the formation of R(-)-2-hydroxyglutarate (2HG) from α-ketoglutarate, dependent on NADPH. Significant amounts of 2HG are secreted extracellularly. 2HG is considered an oncometabolite in secondary gliomas and in acute myeloid leukemias. Mutant IDH1 or IDH2 causes in increase in 2-HG, which then leads to an aberrant histone modification profile and a decrease in 5-hydroxymethylcytosine causes epigenetic dysregulation.

In summary, a mutation producing abnormal growth factor signalling along with either a deficiency of an essential metabolite or an excess of an unusual metabolite may cause a change of cell type.

**(j) Leukemia, other Non-Solid (Liquid) Cancers and Asymmetric Cell Division**

Much of the emphasis above has been on solid tumours but cancers that are initially liquid are very common. Liquid tumors occur in the blood, bone marrow, and lymph nodes and include leukemia, lymphoma, and myeloma. Leukemia relates to an abnormal increase of progenitors of [white blood cells](http://en.wikipedia.org/wiki/White_blood_cell), lymphoma relates to the progenitors of B and T cells of the immune system and may become a solid tumour while myeloma relates to both a white blood cell (plasma cell) and the immune system.

Asymmetric division is known to regulate cell fate decisions in the mammalian hematopoietic system and plays a role in stem cell renewal but more importantly here, in leukemogenesis. The common myeloid progenitor (CMP) plus the common lymphocyte progenitor (CLP) are likely derived from the multipotent progenitor (MPP) by AsCD. Subsequent cell division of the CMP to produce the granulocyte-macrophage progenitor cells of many of the leucocytes would also likely be via AsCD. The regulation of the timing and relative amounts of AsCD and SCD determines the normal development and continuing function of the immune system. Abnormal AsCd or dysregulation of scheduled AsCD vs CD could lead to cancer in the hematopoietic system. Indeed, some view polarity regulators which are part of AsCD, as tumour suppressors! Again, disruption of polarity might alter the cells derived from AsCD and possibly initiate leukemia development.

AsCD occurs also in the generation of cells of the immune system – the T and B lymphocytes. The common lymphocyte progenitor (CLP) (likely derived from AsCD as mentioned above) produces the progenitor cells of the lymphocytes also likely via AsCD. T-lymphocyte precursors do divide by AsCD early in the adaptive immunity response to produce effector and memory lineage progenitors.

Precursor T-cell lymphoblastic lymphoma (T-ALL) originates during normal thymocyte development, so the initial oncogenic events may occur in the thymus. Therein, cell polarization and signalling pathways (eg. Notch receptors and Wnt ligands) are involved in development of T-ALL.

A model of “cooperativity of activating mutations” causing leukemia (esp. acute myelogenous leukemia, AML) defines two classes of mutations in another “two-hit” model. Class I mutations confers a proliferative and/or survival advantage to hematopoietic progenitors but does not affect differentiation. The cell receptor Fms-related tyrosine kinase 3 (FLT3) and signal transmitters N- and K-ras are included in this class. Class II mutations impair hematopoietic differentiation and subsequent apoptosis. Fusion genes eg. *PML/RARα* ([promyelocytic leukemia/ retinoic acid receptor alpha)](http://en.wikipedia.org/wiki/Promyelocytic_leukemia_gene) transcription factor and its blocker *AML1/ETO* (AML-1 transcription factor and the eight-twenty-one corepressor) are included in this class. Expression of both classes of mutations results in the AML phenotype but not all mutations fit neatly into these classes.

Overall, AsCD along with potential Trefones, receptors and their signalling pathways will be part of the etiology of non-solid tumours.

**(k) Breast Cancer**

Some normal breast luminal epithelial cells respond to estrogen (E) via estrogen receptors (ER) and are estrogen-receptor positive. Within the CTC model, E is a Trefone with SHBG as the partner Trefone of this couplet of Trefones. Some breast epithelial cells (eg. the a-Cells) would respond to E and the couplet cells (the i-Cells) will respond to SHBG. SHBG is not just an inhibitor of estrogen; SHBG membrane receptor is present in some breast cancer cell lines and patient tissues, it induces proliferation in a transfected breast cancer cell line, MCF-7, and SHBG itself is expressed in breast cancer cells.

The normal breast cells are then a collection of various couplet cells: some respond to E some to SHBG; some will respond to Progesterone (P) and some to its Trefone partner (Transcortin or CBG); some will respond to EGF via its receptor HER-2 and some to EGF’s couplet Trefone partner, soluble HER-2, the equivalent of an EGF binding protein. The couplet complexes will also be involved. For example, SHBG bound to receptor will bind estrogen to trigger downstream signalling and the CBG:progesterone complex stimulates a breast cancer cell line, MCF-7.

Breast epithelial cells become cancerous and it is known that some of these cancer cells will have receptors for E, some for P, some for both or neither, and some of these will be HER-2 positive. In the current model, some cells will also be positive for receptors for SHBG, CBG and/or soluble HER-2. A normal cell may become cancerous due to a mutation in one gene or more genes which affect any part of the intracellular transduction steps which lead to dysregulated cell division. Of course, the loss-of-function mutation in BRCA1, with loss of DNA repair of mutated or otherwise damaged cells, causes cancer but other DNA changes may lead to more selected loss of cell regulation now stimulated by the Trefones - E, P, EGF and/or their binding partners. According to this CTC model, the way forward in cancer treatment will be to discover inhibitors of the receptors for SHBG, CBG and soluble HER-2.

(Note that within the CTC model, ER-alpha/-beta and PR-alpha/-beta receptors may be intracellular or plasma-membrane bound.)

**REFERENCES:-**

1.	Hanahan D, Weinberg RA: **Hallmarks of cancer: the next generation**. *Cell* 2011, **144**(5):646-674.

2.	Sporn MB: **The war on cancer**. *Lancet* 1996, **347**(9012):1377-1381.

3.	Vineis P, Berwick M: **The population dynamics of cancer: a Darwinian perspective**. *Int J Epidemiol* 2006, **35**(5):1151-1159.

4.	Jablonka E: **Commentary: induction and selection of variations during cancer development**. *Int J Epidemiol* 2006, **35**(5):1163-1165.

5.	Pancione M, Remo A, Colantuoni V: **Genetic and epigenetic events generate multiple pathways in colorectal cancer progression**. *Patholog Res Int* 2012, **2012**:509348.

6.	You JS, Jones PA: **Cancer genetics and epigenetics: two sides of the same coin?** *Cancer Cell* 2012, **22**(1):9-20.

7.	Prensner JR, Chinnaiyan AM: **The emergence of lncRNAs in cancer biology**. *Cancer Discov* 2011, **1**(5):391-407.

8.	Bjedov I, Tenaillon O, Gerard B, Souza V, Denamur E, Radman M, Taddei F, Matic I: **Stress-induced mutagenesis in bacteria**. *Science* 2003, **300**(5624):1404-1409.

9.	Vogelstein B, Kinzler KW: **Cancer genes and the pathways they control**. *Nat Med* 2004, **10**(8):789-799.

10.	Cairns J, Overbaugh J, Miller S: **The origin of mutants**. *Nature* 1988, **335**(6186):142-145.

11.	Knudson AG: **Hereditary cancer: two hits revisited**. *J Cancer Res Clin Oncol* 1996, **122**(3):135-140.

12.	Bozic I, Antal T, Ohtsuki H, Carter H, Kim D, Chen S, Karchin R, Kinzler KW, Vogelstein B, Nowak MA: **Accumulation of driver and passenger mutations during tumor progression**. *Proc Natl Acad Sci U S A* 2010, **107**(43):18545-18550.

13.	Cairns J: **Mutation selection and the natural history of cancer**. *Nature* 1975, **255**(5505):197-200.

14.	Greenman C, Stephens P, Smith R, Dalgliesh GL, Hunter C, Bignell G, Davies H, Teague J, Butler A, Stevens C *et al*: **Patterns of somatic mutation in human cancer genomes**. *Nature* 2007, **446**(7132):153-158.

15.	Vogelstein B, Papadopoulos N, Velculescu VE, Zhou S, Diaz LA, Jr., Kinzler KW: **Cancer genome landscapes**. *Science* 2013, **339**(6127):1546-1558.

16.	Duesberg P, Fabarius A, Hehlmann R: **Aneuploidy, the primary cause of the multilateral genomic instability of neoplastic and preneoplastic cells**. *IUBMB Life* 2004, **56**(2):65-81.

17.	Rajagopalan H, Lengauer C: **Aneuploidy and cancer**. *Nature* 2004, **432**(7015):338-341.

18.	Michor F, Iwasa Y, Vogelstein B, Lengauer C, Nowak MA: **Can chromosomal instability initiate tumorigenesis?** *Semin Cancer Biol* 2005, **15**(1):43-49.

19.	Vincent TL, Gatenby RA: **An evolutionary model for initiation, promotion, and progression in carcinogenesis**. *Int J Oncol* 2008, **32**(4):729-737.

20.	Rajagopalan H, Jallepalli PV, Rago C, Velculescu VE, Kinzler KW, Vogelstein B, Lengauer C: **Inactivation of hCDC4 can cause chromosomal instability**. *Nature* 2004, **428**(6978):77-81.

21.	Bailly K, Soulet F, Leroy D, Amalric F, Bouche G: **Uncoupling of cell proliferation and differentiation activities of basic fibroblast growth factor**. *Faseb J* 2000, **14**(2):333-344.

22.	Irvin WJ, Jr., Carey LA: **What is triple-negative breast cancer?** *Eur J Cancer* 2008, **44**(18):2799-2805.

23.	Hynes NE, Dey JH: **Potential for targeting the fibroblast growth factor receptors in breast cancer**. *Cancer Res* 2010, **70**(13):5199-5202.

24.	Lengyel E, Sawada K, Salgia R: **Tyrosine kinase mutations in human cancer**. *Curr Mol Med* 2007, **7**(1):77-84.

25.	Zandi R, Larsen AB, Andersen P, Stockhausen MT, Poulsen HS: **Mechanisms for oncogenic activation of the epidermal growth factor receptor**. *Cell Signal* 2007, **19**(10):2013-2023.

26.	Chaffer CL, Dopheide B, Savagner P, Thompson EW, Williams ED: **Aberrant fibroblast growth factor receptor signaling in bladder and other cancers**. *Differentiation* 2007, **75**(9):831-842.

27.	Sporn MB, Todaro GJ: **Autocrine secretion and malignant transformation of cells**. *New England Journal of Medicine* 1980, **303**(15):878-880.

28.	Sporn MB, Roberts AB: **Autocrine growth factors and cancer**. *Nature* 1985, **313**(6005):745-747.

29.	Breuhahn K, Longerich T, Schirmacher P: **Dysregulation of growth factor signaling in human hepatocellular carcinoma**. *Oncogene* 2006, **25**(27):3787-3800.

30.	Kim JY, Hwang JH, Zhou W, Shin J, Noh SM, Song IS, Lee SH, Kim J: **The expression of VEGF receptor genes is concurrently influenced by epigenetic gene silencing of the genes and VEGF activation**. *Epigenetics* 2009, **4**(5):313-321.

31.	Kim SH, Park J, Choi MC, Park JH, Kim HP, Lee JH, Oh DY, Im SA, Bang YJ, Kim TY: **DNA methyltransferase 3B acts as a co-repressor of the human polycomb protein hPc2 to repress fibroblast growth factor receptor 3 transcription**. *Int J Biochem Cell Biol* 2008, **40**(11):2462-2471.

32.	Ullah Z, Lee CY, Depamphilis ML: **Cip/Kip cyclin-dependent protein kinase inhibitors and the road to polyploidy**. *Cell Div* 2009, **4**:10.

33.	Comai L: **The advantages and disadvantages of being polyploid**. *Nat Rev Genet* 2005, **6**(11):836-846.

34.	Wise DR, DeBerardinis RJ, Mancuso A, Sayed N, Zhang XY, Pfeiffer HK, Nissim I, Daikhin E, Yudkoff M, McMahon SB *et al*: **Myc regulates a transcriptional program that stimulates mitochondrial glutaminolysis and leads to glutamine addiction**. *Proc Natl Acad Sci U S A* 2008, **105**(48):18782-18787.

35.	Metallo CM, Gameiro PA, Bell EL, Mattaini KR, Yang J, Hiller K, Jewell CM, Johnson ZR, Irvine DJ, Guarente L *et al*: **Reductive glutamine metabolism by IDH1 mediates lipogenesis under hypoxia**. *Nature* 2012, **481**(7381):380-384.

36.	Dang L, White DW, Gross S, Bennett BD, Bittinger MA, Driggers EM, Fantin VR, Jang HG, Jin S, Keenan MC *et al*: **Cancer-associated IDH1 mutations produce 2-hydroxyglutarate**. *Nature* 2009, **462**(7274):739-744.

37.	Erenpreisa J, Huna A, Salmina K, Jackson TR, Cragg MS: **Macroautophagy-aided elimination of chromatin: sorting of waste, sorting of fate?** *Autophagy* 2012, **8**(12):1877-1881.

38.	Sundaram M, Guernsey DL, Rajaraman MM, Rajaraman R: **Neosis: a novel type of cell division in cancer**. *Cancer Biol Ther* 2004, **3**(2):207-218.

39.	Walen KH: **Mitosis is not the only distributor of mutated cells: non-mitotic endopolyploid cells produce reproductive genome-reduced cells**. *Cell Biol Int* 2010, **34**(8):867-872.

40.	Erenpreisa J, Cragg MS: **Cancer: a matter of life cycle?** *Cell Biol Int* 2007, **31**(12):1507-1510.

41.	Sell S, Leffert HL: **Liver cancer stem cells**. *J Clin Oncol* 2008, **26**(17):2800-2805.

42.	Loeb LA: **Mutator phenotype may be required for multistage carcinogenesis**. *Cancer Res* 1991, **51**(12):3075-3079.

43.	Loeb LA, Loeb KR, Anderson JP: **Multiple mutations and cancer**. *Proc Natl Acad Sci U S A* 2003, **100**(3):776-781.

44.	Braakhuis BJ, Tabor MP, Kummer JA, Leemans CR, Brakenhoff RH: **A genetic explanation of Slaughter's concept of field cancerization: evidence and clinical implications**. *Cancer Res* 2003, **63**(8):1727-1730.

45.	Perona R: **Cell signalling: growth factors and tyrosine kinase receptors**. *Clin Transl Oncol* 2006, **8**(2):77-82.

46.	Matsuhisa T: **Asymmetric cell division in human leukemic promyelocytic cells (HL-60)**. *Cell Biol Int* 1995, **19**(9):785-789.

47.	Pine SR, Ryan BM, Varticovski L, Robles AI, Harris CC: **Microenvironmental modulation of asymmetric cell division in human lung cancer cells**. *Proc Natl Acad Sci U S A* 2010, **107**(5):2195-2200.

48.	Bhowmick NA, Neilson EG, Moses HL: **Stromal fibroblasts in cancer initiation and progression**. *Nature* 2004, **432**(7015):332-337.

49.	Egeblad M, Nakasone ES, Werb Z: **Tumors as organs: complex tissues that interface with the entire organism**. *Dev Cell* 2010, **18**(6):884-901.

50.	Galdiero MR, Garlanda C, Jaillon S, Marone G, Mantovani A: **Tumor associated macrophages and neutrophils in tumor progression**. *J Cell Physiol* 2012.

51.	Mantovani A, Sica A: **Macrophages, innate immunity and cancer: balance, tolerance, and diversity**. *Curr Opin Immunol* 2010, **22**(2):231-237.

52.	Tecchio C, Scapini P, Pizzolo G, Cassatella MA: **On the cytokines produced by human neutrophils in tumors**. *Semin Cancer Biol* 2013, **23**(3):159-170.

53.	Qian BZ, Pollard JW: **Macrophage diversity enhances tumor progression and metastasis**. *Cell* 2010, **141**(1):39-51.

54.	Joyce JA, Pollard JW: **Microenvironmental regulation of metastasis**. *Nat Rev Cancer* 2009, **9**(4):239-252.

55.	Gabrilovich DI, Ostrand-Rosenberg S, Bronte V: **Coordinated regulation of myeloid cells by tumours**. *Nat Rev Immunol* 2012, **12**(4):253-268.

56.	Kalluri R, Zeisberg M: **Fibroblasts in cancer**. *Nat Rev Cancer* 2006, **6**(5):392-401.

57.	Su G, Blaine SA, Qiao D, Friedl A: **Shedding of syndecan-1 by stromal fibroblasts stimulates human breast cancer cell proliferation via FGF2 activation**. *J Biol Chem* 2007, **282**(20):14906-14915.

58.	Nikolova V, Koo CY, Ibrahim SA, Wang Z, Spillmann D, Dreier R, Kelsch R, Fischgrabe J, Smollich M, Rossi LH *et al*: **Differential roles for membrane-bound and soluble syndecan-1 (CD138) in breast cancer progression**. *Carcinogenesis* 2009, **30**(3):397-407.

59.	Gerlinger M, Rowan AJ, Horswell S, Larkin J, Endesfelder D, Gronroos E, Martinez P, Matthews N, Stewart A, Tarpey P *et al*: **Intratumor heterogeneity and branched evolution revealed by multiregion sequencing**. *N Engl J Med* 2012, **366**(10):883-892.

60.	Kaiser J: **The downside of diversity**. *Science* 2013, **339**(6127):1543-1545.

61.	Nowell PC: **The clonal evolution of tumor cell populations**. *Science* 1976, **194**(4260):23-28.

62.	Munoz J, Swanton C, Kurzrock R: **Molecular profiling and the reclassification of cancer: divide and conquer**. *Am Soc Clin Oncol Educ Book* 2013, **2013**:127-134.

63.	Fisher R, Pusztai L, Swanton C: **Cancer heterogeneity: implications for targeted therapeutics**. *Br J Cancer* 2013, **108**(3):479-485.

64.	Almendro V, Marusyk A, Polyak K: **Cellular heterogeneity and molecular evolution in cancer**. *Annu Rev Pathol* 2013, **8**:277-302.

65.	Jamal-Hanjani M, Thanopoulou E, Peggs KS, Quezada SA, Swanton C: **Tumour heterogeneity and immune-modulation**. *Curr Opin Pharmacol* 2013.

66.	Nieman KM, Kenny HA, Penicka CV, Ladanyi A, Buell-Gutbrod R, Zillhardt MR, Romero IL, Carey MS, Mills GB, Hotamisligil GS *et al*: **Adipocytes promote ovarian cancer metastasis and provide energy for rapid tumor growth**. *Nat Med* 2011, **17**(11):1498-1503.

67.	Martinez-Outschoorn UE, Balliet RM, Rivadeneira DB, Chiavarina B, Pavlides S, Wang C, Whitaker-Menezes D, Daumer KM, Lin Z, Witkiewicz AK *et al*: **Oxidative stress in cancer associated fibroblasts drives tumor-stroma co-evolution: A new paradigm for understanding tumor metabolism, the field effect and genomic instability in cancer cells**. *Cell Cycle* 2010, **9**(16):3256-3276.

68.	Morrison SJ, Kimble J: **Asymmetric and symmetric stem-cell divisions in development and cancer**. *Nature* 2006, **441**(7097):1068-1074.

69.	Lee CY, Robinson KJ, Doe CQ: **Lgl, Pins and aPKC regulate neuroblast self-renewal versus differentiation**. *Nature* 2006, **439**(7076):594-598.

70.	Bilder D, Li M, Perrimon N: **Cooperative regulation of cell polarity and growth by Drosophila tumor suppressors**. *Science* 2000, **289**(5476):113-116.

71.	Humbert P, Russell S, Richardson H: **Dlg, Scribble and Lgl in cell polarity, cell proliferation and cancer**. *Bioessays* 2003, **25**(6):542-553.

72.	Caussinus E, Gonzalez C: **Induction of tumor growth by altered stem-cell asymmetric division in Drosophila melanogaster**. *Nat Genet* 2005, **37**(10):1125-1129.

73.	Tomasetti C, Levy D: **Role of symmetric and asymmetric division of stem cells in developing drug resistance**. *Proc Natl Acad Sci U S A* 2010, **107**(39):16766-16771.

74.	Lathia JD, Hitomi M, Gallagher J, Gadani SP, Adkins J, Vasanji A, Liu L, Eyler CE, Heddleston JM, Wu Q *et al*: **Distribution of CD133 reveals glioma stem cells self-renew through symmetric and asymmetric cell divisions**. *Cell Death Dis* 2011, **2**:e200.

75.	Pavlides S, Whitaker-Menezes D, Castello-Cros R, Flomenberg N, Witkiewicz AK, Frank PG, Casimiro MC, Wang C, Fortina P, Addya S *et al*: **The reverse Warburg effect: aerobic glycolysis in cancer associated fibroblasts and the tumor stroma**. *Cell Cycle* 2009, **8**(23):3984-4001.

76.	Boidot R, Vegran F, Meulle A, Le Breton A, Dessy C, Sonveaux P, Lizard-Nacol S, Feron O: **Regulation of monocarboxylate transporter MCT1 expression by p53 mediates inward and outward lactate fluxes in tumors**. *Cancer Res* 2012, **72**(4):939-948.

77.	Whitaker-Menezes D, Martinez-Outschoorn UE, Lin Z, Ertel A, Flomenberg N, Witkiewicz AK, Birbe RC, Howell A, Pavlides S, Gandara R *et al*: **Evidence for a stromal-epithelial "lactate shuttle" in human tumors: MCT4 is a marker of oxidative stress in cancer-associated fibroblasts**. *Cell Cycle* 2011, **10**(11):1772-1783.

78.	Sotgia F, Whitaker-Menezes D, Martinez-Outschoorn UE, Salem AF, Tsirigos A, Lamb R, Sneddon S, Hulit J, Howell A, Lisanti MP: **Mitochondria "fuel" breast cancer metabolism: fifteen markers of mitochondrial biogenesis label epithelial cancer cells, but are excluded from adjacent stromal cells**. *Cell Cycle* 2012, **11**(23):4390-4401.

79.	Schwiebert EM, Zsembery A: **Extracellular ATP as a signaling molecule for epithelial cells**. *Biochim Biophys Acta* 2003, **1615**(1-2):7-32.

80.	Franke H, Illes P: **Involvement of P2 receptors in the growth and survival of neurons in the CNS**. *Pharmacol Ther* 2006, **109**(3):297-324.

81.	Erlinge D: **Extracellular ATP: a growth factor for vascular smooth muscle cells**. *Gen Pharmacol* 1998, **31**(1):1-8.

82.	White N, Burnstock G: **P2 receptors and cancer**. *Trends Pharmacol Sci* 2006, **27**(4):211-217.

83.	Rattigan YI, Patel BB, Ackerstaff E, Sukenick G, Koutcher JA, Glod JW, Banerjee D: **Lactate is a mediator of metabolic cooperation between stromal carcinoma associated fibroblasts and glycolytic tumor cells in the tumor microenvironment**. *Exp Cell Res* 2012, **318**(4):326-335.

84.	Furthauer M, Gonzalez-Gaitan M: **Endocytosis, asymmetric cell division, stem cells and cancer: unus pro omnibus, omnes pro uno**. *Mol Oncol* 2009, **3**(4):339-353.

85.	Cullen KJ, Smith HS, Hill S, Rosen N, Lippman ME: **Growth factor messenger RNA expression by human breast fibroblasts from benign and malignant lesions**. *Cancer Res* 1991, **51**(18):4978-4985.

86.	Yee D, Paik S, Lebovic GS, Marcus RR, Favoni RE, Cullen KJ, Lippman ME, Rosen N: **Analysis of insulin-like growth factor I gene expression in malignancy: evidence for a paracrine role in human breast cancer**. *Mol Endocrinol* 1989, **3**(3):509-517.

87.	Deng H, Lin Y, Badin M, Vasilcanu D, Stromberg T, Jernberg-Wiklund H, Sehat B, Larsson O: **Over-accumulation of nuclear IGF-1 receptor in tumor cells requires elevated expression of the receptor and the SUMO-conjugating enzyme Ubc9**. *Biochem Biophys Res Commun* 2011, **404**(2):667-671.

88.	Ribatti D, Nico B, Crivellato E: **The role of pericytes in angiogenesis**. *Int J Dev Biol* 2011, **55**(3):261-268.

89.	Witsch E, Sela M, Yarden Y: **Roles for growth factors in cancer progression**. *Physiology (Bethesda)* 2010, **25**(2):85-101.

90.	Cheng N, Chytil A, Shyr Y, Joly A, Moses HL: **Transforming growth factor-beta signaling-deficient fibroblasts enhance hepatocyte growth factor signaling in mammary carcinoma cells to promote scattering and invasion**. *Mol Cancer Res* 2008, **6**(10):1521-1533.

91.	Karnoub AE, Dash AB, Vo AP, Sullivan A, Brooks MW, Bell GW, Richardson AL, Polyak K, Tubo R, Weinberg RA: **Mesenchymal stem cells within tumour stroma promote breast cancer metastasis**. *Nature* 2007, **449**(7162):557-563.

92.	Kim R, Emi M, Tanabe K, Arihiro K: **Tumor-driven evolution of immunosuppressive networks during malignant progression**. *Cancer Res* 2006, **66**(11):5527-5536.

93.	Berger AH, Knudson AG, Pandolfi PP: **A continuum model for tumour suppression**. *Nature* 2011, **476**(7359):163-169.

94.	Liang J, Mills GB: **AMPK: a contextual oncogene or tumor suppressor?** *Cancer Res* 2013, **73**(10):2929-2935.

95.	Zweibaum A, Pinto M, Chevalier G, Dussaulx E, Triadou N, Lacroix B, Haffen K, Brun JL, Rousset M: **Enterocytic differentiation of a subpopulation of the human colon tumor cell line HT-29 selected for growth in sugar-free medium and its inhibition by glucose**. *J Cell Physiol* 1985, **122**(1):21-29.

96.	Meany DL, Chan DW: **Aberrant glycosylation associated with enzymes as cancer biomarkers**. *Clin Proteomics* 2011, **8**(1):7.

97.	Wang F, Travins J, DeLaBarre B, Penard-Lacronique V, Schalm S, Hansen E, Straley K, Kernytsky A, Liu W, Gliser C *et al*: **Targeted inhibition of mutant IDH2 in leukemia cells induces cellular differentiation**. *Science* 2013, **340**(6132):622-626.

98.	Prensner JR, Chinnaiyan AM: **Metabolism unhinged: IDH mutations in cancer**. *Nat Med* 2011, **17**(3):291-293.

99.	Congdon KL, Reya T: **Divide and conquer: how asymmetric division shapes cell fate in the hematopoietic system**. *Curr Opin Immunol* 2008, **20**(3):302-307.

100.	Hawkins ED, Russell SM: **Upsides and downsides to polarity and asymmetric cell division in leukemia**. *Oncogene* 2008, **27**(55):7003-7017.

101.	Chang JT, Palanivel VR, Kinjyo I, Schambach F, Intlekofer AM, Banerjee A, Longworth SA, Vinup KE, Mrass P, Oliaro J *et al*: **Asymmetric T lymphocyte division in the initiation of adaptive immune responses**. *Science* 2007, **315**(5819):1687-1691.

102.	Oliaro J, Van Ham V, Sacirbegovic F, Pasam A, Bomzon Z, Pham K, Ludford-Menting MJ, Waterhouse NJ, Bots M, Hawkins ED *et al*: **Asymmetric cell division of T cells upon antigen presentation uses multiple conserved mechanisms**. *J Immunol* 2010, **185**(1):367-375.

103.	Staal FJ, van Dongen JJ, Langerak AW: **Novel insights into the development of T-cell acute lymphoblastic leukemia**. *Curr Hematol Malig Rep* 2007, **2**(3):176-182.

104.	Gilliland DG, Griffin JD: **The roles of FLT3 in hematopoiesis and leukemia**. *Blood* 2002, **100**(5):1532-1542.

105.	Takahashi S: **Current findings for recurring mutations in acute myeloid leukemia**. *J Hematol Oncol* 2011, **4**:36.

106.	Fortunati N, Becchis M, Catalano MG, Comba A, Ferrera P, Raineri M, Berta L, Frairia R: **Sex hormone-binding globulin, its membrane receptor, and breast cancer: a new approach to the modulation of estradiol action in neoplastic cells**. *J Steroid Biochem Mol Biol* 1999, **69**(1-6):473-479.

107.	Fortunati N, Raineri M, Cignetti A, Hammond GL, Frairia R: **Control of the membrane sex hormone-binding globulin-receptor (SHBG-R) in MCF-7 cells: effect of locally produced SHBG**. *Steroids* 1998, **63**(5-6):282-284.

108.	Becchis M, Frairia R, Ferrera P, Fazzari A, Ondei S, Alfarano A, Coluccia C, Biglia N, Sismondi P, Fortunati N: **The additionally glycosylated variant of human sex hormone-binding globulin (SHBG) is linked to estrogen-dependence of breast cancer**. *Breast Cancer Res Treat* 1999, **54**(2):101-107.

109.	Carney WP, Bernhardt D, Jasani B: **Circulating HER2 Extracellular Domain: A Specific and Quantitative Biomarker of Prognostic Value in all Breast Cancer Patients?** *Biomark Cancer* 2013, **5**:31-39.

110.	Fazzari A, Catalano MG, Comba A, Becchis M, Raineri M, Frairia R, Fortunati N: **The control of progesterone receptor expression in MCF-7 breast cancer cells: effects of estradiol and sex hormone-binding globulin (SHBG)**. *Mol Cell Endocrinol* 2001, **172**(1-2):31-36.

111.	Nakhla AM, Khan MS, Rosner W: **Induction of adenylate cyclase in a mammary carcinoma cell line by human corticosteroid-binding globulin**. *Biochem Biophys Res Commun* 1988, **153**(3):1012-1018.
